# Photocontrolled Cobalt Catalysis for Selective Hydroboration of α,β‐Unsaturated Ketones

**DOI:** 10.1002/anie.202009893

**Published:** 2020-09-11

**Authors:** Frédéric Beltran, Enrico Bergamaschi, Ignacio Funes‐Ardoiz, Christopher J. Teskey

**Affiliations:** ^1^ Institute of Organic Chemistry RWTH Aachen University Landoltweg 1 52074 Aachen Germany

**Keywords:** homogeneous catalysis, cobalt, DFT calculations, hydroboration, selectivity

## Abstract

Selectivity between 1,2 and 1,4 addition of a nucleophile to an α,β‐unsaturated carbonyl compound has classically been modified by the addition of stoichiometric additives to the substrate or reagent to increase their “hard” or “soft” character. Here, we demonstrate a conceptually distinct approach that instead relies on controlling the coordination sphere of a catalyst with visible light. In this way, we bias the reaction down two divergent pathways, giving contrasting products in the catalytic hydroboration of α,β‐unsaturated ketones. This includes direct access to previously elusive cyclic enolborates, via 1,4‐selective hydroboration, providing a straightforward and stereoselective route to rare syn‐aldol products in one‐pot. DFT calculations and mechanistic experiments confirm two different mechanisms are operative, underpinning this unusual photocontrolled selectivity switch.

## Graphical abstract

A single catalytic platform is described for which control of 1,2 versus 1,4 selectivity for enone hydroboration can be controlled by light. This was used to obtain rare *syn*‐aldol products on cyclic substrates. Experiments and DFT calculations demonstrate that two distinctive mechanisms are operative.

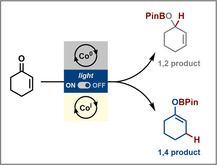

## Introduction

**Scheme 1:**
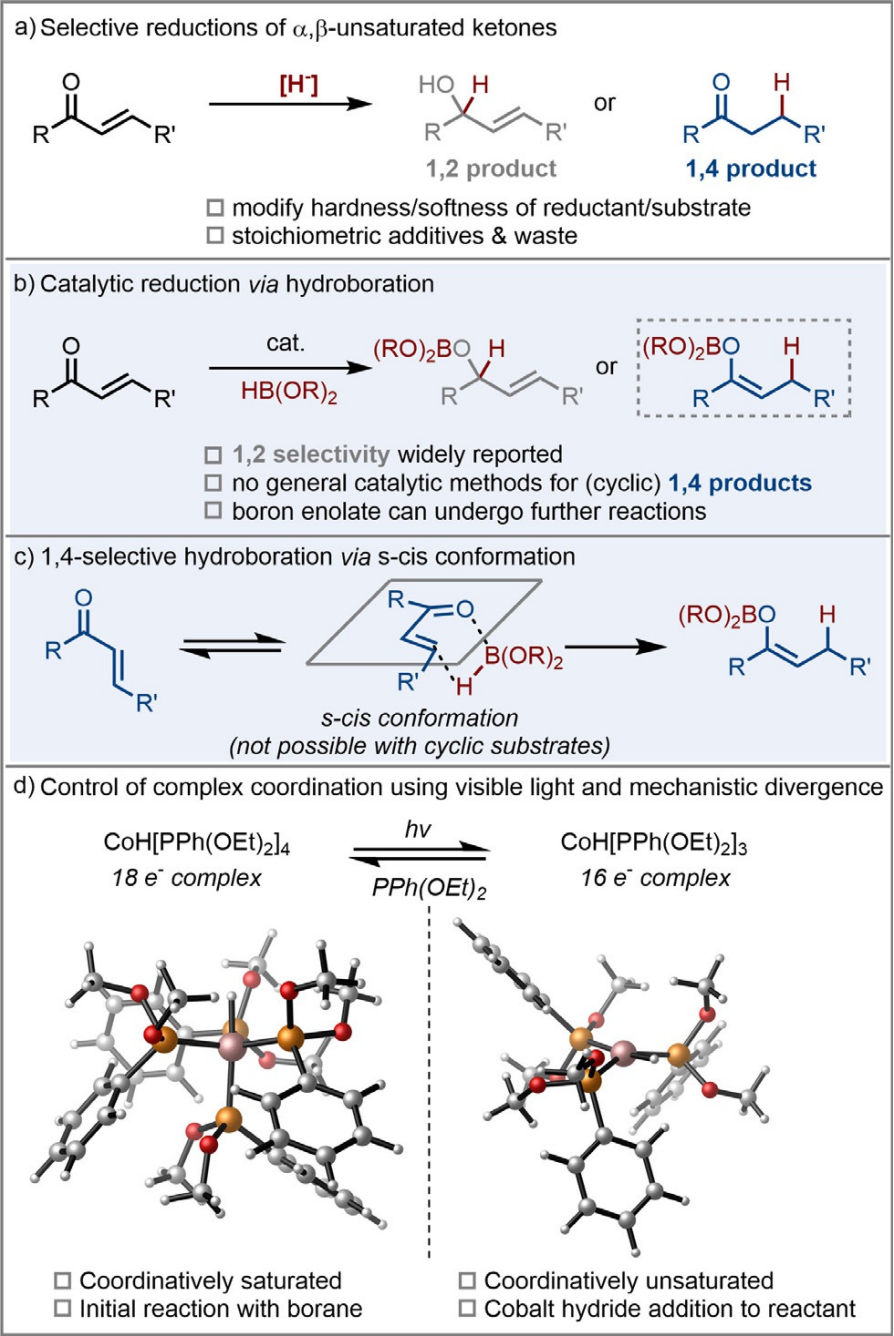
Context of this work: selective reduction and hydroboration of α,β‐unsaturated ketones.

The selective reduction of α,β‐unsaturated carbonyl compounds is a transformation of widespread importance in synthetic chemistry and the underlying reactivity principles have made it into well‐established text‐book knowledge.^[1]^ In this context, the decisive factor underpinning selectivity is the concept of hardness and softness, of the nucleophile/reducing agent and the reactant, which dictate whether attack occurs at the carbonyl (1,2‐addition) or the β‐carbon (1,4‐addition), respectively (Scheme 1 a).[^2^, ^3^, ^4^] Although significant progress has been made with the use of metal‐additives to tune the hardness/softness of classical reducing agents, challenges still exist in this area.

Catalytic hydroboration is considered to be a mild method of reduction, relying on commercially available boranes and displaying increased functional group tolerance and selectivity when compared with traditional reducing agents.[^5^, ^6^, ^7^] A broad range of mechanistic pathways have been reported,^[8]^ however the vast majority of methods for α,β‐unsaturated ketones are 1,2‐selective (Scheme 1 b).[^9^, ^10^, ^11^, ^12^] 1,4‐selective methods have been restricted to linear substrates and do not work on cyclic α,β‐unsaturated ketones which cannot access the required reactive s‐cis conformation (Scheme 1 c).[^13^, ^14^, ^15^] A method to address this significant limitation would be extremely valuable, enabling regioselective formation of cyclic enolborates which give contrasting stereoselectivity in aldol reactions to their enolborinate and silyl enol ether counterparts.[^16^, ^17^, ^18^]

With the recent attention directed towards the use of light to control reactivity[^19^, ^20^, ^21^] and selectivity[^22^, ^23^, ^24^] in catalytic reactions, we became interested in developing a method which, in contrast to the established hardness/softness reactivity concepts, allowed for a fundamental selectivity reversal by simple use of visible light. This would enable us to exert precise control over reactions with a non‐invasive, external stimulus and avoid the need for any stoichiometric additives.

In the field of transition metal catalysis, ligand photodissociation has been shown as a method to switch a reaction between on and off states by revealing ligation sites at a metal centre. One such example is the 18 electron earth‐abundant transition‐metal complex, CoH[PPh(OEt)_2_]_4_ which undergoes photodissociation with visible light to generate 16 electron complex, CoH[PPh(OEt)_2_]_3_.[^25^, ^26^]

It can be seen from the optimised structures^[27]^ that any coordinative interaction of a substrate with the metal centre of CoH[PPh(OEt)_2_]_4_ appears improbable, whereas in contrast, the corresponding complex generated upon light irradiation, CoH[PPh(OEt)_2_]_3_, has a vacant coordination site (Scheme 1 d). Despite this, we have recently reported a catalytic system based on the use of CoH[PPh(OEt)_2_]_4_ in conjunction with pinacolborane for alkene isomerisation, indicating the potential for a different mechanistic scenario.^[28]^ We therefore hypothesised that if two different mechanistic pathways, controlled only by the presence or absence of light, are operative, this may lead to contrasting selectivity in the context of catalytic hydroboration of α,β‐unsaturated carbonyls. In addition, this divergent mechanistic control may obviate the requirement of s‐*cis* conformation to perform 1,4‐hydroboration, leaving a single catalytic platform able to carry out both 1,2 and 1,4‐hydroboration of linear and, the previously unsolved, cyclic unsaturated ketones.

## Results and Discussion

**Scheme 2:**
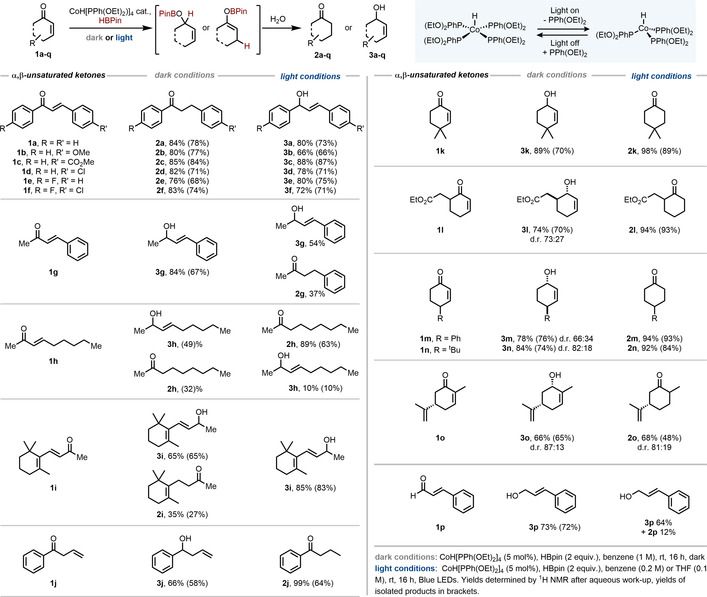
Substrate scope of the photocontrolled cobalt‐catalysed hydroboration of α,β‐unsaturated ketones.

Our investigations began using 5 mol % of CoH[PPh(OEt)_2_]_4_ in conjunction with pinacolborane for the reduction of chalcone **1 a**. In benzene solvent and in the dark, product **2 a** was obtained upon quenching the reaction with water, selectivity in line with previous reports of hydroboration with catechol borane.^[14]^ Interestingly, upon irradiating the reaction with blue light, a complete switch in selectivity was noted, yielding the allylic alcohol, **3 a**. A scope of this reaction was then carried out to probe the generality of this light controlled, regioselective reduction procedure (Scheme 2).

Chalcones containing both electron rich (**1 b**) and electron poor arenes (**1 c**–**1 f**) were selectively reduced under light or dark reaction conditions, displaying precise control over selectivity. Notably, the ester functionality of **1 c** was untouched despite using excess pinacolborane. Upon changing from a phenyl ketone to a methyl ketone with β‐phenyl substitution, the selectivity unexpectedly changed in the dark to yield the allylic alcohol, **2 g** in good yield which we initially attributed to the conformational preference of the starting material. However, substrates **1 h** and **1 i** gave a mixture of products under the “dark conditions”. In contrast, substrate **1 g** underwent non‐selective hydroboration in the light whereas substrates **1 h** and **1 i** gave selective, although contrasting 1,4‐ and 1,2‐products, respectively.

Starting material **1 j**, with a methylene group between the alkene and ketone functionality, was reduced in a 1,2 fashion in the dark to give **3 j** as the major product. Notably we obtained product **2 j** when the reaction was carried out in the light which we believe arises from the following sequence: isomerisation of the olefin to give the α,β‐unsaturated ketone, 1,4‐selective hydroboration and protonation. Previous studies have established that this cobalt complex is able to isomerise alkenes under light irradiation.^[25]^

As we had shown that light was able to switch the reaction outcome on a range of linear substrates, we therefore then turned to more challenging cyclic α,β‐unsaturated ketone substrates. The inability of these substrates to undergo 1,4 selective hydroboration to initially form the boron enolate, had been highlighted as a restriction in previous methodologies.[^14^, ^29^, ^30^] With 4,4‐dimethylcyclohex‐2‐en‐1‐one, **1 k**, as the starting material in benzene, using pinacolborane in the dark, we observed 1,2‐selective reduction product **3 k** upon quenching with water—selectivity that has been reported for a broad range of catalysts.[^5^, ^8^] Upon carrying out the identical reaction in the light, however, we were delighted to obtain the product **2 k** from conjugate reduction. This unusual control of regioselectivity using only light as an external stimulus appeared to offer an excellent route to the desired boron enolates. Furthermore, this showcases the concept of using light to control the catalytic pathway and thus the selectivity of a hydridic reagent, rather than stoichiometric quantities of additives which generate significant waste. Further optimisation (see the Supporting Information for details) demonstrated that in the light, the saturated ketone was obtained with even higher yield when the reaction was carried out in THF.

We carried out a scope of the light‐switchable reduction under the optimised conditions. Starting material **1 l**, containing an ester group, showed complete selectivity for enone reduction under both sets of conditions, yielding either **3 l** or **2 l** selectively. Cyclohexenone rings substituted at the γ‐position (**1 m** & **1 n**) were also suitable substrates for these reactions (albeit showing a mixture of diastereomers in the dark). Carvone, **1 o**, is a challenging substrate to selectively hydroborate due to the presence of the electron rich alkene. Previously reported methods which rely on more reactive alkylboranes would also hydroborate this functionality, however under our conditions, this handle remains untouched to yield either **2 o** or **3 o** in the light or dark, respectively. We also sought to see if this reactivity was also applicable to α,β‐unsaturated aldehydes, however, substrate **1 p** gave only the 1,2‐reduced product in the dark with this also being the major product in the light.

Our next step sought to build upon the new reactivity discovered in the light by reacting the boron enolates with other electrophiles. Lipshutz and Papa have reported a one‐pot reductive aldol reaction using air‐sensitive Stryker's reagent and in situ generated diethyl borane which leads to *anti*‐selective aldol products for cyclic substrates.^[15]^ Similarly, there are limited reports on precious metal hydride catalysis for reductive aldol reactions which are applicable to cyclic enone substrates, though these again favour the *anti*‐products.^[31]^

Unlike for linear substrates, where control of enolate geometry is the major factor for controlling aldol stereoselectivity, cyclic substrates require a change of enolate. Methods to generate the *syn*‐aldol products have relied on tin,^[32]^ zirconium^[33]^ or titanium^[34]^ enolates, however there have also been previous reports that enolborates favour *syn*‐aldol products in direct contrast to enolborinates.[^16^, ^17^, ^18^] Previous difficulties in directly generating these starting materials had limited the practical utility of the method hence we were attracted to the possibility of using our chemistry in this context.

**Scheme 3:**
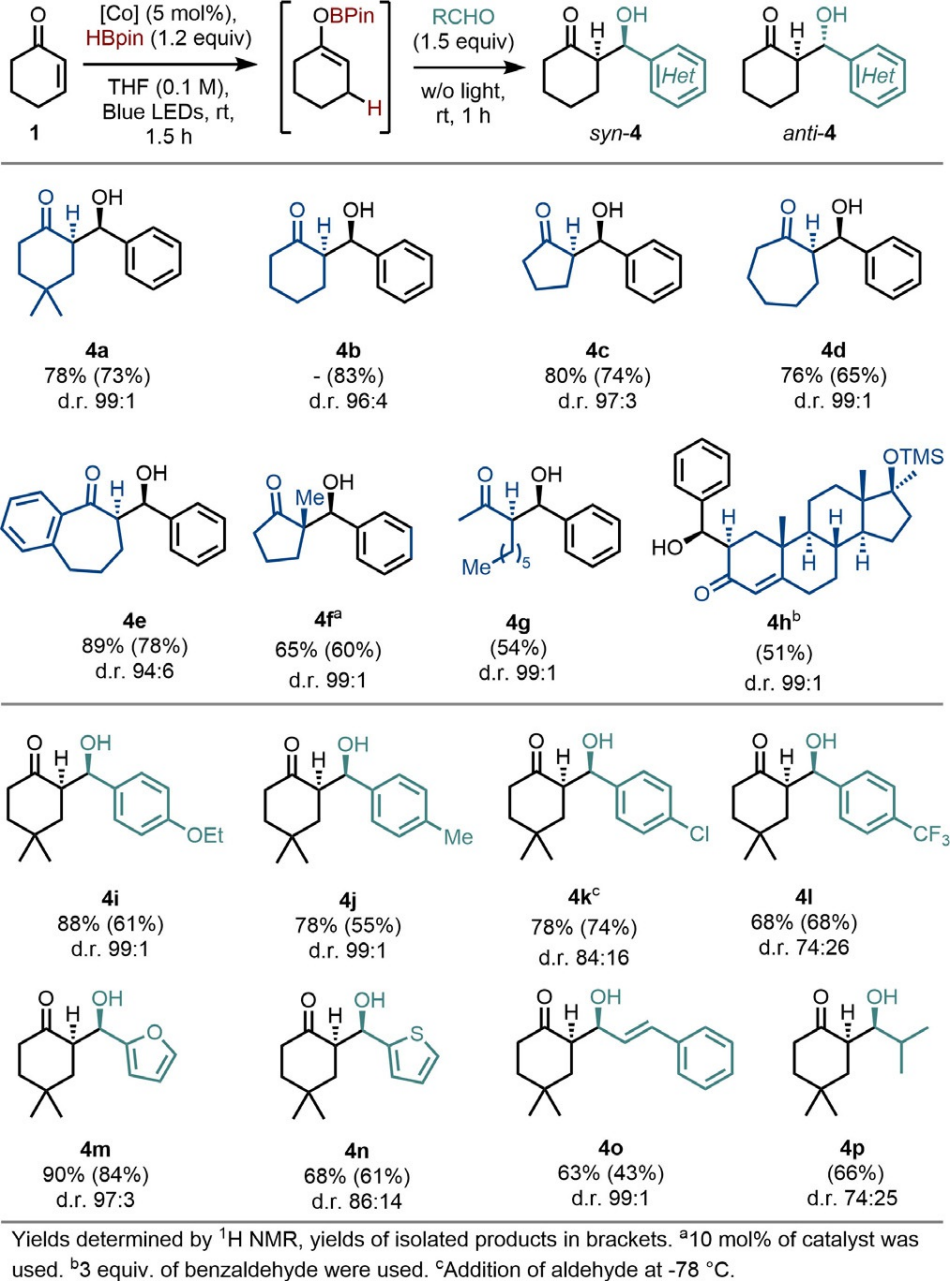
*Syn*‐selective aldol reactions with cyclic substrates.

Pleasingly, upon addition of 1.5 equivalents of benzaldehyde to the hydroborated intermediate, we obtained product **4 a** product in excellent 78 % yield with only the *syn*‐isomer observable, in direct contrast to the previous copper hydride catalysed method. A range of cyclic enone substrates with different ring sizes were suitable substrates for this one‐pot aldol reaction (**4 b**–**4 e**), with the *syn*‐diastereomer being produced with excellent selectivity in almost all cases (Scheme 3). Notably, we were able to form a quaternary centre (**4 f**) from an α‐substituted starting material, however this gave almost exclusively the *anti*‐product, in line with previous literature.^[17]^ Linear starting material also gave *syn*‐aldol product **4 g**. Application of this methodology to a more complex substrate derived from Metandienone (Dianabol), enabled a site selective functionalisation to give product **4 h**. Significantly, only the less‐hindered 1,4‐site underwent hydroboration and no products were observed from 1,2‐hydroboration.

We then turned to the scope of the aldehydes that could be used in the reaction. Notably, electron rich aldehydes gave excellent d.r. (**4 i** & **4 j**) with only the *syn*‐diastereomer detected. More electron deficient aldehydes were less selective though the yields were still good (**4 k** & **4 l**) and heterocyclic aldehydes worked well (**4 m** & **4 n**). The use of cinnamaldehyde gave a completely *syn*‐selective product (**4 o**) whereas the selectivity was lower using aliphatic isobutyraldehyde, **4 p**.

**Scheme 4:**
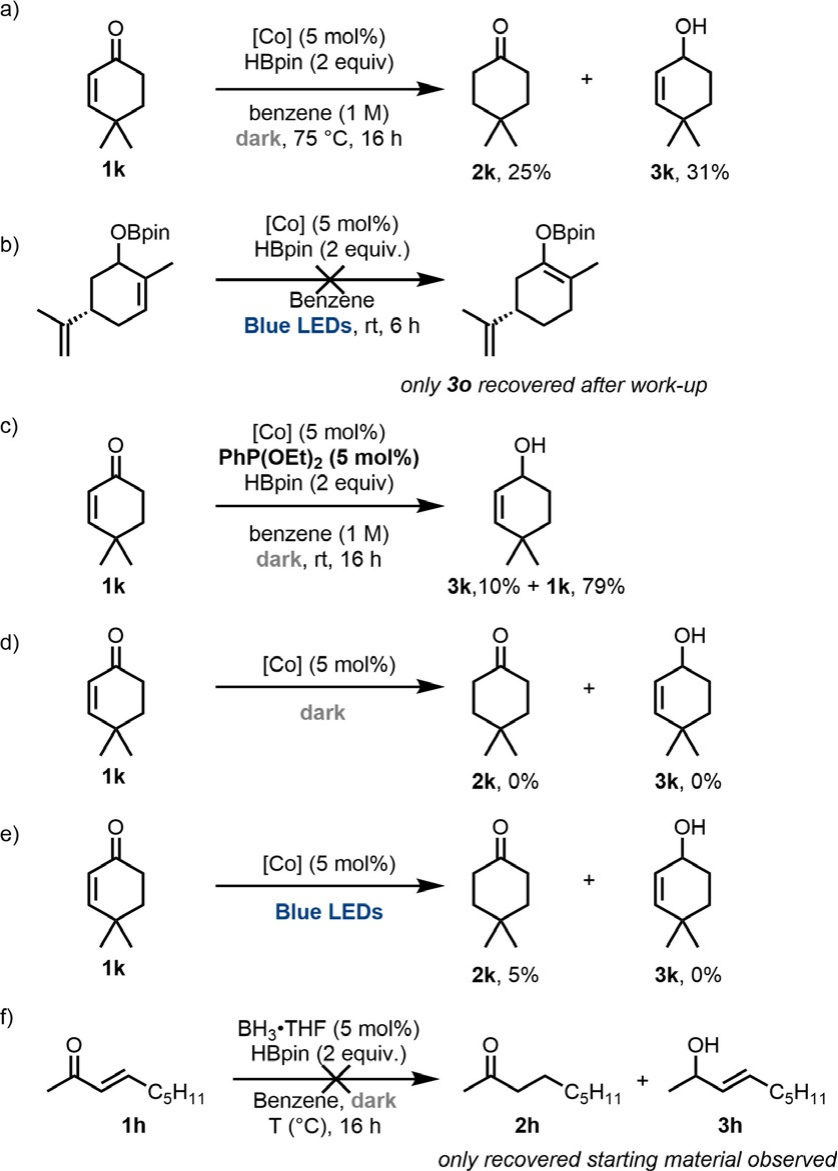
Mechanistic probes. [Co]=CoH[PPh(OEt)_2_]_4_.

Having established this platform for selectively accessing a broad range of hydroborated enone products and demonstrated their application in the aldol reaction, our attention turned to the mechanism and understanding the observed reactivity. Heating the reaction, rather than irradiating it with light increased the proportion of 1,4‐reduced product (Scheme 4 a). However, the selectivities and yields obtained failed to match those obtained under light irradiation, demonstrating the unique ability of light to promote 1,4‐selective hydroboration under mild conditions.

We next sought to rule out a mechanism whereby the light promoted selectivity arose from sequential hydroboration and isomerisation. To this end, the product arising from 1,2‐hydroboration of substrate **1 o** was irradiated with blue light in the presence of catalytic CoH[PPh(OEt)_2_]_4_ and pinacolborane but no 1,4‐hydroborated product was produced (Scheme 4 b), providing strong evidence against such a scenario. Next, to probe the role of “freed” phosphonite ligand, we carried out the reaction in the dark with an additional 5 mol % of PPh(OEt)_2_. In this case, we observed significantly decreased reactivity but no change in the selectivity (Scheme 4 c).

In order to shed further light onto the mechanistic differences between the dark (saturated 18 electron cobalt complex) and light (unsaturated 16 electron cobalt complex), we analysed the mechanism by means of DFT calculations which have emerged as a powerful methodology to analyse 3d transition metal catalysis.^[35]^ Computations were carried out at the CPCM(benzene)/B3LYP‐D3/Def2TZVPP//B3LYP‐D3/‐ 6‐31G(d)/LANL2DZ level of theory (see the Supporting Information for more information).

It is well‐established that light promotes phosphonite dissociation in this complex and, in almost all previously reported cases, the dark species has been considered inactive.[^25^, ^36^] Direct comparison of the optimised structures of both saturated and unsaturated catalyst (Scheme 1 d), demonstrates that the available space for substrate coordination is completely different: while in the unsaturated species a substrate can directly coordinate to the cobalt centre, there is no space available in the saturated one. Therefore, given the difference in this starting point, it would be expected that vastly different mechanistic pathways occur.

Furthermore, experiments carried out in absence of pinacolborane strongly suggest that a different intrinsic mechanism must be operative: no reduced products are detected in the dark (Scheme 4 d), indicating a mechanism reliant on activation of pinacolborane. However, in the light, 5 % of reduced product **2 k** was observed after aqueous work‐up, in absence of pinacolborane (Scheme 4 e), which is consistent with the borane being required only to turn over the catalytic cycle in the final steps, after generation of a cobalt enolate species. This also correlates with previous literature suggesting that cobalt hydrides are “soft” in character.[^37^, ^38^]

**Figure 1:**
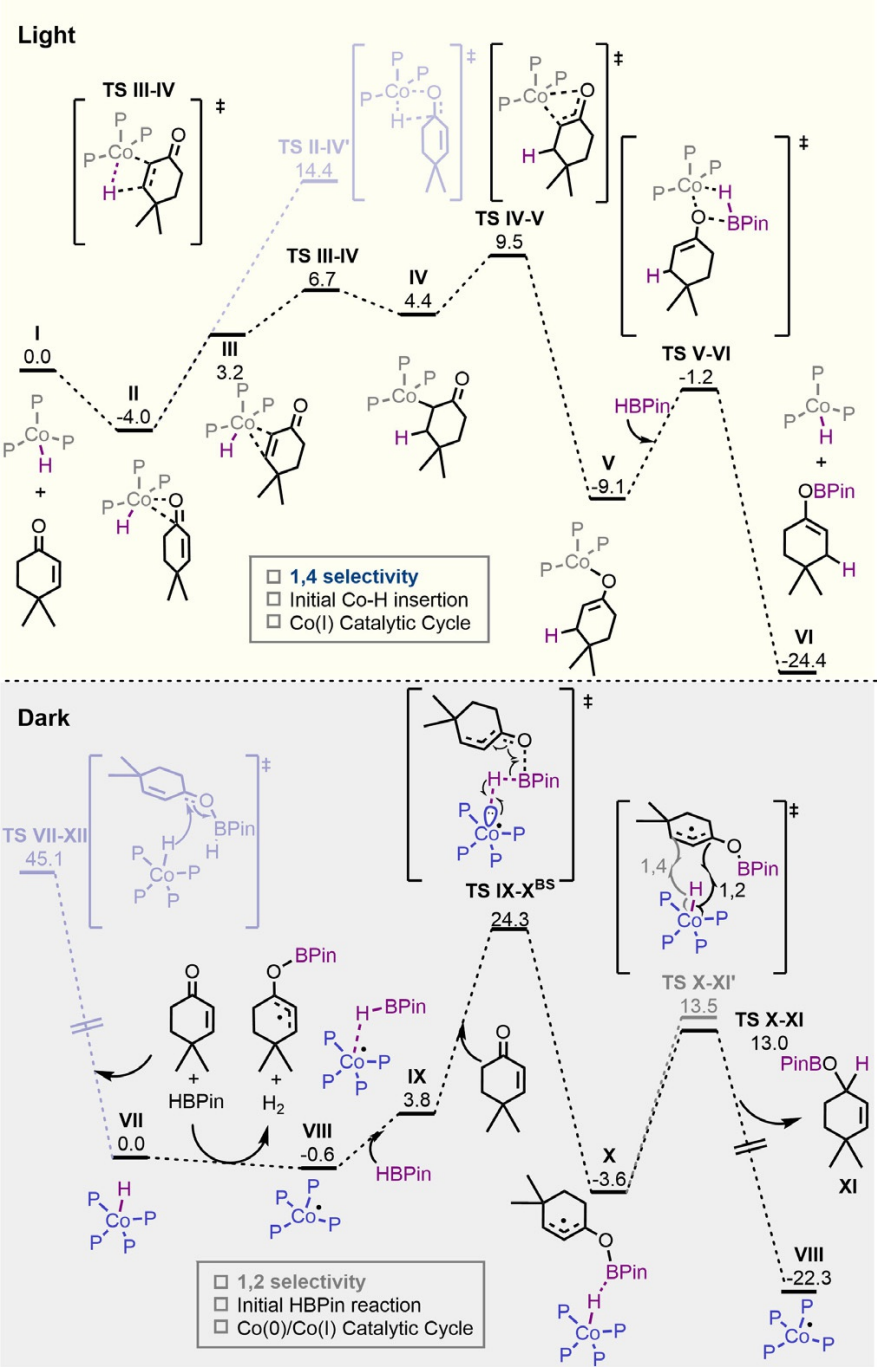
Free‐energy profiles of cyclohexanone hydroboration under light (top) and dark (bottom) conditions (P=PPh(OMe)_2_). Energies given in kcal mol^−1^.

We began our computational investigation of the light‐mediated catalytic reaction considering the unsaturated 16 e^−^ species **I** as the starting point (Figure 1) and substrate **1 k** as the model reaction. First, coordination of the substrate can take place in two different orientations, one through the carbonyl moiety (**II**) and the other through the double bond (**III**). Although coordination via the carbonyl group is largely favoured, the hydride migration into the C=O bond, though still thermally accessible at room temperature, is significantly higher in energy than into the C=C bond (18.4 kcal mol^−1^ vs. 10.7 kcal mol^−1^). This difference can be explained in terms of the highly distorted 4‐membered ring transition state in the case of C=O insertion, while in the case of C=C insertion, good conjugation of the resulting formal carbanion with the carbonyl facilitates the Co−C bond formation. From this point, keto‐enol tautomerisation takes place from **IV** with an overall barrier of 13.5 kcal mol^−1^, forming intermediate **V** exergonically. This result is in agreement with the experimental observation depicted in Scheme 4 e, since HBPin is not needed to promote the reaction up to this point and intermediate **V** can easily proceed to the 1,4 reduced product during work‐up. In the presence of HBPin, intermediate **V** easily evolves towards the final boron‐enolate and Co^I^‐H, completing the catalytic cycle.^[39]^

Our attention then turned to the more challenging dark mechanism. Monitoring of the reaction by ^1^H, ^11^B and ^31^P NMR revealed no new cobalt hydride species during the course of the reaction and no evidence of phosphonite ligand dissociation or modification occurring. We observed small traces of BH_3_ by ^11^B NMR appear after several hours,^[40]^ but replacing the cobalt complex with catalytic BH_3_⋅THF under our conditions did not prove effective in generating hydroborated products (Scheme 4 f).

Therefore, with this information in hand, we initially explored the direct outer sphere transfer of hydride to the substrate with the assistance of HBPin (**TS VII**–**XII**) (without the assistance of HBPin the reaction is even more endergonic).^[41]^ Interestingly, the barrier is extremely high (45.1 kcal mol^−1^) and that is in agreement with the fact that no product is detected when HBPin is not used in the reaction mixture (Scheme 4 d). Intriguingly, we observed experimentally, that after addition of HBPin to the catalyst, an NMR signal corresponding to H_2_ is clearly observed, suggesting a first reduction step of Co^I^ to Co^0^ promoted by the reaction mixture. In order to clarify that point, the Co^I^‐H to Co^0^ equilibrium was explored computationally and surprisingly, the reduction is exergonic even with the concomitant formation of a radical species (**VII** to **VIII**).^[42]^ Encouraged by this finding, we calculated an alternative pathway based on a Co^0^‐Co^I^ electron transfer mediated catalytic cycle.

**Figure 2:**
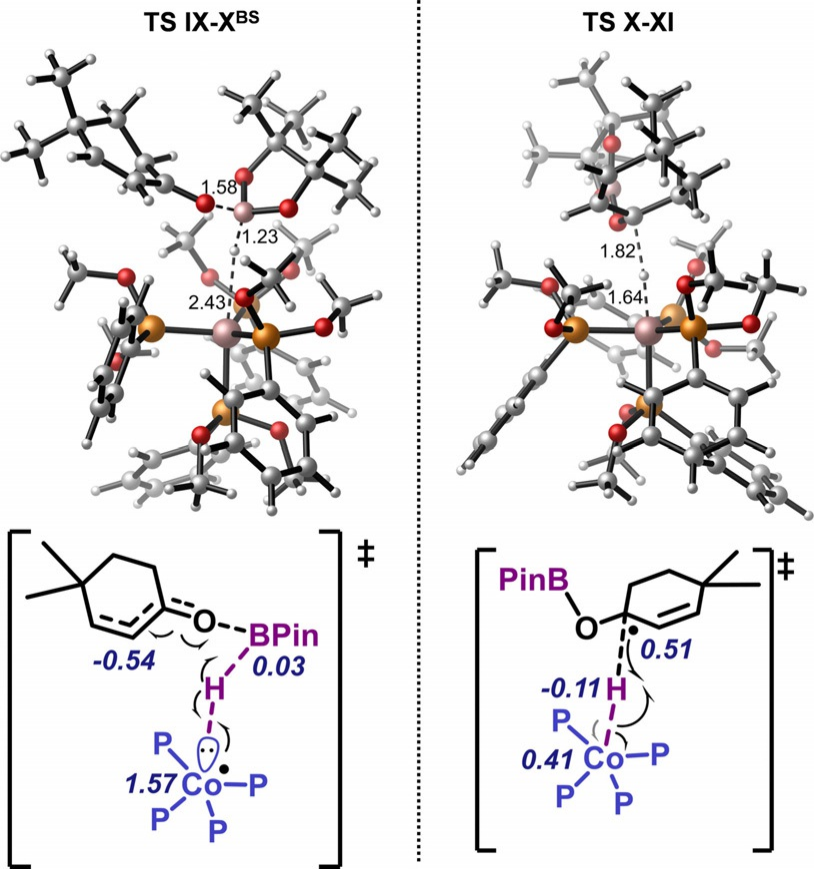
Optimised structures of **TS IX‐X** (left) and **TS X‐XI** (right). Relevant bond lengths (top) and spin densities (bottom) are shown. BS=broken symmetry.

Once **VIII** is formed, Co^0^ complex can interact with HBPin to form **IX** that is then activated with the assistance of the cyclohexanone substrate through **TS IX**‐**X^BS^** (Figure 2, left). This is the rate determining step of the reaction, where a single electron transfer from a lone pair of the cobalt centre to the HBPin occurs, forming Co^I^‐H through a hydrogen atom abstraction. BPin radical is simultaneously trapped by the oxygen of the substrate, forming a conjugated radical (**X**). This step is exergonic by 3.0 kcal mol^−1^ respect to intermediate **VIII**, and the overall barrier is only 24.9 kcal mol^−1^, more than 20 kcal mol^−1^ lower than the outer sphere Co^I^‐H attack. The reason behind the low energy of this pathway lies in the broken‐symmetry electronic structure (see the Supporting Information for further details). Finally, OBPin allyl radical **X** reacts with Co^I^‐H via hydrogen atom transfer, regenerating Co^0^ and substrate **XI** through transition state **TS X‐XI** (Figure 2, right). Interestingly, 1,2 recombination is faster than 1,4, which is in agreement with the experimental observations, although the predicted ratio (70:30) slightly differs to the observed one (only 1,2). This difference may arise from the use of methoxy groups instead of ethoxy in the phosphonite ligand.

## Conclusion

In conclusion, we have developed a catalytic platform for the hydroboration of α,β‐unsaturated ketones. This system relies on a bench‐stable, earth‐abundant cobalt hydride catalyst, uses commercially available pinacolborane, occurs at room temperature, and showcases a unique ability to control the regioselectivity depending on the presence or absence of light. As a result, we are able to access a broad range of products using just one reaction system, including access to cyclic boron enolates of which we have demonstrated the utility for *syn*‐selective aldol reactions. Experimental and computational experiments demonstrate that two distinct mechanistic pathways are operative in the dark and light which provides an explanation for the selectivities observed. Furthermore, it highlights the significant role that the coordination sphere of a metal complex can have on reactivity and selectivity and the underexplored potential that exists in controlling this with light.

## Conflict of interest

The authors declare no conflict of interest.

## Supporting information

As a service to our authors and readers, this journal provides supporting information supplied by the authors. Such materials are peer reviewed and may be re‐organized for online delivery, but are not copy‐edited or typeset. Technical support issues arising from supporting information (other than missing files) should be addressed to the authors.

Supplementary
