# Supplementary material for: Photocontrolled Cobalt Catalysis for Selective Hydroboration of α,β‐Unsaturated Ketones
Source: Angew Chem Int Ed Engl. 2020 Sep 11;59(47):21176–82. doi: 10.1002/anie.202009893 (PMC7692884; doi:10.1002/anie.202009893)
Supplement: Supplementary file 1 — Supplementary [file ANIE-59-21176-s001.pdf]

Angewandte Chemie
Eine Zeitschrift der Gesellschaft Deutscher Chemiker

Supporting Information

# Photocontrolled Cobalt Catalysis for Selective Hydroboration of α,β-Unsaturated Ketones

*Frédéric Beltran[+], Enrico Bergamaschi[+], Ignacio Funes-Ardoiz, and Christopher J. Teskey**

anie_202009893_sm_miscellaneous_information.pdf

# Table of Contents

**1. Materials and Methods ........ 5**

**2. Synthesis and Characterization Data of $CoH[PPh(OEt)_2]_4$ ........ 5**

**3. Selected optimization for the reduction of cyclic enones ........ 7**

**4. Preparation of starting materials ........ 8**

4.1 Procedure for the synthesis of 1-phenylbut-3-en-1-one 1j ........ 8

4.2 Procedure for the synthesis of ethyl 2-(2-oxocyclohex-3-en-1-yl)acetate (1l) ........ 8

4.3 General procedure for the synthesis of enones: Saegusa-Ito oxidation of cycloalkanones (Procedure A) ........ 9

2,3-dihydro-[1,1'-biphenyl]-4(1H)-one (1m) ........ 9

4-(tert-butyl)cyclohex-2-en-1-one (1n) ........ 9

8,9-dihydro-5H-benzo[7]annulen-5-one (S1) ........ 10

4.4 Procedure for the TMS protection of the steroid S2 to give (8R,9S,10R,13S,14S,17S)-10,13,17-trimethyl-17-((trimethylsilyl)oxy)-6,7,8,9,10,11,12,13,14,15,16,17-dodecahydro-3H-cyclopenta[a]phenanthren-3-one (S3) ........ 10

**5. General procedures and characterization data of reduction products ........ 11**

5.1 General procedure 1 ........ 11

5.2 General procedure 2 ........ 11

5.3 General procedure 3 ........ 11

5.4 General procedure 4 ........ 12

1,3-diphenylpropan-1-one (2a) ........ 12

3-(4-methoxyphenyl)-1-phenylpropan-1-one (2b) ........ 12

Methyl 4-(3-oxo-3-phenylpropyl)benzoate (2c) ........ 13

3-(4-chlorophenyl)-1-phenylpropan-1-one (2d) ........ 13

1-(4-fluorophenyl)-3-phenylpropan-1-one (2e) ........ 13

3-(4-chlorophenyl)-1-(4-fluorophenyl)propan-1-one (2f) ........ 13

4-phenylbutan-2-one (2g) ........ 14

Nonan-2-one (2h) ........ 14

4-(2,6,6-trimethylcyclohex-1-en-1-yl)butan-2-one (2i) ........ 14

1-phenylbutan-1-one (2j) ........ 14

4,4-dimethylcyclohexan-1-one (2k) ........ 15

Ethyl 2-(2-oxocyclohexyl)acetate (2l) ........ 15

4-phenylcyclohexan-1-one (2m) ........ 15

4-(tert-butyl)cyclohexan-1-one (2n) ........ 15

(2R,5R)-2-methyl-5-(prop-1-en-2-yl)cyclohexan-1-one (2o) ........ 16

(E)-1,3-diphenylprop-2-en-1-ol (3a) ........ 16

(E)-3-(4-methoxyphenyl)-1-phenylprop-2-en-1-ol (3b) ........ 17

Methyl (E)-4-(3-hydroxy-3-phenylprop-1-en-1-yl)benzoate (3c) ........ 17

(E)-3-(4-chlorophenyl)-1-phenylprop-2-en-1-ol (3d) ........ 18

(E)-1-(4-fluorophenyl)-3-phenylprop-2-en-1-ol (3e) ........................................................18
(E)-3-(4-chlorophenyl)-1-(4-fluorophenyl)prop-2-en-1-ol (3f) ...........................................19
(E)-4-phenylbut-3-en-2-ol (3g) ............................................................................19
(E)-non-3-en-2-ol (3h) ........................................................................................19
(E)-4-(2,6,6-trimethylcyclohex-1-en-1-yl)but-3-en-2-ol (3i)...............................................20
1-phenylbut-3-en-1-ol (3j) ....................................................................................20
4,4-dimethylcyclohex-2-en-1-ol (3k) ......................................................................21
Ethyl 2-((1R,2R)-2-hydroxycyclohex-3-en-1-yl)acetate (3l)..............................................21
(1S,4S)-1,2,3,4-tetrahydro-[1,1'-biphenyl]-4-ol (3m) ........................................................21
(1R,4R)-4-(tert-butyl)cyclohex-2-en-1-ol (3n) ..................................................................22
(1R,5R)-2-methyl-5-(prop-1-en-2-yl)cyclohex-2-en-1-ol (3o) ............................................22
(E)-3-phenylprop-2-en-1-ol (3p) ..........................................................................23
5.5 General procedure for the reductive aldol reaction (Procedure 5) ..............................24
2-(hydroxy(phenyl)methyl)-4,4-dimethylcyclohexan-1-one (4a) .......................................24
2-(hydroxy(phenyl)methyl)cyclohexan-1-one (4b) ...........................................................24
2-(hydroxy(phenyl)methyl)cyclopentan-1-one (4c) ...........................................................24
2-(hydroxy(phenyl)methyl)cycloheptan-1-one (4d) ...........................................................25
6-(hydroxy(phenyl)methyl)-6,7,8,9-tetrahydro-5H-benzo[7]annulen-5-one (4e) ................25
2-(hydroxy(phenyl)methyl)-2-methylcyclopentan-1-one (4f) .............................................25
3-(hydroxy(phenyl)methyl)nonan-2-one (4g) ...................................................................26
(2R,8R,9S,10R,13S,14S,17S)-2-(hydroxy(phenyl)methyl)-10,13,17-trimethyl-17-((trimethylsilyl)oxy)-1,2,6,7,8,9,10,11,12,13,14,15,16,17-tetradecahydro-3H-cyclopenta[a]phenanthren-3-one (4h) ..................................................................26
2-((4-ethoxyphenyl)(hydroxy)methyl)-4,4-dimethylcyclohexan-1-one (4i) ..........................26
2-(hydroxy(p-tolyl)methyl)-4,4-dimethylcyclohexan-1-one (4j) ..........................................26
2-((4-chlorophenyl)(hydroxy)methyl)-4,4-dimethylcyclohexan-1-one (4k) ..........................27
2-(-hydroxy(4-(trifluoromethyl)phenyl)methyl)-4,4-dimethylcyclohexan-1-one (4l) .............27
2-(furan-2-yl(hydroxy)methyl)-4,4-dimethylcyclohexan-1-one (4m) ..................................27
2-(hydroxy(thiophen-2-yl)methyl)-4,4-dimethylcyclohexan-1-one (4n)..............................27
2-(E-1-hydroxy-3-phenylallyl)-4,4-dimethylcyclohexan-1-one (4o)....................................28
2-(1-hydroxy-2-methylpropyl)-4,4-dimethylcyclohexan-1-one (4p)....................................28
**6. Mechanistic Experiments.......................................................................................29**
6.1 Temperature dependence of the reaction in the dark ..................................................29
6.2 1,2 hydroboraton of carvone and attempted isomerization .........................................29
**7. Computational details ..........................................................................................30**
7.1 General computational details.......................................................................................30
7.2 Benchmark of the computational method: ..................................................................31
7.3 Complementary pathways:...........................................................................................32
**8. NMR Spectra .........................................................................................................72**

1,3-diphenylpropan-1-one (2a)....72
3-(4-methoxyphenyl)-1-phenylpropan-1-one (2b)....73
methyl 4-(3-oxo-3-phenylpropyl)benzoate (2c)....74
3-(4-chlorophenyl)-1-phenylpropan-1-one (2d)....75
1-(4-fluorophenyl)-3-phenylpropan-1-one (2e)....76
3-(4-chlorophenyl)-1-(4-fluorophenyl)propan-1-one (2f)....77
4-phenylbutan-2-one (2g)....79
Nonan-2-one (2h)....80
4-(2,6,6-trimethylcyclohex-1-en-1-yl)butan-2-one (2i)....81
1-phenylbutan-1-one (2j)....82
4,4-dimethylcyclohexan-1-one (2k)....83
ethyl 2-(2-oxocyclohexyl)acetate (2l)....84
4-phenylcyclohexan-1-one (2m)....85
4-(tert-butyl)cyclohexan-1-one (2n)....86
(2R,5R)-2-methyl-5-(prop-1-en-2-yl)cyclohexan-1-one (2o)....87
(E)-1,3-diphenylprop-2-en-1-ol (3a)....88
(E)-3-(4-methoxyphenyl)-1-phenylprop-2-en-1-ol (3b)....89
methyl (E)-4-(3-hydroxy-3-phenylprop-1-en-1-yl)benzoate (3c)....90
(E)-3-(4-chlorophenyl)-1-phenylprop-2-en-1-ol (3d)....91
(E)-1-(4-fluorophenyl)-3-phenylprop-2-en-1-ol (3e)....92
(E)-3-(4-chlorophenyl)-1-(4-fluorophenyl)prop-2-en-1-ol (3f)....93
(E)-4-phenylbut-3-en-2-ol (3g)....95
(E)-non-3-en-2-ol (3h)....96
(E)-4-(2,6,6-trimethylcyclohex-1-en-1-yl)but-3-en-2-ol (3i)....97
1-phenylbut-3-en-1-ol (3j)....98
4,4-dimethylcyclohex-2-en-1-ol (3k)....99
ethyl 2-((1R,2R)-2-hydroxycyclohex-3-en-1-yl)acetate (3l)....99
(1S,4S)-1,2,3,4-tetrahydro-[1,1'-biphenyl]-4-ol (3m)....100
(1R,4R)-4-(tert-butyl)cyclohex-2-en-1-ol (3n)....101
(1R,5R)-2-methyl-5-(prop-1-en-2-yl)cyclohex-2-en-1-ol (3o)....103
(E)-3-phenylprop-2-en-1-ol (3p)....104
2-(hydroxy(phenyl)methyl)-4,4-dimethylcyclohexan-1-one (4a)....105
2-(hydroxy(phenyl)methyl)cyclohexan-1-one (4b)....106
2-(hydroxy(phenyl)methyl)cyclopentan-1-one (4c)....107
2-(hydroxy(phenyl)methyl)cycloheptan-1-one (4d)....108
6-(hydroxy(phenyl)methyl)-6,7,8,9-tetrahydro-5H-benzo[7]annulen-5-one (4e)....109
2-(hydroxy(phenyl)methyl)-2-methylcyclopentan-1-one (4f)....110
3-(hydroxy(phenyl)methyl)nonan-2-one (4g)....111

(2R,8R,9S,10R,13S,14S,17S)-2-(hydroxy(phenyl)methyl)-10,13,17-trimethyl-17-((trimethylsilyl)oxy)-1,2,6,7,8,9,10,11,12,13,14,15,16,17-tetradecahydro-3H-cyclopenta[a]phenanthren-3-one (4h) ....... 112

2-((4-ethoxyphenyl)(hydroxy)methyl)-4,4-dimethylcyclohexan-1-one (4i) ....... 113

2-(hydroxy(p-tolyl)methyl)-4,4-dimethylcyclohexan-1-one (4j) ....... 114

2-((4-chlorophenyl)(hydroxy)methyl)-4,4-dimethylcyclohexan-1-one (4k) ....... 115

2-(-hydroxy(4-(trifluoromethyl)phenyl)methyl)-4,4-dimethylcyclohexan-1-one (4l) ....... 116

2-(furan-2-yl(hydroxy)methyl)-4,4-dimethylcyclohexan-1-one (4m) ....... 117

2-(hydroxy(thiophen-2-yl)methyl)-4,4-dimethylcyclohexan-1-one (4n) ....... 118

2-(E-1-hydroxy-3-phenylallyl)-4,4-dimethylcyclohexan-1-one (4o) ....... 119

2-(1-hydroxy-2-methylpropyl)-4,4-dimethylcyclohexan-1-one (4p) ....... 120

**8. References ....... 121**

# 1. Materials and Methods

Unless otherwise stated, all reactions were performed utilizing standard Schlenk techniques. All reagents and starting materials were purchased at reagent grade and used as received. Anhydrous solvents were dried using an Innovative Technology PS-MD-5 solvent purification system. Thin layer chromatography (TLC) was performed on Merck Kieselgel 60 F254 aluminum plates with unmodified silica and visualized either under UV light or stained with potassium permanganate or vanillin. Column chromatography was performed with Merck silica gel 60 (35 – 70 mesh).

All $^{1}H$, $^{13}C$ and $^{19}F$ NMR spectra were recorded at ambient temperature on either Varian V-NMRS 600, Varian V-NMRS 400, Bruker AV-600, Bruker AV-400 or Varian Mercury 300 spectrometers. Chemical shifts (δ/ppm) were referenced to the residual solvent peak in $^{1}H$ (7.26 ppm for $CDCl_3$) and $^{13}C$ spectra (77.16 ppm for $CDCl_3$). Coupling constants (*J*) are given in Hz. Signals are described as br = broad, s = singlet, d = doublet, dd = doublet of doublets, t = triplet, q = quartet, p = quintet, h = sextet and m = multiplet.

High-resolution mass spectrometry (HRMS) was performed using a Thermo Scientific LTQ Orbitrap XL spectrometer. Infrared (IR) spectra were recorded on a Perkin Elmer Spektrum 100 FT-IR spectrometer Spectrum 100 spectrometer with an UATR Diamond/KRS-5 crystal with attenuated total reflectance (ATR) and signals reported as wavenumbers in reciprocal centimeters.

# 2. Synthesis and Characterization Data of $CoH[PPh(OEt)_2]_4$

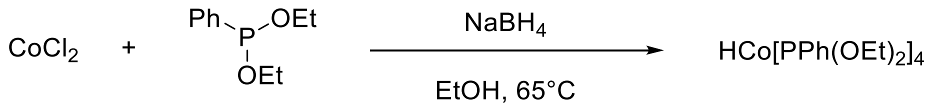


**Procedure**

Anhydrous $CoCl_2$ (650 mg, 5 mmol, 1 eq) was dissolved in dry ethanol (30 ml) inside an oven dried schlenk flask under an Argon atmosphere, forming a blue solution. This solution was then heated in an oil bath at 65°C. $PPh(OEt)_2$ (4,9 ml, 25 mmol, 5 eq) was added and the solution turned green. The $NaBH_4$ (430 mg, 11 mmol, 2.2 eq) was slowly added dropwise as suspension in dry ethanol (20 ml), during the addition the solution becomes bright yellow and $H_2$ is produced. Once the gas evolution has stopped, the solution was filtered under Argon into a clean Schlenk flask and around half of the solvent was removed under vacuum. The remaining solution was cooled at 0°C with an ice bath to induce the precipitation of the product. After 20 minutes the precipitate (2.25 g, 2.65 mmol, 53%), a bright yellow solid, was filtered, dried under high vacuum and weighed.

**$^{1}H$ NMR (400 MHz, $CD_2Cl_2$)** δ 7.44 (s, 8H), 7.17 (s, 12H), 3.62 (s, 8H), 3.32 (s, 8H), 1.04 (s, 24H), -14.71 (q, *J* = 22.7 Hz, 1H). **$^{13}C$ NMR (101 MHz, $CD_2Cl_2$)** δ 131.2 – 130.2 (m), 127.8, 127.1, 60.0, 16.5. **$^{31}P$ NMR** (162 MHz, $CD_2Cl_2$) δ = 174.32.

**IR (neat):** ν = 3056, 2971, 2927, 2887, 1480, 1436, 1384, 1099, 1024, 913, 726, 691 $cm^{-1}$

**HRMS (ESI):** *m/z* $[M+Na]^+$ calcd. for $[C_{40}H_{61}O_8P_4CoNa]^+$: 875.2547, found: 875.2544.

**Figure S1. UV-Vis absorption spectra of Cobalt catalyst $CoH[PPh(OEt)_2]_4$**

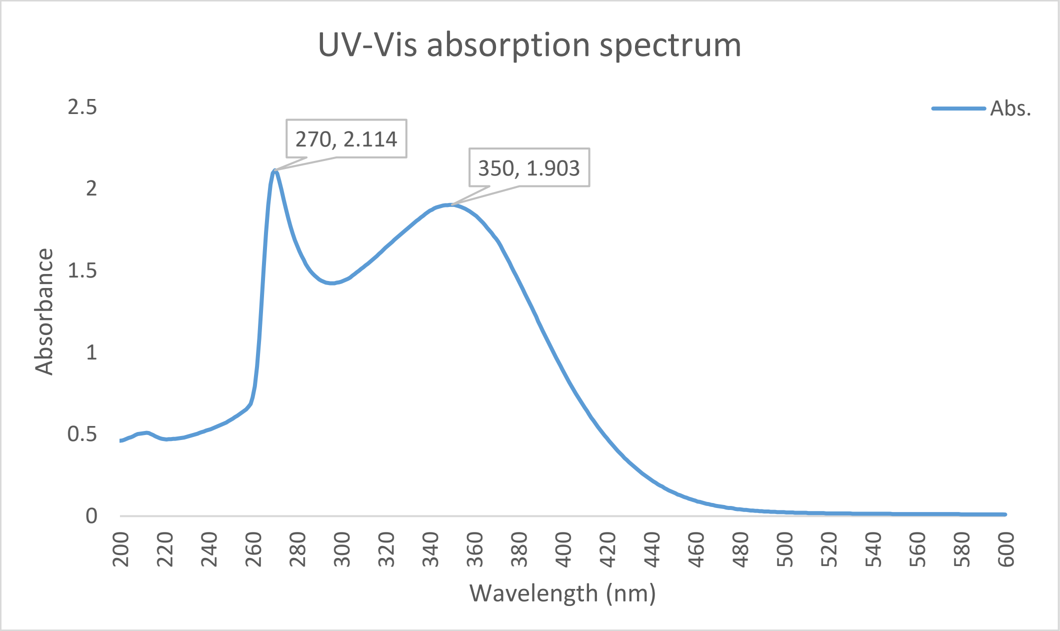

# 3. Selected optimization for the reduction of cyclic enones

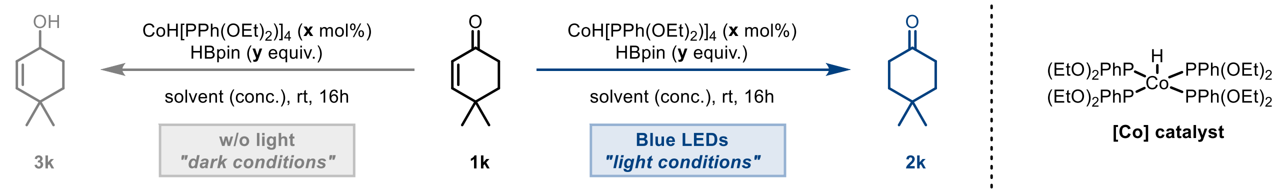


| | entry | [Co] **x** mol% | HBpin **y** equiv. | solvent (conc.) | Light source | Yield **3k** (%) | Yield **2k** (%) | conversion (%) |
|---|---|---|---|---|---|---|---|---|
| "dark conditions" | 1 | 5 | 1 | benzene (0.2 M) | off | 33 | 10 | 100 |
| | 2 | 2 | 2 | benzene (0.2 M) | off | 82 | 7 | 100 |
| | 3 | 2 | 2 | benzene (1 M) | off | 75 | 12 | 100 |
| | 4 | 5 | 2 | benzene (1 M) | off | 89 | 5 | 100 |
| | 5 | 5 | 2 | benzene (0.1 M) | off | 77 | 15 | 100 |
| | 6 | - | 2 | benzene (1 M) | off | 0 | 0 | 0 |
| | 7 | 5 | - | benzene (1 M) | off | 0 | 0 | 0 |
| "light conditions" | 8 | 2 | 2 | benzene (0.2 M) | on | 0 | 59 | 100 |
| | 9 | 5 | 2 | benzene (1 M) | on | 18 | 74 | 100 |
| | 10 | 5 | 2 | benzene (0.1 M) | on | 12 | 77 | 100 |
| | 11 | 5 | 2 | THF (0.2 M) | on | 8 | 87 | 100 |
| | 12 | 5 | 2 | THF (0.1 M) | on | 0 | 98 | 100 |
| | 13 | - | 2 | THF (0.1 M) | on | 0 | 0 | 0 |
| | 14 | 5 | - | THF (0.1 M) | on | 0 | 5 | 5 |

# 4. Preparation of starting materials

### 4.1 Procedure for the synthesis of 1-phenylbut-3-en-1-one 1j

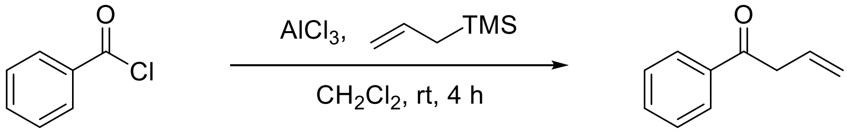


**C1** **1j**

In a Schlenk tube under Argon was introduced $AlCl_3$ (1.1 equiv., 2.2 mmol, 293 mg), $CH_2Cl_2$ (C = 0.25 M, 8 mL) and benzoyl chloride (1 equiv., 2 mmol, 0.23 mL) and the resulting mixture was stirred for 20 min at room temperature. Then, a solution of allyltrimethylsilane (1.2 equiv., 2.4 mmol, 0.38 mL) in $CH_2Cl_2$ (1 mL) was added dropwise and the reaction mixture was stirred at room temperature for 4 h. After this time, the reaction mixture was quenched by addition of cold distilled water. The aqueous layer was extracted with $Et_2O$ (3x) and the combined organic layers were dried ($MgSO_4$), filtered and concentrated under vacuum. The crude material was purified by column chromatography (0-5% $Et_2O$ in pentane) to afford title compound **1j** as a colorless liquid (176 mg, 1,20 mmol, 60%). $R_f$ = 0.74 (pentane/$Et_2O$ 7:3, vanillin).

**$^1$H NMR** (400 MHz, $CDCl_3$) δ 8.08 – 7.88 (m, 2H), 7.57 (t, *J* = 7.3 Hz, 1H), 7.47 (t, *J* = 7.6 Hz, 2H), 6.09 (ddt, *J* = 17.2, 10.5, 6.7 Hz, 1H), 5.38 – 5.14 (m, 2H), 3.77 (dt, *J* = 7.1, 1.5 Hz, 2H). **$^{13}$C NMR** (101 MHz, $CDCl_3$) δ 198.2, 136.7, 133.3, 131.2, 128.8 (x2), 128.4 (x2), 118.9, 43.6.

These data are in agreement with those reported previously in the literature.[1]

### 4.2 Procedure for the synthesis of ethyl 2-(2-oxocyclohex-3-en-1-yl)acetate (1l)

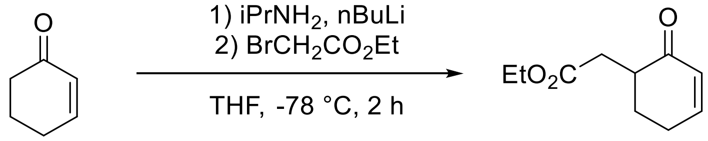


**B1** **1l**

In a Schlenk tube under Argon was introduced diisopropylamine (1.1 equiv., 5.5 mmol), and THF (C = 3 M, 1.7 mL). The mixture was cooled to 0 °C and *n*-butyllithium (1.1 equiv., 5.5 mmol, 2.2 mL, 2.5 M in hexanes) was added dropwise and the resulting mixture was stirred at 0 °C for 30 min. A solution of enone **B1** (1 equiv., 5 mmol, 0.48 mL) in THF (C = 3 M, 1.7 mL) was added dropwise at -78 °C and the reaction mixture was stirred for 15 min at the same temperature. Then, a solution of ethyl bromoacetate (1.2 equiv., 6 mmol, 0.67 mL) in THF (C = 3 M, 1.7 ml) was added dropwise and the reaction mixture was stirred at - 78 °C for 2 h. After this time, the reaction mixture was diluted with $Et_2O$ and quenched at 0 °C by the addition of a saturated aqueous solution of $NH_4Cl$. After warming up to room temperature, the aqueous layer was extracted with $Et_2O$ (3x). The combined organic layers were washed with a saturated aqueous solution of $NH_4Cl$, brine, dried ($MgSO_4$), filtered and concentrated under vacuum. The crude material was purified by column chromatography (0-30% $Et_2O$ in pentane) to afford title compound **1l** as a colorless liquid (411 mg, 2.26 mmol, 45%). $R_f$ = 0.37 (pentane/$Et_2O$ 1:1, vanillin).

**$^1$H NMR** (400 MHz, $CDCl_3$) δ 7.01 – 6.91 (m, 1H), 6.02 (ddd, *J* = 10.0, 2.8, 1.2 Hz, 1H), 4.15 (qd, *J* = 7.1, 2.1 Hz, 2H), 2.94 – 2.81 (m, 2H), 2.56 – 2.40 (m, 2H), 2.31 – 2.21 (m, 1H), 2.18 – 2.07 (m, 1H), 1.90 – 1.75 (m, 1H), 1.26 (t, *J* = 7.1 Hz, 3H). **$^{13}$C NMR** (101 MHz, $CDCl_3$) δ 199.6, 172.7, 150.0, 129.4, 60.7, 43.8, 34.7, 28.8, 26.1, 14.3.

These data are in agreement with those reported previously in the literature.[2]

### 4.3 General procedure for the synthesis of enones: Saegusa-Ito oxidation of cycloalkanones (Procedure A)

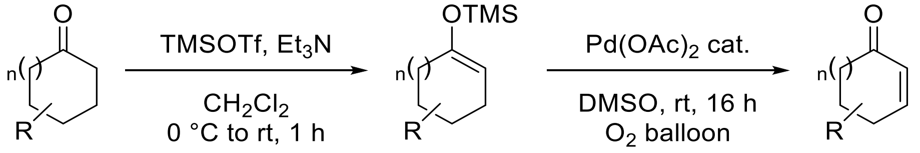


**Step 1 (Preparation of silyl enol ether):** In a Schlenk tube under Argon was introduced alkanone **A1** (1 equiv, 3 mmol) and dry $CH_2Cl_2$ (C = 0.21 M, 14 mL). The mixture was then cooled to 0 °C before the dropwise addition of TMSOTf (1.5 equiv, 4.5 mmol, 0.82 mL) and $Et_3N$ (3 equiv, 9 mmol, 1.25 mL). The reaction mixture was then allowed to reach room temperature and the stirring was continued for 1 h at the same temperature or until complete consumption of the starting material (monitored by TLC). The reaction mixture was quenched at 0 °C by the addition of a cold saturated aqueous solution of $NaHCO_3$. The aqueous layer was then extracted twice with $CH_2Cl_2$. The combined organic layers were dried ($MgSO_4$), filtered and concentrated under vacuum. The crude material **A2** was directly engaged in the next step without further purification.

**Step 2 (Saegusa-Ito oxidation):** In a Schlenk tube under air was introduced silyl enol ether **A2** (1 equiv, 3 mmol), DMSO (C = 0.1 M, 30 mL) and $Pd(OAc)_2$ (10 mol%, 0.3 mmol, 67 mg). Then, the Schlenk tube was purged with oxygen and a balloon of oxygen was placed on the top and the reaction mixture was stirred at room temperature overnight. After this time, the reaction mixture was quenched by the addition of distilled water. The aqueous layer was extracted with $Et_2O$ (3x) and the combined organic layers were washed with brine, dried ($MgSO_4$), filtered and concentrated under vacuum (note: some of the synthesized enones are volatile). The crude material was purified by column chromatography to afford title compounds **A3**.

**2,3-dihydro-[1,1'-biphenyl]-4(1H)-one (1m)**

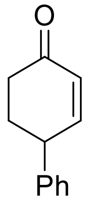


Prepared following general procedure A, **1m** was obtained after purification by column chromatography (0-20% $Et_2O$ in pentane) as a colorless liquid (491 mg, 2.85 mmol, 95%). $R_f$ = 0.31 (pentane/$Et_2O$ 3:2, vanillin). **$^1$H NMR** (600 MHz, $CDCl_3$) δ 7.36 (t, *J* = 7.6 Hz, 2H), 7.29 (t, *J* = 7.4 Hz, 1H), 7.22 (dd, *J* = 7.0, 1.7 Hz, 2H), 6.99 (ddd, *J* = 10.1, 2.9, 1.3 Hz, 1H), 6.17 (dd, *J* = 10.1, 2.6 Hz, 1H), 3.73 (ddt, *J* = 10.0, 5.2, 2.7 Hz, 1H), 2.56 (dt, *J* = 16.7, 5.0 Hz, 1H), 2.48 (ddd, *J* = 16.6, 11.9, 4.7 Hz, 1H), 2.37 (dqd, *J* = 15.2, 5.0, 1.4 Hz, 1H), 2.06 (dddd, *J* = 13.7, 11.8, 9.4, 4.5 Hz, 1H). **$^{13}$C NMR** (151 MHz, $CDCl_3$) δ 199.4, 153.0, 143.0, 130.2, 129.0 (x2), 127.7 (x2), 127.3, 42.8, 37.1, 32.6.

These data are in agreement with those reported previously in the literature.[3]

**4-(tert-butyl)cyclohex-2-en-1-one (1n)**

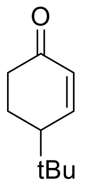


Prepared following general procedure A, **1n** was obtained after purification by column chromatography (0-10% $Et_2O$ in pentane) as a colorless liquid (420 mg, 2.76 mmol, 92%). $R_f$ = 0.41 (pentane/$Et_2O$ 7:3, vanillin). **$^1$H NMR** (600 MHz, $CDCl_3$) δ 7.01 (dt, *J* = 11.0, 2.1 Hz, 1H), 6.03 (ddd, *J* = 11.0, 2.8, 0.9 Hz, 1H), 2.51 (dt, *J* = 16.7, 3.7 Hz, 1H), 2.33 (ddd, *J* = 16.5, 14.1, 5.1 Hz, 1H), 2.23 – 2.16 (m, 1H), 2.13 – 2.06 (m, 1H), 1.73 (ddd, *J* = 24.1, 13.7, 3.8 Hz, 1H), 0.97 (s, 9H). **$^{13}$C NMR** (151 MHz, $CDCl_3$) δ 200.2, 153.1, 130.1, 47.0, 38.0, 33.0, 27.5 (x3), 24.5.

These data are in agreement with those reported previously in the literature.[4]

**8,9-dihydro-5H-benzo[7]annulen-5-one (S1)**

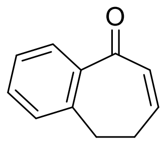


Prepared following general procedure A, **S1** was obtained after purification by column chromatography (0-10% $Et_2O$ in pentane) as a yellow liquid (313 mg, 1.98 mmol, 66%). $R_f$ = 0.44 (pentane/$Et_2O$ 3:2, vanillin). **$^1$H NMR** (400 MHz, $CDCl_3$) δ 7.75 (dd, *J* = 7.7, 1.5 Hz, 1H), 7.42 (td, *J* = 7.4, 1.5 Hz, 1H), 7.31 (td, *J* = 7.6, 1.3 Hz, 1H), 7.19 (d, *J* = 7.6 Hz, 1H), 6.75 (dt, *J* = 12.1, 4.9 Hz, 1H), 6.28 (dt, *J* = 12.1, 1.9 Hz, 1H), 3.18 – 3.00 (m, 2H), 2.60 (dd, *J* = 8.0, 4.1 Hz, 3H). **$^{13}$C NMR** (101 MHz, $CDCl_3$) δ 195.2, 151.2, 147.2, 139.9, 132.6, 132.3, 129.8, 129.0, 126.9, 34.6, 29.9.

These data are in agreement with those reported previously in the literature.[5]

**4.4 Procedure for the TMS protection of the steroid S2 to give (8R,9S,10R,13S,14S,17S)-10,13,17-trimethyl-17-((trimethylsilyl)oxy)-6,7,8,9,10,11,12,13,14,15,16,17-dodecahydro-3H-cyclopenta[a]phenanthren-3-one (S3)**

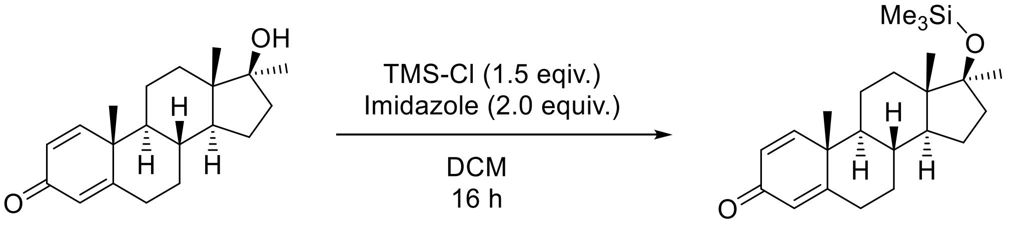


**S2** **S3**

The starting material **S2** (200 mg, 0.68 mmol) was dissolved in anhydrous DCM (10 ml). Imidazole (1.36 mmol, 92 mg) was then added in one portion, followed by TMSCl (111 mg, 1.0 mmol). The resulting solution was left at stirring for 16 hours; after this time, it was quenched with a saturated aqueous solution of $NaHCO_3$ (10 ml). The layers were separated and the organic layer was washed with water (2x10ml) and brine, dried over $MgSO_4$ and evaporated. The resulting white solid (245 mg, 0.66 mmol, 97%) was used without further purification.

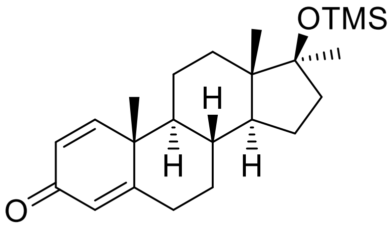


**$^1$H NMR** (600 MHz, Chloroform-*d*) δ 6.98 (d, *J* = 10.1 Hz, 1H), 6.14 (dd, *J* = 10.1, 1.9 Hz, 1H), 5.98 (t, *J* = 1.7 Hz, 1H), 2.38 (td, *J* = 13.4, 5.4 Hz, 1H), 2.27 (dt, *J* = 13.3, 3.5 Hz, 1H), 1.91 – 1.77 (m, 2H), 1.70 – 1.52 (m, 3H), 1.47 (s, 8H), 1.23 (tt, *J* = 12.3, 6.2 Hz, 1H), 1.16 (s, 3H), 1.07 (s, 3H), 1.00 (td, *J* = 11.4, 7.5 Hz, 1H), 0.92 (ddt, *J* = 20.3, 13.5, 6.3 Hz, 2H), 0.76 (s, 3H), 0.00 (s, 9H). **$^{13}$C NMR** (151 MHz, Chloroform-*d*) δ 184.0, 167.0, 153.7, 125.0, 121.4, 81.3, 50.2, 46.3, 44.2, 41.3, 36.4, 34.1, 31.0, 30.5, 28.8, 23.9, 21.3, 20.27, 16.4, 11.9, 0.00. **IR (neat):** ν = 2943, 2868, 1664, 1623, 1247, 1169, 1091, 1018, 878, 832 $cm^{-1}$. **HRMS (ESI):** *m/z* $[M+H]^+$ calcd. for $[C_{23}H_{37}OSi]^+$: 373.2557, found: 373.2562.

# 5. General procedures and characterization data of reduction products

## 5.1 General procedure 1

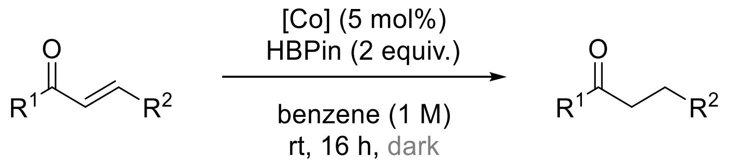


**F1** **F2**

**General procedure:** In an oven-dried vial were introduced the cobalt catalyst (5 mol%, 10 μmol, 8.6 mg) and a stirring bar. A cap with rubber septum was used to close the vial and the system was then purged with Argon. benzene (C = 1 M, 0.2 mL), chalcone **F1** (1 equiv, 0.2 mmol) and HBPin (2 equiv, 0.4 mmol, 58 μL) were then added successively and the vial was placed in the dark overnight. After this time, the solution was diluted with $Et_2O$ and washed two times with distilled water, the combined aqueous layers were extracted two times with $Et_2O$ and the combined organic layers were washed with brine, dried ($MgSO_4$), filtered and concentrated under vacuum. The crude material was purified by column chromatography to afford the corresponding ketone **F2**.

## 5.2 General procedure 2

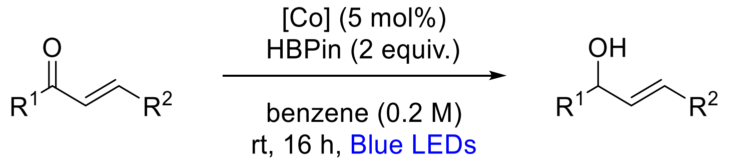


**G1** **G2**

**General procedure:** In an oven-dried vial were introduced the cobalt catalyst (5 mol%, 10 μmol, 8.6 mg) and a stirring bar. A cap with rubber septum was used to close the vial and the system was then purged with Argon. benzene (C = 0.2 M, 1 mL), chalcone **G1** (1 equiv, 0.2 mmol) and HBPin (2 equiv, 0.4 mmol, 58 μL) were then added successively and the vial was placed in the dark overnight. After this time, the solution was diluted with $Et_2O$ and washed two times with distilled water, the combined aqueous layers were extracted two times with $Et_2O$ and the combined organic layers were washed with brine, dried ($MgSO_4$), filtered and concentrated under vacuum. The crude material was purified by column chromatography to afford the corresponding allylic alcohol **G2**.

## 5.3 General procedure 3

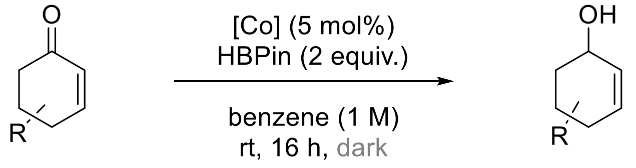


**D1** **D2**

**General procedure:** In an oven-dried vial were introduced the cobalt catalyst (5 mol%, 10 μmol, 8.6 mg) and a stirring bar. A cap with rubber septum was used to close the vial and the system was then purged with Argon. benzene (C = 1 M, 0.2 mL), enone **D1** (1 equiv, 0.2 mmol) and HBPin (2 equiv, 0.4 mmol, 58 μL) were then added successively and the vial was placed

in the dark overnight. After this time, the solution was diluted with $Et_2O$ and washed two times with distilled water, the combined aqueous layers were extracted two times with $Et_2O$ and the combined organic layers were washed with brine, dried ($MgSO_4$), filtered and concentrated under vacuum. The crude material was purified by column chromatography to afford the corresponding allylic alcohol **D2**.

### 5.4 General procedure 4

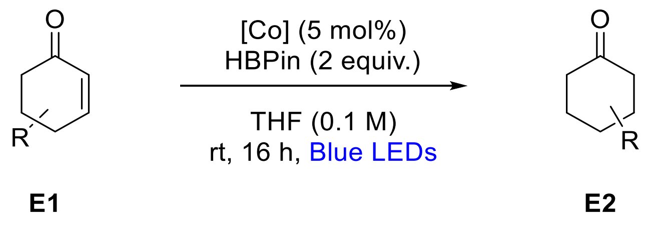

**General procedure:** In an oven-dried vial were introduced the cobalt catalyst (5 mol%, 10 µmol, 8.6 mg) and a stirring bar. A cap with rubber septum was used to close the vial and the system was then purged with Argon. THF (C = 0.1 M, 2 mL), enone **E1** (1 equiv., 0.2 mmol) and HBPin (2 equiv., 0.4 mmol, 58 µL) were then added successively and the vial was exposed to blue LEDs overnight. After this time, the solution was diluted with $Et_2O$ and washed two times with distilled water, the combined aqueous layers were extracted two times with $Et_2O$ and the combined organic layers were washed with brine, dried ($MgSO_4$), filtered and concentrated under vacuum. The crude material was purified by column chromatography to afford the corresponding ketone **E2**.

### 1,3-diphenylpropan-1-one (2a)

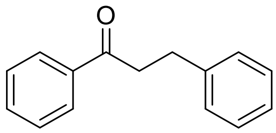

Prepared following general procedure 1, **2a** was obtained after purification by column chromatography (0-10% $Et_2O$ in pentane) as a white solid (32.8 mg, 156 µmol, 78%). $R_f$ = 0.58 (pentane/$Et_2O$ 6:4, vanillin). **$^1$H NMR** (600 MHz, $CDCl_3$) δ 8.01 – 7.91 (m, 2H), 7.66 – 7.51 (m, 1H), 7.45 (t, *J* = 7.8 Hz, 2H), 7.30 (t, *J* = 7.5 Hz, 2H), 7.27 – 7.24 (m, 2H), 7.23 – 7.16 (m, 1H), 3.30 (dd, *J* = 8.5, 7.0 Hz, 2H), 3.07 (t, *J* = 7.7 Hz, 2H). **$^{13}$C NMR** (151 MHz, $CDCl_3$) δ 199.3, 141.4, 137.0, 133.2, 128.7 (x2), 128.7 (x2), 128.6 (x2), 128.2 (x2), 126.3, 40.6, 30.3. **IR (neat):** ν = 2922, 1678, 1593, 1446, 1364, 1288, 1203, 971, 743, 692 $cm^{-1}$. **HRMS (ESI):** *m/z* $[M+Na]^+$ calcd. for $[C_{15}H_{14}ONa]^+$: 233.0937, found: 233.0937.

These data are in agreement with those reported previously in the literature.[6]

### 3-(4-methoxyphenyl)-1-phenylpropan-1-one (2b)

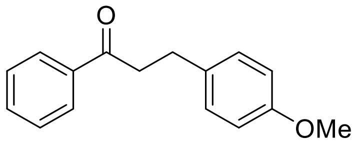

Prepared following general procedure 1, **2b** was obtained after purification by column chromatography (0-10% $Et_2O$ in pentane) as a white solid (37 mg, 154 µmol, 77%). $R_f$ = 0.49 (pentane/$Et_2O$ 6:4, vanillin).

**$^1$H NMR** (600 MHz, $CDCl_3$) δ 8.03 – 7.91 (m, 2H), 7.63 – 7.53 (m, 1H), 7.48 – 7.42 (m, 2H), 7.18 (d, *J* = 8.6 Hz, 2H), 6.85 (d, *J* = 8.6 Hz, 2H), 3.79 (s, 3H), 3.27 (dd, *J* = 8.4, 7.0 Hz, 2H), 3.02 (t, *J* = 7.7 Hz, 2H). **$^{13}$C NMR** (151 MHz, $CDCl_3$) δ 199.5, 158.1, 137.0, 133.4, 133.1, 129.5 (x2), 128.7 (x2), 128.2 (x2), 114.1 (x2), 55.4, 40.8, 29.4. **IR (neat):** ν = 2935, 1679, 1603, 1508, 1445, 1364, 1295, 1236, 1201, 1177, 1031, 975, 909, 822, 735, 690 $cm^{-1}$. **HRMS (ESI):** *m/z* $[M+Na]^+$ calcd. for $[C_{16}H_{16}O_2Na]^+$: 263.1042, found: 163.1039.

These data are in agreement with those reported previously in the literature.[7]

**Methyl 4-(3-oxo-3-phenylpropyl)benzoate (2c)**

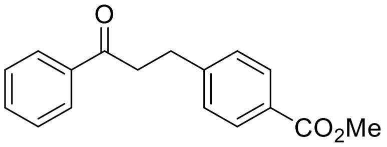

Prepared following general procedure 1, **2c** was obtained after purification by column chromatography (0-20% $Et_2O$ in pentane) as a white solid (45.1 mg, 168 µmol, 84%). $R_f$ = 0.41 (pentane/$Et_2O$ 6:4, vanillin). **$^1$H NMR** (600 MHz, $CDCl_3$) δ 7.99 – 7.90 (m, 4H), 7.59 – 7.53 (m, 1H), 7.49 – 7.42 (m, 2H), 7.32 (d, *J* = 8.2 Hz, 2H), 3.90 (s, 3H), 3.32 (dd, *J* = 8.2, 7.0 Hz, 2H), 3.13 (t, *J* = 7.6 Hz, 2H). **$^{13}$C NMR** (151 MHz, $CDCl_3$) δ 198.8, 167.1, 146.9, 136.8, 133.3, 130.0 (x2), 128.8 (x2), 128.6 (x2), 128.3, 128.1 (x2), 52.1, 39.9, 30.1. **IR (neat):** ν = 2951, 1707, 1681, 1603, 1436, 1362, 1276, 1198, 1179, 1102, 969, 766, 740, 686 $cm^{-1}$. **HRMS (ESI):** *m/z* $[M+Na]^+$ calcd. for $[C_{17}H_{16}O_3Na]^+$: 291.0992, found: 291.0989.

These data are in agreement with those reported previously in the literature.[8]

**3-(4-chlorophenyl)-1-phenylpropan-1-one (2d)**

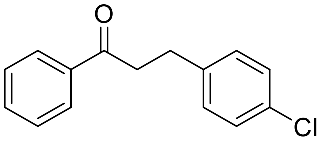

Prepared following general procedure 1, **2d** was obtained after purification by column chromatography (0-10% $Et_2O$ in pentane) as a white solid (34.7 mg, 142 µmol, 71%). $R_f$ = 0.57 (pentane/$Et_2O$ 6:4, vanillin). **$^1$H NMR** (600 MHz, $CDCl_3$) δ 8.08 – 7.90 (m, 2H), 7.61 – 7.53 (m, 1H), 7.50 – 7.43 (m, 2H), 7.26 (d, *J* = 8.4 Hz, 2H), 7.22 – 7.16 (m, 2H), 3.28 (dd, *J* = 8.1, 7.0 Hz, 2H), 3.05 (t, *J* = 7.5 Hz, 2H). **$^{13}$C NMR** (151 MHz, $CDCl_3$) δ 198.9, 139.9, 136.9, 133.3, 132.0, 129.9 (x2), 128.8 (x2), 128.7 (x2), 128.1 (x2), 40.2, 29.5. **IR (neat):** ν = 2924, 1665, 1592, 1489, 1446, 1366, 1266, 1207, 1091, 981, 824, 772, 741, 682 $cm^{-1}$. **HRMS (ESI):** *m/z* $[M+Na]^+$ calcd. for $[C_{15}H_{13}OClNa]^+$: 267.0547, found: 267.0547.

These data are in agreement with those reported previously in the literature.[9]

**1-(4-fluorophenyl)-3-phenylpropan-1-one (2e)**

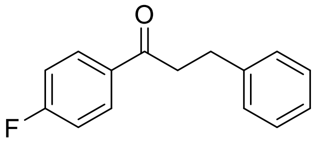

Prepared following general procedure 1, **2e** was obtained after purification by column chromatography (0-10% $Et_2O$ in pentane) as a white solid (31 mg, 136 µmol, 68%). $R_f$ = 0.59 (pentane/$Et_2O$ 6:4, vanillin). **$^1$H NMR** (600 MHz, $CDCl_3$) δ 8.09 – 7.95 (m, 2H), 7.37 – 7.28 (m, 2H), 7.27 – 7.24 (m, 2H), 7.24 – 7.19 (m, 1H), 7.16 – 7.07 (m, 2H), 3.28 (dd, *J* = 8.4, 6.9 Hz, 2H), 3.07 (t, *J* = 7.7 Hz, 2H). **$^{19}$F NMR** (565 MHz, $CDCl_3$) δ -105.32 (m). **$^{13}$C NMR** (151 MHz, $CDCl_3$) δ 197.7, 165.9 (d, $^1J_{C-F}$ = 254.9 Hz), 141.3, 133.4 (d, $^4J_{C-F}$ = 3.2 Hz), 130.8 (d, $^3J_{C-F}$ = 9.5 Hz, x2), 128.7 (x2), 128.5 (x2), 126.3, 115.8 (d, $^2J$ = 21.8 Hz, x2), 40.5, 30.2. **IR (neat):** ν = 2927, 1678, 1593, 1499, 1449, 1410, 1364, 1294, 1236, 1203, 1155, 976, 845, 781, 746, 699 $cm^{-1}$. **HRMS (ESI):** *m/z* $[M+Na]^+$ calcd. for $[C_{15}H_{13}OFNa]^+$: 251.0843, found: 251.0842.

These data are in agreement with those reported previously in the literature.[10]

**3-(4-chlorophenyl)-1-(4-fluorophenyl)propan-1-one (2f)**

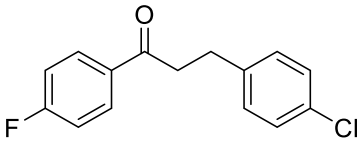

Prepared following general procedure 1, **2f** was obtained after purification by column chromatography (0-10% $Et_2O$ in pentane) as a white solid (38.9 mg, 148 µmol, 74%). $R_f$ = 0.56 (pentane/$Et_2O$ 6:4, vanillin). **$^1$H NMR** (600 MHz, $CDCl_3$) δ 8.44 – 7.78 (m, 2H), 7.29 – 7.23 (m, 2H), 7.20 – 7.16 (m, 2H), 7.14 – 7.09 (m, 2H), 3.25 (dd, *J* = 8.0, 7.0 Hz, 2H), 3.03 (t, *J* = 7.5 Hz, 2H). **$^{19}$F NMR** (565 MHz, $CDCl_3$) δ -105.07 (m). **$^{13}$C NMR** (151 MHz, $CDCl_3$) δ 197.3, 165.9 (d, $^1J_{C-F}$ = 254.7 Hz), 139.7, 133.3 (d, $^4J_{C-F}$ = 2.4 Hz), 132.1, 130.8 (d, $^3J_{C-F}$ = 8.8 Hz, x2), 129.9 (x2), 128.7 (x2), 115.9 (d, $^2J_{C-F}$ =

22.1 Hz, x2), 40.2, 29.5. **IR (neat):** ν = 2923, 1672, 1595, 1491, 1409, 1367, 1233, 1204, 1154, 1093, 983, 839, 812, 757 $cm^{-1}$. **HRMS (ESI):** *m/z* $[M+Na]^+$ calcd. for $[C_{15}H_{12}OClFNa]^+$: 285.0453, found: 285.0454.

These data are in agreement with those reported previously in the literature.[11]

**4-phenylbutan-2-one (2g)**

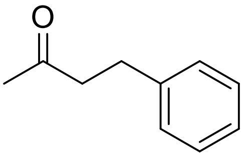


Prepared following general procedure 2, **2g** was obtained after purification by column chromatography (0-25% $Et_2O$ in pentane) as a colorless liquid (10.9 mg, 74 µmol, 37%). $R_f$ = 0.61 (pentane/$Et_2O$ 6:4, vanillin). **$^1$H NMR** (600 MHz, Chloroform-*d*) δ 7.35 – 7.24 (m, 1H), 7.25 – 7.15 (m, 1H), 2.90 (t, *J* = 7.7 Hz, 1H), 2.76 (t, *J* = 7.7 Hz, 1H), 2.14 (s, 1H).. **$^{13}$C NMR** (151 MHz, Chloroform-d) δ 207.9, 141.0, 128.5, 128.3, 126.1, 45.2, 30.1, 29.8. **HRMS (ESI):** *m/z* $[M+H]^+$ calcd. for $[C_{10}H_{12}ONa]^+$: 171.0780, found: 171.0779.

These data are in agreement with those reported previously in the literature.[12]

**Nonan-2-one (2h)**

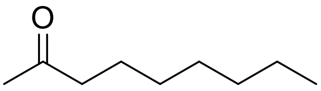


Prepared following general procedure 4, **2h** was obtained after purification by column chromatography (10% $Et_2O$ in pentane) as a colorless liquid (18 mg, 126 µmol, 63%). $R_f$ = 0.46 (pentane/$Et_2O$ 9:1, vanillin). **$^1$H NMR** (600 MHz, Chloroform-*d*) δ 2.40 (t, *J* = 7.5 Hz, 2H), 2.12 (s, 3H), 1.56 (t, *J* = 7.2 Hz, 2H), 1.27 (m, 8H), 0.87 (t, *J* = 6.8 Hz, 3H). **$^{13}$C NMR** (151 MHz, Chloroform-*d*) δ 209.4, 43.8, 31.7, 29.8, 29.1, 29.0, 23.9, 22.6, 14.0. **HRMS (ESI):** *m/z* $[M+H]^+$ calcd. for $[C_9H_{18}ONa]^+$: 165.1253, found: 165.1250. **IR (neat):** ν = 2926, 2857, 1715, 1459, 1414, 1359, 1163, 719 $cm^{-1}$

These data are in agreement with those reported previously in the literature.[13]

**4-(2,6,6-trimethylcyclohex-1-en-1-yl)butan-2-one (2i)**

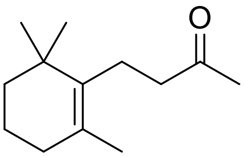


Prepared following general procedure 1, **2i** was obtained after purification by column chromatography (0-20% $Et_2O$ in pentane) as a colorless liquid (11 mg, 54 µmol, 27%). $R_f$ = 0.70 (pentane/$Et_2O$ 6:4, vanillin). **$^1$H NMR** (600 MHz, Chloroform-*d*) δ 2.53 – 2.47 (m, 2H), 2.29 – 2.23 (m, 2H), 2.14 (s, 3H), 1.90 (t, *J* = 6.3 Hz, 2H), 1.56 (q, *J* = 6.3, 5.8 Hz, 6H), 1.44 – 1.38 (m, 2H), 0.97 (s, 6H). **$^{13}$C NMR** (151 MHz, Chloroform-*d*) δ 209.0, 136.0, 127.8, 44.5, 39.7, 35.0, 32.7, 29.8, 28.4, 22.3, 19.7, 19.4. **HRMS (ESI):** *m/z* $[M+H]^+$ calcd. for $[C_{13}H_{22}ONa]^+$: 217.1563, found: 217.1561.

These data are in agreement with those reported previously in the literature.[14]

**1-phenylbutan-1-one (2j)**

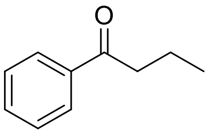


Prepared following general procedure 4, **3j** was obtained after purification by column chromatography (% $Et_2O$ in pentane) as a colorless liquid (19.3 mg, 130 µmol, 65%). **$^1$H NMR** (400 MHz, $CDCl_3$) δ 7.96 (dd, *J* = 7.3, 1.8 Hz, 2H), 7.63 – 7.51 (m, 1H), 7.46 (dd, *J* = 8.3, 6.8 Hz, 2H), 2.95 (t, *J* = 7.3 Hz, 2H), 1.77 (h, *J* = 7.4 Hz, 2H), 1.01 (t, *J* = 7.4 Hz, 3H). **$^{13}$C NMR** (101 MHz, $CDCl_3$) δ 200.6, 137.2, 133.0, 128.7 (x2), 128.2 (x2), 40.6, 17.9, 14.0. **IR (neat):** ν = 3062, 2963, 2876, 1685, 1597, 1451, 1408, 1368, 1311, 1275, 1214, 1181, 996, 897, 735, 692, 569 $cm^{-1}$. **HRMS (ESI):** *m/z* $[M+H]^+$ calcd. for $[C_{10}H_{13}O]^+$: 149.0961, found: 149.0960; *m/z* $[M+Na]^+$ calcd. for $[C_{10}H_{12}ONa]^+$: 171.0780, found: 171.0780.

These data are in agreement with those reported previously in the literature.[10]

**4,4-dimethylcyclohexan-1-one (2k)**

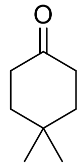

Prepared following general procedure 4, **2k** was obtained after purification by column chromatography (0-10% $Et_2O$ in pentane) as a colorless liquid (22.5 mg, 178 µmol, 89%). $R_f$ = 0.36 (pentane/$Et_2O$ 7:3, vanillin). **$^1$H NMR** (600 MHz, $CDCl_3$) δ 2.34 (t, $J$ = 6.7 Hz, 4H), 1.66 (td, $J$ = 6.8, 0.9 Hz, 4H), 1.09 (d, $J$ = 0.9 Hz, 6H). **$^{13}$C NMR** (151 MHz, $CDCl_3$) δ 212.7, 39.3 (x2), 38.1 (x2), 30.0, 27.6 (x2). **IR (neat):** ν = 2950, 2865, 2250, 1712, 1459, 1422, 1387, 1365, 1334, 1234, 1141, 1009, 916, 760, 734, 512 $cm^{-1}$. **HRMS (ESI):** *m/z* $[M+Na]^+$ calcd. for $[C_8H_{14}ONa]^+$: 149.0937, found: 149.0936.

These data are in agreement with those reported previously in the literature.[15]

**Ethyl 2-(2-oxocyclohexyl)acetate (2l)**

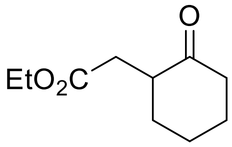


Prepared following general procedure 4, **2l** was obtained after purification by column chromatography (0-15% $Et_2O$ in pentane) as a colorless liquid (33.2 mg, 180 µmol, 90%). $R_f$ = 0.43 (pentane/$Et_2O$ 1:1, vanillin). **$^1$H NMR** (600 MHz, $CDCl_3$) δ 4.17 – 4.05 (m, 2H), 2.89 – 2.80 (m, 1H), 2.74 (dd, $J$ = 16.5, 7.1 Hz, 1H), 2.44 – 2.38 (m, 1H), 2.34 (tdd, $J$ = 13.5, 5.9, 1.2 Hz, 1H), 2.16 – 2.05 (m, 3H), 1.91 – 1.83 (m, 1H), 1.71 (qt, $J$ = 12.8, 3.4 Hz, 1H), 1.62 (qdd, $J$ = 13.1, 4.5, 3.3 Hz, 1H), 1.40 (qd, $J$ = 12.9, 3.7 Hz, 1H), 1.24 (t, $J$ = 7.1 Hz, 3H). **$^{13}$C NMR** (151 MHz, $CDCl_3$) δ 211.1, 172.7, 60.5, 47.2, 41.9, 34.6, 34.0, 27.9, 25.3, 14.3. **IR (neat):** ν = 2934, 2862, 2328, 2089, 1711, 1449, 1372, 1277, 1166, 1028 $cm^{-1}$. **HRMS (ESI):** *m/z* $[M+Na]^+$ calcd. for $[C_8H_{16}O_3Na]^+$: 207.0992, found: 207.0993.

These data are in agreement with those reported previously in the literature.[16]

**4-phenylcyclohexan-1-one (2m)**

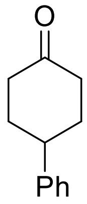


Prepared following general procedure 4, **2m** was obtained after purification by column chromatography (0-10% $Et_2O$ in pentane) as a white solid (32.4 mg, 186 µmol, 93%). $R_f$ = 0.33 (pentane/$Et_2O$ 6:4, vanillin). **$^1$H NMR** (600 MHz, $CDCl_3$) δ 7.35 – 7.31 (m, 2H), 7.26 – 7.22 (m, 3H), 3.03 (tt, $J$ = 12.1, 3.5 Hz, 1H), 2.55 – 2.44 (m, 4H), 2.29 – 2.19 (m, 2H), 2.03 – 1.90 (m, 2H). **$^{13}$C NMR** (151 MHz, $CDCl_3$) δ 211.2, 144.9, 128.7 (x2), 126.8 (x2), 126.7, 42.9, 41.5 (x2), 34.1 (x2). **IR (neat):** ν = 2939, 2868, 1701, 1493, 1450, 1327, 1164, 952, 757, 698 $cm^{-1}$. **HRMS (APCI):** *m/z* $[M+H]^+$ calcd. for $[C_{12}H_{15}O]^+$: 175.1123, found: 175.1116.

These data are in agreement with those reported previously in the literature.[17]

**4-(tert-butyl)cyclohexan-1-one (2n)**

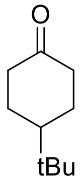


Prepared following general procedure 4, **2n** was obtained after purification by column chromatography (0-10% $Et_2O$ in pentane) as a white solid (25.9 mg, 168 µmol, 84%). $R_f$ = 0.44 (pentane/$Et_2O$ 7:3, vanillin). **$^1$H NMR** (600 MHz, $CDCl_3$) δ 2.47 – 2.35 (m, 2H), 2.34 – 2.25 (m, 2H), 2.12 – 2.02 (m, 2H), 1.54 – 1.38 (m, 3H), 0.91 (s, 9H). **$^{13}$C NMR** (151 MHz, $CDCl_3$) δ 212.7, 46.9, 41.4 (x2), 32.6, 27.7 (x2), 27.7 (x 3). **IR (neat):** ν = 2948, 2868, 1720, 1462, 1421, 1364, 1332, 1219, 1161, 944, 776 $cm^{-1}$. **HRMS (ESI):** *m/z* $[M+H]^+$ calcd. for $[C_{10}H_{19}O]^+$: 155.1430, found: 155.1432.

These data are in agreement with those reported previously in the literature.[18]

**(2R,5R)-2-methyl-5-(prop-1-en-2-yl)cyclohexan-1-one (2o)**

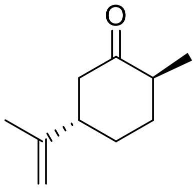

Prepared following general procedure 4, **2o** was obtained after purification by column chromatography (0-10% $Et_2O$ in pentane) as a colorless liquid (14.6 mg, 96 µmol, 48%). $R_f$ = 0.53 (pentane/$Et_2O$ 7:3, vanillin).

**2o** was obtained as an inseparable mixture of diastereomers: d.r. 81:19 (anti:syn).

**Major diastereomer (anti, fully described):** **$^1$H NMR** (600 MHz, $CDCl_3$) δ 4.75 (p, *J* = 1.5 Hz, 1H), 4.74 – 4.71 (m, 1H), 2.44 (dt, *J* = 11.9, 2.6 Hz, 1H), 2.40 – 2.25 (m, 3H), 2.12 (ddt, *J* = 13.1, 6.2, 3.4 Hz, 1H), 1.99 – 1.90 (m, 1H), 1.74 (s, 3H), 1.69 – 1.60 (m, 1H), 1.37 (qd, *J* = 13.1, 3.5 Hz, 1H), 1.03 (d, *J* = 6.5 Hz, 3H). **$^{13}$C NMR** (151 MHz, $CDCl_3$) δ 212.8, 147.8, 109.8, 47.2, 47.0, 44.9, 35.1, 30.9, 20.6, 14.5.

**Minor diastereomer (syn, only distinct signals):** **$^1$H NMR** (600 MHz, $CDCl_3$) δ 4.86 – 4.81 (m, 1H), 4.69 (s, 1H), 2.62 – 2.53 (m, 2H), 1.90 – 1.81 (m, 3H), 1.73 (d, *J* = 1.4 Hz, 3H), 1.09 (d, *J* = 6.9 Hz, 3H). **$^{13}$C NMR** (151 MHz, $CDCl_3$) δ 214.1, 147.0, 111.6, 44.7, 44.2, 44.1, 30.8, 26.5, 21.7, 15.8.

**IR (neat):** ν = 2965, 2930, 2862, 1710, 1645, 1449, 1373, 1217; 891, 729 $cm^{-1}$. **HRMS (ESI):** *m/z* $[M+H]^+$ calcd. for $[C_{10}H_{17}O]^+$: 153.1274, found: 153.1273.

These data are in agreement with those reported previously in the literature.[19,20]

**(E)-1,3-diphenylprop-2-en-1-ol (3a)**

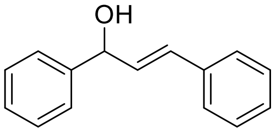

Prepared following general procedure 2, **3a** was obtained after purification by column chromatography (0-20% $Et_2O$ in pentane) as a colorless liquid (30.7 mg, 146 µmol, 73%). $R_f$ = 0.36 (pentane/$Et_2O$ 6:4, vanillin).

**3a** was obtained as an inseparable mixture of A:B (95:5).

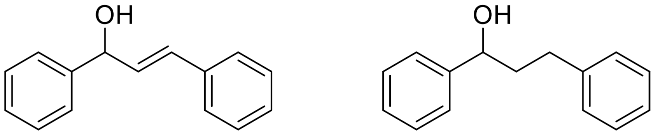

**A** **B**

**Major compound (A, fully described):** **$^1$H NMR** (600 MHz, $CDCl_3$) δ 7.42 – 7.33 (m, 2H), 7.32 – 7.27 (m, 4H), 7.25 – 7.19 (m, 3H), 7.18 – 7.13 (m, 1H), 6.60 (dd, *J* = 15.9, 1.3 Hz, 1H), 6.30 (dd, *J* = 15.8, 6.5 Hz, 1H), 5.30 (d, *J* = 6.5 Hz, 1H), 2.01 (s, 1H). **$^{13}$C NMR** (151 MHz, $CDCl_3$) δ 142.9, 136.7, 131.7, 130.7, 128.8 (x2), 128.7 (x2), 127.9, 127.9, 126.8 (x2), 126.5 (x2), 75.3.

**Minor compound (B, only distinct signals):** **$^1$H NMR** (600 MHz, $CDCl_3$) δ 4.60 (dd, *J* = 7.9, 5.3 Hz, 1H), 2.67 (ddd, *J* = 13.9, 9.8, 5.7 Hz, 1H), 2.58 (ddd, *J* = 14.0, 9.6, 6.5 Hz, 1H). **$^{13}$C NMR** (151 MHz, $CDCl_3$) δ 144.7, 128.6 (x2), 128.5 (x2), 127.6, 126.1, 74.0, 40.6, 32.2.

**IR (neat):** ν = 3345, 3027, 2860, 2325, 2091, 1598, 1493, 1449, 1299, 1189, 1067, 1010, 965, 745, 694 $cm^{-1}$. **HRMS (ESI):** *m/z* $[M+Na]^+$ calcd. for $[C_{15}H_{14}ONa]^+$: 233.0937, found: 233.0937.

These data are in agreement with those reported previously in the literature.[21,22]

**(E)-3-(4-methoxyphenyl)-1-phenylprop-2-en-1-ol (3b)**

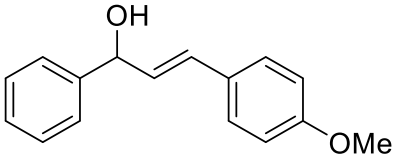


Prepared following general procedure 2, **3b** was obtained after purification by column chromatography (0-25% $Et_2O$ in pentane) as a colorless liquid (31.7 mg, 132 µmol, 66%). $R_f$ = 0.26 (pentane/$Et_2O$ 6:4, vanillin).

**3b** was obtained as an inseparable mixture of A:B (96:4).

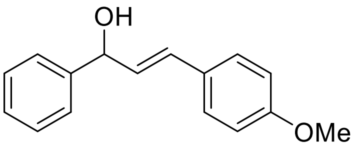


**A**

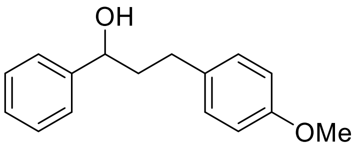


**B**

**Major compound (A, fully described):** $^{1}$**H NMR** (600 MHz, $CDCl_3$) δ 7.47 – 7.42 (m, 2H), 7.41 – 7.36 (m, 2H), 7.34 – 7.28 (m, 3H), 6.85 (d, *J* = 8.8 Hz, 2H), 6.63 (dd, *J* = 15.8, 1.2 Hz, 1H), 6.26 (dd, *J* = 15.8, 6.7 Hz, 1H), 5.39 – 5.34 (m, 1H), 3.80 (s, 3H), 2.07 (d, *J* = 3.0 Hz, 1H). $^{13}$**C NMR** (151 MHz, $CDCl_3$) δ 159.5, 143.1, 130.4, 129.5, 129.4, 128.7 (x2), 128.0 (x2), 127.8, 126.4 (x2), 114.1 (x2), 75.4, 55.4.

**Minor compound (B, only distinct signals):** $^{1}$**H NMR** (600 MHz, $CDCl_3$) δ 4.68 (t, *J* = 6.0 Hz, 1H), 3.79 (s, 3H), 2.70 (ddd, *J* = 15.1, 9.7, 5.7 Hz, 1H), 2.62 (ddd, *J* = 14.2, 9.5, 6.6 Hz, 1H). $^{13}$**C NMR** (151 MHz, $CDCl_3$) δ 158.0, 144.8, 133.9, 129.4 (x2), 128.6 (x2), 127.7, 126.1 (x2), 114.0 (x2), 74.0, 40.8, 31.2.

**IR (neat):** ν = 3391, 3029, 2837, 1606, 1509, 1453, 1297, 1246, 1175, 1029, 967, 828, 699 $cm^{-1}$. **HRMS (APCI):** *m/z* $[M+H]^+$ calcd. for $[C_{16}H_{17}O_2]^+$: 241.1229, found: 241.1205.

These data are in agreement with those reported previously in the literature.[22,23]

**Methyl (E)-4-(3-hydroxy-3-phenylprop-1-en-1-yl)benzoate (3c)**

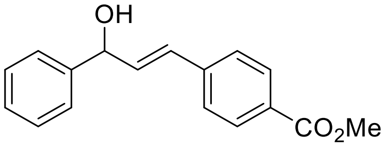


Prepared following general procedure 2, **3c** was obtained after purification by column chromatography (0-25% $Et_2O$ in pentane) as a colorless liquid (46.7 mg, 174 µmol, 87%). $R_f$ = 0.21 (pentane/$Et_2O$ 6:4, vanillin).

**3c** was obtained as an inseparable mixture of A:B (95:5).

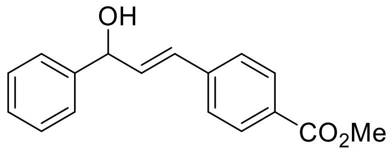


**A**

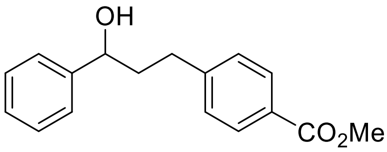


**B**

**Major compound (A, fully described):** $^{1}$**H NMR** (600 MHz, $CDCl_3$) δ 7.96 (d, *J* = 8.4 Hz, 2H), 7.45 – 7.41 (m, 4H), 7.40 – 7.36 (m, 2H), 7.33 – 7.29 (m, 1H), 6.72 (dd, *J* = 15.9, 1.3 Hz, 1H), 6.49 (dd, *J* = 15.8, 6.2 Hz, 1H), 5.40 (d, *J* = 6.1 Hz, 1H), 3.90 (s, 3H), 2.37 (br, 1H). $^{13}$**C NMR** (151 MHz, $CDCl_3$) δ 167.0, 142.6, 141.2, 134.3, 130.0 (x2), 129.4, 129.2, 128.8 (x2), 128.1, 126.6 (x2), 126.5 (x2), 75.0, 52.2.

**Minor compound (B, only distinct signals):** $^{1}$**H NMR** (600 MHz, $CDCl_3$) δ 4.67 (dd, *J* = 8.0, 5.3 Hz, 1H), 2.80 (ddd, *J* = 15.0, 9.9, 5.6 Hz, 1H), 2.72 (ddd, *J* = 14.4, 9.7, 6.5 Hz, 1H), 2.17 – 2.10 (m, 1H), 2.06 – 1.98 (m, 1H). $^{13}$**C NMR** (151 MHz, $CDCl_3$) δ 129.9 (x2), 128.7 (x2), 128.6 (x2), 126.0 (x2), 73.8, 40.2, 32.2.

**IR (neat):** ν = 3450, 3030, 2948, 1711, 1692, 1434, 1280, 1184, 1107, 1013, 965, 757, 695 $cm^{-1}$. **HRMS (ESI):** *m/z* $[M+Na]^+$ calcd. for $[C_{17}H_{16}O_3Na]^+$: 291.0992, found: 291.0987.

**(E)-3-(4-chlorophenyl)-1-phenylprop-2-en-1-ol (3d)**

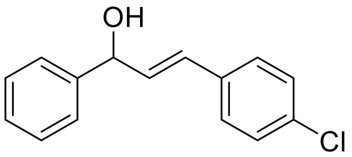

Prepared following general procedure 2, **3d** was obtained after purification by column chromatography (0-20% $Et_2O$ in pentane) as a colorless liquid (34.7 mg, 142 µmol, 71%). $R_f$ = 0.32 (pentane/$Et_2O$ 6:4, vanillin).

**3d** was obtained as an inseparable mixture of A:B (93:7).

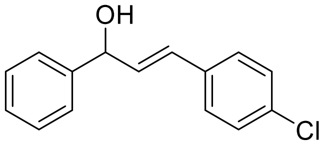

**A**

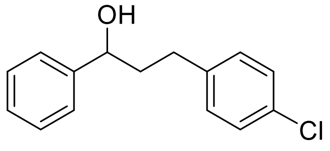

**B**

**Major compound (A, fully described):** $^{1}$**H NMR** (600 MHz, $CDCl_3$) δ 7.50 – 7.41 (m, 2H), 7.41 – 7.37 (m, 2H), 7.34 – 7.29 (m, 3H), 7.27 (d, *J* = 8.6 Hz, 2H), 6.64 (dd, *J* = 15.8, 1.3 Hz, 1H), 6.36 (dd, *J* = 15.8, 6.4 Hz, 1H), 5.38 (dd, *J* = 6.0, 2.1 Hz, 1H), 2.13 (d, *J* = 3.3 Hz, 1H). $^{13}$**C NMR** (151 MHz, $CDCl_3$) δ 142.7, 135.2, 133.5, 132.3, 129.3, 128.9 (x2), 128.8 (x2), 128.1, 127.9 (x2), 126.5 (x2), 75.1.

**Minor compound (B, only distinct signals):** $^{1}$**H NMR** (600 MHz, $CDCl_3$) δ 4.71 – 4.64 (m, 1H), 2.79 – 2.60 (m, 2H). $^{13}$**C NMR** (151 MHz, $CDCl_3$) δ 129.9 (x2), 126.0 (x2), 40.4, 31.5.

**IR (neat):** ν = 3345, 3029, 2862, 2327, 2086, 1594, 1489, 1451, 1403, 1300, 1185, 1088, 1010, 966, 821, 698 $cm^{-1}$. **HRMS (ESI):** *m/z* $[M+H]^+$ calcd. for $[C_{13}H_{14}OCl]^+$: 245.0733, found: 245.0733.

These data are in agreement with those reported previously in the literature.[24,25]

**(E)-1-(4-fluorophenyl)-3-phenylprop-2-en-1-ol (3e)**

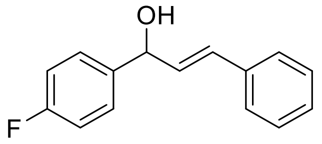

Prepared following general procedure 2, **3e** was obtained after purification by column chromatography (0-20% $Et_2O$ in pentane) as a colorless liquid (34.2 mg, 150 µmol, 75%). $R_f$ = 0.31 (pentane/$Et_2O$ 6:4, vanillin).

**3e** was obtained as an inseparable mixture of A:B (95:5).

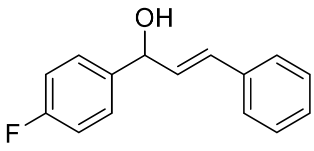

**A**

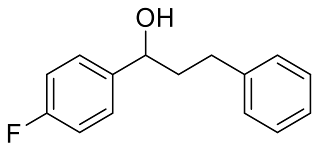

**B**

**Major compound (A, fully described):** $^{1}$**H NMR** (600 MHz, $CDCl_3$) δ 7.46 – 7.36 (m, 4H), 7.32 (dd, *J* = 8.4, 6.8 Hz, 2H), 7.28 – 7.24 (m, 1H), 7.09 – 7.03 (m, 2H), 6.68 (dd, *J* = 15.8, 1.2 Hz, 1H), 6.36 (dd, *J* = 15.8, 6.6 Hz, 1H), 5.37 (d, *J* = 6.5 Hz, 1H), 2.16 (s, 1H). $^{13}$**C NMR** (151 MHz, $CDCl_3$) δ 162.5 (d, $^{1}J_{C-F}$ = 245.9 Hz), 138.6 (d, $^{4}J_{C-F}$ = 3.3 Hz), 136.5, 131.4, 130.9, 128.8 (x2), 128.2 (d, $^{3}J_{C-F}$ = 8.5 Hz, x2), 128.1, 126.8 (x2), 115.6 (d, $^{2}J_{C-F}$ = 21.1 Hz, x2), 74.6.

**Minor compound (B, only distinct signals):** $^{1}$**H NMR** (600 MHz, $CDCl_3$) δ 4.67 (t, *J* = 6.7 Hz, 1H), 2.74 (ddd, *J* = 15.1, 9.7, 5.8 Hz, 1H), 2.66 (ddd, *J* = 13.9, 9.5, 6.5 Hz, 1H). $^{13}$**C NMR** (151 MHz, $CDCl_3$) δ 73.3, 40.7, 32.1.

**IR (neat):** ν = 3341, 3028, 2860, 2325, 2088, 1896, 1602, 1505, 1222, 1156, 1086, 1011, 966, 833, 741, 693 $cm^{-1}$. **HRMS (ESI):** *m/z* $[M+H]^+$ calcd. for $[C_{15}H_{14}FO]^+$: 229.1023, found: 229.1020.

These data are in agreement with those reported previously in the literature.[26,27]

**(E)-3-(4-chlorophenyl)-1-(4-fluorophenyl)prop-2-en-1-ol (3f)**

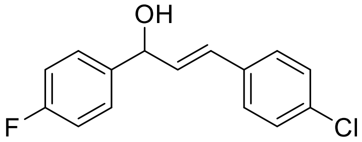


Prepared following general procedure 2, **3f** was obtained after purification by column chromatography (0-20% $Et_2O$ in pentane) as a colorless liquid (37.3 mg, 142 µmol, 71%). $R_f$ = 0.29 (pentane/$Et_2O$ 6:4, vanillin).

**3f** was obtained as an inseparable mixture of A:B (91:9).

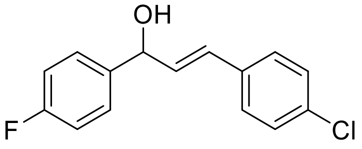


**A**

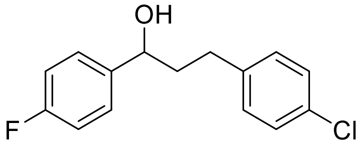


**B**

**Major compound (A, fully described):** $^{1}$**H NMR** (600 MHz, $CDCl_3$) δ 7.43 – 7.36 (m, 2H), 7.34 – 7.26 (m, 4H), 7.11 – 7.03 (m, 2H), 6.63 (dd, *J* = 15.8, 1.3 Hz, 1H), 6.32 (dd, *J* = 15.8, 6.4 Hz, 1H), 5.37 (dd, *J* = 6.5, 3.1 Hz, 1H), 2.07 (d, *J* = 3.5 Hz, 1H). $^{13}$**C NMR** (151 MHz, $CDCl_3$) δ 162.5 (d, $^{1}J_{C-F}$ = 246.2 Hz), 138.5 (d, $^{4}J_{C-F}$ = 2.7 Hz), 135.0, 133.7, 132.1, 129.6, 128.9 (x2), 128.2 (d, $^{3}J_{C-F}$ = 8.2 Hz, x2), 128.0 (x2), 115.7 (d, $^{2}J_{C-F}$ = 21.3 Hz, x2), 74.5.

**Minor compound (B, only distinct signals):** $^{1}$**H NMR** (600 MHz, $CDCl_3$) δ 4.73 – 4.57 (m, 1H), 2.82 – 2.57 (m, 2H).

**HRMS (ESI):** *m/z* $[M+H]^+$ calcd. for $[C_{15}H_{13}ClFO]^+$: 263.0633, found: 263.0624. **IR (neat):** ν = 3340, 2864, 2325, 1896, 1601, 1504, 1406, 1223, 1156, 1085, 1011, 967, 833, 730 $cm^{-1}$.

**(E)-4-phenylbut-3-en-2-ol (3g)**

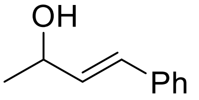


Prepared following general procedure 1, **3g** was obtained after purification by column chromatography (0-25% $Et_2O$ in pentane) as a white solid (19.9 mg, 134 µmol, 67%). $R_f$ = 0.34 (pentane/$Et_2O$ 6:4, vanillin). $^{1}$**H NMR** (600 MHz, Chloroform-*d*) δ 7.38 – 7.35 (m, 2H), 7.30 (dd, *J* = 8.4, 6.8 Hz, 2H), 7.25 – 7.21 (m, 1H), 6.67 – 6.50 (m, 1H), 6.25 (dd, *J* = 15.9, 6.4 Hz, 1H), 4.48 (td, *J* = 6.4, 1.3 Hz, 1H), 1.36 (d, *J* = 6.4 Hz, 3H).$^{13}$**C NMR** (151 MHz, $CDCl_3$) δ 136.7, 133.6, 129.4, 128.6, 127.7, 126.5, 69.0, 23.4. **HRMS (ESI):** *m/z* $[M+Na]^+$ calcd. for $[C_{10}H_{12}ONa]^+$: 171.0780, found: 171.0779.**IR (neat):** ν = 3343, 3027, 2972, 1493, 1448, 1368, 1293, 1139, 1058, 965, 943, 746, 691 $cm^{-1}$.

These data are in agreement with those reported previously in the literature.[28]

**(E)-non-3-en-2-ol (3h)**

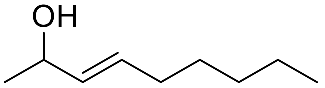


Prepared following general procedure 1, **3h** was obtained after purification by column chromatography (10% $Et_2O$ in pentane) as a colorless oil (14 mg, 98 µmol, 49%). $R_f$ = 0.12 (pentane/$Et_2O$ 9:1, vanillin). $^{1}$**H NMR** (600 MHz, Chloroform-*d*) δ 5.63 (dd, *J* = 15.3, 6.8 Hz, 1H), 5.50 (ddt, *J* = 15.4, 6.7, 1.4 Hz, 1H), 4.25 (p, *J* = 6.4 Hz, 1H), 2.00 (q, *J* = 7.2 Hz, 2H), 1.36 (p, *J* = 7.4 Hz,

2H), 1.32 – 1.24 (m, 7H), 1.22 – 1.17 (m, 1H), 0.88 (t, *J* = 6.9 Hz, 3H). **$^{13}$C NMR** (151 MHz, Chloroform-*d*) δ 134.1, 131.2, 69.0, 32.1, 31.4, 28.8, 23.4, 22.5, 14.0. **HRMS (ESI):** *m/z* $[M+H]^+$ calcd. for $[C_9H_{18}ONa]^+$: 165.1253, found: 165.1247. **IR (neat):** ν = 3339, 2959, 2857, 2325, 2109, 1884, 1456, 1370,1370, 1146, 1123, 1059, 967, 935 cm$^{-1}$

These data are in agreement with those reported previously in the literature.[29]

**(E)-4-(2,6,6-trimethylcyclohex-1-en-1-yl)but-3-en-2-ol (3i)**

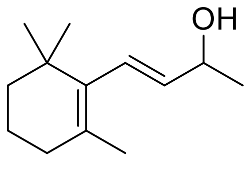


Prepared following general procedure 4, **3i** was obtained after purification by column chromatography (0-20% $Et_2O$ in pentane) as a colorless oil (33 mg, 170 µmol, 85%). $R_f$ = 0.44 (pentane/$Et_2O$ 6:4, vanillin). **$^1$H NMR** (600 MHz, Chloroform-*d*) δ 6.11 – 6.00 (m, 1H), 5.49 (dd, *J* = 15.9, 6.7 Hz, 1H), 4.36 (t, *J* = 6.4 Hz, 1H), 2.01 – 1.92 (m, 2H), 1.68 – 1.63 (m, 3H), 1.64 – 1.57 (m, 2H), 1.48 – 1.42 (m, 2H), 1.31 (d, *J* = 6.3 Hz, 3H), 0.98 (d, *J* = 2.0 Hz, 6H). **$^{13}$C NMR** (151 MHz, Chloroform-*d*) δ 137.63, 136.64, 128.80, 127.52, 69.51, 39.42, 33.92, 32.70, 28.70, 28.69, 23.58, 21.35, 19.24. . **IR (neat):** ν = 3331, 2962, 2925, 2865, 1453, 1364, 1141, 1058, 970, 941 cm$^{-1}$.**HRMS (APCI):** *m/z* $[M+H]^+$ calcd. for $[C_{13}H_{23}O]^+$: 195.1743, found: 195.1756.

These data are in agreement with those reported previously in the literature.[30]

**1-phenylbut-3-en-1-ol (3j)**

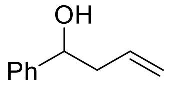


Prepared following general procedure 2, **3j** was obtained after purification by column chromatography (0-20% $Et_2O$ in pentane) as a colorless liquid (21 mg, 142 µmol, 71%). $R_f$ = 0.45 (pentane/$Et_2O$ 7:3, vanillin).

**3j** was obtained as an inseparable mixture of A:B:C (82:10:8).

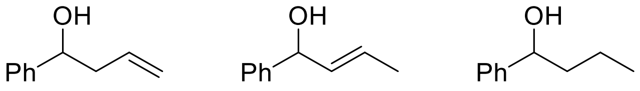


**A** **B** **C**

**Major compound (A, fully described): $^1$H NMR** (600 MHz, $CDCl_3$) δ 7.40 – 7.33 (m, 4H), 7.32 – 7.27 (m, 1H), 5.82 (ddt, *J* = 17.3, 10.1, 7.1 Hz, 1H), 5.23 – 5.11 (m, 2H), 4.74 (dd, *J* = 7.8, 5.2 Hz, 1H), 2.59 – 2.47 (m, 2H), 2.07 (s, 1H). **$^{13}$C NMR** (151 MHz, $CDCl_3$) δ 144.0, 134.6, 128.6 (x2), 127.7, 126.0 (x2), 118.5, 73.4, 44.0.

**Minor compound (B, only distinct signals): $^1$H NMR** (600 MHz, $CDCl_3$) δ 5.77 – 5.63 (m, 1H), 1.73 (d, *J* = 6.4 Hz, 3H). **$^{13}$C NMR** (151 MHz, $CDCl_3$) δ 143.5, 133.8, 128.6, 126.3, 75.3, 17.8.

**Minor compound (C, only distinct signals): $^1$H NMR** (600 MHz, $CDCl_3$) δ 0.94 (t, *J* = 7.4 Hz, 3H). **$^{13}$C NMR** (151 MHz, $CDCl_3$) δ 145.1, 127.6, 126.0, 74.6, 41.4, 19.2, 14.1.

**IR (neat):** ν = 3369, 2927, 2333, 2091, 1641, 1492, 1450, 1311, 1199, 1035, 994, 914, 870, 756, 698 cm$^{-1}$. **HRMS (ESI):** *m/z* $[M+Na]^+$ calcd. for $[C_{10}H_{12}ONa]^+$: 171.0780, found: 171.0782; *m/z* $[M+Na]^+$ calcd. for $[C_{10}H_{14}ONa]^+$: 173.0937, found: 173.0937.

These data are in agreement with those reported previously in the literature.[31–33]

**4,4-dimethylcyclohex-2-en-1-ol (3k)**

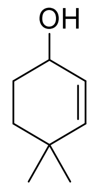


Prepared following general procedure 3, **3k** was obtained after purification by column chromatography (0-30% $Et_2O$ in pentane) as a colorless liquid (16.9 mg, 139 µmol, 70%). $R_f$ = 0.25 (pentane/$Et_2O$ 7:3, vanillin). **$^1$H NMR** (600 MHz, $CDCl_3$) δ 5.58 (dd, *J* = 10.0, 3.1 Hz, 1H), 5.52 (d, *J* = 10.0 Hz, 1H), 4.17 – 4.11 (m, 1H), 1.90 (dddd, *J* = 13.4, 8.4, 5.2, 3.1 Hz, 1H), 1.62 (dddd, *J* = 13.0, 9.6, 6.4, 3.1 Hz, 1H), 1.56 (ddd, *J* = 12.2, 8.6, 3.2 Hz, 1H), 1.52 (s, 1H), 1.42 (ddd, *J* = 13.2, 9.7, 3.2 Hz, 1H), 1.01 (s, 3H), 0.96 (s, 3H). **$^{13}$C NMR** (151 MHz, $CDCl_3$) δ 140.8, 127.5, 66.0, 33.7, 32.0, 29.4, 29.3, 29.2. **IR (neat):** ν = 3337, 2952, 2864, 1649, 1458, 1367, 1287, 1051, 1004, 835, 742 $cm^{-1}$. **HRMS (ESI):** *m/z* $[M+Na]^+$ calcd. for $[C_8H_{14}ONa]^+$: 149.0937, found: 149.0937.

These data are in agreement with those reported previously in the literature.[34]

**Ethyl 2-((1R,2R)-2-hydroxycyclohex-3-en-1-yl)acetate (3l)**

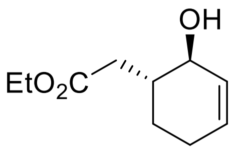


Prepared following general procedure 3, **3l** was obtained after purification by column chromatography (0-30% $Et_2O$ in pentane) as a colorless liquid (25.8 mg, 140 µmol, 70%). $R_f$ = 0.25 (pentane/$Et_2O$ 1:1, vanillin).

**3l** was obtained as an inseparable mixture of diastereomers: d.r. 73:27 (anti:syn).

**Major diastereomer (anti, fully described): $^1$H NMR** (600 MHz, $CDCl_3$) δ 5.80 – 5.75 (m, 1H), 5.65 (dq, *J* = 9.9, 2.2 Hz, 1H), 4.15 (q, *J* = 7.1 Hz, 2H), 3.92 (s, 1H), 2.62 (dd, *J* = 15.4, 6.2 Hz, 1H), 2.26 (dd, *J* = 15.4, 7.2 Hz, 1H), 2.17 – 1.91 (m, 4H), 1.83 – 1.72 (m, 1H), 1.46 – 1.35 (m, 1H), 1.26 (t, *J* = 7.2 Hz, 3H). **$^{13}$C NMR** (151 MHz, $CDCl_3$) δ 173.8, 130.2, 129.4, 71.6, 60.7, 39.5, 38.6, 26.7, 24.7, 14.4.

**Minor diastereomer (syn, only distinct signals): $^1$H NMR** (600 MHz, $CDCl_3$) δ 5.88 (ddd, *J* = 10.1, 4.6, 2.6 Hz, 1H), 5.82 (ddt, *J* = 9.8, 4.4, 2.0 Hz, 1H), 4.12 – 4.09 (m, 1H), 2.56 (dd, *J* = 15.2, 8.2 Hz, 1H), 2.31 (dd, *J* = 15.2, 6.6 Hz, 1H), 1.58 – 1.48 (m, 2H). **$^{13}$C NMR** (151 MHz, $CDCl_3$) δ 173.7, 131.2, 128.4, 65.7, 60.6, 36.5, 36.4, 25.2, 23.3, 14.4.

**IR (neat):** ν = 3421, 2979, 2921, 2327, 2089, 1726, 1446, 1374, 1269, 1208, 1174, 1054, 1026, 862, 678 $cm^{-1}$. **HRMS (ESI):** *m/z* $[M+Na]^+$ calcd. for $[C_{10}H_{16}O_3Na]^+$: 207.0992, found: 207.0993.

**(1S,4S)-1,2,3,4-tetrahydro-[1,1'-biphenyl]-4-ol (3m)**

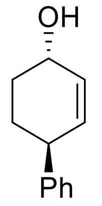


Prepared following general procedure 3, **3m** was obtained after purification by column chromatography (0-30% $Et_2O$ in pentane) as a colorless liquid (26.5 mg, 152 µmol, 76%). $R_f$ = 0.11 (pentane/$Et_2O$ 7:3, vanillin).

**3m** was obtained as an inseparable mixture of diastereomers: d.r. 66:34 (anti:syn).

**Major diastereomer (anti, fully described): $^1$H NMR** (600 MHz, $CDCl_3$) δ 7.37 – 7.28 (m, 2H), 7.25 – 7.16 (m, 3H), 5.95 – 5.88 (m, 1H), 5.84 (ddt, *J* = 10.1, 2.8, 1.3 Hz, 1H), 4.35 (ddq, *J* = 7.2, 4.7, 2.4, 1.9 Hz, 1H), 3.43 (ddq, *J* = 8.5, 5.7, 2.7 Hz, 1H), 2.16 – 2.09 (m, 1H), 1.87 – 1.80 (m, 1H), 1.68 – 1.62 (m, 1H), 1.68 – 1.54 (m, 2H). **$^{13}$C NMR** (151 MHz, $CDCl_3$) δ 145.4, 132.9, 131.7, 128.6 (x2), 127.7 (x2), 126.4, 66.7, 42.1, 32.0, 30.6.

**Minor diastereomer (syn, only distinct signals): $^1$H NMR** (600 MHz, $CDCl_3$) δ 6.00 (ddd, *J* = 10.0, 4.0, 2.3 Hz, 1H), 4.26 (q, *J* = 4.3 Hz, 1H), 3.35 (ddq, *J* = 8.0, 4.8, 2.3 Hz, 1H), 1.99 – 1.91 (m, 1H), 1.79 – 1.70 (m, 1H), 1.33 – 1.22 (m, 1H). **$^{13}$C NMR** (151 MHz, $CDCl_3$) δ 145.3, 134.0, 130.2, 128.6, 127.8, 64.5, 30.2, 27.9.

**IR (neat):** ν = 3331, 2932, 2859, 2328, 2088, 1874, 1806, 1601, 1491, 1448, 1386, 1282, 1051, 944, 854, 756, 699 $cm^{-1}$. **HRMS (ESI):** *m/z* $[M+H]^+$ calcd. for $[C_{12}H_{15}O]^+$: 175.1117, found: 175.1114.

These data are in agreement with those reported previously in the literature.[35,36]

**(1R,4R)-4-(tert-butyl)cyclohex-2-en-1-ol (3n)**

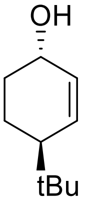


Prepared following general procedure 3, **3n** was obtained after purification by column chromatography (0-30% $Et_2O$ in pentane) as a colorless liquid (22.8 mg, 148 µmol, 74%). $R_f$ = 0.19 (pentane/$Et_2O$ 7:3, vanillin).

**3n** was obtained as an inseparable mixture of diastereomers: d.r. 82:18 (anti:syn).

**Major diastereomer (anti, fully described):** $^1$**H NMR** (600 MHz, $CDCl_3$) δ 5.77 (dq, *J* = 10.4, 1.9 Hz, 1H), 5.71 (ddd, *J* = 10.4, 4.5, 1.7 Hz, 1H), 4.20 (dddt, *J* = 9.7, 5.5, 3.8, 1.8 Hz, 1H), 2.17 – 2.11 (m, 1H), 1.94 – 1.86 (m, 1H), 1.84 – 1.75 (m, 1H), 1.47 (br, 1H), 1.38 (dddd, *J* = 14.2, 11.9, 9.7, 2.5 Hz, 1H), 1.29 (tdd, *J* = 13.4, 10.5, 2.4 Hz, 1H), 0.86 (s, 9H). $^{13}$**C NMR** (151 MHz, $CDCl_3$) δ 132.2, 131.4, 67.8, 46.5, 33.4, 33.0, 27.3 (x3), 22.9.

**Minor diastereomer (syn, only distinct signals):** $^1$**H NMR** (600 MHz, $CDCl_3$) δ 5.92 (dt, *J* = 10.4, 1.8 Hz, 1H), 5.87 (dddd, *J* = 10.3, 4.3, 2.5, 1.5 Hz, 1H), 4.11 – 4.08 (m, 1H), 1.70 – 1.58 (m, 3H), 0.90 (s, 9H). $^{13}$**C NMR** (151 MHz, $CDCl_3$) δ 134.2, 129.1, 63.5, 46.1, 32.7, 31.3, 27.4 (x3), 18.5.

**IR (neat):** ν = 3326, 2949, 2866, 2323, 2087, 1468, 1365, 1052, 875, 730 $cm^{-1}$. **HRMS (ESI):** *m/z* $[M+H]^+$ calcd. for $[C_{10}H_{19}O]^+$: 155.1430, found: 155.1429.

These data are in agreement with those reported previously in the literature.[37]

**(1R,5R)-2-methyl-5-(prop-1-en-2-yl)cyclohex-2-en-1-ol (3o)**

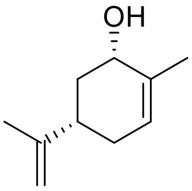


Prepared following general procedure 3, **3o** was obtained after purification by column chromatography (0-20% $Et_2O$ in pentane) as a colorless liquid (19.8 mg, 130 µmol, 65%). $R_f$ = 0.27 (pentane/$Et_2O$ 7:3, vanillin).

**3o** was obtained as an inseparable mixture of diastereomers: d.r. 87:13 (syn:anti).

**Major diastereomer (syn, fully described):** $^1$**H NMR** (600 MHz, $CDCl_3$) δ 5.50 (dq, *J* = 5.4, 1.7 Hz, 1H), 4.73 (s, 2H), 4.34 – 4.13 (m, 1H), 2.40 – 2.22 (m, 1H), 2.20 – 2.13 (m, 1H), 2.11 – 2.04 (m, 1H), 2.01 – 1.91 (m, 1H), 1.76 (d, *J* = 1.4 Hz, 3H), 1.74 (s, 3H), 1.51 (td, *J* = 12.2, 9.5 Hz, 2H). $^{13}$**C NMR** (151 MHz, $CDCl_3$) δ 149.1, 136.3, 124.0, 109.3, 71.1, 40.6, 38.2, 31.2, 20.8, 19.1.

**Minor diastereomer (anti, only distinct signals):** $^1$**H NMR** (600 MHz, $CDCl_3$) δ 5.62 – 5.56 (m, 1H), 4.02 (s, 1H), 2.39 – 2.32 (m, 1H), 1.89 – 1.84 (m, 1H), 1.82 – 1.79 (m, 2H), 1.66 – 1.59 (m, 1H). $^{13}$**C NMR** (151 MHz, $CDCl_3$) δ 149.3, 134.5, 125.5, 109.2, 68.7, 36.9, 35.4, 31.1, 21.1, 21.0.

**IR (neat):** ν = 3325, 2917, 2855, 2325, 2079, 1644, 1442, 1374, 1033, 888, 808 $cm^{-1}$. **HRMS (ESI):** *m/z* $[M+H]^+$ calcd. for $[C_{10}H_{17}O]^+$: 153.1274, found: 153.1272.

These data are in agreement with those reported previously in the literature.[24,38]

**(E)-3-phenylprop-2-en-1-ol (3p)**

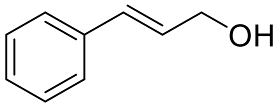

Prepared following general procedure 1, **3p** was obtained after purification by column chromatography (70:30 pentane/$Et_2O$) as a colorless oil. (20 mg, 147 µmol, 73%). $R_f$ = 0.30 (pentane:$Et_2O$ 6:4, vanillin).

**3p** was obtained as an inseparable mixture of A:B: (95:5).

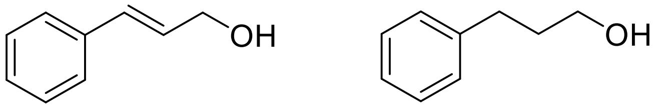

**A** **B**

**Major compound (A, fully described):** **$^1$H NMR** (400 MHz, Chloroform-*d*) δ 7.40 – 7.36 (m, 2H), 7.34 – 7.28 (m, , 2H), 7.26 – 7.22 (m, 1H), 6.68 – 6.56 (m, 1H), 6.36 (dt, *J* = 15.9, 5.7 Hz, 1H), 4.31 (dd, *J* = 5.7, 1.5 Hz, 2H).**$^{13}$C NMR** (101 MHz, Chloroform-*d*) δ 136.6, 131.1, 128.6, 128.5, 127.7, 126.4, 63.7. **HRMS (EI)** *m/z* $[M]^+$calculated for $[C_9H_{10}O]^+$: 134.0725, found: 134.0726. **IR (neat):** ν = 3290, 3028, 2896, 2856, 1492, 1448, 3172, 1274, 1213, 1090, 1010, 967, 916, 734, 688cm$^{-1}$.

**Minor compound (B, only distinct signals):** **$^1$H NMR** (400 MHz, Chloroform-*d*) δ 3.66 (t, *J* = 6.4 Hz, 2H), 2.70 (t, *J* = 8.1 Hz, 2H), 1.94 – 1.83 (m, 2H).

These data are in agreement with those reported previously in the literature.[39]

### 5.5 General procedure for the reductive aldol reaction (Procedure 5)

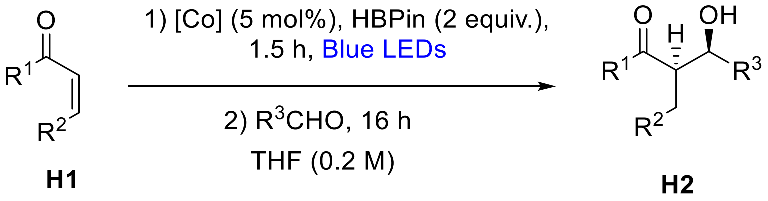

**General procedure:** In an oven-dried vial were introduced the cobalt catalyst (5 mol%, 10 µmol, 8.6 mg) and a stirring bar. A cap with rubber septum was used to close the vial and the system was then purged with Argon. THF (C = 0.1 M, 2 mL), enone **H1** (1 equiv., 0.2 mmol) and HBPin (2 equiv., 0.4 mmol, 58 µL) were then added successively and the vial was exposed to blue LEDs for 1.5 hours. After this time, the aldehyde was added (1.5 equiv, 0.3 mmol) neat, if liquid or dissolved in THF (0.2 ml) if solid. The resulting mixture was left at stirring for 16 hours. The crude was washed twice with a buffered aqueous solution (pH=8, $Na_2HPO_4/NaH_2PO_4$) and the aqueous layer extracted twice with $Et_2O$. The combined organic phase was then dried over $MgSO_4$ and concentrated in vacuum. The resulting residue was then purified through column chromatography to afford the corresponding aldol product **H2**.

#### 2-(hydroxy(phenyl)methyl)-4,4-dimethylcyclohexan-1-one (4a)

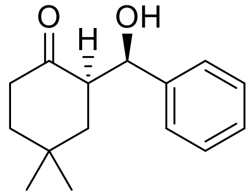

Prepared following general procedure 5, **4a** was obtained after purification by column chromatography (85:15 pentane/$Et_2O$) as a white solid. (34 mg, 146 µmol, 73%). $R_f$ = 0.42 (pentane:$Et_2O$ 6:4, vanillin). **$^1$H NMR** (600 MHz, Chloroform-*d*) δ 7.36 – 7.32 (m, 2H), 7.30 – 7.27 (m, 2H), 7.25 – 7.23 (m, 1H), 5.41 (t, *J* = 3.0 Hz, 1H), 2.97 (d, *J* = 3.5 Hz, 1H), 2.75 (dddd, *J* = 13.4, 5.6, 2.6, 1.0 Hz, 1H), 2.58 – 2.50 (m, 1H), 2.31 (ddd, *J* = 14.4, 4.6, 2.8 Hz, 1H), 1.74 – 1.62 (m, 3H), 1.34 (ddd, *J* = 13.5, 5.6, 3.4 Hz, 1H), 1.05 (s, 3H), 0.95 (s, 3H).. **$^{13}$C NMR** (151 MHz, Chloroform-d) δ 215.3, 141.4, 128.2, 126.9, 125.6, 70.5, 52.7, 39.9, 38.7, 38.1, 31.4, 30.4, 24.3. **HRMS (ESI)** *m/z* $[M+Na]^+$calculated for $[C_{15}H_{20}O_2Na]^+$: 255.1355, found: 255.1347. **IR (neat):** v = 3496, 3419, 2948, 2864, 1696, 1491, 1448, 1317, 1183, 1155, 1033, 749, 698, 659 $cm^{-1}$.

#### 2-(hydroxy(phenyl)methyl)cyclohexan-1-one (4b)

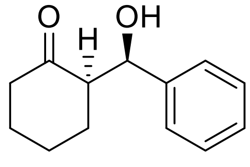

Prepared following general procedure 5, **4b** was obtained after purification by column chromatography (90:9:1 pentane/$Et_2O$/AcOH) as a white solid. (34 mg, 166 µmol, 83%). $R_f$ = 0.31 (pentane:$Et_2O$ 6:4, vanillin). **$^1$H NMR** (600 MHz, Chloroform-*d*) δ 7.29 – 7.22 (m, 4H), 7.20 – 7.16 (m, 1H), 5.32 (d, *J* = 2.8 Hz, 1H), 2.94 (d, *J* = 3.3 Hz, 1H), 2.55 – 2.51 (m, 1H), 2.41 – 2.36 (m, 1H), 2.30 (tdd, *J* = 13.5, 6.1, 1.3 Hz, 1H), 2.01 (ddt, *J* = 13.3, 5.9, 2.9 Hz, 1H), 1.81 – 1.74 (m, 1H), 1.68 (dd, *J* = 10.6, 3.7 Hz, 1H), 1.61 (dtt, *J* = 21.4, 13.3, 3.9 Hz, 1H), 1.49 – 1.40 (m, 1H).. **$^{13}$C NMR** (151 MHz, Chloroform-d) δ 214.8, 141.5, 128.2, 127.0, 125.8, 70.6, 57.2, 42.7, 28.0, 26.0, 24.9. **HRMS (ESI)** *m/z* $[M+Na]^+$calculated for $[C_{13}H_{16}O_2Na]^+$: 227.1048, found: 277.1055. **IR (neat):** v = 3505, 2942, 2864, 1694, 1596, 1492, 1448, 1313, 1255, 1198, 1132, 1066, 1033, 746, 697 $cm^{-1}$.

These data are in agreement with those reported previously in the literature.[40]

#### 2-(hydroxy(phenyl)methyl)cyclopentan-1-one (4c)

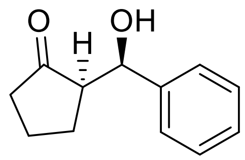

Prepared following general procedure 5, **4c** was obtained after purification by column chromatography (90:9:1 pentane/$Et_2O$/AcOH) as a colorless oil. (28 mg, 148 µmol, 74%). $R_f$ = 0.50 (pentane:$Et_2O$ 6:4, vanillin). **$^1$H NMR** (600 MHz, Chloroform-*d*) δ 7.28 – 7.24 (m, 4H), 7.18 (m, 1H), 5.22 (d, *J* = 3.1 Hz, 1H), 2.57 (s, 1H), 2.37 (ddd, *J* = 11.9, 6.2, 2.3 Hz, 1H), 2.30 – 2.23 (m, 1H), 2.06 (ddd, *J* = 19.0, 11.0, 8.6 Hz, 1H), 2.01 – 1.87 (m, 2H), 1.78 – 1.69 (m, 1H), 1.66 – 1.55 (m, 1H). **$^{13}$C NMR** (151 MHz, Chloroform-d) δ 220.6, 142.8, 128.4, 127.3, 125.6, 71.5, 56.2, 39.2, 22.8, 20.5. **HRMS (ESI)** *m/z* $[M+Na]^+$ calculated for $[C_{12}H_{14}O_2Na]^+$: 213.0886, found:213.0888. **IR (neat):** v = 3400, 2964, 2884, 1723, 1449, 1403, 1332, 1264, 1156, 1107, 1025, 968, 725, 698, 669 $cm^{-1}$.

These data are in agreement with those reported previously in the literature.[41]

**2-(hydroxy(phenyl)methyl)cycloheptan-1-one (4d)**

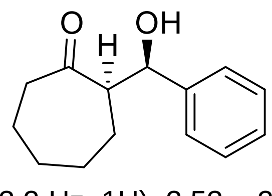

Prepared following general procedure 5, **4d** was obtained after purification by column chromatography (80:20 pentane/$Et_2O$) as a colorless oil (28 mg, 130 µmol, 65%). $R_f$ = 0.60 (pentane:$Et_2O$ 6:4, vanillin). **$^1$H NMR** (600 MHz, Chloroform-*d*) δ 7.30 – 7.23 (m, 4H), 7.21 – 7.15 (m, 1H), 5.11 (d, *J* = 3.0 Hz, 1H), 3.27 (s, 1H), 2.76 (dd, *J* = 10.9, 3.2 Hz, 1H), 2.53 – 2.46 (m, 1H), 2.38 (ddd, *J* = 15.6, 11.9, 3.5 Hz, 1H), 1.83 – 1.74 (m, 3H), 1.69 (ddd, *J* = 14.3, 6.2, 3.0 Hz, 1H), 1.55 (dd, *J* = 11.4, 3.9 Hz, 1H), 1.46 – 1.35 (m, 1H), 1.25 – 1.12 (m, 2H). **$^{13}$C NMR** (151 MHz, Chloroform-d) δ 218.2, 142.0, 128.2, 127.2, 125.9, 73.4, 57.8, 44.0, 29.2, 29.1, 24.2, 23.8. **HRMS (ESI)** *m/z* $[M+Na]^+$ calculated for $[C_{14}H_{18}O_2Na]^+$: 242.1199, found: 242.1191. **IR (neat):** v = 3434, 2927, 2856, 1687, 1450, 1343, 1043, 926, 763, 735, 701 $cm^{-1}$.

These data are in agreement with those reported previously in the literature.[42]

**6-(hydroxy(phenyl)methyl)-6,7,8,9-tetrahydro-5H-benzo[7]annulen-5-one (4e)**

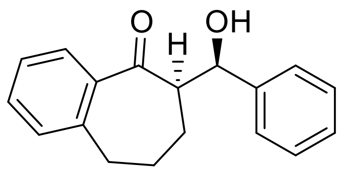

Prepared following general procedure 5, **4e** was obtained after purification by column chromatography (80:20 pentane/$Et_2O$) as a white solid. (37 mg, 156 µmol, 78%). $R_f$ = 0.65 (pentane:$Et_2O$ 6:4, vanillin). **$^1$H NMR** (600 MHz, Chloroform-*d*) δ 7.68 (dd, *J* = 7.7, 1.5 Hz, 1H), 7.34 (td, *J* = 7.5, 1.5 Hz, 1H), 7.32 – 7.22 (m, 4H), 7.21 – 7.13 (m, 3H), 5.34 (s, 1H), 3.84 (s, 1H), 3.11 – 3.06 (m, 1H), 2.92 – 2.77 (m, 2H), 1.95 (ddd, *J* = 24.4, 12.9, 6.0 Hz, 2H), 1.60 – 1.50 (m, 2H). **$^{13}$C NMR** (151 MHz, Chloroform-d) δ 208.8, 143.4, 141.7, 138.8, 131.9, 130.2, 128.5, 128.2, 127.1, 126.5, 125.9, 72.7, 55.6, 33.7, 25.4, 22.4. **HRMS (ESI)** *m/z* $[M+Na]^+$calculated for $[C_{18}H_{18}O_2Na^+]$: 289.1199, found:289.1204. **IR (neat):** v = 3448, 2926, 2860, 1654, 1596, 1449, 1287, 1251, 1092, 1045, 962, 762, 701 $cm^{-1}$.

**2-(hydroxy(phenyl)methyl)-2-methylcyclopentan-1-one (4f)**

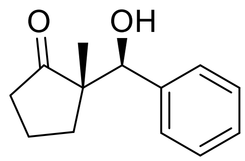

Prepared following general procedure 5, **4f** was obtained after purification by column chromatography (80:20 pentane/$Et_2O$) as a colorless oil. (24 mg 120 µmol, 60%). $R_f$ = 0.35 (pentane:$Et_2O$ 6:4, vanillin). **$^1$H NMR** (600 MHz, Chloroform-d) δ 7.36 – 7.30 (m, 4H), 7.29 – 7.25 (m, 1H), 4.80 (s, 1H), 4.12 (d, *J* = 1.3 Hz, 1H), 2.43 (dddd, *J* = 19.3, 8.5, 3.3, 1.8 Hz, 1H), 2.23 (dt, *J* = 19.2, 9.5 Hz, 1H), 1.97 (ddd, *J* = 12.9, 10.7, 7.2 Hz, 1H), 1.91 – 1.75 (m, 2H), 1.47 – 1.39 (m, 1H), 1.06 (s, 3H).. **$^{13}$C NMR** (151 MHz, Chloroform-d) δ 226.0, 139.5, 127.9, 127.2, 52.3, 38.0, 34.1, 18.5, 15.7. **HRMS (ESI)** *m/z* $[M+Na]^+$ calculated for $[C_{13}H_{16}O_2Na]^+$: 227.1035, found: 227.1043. **IR (neat):** v = 3457, 2963, 2875, 1720, 1452, 1404, 1197, 1163, 1043, 772, 713 $cm^{-1}$

These data are in agreement with those reported previously in the literature.[43]

**3-(hydroxy(phenyl)methyl)nonan-2-one (4g)**

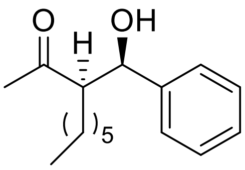

Prepared following general procedure 5, **4g** was obtained after purification by column chromatography (90:9:1 pentane/$Et_2O$/AcOH) as a colorless oil. (27 mg, 108 µmol, 54%). $R_f$ = 0.72 (pentane:$Et_2O$ 6:4, vanillin).

**$^1$H NMR** (600 MHz, Chloroform-d) δ 7.28 – 7.23 (m, 4H), 7.19 (m, 1H), 4.79 (d, *J* = 5.9 Hz, 1H), 2.80 (ddd, *J* = 9.7, 5.9, 4.0 Hz, 1H), 2.66 (s, 1H), 1.92 (s, 3H), 1.70 – 1.50 (m, 2H), 1.22 – 1.00 (m, 8H), 0.78 (t, *J* = 7.1 Hz, 3H). **$^{13}$C NMR** (151 MHz, Chloroform-d) δ 213.2, 142.0, 128.4, 127.7, 126.2, 74.1, 59.6, 31.8, 29.5, 27.8, 27.3, 22.5, 14.0. **HRMS (ESI)** *m/z* $[M+Na]^+$calculated for $[C_{16}H_{24}O_2Na]^+$: 271.1668, found: 271.1672. **IR (neat):** ν = 3423, 2925, 2858, 1702, 1455, 1419, 1356, 1164, 1046, 763, 700 $cm^{-1}$.

**(2R,8R,9S,10R,13S,14S,17S)-2-(hydroxy(phenyl)methyl)-10,13,17-trimethyl-17-((trimethylsilyl)oxy)-1,2,6,7,8,9,10,11,12,13,14,15,16,17-tetradecahydro-3H-cyclopenta[a]phenanthren-3-one (4h)**

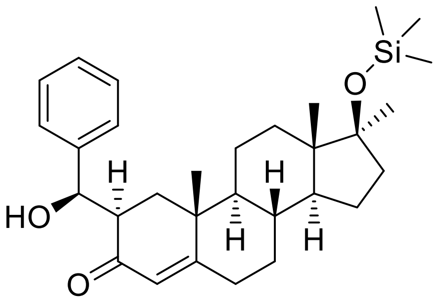

Prepared following general procedure 5, **4g** was obtained after purification by column chromatography (70:30 70:30 pentane/$Et_2O$) as a colorless oil. (27 mg, 102 µmol, 51%). $R_f$ = 0.72 (pentane:$Et_2O$ 7:3, vanillin). **$^1$H NMR** (400 MHz, Chloroform-*d*) δ 7.38 – 7.30 (m, 4H), 7.26 (m, 1H), 5.76 (d, *J* = 1.5 Hz, 1H), 5.54 (t, *J* = 3.9 Hz, 1H), 3.08 (d, *J* = 5.1 Hz, 1H), 2.74 (ddd, *J* = 10.7, 7.6, 2.9 Hz, 1H), 2.39 – 2.22 (m, 2H), 1.92 – 1.78 (m, 2H), 1.63 (dd, *J* = 10.3, 6.7 Hz, 3H), 1.60 – 1.48 (m, 2H), 1.47 – 1.36 (m, 2H), 1.33 – 1.19 (m, 3H), 1.14 (s, 3H), 1.06 (s, 3H), 0.95 (dd, *J* = 12.3, 4.5 Hz, 1H), 0.91 – 0.78 (m, 2H), 0.75 (s, 3H), 0.07 (s, 9H).**$^{13}$C NMR** (101 MHz, Chloroform-*d*) δ 201.0, 172.2, 141.7, 128.2, 126.9, 125.8, 123.7, 83.7, 71.3, 54.2, 49.0, 48.7, 46.2, 39.0, 38.9, 36.4, 34.9, 32.6, 31.6, 31.1, 26.2, 23.4, 20.5, 17.5, 14.1, 2.4. **HRMS (ESI)** *m/z* $[M+Na]^+$ calculated for $[C_{30}H_{44}O_3SiNa]^+$: 503.2952, found: 503.2958. **IR (neat):** ν = 3442, 2948, 1660, 1450, 1249, 1168, 1092, 1065, 1019, 877, 836, 730, 699 $cm^-$

**2-((4-ethoxyphenyl)(hydroxy)methyl)-4,4-dimethylcyclohexan-1-one (4i)**

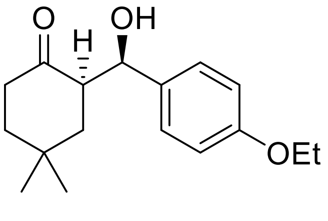

Prepared following general procedure 5, **4i** was obtained after purification by column chromatography (90:9:1 pentane/$Et_2O$/AcOH) as a white solid. (45 mg, 162 µmol 81%). $R_f$ = 0.63 (pentane:$Et_2O$ 6:4, vanillin). **$^1$H NMR** (600 MHz, Chloroform-*d*) δ 7.19 (m , 2H), 6.94 – 6.72 (m, 2H), 5.34 (t, *J* = 3.1 Hz, 1H), 4.03 (q, *J* = 7.0 Hz, 2H), 2.94 (d, *J* = 3.5 Hz, 1H), 2.71 (ddd, *J* = 13.8, 6.0, 2.8 Hz, 1H), 2.53 (td, *J* = 14.0, 6.4 Hz, 1H), 2.30 (ddd, *J* = 14.3, 4.6, 2.8 Hz, 1H), 1.76 – 1.63 (m, 3H), 1.45 – 1.35 (m, 4H), 1.06 (s, 3H), 0.96 (s, 3H). **$^{13}$C NMR** (151 MHz, Chloroform-d) δ 215.4, 157.9, 133.3, 126.7, 114.2, 70.3, 63.4, 52.7, 39.9, 38.7, 38.2, 31.4, 30.4, 24.3, 14.9. **HRMS (ESI)** *m/z* $[M+Na]^+$calculated for $[C_{17}H_{24}O_3Na]^+$: 299.1618, found:299.1623. **IR (neat):** ν = 3433, 2927, 2870, 1700, 1610, 1511, 1473, 1244, 1175, 1115, 1091, 1049, 845, 816, 659 $cm^{-1}$.

**2-(hydroxy(p-tolyl)methyl)-4,4-dimethylcyclohexan-1-one (4j)**

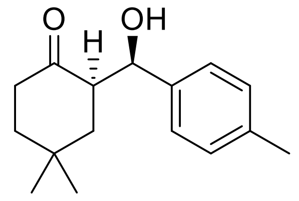

Prepared following general procedure 5, **4j** was obtained after purification by column chromatography (90:9:1 pentane/$Et_2O$/AcOH) as a white solid. (27 mg, 110 µmol, 55%). $R_f$ = 0.59 (pentane:$Et_2O$ 6:4, vanillin). **$^1$H NMR** (600 MHz, Chloroform-*d*) δ 7.17 (d, *J* = 7.9 Hz, 2H), 7.14 (d, *J* = 8.0 Hz, 2H), 5.39 – 5.36 (m, 1H), 2.96 (s, 1H), 2.74 (ddd, *J* = 13.4, 5.6, 2.5 Hz, 1H), 2.53 (td, *J* = 14.0, 6.5 Hz, 1H), 2.34 (s, 3H), 2.31 (ddd, *J* = 14.4, 4.5, 2.9 Hz, 1H), 1.74 – 1.62 (m, 3H), 1.38 (ddd, *J* = 13.5, 5.7, 3.4 Hz, 1H), 1.06 (s, 3H), 0.96 (s, 3H). **$^{13}$C NMR** (151 MHz, Chloroform-d) δ 215.3, 138.4, 136.4, 128.9, 125.5, 70.4, 52.7, 39.9, 38.7, 38.1, 31.4, 30.4, 24.3, 21.1. **HRMS (ESI)** *m/z* $[M+Na]^+$ calculated for $[C_{16}H_{22}O_2Na]^+$: 269.1512, found: 269.1503. **IR (neat):** ν = 3419, 2947, 2865, 1700, 1512, 1643, 1399, 1368, 1154, 1092, 1043, 836, 784, 659 $cm^{-1}$.

**2-((4-chlorophenyl)(hydroxy)methyl)-4,4-dimethylcyclohexan-1-one (4k)**

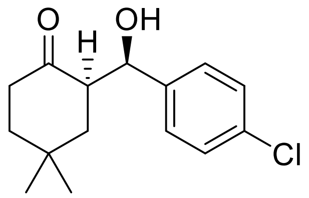

Prepared following general procedure 5, **4k** was obtained after purification by column chromatography (70:30 pentane/$Et_2O$) as a white solid. (29 mg, 156 µmol, 78%). $R_f$ = 0.41 (pentane:$Et_2O$ 6:4, vanillin). **$^1$H NMR** (600 MHz, Chloroform-*d*) δ 7.31 (d, *J* = 8.2 Hz, 2H), 7.23 (d, *J* = 8.1 Hz, 2H), 5.38 (t, *J* = 3.0 Hz, 1H), 3.00 (d, *J* = 3.4 Hz, 1H), 2.71 (ddd, *J* = 13.5, 5.7, 2.4 Hz, 1H), 2.54 (td, *J* = 14.1, 6.4 Hz, 1H), 2.32 (dt, *J* = 14.4, 3.7 Hz, 1H), 1.80 – 1.60 (m, 3H), 1.28 (ddd, *J* = 13.5, 5.6, 3.6 Hz, 1H), 1.06 (s, 3H), 0.96 (s, 3H). **$^{13}$C NMR** (151 MHz, Chloroform-d) δ 215.1, 139.9, 132.6, 128.4, 127.0, 70.0, 52.6, 39.9, 38.6, 38.1, 31.4, 30.4, 24.3. **HRMS (ESI)** *m/z* $[M+Na]^+$ calculated for $[C_{15}H_{19}O_2ClNa]^+$: 289.0966, found: 289.0970. **IR (neat):** ν = 3470, 2953, 2925, 1690, 1490, 197, 1311, 1257, 1212, 1185, 1161, 1088, 1043, 1011, 838, 802 $cm^{-1}$.

**2-(-hydroxy(4-(trifluoromethyl)phenyl)methyl)-4,4-dimethylcyclohexan-1-one (4l)**

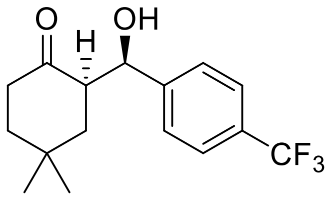

Prepared following general procedure 5, **4l** was obtained after purification by column chromatography (70:30 pentane/$Et_2O$) as a white solid. (41 mg, 136 µmol, 68%). $R_f$ = 0.45 (pentane:$Et_2O$ 6:4, vanillin). **$^1$H NMR** (600 MHz, Chloroform-*d*) δ 7.60 (d, *J* = 8.1 Hz, 2H), 7.41 (d, *J* = 8.1 Hz, 2H), 5.47 (s, 1H), 3.04 (s, 1H), 2.83 – 2.70 (m, 1H), 2.56 (td, *J* = 14.1, 6.4 Hz, 1H), 2.34 (ddd, *J* = 14.4, 4.6, 2.8 Hz, 1H), 1.79 – 1.61 (m, 3H), 1.06 (s, 3H), 0.96 (s, 3H). **$^{19}$F NMR** (565 MHz, Chloroform-*d*) δ -62.39. **HRMS (ESI)** *m/z* $[M+Na]^+$ calculated for $[C_{16}H_{19}O_2F_3Na]^+$: 323.1229, found: 323.1235. **IR (neat):** ν = 3414, 2951, 2929, 1699, 1329, 1154, 1123, 1095, 1068, 1015, 849, 659 $cm^{-1}$.

**2-(furan-2-yl(hydroxy)methyl)-4,4-dimethylcyclohexan-1-one (4m)**

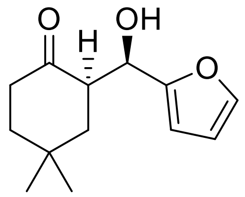

Prepared following general procedure 5, **4m** was obtained after purification by column chromatography (85:15 pentane/Et2O) as a white solid. (39 mg, 168 µmol 84%). $R_f$ = 0.62 (pentane:Et2O 6:4, vanillin). **$^1$H NMR** (600 MHz, Chloroform-*d*) δ 7.41 – 7.30 (m, 1H), 6.33 (dd, *J* = 3.2, 1.8 Hz, 1H), 6.25 (dd, *J* = 3.2, 1.0 Hz, 1H), 5.34 – 5.20 (m, 1H), 3.07 (d, *J* = 5.2 Hz, 1H), 3.03 – 2.85 (m, 1H), 2.61 – 2.46 (m, 1H), 2.31 (ddd, *J* = 14.7, 4.7, 2.8 Hz, 1H), 1.77 – 1.61 (m, 3H), 1.54 (ddd, *J* = 13.5, 5.7, 3.4 Hz, 1H), 1.16 (s, 3H), 1.00 (s, 3H).. **$^{13}$C NMR** (151 MHz, Chloroform-d) δ 214.1, 154.6, 141.5, 110.2, 106.6, 66.7, 50.1, 39.5, 39.4, 38.5, 31.4, 30.4, 24.4. **HRMS (ESI)** *m/z* $[M+Na]^+$ calculated for $[C_{13}H_{18}O_3Na]^+$: 245.1154, found:.**IR (neat):** ν = 3417, 2945,2865, 1698, 1365, 1254,1148, 1116, 1002, 953, 816, 795, 733 $cm^{-1}$.

**2-(hydroxy(thiophen-2-yl)methyl)-4,4-dimethylcyclohexan-1-one (4n)**

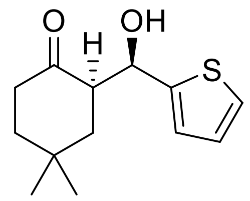

Prepared following general procedure 5, **4n** was obtained after purification by column chromatography (50:50 pentane/$Et_2O$) as a white solid. (29 mg, 122 µmol, 61%). $R_f$ = 0.63 (pentane:$Et_2O$ 6:4, vanillin). **$^1$H NMR** (600 MHz, Chloroform-*d*) δ 7.15 (d, *J* = 5.0 Hz, 1H), 6.91 (t, *J* = 4.2 Hz, 1H), 6.83 (d, *J* = 3.6 Hz, 1H), 5.49 (d, *J* = 3.6 Hz, 1H), 3.08 (d, *J* = 4.3 Hz, 1H), 2.80 (ddd, *J* = 13.5, 5.8, 2.8 Hz, 1H), 2.48 (td, *J* = 14.1, 6.4 Hz, 1H), 2.25 (dt, *J* = 14.3, 3.9 Hz, 1H), 1.72 – 1.44 (m, 4H), 1.08 (s, 3H), 0.93 (s, 3H). **$^{13}$C NMR** (151 MHz, Chloroform-d) δ 214.5, 145.3, 126.7, 124.1, 123.1, 68.5, 52.7, 39.7, 38.8, 38.6, 31.4, 30.5, 24.3. **HRMS (ESI)** *m/z* $[M+Na]^+$ calculated for $[C_{13}H_{18}O_2SNa]^+$: 261.0920, found:261.0923. **IR (neat):** ν = 3410, 2956, 2928, 1695, 1443, 1365, 1310, 1216, 1156, 1121, 1094, 1039, 837, 704 $cm^{-1}$.

**2-(E-1-hydroxy-3-phenylallyl)-4,4-dimethylcyclohexan-1-one (4o)**

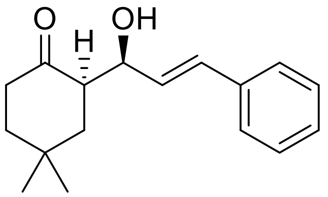

Prepared following general procedure 5, **4o** was obtained after purification by column chromatography (90:9:1 pentane/$Et_2O$/AcOH) as a white solid. (22 mg, 86 µmol, 43%). $R_f$ = 0.54 (pentane:$Et_2O$ 6:4, vanillin). **$^1$H NMR** (600 MHz, Chloroform-*d*) δ 7.41 – 7.38 (m, 2H), 7.32 (m, 2H), 7.26 – 7.22 (m, 1H). 6.62 (dd, *J* = 16.0, 1.6 Hz, 1H), 6.19 (dd, *J* = 15.9, 5.9 Hz, 1H). 4.82 – 4.70 (m, 1H), 2.95 (d, *J* = 5.2 Hz, 1H), 2.71 (ddd, *J* = 12.2, 6.6, 2.9 Hz, 1H), 2.51 (td, *J* = 14.2, 6.2 Hz, 1H), 2.30 (ddd, *J* = 14.7, 4.7, 2.6 Hz, 1H), 1.77 – 1.61 (m, 4H), 1.19 (s, 3H), 1.03 (s, 3H).. **$^{13}$C NMR** (151 MHz, Chloroform-d) δ 214.8, 136.8, 131.0, 128.9, 128.6, 127.6, 126.5, 70.7, 51.2, 39.6, 39.5, 38.6, 31.5, 30.5, 24.4. **HRMS (ESI)** *m/z* $[M+Na]^+$ calculated for $[C_{17}H_{22}O_2Na]^+$: 281.1512, found: 281.1517. **IR (neat):** ν = 3433, 3056, 3027, 2927, 2859, 1701, 1447, 1327, 1154, 1127, 967, 742, 693 $cm^{-1}$.

**2-(1-hydroxy-2-methylpropyl)-4,4-dimethylcyclohexan-1-one (4p)**

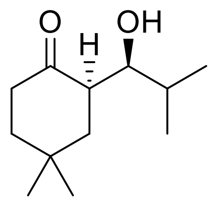

Prepared following general procedure 5, **4p** was obtained after purification by column chromatography (85:15 pentane/$Et_2O$) as a colorless oil. (26 mg, 132 µmol, 66%). $R_f$ = 0.71 (pentane:$Et_2O$ 6:4, vanillin). **$^1$H NMR** (600 MHz, Chloroform-*d*) δ 3.52 (dd, *J* = 7.6, 3.6 Hz, 1H), 2.65 – 2.54 (m, 1H), 2.48 (tdd, *J* = 14.1, 6.2, 1.2 Hz, 1H), 2.25 (ddd, *J* = 14.2, 4.7, 2.6 Hz, 1H), 1.80 – 1.69 (m, 3H), 1.69 – 1.58 (m, 3H), 1.40 (t, *J* = 13.4 Hz, 1H), 1.21 (s, 3H), 1.01 (s, 3H), 0.98 (d, *J* = 6.8 Hz, 3H), 0.86 (d, *J* = 6.8 Hz, 3H). **$^{13}$C NMR** (151 MHz, Chloroform-d) δ 216.8, 75.4, 49.4, 43.0, 39.7, 39.0, 31.4, 30.6, 28.9, 24.5, 20.1, 14.8. **HRMS (ESI)** *m/z* $[M+Na]^+$ calculated for $[C_{12}H_{22}O_2Na]^+$: 221.1512, found: 221.1502. **IR (neat):** ν = 3377, 2955, 2869, 1688, 1617, 1463, 1365, 1303, 1176, 1142, 857, 834 $cm^{-1}$.

# 6. Mechanistic Experiments

## 6.1 Temperature dependence of the reaction in the dark

The hydroboration of 4,4-dimethylcylohexenone has been carried out following the general procedure 3 with the difference that the reaction vial has been kept for the whole reaction time in an heated oil bath at the given temperature.

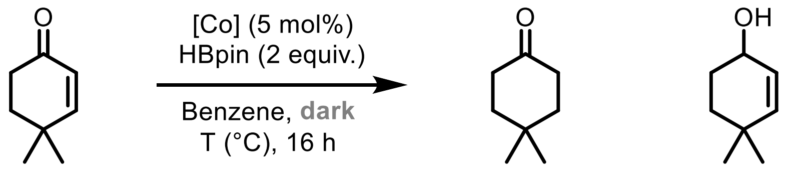


**1k**

| **T (°C)** | **2k** | **3k** |
|---|---|---|
| 25 | 5% | 89% |
| 50 | 6% | 36% |
| 75 | 25% | 31% |

## 6.2 1,2 hydroboraton of carvone and attempted isomerization

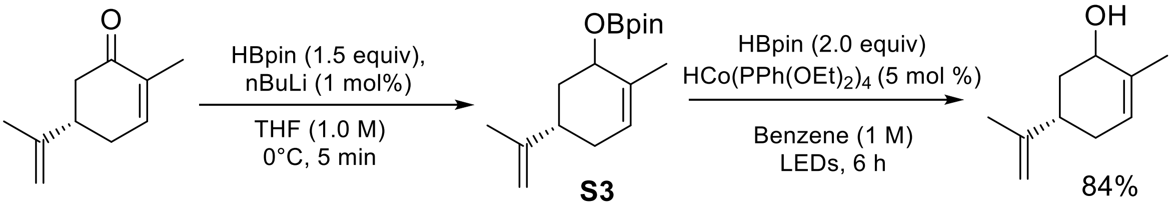


**S3** 84%

In a schlenk tube under argon carvone (1 equiv, 0.1 mmol) was dissolved in dry THF (c= 1M, 1.0 ml) and the resulting solution was cooled at 0°C. HBpin (1.5 equiv, 1.5 mmol) was added followed by nBuli (1 mol%, 0.001 mmol, 2.5 M in hexane). After 5 minutes the volatiles were removed under high vacuum. The colorless oil obtained was used without further purification. Dry benzene (c = 1 M, 0.1 ml) was added under Ar; the cobalt catalyst (5 mol %, 0.005 mmol) was then quickly added as a solid followed by HBpin (2 equiv, 0.2 mmol). The reaction mixture has been irradiated with Blue LEDs for 6 hours. After this time, the solution was diluted with $Et_2O$ and washed two times with distilled water, the combined aqueous layers were extracted two times with $Et_2O$ and the combined organic layers were washed with brine, dried ($MgSO_4$), filtered and concentrated under vacuum. $^1$H NMR analysis of the residue revealed that only the 1,2 reduced product was formed (84%, $CH_2Br_2$ as internal standard)

### 4,4,5,5-tetramethyl-2-(((5S)-2-methyl-5-(prop-1-en-2-yl)cyclohex-2-en-1-yl)oxy)-1,3,2-dioxaborolane (S3)

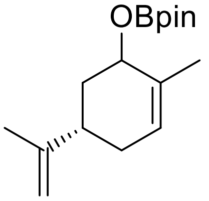


**$^1$H NMR** (400 MHz, Benzene-$d_6$) δ 5.38 – 5.31 (m, 1H), 4.90 – 4.81 (m, 1H), 4.70 – 4.67 (m, 2H), 2.23 – 2.17 (m, 1H), 2.16 – 2.09 (m, 1H), 1.83 – 1.66 (m, 6H), 1.53 (s, 3H), 1.08 (s, 12H). **$^{13}$C NMR** (101 MHz, Benzene-$d_6$) δ 148.8, 135.9, 124.0, 109.5, 82.5, 73.4, 41.0, 37.2, 31.4, 24. 7, 20.4, 19.2.

These data are in agreement with those reported previously in the literature.[44]

# 7. Computational details

## 7.1 General computational details

The computational study was carried out using Gaussian16 program package(A.03)[45] at the B3LYP-D3 level of theory[46] (this functional has been successfully used for low-valent cobalt homogeneous catalysis,[47] other functionals were also tested for the light-selectivity determining step but not significant differences were found, see below). The combination of LANL2DZ basis set (for Co) and 6-31G(d) was used for optimization and frequency calculations. In addition, potential energies were further refined using the larger basis set Def2TZVPP for all atoms. All the calculations (optimizations and single points) were computed using the CPCM implicit solvation model with benzene as solvent. Minima and transition states were analyzed by frequency calculation to check the nature of stationary points (zero imaginary frequencies for minima and one for transition states). Thermochemistry corrections were added at standard conditions (298.15 K). Additionally, 1M standard state correction was applied by adding 1.89 kcal/mol when needed. Spin state was checked for all the relevant steps (singlet or triplet for Co(I) species and doublet or quartet for Co(0) species), finding low spin state more stable. The Minimum Energy Crossing Point (MECP) to study the inner-sphere single electron transfer (see below, Figure S3) was calculated using the EasyMECP tool[48,49]

3D structures were prepared using CYLview.[50]

Ethyl groups on the phosphonite ligand were replaced by methyl groups to reduce the conformational space of the system. We validated that simplification by comparing the highest barrier of the mechanism using the real ligand and the simplified one, being both free energy barriers very similar.

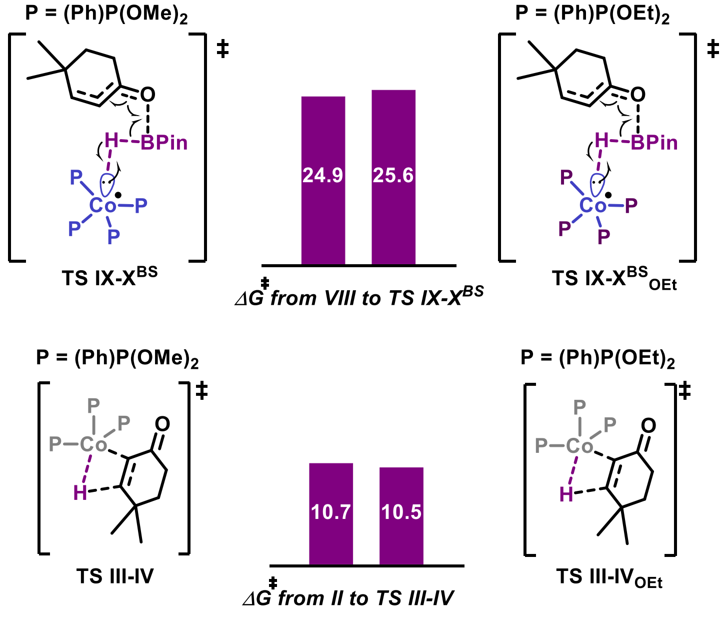

### 7.2 Benchmark of the computational method:

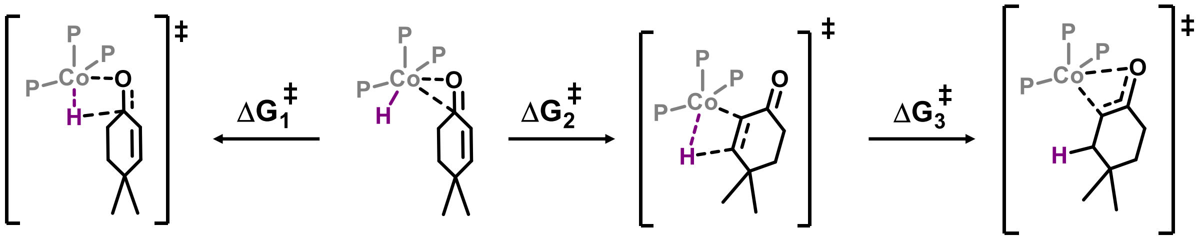


| Functional | $\Delta G_1^‡$ | $\Delta G_2^‡$ | $\Delta G_3^‡$ | $\Delta\Delta G^‡$ |
|---|---|---|---|---|
| B3LYP-D3 | 18.4 | 10.7 | 13.5 | 4.9 |
| M06 | 17.2 | 11.4 | 11.1 | 5.8 |
| M06L | 19.9 | 13.5 | 15.3 | 4.6 |
| wB97xD | 14.5 | 8.2 | 9.9 | 4.6 |

**Table S3. Functional benchmarking of the light mediated Co(I) hydroboration. Energies in kcal/mol. $\Delta\Delta G^‡$ refers to the difference between 1,2 free energy barrier ($\Delta G_1^‡$) and the highest of 1,4 hydroboration pathway ($\Delta G_1^‡$ or $\Delta G_1^‡$)**

### 7.3 Complementary pathways:

### 'Light' Pathways:

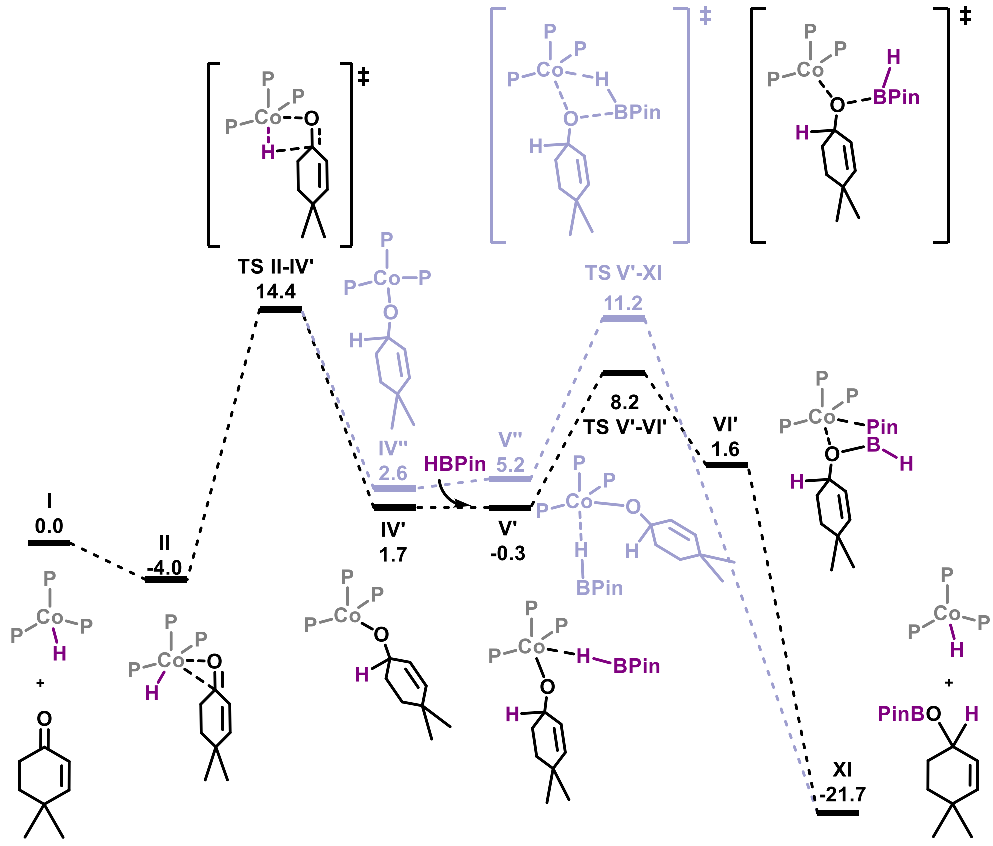


**Figure S1. Free energy profile of light-mediated 1,2 hydroboration of cyclohexenone catalysed by Co(I). Energies in kcal/mol. P = P(Ph)(OMe)$_2$**

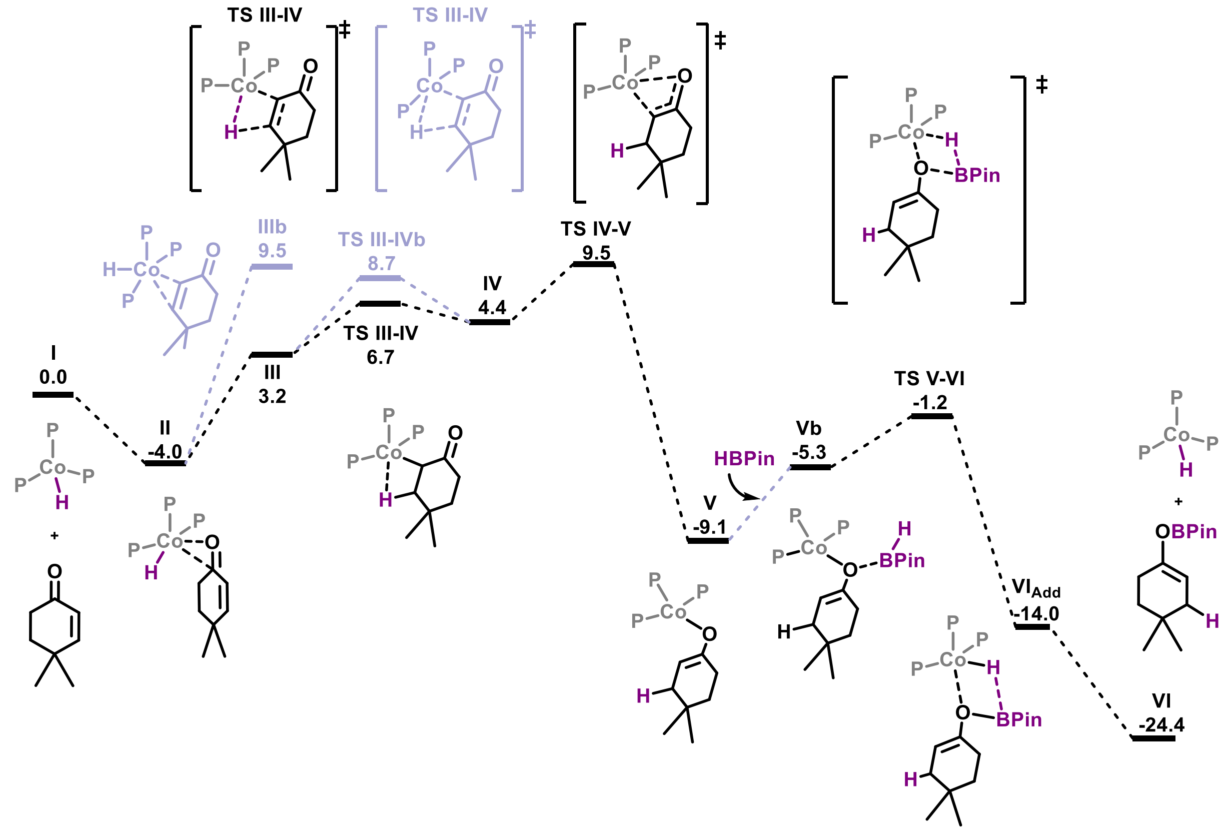


**Figure S2. Detailed free energy profile of light-mediated 1,4 hydroboration of cyclohexenone catalysed by Co(I). Energies in kcal/mol. P = (P(Ph)(OMe)$_2$**

## 'Dark' pathways:

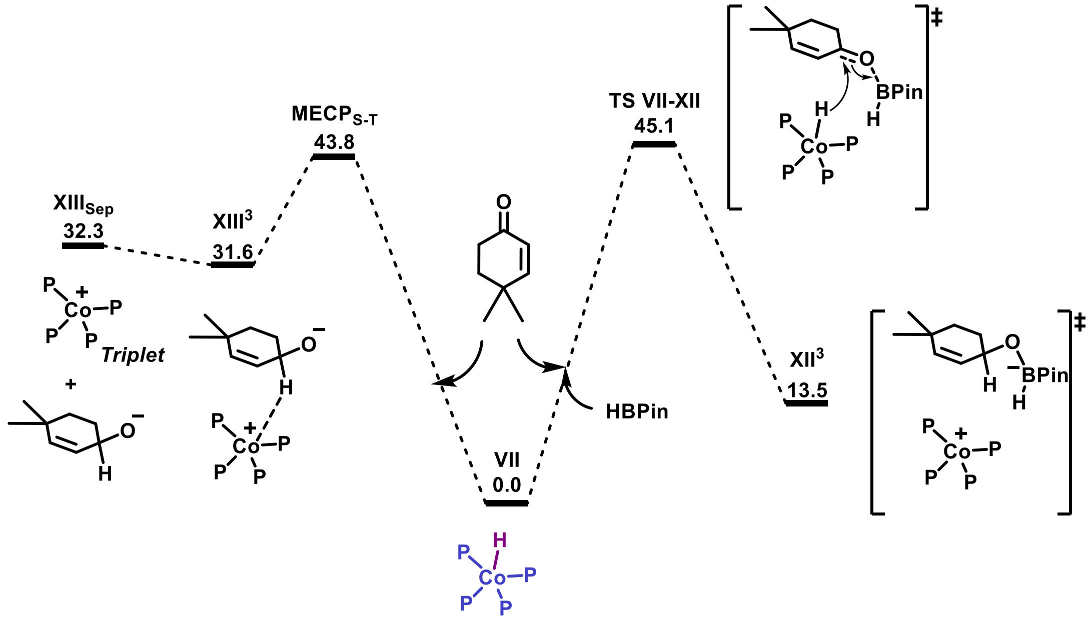


**Figure S3. Hydrogen transfer pathways from Co(I)-H. Free energies in kcal/mol. $^3$ superscript means triplet state. P = (P(Ph)(OMe)$_2$**

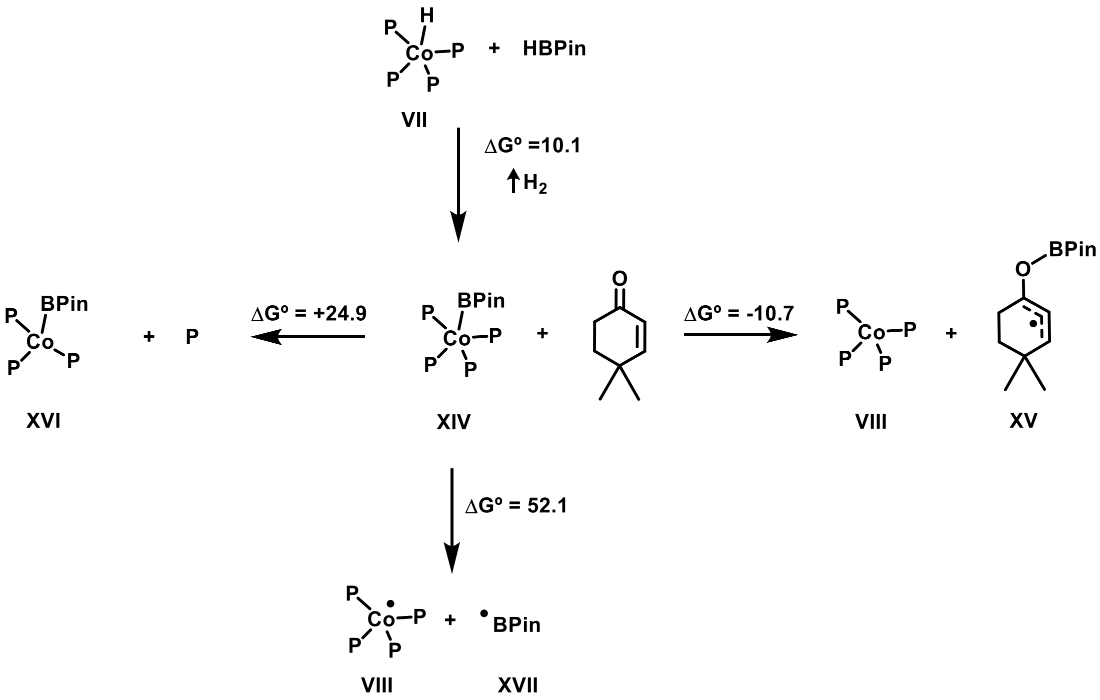


**Figure S4. Alternative pathway for Co(0) formation via catalyst reduction with HBPin. Energies in kcal/mol. P = (P(Ph)(OMe)$_2$**

Due to the experimental detection of $H_2$, we explored the formation of Co-Bpin species. According to the mechanism proposed in our previous manuscript on cobalt catalysis[51] we calculated the dissociation of a phosphine ligand after CoBPin formation but it is endergonic by 24.9 kcal/mol. However, the formation of P4-Co(0) species is much more favored, through a single electron transfer from the cyclohexenone substrate to Co-Bpin species. The homolytic

Co-B bond cleavage was also explored and was found highly endergonic, discarding the formation of free BPin radical in solution.

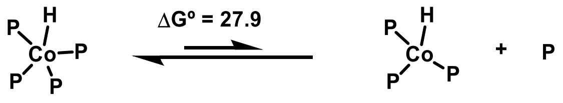


VII I

**Figure S5. Thermal ligand dissociation in $P_4Co(I)$-H catalyst. Energies in kcal/mol. P = $(P(Ph)(OMe)_2$**

The high energy of ligand dissociation excludes this pathway under thermal conditions, since the overall barrier of light mechanism would be added to that equilibrium, yielding an energy span of more than 35 kcal/mol.

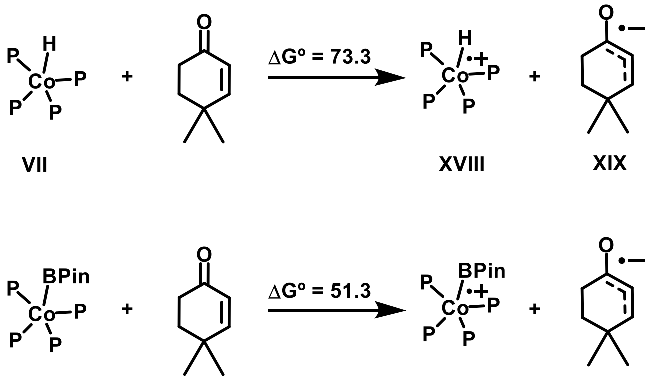


VII XVIII XIX

XIV XX

**Figure S6. Co(II) formation through Single Electron Transfer from Co(I)-H to the substrate. Energies in kcal/mol. P = $(P(Ph)(OMe)_2$**

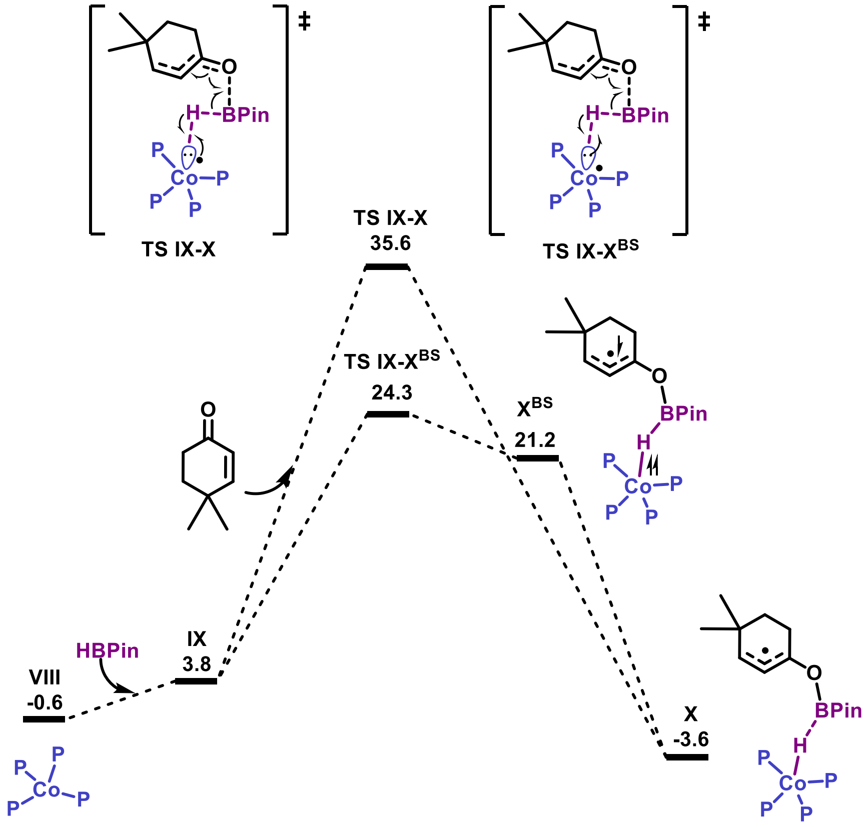


**Figure S7. Hydrogen atom abstraction of HBPin by Co(0) – broken symmetry vs standard pathway. Energies in kcal/mol. P = (P(Ph)(OMe)$_2$**

**Optimized cartesian coordinates and energies of all calculated compounds.**

*$G_{Corr}$ stands for the thermochemistry correction calculated at low level and E stands for single point energy calculated at high level.*

## HBPin

E = -412.0613643
$G_{Corr}$= 0.159625

| | | | |
|---|---|---|---|
| C | 0.00000000 | 0.78782700 | -0.19232100 |
| C | 0.00000000 | -0.78782700 | -0.19232100 |
| O | 0.32085400 | 1.10147600 | 1.19963800 |
| O | -0.32085400 | -1.10147600 | 1.19963800 |
| C | 1.05109500 | 1.43415000 | -1.08959000 |
| H | 0.88428200 | 1.15716600 | -2.13635400 |
| H | 0.98320700 | 2.52368000 | -1.00954300 |
| H | 2.06237200 | 1.13589200 | -0.80495700 |
| C | -1.38026100 | 1.39628500 | -0.46564300 |
| H | -1.34331700 | 2.46924800 | -0.25436400 |
| H | -1.67865700 | 1.25734500 | -1.50955700 |
| H | -2.14316700 | 0.94913600 | 0.17926800 |
| C | -1.05109500 | -1.43415000 | -1.08959000 |
| H | -0.88428200 | -1.15716600 | -2.13635400 |
| H | -0.98320700 | -2.52368000 | -1.00954300 |
| H | -2.06237200 | -1.13589200 | -0.80495700 |
| C | 1.38026100 | -1.39628500 | -0.46564300 |
| H | 1.34331700 | -2.46924800 | -0.25436400 |
| H | 1.67865700 | -1.25734500 | -1.50955700 |
| H | 2.14316700 | -0.94913600 | 0.17926800 |
| B | 0.00000000 | 0.00000000 | 1.94269700 |
| H | 0.00000000 | 0.00000000 | 3.13398600 |

## Cyclohexenone

E = -387.4700977
$G_{Corr}$= 0.151558

| | | | |
|---|---|---|---|
| C | -2.40112800 | 0.29066000 | -0.93402400 |
| H | -3.03332800 | 1.09095000 | -0.53093900 |
| H | -2.07965700 | 0.58407700 | -1.93996400 |
| H | -3.01566800 | -0.61303700 | -1.02332200 |
| C | -1.18994800 | 0.02313600 | -0.01762600 |
| C | -0.34688500 | -1.13308600 | -0.60399100 |
| C | -0.34459000 | 1.27545900 | 0.07096100 |
| C | 1.04336500 | -1.24986400 | 0.03489000 |
| H | -0.22179600 | -0.95695000 | -1.68071600 |
| H | -0.89459100 | -2.07755500 | -0.49975900 |
| C | 0.99950700 | 1.29717200 | 0.05129300 |
| H | -0.89018400 | 2.21443400 | 0.17191300 |
| C | 1.81078500 | 0.06436200 | -0.01356000 |
| H | 1.65009100 | -2.02267500 | -0.44710300 |
| H | 0.95790000 | -1.53184800 | 1.09423600 |
| H | 1.55579600 | 2.22957600 | 0.10800000 |
| C | -1.70315100 | -0.32047000 | 1.40213000 |
| H | -2.27474500 | 0.51498000 | 1.82264300 |
| H | -2.36238600 | -1.19597700 | 1.36261400 |
| H | -0.87926600 | -0.53934100 | 2.08896100 |
| O | 3.03501300 | 0.10239400 | -0.05837500 |

## $H_2$

E = -1.180095054
$G_{Corr}$= -0.001349

| | | | |
|---|---|---|---|
| H | 0.00000000 | 0.00000000 | 0.37146800 |
| H | 0.00000000 | 0.00000000 | -0.37146800 |

## I

E = -3794.102427
$G_{Corr}$= 0.470988

| | | | |
|---|---|---|---|
| Co | 0.18635900 | -0.16768100 | -0.74349300 |
| H | 0.70447800 | -1.21389800 | -1.72805400 |
| P | -0.54008300 | 1.44765800 | 0.45136300 |
| O | -0.28716800 | 1.26125200 | 2.07026300 |
| O | -2.16813800 | 1.79992000 | 0.54245200 |
| C | 0.09109900 | 3.13517300 | 0.04664200 |
| C | -0.76851600 | 2.19328600 | 3.04937700 |
| C | -2.88553400 | 2.04385000 | -0.66794000 |
| C | 1.28041000 | 3.27241200 | -0.68577200 |
| C | -0.60785100 | 4.29433800 | 0.42927800 |
| H | -0.49840400 | 1.78090400 | 4.02417200 |
| H | -0.29146100 | 3.17244500 | 2.92630600 |
| H | -1.85470300 | 2.30285200 | 2.98316000 |
| H | -3.94720900 | 2.04130900 | -0.41126400 |
| H | -2.61351400 | 3.01723600 | -1.09607900 |
| H | -2.70216900 | 1.25938600 | -1.41052700 |
| C | 1.77245200 | 4.53842100 | -1.01044700 |
| H | 1.82142400 | 2.38747800 | -1.00185000 |
| C | -0.11380300 | 5.55893400 | 0.10850400 |
| H | -1.54651200 | 4.19844900 | 0.96617500 |
| C | 1.07963000 | 5.68295400 | -0.60972300 |
| H | 2.69532100 | 4.62875300 | -1.57693400 |
| H | -0.66156200 | 6.44709600 | 0.41240400 |
| H | 1.46266000 | 6.66823800 | -0.86180000 |
| P | -1.56353600 | -1.30990600 | -1.05674000 |
| O | -1.19758000 | -2.92723700 | -1.13400600 |
| O | -2.45373000 | -1.07170800 | -2.43919100 |
| C | -2.94720300 | -1.29300300 | 0.14770800 |
| C | -2.23359000 | -3.90963000 | -1.27572700 |
| C | -1.73224800 | -0.62415200 | -3.59700800 |
| C | -2.59717500 | -1.29437900 | 1.50647000 |
| C | -4.30073600 | -1.26596000 | -0.21395400 |
| H | -1.73256700 | -4.86912900 | -1.42247500 |
| H | -2.85960500 | -3.95696000 | -0.37748800 |
| H | -2.86394200 | -3.68957500 | -2.14420700 |
| H | -1.15214600 | -1.44363500 | -4.03601200 |
| H | -2.47505400 | -0.27377800 | -4.31782000 |
| H | -1.04884900 | 0.19294000 | -3.33476200 |
| C | -3.58536400 | -1.27482400 | 2.49016900 |
| H | -1.54737400 | -1.27518700 | 1.78922900 |
| C | -5.28967000 | -1.23874700 | 0.77245500 |
| H | -4.57519400 | -1.25573900 | -1.26376200 |
| C | -4.93425100 | -1.24547500 | 2.12389300 |
| H | -3.30415700 | -1.27053000 | 3.53984100 |
| H | -6.33776000 | -1.21229400 | 0.48551300 |
| H | -5.70566700 | -1.22212600 | 2.88914100 |
| P | 2.28425800 | -0.26612600 | -0.43172900 |
| O | 3.24449200 | 0.80731200 | 0.42237900 |
| O | 3.16723100 | -0.20475700 | -1.82474900 |
| C | 2.78263200 | -1.83911200 | 0.39658000 |
| C | 2.87221800 | 1.19095900 | 1.74883600 |
| C | 4.59918000 | -0.30209200 | -1.81870100 |
| C | 1.96823000 | -2.97743500 | 0.27416000 |
| C | 3.95636200 | -1.92020800 | 1.16545500 |
| H | 2.23665600 | 2.08042500 | 1.72365600 |
| H | 2.33651200 | 0.39382100 | 2.27506200 |
| H | 3.79660200 | 1.41737400 | 2.28853600 |
| H | 4.91187200 | -0.28103700 | -2.86506700 |
| H | 5.04830000 | 0.53812200 | -1.28096900 |
| H | 4.92756200 | -1.24338900 | -1.36254700 |
| C | 2.32662000 | -4.17093300 | 0.90384600 |

| | | | |
|---|---|---|---|
| H | 1.05285800 | -2.92304000 | -0.30594200 |
| C | 4.31333300 | -3.11609300 | 1.79075900 |
| H | 4.58611800 | -1.04310100 | 1.27488000 |
| C | 3.49862000 | -4.24404300 | 1.66129900 |
| H | 1.68675400 | -5.04387400 | 0.80388900 |
| H | 5.22505300 | -3.16636200 | 2.38044500 |
| H | 3.77447600 | -5.17389300 | 2.1518860 |

## II

E = -4181.600021

$G_{Corr}$= 0.646686

| | | | |
|---|---|---|---|
| Co | 0.26314400 | -0.17622200 | 0.13502200 |
| H | 1.38899600 | -0.28170800 | 1.12789400 |
| P | -1.37872200 | -0.04139500 | -1.30187900 |
| O | -1.71336300 | -1.40083400 | -2.18916300 |
| O | -1.14866400 | 1.12288100 | -2.45409000 |
| C | -3.09876200 | 0.26679500 | -0.74972700 |
| C | -0.61315100 | -2.18011300 | -2.68612900 |
| C | -2.04813100 | 1.25642300 | -3.56351300 |
| C | -3.51033100 | 1.58377500 | -0.49600100 |
| C | -3.97726500 | -0.79061400 | -0.47460600 |
| H | -0.94413400 | -3.22036200 | -2.71710100 |
| H | -0.34409600 | -1.84131900 | -3.69358200 |
| H | 0.25620200 | -2.09351200 | -2.02954800 |
| H | -1.64125100 | 2.04584400 | -4.19971900 |
| H | -2.11135700 | 0.32142900 | -4.12996700 |
| H | -3.05100900 | 1.54145600 | -3.22639300 |
| C | -4.77219500 | 1.83742100 | 0.04318000 |
| H | -2.84971600 | 2.41069100 | -0.72662900 |
| C | -5.23980800 | -0.53502300 | 0.06321800 |
| H | -3.67057000 | -1.80808100 | -0.68466900 |
| C | -5.63739300 | 0.77840600 | 0.33086600 |
| H | -5.07426300 | 2.86313200 | 0.23709700 |
| H | -5.91313600 | -1.36183000 | 0.27431600 |
| H | -6.61860200 | 0.97495700 | 0.75507600 |
| P | 0.51787900 | 1.78648100 | 0.85123200 |
| O | 2.04117200 | 2.40528200 | 0.51058400 |
| O | 0.41413900 | 2.04444300 | 2.49363000 |
| C | -0.54676700 | 3.22032200 | 0.39943900 |
| C | 2.42761600 | 3.69464700 | 1.00652700 |
| C | 0.98476400 | 1.10271300 | 3.40597200 |
| C | -0.46987100 | 3.69957700 | -0.91846200 |
| C | -1.49493900 | 3.77356900 | 1.27175600 |
| H | 3.50131700 | 3.79516800 | 0.82813300 |
| H | 1.89583700 | 4.49442300 | 0.47830300 |
| H | 2.22683400 | 3.77891900 | 2.07970400 |
| H | 2.07451700 | 1.05388700 | 3.28905100 |
| H | 0.74643000 | 1.46479300 | 4.41012300 |
| H | 0.57273000 | 0.10107700 | 3.25835400 |
| C | -1.32673300 | 4.70986000 | -1.35557400 |
| H | 0.24592400 | 3.26187300 | -1.60739500 |
| C | -2.35713000 | 4.78127800 | 0.83159900 |
| H | -1.55692000 | 3.41108100 | 2.29207300 |
| C | -2.27794000 | 5.24901600 | -0.48250500 |
| H | -1.25935900 | 5.07108300 | -2.37848700 |
| H | -3.09228200 | 5.19891700 | 1.51485100 |
| H | -2.95268200 | 6.02876400 | -0.82591600 |
| P | -0.45600500 | -1.85196700 | 1.19060200 |
| O | -1.98599300 | -1.77808400 | 1.85199100 |
| O | 0.47746700 | -2.27480000 | 2.50863000 |
| C | -0.68082400 | -3.49862200 | 0.38939400 |
| C | -2.38188800 | -0.56155800 | 2.49617400 |
| C | 0.04573900 | -3.32276500 | 3.38639700 |
| C | 0.47647700 | -4.17066500 | -0.03673000 |
| C | -1.93320200 | -4.03887600 | 0.06855800 |
| H | -1.93901000 | 0.30649200 | 1.99600800 |
| H | -3.47051200 | -0.50225600 | 2.43309400 |
| H | -2.06863400 | -0.57100600 | 3.54771100 |
| H | 0.73549300 | -3.32506400 | 4.23411500 |
| H | -0.97395700 | -3.14322700 | 3.74387500 |
| H | 0.08194000 | -4.29741600 | 2.88539000 |
| C | 0.38526100 | -5.35481800 | -0.76735300 |
| H | 1.45515700 | -3.75916300 | 0.19965900 |
| C | -2.02667800 | -5.21804000 | -0.67561200 |
| H | -2.83178500 | -3.53721100 | 0.40934700 |
| C | -0.86972400 | -5.87762800 | -1.09740200 |
| H | 1.28990300 | -5.86655500 | -1.08555600 |
| H | -3.00473100 | -5.62324200 | -0.92268200 |
| H | -0.94371800 | -6.79369300 | -1.67742000 |
| C | 7.18760900 | -0.39932100 | -0.37957200 |
| H | 7.52197500 | -0.66141200 | 0.63152700 |
| H | 7.28066900 | -1.29128200 | -1.01033800 |
| H | 7.86732300 | 0.36630200 | -0.77194000 |
| C | 5.73986100 | 0.12467800 | -0.36486700 |
| C | 5.30492800 | 0.48498500 | -1.80480500 |
| C | 4.80920400 | -0.93869300 | 0.17720700 |
| C | 3.80984500 | 0.80336600 | -1.91663400 |
| H | 5.53752900 | -0.36843900 | -2.45566900 |
| H | 5.89935400 | 1.33268300 | -2.16742300 |
| C | 3.52552100 | -1.07531500 | -0.20644600 |
| H | 5.20393900 | -1.58712500 | 0.95950000 |
| C | 2.92464300 | -0.19857000 | -1.20882900 |
| H | 3.48618600 | 0.88152500 | -2.96045300 |
| H | 3.56905200 | 1.76604300 | -1.44490000 |
| H | 2.85762400 | -1.79762700 | 0.25167800 |
| C | 5.64873800 | 1.35705400 | 0.56971400 |
| H | 5.97237000 | 1.09754900 | 1.58449900 |
| H | 6.29823700 | 2.16142100 | 0.20198000 |
| H | 4.62194300 | 1.72917900 | 0.63214700 |
| O | 1.70614300 | -0.21208000 | -1.44858600 |

## III

E = -4181.596399

$G_{Corr}$= 0.654547

| | | | |
|---|---|---|---|
| Co | 0.23967900 | -0.64293900 | -0.01756500 |
| H | 0.65486100 | -1.63261400 | -1.04111200 |
| P | -0.60260900 | 0.98961900 | 1.21148900 |
| O | 0.30003000 | 2.01556600 | 2.15420000 |
| O | -1.67398400 | 0.37233900 | 2.30980900 |
| C | -1.50564600 | 2.34363900 | 0.34499100 |
| C | 0.82095500 | 1.61037700 | 3.43245600 |
| C | -2.63225300 | 1.15698100 | 3.02946700 |
| C | -1.87162500 | 2.18403900 | -0.99701900 |
| C | -1.83565400 | 3.54492500 | 0.99722800 |
| H | 1.34611800 | 2.48252000 | 3.82951500 |
| H | 0.00726500 | 1.33935600 | 4.11407100 |
| H | 1.51714000 | 0.77194800 | 3.35331600 |
| H | -3.13003300 | 0.47468600 | 3.72160700 |
| H | -2.15147500 | 1.96311200 | 3.59452000 |
| H | -3.37515800 | 1.58122900 | 2.34714900 |
| C | -2.57708100 | 3.18381800 | -1.66877600 |
| H | -1.57288700 | 1.29122300 | -1.52998600 |
| C | -2.53803300 | 4.54598500 | 0.32684600 |
| H | -1.52061000 | 3.70237100 | 2.02279100 |
| C | -2.91721000 | 4.36458200 | -1.00714500 |
| H | -2.85063400 | 3.04033800 | -2.71070700 |
| H | -2.78577500 | 5.46959100 | 0.84313200 |
| H | -3.46364000 | 5.14578400 | -1.52889100 |
| P | -1.62789300 | -1.29441900 | -0.87411200 |
| O | -1.74517800 | -2.94802100 | -0.89835800 |
| O | -1.95818900 | -0.91193100 | -2.45815600 |
| C | -3.29983300 | -0.86971900 | -0.22496500 |
| C | -2.83964200 | -3.60693500 | -1.54708100 |
| C | -0.89531400 | -0.88153000 | -3.42352500 |
| C | -3.65298600 | -1.40530700 | 1.02377600 |
| C | -4.21287100 | -0.04520600 | -0.89423300 |

| | | | |
|---|---|---|---|
| H | -2.58236400 | -4.66852300 | -1.58233900 |
| H | -3.76910300 | -3.47320300 | -0.98210300 |
| H | -2.97908500 | -3.22850600 | -2.56479700 |
| H | -0.37517000 | 0.07873300 | -3.38713700 |
| H | -0.16953200 | -1.68204200 | -3.25351300 |
| H | -1.36566600 | -1.01539000 | -4.40148400 |
| C | -4.89314500 | -1.12009300 | 1.59307000 |
| H | -2.94877400 | -2.03816400 | 1.55594900 |
| C | -5.45062000 | 0.25252700 | -0.31766100 |
| H | -3.95597200 | 0.35958700 | -1.86605000 |
| C | -5.79317800 | -0.28297900 | 0.92545300 |
| H | -5.15550900 | -1.54422200 | 2.55870900 |
| H | -6.14761700 | 0.89970600 | -0.84366900 |
| H | -6.75675200 | -0.05244100 | 1.37222000 |
| P | 1.66125600 | 0.46833200 | -1.23413600 |
| O | 1.07523300 | 1.64839100 | -2.28017100 |
| O | 2.29858000 | -0.52774500 | -2.39402200 |
| C | 3.12063600 | 1.36902900 | -0.53994400 |
| C | 1.04443000 | 3.03479000 | -1.92391700 |
| C | 3.10912700 | -0.10280800 | -3.49267700 |
| C | 3.25763200 | 1.34682700 | 0.85089700 |
| C | 4.07416200 | 2.07439800 | -1.29948300 |
| H | 0.85745400 | 3.18640900 | -0.85594600 |
| H | 1.98819000 | 3.52291300 | -2.18888900 |
| H | 0.22347500 | 3.48856500 | -2.48270600 |
| H | 3.05081900 | -0.89546100 | -4.24337400 |
| H | 2.73607400 | 0.83117900 | -3.92417900 |
| H | 4.15276100 | 0.01903800 | -3.18243100 |
| C | 4.33344300 | 1.97345000 | 1.48293300 |
| H | 2.50872800 | 0.83518900 | 1.43342700 |
| C | 5.14859300 | 2.70482100 | -0.67262200 |
| H | 3.97329100 | 2.15390900 | -2.37594100 |
| C | 5.28522400 | 2.64966600 | 0.71965300 |
| H | 4.42211900 | 1.92228300 | 2.56419600 |
| H | 5.87961800 | 3.24414000 | -1.26941400 |
| H | 6.12750400 | 3.13857900 | 1.20204400 |
| C | 0.94987500 | -4.31823800 | -0.90319900 |
| H | -0.01132100 | -4.80071100 | -0.70297500 |
| H | 0.78760400 | -3.57367600 | -1.68685100 |
| H | 1.65009900 | -5.07641300 | -1.27639100 |
| C | 1.52041800 | -3.66518400 | 0.36476600 |
| C | 2.87108700 | -3.00125400 | 0.03140300 |
| C | 0.57469600 | -2.63761700 | 0.99019000 |
| C | 3.48866000 | -2.24995600 | 1.21710000 |
| H | 2.70542300 | -2.30374900 | -0.79266400 |
| H | 3.57283700 | -3.76594600 | -0.32694700 |
| C | 1.06489900 | -1.65084900 | 1.84490700 |
| H | -0.43929800 | -2.99321300 | 1.14267100 |
| C | 2.49821900 | -1.51076100 | 2.10593200 |
| H | 4.22831900 | -1.51505000 | 0.87710800 |
| H | 4.03148800 | -2.93327500 | 1.88365100 |
| H | 0.41603300 | -1.24239400 | 2.61344100 |
| C | 1.70616300 | -4.77772500 | 1.43062600 |
| H | 0.74551300 | -5.24907500 | 1.67138600 |
| H | 2.37900100 | -5.55712400 | 1.05181200 |
| H | 2.12633500 | -4.38521700 | 2.36251500 |
| O | 2.92437100 | -0.86006900 | 3.06847800 |

## TS III-IV

E = -4181.591504

$G_{Corr}$= 0.655203

| | | | |
|---|---|---|---|
| Co | 0.31919700 | 0.44072200 | -0.01791500 |
| H | 0.95408900 | 1.60833400 | 0.70974600 |
| P | -0.34054700 | -1.28705100 | -1.15969300 |
| O | 0.92923800 | -2.25815500 | -1.58152500 |
| O | -1.03031500 | -1.18803600 | -2.65481500 |
| C | -1.48208800 | -2.47602300 | -0.32955200 |
| C | 0.75700600 | -3.47388900 | -2.32375300 |
| C | -2.22121500 | -0.39584300 | -2.84856300 |
| C | -1.38488600 | -2.65008900 | 1.05875500 |
| C | -2.43896100 | -3.21380800 | -1.04301700 |
| H | 1.75818000 | -3.88616400 | -2.46766100 |
| H | 0.14169000 | -4.19048800 | -1.76730400 |
| H | 0.29448700 | -3.27395400 | -3.29432000 |
| H | -1.98729600 | 0.66671700 | -2.77734600 |
| H | -2.57465300 | -0.62982600 | -3.85624400 |
| H | -2.99783300 | -0.66442000 | -2.12565500 |
| C | -2.22936200 | -3.54017900 | 1.72291500 |
| H | -0.65651800 | -2.07604000 | 1.61752600 |
| C | -3.29508700 | -4.09262600 | -0.37699900 |
| H | -2.51659900 | -3.09267500 | -2.11865300 |
| C | -3.19293200 | -4.25623300 | 1.00736400 |
| H | -2.14415900 | -3.66261200 | 2.79933200 |
| H | -4.04113600 | -4.64936100 | -0.93781000 |
| H | -3.86130700 | -4.93884000 | 1.52542200 |
| P | -1.44566300 | 0.94172200 | 1.13008400 |
| O | -1.51075900 | 2.52152400 | 1.66396000 |
| O | -1.77175500 | 0.12719500 | 2.55270300 |
| C | -3.13154000 | 0.73085000 | 0.41749200 |
| C | -2.59300200 | 2.99014400 | 2.48128100 |
| C | -0.72314400 | 0.02776800 | 3.52021400 |
| C | -3.57703800 | 1.67616700 | -0.52113600 |
| C | -3.93431300 | -0.38628800 | 0.69058300 |
| H | -2.31819300 | 3.99455700 | 2.81242800 |
| H | -3.52570900 | 3.03501900 | 1.90876400 |
| H | -2.73610000 | 2.34070800 | 3.35063400 |
| H | -1.12925100 | -0.53728500 | 4.36331400 |
| H | 0.14996300 | -0.49209100 | 3.11554100 |
| H | -0.41140100 | 1.02077400 | 3.86684900 |
| C | -4.79678800 | 1.50531400 | -1.17551200 |
| H | -2.96425100 | 2.53704400 | -0.76394400 |
| C | -5.14912500 | -0.56268500 | 0.02403800 |
| H | -3.60670100 | -1.12043300 | 1.41620300 |
| C | -5.58330800 | 0.38012300 | -0.91078200 |
| H | -5.12569900 | 2.24477400 | -1.90068300 |
| H | -5.75289500 | -1.44125900 | 0.23608300 |
| H | -6.52755500 | 0.23982300 | -1.43041800 |
| P | 1.96978100 | -0.60238400 | 0.92619100 |
| O | 1.81493100 | -2.01939600 | 1.79859900 |
| O | 2.52407800 | 0.34009800 | 2.16589800 |
| C | 3.49001000 | -0.98029800 | -0.06729200 |
| C | 1.90652200 | -3.33931900 | 1.25169100 |
| C | 3.56154200 | -0.02478000 | 3.08228300 |
| C | 3.48643600 | -0.64410000 | -1.42866500 |
| C | 4.63467600 | -1.59551000 | 0.46974800 |
| H | 1.00810400 | -3.59511900 | 0.68603700 |
| H | 2.77955200 | -3.44677000 | 0.60119900 |
| H | 2.00337300 | -4.01669300 | 2.10442200 |
| H | 3.46074600 | 0.64338500 | 3.94108400 |
| H | 3.45137200 | -1.06158800 | 3.41469100 |
| H | 4.54881400 | 0.11910000 | 2.62977000 |
| C | 4.60782900 | -0.87460800 | -2.22719500 |
| H | 2.58274500 | -0.23117800 | -1.86301000 |
| C | 5.75677100 | -1.82978900 | -0.32701000 |
| H | 4.64488600 | -1.92165300 | 1.50425000 |
| C | 5.74904400 | -1.46058900 | -1.67515400 |
| H | 4.58586600 | -0.60569300 | -3.27983400 |
| H | 6.63358900 | -2.30721400 | 0.10239400 |
| H | 6.62350700 | -1.64226500 | -2.29421100 |
| C | 3.29073200 | 3.96666500 | -0.53403700 |
| H | 3.99944400 | 3.31715600 | -0.00548100 |
| H | 3.46671900 | 3.85904400 | -1.61123900 |
| H | 3.51032500 | 5.00498900 | -0.25733100 |
| C | 1.83361400 | 3.61018300 | -0.16595000 |
| C | 0.88500400 | 4.61964500 | -0.85454800 |
| C | 1.58064300 | 2.18469700 | -0.73385900 |
| C | -0.53766400 | 4.07139500 | -1.01365500 |
| H | 1.27993300 | 4.86196200 | -1.85106400 |
| H | 0.87549200 | 5.55710800 | -0.28448300 |
| C | 0.57522000 | 1.89828600 | -1.69252300 |

| | | | |
|---|---|---|---|
| H | 2.51078900 | 1.63824300 | -0.84618500 |
| C | -0.52482500 | 2.83930800 | -1.90774200 |
| H | -1.20816600 | 4.80677100 | -1.46967200 |
| H | -0.94606000 | 3.79748100 | -0.03562900 |
| H | 0.75583800 | 1.18279500 | -2.49071800 |
| C | 1.68661100 | 3.69468000 | 1.36550600 |
| H | 2.30944700 | 2.93853700 | 1.85501400 |
| H | 2.00058800 | 4.68647100 | 1.71514800 |
| H | 0.65196600 | 3.52456100 | 1.67449800 |
| O | -1.37544300 | 2.69965100 | -2.79432500 |

# IV

E = -4181.599567

$G_{Corr}$= 0.65966

| | | | |
|---|---|---|---|
| A Co | 0.32862700 | 0.41135600 | -0.20373900 |
| H | 1.89703700 | 1.34163600 | -0.46623100 |
| P | -0.14321800 | -1.30366600 | -1.41110800 |
| O | 1.11474400 | -2.37291200 | -1.58059200 |
| O | -0.52330400 | -1.19070100 | -3.03186400 |
| C | -1.52029100 | -2.40200000 | -0.83627500 |
| C | 1.01938000 | -3.61074700 | -2.29593800 |
| C | -1.73210800 | -0.58567900 | -3.51744000 |
| C | -1.89638600 | -2.35447900 | 0.51258900 |
| C | -2.20655100 | -3.27420700 | -1.69714700 |
| H | 2.02938100 | -4.02666700 | -2.32545500 |
| H | 0.35168300 | -4.31119400 | -1.78081000 |
| H | 0.65874000 | -3.44692000 | -3.31581200 |
| H | -1.76207900 | 0.48887800 | -3.31146700 |
| H | -1.73993600 | -0.75080200 | -4.59796400 |
| H | -2.61723600 | -1.05973900 | -3.07824400 |
| C | -2.93486300 | -3.14888700 | 0.99675600 |
| H | -1.36548800 | -1.68466500 | 1.17768400 |
| C | -3.25254200 | -4.06574900 | -1.21825700 |
| H | -1.92719000 | -3.32698800 | -2.74489700 |
| C | -3.62093500 | -4.00183900 | 0.12846500 |
| H | -3.21621100 | -3.09145500 | 2.04478600 |
| H | -3.78131100 | -4.73019900 | -1.89657000 |
| H | -4.44001900 | -4.61294200 | 0.49799800 |
| P | -1.10727600 | 1.30075900 | 1.18398700 |
| O | -1.17212400 | 2.95318700 | 1.24963700 |
| O | -1.09282400 | 1.08584900 | 2.84476500 |
| C | -2.87193700 | 0.85666300 | 0.83882000 |
| C | -2.14041000 | 3.67473000 | 2.02161900 |
| C | -1.15582600 | -0.18981400 | 3.47751400 |
| C | -3.23958200 | 0.57794900 | -0.48355500 |
| C | -3.84722400 | 0.77110200 | 1.84540700 |
| H | -1.92243400 | 4.73499700 | 1.87122000 |
| H | -3.15725800 | 3.46051800 | 1.67493400 |
| H | -2.05569500 | 3.42601400 | 3.08353000 |
| H | -0.29415500 | -0.81268900 | 3.23236000 |
| H | -1.16315700 | -0.00031900 | 4.55479800 |
| H | -2.07612200 | -0.72599800 | 3.21193700 |
| C | -4.54472200 | 0.20082700 | -0.79697500 |
| H | -2.49618600 | 0.68183400 | -1.26189900 |
| C | -5.15180100 | 0.37903700 | 1.53643300 |
| H | -3.58607600 | 1.01226700 | 2.87094600 |
| C | -5.50115100 | 0.08601900 | 0.21535700 |
| H | -4.81269200 | -0.00828600 | -1.82937200 |
| H | -5.89515800 | 0.30488500 | 2.32614800 |
| H | -6.51538000 | -0.22312400 | -0.02374200 |
| P | 1.63911900 | -0.65059500 | 1.18679200 |
| O | 1.24981300 | -2.01859300 | 2.05019000 |
| O | 1.94170100 | 0.34113700 | 2.45885300 |
| C | 3.30490000 | -1.12770700 | 0.55238600 |
| C | 1.27270300 | -3.34385800 | 1.50252000 |
| C | 2.74207300 | 0.01519000 | 3.60307900 |
| C | 3.65985000 | -0.83104600 | -0.77077100 |
| C | 4.23178000 | -1.80418400 | 1.36574800 |
| H | 0.42523900 | -3.50673500 | 0.83377200 |
| H | 2.20272300 | -3.53561700 | 0.95916300 |
| H | 1.20061600 | -4.02433800 | 2.35454300 |
| H | 2.51116100 | 0.76841200 | 4.35934300 |
| H | 2.49205000 | -0.97791800 | 3.98829500 |
| H | 3.80818800 | 0.06256600 | 3.35675100 |
| C | 4.92663000 | -1.15594400 | -1.25835200 |
| H | 2.92765100 | -0.37696800 | -1.42493500 |
| C | 5.49756500 | -2.13173300 | 0.87841400 |
| H | 3.95700600 | -2.10114700 | 2.37239900 |
| C | 5.85133800 | -1.79825500 | -0.43206000 |
| H | 5.18531700 | -0.91739000 | -2.28638300 |
| H | 6.20417700 | -2.65269800 | 1.51881200 |
| H | 6.83747800 | -2.05285400 | -0.81093200 |
| C | 3.55147800 | 3.57275200 | -1.43500500 |
| H | 4.18347100 | 2.80978000 | -0.96249400 |
| H | 3.65522200 | 3.47084300 | -2.52260400 |
| H | 3.94138500 | 4.55753400 | -1.14898200 |
| C | 2.08448200 | 3.41385900 | -0.99454700 |
| C | 1.20657300 | 4.43976500 | -1.74024600 |
| C | 1.61653000 | 1.97421300 | -1.37421700 |
| C | -0.29156300 | 4.24539500 | -1.46889100 |
| H | 1.39011600 | 4.34578000 | -2.82074000 |
| H | 1.51160800 | 5.45649500 | -1.45853800 |
| C | 0.17035200 | 1.72748700 | -1.75328100 |
| H | 2.26279100 | 1.57221200 | -2.16124500 |
| C | -0.76832300 | 2.85632700 | -1.87309300 |
| H | -0.89864900 | 4.97351200 | -2.01734800 |
| H | -0.50689600 | 4.37445900 | -0.40426200 |
| H | 0.08515700 | 1.12590100 | -2.65506700 |
| C | 2.00992400 | 3.63122100 | 0.52817200 |
| H | 2.68940000 | 2.94208700 | 1.04439600 |
| H | 2.30639100 | 4.65700400 | 0.78302900 |
| H | 1.00840400 | 3.45135300 | 0.92051900 |
| O | -1.89443500 | 2.70905600 | -2.36210000 |

# TS IV-V

E = -4181.588622

$G_{Corr}$= 0.656733

| | | | |
|---|---|---|---|
| Co | 0.06178500 | 0.20560900 | 0.15499200 |
| H | -0.65940800 | 3.19944000 | -0.63503100 |
| P | 1.17579500 | -1.40454500 | -0.93405700 |
| O | 2.48222200 | -1.90110000 | -0.04540600 |
| O | 1.99639700 | -1.22534200 | -2.35552000 |
| C | 0.25796400 | -2.97895800 | -1.14452800 |
| C | 3.42082700 | -2.89397200 | -0.46417300 |
| C | 1.42585200 | -1.10756700 | -3.66550300 |
| C | -0.85488300 | -3.22060000 | -0.32854500 |
| C | 0.67069500 | -3.96764300 | -2.04921300 |
| H | 4.23809100 | -2.86757100 | 0.26086400 |
| H | 2.96115900 | -3.88968700 | -0.46527600 |
| H | 3.81004100 | -2.66972700 | -1.46137800 |
| H | 0.59526600 | -1.80146500 | -3.80689900 |
| H | 1.07080900 | -0.08976400 | -3.84011900 |
| H | 2.23559600 | -1.34439100 | -4.36210400 |
| C | -1.55358600 | -4.42419200 | -0.41910600 |
| H | -1.18172000 | -2.45027200 | 0.36250300 |
| C | -0.02743000 | -5.17463100 | -2.13936000 |
| H | 1.53343400 | -3.79396200 | -2.68506300 |
| C | -1.14071700 | -5.40405100 | -1.32677600 |
| H | -2.42436500 | -4.59026500 | 0.20956100 |
| H | 0.29668100 | -5.93364200 | -2.84648300 |
| H | -1.68520800 | -6.34149500 | -1.40245100 |
| P | -1.67731800 | 0.93050200 | 1.29310500 |
| O | -2.17401700 | 2.46428000 | 0.95657800 |
| O | -1.75114800 | 1.07236300 | 2.94532300 |
| C | -3.17465500 | -0.11958000 | 1.01721900 |
| C | -3.35218000 | 3.05929400 | 1.52053200 |
| C | -1.44453100 | -0.03449300 | 3.79436100 |

| | | | |
|---|---|---|---|
| C | -3.29945100 | -0.90421800 | -0.14040400 |
| C | -4.20879700 | -0.14650400 | 1.97171000 |
| H | -3.39687900 | 4.07710800 | 1.12675200 |
| H | -4.25011100 | 2.51027500 | 1.21606300 |
| H | -3.28965200 | 3.08607400 | 2.61217000 |
| H | -1.89914100 | -0.96401900 | 3.42996800 |
| H | -0.36410700 | -0.16362800 | 3.88659600 |
| H | -1.86529000 | 0.20009300 | 4.77580800 |
| C | -4.43387200 | -1.69545600 | -0.33660400 |
| H | -2.51156300 | -0.91404600 | -0.88758200 |
| C | -5.34199000 | -0.93481100 | 1.76945600 |
| H | -4.12036900 | 0.44670200 | 2.87623400 |
| C | -5.45676500 | -1.71296100 | 0.61379600 |
| H | -4.51031700 | -2.30339000 | -1.23418700 |
| H | -6.13259900 | -0.94447400 | 2.51520800 |
| H | -6.33681200 | -2.33176400 | 0.45921100 |
| P | 1.45444600 | 0.29801300 | 1.72989700 |
| O | 1.80741200 | -0.87397100 | 2.84953900 |
| O | 1.12259200 | 1.57713700 | 2.71452300 |
| C | 3.14527700 | 0.71971900 | 1.15275600 |
| C | 1.19755500 | -2.16570100 | 2.76170500 |
| C | 1.98949300 | 1.91742700 | 3.80574900 |
| C | 3.27639000 | 1.78171000 | 0.24659200 |
| C | 4.28618500 | 0.02050300 | 1.56671600 |
| H | 0.26513600 | -2.11740900 | 2.18817800 |
| H | 1.87814300 | -2.86350600 | 2.26772400 |
| H | 0.98620800 | -2.50031200 | 3.78130700 |
| H | 1.56040100 | 2.80468100 | 4.27544800 |
| H | 2.03401400 | 1.09956000 | 4.53255900 |
| H | 3.00146200 | 2.14306900 | 3.45007700 |
| C | 4.53385300 | 2.13687400 | -0.24463500 |
| H | 2.39053800 | 2.31691400 | -0.07990900 |
| C | 5.54294700 | 0.37767000 | 1.07571800 |
| H | 4.18139300 | -0.80458500 | 2.26305000 |
| C | 5.66871800 | 1.43317800 | 0.16729300 |
| H | 4.62616900 | 2.95900200 | -0.94938000 |
| H | 6.42465600 | -0.16968900 | 1.39906600 |
| H | 6.64785400 | 1.70587300 | -0.21752700 |
| C | 0.33278900 | 3.83474000 | -3.86272200 |
| H | 1.07908300 | 4.57358600 | -3.54291000 |
| H | 0.85912700 | 2.90011800 | -4.09447800 |
| H | -0.12855800 | 4.20056000 | -4.78917900 |
| C | -0.72102700 | 3.60129400 | -2.76656700 |
| C | -1.82089200 | 2.63002300 | -3.30540700 |
| C | -0.03235000 | 2.98453300 | -1.50529200 |
| C | -2.18697600 | 1.51070100 | -2.30764000 |
| H | -1.46926200 | 2.15149300 | -4.22889000 |
| H | -2.72107100 | 3.19518600 | -3.57817400 |
| C | 0.19468700 | 1.48972700 | -1.61393300 |
| H | 0.91789400 | 3.50611500 | -1.33203800 |
| C | -0.91758400 | 0.71782500 | -2.00745100 |
| H | -2.94206800 | 0.83597000 | -2.72242700 |
| H | -2.59638200 | 1.95187700 | -1.39306200 |
| H | 1.18657500 | 1.15734100 | -1.90915600 |
| C | -1.35631500 | 4.94379000 | -2.36531000 |
| H | -0.61211100 | 5.61060400 | -1.91076800 |
| H | -1.78580800 | 5.45906400 | -3.23409400 |
| H | -2.15881100 | 4.79074900 | -1.63209200 |
| O | -0.91040000 | -0.56154400 | -2.18869200 |

# V

E = -4181.613207

$G_{Corr}$= 0.651753

| | | | |
|---|---|---|---|
| Co | -0.08067800 | 0.17680500 | -0.30127000 |
| H | -5.00592500 | -1.52203400 | 1.95511900 |
| P | 1.88863500 | 0.87199500 | -0.75151000 |
| O | 1.80889400 | 2.42659900 | -1.32193200 |
| O | 2.66401000 | 0.09706000 | -1.98362000 |
| C | 3.25615500 | 0.86993000 | 0.45575200 |
| C | 2.98012000 | 3.08709500 | -1.82489400 |
| C | 1.86825800 | -0.26740800 | -3.12216200 |
| C | 2.90360700 | 0.97585400 | 1.80987500 |
| C | 4.60339700 | 0.70516800 | 0.10078100 |
| H | 2.65342600 | 4.06066100 | -2.19570000 |
| H | 3.72118700 | 3.22812700 | -1.02926700 |
| H | 3.43134100 | 2.51359900 | -2.64097800 |
| H | 0.91532500 | -0.71012900 | -2.80561000 |
| H | 1.67023100 | 0.60903100 | -3.75003600 |
| H | 2.44206200 | -1.00678500 | -3.68291300 |
| C | 3.88742900 | 0.92030500 | 2.79842100 |
| H | 1.85890300 | 1.09351200 | 2.08300500 |
| C | 5.58409900 | 0.64627100 | 1.09122200 |
| H | 4.87287400 | 0.58919500 | -0.94352800 |
| C | 5.22754200 | 0.75139800 | 2.43953200 |
| H | 3.60772400 | 0.99883700 | 3.84532000 |
| H | 6.62547200 | 0.50860100 | 0.81302800 |
| H | 5.99314500 | 0.69701200 | 3.20878700 |
| P | 0.55064600 | -1.87039400 | 0.20295600 |
| O | -0.52264200 | -3.06683000 | -0.16029400 |
| O | 0.87857800 | -2.06282600 | 1.81336600 |
| C | 2.12256800 | -2.61445800 | -0.39239900 |
| C | -0.38998400 | -4.36667300 | 0.43674300 |
| C | 0.13023000 | -1.35449100 | 2.81129400 |
| C | 2.12884800 | -3.26706800 | -1.63522500 |
| C | 3.32879300 | -2.45473700 | 0.30282600 |
| H | -1.15153600 | -4.99852000 | -0.02313300 |
| H | 0.60312200 | -4.79138000 | 0.25128600 |
| H | -0.56533300 | -4.31087600 | 1.51570800 |
| H | -0.74943600 | -1.93706700 | 3.10510900 |
| H | 0.79191000 | -1.23058300 | 3.67281400 |
| H | -0.19447800 | -0.37461500 | 2.44855400 |
| C | 3.32198500 | -3.74621800 | -2.17626700 |
| H | 1.19523300 | -3.40404500 | -2.17472900 |
| C | 4.52308100 | -2.92995200 | -0.24221500 |
| H | 3.33382700 | -1.95298700 | 1.26405700 |
| C | 4.52366000 | -3.57224800 | -1.48241500 |
| H | 3.31496900 | -4.25545300 | -3.13645100 |
| H | 5.45333400 | -2.79219100 | 0.30236700 |
| H | 5.45470000 | -3.93937800 | -1.90616800 |
| P | -0.80287000 | 2.02219900 | 0.64547700 |
| O | 0.12818500 | 3.03342500 | 1.58470200 |
| O | -1.78636200 | 1.52033400 | 1.85792400 |
| C | -1.79854500 | 3.22201400 | -0.32556300 |
| C | 1.06224000 | 3.99970700 | 1.09494700 |
| C | -2.53479600 | 2.42788200 | 2.67831100 |
| C | -2.32582400 | 2.78824000 | -1.55292200 |
| C | -2.03265100 | 4.53942900 | 0.10044900 |
| H | 2.05775900 | 3.55576000 | 1.01152700 |
| H | 0.76765700 | 4.40007900 | 0.12108600 |
| H | 1.08580000 | 4.81121100 | 1.82857000 |
| H | -3.06919600 | 1.81112400 | 3.40375600 |
| H | -1.86980900 | 3.12333600 | 3.20014800 |
| H | -3.25799600 | 2.99099000 | 2.07832700 |
| C | -3.08044300 | 3.66207500 | -2.33760400 |
| H | -2.15847200 | 1.75943600 | -1.85838000 |
| C | -2.78366000 | 5.41142000 | -0.68964000 |
| H | -1.62466300 | 4.88158900 | 1.04707900 |
| C | -3.30785600 | 4.97318300 | -1.90918400 |
| H | -3.49170600 | 3.31971800 | -3.28356400 |
| H | -2.95947500 | 6.43043200 | -0.35507600 |
| H | -3.89185600 | 5.65309700 | -2.52393100 |
| C | -6.31001500 | -3.41888800 | 0.73891800 |
| H | -6.19073100 | -4.08781900 | 1.60124200 |
| H | -6.97822500 | -2.60137400 | 1.03846100 |
| H | -6.80809200 | -3.98555900 | -0.05860000 |
| C | -4.94970500 | -2.88080500 | 0.26874400 |
| C | -5.15870900 | -1.87454100 | -0.88342600 |
| C | -4.24967000 | -2.13090500 | 1.42762400 |
| C | -3.83703500 | -1.29836600 | -1.40860500 |
| H | -5.78821300 | -1.05292100 | -0.51212000 |

| | | | |
|---|---|---|---|
| H | -5.71477900 | -2.35145200 | -1.70306100 |
| C | -3.08871400 | -1.27632700 | 0.97912800 |
| H | -3.90926700 | -2.87024500 | 2.17142300 |
| C | -2.87819400 | -0.90498000 | -0.30177700 |
| H | -4.02552400 | -0.41578100 | -2.03487100 |
| H | -3.32888300 | -2.02391100 | -2.06175300 |
| H | -2.38420100 | -0.93556600 | 1.72856900 |
| C | -4.07483600 | -4.05929900 | -0.20059700 |
| H | -3.99277700 | -4.81521100 | 0.59120900 |
| H | -4.50896700 | -4.54458400 | -1.08460300 |
| H | -3.06497500 | -3.72182000 | -0.44807300 |
| O | -1.85086400 | -0.18643800 | -0.75159300 |

## TS V-VI

E = -4593.6834

$G_{Corr}$= 0.83575

| | | | |
|---|---|---|---|
| Co | -0.33683500 | 0.35768200 | -0.02077100 |
| H | 3.67004700 | 0.37461500 | -3.93083900 |
| P | -2.14431800 | 0.87572900 | 1.03584000 |
| O | -1.94483300 | 2.28224000 | 1.87056800 |
| O | -2.65921100 | -0.02911900 | 2.31521100 |
| C | -3.69007200 | 1.12334500 | 0.08741000 |
| C | -2.98102700 | 2.81770600 | 2.70832400 |
| C | -2.72534400 | -1.46222900 | 2.27502200 |
| C | -3.57613000 | 1.63461300 | -1.21533200 |
| C | -4.94981500 | 0.75209100 | 0.57765000 |
| H | -2.61983000 | 3.78529900 | 3.06275600 |
| H | -3.90939100 | 2.95944900 | 2.14253300 |
| H | -3.16735400 | 2.15618000 | 3.55848400 |
| H | -1.79595300 | -1.89120400 | 1.89733500 |
| H | -2.88303900 | -1.78805200 | 3.30606000 |
| H | -3.56320800 | -1.79388800 | 1.65465000 |
| C | -4.70940000 | 1.77250100 | -2.01661200 |
| H | -2.59689700 | 1.90838200 | -1.59666500 |
| C | -6.08193100 | 0.88222900 | -0.22887700 |
| H | -5.03474400 | 0.34552200 | 1.58064600 |
| C | -5.96204800 | 1.38842000 | -1.52640800 |
| H | -4.61492700 | 2.16492600 | -3.02540000 |
| H | -7.05502900 | 0.58283000 | 0.15091600 |
| H | -6.84285500 | 1.48125400 | -2.15600400 |
| P | -1.13847000 | -1.25544100 | -1.28818200 |
| O | -0.01333900 | -2.36381100 | -1.75517000 |
| O | -1.74300800 | -0.67781100 | -2.72444500 |
| C | -2.56868500 | -2.35041900 | -0.92253400 |
| C | -0.23564900 | -3.23010200 | -2.87358300 |
| C | -1.02146500 | 0.34075900 | -3.43221000 |
| C | -2.32438200 | -3.54630600 | -0.22633100 |
| C | -3.88994600 | -1.98227300 | -1.21094500 |
| H | 0.63250600 | -3.89108500 | -2.92642400 |
| H | -1.14434100 | -3.82922500 | -2.74495300 |
| H | -0.31392900 | -2.65481200 | -3.80211900 |
| H | -0.40649700 | 0.94462100 | -2.75874100 |
| H | -0.37343200 | -0.12035900 | -4.18660100 |
| H | -1.76014900 | 0.97677200 | -3.92850200 |
| C | -3.38459800 | -4.35790000 | 0.17549200 |
| H | -1.30264800 | -3.82096100 | 0.02015200 |
| C | -4.95098200 | -2.79276100 | -0.79884600 |
| H | -4.09015600 | -1.06439000 | -1.75182400 |
| C | -4.70214800 | -3.97935800 | -0.10498400 |
| H | -3.18527100 | -5.28150800 | 0.71264100 |
| H | -5.97163600 | -2.49143500 | -1.01914300 |
| H | -5.52916600 | -4.60715600 | 0.21611500 |
| P | 0.27192500 | 2.45173800 | -0.33148400 |
| O | -0.72893500 | 3.67870500 | -0.85212000 |
| O | 1.24341600 | 2.46066800 | -1.64924400 |
| C | 1.25301800 | 3.27132100 | 0.98827300 |
| C | -1.63817300 | 4.44818200 | -0.06270300 |
| C | 1.98556700 | 3.60679000 | -2.07645900 |
| C | 1.80371500 | 2.43887500 | 1.97501300 |
| C | 1.47570300 | 4.65552600 | 1.05042400 |
| H | -2.61099200 | 3.95275400 | -0.00487300 |
| H | -1.26186200 | 4.61426000 | 0.95014300 |
| H | -1.75043800 | 5.41061000 | -0.57138300 |
| H | 2.49633100 | 3.31231500 | -2.99573900 |
| H | 1.32156900 | 4.45455800 | -2.27608400 |
| H | 2.73001900 | 3.89166200 | -1.32473500 |
| C | 2.56882100 | 2.98330600 | 3.00726500 |
| H | 1.64703900 | 1.36726200 | 1.90780500 |
| C | 2.23532500 | 5.19928200 | 2.08824600 |
| H | 1.05553200 | 5.30610300 | 0.28879000 |
| C | 2.78159800 | 4.36366000 | 3.06709400 |
| H | 2.99789500 | 2.32977000 | 3.76193800 |
| H | 2.40202300 | 6.27235300 | 2.13296300 |
| H | 3.37254000 | 4.78866300 | 3.87417100 |
| C | 6.20365300 | 0.01797300 | -3.40711300 |
| H | 6.30157200 | -0.73175800 | -4.20305900 |
| H | 6.02167400 | 0.99025100 | -3.88278400 |
| H | 7.16528500 | 0.07596000 | -2.88067500 |
| C | 5.06571200 | -0.34869500 | -2.44166000 |
| C | 4.89518500 | 0.76969700 | -1.39261700 |
| C | 3.73205900 | -0.46655200 | -3.21644800 |
| C | 3.76410500 | 0.48809200 | -0.39562700 |
| H | 4.67369700 | 1.70532700 | -1.92709900 |
| H | 5.84173400 | 0.93178800 | -0.85713400 |
| C | 2.51605200 | -0.50195100 | -2.32241800 |
| H | 3.76424700 | -1.37204700 | -3.84392800 |
| C | 2.50963700 | -0.07265500 | -1.04188600 |
| H | 3.48951900 | 1.41017100 | 0.12977900 |
| H | 4.09270200 | -0.20605200 | 0.39030400 |
| H | 1.59237500 | -0.88263500 | -2.74213600 |
| C | 5.39982400 | -1.68851800 | -1.75754200 |
| H | 5.62189900 | -2.45901800 | -2.50690700 |
| H | 6.27733300 | -1.59091600 | -1.10524200 |
| H | 4.55908000 | -2.04539400 | -1.15547700 |
| O | 1.47476800 | -0.12442600 | -0.20341100 |
| C | 2.73821400 | -2.69926300 | 2.69258400 |
| C | 1.73852500 | -3.47500200 | 1.75087100 |
| O | 2.24291800 | -1.32799800 | 2.62827900 |
| O | 0.53813600 | -2.64294200 | 1.82423400 |
| C | 2.64009800 | -3.12080100 | 4.16393100 |
| H | 3.03999100 | -4.12753600 | 4.32247600 |
| H | 3.21660600 | -2.41797700 | 4.77343300 |
| H | 1.60176300 | -3.09715900 | 4.50972400 |
| C | 4.19304000 | -2.71579100 | 2.23475600 |
| H | 4.80237400 | -2.12453200 | 2.92613300 |
| H | 4.58129000 | -3.74048300 | 2.22675800 |
| H | 4.30475600 | -2.29275900 | 1.23537300 |
| C | 2.17735700 | -3.50060200 | 0.28451500 |
| H | 3.07587000 | -4.11308800 | 0.15353000 |
| H | 1.37132800 | -3.92307400 | -0.31989600 |
| H | 2.35614800 | -2.49230700 | -0.09220300 |
| C | 1.37057700 | -4.87923100 | 2.22187000 |
| H | 0.65794900 | -5.32387300 | 1.51922900 |
| H | 2.25941500 | -5.51915400 | 2.25479600 |
| H | 0.90903500 | -4.86888900 | 3.21201000 |
| B | 0.94576600 | -1.38924300 | 2.19758200 |
| H | 0.19402200 | -0.47032600 | 2.27839000 |

## VI

E = -799.5914689

$G_{Corr}$= 0.33539

| | | | |
|---|---|---|---|
| C | -5.17342300 | 0.70709600 | 0.17918500 |
| H | -5.53141000 | 1.26764900 | -0.69356300 |
| H | -5.76090100 | -0.21691200 | 0.24989900 |
| H | -5.38199100 | 1.30840700 | 1.07286300 |

| | | | |
|---|---|---|---|
| C | -3.67179300 | 0.40391700 | 0.05930200 |
| C | -3.22091900 | -0.45661000 | 1.26136900 |
| C | -1.72562700 | -0.80718600 | 1.21139500 |
| H | -3.80806900 | -1.38523800 | 1.26203900 |
| H | -3.44872800 | 0.06279700 | 2.20103500 |
| C | -2.03214100 | -1.02345100 | -1.27365300 |
| H | -1.50092900 | -1.64117400 | 1.88958300 |
| H | -1.11048500 | 0.03651000 | 1.55330300 |
| H | -1.62410900 | -1.33281800 | -2.23320000 |
| C | -2.88810400 | 1.73180200 | 0.02110900 |
| H | -3.25917100 | 2.37228300 | -0.78855400 |
| H | -3.00885400 | 2.27973100 | 0.96409100 |
| H | -1.81807600 | 1.56981900 | -0.14457500 |
| O | -0.01189700 | -1.73241500 | -0.26186800 |
| C | 2.34766000 | 0.96303200 | -0.12408400 |
| C | 3.23560400 | -0.31150700 | 0.14342400 |
| O | 1.00136900 | 0.44335700 | 0.07463400 |
| O | 2.35266400 | -1.39661500 | -0.26539100 |
| C | 2.57009200 | 2.12040600 | 0.84360100 |
| H | 3.60383100 | 2.47813200 | 0.78349400 |
| H | 1.90544500 | 2.95062700 | 0.58384600 |
| H | 2.35933700 | 1.82718600 | 1.87438700 |
| C | 2.42043600 | 1.45415300 | -1.57437100 |
| H | 1.62904600 | 2.19194600 | -1.73798000 |
| H | 3.38503100 | 1.92359200 | -1.79162300 |
| H | 2.26721700 | 0.62853100 | -2.27668400 |
| C | 4.51617100 | -0.38742300 | -0.68125600 |
| H | 5.16551500 | 0.46697300 | -0.46059800 |
| H | 5.06094900 | -1.30385600 | -0.43301100 |
| H | 4.30305400 | -0.39927700 | -1.75238700 |
| C | 3.54168300 | -0.52662700 | 1.63026000 |
| H | 3.97262100 | -1.52358600 | 1.76257200 |
| H | 4.25549500 | 0.21400500 | 2.00443900 |
| H | 2.62885000 | -0.46725000 | 2.23179300 |
| B | 1.07263700 | -0.91371100 | -0.15357600 |
| C | -1.28933500 | -1.17990800 | -0.17717000 |
| C | -3.40848400 | -0.40603000 | -1.23417100 |
| H | -3.54117600 | 0.24806100 | -2.10759800 |
| H | -4.17368900 | -1.19218600 | -1.33434600 |

## TS II-IV’

E = -4181.575283
$G_{Corr}$= 0.651225

| | | | |
|---|---|---|---|
| Co | 0.13244500 | 0.02619600 | 0.07791100 |
| H | 1.35647700 | -0.12209900 | -0.84141500 |
| P | -1.01297400 | -0.73458700 | -1.66118300 |
| O | -0.40268600 | -2.18787600 | -2.16435600 |
| O | -0.86351100 | 0.05183100 | -3.13320500 |
| C | -2.84613000 | -1.00926200 | -1.66523300 |
| C | -0.89684900 | -2.88238000 | -3.31685900 |
| C | -1.32300300 | 1.40431400 | -3.26066100 |
| C | -3.49320000 | -1.16533400 | -0.43322400 |
| C | -3.61777700 | -1.01748700 | -2.83887700 |
| H | -0.26680000 | -3.76699400 | -3.43619100 |
| H | -1.93753700 | -3.19492200 | -3.17251900 |
| H | -0.82576000 | -2.25710600 | -4.21200700 |
| H | -1.15213700 | 1.69296500 | -4.30128400 |
| H | -2.39675400 | 1.47926000 | -3.04452400 |
| H | -0.76386400 | 2.06230900 | -2.59159000 |
| C | -4.87524000 | -1.34185600 | -0.36411800 |
| H | -2.90585800 | -1.12002900 | 0.47659600 |
| C | -5.00188100 | -1.18821900 | -2.77534400 |
| H | -3.13029500 | -0.87669000 | -3.79904000 |
| C | -5.63252200 | -1.35252100 | -1.53807900 |
| H | -5.36047000 | -1.44984600 | 0.60214100 |
| H | -5.58976700 | -1.18924000 | -3.68961900 |
| H | -6.71095500 | -1.47796600 | -1.48990600 |
| P | -0.99829400 | 0.84806400 | 1.76847200 |
| O | -0.15486300 | 2.05137900 | 2.50598000 |
| O | -1.43847000 | 0.03125800 | 3.14623600 |
| C | -2.60984600 | 1.63623800 | 1.33162600 |
| C | -0.73122000 | 2.84799300 | 3.55195400 |
| C | -2.05037300 | -1.25159700 | 3.08812800 |
| C | -2.76349100 | 2.19567300 | 0.05352800 |
| C | -3.70831500 | 1.61860900 | 2.20448900 |
| H | 0.02479700 | 3.58684800 | 3.82637600 |
| H | -1.63402700 | 3.36249600 | 3.20385000 |
| H | -0.97594200 | 2.22978100 | 4.42128200 |
| H | -1.52484000 | -1.91620200 | 2.39955100 |
| H | -1.99792400 | -1.67598200 | 4.09393300 |
| H | -3.10380700 | -1.17283300 | 2.78844500 |
| C | -3.99149600 | 2.72246400 | -0.34471600 |
| H | -1.91528100 | 2.20816700 | -0.61946400 |
| C | -4.94160100 | 2.13644600 | 1.80042900 |
| H | -3.59768800 | 1.19588500 | 3.19834100 |
| C | -5.08612700 | 2.68608200 | 0.52444100 |
| H | -4.09809100 | 3.15129700 | -1.33782000 |
| H | -5.78819900 | 2.10995500 | 2.48171800 |
| H | -6.04714200 | 3.08314700 | 0.20832200 |
| P | 1.10542100 | -1.61361700 | 1.03189800 |
| O | 0.74188900 | -3.18730400 | 0.63339500 |
| O | 1.05219700 | -1.64732500 | 2.68775100 |
| C | 2.89673500 | -1.79001300 | 0.67855500 |
| C | -0.60542200 | -3.64580000 | 0.53897100 |
| C | 1.41813500 | -2.83014600 | 3.41551800 |
| C | 3.86699100 | -1.41396800 | 1.61634600 |
| C | 3.29824800 | -2.22841800 | -0.59275600 |
| H | -1.28987100 | -2.84524600 | 0.24808000 |
| H | -0.63514900 | -4.42219900 | -0.22909600 |
| H | -0.92743600 | -4.07159600 | 1.49809700 |
| H | 1.50598500 | -2.53364100 | 4.46321000 |
| H | 0.64320500 | -3.59778600 | 3.31800800 |
| H | 2.37260500 | -3.24155000 | 3.07104700 |
| C | 5.22507200 | -1.49936500 | 1.29660200 |
| H | 3.55954700 | -1.05040300 | 2.59260700 |
| C | 4.65355300 | -2.31001400 | -0.91033600 |
| H | 2.54515800 | -2.50150800 | -1.32671400 |
| C | 5.61990500 | -1.95139900 | 0.03583500 |
| H | 5.97172300 | -1.20801200 | 2.03039400 |
| H | 4.95791700 | -2.65304900 | -1.89556300 |
| H | 6.67551400 | -2.01272700 | -0.21442600 |
| C | 4.84194800 | 3.96340800 | -1.87177900 |
| H | 5.43306900 | 3.76454500 | -2.77455700 |
| H | 4.04908800 | 4.67347200 | -2.13504400 |
| H | 5.49994000 | 4.44426800 | -1.13730000 |
| C | 4.25197600 | 2.66020000 | -1.29533400 |
| C | 3.45156800 | 2.98442300 | -0.01187100 |
| C | 3.33136500 | 2.03564800 | -2.32638100 |
| C | 2.54003100 | 1.82404000 | 0.42561700 |
| H | 2.83044900 | 3.87192500 | -0.19653200 |
| H | 4.14528200 | 3.24441000 | 0.79840000 |
| C | 2.11872800 | 1.53089700 | -2.06470300 |
| H | 3.70373900 | 2.01865500 | -3.35173900 |
| C | 1.54093300 | 1.51893200 | -0.68338200 |
| H | 1.97862300 | 2.06355200 | 1.33072800 |
| H | 3.14898200 | 0.94067700 | 0.63091900 |
| H | 1.48543900 | 1.12920500 | -2.85053800 |
| C | 5.40835500 | 1.67981300 | -0.99411400 |
| H | 5.95362200 | 1.42904400 | -1.91240600 |
| H | 6.12080800 | 2.13169400 | -0.29172400 |
| H | 5.04446100 | 0.74455000 | -0.56078400 |
| O | 0.29626300 | 1.90427700 | -0.56421100 |

## IV’

E = -4181.596814
$G_{Corr}$= 0.652491

| | | | |
|---|---|---|---|
| Co | -0.00822600 | -0.43826700 | 0.23767100 |
| H | 1.90129100 | 0.76579100 | 1.62897600 |
| P | -0.24571100 | -0.51006800 | -1.94658000 |
| O | 0.08580300 | -1.93091100 | -2.71432900 |
| O | 0.53880100 | 0.49112900 | -3.00328800 |
| C | -2.00755700 | -0.16993500 | -2.38355900 |
| C | -0.16517100 | -2.13685800 | -4.11203700 |
| C | 0.49845000 | 1.91732000 | -2.78475600 |
| C | -3.00279300 | -0.41818900 | -1.42646500 |
| C | -2.38022200 | 0.35428700 | -3.63211300 |
| H | 0.20166900 | -3.13902400 | -4.34469300 |
| H | -1.23818900 | -2.08211500 | -4.32809100 |
| H | 0.36785700 | -1.39792000 | -4.71734000 |
| H | 1.32282500 | 2.34062400 | -3.36268400 |
| H | -0.45194000 | 2.32528700 | -3.15243000 |
| H | 0.62408500 | 2.13485700 | -1.72297300 |
| C | -4.34337200 | -0.15373100 | -1.70718400 |
| H | -2.71083300 | -0.79195100 | -0.44971100 |
| C | -3.72153100 | 0.61941600 | -3.91481500 |
| H | -1.61618700 | 0.56167800 | -4.37545400 |
| C | -4.70424600 | 0.36615000 | -2.95290700 |
| H | -5.10036100 | -0.33545000 | -0.94914000 |
| H | -3.99973300 | 1.02836600 | -4.88256900 |
| H | -5.74671100 | 0.58143000 | -3.17189900 |
| P | -1.04644700 | 0.13492200 | 2.04232200 |
| O | -0.14902500 | 0.81516000 | 3.23608800 |
| O | -1.88911500 | -0.95189000 | 2.98461700 |
| C | -2.40059500 | 1.34570300 | 1.68984000 |
| C | -0.75160300 | 1.43761300 | 4.38081200 |
| C | -2.77091900 | -1.87782100 | 2.35267400 |
| C | -2.29350500 | 2.23246400 | 0.60501000 |
| C | -3.58108200 | 1.34472800 | 2.45330100 |
| H | 0.07073200 | 1.79796600 | 5.00242900 |
| H | -1.38048300 | 2.28327900 | 4.08142300 |
| H | -1.35180100 | 0.71692800 | 4.94541300 |
| H | -2.27215800 | -2.41637600 | 1.54133800 |
| H | -3.07934400 | -2.59619900 | 3.11606600 |
| H | -3.65988200 | -1.36997400 | 1.95544200 |
| C | -3.34826200 | 3.08952100 | 0.28918600 |
| H | -1.37198500 | 2.23995700 | 0.03475800 |
| C | -4.63449600 | 2.20378300 | 2.13385500 |
| H | -3.67478100 | 0.66895600 | 3.29761500 |
| C | -4.52198600 | 3.07488500 | 1.04716300 |
| H | -3.25498200 | 3.76669700 | -0.55603200 |
| H | -5.54187300 | 2.19121900 | 2.73226900 |
| H | -5.34479100 | 3.73852600 | 0.79391500 |
| P | 0.40986100 | -2.48967900 | 0.57124900 |
| O | 0.07784400 | -3.80464400 | -0.37200600 |
| O | 0.18408600 | -3.06608100 | 2.10148200 |
| C | 2.22260800 | -2.44186500 | 0.37842900 |
| C | -1.27348700 | -4.00552200 | -0.80338900 |
| C | 0.67434600 | -4.36433800 | 2.47596500 |
| C | 3.01361800 | -2.03948300 | 1.46665200 |
| C | 2.81333600 | -2.62371400 | -0.88216600 |
| H | -1.73609100 | -3.06432300 | -1.11630900 |
| H | -1.23702100 | -4.68455700 | -1.65799400 |
| H | -1.87053100 | -4.46071200 | -0.00338100 |
| H | 0.40493400 | -4.50556800 | 3.52461100 |
| H | 0.21116900 | -5.14592200 | 1.86544500 |
| H | 1.76319100 | -4.42021300 | 2.36665700 |
| C | 4.38327700 | -1.83467200 | 1.29870700 |
| H | 2.55062700 | -1.87887800 | 2.43622400 |
| C | 4.18406200 | -2.41729100 | -1.04389500 |
| H | 2.19276400 | -2.90475900 | -1.72627100 |
| C | 4.96914800 | -2.02189800 | 0.04362100 |
| H | 4.99087200 | -1.52187900 | 2.14330500 |
| H | 4.63912700 | -2.55692800 | -2.02080800 |
| H | 6.03449200 | -1.85374900 | -0.08832300 |
| C | 3.52560900 | 4.44541000 | -1.79073000 |
| H | 4.43080000 | 4.26008900 | -2.38317400 |
| H | 2.65855900 | 4.17988900 | -2.40659500 |
| H | 3.47561900 | 5.52138000 | -1.57981500 |
| C | 3.54126400 | 3.63370000 | -0.47952900 |
| C | 2.25849400 | 3.94235300 | 0.32991800 |
| C | 3.60037900 | 2.15235700 | -0.80861000 |
| C | 1.99001200 | 2.90227400 | 1.42142500 |
| H | 1.39997200 | 3.94760000 | -0.35419600 |
| H | 2.33053000 | 4.95226700 | 0.75645900 |
| C | 2.84234200 | 1.21249200 | -0.23500200 |
| H | 4.32848100 | 1.86814200 | -1.57155300 |
| C | 1.78785300 | 1.50380200 | 0.81375900 |
| H | 1.09220700 | 3.15617500 | 1.99477000 |
| H | 2.83053500 | 2.87062700 | 2.12835100 |
| H | 2.92739500 | 0.17519000 | -0.55111500 |
| C | 4.80460900 | 4.02320500 | 0.32262500 |
| H | 5.71403000 | 3.76911800 | -0.23608200 |
| H | 4.82142000 | 5.10287800 | 0.52174800 |
| H | 4.84710700 | 3.49687500 | 1.28200300 |
| O | 0.49066300 | 1.41141400 | 0.25027700 |

# V’

E = -4593.680624

$G_{Corr}$= 0.8345

| | | | |
|---|---|---|---|
| Co | 0.61942500 | 0.55934700 | 0.26013500 |
| H | 1.28485300 | -2.49409900 | 1.22312100 |
| P | 0.91979300 | 0.65092500 | -1.91979300 |
| O | 1.16822400 | 2.15073400 | -2.57838900 |
| O | -0.24234100 | -0.00220100 | -2.88584200 |
| C | 2.38671500 | -0.24953300 | -2.54752700 |
| C | 1.29553200 | 2.33894400 | -3.99285900 |
| C | -1.53148500 | -0.25985100 | -2.28722700 |
| C | 3.65410400 | 0.21106800 | -2.16257600 |
| C | 2.28716100 | -1.39312500 | -3.34845200 |
| H | 1.36421800 | 3.41643200 | -4.16022300 |
| H | 2.20020700 | 1.84856300 | -4.37164000 |
| H | 0.42008200 | 1.94274100 | -4.51678700 |
| H | -1.99422700 | 0.66241400 | -1.92564100 |
| H | -2.15011100 | -0.69509300 | -3.07604700 |
| H | -1.41748200 | -0.96167600 | -1.45899400 |
| C | 4.80535600 | -0.47137300 | -2.55873300 |
| H | 3.74134500 | 1.09593100 | -1.53847900 |
| C | 3.43851300 | -2.07643400 | -3.74458300 |
| H | 1.30820400 | -1.74627900 | -3.64996100 |
| C | 4.69869800 | -1.62124100 | -3.34674900 |
| H | 5.78206500 | -0.10953000 | -2.24875200 |
| H | 3.35093400 | -2.96804600 | -4.35991800 |
| H | 5.59340600 | -2.15804400 | -3.65072500 |
| P | 1.30004600 | 0.16511100 | 2.28871400 |
| O | 0.39046700 | -0.93845400 | 3.10025900 |
| O | 1.44524400 | 1.25458500 | 3.53606900 |
| C | 3.01804000 | -0.52397400 | 2.27758400 |
| C | 0.77679700 | -1.51280600 | 4.35671000 |
| C | 2.27561600 | 2.41197000 | 3.42872000 |
| C | 3.55312300 | -1.02085100 | 1.07805500 |
| C | 3.81797500 | -0.52973200 | 3.43363600 |
| H | -0.04947200 | -2.15665900 | 4.66544100 |
| H | 1.68610600 | -2.11436700 | 4.24694300 |
| H | 0.93923300 | -0.73486500 | 5.10901600 |
| H | 1.86152100 | 3.13201800 | 2.71990600 |
| H | 2.30373000 | 2.86584500 | 4.42269100 |
| H | 3.29795800 | 2.14682200 | 3.13194000 |
| C | 4.85515200 | -1.52171100 | 1.03767100 |
| H | 2.93693200 | -1.01853600 | 0.18465200 |
| C | 5.11994700 | -1.03189400 | 3.39177100 |
| H | 3.42288500 | -0.13028100 | 4.36292200 |
| C | 5.64089100 | -1.52878500 | 2.19315100 |
| H | 5.25211200 | -1.89759000 | 0.09845500 |
| H | 5.72887800 | -1.03082000 | 4.29213400 |
| H | 6.65667800 | -1.91436800 | 2.16091700 |
| P | 0.12927300 | 2.63002400 | 0.48740900 |

| | | | |
|---|---|---|---|
| O | 1.31995300 | 3.77891900 | 0.33825100 |
| O | -0.52211600 | 2.99079000 | 1.95950800 |
| C | -1.11781000 | 3.37042800 | -0.64021100 |
| C | 2.66600000 | 3.37873200 | 0.05029800 |
| C | -0.86286400 | 4.33854900 | 2.31542600 |
| C | -2.45989600 | 2.99597800 | -0.48235500 |
| C | -0.76527100 | 4.23520900 | -1.68525800 |
| H | 2.84892600 | 2.34973300 | 0.38199400 |
| H | 2.84206900 | 3.43802000 | -1.02763700 |
| H | 3.33386300 | 4.06398100 | 0.57980200 |
| H | -1.23988500 | 4.30209700 | 3.33961200 |
| H | 0.02029000 | 4.98475800 | 2.27142900 |
| H | -1.63875200 | 4.73784100 | 1.65279400 |
| C | -3.43353600 | 3.46314400 | -1.36652700 |
| H | -2.74828500 | 2.33043500 | 0.32109400 |
| C | -1.74047400 | 4.70625300 | -2.56590900 |
| H | 0.27055100 | 4.52837100 | -1.80779300 |
| C | -3.07470600 | 4.31695800 | -2.41264500 |
| H | -4.46736600 | 3.15379600 | -1.23763900 |
| H | -1.45879500 | 5.37654900 | -3.37399000 |
| H | -3.83029700 | 4.67978500 | -3.10466900 |
| C | -2.24125600 | -4.30332900 | -2.25608600 |
| H | -3.19850100 | -4.69608500 | -1.88930200 |
| H | -2.39093600 | -3.25513500 | -2.53787200 |
| H | -1.97273900 | -4.86275800 | -3.16144400 |
| C | -1.13573200 | -4.43520800 | -1.18879600 |
| C | 0.18387500 | -3.85348100 | -1.74763100 |
| C | -1.54301100 | -3.67207000 | 0.05887000 |
| C | 1.20672300 | -3.55841700 | -0.64708800 |
| H | -0.03780400 | -2.90662100 | -2.25290400 |
| H | 0.59631000 | -4.53585800 | -2.50358300 |
| C | -0.75532400 | -2.82400000 | 0.73048600 |
| H | -2.55265600 | -3.86775200 | 0.42519500 |
| C | 0.66012100 | -2.49103500 | 0.31535700 |
| H | 2.14207100 | -3.18953700 | -1.08151200 |
| H | 1.44067500 | -4.47336000 | -0.08498700 |
| H | -1.12826200 | -2.30684600 | 1.60854100 |
| C | -0.97498700 | -5.93063700 | -0.83083200 |
| H | -1.90207400 | -6.33085000 | -0.40149500 |
| H | -0.73549600 | -6.52009500 | -1.72558000 |
| H | -0.17674700 | -6.08236800 | -0.09665200 |
| O | 0.71046500 | -1.23313200 | -0.31870700 |
| C | -4.94038200 | -1.28444300 | 1.53704300 |
| C | -4.90209300 | -0.62282000 | 0.10634400 |
| O | -3.53710600 | -1.25305700 | 1.93996700 |
| O | -3.70003300 | 0.19986500 | 0.17677700 |
| C | -5.70292200 | -0.44913000 | 2.57199300 |
| H | -6.78031400 | -0.45495100 | 2.37827500 |
| H | -5.52542900 | -0.86888800 | 3.56673800 |
| H | -5.35205800 | 0.58770500 | 2.57448800 |
| C | -5.42200300 | -2.73154600 | 1.56514400 |
| H | -5.38319600 | -3.11136500 | 2.59095600 |
| H | -6.45750400 | -2.79894200 | 1.21347800 |
| H | -4.79866300 | -3.37557700 | 0.94158600 |
| C | -4.67086000 | -1.62737300 | -1.02623500 |
| H | -5.54153400 | -2.27359700 | -1.17636000 |
| H | -4.48289200 | -1.07837500 | -1.95419800 |
| H | -3.79782400 | -2.25071600 | -0.82092400 |
| C | -6.08308900 | 0.28626200 | -0.22124700 |
| H | -5.94501500 | 0.71671500 | -1.21848400 |
| H | -7.02036100 | -0.28092200 | -0.22012000 |
| H | -6.16940400 | 1.10808300 | 0.49313300 |
| B | -2.91675700 | -0.29370300 | 1.18722500 |
| H | -1.81729400 | 0.10088600 | 1.39680500 |

## TS V'-VI'

E = -4593.668967

$G_{Corr}$= 0.836273

| | | | |
|---|---|---|---|
| Co | -0.31620000 | -0.09538100 | -0.30089900 |
| P | -2.36783400 | 0.72217200 | -0.47469300 |
| O | -3.57345800 | -0.24105400 | 0.06878800 |
| O | -2.89506600 | 1.00168000 | -2.01889500 |
| C | -2.80652100 | 2.33070800 | 0.30907100 |
| C | -4.95755000 | 0.07192300 | -0.14568900 |
| C | -1.98018700 | 1.35971700 | -3.06484800 |
| C | -2.89602400 | 2.41916000 | 1.70721600 |
| C | -3.08799200 | 3.46437300 | -0.46593500 |
| H | -5.52687800 | -0.68464600 | 0.39729500 |
| H | -5.20119900 | 1.06842200 | 0.23955200 |
| H | -5.19744100 | 0.01553800 | -1.21081500 |
| H | -2.20810000 | 0.74127000 | -3.93539400 |
| H | -2.10853000 | 2.41857800 | -3.31373300 |
| H | -0.94628300 | 1.19096300 | -2.76176900 |
| C | -3.25929000 | 3.62209500 | 2.31458000 |
| H | -2.66958800 | 1.55412000 | 2.32049800 |
| C | -3.43958900 | 4.67025100 | 0.14474600 |
| H | -3.04643700 | 3.40633200 | -1.54705700 |
| C | -3.52705500 | 4.75167900 | 1.53657900 |
| H | -3.32679400 | 3.67837200 | 3.39785900 |
| H | -3.65011900 | 5.54303200 | -0.46758000 |
| H | -3.80275700 | 5.68942500 | 2.01150200 |
| P | -0.67348900 | -1.67859700 | -1.74656300 |
| O | 0.48379700 | -2.85967300 | -1.61197200 |
| O | -0.85981800 | -1.58802900 | -3.40768200 |
| C | -2.22436900 | -2.60623200 | -1.43492600 |
| C | 0.37436300 | -4.10308200 | -2.31937100 |
| C | 0.22681700 | -1.03186300 | -4.15598500 |
| C | -2.29407700 | -3.39626200 | -0.27726200 |
| C | -3.36333700 | -2.45785000 | -2.23613300 |
| H | 1.34515800 | -4.59627700 | -2.23146400 |
| H | -0.40241600 | -4.73440100 | -1.87463300 |
| H | 0.13977100 | -3.93548600 | -3.37536300 |
| H | 0.69442800 | -0.19666400 | -3.62578000 |
| H | 0.98913600 | -1.79605200 | -4.34998100 |
| H | -0.18027800 | -0.67836100 | -5.10696500 |
| C | -3.48537100 | -4.03274200 | 0.07105600 |
| H | -1.41676300 | -3.50714800 | 0.35487500 |
| C | -4.55687200 | -3.09025500 | -1.88224400 |
| H | -3.31687500 | -1.83988400 | -3.12620100 |
| C | -4.62167000 | -3.87592300 | -0.72866700 |
| H | -3.52837000 | -4.64693200 | 0.96682600 |
| H | -5.43775100 | -2.96793200 | -2.50725700 |
| H | -5.55222200 | -4.36604100 | -0.45461700 |
| P | -0.23208800 | -0.33168000 | 1.89434700 |
| O | -1.57201800 | -0.24131000 | 2.89664700 |
| O | 0.66489600 | 0.84138600 | 2.61579200 |
| C | 0.46212200 | -1.92333500 | 2.53125600 |
| C | -2.56114000 | -1.28095700 | 2.84054700 |
| C | 0.98562600 | 0.92386400 | 4.00812900 |
| C | 0.98360200 | -2.85779500 | 1.62813700 |
| C | 0.41757700 | -2.25501400 | 3.89796400 |
| H | -3.48059500 | -0.85975500 | 3.25531900 |
| H | -2.75506700 | -1.60131600 | 1.81425400 |
| H | -2.24641200 | -2.13980700 | 3.44400000 |
| H | 1.45311200 | 1.90055700 | 4.15314200 |
| H | 0.08424300 | 0.85401600 | 4.62532700 |
| H | 1.69314400 | 0.13770200 | 4.29267300 |
| C | 1.48485800 | -4.08172200 | 2.07762400 |
| H | 0.98013000 | -2.63497200 | 0.57037300 |
| C | 0.92252500 | -3.47493200 | 4.34852400 |
| H | -0.03632700 | -1.57055700 | 4.60713400 |
| C | 1.46429700 | -4.38912000 | 3.43911800 |
| H | 1.88767900 | -4.79261100 | 1.36102100 |
| H | 0.88481500 | -3.71685000 | 5.40742400 |
| H | 1.85502500 | -5.34007900 | 3.79134900 |
| C | 6.85888500 | -1.78862500 | -0.42357100 |
| H | 7.53010800 | -1.47134600 | 0.38480500 |
| H | 6.47140400 | -2.78267100 | -0.16966100 |
| H | 7.45603900 | -1.87976200 | -1.34023000 |
| C | 5.70920200 | -0.78058300 | -0.61993300 |

| | | | |
|---|---|---|---|
| C | 4.80316900 | -1.25000900 | -1.78416800 |
| C | 4.88924600 | -0.69157000 | 0.65431800 |
| C | 3.45344100 | -0.52373800 | -1.80509100 |
| H | 4.62618500 | -2.33081200 | -1.67617000 |
| H | 5.33383100 | -1.11420800 | -2.73671100 |
| C | 3.55365700 | -0.71879900 | 0.69319400 |
| H | 5.45848300 | -0.57784000 | 1.57931800 |
| H | 2.86252300 | -0.82634900 | -2.67778200 |
| H | 3.60446700 | 0.55577400 | -1.87602000 |
| H | 3.02830100 | -0.63322500 | 1.64235100 |
| C | 6.31458600 | 0.61020200 | -0.92378300 |
| H | 6.92067000 | 0.96177500 | -0.07931300 |
| H | 6.96338900 | 0.56782200 | -1.80870200 |
| H | 5.53230400 | 1.35454600 | -1.10442200 |
| O | 1.55504800 | 0.03759500 | -0.37327400 |
| C | 2.65479500 | -0.82150900 | -0.52500200 |
| H | 2.31349200 | -1.86232100 | -0.60832600 |
| C | 0.87442200 | 3.79760800 | -0.22910500 |
| C | 2.42741600 | 3.54967000 | -0.23175000 |
| O | 0.35990800 | 2.61291900 | -0.92296200 |
| O | 2.60709500 | 2.73205100 | -1.42311100 |
| C | 0.26419100 | 3.89682200 | 1.16236600 |
| H | 0.76989300 | 4.68615100 | 1.73193800 |
| H | -0.79337900 | 4.15511500 | 1.09325400 |
| H | 0.35790300 | 2.95338900 | 1.70029200 |
| C | 0.44717900 | 4.99761000 | -1.08349100 |
| H | -0.64221100 | 4.98232900 | -1.18459100 |
| H | 0.73877700 | 5.94541300 | -0.61879200 |
| H | 0.88845000 | 4.94727100 | -2.08420600 |
| C | 3.27282800 | 4.81099200 | -0.38900000 |
| H | 3.09511100 | 5.49923300 | 0.44522800 |
| H | 4.33405300 | 4.54110100 | -0.38749100 |
| H | 3.05628900 | 5.33133000 | -1.32513500 |
| C | 2.90997400 | 2.72596200 | 0.96555000 |
| H | 3.94933800 | 2.42918700 | 0.79386600 |
| H | 2.86238900 | 3.30991700 | 1.89152800 |
| H | 2.31598600 | 1.81843100 | 1.07325400 |
| B | 1.41158100 | 2.10355300 | -1.66596800 |
| H | 1.25195400 | 1.43747800 | -2.63314900 |

# TS V’-XI

E = -4593.669231

$G_{Corr}$= 0.841354

| | | | |
|---|---|---|---|
| Co | 0.49155500 | -0.45824900 | 0.30859100 |
| H | -2.43162500 | -0.29038900 | 1.24497000 |
| P | 0.59084600 | -0.31135400 | -1.86978900 |
| O | 1.81430900 | -1.26320600 | -2.47566900 |
| O | 0.78249900 | 1.09464500 | -2.72214700 |
| C | -0.82534000 | -0.91308300 | -2.87463000 |
| C | 2.06635200 | -1.36568700 | -3.88199100 |
| C | 1.96377300 | 1.88853000 | -2.50514900 |
| C | -1.08927900 | -2.29110500 | -2.86896900 |
| C | -1.63939000 | -0.06436100 | -3.63612700 |
| H | 3.10896000 | -1.67418200 | -3.99352400 |
| H | 1.40678300 | -2.11484800 | -4.33387200 |
| H | 1.91520900 | -0.40503000 | -4.38367800 |
| H | 2.86479800 | 1.32372200 | -2.76882700 |
| H | 1.87152100 | 2.75319900 | -3.16796000 |
| H | 2.01882900 | 2.21772400 | -1.46886200 |
| C | -2.16612200 | -2.80994300 | -3.58899200 |
| H | -0.45093600 | -2.95961600 | -2.29807300 |
| C | -2.72012200 | -0.58297800 | -4.35205600 |
| H | -1.43159400 | 0.99887900 | -3.65535700 |
| C | -2.99082100 | -1.95327100 | -4.32457300 |
| H | -2.35977300 | -3.87923500 | -3.57692300 |
| H | -3.35372500 | 0.08572000 | -4.92844600 |
| H | -3.83546500 | -2.35357000 | -4.87875000 |
| P | -0.30992400 | -1.66151200 | 2.02886300 |
| O | -0.99757600 | -0.64779200 | 3.12129500 |
| O | 0.55775200 | -2.55957600 | 3.13195500 |
| C | -1.61658300 | -2.89334000 | 1.60008400 |
| C | -1.76858200 | -1.08893900 | 4.24564400 |
| C | 1.25152300 | -3.75392100 | 2.77496600 |
| C | -2.13530000 | -2.86158300 | 0.30010200 |
| C | -2.08665500 | -3.86601900 | 2.49854000 |
| H | -2.00287100 | -0.19357800 | 4.82511600 |
| H | -2.70012300 | -1.56284400 | 3.91592500 |
| H | -1.19718200 | -1.78852100 | 4.86373500 |
| H | 2.14163800 | -3.53070800 | 2.18264600 |
| H | 1.55718300 | -4.22750900 | 3.71160000 |
| H | 0.60452400 | -4.44781600 | 2.22384900 |
| C | -3.12491100 | -3.76066700 | -0.09765900 |
| H | -1.74961000 | -2.12422600 | -0.39425100 |
| C | -3.07368600 | -4.77019500 | 2.10243900 |
| H | -1.67053100 | -3.92175300 | 3.50001000 |
| C | -3.59760400 | -4.71548600 | 0.80623600 |
| H | -3.51710700 | -3.71303300 | -1.10979400 |
| H | -3.43261000 | -5.51992000 | 2.80266900 |
| H | -4.36616500 | -5.42117800 | 0.50205200 |
| P | 2.53456500 | -1.18609100 | 0.46378500 |
| O | 2.99039100 | -2.68609100 | -0.05024800 |
| O | 3.02277300 | -1.17296800 | 2.03636600 |
| C | 3.84318200 | -0.17100000 | -0.31985400 |
| C | 2.02536600 | -3.60889500 | -0.57429700 |
| C | 4.35256700 | -1.56689400 | 2.40799300 |
| C | 4.02856200 | 1.13708400 | 0.15245000 |
| C | 4.60534300 | -0.62973800 | -1.40163700 |
| H | 1.00512100 | -3.27746200 | -0.35133200 |
| H | 2.14707300 | -3.67488000 | -1.65765900 |
| H | 2.20797800 | -4.58298600 | -0.11207300 |
| H | 4.39412700 | -1.52127700 | 3.49793900 |
| H | 4.56072400 | -2.58919800 | 2.07459100 |
| H | 5.09751200 | -0.88564500 | 1.98244600 |
| C | 4.96215800 | 1.97679200 | -0.45537700 |
| H | 3.41918300 | 1.51265700 | 0.96742200 |
| C | 5.53866100 | 0.21273200 | -2.00876500 |
| H | 4.45733500 | -1.64010900 | -1.76558500 |
| C | 5.71646000 | 1.51746000 | -1.53876200 |
| H | 5.09129700 | 2.99216700 | -0.09037200 |
| H | 6.12568700 | -0.14801000 | -2.84938400 |
| H | 6.43884800 | 2.17394600 | -2.01657000 |
| C | -4.38880900 | 3.92282500 | -1.19539500 |
| H | -4.96269300 | 4.65037300 | -0.60692600 |
| H | -3.35822800 | 4.28646900 | -1.27165500 |
| H | -4.81649100 | 3.89554100 | -2.20597900 |
| C | -4.43531700 | 2.52269200 | -0.54967100 |
| C | -3.62898700 | 1.52628500 | -1.41224800 |
| C | -3.82664200 | 2.59260500 | 0.83832500 |
| C | -3.27959300 | 0.25344600 | -0.64060600 |
| H | -2.68971900 | 2.00118300 | -1.72266900 |
| H | -4.18497200 | 1.29269100 | -2.33002700 |
| C | -2.90100400 | 1.75363700 | 1.31783800 |
| H | -4.18869700 | 3.40940200 | 1.46714800 |
| C | -2.36333600 | 0.56068200 | 0.55684300 |
| H | -2.79252900 | -0.46774000 | -1.29637400 |
| H | -4.19564900 | -0.22584400 | -0.26691900 |
| H | -2.47510700 | 1.91366300 | 2.30282800 |
| C | -5.91378200 | 2.08508100 | -0.43627300 |
| H | -6.47452500 | 2.77061200 | 0.21138900 |
| H | -6.39418500 | 2.08493400 | -1.42353300 |
| H | -6.00348500 | 1.07957500 | -0.01185200 |
| O | -1.01325600 | 0.77358500 | 0.16849400 |
| C | 0.11052600 | 4.04644100 | 2.01387200 |
| C | 0.60903400 | 4.02538200 | 0.52209400 |
| O | -0.14898100 | 2.64204400 | 2.28579200 |
| O | 1.20307500 | 2.70496600 | 0.43164400 |
| C | 1.20053500 | 4.48286300 | 3.00199100 |
| H | 1.42520100 | 5.55117200 | 2.91389500 |
| H | 0.85367900 | 4.28335500 | 4.02072500 |
| H | 2.12216300 | 3.91463800 | 2.83965600 |

| | | | |
|---|---|---|---|
| C | -1.16702300 | 4.84690000 | 2.24703700 |
| H | -1.44697300 | 4.79511300 | 3.30481800 |
| H | -1.01178500 | 5.90037800 | 1.98650200 |
| H | -1.99316100 | 4.45141200 | 1.65590600 |
| C | -0.52949100 | 4.08321600 | -0.50211500 |
| H | -1.00387200 | 5.07038300 | -0.51997900 |
| H | -0.12138600 | 3.87383800 | -1.49617800 |
| H | -1.27811900 | 3.32227400 | -0.27875500 |
| C | 1.68518700 | 5.05392200 | 0.18346000 |
| H | 1.97368400 | 4.94891300 | -0.86783300 |
| H | 1.30938800 | 6.07228200 | 0.33360000 |
| H | 2.58040300 | 4.91626300 | 0.79491400 |
| B | 0.56480700 | 1.90284700 | 1.36108700 |
| H | 1.03075300 | 0.82780800 | 1.64487700 |

# VI’

E = -4593.683488

$G_{Corr}$= 0.840412

| | | | |
|---|---|---|---|
| Co | -0.31707500 | 0.11122300 | -0.30864400 |
| P | -2.47235400 | 0.54053300 | -0.46403000 |
| O | -3.47966100 | -0.65549100 | 0.00513800 |
| O | -3.00214900 | 0.78566900 | -2.00542800 |
| C | -3.20001500 | 1.99617500 | 0.38316300 |
| C | -4.89548700 | -0.59525700 | -0.22552400 |
| C | -2.16113700 | 1.37572300 | -3.01655900 |
| C | -3.23211200 | 2.03256000 | 1.78610200 |
| C | -3.73290300 | 3.06842600 | -0.34561200 |
| H | -5.31935500 | -1.47045900 | 0.26955500 |
| H | -5.32414100 | 0.31898500 | 0.19976700 |
| H | -5.10641300 | -0.64274400 | -1.29702800 |
| H | -2.19457900 | 0.72641200 | -3.89272800 |
| H | -2.55854900 | 2.36441200 | -3.26910500 |
| H | -1.13293400 | 1.48278100 | -2.66765800 |
| C | -3.77759000 | 3.13428900 | 2.44499800 |
| H | -2.81504700 | 1.21231500 | 2.36055400 |
| C | -4.26819700 | 4.17471300 | 0.31795600 |
| H | -3.73659200 | 3.03844200 | -1.42899100 |
| C | -4.28835900 | 4.21080300 | 1.71392600 |
| H | -3.79583300 | 3.15673900 | 3.53137400 |
| H | -4.67011700 | 5.00528300 | -0.25590100 |
| H | -4.70261700 | 5.07269200 | 2.23006500 |
| P | -0.51024400 | -1.54976000 | -1.74445500 |
| O | 0.84376000 | -2.50693100 | -1.67537400 |
| O | -0.75513900 | -1.45460100 | -3.39347300 |
| C | -1.85946700 | -2.75523400 | -1.44166400 |
| C | 0.93029600 | -3.74316100 | -2.40058100 |
| C | 0.23057300 | -0.77899700 | -4.18703100 |
| C | -1.76314100 | -3.57492000 | -0.30772500 |
| C | -3.00668200 | -2.82004100 | -2.24245300 |
| H | 1.98951300 | -4.00821100 | -2.43684100 |
| H | 0.36898300 | -4.52910000 | -1.88464600 |
| H | 0.54209900 | -3.63218900 | -3.41763900 |
| H | 0.55826000 | 0.15107600 | -3.71413000 |
| H | 1.10317500 | -1.42498900 | -4.34358200 |
| H | -0.23378300 | -0.56237400 | -5.15244700 |
| C | -2.80031800 | -4.44821100 | 0.02130900 |
| H | -0.87871700 | -3.52600700 | 0.32177900 |
| C | -4.04703200 | -3.68878700 | -1.90757700 |
| H | -3.08684800 | -2.18416900 | -3.11727300 |
| C | -3.94815700 | -4.50163000 | -0.77511600 |
| H | -2.71444900 | -5.08273000 | 0.89958500 |
| H | -4.93668300 | -3.72997300 | -2.53069100 |
| H | -4.75976800 | -5.17644600 | -0.51613900 |
| P | -0.18998200 | -0.38006000 | 1.88197700 |
| O | -1.54925500 | -0.50613000 | 2.85470100 |
| O | 0.54939000 | 0.83595700 | 2.71077800 |
| C | 0.69432700 | -1.90368500 | 2.45175500 |
| C | -2.41001800 | -1.64979000 | 2.74255600 |
| C | 0.81926800 | 0.86138700 | 4.11713300 |
| C | 1.41153100 | -2.66545400 | 1.52127800 |
| C | 0.64152700 | -2.34212400 | 3.78772400 |
| H | -3.36705400 | -1.36216500 | 3.18551900 |
| H | -2.57517600 | -1.93047300 | 1.70088100 |
| H | -1.99238100 | -2.49947700 | 3.29420400 |
| H | 1.18236400 | 1.86794200 | 4.33667900 |
| H | -0.08933600 | 0.66368000 | 4.69427200 |
| H | 1.59202800 | 0.13111600 | 4.37989800 |
| C | 2.08852700 | -3.82247400 | 1.91451500 |
| H | 1.41515800 | -2.36945300 | 0.48131700 |
| C | 1.31808200 | -3.49642700 | 4.18250800 |
| H | 0.05282600 | -1.79146200 | 4.51444300 |
| C | 2.04939500 | -4.23604000 | 3.24712500 |
| H | 2.64025100 | -4.39969200 | 1.17728200 |
| H | 1.26989400 | -3.82340400 | 5.21785100 |
| H | 2.57461600 | -5.13582300 | 3.55612500 |
| C | 7.03511300 | -1.06392600 | -0.17965400 |
| H | 7.61434300 | -0.69143600 | 0.67465900 |
| H | 6.68027500 | -2.07199300 | 0.06654900 |
| H | 7.71421000 | -1.14196500 | -1.03832000 |
| C | 5.85618800 | -0.12498200 | -0.50288700 |
| C | 5.08186800 | -0.67358400 | -1.72584100 |
| C | 4.92495500 | -0.04873600 | 0.69220500 |
| C | 3.70746600 | -0.01456200 | -1.89761000 |
| H | 4.94810000 | -1.75829400 | -1.59918100 |
| H | 5.68566900 | -0.53657400 | -2.63256500 |
| C | 3.59434900 | -0.12991600 | 0.61426700 |
| H | 5.40086100 | 0.10614100 | 1.66191900 |
| H | 3.20941800 | -0.37820200 | -2.80349100 |
| H | 3.81836600 | 1.06676200 | -1.99862200 |
| H | 2.98040800 | -0.05505800 | 1.50799800 |
| C | 6.40843200 | 1.29100600 | -0.79031600 |
| H | 6.93385100 | 1.68637400 | 0.08781200 |
| H | 7.11946600 | 1.26846500 | -1.62643900 |
| H | 5.60302900 | 1.99003400 | -1.03709800 |
| O | 1.62724600 | 0.44727300 | -0.62762000 |
| C | 2.83425700 | -0.32316900 | -0.67611100 |
| H | 2.52895500 | -1.36859900 | -0.75450800 |
| C | 0.35652600 | 3.54601400 | -0.28055000 |
| C | 1.92351100 | 3.61932600 | -0.14551800 |
| O | 0.13164700 | 2.21011000 | -0.80957200 |
| O | 2.38213000 | 2.77562600 | -1.20076100 |
| C | -0.36683900 | 3.75524600 | 1.04580700 |
| H | 0.01508400 | 4.65995300 | 1.53458100 |
| H | -1.43722900 | 3.89125900 | 0.89103000 |
| H | -0.21334100 | 2.90397400 | 1.71033400 |
| C | -0.19698400 | 4.51066300 | -1.33949600 |
| H | -1.25063500 | 4.26886300 | -1.51320500 |
| H | -0.13340100 | 5.55520400 | -1.01455400 |
| H | 0.34414900 | 4.40007000 | -2.28411700 |
| C | 2.49810700 | 5.02103400 | -0.36869000 |
| H | 2.08371500 | 5.73803700 | 0.35019900 |
| H | 3.58506000 | 4.99360200 | -0.23270800 |
| H | 2.29487000 | 5.37909700 | -1.38098300 |
| C | 2.43128000 | 3.06873000 | 1.19649000 |
| H | 3.51493200 | 2.93116600 | 1.12505200 |
| H | 2.22008400 | 3.75310000 | 2.02692700 |
| H | 1.97856400 | 2.10320400 | 1.41296200 |
| B | 1.40662100 | 1.74283500 | -1.42632200 |
| H | 1.30844900 | 1.47663000 | -2.61087600 |

# IV’’

E = -4181.595696

$G_{Corr}$= 0.65285

| | | | |
|---|---|---|---|
| Co | 0.03268000 | -0.13443000 | -0.04970800 |
| P | -0.03251500 | -2.29331200 | 0.09949700 |
| O | -1.18229200 | -3.18941400 | -0.64754500 |

| | | | |
|---|---|---|---|
| O | 0.01215700 | -3.17107200 | 1.51155100 |
| C | 1.56065500 | -2.79909800 | -0.67991500 |
| C | -1.09482200 | -4.61987600 | -0.74867600 |
| C | 0.94264200 | -2.84423700 | 2.55097000 |
| C | 2.08889400 | -2.02383900 | -1.72772000 |
| C | 2.26880400 | -3.93237000 | -0.24530900 |
| H | -1.96526200 | -4.93839300 | -1.32578300 |
| H | -0.18001700 | -4.91889800 | -1.27243800 |
| H | -1.11659000 | -5.08089700 | 0.24299900 |
| H | 1.23845500 | -3.78357400 | 3.02657500 |
| H | 1.83957000 | -2.35305400 | 2.15725800 |
| H | 0.46660600 | -2.19132400 | 3.28456800 |
| C | 3.30598700 | -2.37118600 | -2.31583000 |
| H | 1.55681500 | -1.14388500 | -2.07652400 |
| C | 3.48309100 | -4.27936700 | -0.83938400 |
| H | 1.86775600 | -4.54107100 | 0.55901700 |
| C | 4.00546400 | -3.49658700 | -1.87288000 |
| H | 3.70763200 | -1.75901500 | -3.11862100 |
| H | 4.02142500 | -5.15847500 | -0.49523000 |
| H | 4.95365600 | -3.76416700 | -2.33143400 |
| P | -0.66914900 | 0.47923900 | 1.87294500 |
| O | -0.78340400 | 2.12716700 | 1.93674000 |
| O | -0.17010500 | 0.10773400 | 3.42910400 |
| C | -2.41049900 | -0.05793800 | 2.06291100 |
| C | -1.30142800 | 2.79628100 | 3.09553000 |
| C | 1.20662400 | 0.35804700 | 3.73877400 |
| C | -3.43254300 | 0.74704100 | 1.53445400 |
| C | -2.72612200 | -1.32782700 | 2.56871400 |
| H | -1.42631600 | 3.84434400 | 2.81425800 |
| H | -2.26917900 | 2.38412300 | 3.40071400 |
| H | -0.60213600 | 2.72350300 | 3.93474200 |
| H | 1.86859900 | -0.03604900 | 2.95974300 |
| H | 1.39587700 | 1.43233400 | 3.84874800 |
| H | 1.41786400 | -0.14436400 | 4.68582400 |
| C | -4.75249000 | 0.29485900 | 1.53033300 |
| H | -3.18933000 | 1.71586800 | 1.10873700 |
| C | -4.04639900 | -1.78095300 | 2.55223100 |
| H | -1.94131100 | -1.96998500 | 2.95483200 |
| C | -5.06212500 | -0.97077700 | 2.03568500 |
| H | -5.53631200 | 0.92653000 | 1.12089200 |
| H | -4.28213900 | -2.76770000 | 2.94188400 |
| H | -6.08873600 | -1.32669000 | 2.02287700 |
| P | -1.16706900 | 0.12354300 | -1.86892800 |
| O | -2.36439200 | -0.93491500 | -2.30490300 |
| O | -0.32611500 | 0.11624400 | -3.28226800 |
| C | -2.11322200 | 1.70842300 | -1.86952800 |
| C | -3.38250100 | -1.26891600 | -1.35171200 |
| C | -0.94750500 | 0.39330200 | -4.54747600 |
| C | -1.66101000 | 2.78452000 | -1.08948600 |
| C | -3.31802300 | 1.83319700 | -2.58250300 |
| H | -3.86892000 | -2.17542700 | -1.71804800 |
| H | -2.96107000 | -1.46591100 | -0.36395300 |
| H | -4.11691100 | -0.45996800 | -1.27519600 |
| H | -0.16187400 | 0.30961800 | -5.30137800 |
| H | -1.74006000 | -0.33152500 | -4.75783900 |
| H | -1.36273500 | 1.40703400 | -4.56469900 |
| C | -2.40714800 | 3.96308800 | -1.02432500 |
| H | -0.73261400 | 2.67133700 | -0.53997300 |
| C | -4.06031800 | 3.01334500 | -2.51396500 |
| H | -3.67700900 | 1.00179200 | -3.18168900 |
| C | -3.60725600 | 4.07940600 | -1.73118600 |
| H | -2.05209400 | 4.79018400 | -0.41456900 |
| H | -4.99229800 | 3.09930100 | -3.06671400 |
| H | -4.18956100 | 4.99543900 | -1.67179700 |
| C | 5.91403600 | 3.26248700 | 1.26130900 |
| H | 6.41067200 | 4.08282500 | 0.72780700 |
| H | 5.33554200 | 3.69676000 | 2.08535900 |
| H | 6.69334100 | 2.62084700 | 1.69251200 |
| C | 5.00527900 | 2.45194000 | 0.31481100 |
| C | 4.35904600 | 1.28000800 | 1.09610800 |
| C | 3.92437800 | 3.36175400 | -0.24381900 |
| C | 3.12528600 | 0.72044800 | 0.38367900 |
| H | 4.05621600 | 1.64306100 | 2.08977800 |
| H | 5.10942900 | 0.49600200 | 1.26518700 |
| C | 2.61863000 | 3.07495900 | -0.25971400 |
| H | 4.27573000 | 4.30723400 | -0.66133400 |
| H | 2.71974200 | -0.15835900 | 0.90231600 |
| H | 3.37445000 | 0.38645100 | -0.63043200 |
| H | 1.90022100 | 3.78025400 | -0.67640800 |
| C | 5.86352100 | 1.91498400 | -0.85412100 |
| H | 6.27484000 | 2.74064800 | -1.44775100 |
| H | 6.70466800 | 1.31962800 | -0.47574800 |
| H | 5.27122400 | 1.28506100 | -1.52608600 |
| O | 0.97227300 | 1.38145600 | -0.56880300 |
| C | 2.02424000 | 1.78641900 | 0.26858900 |
| H | 1.63679900 | 1.98929100 | 1.28611300 |

## V’’

E = -4593.671826
$G_{Corr}$= 0.834365

| | | | |
|---|---|---|---|
| Co | -0.56723100 | 0.08624200 | 0.03819000 |
| P | -0.48325000 | -2.05200200 | 0.50732300 |
| O | -1.87350000 | -2.91124900 | 0.39590200 |
| O | -0.11131000 | -2.41919900 | 2.08409000 |
| C | 0.73883800 | -3.09844500 | -0.38750200 |
| C | -1.99656500 | -4.26340900 | 0.85650700 |
| C | 1.01394100 | -1.77216800 | 2.69327000 |
| C | 1.01866400 | -2.85505400 | -1.74049000 |
| C | 1.39889000 | -4.14991500 | 0.26803400 |
| H | -3.01234200 | -4.57681000 | 0.60745100 |
| H | -1.27534700 | -4.91923900 | 0.35572700 |
| H | -1.85272600 | -4.31258200 | 1.93922500 |
| H | 0.76242800 | -1.60497000 | 3.74283600 |
| H | 1.90084600 | -2.40792900 | 2.60984600 |
| H | 1.23014600 | -0.81676200 | 2.20758600 |
| C | 1.94355100 | -3.64785900 | -2.42238100 |
| H | 0.53830900 | -2.03491100 | -2.26026100 |
| C | 2.32407100 | -4.94144800 | -0.41480000 |
| H | 1.19195000 | -4.34118700 | 1.31602200 |
| C | 2.59855700 | -4.69126100 | -1.76218500 |
| H | 2.15897100 | -3.44316700 | -3.46750500 |
| H | 2.83048900 | -5.75103200 | 0.10418600 |
| H | 3.32231500 | -5.30343800 | -2.29361600 |
| P | -1.64721500 | 0.56666600 | 1.83791200 |
| O | -2.30709800 | 2.07938000 | 1.68487000 |
| O | -1.16887300 | 0.54819700 | 3.44322000 |
| C | -3.13557400 | -0.47760000 | 2.06828200 |
| C | -3.20298600 | 2.60552500 | 2.67345400 |
| C | -0.02778600 | 1.32915000 | 3.80741700 |
| C | -4.20702300 | -0.28320200 | 1.18270700 |
| C | -3.19102400 | -1.52117700 | 3.00024700 |
| H | -3.37547400 | 3.65107900 | 2.40857700 |
| H | -4.15518900 | 2.06374600 | 2.66696000 |
| H | -2.76570900 | 2.54700200 | 3.67556200 |
| H | 0.80014500 | 1.18698300 | 3.10532000 |
| H | -0.27779100 | 2.39659100 | 3.84096100 |
| H | 0.28094400 | 0.99981600 | 4.80276400 |
| C | -5.32302200 | -1.11857400 | 1.23452000 |
| H | -4.15924800 | 0.51833700 | 0.44937400 |
| C | -4.30623000 | -2.36024500 | 3.04541000 |
| H | -2.35693300 | -1.68375800 | 3.67364800 |
| C | -5.37198900 | -2.16271800 | 2.16343500 |
| H | -6.15038900 | -0.95874900 | 0.54805700 |
| H | -4.34224200 | -3.17064700 | 3.76897300 |
| H | -6.23801000 | -2.81839500 | 2.19984300 |
| P | -1.36547600 | -0.04159700 | -2.01527000 |
| O | -2.12919300 | -1.39653600 | -2.61326400 |
| O | -0.21097200 | 0.07921600 | -3.18368000 |
| C | -2.62172300 | 1.24787500 | -2.43067300 |
| C | -3.37504400 | -1.81191900 | -2.03383200 |
| C | -0.50055200 | 0.12830700 | -4.58761400 |

| | | | |
|---|---|---|---|
| C | -2.79108500 | 2.35750400 | -1.58807600 |
| C | -3.45928700 | 1.10554700 | -3.55130200 |
| H | -3.51678900 | -2.85867900 | -2.31347500 |
| H | -3.35737800 | -1.73860000 | -0.94510000 |
| H | -4.20185700 | -1.21193200 | -2.43174900 |
| H | 0.46401600 | 0.10445800 | -5.09956200 |
| H | -1.10145500 | -0.73377500 | -4.89443100 |
| H | -1.02543400 | 1.05441200 | -4.84577200 |
| C | -3.77104200 | 3.31160300 | -1.87178200 |
| H | -2.14726000 | 2.45832300 | -0.72368900 |
| C | -4.43402300 | 2.06352800 | -3.83405000 |
| H | -3.35687700 | 0.23680800 | -4.19456600 |
| C | -4.59108800 | 3.16979200 | -2.99386100 |
| H | -3.89349200 | 4.16684900 | -1.21193100 |
| H | -5.07355200 | 1.94362700 | -4.70469700 |
| H | -5.35379400 | 3.91322400 | -3.21105500 |
| C | 3.02788200 | 6.01924800 | 1.46757300 |
| H | 3.16682100 | 6.91829400 | 0.85383600 |
| H | 2.17844000 | 6.19587400 | 2.13810200 |
| H | 3.92747600 | 5.88979500 | 2.08331200 |
| C | 2.78810500 | 4.77573800 | 0.58774300 |
| C | 2.63984700 | 3.52407800 | 1.48823100 |
| C | 1.52207700 | 4.97528900 | -0.22923400 |
| C | 1.97049900 | 2.36476700 | 0.74732700 |
| H | 2.02500500 | 3.78560400 | 2.36252400 |
| H | 3.62613000 | 3.23600600 | 1.87727800 |
| C | 0.52916500 | 4.08465800 | -0.32583300 |
| H | 1.45447000 | 5.92302800 | -0.76689300 |
| H | 1.95917100 | 1.44618800 | 1.34394100 |
| H | 2.51960600 | 2.12922500 | -0.17185700 |
| H | -0.35151000 | 4.29905500 | -0.93027800 |
| C | 3.99034300 | 4.60907100 | -0.37098600 |
| H | 4.06494300 | 5.46244200 | -1.05613900 |
| H | 4.92991900 | 4.54752000 | 0.19397400 |
| H | 3.89509800 | 3.70277400 | -0.97827800 |
| O | -0.03374000 | 1.77613200 | -0.50794300 |
| C | 0.53411900 | 2.72776700 | 0.34983700 |
| H | -0.06288900 | 2.81466700 | 1.27562200 |
| C | 5.12950500 | -0.74187900 | -0.75702400 |
| C | 4.47416600 | -0.99762000 | 0.65579100 |
| O | 4.05914400 | -0.07359900 | -1.48843800 |
| O | 3.05058000 | -1.00160200 | 0.34772800 |
| C | 6.34914700 | 0.17548400 | -0.74538600 |
| H | 7.15290500 | -0.25765300 | -0.13963300 |
| H | 6.72152400 | 0.30242500 | -1.76690500 |
| H | 6.10438600 | 1.16399700 | -0.35021900 |
| C | 5.43841100 | -2.03201500 | -1.52512900 |
| H | 5.71068900 | -1.77424200 | -2.55339400 |
| H | 6.27159700 | -2.57917100 | -1.07224400 |
| H | 4.56354000 | -2.68710100 | -1.55974200 |
| C | 4.83011000 | -2.33755500 | 1.29497700 |
| H | 5.91205300 | -2.41897600 | 1.44689600 |
| H | 4.34301900 | -2.41921300 | 2.27229400 |
| H | 4.49138000 | -3.17361300 | 0.67972000 |
| C | 4.70741600 | 0.14484400 | 1.65026300 |
| H | 4.06439700 | -0.00756100 | 2.52302900 |
| H | 5.74875300 | 0.17584900 | 1.98647700 |
| H | 4.45125300 | 1.11087600 | 1.20895400 |
| B | 2.88116300 | -0.37012400 | -0.85650000 |
| H | 1.82123600 | -0.09490500 | -1.30120800 |

## TS III-IVb

E = -4181.589413

$G_{Corr}$= 0.656294

| | | | |
|---|---|---|---|
| Co | 0.37698700 | 0.43898700 | -0.02501100 |
| H | 1.06002000 | 1.71555900 | 0.43536400 |
| P | -0.28511900 | -1.50584500 | -0.81077300 |
| O | 0.84561300 | -2.63095100 | -0.39032000 |
| O | -0.27497900 | -1.78454100 | -2.44420300 |
| C | -1.83756700 | -2.38934700 | -0.32994200 |
| C | 0.85937400 | -3.98571300 | -0.85366300 |
| C | -1.26513900 | -1.21570200 | -3.32290700 |
| C | -2.43600700 | -2.13851700 | 0.91182200 |
| C | -2.38473800 | -3.39097800 | -1.15076900 |
| H | 1.80211500 | -4.41112900 | -0.50284100 |
| H | 0.01923800 | -4.55129700 | -0.43528900 |
| H | 0.82012500 | -4.02389400 | -1.94583900 |
| H | -1.11644300 | -0.14060400 | -3.44658400 |
| H | -1.14371100 | -1.72572900 | -4.28214000 |
| H | -2.27848500 | -1.40081200 | -2.94827800 |
| C | -3.56449700 | -2.85193600 | 1.31770600 |
| H | -2.03645900 | -1.37136700 | 1.56234800 |
| C | -3.51381300 | -4.10484000 | -0.74705800 |
| H | -1.92280200 | -3.61375600 | -2.10701700 |
| C | -4.10873300 | -3.83401100 | 0.48784700 |
| H | -4.02339500 | -2.62980900 | 2.27693500 |
| H | -3.92773100 | -4.87172200 | -1.39636900 |
| H | -4.99157900 | -4.38524300 | 0.80032500 |
| P | -1.31777000 | 1.31606300 | 0.96717300 |
| O | -1.34747300 | 2.98716600 | 1.03691900 |
| O | -1.51092400 | 0.97377200 | 2.60101800 |
| C | -3.04498300 | 0.92913200 | 0.46604700 |
| C | -2.41761600 | 3.69657800 | 1.67168900 |
| C | -0.45154700 | 1.40498900 | 3.46504100 |
| C | -3.27523900 | 0.75401500 | -0.90601100 |
| C | -4.11173900 | 0.78906900 | 1.36575000 |
| H | -2.11618900 | 4.74645500 | 1.70676200 |
| H | -3.34670300 | 3.60045300 | 1.09928000 |
| H | -2.58752300 | 3.32994000 | 2.68976500 |
| H | 0.53185300 | 1.20585100 | 3.02407100 |
| H | -0.53499100 | 2.47929400 | 3.66857300 |
| H | -0.55411400 | 0.84951700 | 4.40064200 |
| C | -4.55148300 | 0.43493100 | -1.37252900 |
| H | -2.45531000 | 0.86653100 | -1.60860100 |
| C | -5.38478200 | 0.45899800 | 0.89880600 |
| H | -3.93441900 | 0.91363000 | 2.42895600 |
| C | -5.60632300 | 0.27840200 | -0.46993400 |
| H | -4.71784700 | 0.30012100 | -2.43796200 |
| H | -6.20418500 | 0.33800600 | 1.60279900 |
| H | -6.59753600 | 0.01596400 | -0.83025400 |
| P | 2.01336700 | -0.44048700 | 1.12276300 |
| O | 1.82117400 | -1.51405000 | 2.37133700 |
| O | 2.81588600 | 0.78943800 | 1.90599600 |
| C | 3.39482300 | -1.32257400 | 0.26852500 |
| C | 0.52068300 | -1.94190400 | 2.78222400 |
| C | 3.91835100 | 0.55937000 | 2.78996200 |
| C | 3.48780400 | -1.22086600 | -1.12636800 |
| C | 4.33081300 | -2.11384200 | 0.95250400 |
| H | -0.20957400 | -1.13454200 | 2.69892400 |
| H | 0.19143400 | -2.78604400 | 2.16860800 |
| H | 0.60199800 | -2.25651400 | 3.82645500 |
| H | 4.13031100 | 1.51600200 | 3.27356000 |
| H | 3.66369300 | -0.18563900 | 3.55122500 |
| H | 4.80556000 | 0.22854200 | 2.23832100 |
| C | 4.51077500 | -1.86471600 | -1.82358300 |
| H | 2.72614700 | -0.67078800 | -1.66960400 |
| C | 5.35486500 | -2.76057200 | 0.25665100 |
| H | 4.24217600 | -2.24630900 | 2.02578700 |
| C | 5.45206800 | -2.63106900 | -1.13146300 |
| H | 4.56392500 | -1.77984100 | -2.90575900 |
| H | 6.07251800 | -3.37165600 | 0.79806000 |
| H | 6.24810400 | -3.13660600 | -1.67169100 |
| C | 3.50673000 | 3.64439000 | -1.26950200 |
| H | 4.17746300 | 3.12586600 | -0.57349600 |
| H | 3.70575400 | 3.26874500 | -2.28056000 |
| H | 3.75609500 | 4.71226800 | -1.25035900 |
| C | 2.02872600 | 3.43236300 | -0.87234600 |
| C | 1.13499900 | 4.26738600 | -1.82076100 |
| C | 1.74126100 | 1.92042500 | -1.08319600 |
| C | -0.30200800 | 3.73901900 | -1.90414700 |
| H | 1.57131500 | 4.25039200 | -2.82933900 |

| | | | |
|---|---|---|---|
| H | 1.14053500 | 5.31520800 | -1.49528400 |
| C | 0.75488500 | 1.43679900 | -1.97847500 |
| H | 2.65272500 | 1.33540800 | -1.02047400 |
| C | -0.31234700 | 2.32003200 | -2.44971400 |
| H | -0.92218200 | 4.35430600 | -2.56377900 |
| H | -0.76299800 | 3.73488700 | -0.91169400 |
| H | 0.93825900 | 0.53994100 | -2.56291500 |
| C | 1.85209400 | 3.89433400 | 0.58682600 |
| H | 2.43296900 | 3.25752300 | 1.26215600 |
| H | 2.20150100 | 4.92922100 | 0.69332500 |
| H | 0.80521300 | 3.84436900 | 0.89516500 |
| O | -1.16064700 | 1.97401600 | -3.28294800 |

# Vb

E = -4593.690894

$G_{Corr}$= 0.836697

| | | | |
|---|---|---|---|
| Co | -0.28231000 | -0.42324800 | 0.36087900 |
| H | 0.74129400 | -2.41495800 | 2.73261500 |
| P | -1.14310800 | 0.45785400 | -1.44626400 |
| O | -0.46776800 | -0.08910000 | -2.84905600 |
| O | -0.84462900 | 2.05920600 | -1.63586500 |
| C | -2.93946600 | 0.39948600 | -1.84108300 |
| C | -0.72222000 | 0.56332700 | -4.10378600 |
| C | -0.88667900 | 2.95886300 | -0.51369000 |
| C | -3.63937800 | -0.81019600 | -1.72179800 |
| C | -3.63303300 | 1.55646000 | -2.22826800 |
| H | -0.21371800 | -0.03169400 | -4.86562000 |
| H | -1.79640500 | 0.59394900 | -4.31927200 |
| H | -0.31963200 | 1.57974600 | -4.09522300 |
| H | -0.36187100 | 2.52346900 | 0.33658700 |
| H | -0.36987700 | 3.86172800 | -0.83759000 |
| H | -1.92442700 | 3.18504200 | -0.24705000 |
| C | -5.01245000 | -0.85613300 | -1.96405300 |
| H | -3.10426800 | -1.70713800 | -1.43672600 |
| C | -5.00708600 | 1.50919600 | -2.46987600 |
| H | -3.09579500 | 2.49312300 | -2.32969000 |
| C | -5.70100300 | 0.30460200 | -2.32967300 |
| H | -5.54734000 | -1.79664400 | -1.86016900 |
| H | -5.53583700 | 2.41302600 | -2.76099200 |
| H | -6.77257300 | 0.26931600 | -2.50793800 |
| P | -2.04230000 | -0.30272900 | 1.62701300 |
| O | -1.74317800 | -0.67885300 | 3.21870800 |
| O | -3.41453300 | -1.20033700 | 1.33245000 |
| C | -2.91343700 | 1.30803500 | 1.78794800 |
| C | -2.80203800 | -0.66757800 | 4.18625900 |
| C | -3.28144400 | -2.61980800 | 1.21654800 |
| C | -2.29465100 | 2.31311900 | 2.55121500 |
| C | -4.08042700 | 1.61425600 | 1.07459300 |
| H | -2.38216600 | -1.07686700 | 5.10820600 |
| H | -3.15791200 | 0.35281900 | 4.36787000 |
| H | -3.64446300 | -1.28600900 | 3.85981300 |
| H | -2.84387000 | -3.05166700 | 2.12466700 |
| H | -4.29115200 | -3.01496000 | 1.07854200 |
| H | -2.66201100 | -2.89111700 | 0.35927900 |
| C | -2.83034700 | 3.59913700 | 2.59821700 |
| H | -1.39002100 | 2.08559700 | 3.10998400 |
| C | -4.60876300 | 2.90740400 | 1.11272600 |
| H | -4.56897200 | 0.84707200 | 0.48505800 |
| C | -3.98671500 | 3.90198800 | 1.87044800 |
| H | -2.34604600 | 4.36609000 | 3.19699500 |
| H | -5.50669600 | 3.13473000 | 0.54411400 |
| H | -4.39908100 | 4.90713800 | 1.89672800 |
| P | 0.07995000 | -2.42125300 | -0.46409500 |
| O | -1.15785700 | -2.84745900 | -1.50296100 |
| O | 0.02073000 | -3.66385900 | 0.64835700 |
| C | 1.57717900 | -2.90476800 | -1.44723700 |
| C | -1.01685400 | -3.51149300 | -2.76118700 |
| C | -0.46692400 | -4.96696100 | 0.30911500 |
| C | 1.97185100 | -2.06581000 | -2.50442900 |
| C | 2.39792100 | -3.99200100 | -1.10878500 |
| H | -0.57263500 | -2.83711100 | -3.49961600 |
| H | -0.40420700 | -4.41595800 | -2.68534500 |
| H | -2.02804300 | -3.78177800 | -3.07753600 |
| H | -1.53347300 | -4.93089700 | 0.06400100 |
| H | 0.07383800 | -5.40581600 | -0.53717600 |
| H | -0.31843900 | -5.59486100 | 1.19102900 |
| C | 3.14006500 | -2.32777000 | -3.22085500 |
| H | 1.36961500 | -1.19736200 | -2.74873600 |
| C | 3.57047100 | -4.24937200 | -1.82282100 |
| H | 2.14040100 | -4.62581100 | -0.26737500 |
| C | 3.94262600 | -3.42147000 | -2.88409700 |
| H | 3.43142600 | -1.66689500 | -4.03307600 |
| H | 4.19596500 | -5.09339800 | -1.54381300 |
| H | 4.85784600 | -3.61911300 | -3.43573900 |
| C | 3.05038500 | -3.13101000 | 3.81634800 |
| H | 2.69296300 | -3.07637000 | 4.85262700 |
| H | 2.53240100 | -3.96453400 | 3.32520100 |
| H | 4.12200400 | -3.36719400 | 3.84210700 |
| C | 2.79157000 | -1.81038700 | 3.07416200 |
| C | 3.24858200 | -1.95815900 | 1.60593900 |
| C | 1.27083100 | -1.51627800 | 3.06834700 |
| C | 3.00246500 | -0.71021300 | 0.74538800 |
| H | 2.70605100 | -2.80529600 | 1.17570900 |
| H | 4.31466600 | -2.22090300 | 1.56852500 |
| C | 0.91118800 | -0.34481600 | 2.17541700 |
| H | 0.92465600 | -1.31649200 | 4.08945600 |
| C | 1.70370200 | -0.00281600 | 1.05778100 |
| H | 3.05514000 | -0.94271700 | -0.32104800 |
| H | 3.78040300 | 0.04400700 | 0.91598400 |
| H | 0.39725800 | 0.48888200 | 2.64120200 |
| C | 3.55853800 | -0.67537900 | 3.77792800 |
| H | 3.27237700 | -0.61321700 | 4.83546900 |
| H | 4.64142700 | -0.84763500 | 3.73065400 |
| H | 3.35357900 | 0.29959400 | 3.32257200 |
| O | 1.29703700 | 0.97779000 | 0.31474400 |
| C | 3.01784900 | 4.20897900 | -0.87334200 |
| C | 3.99605400 | 3.30975400 | -0.02125700 |
| O | 2.14570100 | 3.23389800 | -1.50436700 |
| O | 3.94486800 | 2.03853600 | -0.72635400 |
| C | 2.16305700 | 5.17577900 | -0.05692200 |
| H | 2.79488000 | 5.89004100 | 0.48300400 |
| H | 1.50599900 | 5.74088400 | -0.72655000 |
| H | 1.53775400 | 4.64486600 | 0.66355800 |
| C | 3.71961300 | 4.96053100 | -2.01205800 |
| H | 2.96001500 | 5.40673100 | -2.66172100 |
| H | 4.36688100 | 5.75868800 | -1.63405300 |
| H | 4.32243100 | 4.27637600 | -2.61736200 |
| C | 5.44841000 | 3.78254100 | 0.00275500 |
| H | 5.52513500 | 4.77607300 | 0.45874600 |
| H | 6.04872600 | 3.08686200 | 0.59825200 |
| H | 5.87662300 | 3.82346400 | -1.00129100 |
| C | 3.50355400 | 3.06594400 | 1.41130900 |
| H | 4.13773600 | 2.30591900 | 1.87969700 |
| H | 3.56444400 | 3.97921000 | 2.01259800 |
| H | 2.47643000 | 2.69703800 | 1.41159300 |
| B | 2.77655000 | 2.01355600 | -1.45448200 |
| H | 2.47111600 | 1.09429500 | -2.14146400 |

# $VI_{Add}$

E = -4593.707909

$G_{Corr}$= 0.83986

| | | | |
|---|---|---|---|
| Co | -0.30610800 | 0.24307800 | -0.06235700 |
| H | 4.02585300 | -0.82500600 | -3.77187300 |
| P | -1.97356300 | 0.88989800 | 1.09958400 |
| O | -1.66254800 | 2.30568500 | 1.87370100 |
| O | -2.38560900 | -0.01244700 | 2.40939000 |
| C | -3.60042900 | 1.16987800 | 0.30584100 |
| C | -2.60687000 | 2.88785100 | 2.78775400 |

| | | | |
|---|---|---|---|
| C | -2.43685800 | -1.44986000 | 2.33147300 |
| C | -3.62957800 | 1.75585600 | -0.96869700 |
| C | -4.80181800 | 0.77299400 | 0.91104500 |
| H | -2.17817200 | 3.84092900 | 3.10339800 |
| H | -3.57091500 | 3.06329900 | 2.29656100 |
| H | -2.74995800 | 2.23835400 | 3.65572700 |
| H | -1.52194100 | -1.85057400 | 1.88845100 |
| H | -2.53765700 | -1.80390100 | 3.36011700 |
| H | -3.30340900 | -1.77219300 | 1.74621500 |
| C | -4.84363000 | 1.94058700 | -1.62967700 |
| H | -2.70028100 | 2.05223200 | -1.44293100 |
| C | -6.01524100 | 0.95090400 | 0.24461200 |
| H | -4.77991600 | 0.31595900 | 1.89504900 |
| C | -6.03698200 | 1.53099400 | -1.02697000 |
| H | -4.85744500 | 2.39068100 | -2.61850500 |
| H | -6.94195300 | 0.63248900 | 0.71420100 |
| H | -6.98162500 | 1.66262300 | -1.54769600 |
| P | -1.38131000 | -0.99364900 | -1.57377100 |
| O | -0.32899200 | -1.97990000 | -2.38061600 |
| O | -2.25224700 | -0.30084300 | -2.81332900 |
| C | -2.68342600 | -2.16828900 | -1.04177700 |
| C | -0.78888200 | -2.84326200 | -3.42974600 |
| C | -1.61448300 | 0.73636800 | -3.56640800 |
| C | -2.26257200 | -3.33825000 | -0.38557200 |
| C | -4.05236200 | -1.88792800 | -1.14640600 |
| H | 0.10506600 | -3.24620100 | -3.91174600 |
| H | -1.38881000 | -3.66547300 | -3.02456000 |
| H | -1.38528900 | -2.29066300 | -4.16329800 |
| H | -0.96381200 | 1.34854900 | -2.93221800 |
| H | -1.01468900 | 0.30428200 | -4.37648800 |
| H | -2.40485100 | 1.35948100 | -3.99354400 |
| C | -3.20319400 | -4.22044500 | 0.14454200 |
| H | -1.20051900 | -3.52619000 | -0.25381700 |
| C | -4.99025800 | -2.76762800 | -0.59903900 |
| H | -4.38274400 | -0.98257700 | -1.64380200 |
| C | -4.56952000 | -3.93486100 | 0.04301500 |
| H | -2.87106600 | -5.12363400 | 0.64997700 |
| H | -6.04973300 | -2.53656000 | -0.67369600 |
| H | -5.30143500 | -4.61716400 | 0.46751800 |
| P | 0.39778400 | 2.25703400 | -0.52885100 |
| O | -0.55318300 | 3.49968300 | -1.12249100 |
| O | 1.39113600 | 2.12428200 | -1.83718600 |
| C | 1.40050000 | 3.15217900 | 0.73715700 |
| C | -1.34795700 | 4.38687300 | -0.32919200 |
| C | 2.16707300 | 3.20863300 | -2.36147700 |
| C | 1.78539600 | 2.42226700 | 1.87196300 |
| C | 1.79993500 | 4.49371900 | 0.61794300 |
| H | -2.32089100 | 3.94224500 | -0.10401100 |
| H | -0.85449700 | 4.64765700 | 0.61125900 |
| H | -1.49192200 | 5.29194000 | -0.92653100 |
| H | 2.60891700 | 2.84789800 | -3.29284300 |
| H | 1.53520800 | 4.07870200 | -2.56736100 |
| H | 2.96645500 | 3.48772100 | -1.66635000 |
| C | 2.57217600 | 3.01608200 | 2.86020800 |
| H | 1.47782000 | 1.38477400 | 1.96657800 |
| C | 2.58034800 | 5.08905700 | 1.61032900 |
| H | 1.49299900 | 5.07596400 | -0.24601600 |
| C | 2.96971600 | 4.34931900 | 2.73140000 |
| H | 2.87211700 | 2.43715100 | 3.72961900 |
| H | 2.88372500 | 6.12794400 | 1.51042100 |
| H | 3.57867400 | 4.81355400 | 3.50273000 |
| C | 6.46315900 | -0.33488800 | -2.99160500 |
| H | 6.82787300 | -1.21672100 | -3.53378700 |
| H | 6.12826400 | 0.40131900 | -3.73332200 |
| H | 7.31107100 | 0.09824100 | -2.44604000 |
| C | 5.32441400 | -0.71061500 | -2.03046200 |
| C | 4.77365200 | 0.56413500 | -1.35418000 |
| C | 4.17026000 | -1.36935100 | -2.82421900 |
| C | 3.62449300 | 0.27293100 | -0.38040700 |
| H | 4.41202200 | 1.24320800 | -2.13905100 |
| H | 5.58318100 | 1.08796600 | -0.82826700 |
| C | 2.86239400 | -1.40994400 | -2.07425600 |
| H | 4.46346900 | -2.38878000 | -3.11582700 |
| C | 2.62268400 | -0.66858100 | -0.98801700 |
| H | 3.12235600 | 1.20196400 | -0.10360100 |
| H | 3.99083200 | -0.15571000 | 0.55874800 |
| H | 2.06598300 | -2.04771100 | -2.44330500 |
| C | 5.86450200 | -1.69184700 | -0.97162400 |
| H | 6.32783300 | -2.56190900 | -1.45308100 |
| H | 6.62413800 | -1.20924600 | -0.34384000 |
| H | 5.06819200 | -2.05998900 | -0.31723500 |
| O | 1.38681000 | -0.77590000 | -0.37028500 |
| C | 2.18886400 | -2.18845500 | 2.83059000 |
| C | 1.58885300 | -3.33811000 | 1.94088500 |
| O | 2.20933800 | -1.08094400 | 1.92034500 |
| O | 0.65877800 | -2.62601700 | 1.11281400 |
| C | 1.27693300 | -1.81902000 | 4.01240900 |
| H | 1.27003700 | -2.59455200 | 4.78606800 |
| H | 1.64186200 | -0.88778400 | 4.45872600 |
| H | 0.25009700 | -1.65209400 | 3.67310300 |
| C | 3.61291500 | -2.43775500 | 3.32458200 |
| H | 3.94763600 | -1.59144000 | 3.93464300 |
| H | 3.66107700 | -3.34324900 | 3.94067000 |
| H | 4.30703900 | -2.54494500 | 2.48730700 |
| C | 2.64004000 | -3.98374700 | 1.02418900 |
| H | 3.35656600 | -4.59770500 | 1.58131100 |
| H | 2.12610800 | -4.62301400 | 0.29846600 |
| H | 3.18469300 | -3.21674200 | 0.46584100 |
| C | 0.83019100 | -4.41611700 | 2.71354700 |
| H | 0.45076000 | -5.17277000 | 2.01753400 |
| H | 1.48433500 | -4.91672300 | 3.43710500 |
| H | -0.02397800 | -3.99137400 | 3.24701400 |
| B | 1.12412600 | -1.26342800 | 0.99862200 |
| H | 0.11135200 | -0.46860800 | 1.36511700 |

# VII

E = -4597.707535

$G_{Corr}$= 0.644896

| | | | |
|---|---|---|---|
| Co | 0.04101200 | -0.37931200 | -0.82168000 |
| H | -0.24076700 | -0.87440300 | -2.20830000 |
| P | -1.13170600 | -2.07832500 | -0.27717000 |
| O | -0.72487300 | -3.02500800 | 1.04264200 |
| O | -1.26439700 | -3.21798100 | -1.48708100 |
| C | -2.89209500 | -1.83913500 | 0.19239300 |
| C | 0.61455900 | -3.53252800 | 1.06594800 |
| C | -1.96973400 | -4.44623200 | -1.25810800 |
| C | -3.83219000 | -1.64368800 | -0.83212000 |
| C | -3.29805200 | -1.70575200 | 1.52747500 |
| H | 1.33155900 | -2.76511500 | 0.75519100 |
| H | 0.82887400 | -3.83614000 | 2.09238900 |
| H | 0.70634300 | -4.40133700 | 0.40305100 |
| H | -1.79176600 | -5.07460700 | -2.13404600 |
| H | -1.60016700 | -4.95400200 | -0.36083300 |
| H | -3.04551000 | -4.26923400 | -1.14901300 |
| C | -5.15604300 | -1.32438600 | -0.52572800 |
| H | -3.51818200 | -1.71936600 | -1.86814900 |
| C | -4.62064100 | -1.37756500 | 1.83213700 |
| H | -2.57475400 | -1.84665900 | 2.32204700 |
| C | -5.55242200 | -1.18433600 | 0.80792900 |
| H | -5.87502100 | -1.17547500 | -1.32711200 |
| H | -4.92485000 | -1.27258300 | 2.87064500 |
| H | -6.58065000 | -0.92587900 | 1.04716600 |
| P | 2.03435500 | -0.62515000 | -1.50748400 |
| O | 2.20262100 | -0.47721100 | -3.16671600 |
| O | 2.85952900 | -2.06478400 | -1.27025200 |
| C | 3.37047600 | 0.48404700 | -0.90041000 |
| C | 3.49874500 | -0.54209500 | -3.77210900 |
| C | 2.28847000 | -3.22933400 | -1.87822000 |
| C | 3.05228500 | 1.84951000 | -0.82685700 |
| C | 4.63881500 | 0.04983500 | -0.49023800 |

| | | | |
|---|---|---|---|
| H | 3.33802400 | -0.60897500 | -4.85106400 |
| H | 4.08382800 | 0.35715100 | -3.54547100 |
| H | 4.05575800 | -1.42162800 | -3.43081200 |
| H | 1.19650300 | -3.23174500 | -1.79365500 |
| H | 2.56238200 | -3.28049600 | -2.93921100 |
| H | 2.69856200 | -4.09849100 | -1.35737900 |
| C | 3.98431600 | 2.76502500 | -0.33576400 |
| H | 2.05843000 | 2.17894800 | -1.11497500 |
| C | 5.56646500 | 0.96550200 | 0.01199200 |
| H | 4.88566800 | -1.00474500 | -0.54825900 |
| C | 5.24005200 | 2.32242800 | 0.09365000 |
| H | 3.72822400 | 3.81978700 | -0.27766500 |
| H | 6.54268800 | 0.62011500 | 0.34266500 |
| H | 5.96110500 | 3.03253700 | 0.49020700 |
| P | -1.18496000 | 1.25142600 | -1.48460000 |
| O | -2.41988600 | 0.71780000 | -2.46419300 |
| O | -0.55136000 | 2.50687100 | -2.38552800 |
| C | -2.08735400 | 2.33819700 | -0.30077500 |
| C | -3.37034800 | 1.63704200 | -3.01793200 |
| C | 0.18708100 | 2.17090800 | -3.56690400 |
| C | -3.22353800 | 1.82342700 | 0.34017700 |
| C | -1.62359800 | 3.61227500 | 0.05313900 |
| H | -3.87316300 | 1.11402800 | -3.83547200 |
| H | -4.10980000 | 1.93274200 | -2.26542800 |
| H | -2.87593300 | 2.53364500 | -3.40703800 |
| H | 0.78735600 | 3.04704200 | -3.82778000 |
| H | 0.83921200 | 1.30987900 | -3.39966200 |
| H | -0.49958600 | 1.94679300 | -4.39319500 |
| C | -3.89454200 | 2.57484500 | 1.30523300 |
| H | -3.58164300 | 0.83209200 | 0.08927800 |
| C | -2.29051600 | 4.36165500 | 1.02606000 |
| H | -0.74105100 | 4.01356200 | -0.43240300 |
| C | -3.42878300 | 3.84599100 | 1.65327500 |
| H | -4.77566600 | 2.16042500 | 1.78827500 |
| H | -1.92445100 | 5.35106700 | 1.28948700 |
| H | -3.94758200 | 4.43081000 | 2.40866000 |
| P | 0.34296400 | 0.41979700 | 1.16933200 |
| O | -1.07025200 | 0.32734400 | 2.01025900 |
| O | 0.78711000 | 2.00420300 | 1.12763700 |
| C | 1.52223400 | -0.26549200 | 2.40719200 |
| C | -1.35284100 | 1.00585900 | 3.23547700 |
| C | 1.09503100 | 2.86493200 | 2.22828200 |
| C | 2.86184800 | 0.15208600 | 2.43594500 |
| C | 1.10433600 | -1.27844200 | 3.28412300 |
| H | -2.27133700 | 0.56119900 | 3.62668400 |
| H | -0.54786300 | 0.87650700 | 3.96859900 |
| H | -1.52767900 | 2.06875000 | 3.04679000 |
| H | 0.26899500 | 3.57030700 | 2.36128700 |
| H | 1.26644500 | 2.31346300 | 3.15738200 |
| H | 2.00808800 | 3.40929200 | 1.96813500 |
| C | 3.76739700 | -0.44131100 | 3.31681500 |
| H | 3.19832000 | 0.94396100 | 1.77691200 |
| C | 2.00929500 | -1.86555700 | 4.16950900 |
| H | 0.07283200 | -1.61548200 | 3.25598000 |
| C | 3.34421400 | -1.45131000 | 4.18465400 |
| H | 4.80258600 | -0.11084100 | 3.32290300 |
| H | 1.67313700 | -2.64792700 | 4.84499200 |
| H | 4.04992000 | -1.91202500 | 4.87076000 |

# VIII

E = -4597.101945

$G_{Corr}$= 0.631905

| | | | |
|---|---|---|---|
| Co | 0.04776800 | -0.44000700 | -0.78295900 |
| P | -1.18412400 | -2.10186600 | -0.11431600 |
| O | -0.78149200 | -2.96666400 | 1.25671500 |
| O | -1.31989900 | -3.28845400 | -1.28014700 |
| C | -2.94225000 | -1.83067400 | 0.34609100 |
| C | 0.57518900 | -3.41512900 | 1.36652500 |
| C | -2.02102200 | -4.51263600 | -1.01258300 |
| C | -3.89461700 | -1.72835100 | -0.68117200 |
| C | -3.33063100 | -1.56367800 | 1.66649900 |
| H | 1.27154500 | -2.65937900 | 0.98733800 |
| H | 0.77403700 | -3.59401700 | 2.42496300 |
| H | 0.71341400 | -4.34821500 | 0.80725600 |
| H | -1.85287400 | -5.16433400 | -1.87320500 |
| H | -1.63558500 | -4.99296400 | -0.10676900 |
| H | -3.09558700 | -4.33595000 | -0.89285900 |
| C | -5.21304500 | -1.37333600 | -0.39154800 |
| H | -3.59483500 | -1.90643900 | -1.70941600 |
| C | -4.64775200 | -1.19966000 | 1.95343500 |
| H | -2.59656000 | -1.62484400 | 2.46129800 |
| C | -5.59190600 | -1.10243900 | 0.92700100 |
| H | -5.94152800 | -1.29890500 | -1.19472900 |
| H | -4.93789900 | -0.99055900 | 2.98014600 |
| H | -6.61582600 | -0.81606300 | 1.15241000 |
| P | 2.05720800 | -0.80672100 | -1.49265000 |
| O | 2.20697200 | -0.82054300 | -3.16326900 |
| O | 2.91016100 | -2.20459400 | -1.11932600 |
| C | 3.36719100 | 0.37607500 | -0.97632500 |
| C | 3.49174900 | -0.97168000 | -3.78000400 |
| C | 2.32576300 | -3.43178800 | -1.56658100 |
| C | 3.01606600 | 1.73570700 | -0.98203300 |
| C | 4.65028000 | -0.00049500 | -0.55305200 |
| H | 3.31674900 | -1.07960200 | -4.85329300 |
| H | 4.11785600 | -0.08969600 | -3.59883100 |
| H | 4.01354500 | -1.85829700 | -3.40339400 |
| H | 1.23288000 | -3.41296200 | -1.47708000 |
| H | 2.59059600 | -3.62491400 | -2.61368300 |
| H | 2.72854900 | -4.23185200 | -0.93989900 |
| C | 3.93075200 | 2.70253600 | -0.56182900 |
| H | 2.01012400 | 2.02263500 | -1.27199800 |
| C | 5.56071700 | 0.96728300 | -0.12278900 |
| H | 4.92207800 | -1.05042900 | -0.54430200 |
| C | 5.20243600 | 2.31879200 | -0.12402000 |
| H | 3.64724000 | 3.75180200 | -0.56378100 |
| H | 6.54866200 | 0.66693500 | 0.21684200 |
| H | 5.91064700 | 3.06959800 | 0.21681100 |
| P | -1.16914200 | 1.15754800 | -1.64041200 |
| O | -2.37963100 | 0.52701700 | -2.59788400 |
| O | -0.54895100 | 2.36998000 | -2.61501700 |
| C | -2.08980800 | 2.29858400 | -0.52789000 |
| C | -3.32078300 | 1.38423400 | -3.25916500 |
| C | 0.26914600 | 1.97362800 | -3.72106200 |
| C | -3.21962000 | 1.80606900 | 0.14099000 |
| C | -1.63805300 | 3.59426900 | -0.24178300 |
| H | -3.87488400 | 0.75922100 | -3.96383300 |
| H | -4.01705200 | 1.82623000 | -2.53750500 |
| H | -2.81075600 | 2.18691800 | -3.80271800 |
| H | 0.88129200 | 2.83719800 | -3.99611100 |
| H | 0.91699500 | 1.13062200 | -3.46003100 |
| H | -0.35697500 | 1.69022000 | -4.57683200 |
| C | -3.89736800 | 2.60017400 | 1.06628100 |
| H | -3.56628900 | 0.79880900 | -0.05587300 |
| C | -2.31169000 | 4.38628400 | 0.69136700 |
| H | -0.75929800 | 3.97827500 | -0.74755400 |
| C | -3.44401400 | 3.89225300 | 1.34645300 |
| H | -4.77268300 | 2.20156400 | 1.57238600 |
| H | -1.95512000 | 5.39147600 | 0.90290300 |
| H | -3.96744300 | 4.51017600 | 2.07174300 |
| P | 0.34954400 | 0.54062200 | 1.19927200 |
| O | -1.06054400 | 0.49995400 | 2.06098300 |
| O | 0.77340800 | 2.12713000 | 1.02666600 |
| C | 1.54841600 | -0.02043300 | 2.48467400 |
| C | -1.34325500 | 1.27754200 | 3.22557800 |
| C | 1.07951900 | 3.07477400 | 2.05290300 |
| C | 2.89295500 | 0.38093200 | 2.43280100 |
| C | 1.15591100 | -0.95934900 | 3.45139500 |
| H | -2.24552200 | 0.85064600 | 3.67164300 |
| H | -0.52556100 | 1.23405200 | 3.95506300 |
| H | -1.54581000 | 2.31545100 | 2.94586800 |

| | | | |
|---|---|---|---|
| H | 0.23919600 | 3.76898700 | 2.15434200 |
| H | 1.28491100 | 2.59723100 | 3.01581300 |
| H | 1.97234700 | 3.62265400 | 1.73512700 |
| C | 3.82397000 | -0.14899300 | 3.32734200 |
| H | 3.21367300 | 1.10963800 | 1.69650100 |
| C | 2.08652900 | -1.48400900 | 4.34974700 |
| H | 0.12101500 | -1.28683500 | 3.48691000 |
| C | 3.42398700 | -1.08235600 | 4.28764100 |
| H | 4.86163900 | 0.16920300 | 3.27113900 |
| H | 1.76851800 | -2.20766800 | 5.09596200 |
| H | 4.14979500 | -1.49456500 | 4.98362100 |

## IX

E = -5009.173919
$G_{Corr}$= 0.812113

| | | | |
|---|---|---|---|
| Co | 0.06380800 | -0.12957100 | 0.14753900 |
| P | 0.20305200 | 1.01468000 | 1.99705200 |
| O | 1.38253300 | 0.61855300 | 3.11394900 |
| O | -1.18942400 | 0.99109700 | 2.91857000 |
| C | 0.56364000 | 2.81784700 | 1.96560800 |
| C | 1.45573100 | -0.76049800 | 3.50095600 |
| C | -1.23237100 | 1.60016300 | 4.21623100 |
| C | -0.49917100 | 3.69912500 | 1.70851600 |
| C | 1.86455500 | 3.32952800 | 2.06289300 |
| H | 1.30037200 | -1.41823100 | 2.63893800 |
| H | 2.45172000 | -0.92889400 | 3.91427300 |
| H | 0.69885800 | -0.98260700 | 4.26326000 |
| H | -2.17920300 | 1.29897500 | 4.67130100 |
| H | -0.39976400 | 1.25658700 | 4.83947100 |
| H | -1.19406600 | 2.69259400 | 4.14204100 |
| C | -0.26519700 | 5.06712000 | 1.55968500 |
| H | -1.50574000 | 3.30631100 | 1.60464000 |
| C | 2.09941600 | 4.69643900 | 1.90187300 |
| H | 2.69107400 | 2.65560500 | 2.25498700 |
| C | 1.03689600 | 5.56893800 | 1.64952900 |
| H | -1.09675200 | 5.73946500 | 1.36458800 |
| H | 3.11343600 | 5.08170700 | 1.97585900 |
| H | 1.22168300 | 6.63274400 | 1.52483600 |
| P | -0.43559000 | -2.23665000 | 0.21268300 |
| O | -1.89557900 | -2.67533800 | -0.50239800 |
| O | -0.59265000 | -3.05635300 | 1.66790300 |
| C | 0.71066000 | -3.44177000 | -0.57594900 |
| C | -2.34446800 | -4.03452600 | -0.43959000 |
| C | -1.51686000 | -2.48845300 | 2.60563800 |
| C | 1.04989500 | -3.20077600 | -1.91731400 |
| C | 1.25562300 | -4.55552800 | 0.07797000 |
| H | -3.18902500 | -4.12256400 | -1.12602100 |
| H | -1.55644200 | -4.73149400 | -0.74739900 |
| H | -2.67211000 | -4.29373900 | 0.57290400 |
| H | -1.31209700 | -1.42589300 | 2.77098700 |
| H | -2.54915000 | -2.58739400 | 2.24916800 |
| H | -1.39649000 | -3.03911300 | 3.54252100 |
| C | 1.92047500 | -4.05736600 | -2.59196300 |
| H | 0.65726900 | -2.32054200 | -2.41669000 |
| C | 2.13430200 | -5.40813000 | -0.59567400 |
| H | 0.99314700 | -4.74734500 | 1.11267900 |
| C | 2.46857000 | -5.16154400 | -1.92982900 |
| H | 2.17832900 | -3.85949500 | -3.62917300 |
| H | 2.55863300 | -6.26447900 | -0.07783900 |
| H | 3.15433100 | -5.82421600 | -2.45131800 |
| P | -0.52924600 | 1.20530200 | -1.48811200 |
| O | -1.67801100 | 2.26589800 | -0.92667500 |
| O | -1.16235200 | 0.69719900 | -2.94734900 |
| C | 0.69138900 | 2.35050800 | -2.25984000 |
| C | -2.44309800 | 3.07594800 | -1.82331900 |
| C | -2.05161900 | -0.42581500 | -2.96860800 |
| C | 0.98388800 | 3.56971800 | -1.63360700 |
| C | 1.41982800 | 1.97665000 | -3.39800800 |
| H | -2.94220700 | 3.83255800 | -1.21148600 |
| H | -1.80445000 | 3.57357600 | -2.56280400 |
| H | -3.19677000 | 2.46945500 | -2.33451500 |
| H | -2.06422600 | -0.79981500 | -3.99694400 |
| H | -1.71232800 | -1.21272200 | -2.28939400 |
| H | -3.06004400 | -0.12215500 | -2.67100700 |
| C | 1.97686800 | 4.40745600 | -2.14316100 |
| H | 0.44036500 | 3.86061900 | -0.74243800 |
| C | 2.41545200 | 2.81403800 | -3.90709900 |
| H | 1.20575600 | 1.03042500 | -3.88262400 |
| C | 2.69580100 | 4.03298700 | -3.28149300 |
| H | 2.19001300 | 5.34940200 | -1.64416100 |
| H | 2.96772200 | 2.51780100 | -4.79581800 |
| H | 3.47123300 | 4.68392600 | -3.67764700 |
| P | 2.26179100 | -0.11174000 | -0.32153300 |
| O | 2.83087800 | 1.42236500 | -0.10581000 |
| O | 2.55299900 | -0.52852300 | -1.89537600 |
| C | 3.56949200 | -1.07209000 | 0.56311300 |
| C | 4.10278000 | 1.92323700 | -0.51988300 |
| C | 3.81754800 | -0.57632500 | -2.56231200 |
| C | 3.89914000 | -2.37801700 | 0.16979100 |
| C | 4.16443800 | -0.54225300 | 1.71965500 |
| H | 4.25578200 | 2.86063400 | 0.02161800 |
| H | 4.91606700 | 1.22893500 | -0.27724100 |
| H | 4.09146400 | 2.13328100 | -1.59281700 |
| H | 3.92953000 | 0.32081600 | -3.17986000 |
| H | 4.65602600 | -0.65042100 | -1.86287300 |
| H | 3.81462700 | -1.46448400 | -3.20196600 |
| C | 4.80156100 | -3.13741000 | 0.91646000 |
| H | 3.45395500 | -2.80290700 | -0.72218200 |
| C | 5.07119200 | -1.29950000 | 2.46258600 |
| H | 3.89950300 | 0.45837500 | 2.04594300 |
| C | 5.39054200 | -2.60101100 | 2.06412500 |
| H | 5.04000000 | -4.14894800 | 0.59861500 |
| H | 5.52629200 | -0.87478000 | 3.35379400 |
| H | 6.09305500 | -3.19252700 | 2.64534300 |
| C | -5.60750100 | 0.22585000 | -0.49226600 |
| C | -5.63458500 | -1.09568800 | 0.36049800 |
| O | -4.18183300 | 0.51643500 | -0.56584600 |
| O | -4.42185200 | -0.96990000 | 1.16311600 |
| C | -6.15078200 | 0.09359200 | -1.91176000 |
| H | -7.20725400 | -0.19640700 | -1.89556600 |
| H | -6.06833300 | 1.05620400 | -2.42686000 |
| H | -5.59295500 | -0.64721400 | -2.48865300 |
| C | -6.25703900 | 1.41960300 | 0.21771400 |
| H | -6.01669900 | 2.33303900 | -0.33502700 |
| H | -7.34589600 | 1.31645300 | 0.26485200 |
| H | -5.86988500 | 1.52711200 | 1.23583700 |
| C | -6.82808300 | -1.23771900 | 1.29902600 |
| H | -7.76473800 | -1.25426800 | 0.73060600 |
| H | -6.74919800 | -2.17771900 | 1.85470000 |
| H | -6.87083400 | -0.41952900 | 2.02138500 |
| C | -5.46461600 | -2.35788300 | -0.49065800 |
| H | -5.28451900 | -3.21027500 | 0.17098300 |
| H | -6.35798100 | -2.56258900 | -1.08930000 |
| H | -4.60229800 | -2.25956800 | -1.15549100 |
| B | -3.58216900 | -0.11901200 | 0.49123600 |
| H | -2.46429800 | 0.07519400 | 0.82001100 |

## TS IX-X$^{BS}$

E = -5396.62933
$G_{Corr}$= 0.98479

| | | | |
|---|---|---|---|
| Co | -0.75868500 | -0.25524000 | 0.09777500 |
| H | 1.55080500 | -0.94600900 | 0.43493900 |
| P | -0.07393600 | 1.20496300 | -1.44606700 |
| O | -1.08238300 | 1.40824900 | -2.75903000 |
| O | 1.39000500 | 0.93088500 | -2.17393200 |
| C | 0.08377500 | 2.97439000 | -0.99893500 |

| | | | |
|---|---|---|---|
| C | -1.36267400 | 0.21773500 | -3.51065300 |
| C | 1.87901800 | 1.82819900 | -3.18546600 |
| C | 1.26313300 | 3.36817000 | -0.34653500 |
| C | -0.93570400 | 3.91251700 | -1.20609400 |
| H | -1.63942300 | -0.60793700 | -2.84583800 |
| H | -2.19924800 | 0.44625500 | -4.17245500 |
| H | -0.48812500 | -0.07644100 | -4.10300200 |
| H | 2.65063300 | 2.47318200 | -2.75603100 |
| H | 2.31027800 | 1.21399100 | -3.98065200 |
| H | 1.07540200 | 2.44002500 | -3.60518600 |
| C | 1.42446300 | 4.68695400 | 0.07897100 |
| H | 2.04121400 | 2.63632600 | -0.15886200 |
| C | -0.77542400 | 5.22949900 | -0.77057000 |
| H | -1.84983600 | 3.61013100 | -1.70322500 |
| C | 0.40379600 | 5.62033200 | -0.12960000 |
| H | 2.34307500 | 4.98377000 | 0.57834700 |
| H | -1.57071300 | 5.95228600 | -0.93419700 |
| H | 0.52639500 | 6.64642000 | 0.20705900 |
| P | -0.78122600 | -2.42251000 | -0.52411800 |
| O | -0.04505800 | -3.44658400 | 0.55657600 |
| O | -0.12904100 | -2.90114100 | -1.97049100 |
| C | -2.40466600 | -3.25987900 | -0.73775200 |
| C | 0.13632800 | -4.83423100 | 0.23708400 |
| C | 1.20931900 | -2.45487700 | -2.27559200 |
| C | -3.17270200 | -3.48575900 | 0.41663800 |
| C | -2.90303400 | -3.66917800 | -1.98152900 |
| H | 0.58274800 | -5.29992100 | 1.11835100 |
| H | -0.82108900 | -5.31949500 | 0.01354400 |
| H | 0.80975800 | -4.95129000 | -0.61761500 |
| H | 1.24388200 | -1.36675700 | -2.37305300 |
| H | 1.91348700 | -2.76373500 | -1.49718900 |
| H | 1.47334500 | -2.92165100 | -3.22834200 |
| C | -4.41173600 | -4.12120000 | 0.32884100 |
| H | -2.79796000 | -3.16512900 | 1.38427000 |
| C | -4.14692600 | -4.29973200 | -2.06908900 |
| H | -2.31059500 | -3.50432400 | -2.87526900 |
| C | -4.90150800 | -4.52990400 | -0.91655600 |
| H | -4.99459200 | -4.29730600 | 1.22907000 |
| H | -4.52539800 | -4.61237900 | -3.03864100 |
| H | -5.86720300 | -5.02345900 | -0.98653000 |
| P | -0.38138300 | 0.74344400 | 2.11501600 |
| O | 1.10538900 | 1.42858300 | 2.10452300 |
| O | -0.43292000 | -0.22448000 | 3.45547500 |
| C | -1.41593400 | 2.09232900 | 2.81780200 |
| C | 1.83164200 | 1.70280000 | 3.31007500 |
| C | -0.39001300 | -1.64870400 | 3.25537000 |
| C | -1.14628400 | 3.42618700 | 2.48388000 |
| C | -2.53617600 | 1.79721000 | 3.60805400 |
| H | 2.27172300 | 0.77388600 | 3.67891500 |
| H | 2.62499500 | 2.40285600 | 3.03622900 |
| H | 1.18874000 | 2.15977100 | 4.07199200 |
| H | -1.36330400 | -2.00519900 | 2.89912900 |
| H | 0.38277000 | -1.92497000 | 2.54164900 |
| H | -0.17266600 | -2.08911700 | 4.23176100 |
| C | -1.97523400 | 4.45037100 | 2.94424200 |
| H | -0.29041000 | 3.66294700 | 1.86250800 |
| C | -3.36439000 | 2.82282700 | 4.07059400 |
| H | -2.75048600 | 0.76592600 | 3.86816600 |
| C | -3.08456400 | 4.15281800 | 3.74051600 |
| H | -1.75227300 | 5.48032500 | 2.67778200 |
| H | -4.22269100 | 2.58475900 | 4.69423300 |
| H | -3.72894700 | 4.95089200 | 4.10034500 |
| P | -2.97897000 | 0.40801300 | -0.00902900 |
| O | -2.98378900 | 2.04524600 | 0.14361400 |
| O | -3.77873700 | -0.23819200 | 1.28474500 |
| C | -4.20588600 | 0.19161200 | -1.36880000 |
| C | -4.11524700 | 2.89070200 | 0.37759900 |
| C | -5.11637600 | 0.07991800 | 1.69508100 |
| C | -4.98767200 | -0.96950200 | -1.45141900 |
| C | -4.28432500 | 1.13896900 | -2.40366100 |
| H | -3.83788600 | 3.88540500 | 0.01945500 |
| H | -5.00410600 | 2.54626300 | -0.16224100 |
| H | -4.31735800 | 2.94260000 | 1.44988200 |
| H | -5.08824600 | 0.89497400 | 2.42490600 |
| H | -5.75035000 | 0.36095600 | 0.84812500 |
| H | -5.52896200 | -0.81683000 | 2.16511800 |
| C | -5.83580600 | -1.17714100 | -2.54036700 |
| H | -4.93212200 | -1.71473700 | -0.66633600 |
| C | -5.13423700 | 0.93183100 | -3.49043500 |
| H | -3.66476000 | 2.02867700 | -2.36431500 |
| C | -5.91271000 | -0.22765700 | -3.56166800 |
| H | -6.42879700 | -2.08606900 | -2.58935200 |
| H | -5.18799300 | 1.67445100 | -4.28230600 |
| H | -6.57206100 | -0.39015600 | -4.41012900 |
| C | 7.72917800 | 1.56565000 | -2.53530800 |
| H | 7.98421300 | 2.59773300 | -2.80607800 |
| H | 8.17675400 | 1.34984900 | -1.55840700 |
| H | 8.18841000 | 0.89925800 | -3.27632400 |
| C | 6.19853700 | 1.36241800 | -2.50162700 |
| C | 5.88557700 | -0.11513500 | -2.16068500 |
| C | 5.60896500 | 2.26664600 | -1.44229100 |
| C | 4.44972300 | -0.34751300 | -1.66010700 |
| H | 6.57649500 | -0.44585100 | -1.37403400 |
| H | 6.08259600 | -0.74377000 | -3.03824300 |
| C | 4.71517800 | 1.85509800 | -0.49306800 |
| H | 5.96216100 | 3.29689900 | -1.43011800 |
| C | 4.15954300 | 0.53986400 | -0.47683900 |
| H | 4.28532700 | -1.38827000 | -1.38613600 |
| H | 3.72081000 | -0.10581100 | -2.44543100 |
| H | 4.40499300 | 2.52910800 | 0.30266900 |
| C | 5.62708100 | 1.73721100 | -3.88959500 |
| H | 5.78105500 | 2.80180200 | -4.10233400 |
| H | 6.12650800 | 1.15765400 | -4.67660600 |
| H | 4.55489900 | 1.53590800 | -3.94767500 |
| O | 3.44629100 | 0.19560000 | 0.53993400 |
| C | 4.21965100 | -2.83993700 | 1.29469800 |
| C | 3.50675200 | -2.45771100 | 2.65019000 |
| O | 3.32389300 | -2.30960000 | 0.31320700 |
| O | 2.76810700 | -1.27760300 | 2.32072400 |
| C | 5.60151300 | -2.17437300 | 1.14205400 |
| H | 6.33483800 | -2.60191100 | 1.83480200 |
| H | 5.96473700 | -2.33526300 | 0.12143500 |
| H | 5.54536100 | -1.09738300 | 1.31677000 |
| C | 4.36424700 | -4.34189400 | 1.03826600 |
| H | 4.86309900 | -4.50187100 | 0.07549500 |
| H | 4.96651500 | -4.82375400 | 1.81742800 |
| H | 3.38805700 | -4.83078100 | 0.99512700 |
| C | 2.51806400 | -3.54058500 | 3.11078400 |
| H | 3.02797000 | -4.46807700 | 3.39476900 |
| H | 1.96881000 | -3.17470700 | 3.98337700 |
| H | 1.79583500 | -3.75254400 | 2.31935400 |
| C | 4.46301800 | -2.13023000 | 3.80177900 |
| H | 3.88248400 | -1.87151000 | 4.69452900 |
| H | 5.10089200 | -2.98803000 | 4.04808000 |
| H | 5.10067100 | -1.27672400 | 3.55918600 |
| B | 2.67847400 | -1.14073900 | 0.88079300 |

# X

E = -5396.678373

$G_{Corr}$= 0.989283

| | | | |
|---|---|---|---|
| Co | -1.15302000 | -0.23998500 | 0.02522000 |
| H | 0.01324000 | -1.09252000 | 0.37915100 |
| P | 0.35194600 | 0.80459500 | -1.06874600 |
| O | 0.05584100 | 1.43618200 | -2.59086300 |
| O | 1.69571300 | -0.13279600 | -1.32789100 |
| C | 1.08648600 | 2.33140600 | -0.36086500 |
| C | -0.49947100 | 0.53251900 | -3.55282800 |
| C | 2.79353800 | 0.32427600 | -2.12675600 |
| C | 2.07621600 | 2.20217400 | 0.62580300 |
| C | 0.58171100 | 3.60460800 | -0.66178200 |
| H | -1.25906100 | -0.11117800 | -3.09591300 |

| | | | |
|---|---|---|---|
| H | -0.95923500 | 1.13440300 | -4.33915100 |
| H | 0.28784400 | -0.09419300 | -3.98902100 |
| H | 3.21976200 | 1.25290700 | -1.73608500 |
| H | 3.54511100 | -0.46433300 | -2.07017900 |
| H | 2.48583900 | 0.47690800 | -3.16737900 |
| C | 2.54946400 | 3.32853800 | 1.30176700 |
| H | 2.47089900 | 1.22298200 | 0.87222800 |
| C | 1.05829700 | 4.72975200 | 0.01380100 |
| H | -0.19182900 | 3.70797300 | -1.41381600 |
| C | 2.04061000 | 4.59535800 | 0.99961500 |
| H | 3.31154800 | 3.21321500 | 2.06829900 |
| H | 0.66022800 | 5.71241000 | -0.22687900 |
| H | 2.40539800 | 5.47093000 | 1.53026200 |
| P | -1.84188200 | -2.11843100 | -0.66215900 |
| O | -1.14216700 | -3.40828100 | 0.14836200 |
| O | -1.60023300 | -2.62184700 | -2.24327900 |
| C | -3.63133100 | -2.54472200 | -0.58748100 |
| C | -1.53405200 | -4.75263300 | -0.14110300 |
| C | -0.24003100 | -2.74512600 | -2.67506100 |
| C | -4.31711100 | -2.13137300 | 0.56568000 |
| C | -4.33555900 | -3.20008700 | -1.60763700 |
| H | -0.80441100 | -5.40340500 | 0.34809100 |
| H | -2.53515400 | -4.96433500 | 0.25336300 |
| H | -1.53113500 | -4.94840200 | -1.21901000 |
| H | 0.39066800 | -1.95425900 | -2.25633400 |
| H | 0.16904100 | -3.71745400 | -2.37495800 |
| H | -0.24002900 | -2.67516500 | -3.76603900 |
| C | -5.68829200 | -2.35932600 | 0.69187700 |
| H | -3.77830600 | -1.58728600 | 1.33466400 |
| C | -5.71025500 | -3.41614000 | -1.48607300 |
| H | -3.80768200 | -3.51721600 | -2.50052500 |
| C | -6.38896700 | -2.99455200 | -0.33874700 |
| H | -6.21155100 | -2.03060300 | 1.58612400 |
| H | -6.25286700 | -3.91056500 | -2.28781800 |
| H | -7.45969900 | -3.15851500 | -0.24889400 |
| P | -1.05948400 | 0.24760500 | 2.11127500 |
| O | 0.52362200 | 0.29091300 | 2.60624800 |
| O | -1.78748700 | -0.69946600 | 3.27738600 |
| C | -1.71206500 | 1.83642700 | 2.77911800 |
| C | 0.89087400 | 0.50666500 | 3.97250000 |
| C | -1.64819100 | -2.12262800 | 3.20585600 |
| C | -0.95690700 | 3.00292300 | 2.59402900 |
| C | -2.98416500 | 1.93368100 | 3.36019400 |
| H | 0.28175100 | -0.10535900 | 4.64676000 |
| H | 1.93968400 | 0.21172600 | 4.05774100 |
| H | 0.77911900 | 1.56216300 | 4.24567300 |
| H | -2.61014200 | -2.56732600 | 3.48104100 |
| H | -1.36889300 | -2.45135200 | 2.20213000 |
| H | -0.88370100 | -2.45115800 | 3.91885800 |
| C | -1.45737800 | 4.24273800 | 2.99330700 |
| H | 0.02168400 | 2.94385700 | 2.13344100 |
| C | -3.48711300 | 3.17538300 | 3.75694000 |
| H | -3.57820200 | 1.03735900 | 3.49835700 |
| C | -2.72472100 | 4.33354700 | 3.57513500 |
| H | -0.85568900 | 5.13524400 | 2.84246800 |
| H | -4.47236200 | 3.23758700 | 4.21286300 |
| H | -3.11726100 | 5.29918500 | 3.88380700 |
| P | -2.82186400 | 1.06595300 | -0.39373800 |
| O | -2.32871500 | 2.63396500 | -0.28253500 |
| O | -4.04799000 | 0.86311400 | 0.68898000 |
| C | -3.73977000 | 1.10367800 | -1.99021600 |
| C | -3.19455700 | 3.76509900 | -0.16796200 |
| C | -5.32767900 | 1.50233900 | 0.70538300 |
| C | -4.79334600 | 0.20825300 | -2.23364100 |
| C | -3.33050200 | 1.97047000 | -3.01499600 |
| H | -2.56859100 | 4.64753100 | -0.32182000 |
| H | -3.98840400 | 3.74925200 | -0.92446200 |
| H | -3.62669000 | 3.80858100 | 0.83566700 |
| H | -5.34582800 | 2.22811700 | 1.52442700 |
| H | -5.55843400 | 2.00352300 | -0.23923500 |
| H | -6.07692500 | 0.72472000 | 0.88357000 |
| C | -5.41652900 | 0.17453000 | -3.48191600 |
| H | -5.13247700 | -0.45805200 | -1.44864300 |
| C | -3.95984300 | 1.94061100 | -4.26048900 |
| H | -2.50622000 | 2.65437800 | -2.83769000 |
| C | -5.00128700 | 1.03936400 | -4.49815400 |
| H | -6.22756900 | -0.52735400 | -3.65667600 |
| H | -3.63491600 | 2.61821500 | -5.04576400 |
| H | -5.48781800 | 1.01275000 | -5.46954600 |
| C | 8.46514600 | 2.07240500 | -2.53270200 |
| H | 8.22279400 | 2.97291300 | -3.11063800 |
| H | 9.04278000 | 2.38082400 | -1.65338700 |
| H | 9.10518300 | 1.43509600 | -3.15573200 |
| C | 7.18289100 | 1.32499700 | -2.11883200 |
| C | 7.56849600 | 0.06403000 | -1.30555200 |
| C | 6.31882500 | 2.21920100 | -1.25691500 |
| C | 6.36717100 | -0.63319400 | -0.64162500 |
| H | 8.27093900 | 0.36665900 | -0.51806400 |
| H | 8.09797100 | -0.64636700 | -1.95288400 |
| C | 5.55130400 | 1.71822400 | -0.21569900 |
| H | 6.27221300 | 3.27778700 | -1.50112000 |
| C | 5.51963600 | 0.36478900 | 0.09322100 |
| H | 6.72225400 | -1.40190800 | 0.05808700 |
| H | 5.76502200 | -1.17194300 | -1.38644900 |
| H | 4.92147300 | 2.38567600 | 0.36427000 |
| C | 6.40427100 | 0.92711100 | -3.39693900 |
| H | 6.11690100 | 1.81766000 | -3.96794300 |
| H | 7.02202600 | 0.29103400 | -4.04503400 |
| H | 5.48843900 | 0.38004500 | -3.15345600 |
| O | 4.74114900 | -0.05010900 | 1.14946300 |
| C | 3.47358300 | -3.38050200 | 0.65406300 |
| C | 3.11937400 | -3.07934600 | 2.16830500 |
| O | 3.83548900 | -2.06627000 | 0.13958000 |
| O | 3.85801200 | -1.84880700 | 2.43534500 |
| C | 4.71645200 | -4.26102400 | 0.47840400 |
| H | 4.53268200 | -5.28897200 | 0.80598700 |
| H | 4.99129700 | -4.27752500 | -0.58090500 |
| H | 5.56583600 | -3.86115200 | 1.04197300 |
| C | 2.31272500 | -3.92398300 | -0.17310400 |
| H | 2.63004800 | -4.03221100 | -1.21584100 |
| H | 2.01367800 | -4.91181200 | 0.19624500 |
| H | 1.44219800 | -3.26807500 | -0.14257300 |
| C | 1.64119300 | -2.77158500 | 2.39760000 |
| H | 1.01834500 | -3.65787400 | 2.24511300 |
| H | 1.51145000 | -2.42550300 | 3.42733900 |
| H | 1.28248100 | -1.98184700 | 1.73612200 |
| C | 3.59830700 | -4.14048500 | 3.15618800 |
| H | 3.32863000 | -3.83939700 | 4.17351600 |
| H | 3.11873100 | -5.10302100 | 2.94685300 |
| H | 4.68206800 | -4.27222100 | 3.11806600 |
| B | 4.15480700 | -1.28862200 | 1.22204900 |

## TS X-XI

E = -5396.650143
$G_{Corr}$= 0.987578

| | | | |
|---|---|---|---|
| Co | -0.75222200 | 0.23842500 | 0.03741500 |
| H | 0.68572300 | 1.01260200 | -0.13782700 |
| P | 0.22973100 | -0.88367500 | 1.60779100 |
| O | -0.65989900 | -1.31340600 | 2.96847200 |
| O | 1.52243700 | -0.15234200 | 2.35266800 |
| C | 0.84026400 | -2.59413000 | 1.27020000 |
| C | -1.17411800 | -0.24438900 | 3.76356800 |
| C | 2.40728300 | -0.88782900 | 3.20356300 |
| C | 1.70957200 | -2.77094600 | 0.18445100 |
| C | 0.43526300 | -3.71572600 | 2.00984300 |
| H | -1.74262800 | 0.46524500 | 3.15353700 |
| H | -1.83827900 | -0.68964000 | 4.50752100 |
| H | -0.36021900 | 0.28498700 | 4.27288100 |
| H | 3.09298200 | -1.49502300 | 2.60371300 |
| H | 2.97816000 | -0.14843000 | 3.77353600 |

| | | | |
|---|---|---|---|
| H | 1.85396900 | -1.53086600 | 3.89765100 |
| C | 2.18099400 | -4.04400200 | -0.14339100 |
| H | 1.99958300 | -1.91516200 | -0.41122600 |
| C | 0.88974000 | -4.99072100 | 1.66545400 |
| H | -0.23906900 | -3.58738000 | 2.84890000 |
| C | 1.76773500 | -5.15863300 | 0.59028300 |
| H | 2.86518500 | -4.16219000 | -0.98009600 |
| H | 0.56159500 | -5.85373900 | 2.24010500 |
| H | 2.12495800 | -6.15122000 | 0.32702800 |
| P | -1.59195100 | 2.16681800 | 0.43454900 |
| O | -0.82592000 | 3.49084500 | -0.25669800 |
| O | -1.75021200 | 2.73900700 | 2.00895800 |
| C | -3.33293600 | 2.52126400 | -0.06154400 |
| C | -1.36408600 | 4.80763200 | -0.09539200 |
| C | -0.54127800 | 2.89930300 | 2.75316100 |
| C | -3.71232000 | 2.10241300 | -1.34626500 |
| C | -4.26880600 | 3.18122500 | 0.74803400 |
| H | -0.57360000 | 5.50880500 | -0.37324600 |
| H | -2.23159200 | 4.95645800 | -0.74834700 |
| H | -1.66406500 | 4.99188300 | 0.94160100 |
| H | 0.12110900 | 2.03691200 | 2.62496500 |
| H | -0.00946500 | 3.80581400 | 2.43932400 |
| H | -0.81992900 | 2.98959400 | 3.80661300 |
| C | -5.01212100 | 2.32153900 | -1.80578500 |
| H | -2.99708500 | 1.56977600 | -1.96507500 |
| C | -5.57119200 | 3.39384200 | 0.29047500 |
| H | -3.97799000 | 3.50836700 | 1.74023200 |
| C | -5.94684100 | 2.96035700 | -0.98443700 |
| H | -5.29803600 | 1.98557500 | -2.79930300 |
| H | -6.29418200 | 3.89357200 | 0.93031400 |
| H | -6.96264300 | 3.12027400 | -1.33681900 |
| P | -0.40654700 | -0.35603100 | -2.02272500 |
| O | 1.18649000 | -0.69434400 | -2.37526800 |
| O | -0.81850100 | 0.68396900 | -3.26637400 |
| C | -1.20411500 | -1.83089400 | -2.80773800 |
| C | 1.60019100 | -1.03734400 | -3.70093800 |
| C | -0.39676300 | 2.05050900 | -3.19855500 |
| C | -0.78863100 | -3.10059500 | -2.38148000 |
| C | -2.20038000 | -1.73676700 | -3.78808100 |
| H | 1.16591200 | -0.35730300 | -4.44216600 |
| H | 2.69034100 | -0.95017800 | -3.72010500 |
| H | 1.31278000 | -2.06570300 | -3.94784700 |
| H | -1.17755700 | 2.65857800 | -3.66678400 |
| H | -0.25069700 | 2.37836500 | -2.16783800 |
| H | 0.54106900 | 2.17720200 | -3.75117900 |
| C | -1.35282900 | -4.25397500 | -2.92917300 |
| H | -0.02799600 | -3.18970900 | -1.61400000 |
| C | -2.77036900 | -2.89058600 | -4.33234500 |
| H | -2.52809100 | -0.75893200 | -4.12273600 |
| C | -2.34826200 | -4.15295200 | -3.90532700 |
| H | -1.01574300 | -5.23012600 | -2.58932100 |
| H | -3.54230900 | -2.80314500 | -5.09319800 |
| H | -2.79079200 | -5.05011400 | -4.33080700 |
| P | -2.59775900 | -0.99421800 | 0.19209500 |
| O | -2.15403400 | -2.57768300 | 0.29096000 |
| O | -3.54415200 | -0.82766700 | -1.14402000 |
| C | -3.87578300 | -0.91170900 | 1.52327900 |
| C | -3.01743200 | -3.71302700 | 0.19757100 |
| C | -4.76078400 | -1.51496500 | -1.44892300 |
| C | -4.96531900 | -0.03424300 | 1.40717400 |
| C | -3.74872300 | -1.68530100 | 2.68780300 |
| H | -2.46203500 | -4.55384500 | 0.62152400 |
| H | -3.94569500 | -3.57247400 | 0.76227400 |
| H | -3.24359600 | -3.92546300 | -0.85139400 |
| H | -4.54870000 | -2.30098800 | -2.17934900 |
| H | -5.23177700 | -1.94524900 | -0.56007100 |
| H | -5.44245700 | -0.77971700 | -1.88766000 |
| C | -5.89498500 | 0.08271000 | 2.44160300 |
| H | -5.09531300 | 0.55084200 | 0.50483400 |
| C | -4.68420100 | -1.57432000 | 3.71754900 |
| H | -2.90385800 | -2.35741500 | 2.78967000 |
| C | -5.75642200 | -0.68530900 | 3.60026600 |
| H | -6.72778500 | 0.77297700 | 2.33649400 |
| H | -4.57434700 | -2.18104700 | 4.61279400 |
| H | -6.48132500 | -0.59555000 | 4.40506300 |
| C | 2.47878300 | 5.67406900 | -1.16734700 |
| H | 2.98644600 | 6.06024300 | -2.06044900 |
| H | 1.41125900 | 5.58746800 | -1.39945800 |
| H | 2.59659800 | 6.41476700 | -0.36624200 |
| C | 3.05991000 | 4.31256500 | -0.74434600 |
| C | 2.34771600 | 3.82298900 | 0.53845700 |
| C | 2.84595400 | 3.29559400 | -1.84579200 |
| C | 2.67175800 | 2.36769200 | 0.89589100 |
| H | 1.26954100 | 3.90135000 | 0.38123100 |
| H | 2.61135800 | 4.47895700 | 1.37954500 |
| C | 2.60539000 | 1.98346700 | -1.60422200 |
| H | 2.96316600 | 3.64052400 | -2.87297700 |
| C | 2.44614200 | 1.44970800 | -0.27874900 |
| H | 2.07719500 | 2.02753700 | 1.74446100 |
| H | 3.72527700 | 2.27570500 | 1.19777000 |
| H | 2.53329400 | 1.27238700 | -2.41914500 |
| C | 4.58305000 | 4.46257200 | -0.50694900 |
| H | 5.08576100 | 4.81233700 | -1.41726500 |
| H | 4.77934900 | 5.19325400 | 0.28939900 |
| H | 5.03341200 | 3.50538200 | -0.22528400 |
| O | 2.89880300 | 0.14051300 | -0.10647900 |
| C | 6.42012300 | 0.02258900 | 0.53513800 |
| C | 6.02571200 | -1.49088800 | 0.34848000 |
| O | 5.26339200 | 0.70865400 | -0.02101300 |
| O | 4.57748400 | -1.44405400 | 0.44643000 |
| C | 6.51366500 | 0.43955500 | 2.00782500 |
| H | 7.39856600 | 0.01440100 | 2.49194600 |
| H | 6.57648400 | 1.53072400 | 2.06309400 |
| H | 5.62447300 | 0.12115400 | 2.56177400 |
| C | 7.66325900 | 0.46663300 | -0.22752500 |
| H | 7.84823600 | 1.53002900 | -0.04409000 |
| H | 8.54152400 | -0.09649700 | 0.10707900 |
| H | 7.54379000 | 0.32459200 | -1.30387300 |
| C | 6.35830100 | -2.02955300 | -1.04822700 |
| H | 7.43641900 | -2.16116100 | -1.18482800 |
| H | 5.86867700 | -2.99984600 | -1.17675300 |
| H | 5.98662600 | -1.35468900 | -1.82615200 |
| C | 6.55328200 | -2.43405700 | 1.42454500 |
| H | 6.21266900 | -3.45363100 | 1.21746400 |
| H | 7.64883600 | -2.43316600 | 1.43596800 |
| H | 6.19091800 | -2.15195100 | 2.41563700 |
| B | 4.20080700 | -0.16562200 | 0.09350300 |

## TS X-XI’

E = 0.987578

$G_{Corr}$= 0.987356

| | | | |
|---|---|---|---|
| Co | 1.03471400 | -0.10557400 | -0.22251100 |
| H | -0.55721600 | -0.03067100 | -0.76748900 |
| P | 0.34103500 | 0.79770400 | 1.60361200 |
| O | 1.03531700 | 0.31377800 | 3.05205700 |
| O | -1.26727700 | 0.62023300 | 1.97354000 |
| C | 0.54433600 | 2.60695500 | 1.86202000 |
| C | 0.90129900 | -1.08209400 | 3.35122700 |
| C | -1.83569800 | 1.11849900 | 3.18958200 |
| C | -0.36729600 | 3.45736900 | 1.21524200 |
| C | 1.61279800 | 3.16681200 | 2.57397600 |
| H | 1.14993500 | -1.69746900 | 2.48021100 |
| H | 1.59310800 | -1.30571000 | 4.16499200 |
| H | -0.12422300 | -1.31242500 | 3.66460000 |
| H | -1.82040400 | 2.21385900 | 3.21333500 |
| H | -2.87060800 | 0.76673700 | 3.20925100 |
| H | -1.29895000 | 0.73588300 | 4.06386100 |
| C | -0.21398100 | 4.84284000 | 1.28191100 |
| H | -1.19158900 | 3.03057700 | 0.65037600 |
| C | 1.77161800 | 4.55316600 | 2.63185800 |

| | | | |
|---|---|---|---|
| H | 2.32195300 | 2.51641800 | 3.07215200 |
| C | 0.86122800 | 5.39457500 | 1.98612100 |
| H | -0.92752700 | 5.49096200 | 0.77976700 |
| H | 2.60720600 | 4.97769300 | 3.18288200 |
| H | 0.98756100 | 6.47307000 | 2.03155800 |
| P | 0.74530300 | -2.21659200 | -0.49027000 |
| O | 0.01355600 | -2.66863000 | -1.92715300 |
| O | -0.17261400 | -3.07573700 | 0.60956700 |
| C | 2.14056500 | -3.43379500 | -0.49117000 |
| C | -0.30393600 | -4.03653900 | -2.20570800 |
| C | -1.45950800 | -2.56532800 | 0.97616000 |
| C | 3.07769000 | -3.35244600 | -1.53499700 |
| C | 2.29008800 | -4.43978200 | 0.47288300 |
| H | -0.96345900 | -4.03259000 | -3.07747400 |
| H | 0.60173100 | -4.61058500 | -2.43344400 |
| H | -0.81936600 | -4.50225300 | -1.35908000 |
| H | -1.46396000 | -1.47512200 | 1.01892600 |
| H | -2.21669100 | -2.89902000 | 0.25728400 |
| H | -1.69854900 | -2.97346200 | 1.96257900 |
| C | 4.13438000 | -4.26012100 | -1.61644800 |
| H | 2.99004000 | -2.56855100 | -2.27798900 |
| C | 3.35336200 | -5.34363900 | 0.39708100 |
| H | 1.56882800 | -4.51680600 | 1.27869600 |
| C | 4.27650300 | -5.25904300 | -0.64697600 |
| H | 4.84830400 | -4.18638900 | -2.43288800 |
| H | 3.45950700 | -6.11447900 | 1.15604600 |
| H | 5.10151100 | -5.96408800 | -0.70626200 |
| P | 1.41341800 | 1.36048600 | -1.79836500 |
| O | 0.14348900 | 2.40658100 | -2.02551800 |
| O | 1.69812700 | 0.91691000 | -3.38596600 |
| C | 2.78682100 | 2.60632000 | -1.70654700 |
| C | 0.11636500 | 3.35478500 | -3.09765900 |
| C | 2.00238000 | -0.42952000 | -3.73498600 |
| C | 2.65603200 | 3.72591800 | -0.87299300 |
| C | 3.96807800 | 2.44528300 | -2.44146900 |
| H | 0.21851100 | 2.85389000 | -4.06536600 |
| H | -0.85571300 | 3.85204200 | -3.04419000 |
| H | 0.91309500 | 4.09921700 | -2.98899100 |
| H | 3.08894900 | -0.58569100 | -3.73034800 |
| H | 1.53637700 | -1.13172900 | -3.04121200 |
| H | 1.61427200 | -0.60379200 | -4.74313400 |
| C | 3.67927000 | 4.66967800 | -0.78418100 |
| H | 1.75173500 | 3.86004200 | -0.29261000 |
| C | 4.99856200 | 3.38648400 | -2.34930500 |
| H | 4.07773200 | 1.58912000 | -3.09834300 |
| C | 4.85519600 | 4.50382500 | -1.52244600 |
| H | 3.55572700 | 5.53194200 | -0.13392500 |
| H | 5.90712900 | 3.25144100 | -2.93142100 |
| H | 5.65339800 | 5.23858600 | -1.45347900 |
| P | 3.17122400 | -0.08361700 | 0.40006600 |
| O | 3.51348000 | 1.42895300 | 0.95343300 |
| O | 4.13812000 | -0.37663100 | -0.90974500 |
| C | 4.01872800 | -1.07223000 | 1.71621100 |
| C | 4.81016400 | 1.98051700 | 1.19067400 |
| C | 5.56954000 | -0.32756600 | -0.95754900 |
| C | 4.63503000 | -2.29996900 | 1.43702600 |
| C | 4.02151900 | -0.60275600 | 3.04106500 |
| H | 4.65710000 | 2.86380800 | 1.81555600 |
| H | 5.46632700 | 1.27580600 | 1.71399800 |
| H | 5.26167500 | 2.29040500 | 0.24521200 |
| H | 5.87881100 | 0.62741300 | -1.39331400 |
| H | 6.02461500 | -0.44738300 | 0.02984300 |
| H | 5.89975100 | -1.14959600 | -1.59957100 |
| C | 5.23063300 | -3.04618500 | 2.45578100 |
| H | 4.65851800 | -2.67527200 | 0.42207400 |
| C | 4.61819100 | -1.34722200 | 4.05862100 |
| H | 3.53781700 | 0.33887500 | 3.27413300 |
| C | 5.22287100 | -2.57420600 | 3.76943500 |
| H | 5.69530000 | -3.99849300 | 2.21584900 |
| H | 4.61104400 | -0.96874700 | 5.07757600 |
| H | 5.68551200 | -3.15555500 | 4.56267000 |
| C | -1.39833400 | -0.23245300 | -3.61900400 |
| H | -0.97397800 | 0.75495300 | -3.81786000 |
| H | -0.62022800 | -0.85751100 | -3.18471900 |
| H | -1.71163500 | -0.67146300 | -4.57617100 |
| C | -2.59033300 | -0.13787600 | -2.66044300 |
| C | -3.13609700 | -1.55809400 | -2.38153500 |
| C | -2.21126100 | 0.51847000 | -1.33816300 |
| C | -4.31266800 | -1.57984000 | -1.39144900 |
| H | -2.31095200 | -2.14781500 | -1.97477300 |
| H | -3.43876400 | -2.03019500 | -3.32556200 |
| C | -3.11626800 | 0.32824500 | -0.25014700 |
| H | -1.71869600 | 1.48016600 | -1.41437000 |
| C | -4.07959100 | -0.62883700 | -0.25791200 |
| H | -4.46005200 | -2.59333300 | -0.99119900 |
| H | -5.25686800 | -1.31359800 | -1.88883700 |
| H | -3.03393800 | 0.95970000 | 0.62379700 |
| C | -3.68615700 | 0.74002100 | -3.32066900 |
| H | -3.29264000 | 1.73962300 | -3.54242600 |
| H | -4.01691900 | 0.29017400 | -4.26664000 |
| H | -4.55934900 | 0.85945300 | -2.67087100 |
| O | -4.90718200 | -0.77210800 | 0.85277700 |
| C | -8.27346200 | -0.00181200 | -0.10642500 |
| C | -8.35510200 | -0.09994000 | 1.46477900 |
| O | -6.83801600 | 0.09153200 | -0.33192400 |
| O | -7.09749000 | -0.75421100 | 1.79988700 |
| C | -8.74940400 | -1.27605600 | -0.81384500 |
| H | -9.83578700 | -1.39160900 | -0.74661500 |
| H | -8.46996200 | -1.21950000 | -1.87039400 |
| H | -8.27595300 | -2.16422000 | -0.38312800 |
| C | -8.94250000 | 1.22708400 | -0.71285600 |
| H | -8.82468400 | 1.21111900 | -1.80111300 |
| H | -10.01403200 | 1.23340200 | -0.48469700 |
| H | -8.49780100 | 2.15133900 | -0.33764000 |
| C | -8.33947100 | 1.27063500 | 2.15190200 |
| H | -9.28275500 | 1.80558800 | 2.00277500 |
| H | -8.18674400 | 1.12662100 | 3.22578200 |
| H | -7.52173500 | 1.89117700 | 1.77142000 |
| C | -9.50372200 | -0.95043500 | 1.99717300 |
| H | -9.47660000 | -0.96372100 | 3.09145200 |
| H | -10.46730100 | -0.53365000 | 1.68390900 |
| H | -9.43566300 | -1.98168800 | 1.64394700 |
| B | -6.23611800 | -0.48594200 | 0.76408500 |

# XI

E = -799.5872832

$G_{Corr}$= 0.335457

| | | | |
|---|---|---|---|
| C | -5.13623400 | 1.35905800 | -0.00519600 |
| H | -5.50839900 | 1.73172400 | 0.95712700 |
| H | -4.67679700 | 2.19898700 | -0.53929300 |
| H | -5.99859500 | 1.01410100 | -0.58936500 |
| C | -4.12683500 | 0.21037100 | 0.19629000 |
| C | -3.66026800 | -0.31325300 | -1.18444800 |
| C | -2.93436600 | 0.72600400 | 0.97991500 |
| C | -2.36297300 | -1.12577400 | -1.10112500 |
| H | -3.49658400 | 0.54535000 | -1.85114500 |
| H | -4.45867600 | -0.91320400 | -1.63849700 |
| C | -1.65632800 | 0.52953200 | 0.64446800 |
| H | -3.16881100 | 1.29488400 | 1.88070900 |
| H | -2.09068500 | -1.53625800 | -2.07998600 |
| H | -2.48072900 | -1.97435500 | -0.41709000 |
| H | -0.85192500 | 0.95088200 | 1.24422100 |
| C | -4.81110200 | -0.91394900 | 1.00803600 |
| H | -5.09457200 | -0.55712100 | 2.00553500 |
| H | -5.72206600 | -1.25494600 | 0.50024600 |
| H | -4.14770500 | -1.77505300 | 1.13966400 |
| O | -0.12080600 | -1.12094000 | -0.24418900 |
| C | 2.88956800 | 0.81700700 | -0.32296400 |
| C | 3.32115700 | -0.49679200 | 0.43218100 |
| O | 1.43686900 | 0.73292700 | -0.27510800 |

| | | | |
|---|---|---|---|
| O | 2.21950100 | -1.40024600 | 0.13635600 |
| C | 3.32554400 | 2.11769000 | 0.34354800 |
| H | 4.41808600 | 2.16963900 | 0.40659800 |
| H | 2.97666600 | 2.97069400 | -0.24743900 |
| H | 2.91109300 | 2.21038700 | 1.34984600 |
| C | 3.28483400 | 0.81495700 | -1.80429800 |
| H | 2.77594800 | 1.64245700 | -2.30810800 |
| H | 4.36459500 | 0.94074900 | -1.93237700 |
| H | 2.98194200 | -0.11852200 | -2.28939100 |
| C | 4.61795300 | -1.12770800 | -0.06481900 |
| H | 5.45640800 | -0.43384300 | 0.06193600 |
| H | 4.83282100 | -2.03241300 | 0.51286200 |
| H | 4.54940000 | -1.40709000 | -1.11849700 |
| C | 3.35931600 | -0.32565800 | 1.95575100 |
| H | 3.45500600 | -1.31171200 | 2.42024000 |
| H | 4.20679300 | 0.29161900 | 2.27042400 |
| H | 2.43624100 | 0.13519700 | 2.32211500 |
| B | 1.12846300 | -0.60578500 | -0.13450800 |
| C | -1.22731900 | -0.25357300 | -0.57247000 |
| H | -0.89396300 | 0.44536800 | -1.35346800 |

## TS IX-X

E = -5396.617177
$G_{Corr}$= 0.990557

| | | | |
|---|---|---|---|
| Co | -0.58209500 | -0.23108500 | 0.04413600 |
| H | 1.07388900 | -0.89591400 | 0.50915400 |
| P | 0.19885500 | 1.13423700 | -1.49109700 |
| O | -0.82428400 | 1.39293900 | -2.79695800 |
| O | 1.60881800 | 0.80586600 | -2.32029700 |
| C | 0.44610900 | 2.90248200 | -1.05334600 |
| C | -1.06367100 | 0.24275200 | -3.61674400 |
| C | 2.09298300 | 1.73630100 | -3.29852200 |
| C | 1.54571900 | 3.20703500 | -0.23412500 |
| C | -0.43704000 | 3.92392000 | -1.42686000 |
| H | -1.32561600 | -0.62922100 | -3.00638700 |
| H | -1.89775800 | 0.48607400 | -4.27682900 |
| H | -0.17573400 | 0.00464000 | -4.21452300 |
| H | 2.70340400 | 2.50843500 | -2.81773900 |
| H | 2.70734300 | 1.16975300 | -4.00365300 |
| H | 1.27017900 | 2.21207600 | -3.84183600 |
| C | 1.76411900 | 4.51900000 | 0.18919900 |
| H | 2.20077300 | 2.40827600 | 0.09596600 |
| C | -0.22609700 | 5.23229100 | -0.98545800 |
| H | -1.28856800 | 3.69221600 | -2.05605900 |
| C | 0.87589900 | 5.53431000 | -0.18009800 |
| H | 2.62003300 | 4.74594400 | 0.81990400 |
| H | -0.92026300 | 6.01781900 | -1.27422200 |
| H | 1.03973600 | 6.55367800 | 0.15996500 |
| P | -0.95029400 | -2.33379300 | -0.46695900 |
| O | -0.29278400 | -3.41655000 | 0.61869900 |
| O | -0.39502600 | -2.93818200 | -1.91777300 |
| C | -2.63772500 | -3.06634100 | -0.62041100 |
| C | -0.26830700 | -4.81920100 | 0.33456700 |
| C | 0.93552600 | -2.58810600 | -2.33392400 |
| C | -3.41984700 | -3.12587800 | 0.54500200 |
| C | -3.15816700 | -3.57961300 | -1.81544200 |
| H | 0.08613900 | -5.31399300 | 1.24227700 |
| H | -1.26682200 | -5.19653900 | 0.08425100 |
| H | 0.41619600 | -5.03663300 | -0.49158200 |
| H | 1.06891900 | -1.50336500 | -2.35074400 |
| H | 1.67950500 | -3.01615700 | -1.65738000 |
| H | 1.05828200 | -2.99257100 | -3.34272700 |
| C | -4.69651100 | -3.68779400 | 0.51535800 |
| H | -3.03207700 | -2.71809300 | 1.47173100 |
| C | -4.43913600 | -4.13794200 | -1.84666000 |
| H | -2.55619500 | -3.54548900 | -2.71680200 |
| C | -5.21078500 | -4.19366100 | -0.68405600 |
| H | -5.29088300 | -3.72945900 | 1.42449600 |
| H | -4.83386300 | -4.52888100 | -2.78085000 |
| H | -6.20664600 | -4.62811900 | -0.70994900 |
| P | -0.45377800 | 0.67427900 | 2.08233700 |
| O | 1.04393000 | 1.30953300 | 2.27815100 |
| O | -0.62014800 | -0.27247600 | 3.44551500 |
| C | -1.45962600 | 2.10407400 | 2.69868400 |
| C | 1.59273100 | 1.59714200 | 3.56561900 |
| C | -1.26558600 | -1.54434100 | 3.35992300 |
| C | -1.11553900 | 3.40847100 | 2.31574600 |
| C | -2.56778100 | 1.91251100 | 3.53348300 |
| H | 1.86983100 | 0.66628000 | 4.06503800 |
| H | 2.48944500 | 2.19723200 | 3.38932500 |
| H | 0.89008900 | 2.16813600 | 4.18523300 |
| H | -2.35578400 | -1.41949200 | 3.33484300 |
| H | -0.94651900 | -2.09388900 | 2.47296400 |
| H | -0.98619500 | -2.10300200 | 4.25711000 |
| C | -1.86057700 | 4.49888900 | 2.76509200 |
| H | -0.26505200 | 3.56971200 | 1.66428200 |
| C | -3.31951400 | 3.00393200 | 3.98035100 |
| H | -2.83524600 | 0.90957100 | 3.84954300 |
| C | -2.96564100 | 4.30104400 | 3.59884600 |
| H | -1.57698600 | 5.50311300 | 2.46007300 |
| H | -4.17254800 | 2.84117400 | 4.63476300 |
| H | -3.54635500 | 5.15064700 | 3.94898800 |
| P | -2.67029500 | 0.54814500 | -0.17855200 |
| O | -2.59022400 | 2.18519100 | -0.08707500 |
| O | -3.55293800 | 0.00592900 | 1.10646400 |
| C | -3.88038800 | 0.37695700 | -1.56602800 |
| C | -3.68354900 | 3.07792300 | 0.14748500 |
| C | -4.90844500 | 0.34490600 | 1.43143000 |
| C | -4.78524900 | -0.69277300 | -1.62532600 |
| C | -3.88236500 | 1.32097300 | -2.60795800 |
| H | -3.32627800 | 4.07317800 | -0.12597700 |
| H | -4.55623400 | 2.82291400 | -0.46395900 |
| H | -3.94720200 | 3.07381200 | 1.20767700 |
| H | -4.90400800 | 1.08108700 | 2.24045800 |
| H | -5.45646700 | 0.74252900 | 0.57274300 |
| H | -5.39815600 | -0.57340500 | 1.76831400 |
| C | -5.66175300 | -0.82476600 | -2.70375700 |
| H | -4.81767600 | -1.41984900 | -0.82463400 |
| C | -4.76082100 | 1.19036400 | -3.68334900 |
| H | -3.18110700 | 2.14659200 | -2.57991700 |
| C | -5.65130800 | 0.11363900 | -3.73724700 |
| H | -6.34912400 | -1.66565900 | -2.73045400 |
| H | -4.74996600 | 1.93018400 | -4.47959800 |
| H | -6.33317100 | 0.01035500 | -4.57718500 |
| C | 8.08442600 | 0.72160100 | -2.00454400 |
| H | 8.55221100 | 1.71187500 | -2.06767000 |
| H | 8.44101000 | 0.23533400 | -1.08911300 |
| H | 8.42973500 | 0.13009600 | -2.86135600 |
| C | 6.54694900 | 0.83393100 | -2.01015700 |
| C | 5.92702600 | -0.58140100 | -1.93104200 |
| C | 6.09073200 | 1.64367000 | -0.81879100 |
| C | 4.42243200 | -0.58156700 | -1.63096000 |
| H | 6.44092000 | -1.13324600 | -1.13399200 |
| H | 6.12244300 | -1.12050000 | -2.86658400 |
| C | 4.95248100 | 1.36517900 | -0.11230000 |
| H | 6.69147700 | 2.51013400 | -0.54674500 |
| C | 4.07402900 | 0.30345500 | -0.46638300 |
| H | 4.04788600 | -1.58716000 | -1.42881900 |
| H | 3.84379900 | -0.21928100 | -2.49046100 |
| H | 4.65620800 | 1.98323900 | 0.73202200 |
| C | 6.10421000 | 1.55950000 | -3.30497900 |
| H | 6.53282200 | 2.56737600 | -3.35304800 |
| H | 6.44269300 | 1.00321700 | -4.18867100 |
| H | 5.01599100 | 1.65744900 | -3.35522900 |
| O | 2.93696700 | 0.20510600 | 0.14874500 |
| C | 3.61435500 | -2.97202800 | 1.27850700 |
| C | 3.07337900 | -2.34674800 | 2.61673200 |
| O | 2.79907300 | -2.33158200 | 0.28007800 |
| O | 2.62249600 | -1.05505400 | 2.19874700 |
| C | 5.08801600 | -2.61819300 | 1.01798300 |

| | | | |
|---|---|---|---|
| H | 5.75281500 | -3.11016600 | 1.73594800 |
| H | 5.36720000 | -2.95180300 | 0.01367000 |
| H | 5.25495200 | -1.53841400 | 1.07768300 |
| C | 3.43459900 | -4.48443200 | 1.15343700 |
| H | 3.82155800 | -4.82342600 | 0.18565200 |
| H | 3.98339800 | -5.01134100 | 1.94293400 |
| H | 2.38201000 | -4.76253700 | 1.21199900 |
| C | 1.87462700 | -3.11409200 | 3.19230300 |
| H | 2.16972400 | -4.09056600 | 3.59418300 |
| H | 1.44215600 | -2.52153100 | 4.00443300 |
| H | 1.10940200 | -3.25336700 | 2.42713700 |
| C | 4.12818600 | -2.15655700 | 3.70952300 |
| H | 3.65812700 | -1.71601900 | 4.59575600 |
| H | 4.57246400 | -3.11547400 | 4.00199400 |
| H | 4.92547300 | -1.48385600 | 3.38397000 |
| B | 2.31437200 | -1.08603400 | 0.80428300 |

# $X^{BS}$

E = -5396.636734

$G_{Corr}$= 0.987256

| | | | |
|---|---|---|---|
| Co | -0.84362500 | -0.17146800 | 0.01355500 |
| H | 1.68010100 | -0.83548300 | 0.49814100 |
| P | 0.03786300 | 1.46585100 | -1.11288900 |
| O | -0.76572200 | 1.94579700 | -2.50385900 |
| O | 1.59377000 | 1.28155700 | -1.70329000 |
| C | 0.18866900 | 3.13361200 | -0.35829400 |
| C | -0.87938800 | 0.93365700 | -3.51340700 |
| C | 2.17388400 | 2.30932700 | -2.52092400 |
| C | 1.28985500 | 3.36816800 | 0.48334000 |
| C | -0.79489200 | 4.12174000 | -0.49622700 |
| H | -1.19046000 | -0.02266800 | -3.07727000 |
| H | -1.63292000 | 1.27064800 | -4.22681800 |
| H | 0.07996800 | 0.79744500 | -4.02829700 |
| H | 2.98150100 | 2.80395400 | -1.97295500 |
| H | 2.57968900 | 1.83063500 | -3.41820700 |
| H | 1.43365900 | 3.05392700 | -2.82626700 |
| C | 1.40363600 | 4.57828400 | 1.16906800 |
| H | 2.04821700 | 2.60064000 | 0.61245600 |
| C | -0.67906000 | 5.32992900 | 0.19470900 |
| H | -1.65205000 | 3.93580600 | -1.13278400 |
| C | 0.41820400 | 5.56151800 | 1.02891400 |
| H | 2.25883700 | 4.75181200 | 1.81747100 |
| H | -1.44775600 | 6.09081700 | 0.08380900 |
| H | 0.50466900 | 6.50130500 | 1.56826100 |
| P | -0.76860500 | -2.18579500 | -0.77697400 |
| O | 0.13465100 | -3.29142600 | 0.11674800 |
| O | -0.15420800 | -2.50406700 | -2.30492800 |
| C | -2.33869800 | -3.12289200 | -0.99599600 |
| C | 0.37687500 | -4.60545600 | -0.39570600 |
| C | 1.16073500 | -1.99827600 | -2.57416000 |
| C | -3.17726700 | -3.21645900 | 0.12712400 |
| C | -2.73952200 | -3.71942400 | -2.19954700 |
| H | 0.81764800 | -5.18778100 | 0.41720000 |
| H | -0.55339800 | -5.08849100 | -0.71782500 |
| H | 1.07499100 | -4.57792800 | -1.23844700 |
| H | 1.22089700 | -0.92400200 | -2.37598000 |
| H | 1.91202700 | -2.50717900 | -1.95801000 |
| H | 1.36192300 | -2.19174500 | -3.63158900 |
| C | -4.39436400 | -3.89402100 | 0.04723400 |
| H | -2.88983900 | -2.72474700 | 1.05094300 |
| C | -3.96348600 | -4.38910100 | -2.28159300 |
| H | -2.09421800 | -3.65131200 | -3.06857600 |
| C | -4.79252400 | -4.47810800 | -1.16044800 |
| H | -5.03631600 | -3.95760900 | 0.92208300 |
| H | -4.26971200 | -4.84052700 | -3.22184100 |
| H | -5.74531000 | -4.99692600 | -1.22677900 |
| P | -0.84516000 | 0.28109400 | 2.16822800 |
| O | 0.65047200 | 0.85360100 | 2.59062900 |
| O | -1.18861900 | -0.83989600 | 3.35618500 |
| C | -1.95589100 | 1.56692400 | 2.88577300 |
| C | 1.04658300 | 0.99174300 | 3.95799200 |
| C | -0.65294200 | -2.16081700 | 3.22967200 |
| C | -1.59110200 | 2.91655700 | 2.78996800 |
| C | -3.19841000 | 1.23435400 | 3.44480400 |
| H | 1.22830000 | 0.00928900 | 4.40490600 |
| H | 1.97796900 | 1.56350300 | 3.95385000 |
| H | 0.29145800 | 1.52941500 | 4.54397000 |
| H | -1.28753700 | -2.82431100 | 3.82550900 |
| H | -0.64311600 | -2.49011400 | 2.18664300 |
| H | 0.37237500 | -2.18711200 | 3.60539300 |
| C | -2.44817700 | 3.91614800 | 3.25303000 |
| H | -0.63797800 | 3.18688800 | 2.35062600 |
| C | -4.05517900 | 2.23471100 | 3.91031400 |
| H | -3.48919200 | 0.19239300 | 3.51711200 |
| C | -3.68208800 | 3.57921100 | 3.81544000 |
| H | -2.14838600 | 4.95748400 | 3.16866400 |
| H | -5.01170000 | 1.96416000 | 4.35148200 |
| H | -4.35003200 | 4.35763000 | 4.17587000 |
| P | -2.98325700 | 0.46595100 | -0.29468700 |
| O | -3.09835800 | 2.08751600 | -0.00258400 |
| O | -3.97485000 | -0.29728800 | 0.78922600 |
| C | -3.97908800 | 0.34300000 | -1.84738200 |
| C | -4.30456600 | 2.82618100 | 0.19904100 |
| C | -5.38801500 | -0.13522300 | 0.94119700 |
| C | -4.65984700 | -0.84031000 | -2.17309100 |
| C | -3.97511600 | 1.39905200 | -2.77298900 |
| H | -4.04439300 | 3.88187900 | 0.08448600 |
| H | -5.07501800 | 2.56365900 | -0.53556100 |
| H | -4.67754600 | 2.66287300 | 1.21345700 |
| H | -5.58378100 | 0.50419500 | 1.80817200 |
| H | -5.85711100 | 0.29646900 | 0.05172800 |
| H | -5.81263600 | -1.12893300 | 1.11479700 |
| C | -5.31877800 | -0.96599000 | -3.39714400 |
| H | -4.68239700 | -1.66322100 | -1.46856400 |
| C | -4.64015800 | 1.27545000 | -3.99393600 |
| H | -3.43075100 | 2.30864400 | -2.54039600 |
| C | -5.31186000 | 0.09105600 | -4.31075300 |
| H | -5.83613500 | -1.89223000 | -3.63283600 |
| H | -4.63157700 | 2.10274800 | -4.69916700 |
| H | -5.82564100 | -0.00668800 | -5.26352000 |
| C | 8.11840500 | 1.53496000 | -2.63100700 |
| H | 8.45051400 | 2.50486800 | -3.02048600 |
| H | 8.63543600 | 1.35306400 | -1.68186900 |
| H | 8.42967200 | 0.76035700 | -3.34209400 |
| C | 6.58699100 | 1.50170600 | -2.44668900 |
| C | 6.15660200 | 0.10206400 | -1.94643900 |
| C | 6.18966200 | 2.55958500 | -1.43950200 |
| C | 4.74596100 | 0.08086700 | -1.34177600 |
| H | 6.86868700 | -0.22798000 | -1.17753400 |
| H | 6.22805600 | -0.61899400 | -2.76978800 |
| C | 5.32059300 | 2.37707200 | -0.42981600 |
| H | 6.65949400 | 3.53577200 | -1.56639000 |
| C | 4.62240300 | 1.09208400 | -0.21908400 |
| H | 4.46747500 | -0.90411800 | -0.96669800 |
| H | 3.99368800 | 0.34958300 | -2.09343700 |
| H | 5.09912500 | 3.16708200 | 0.28379600 |
| C | 5.91535400 | 1.83930100 | -3.79948400 |
| H | 6.18097300 | 2.85094900 | -4.12766600 |
| H | 6.24797500 | 1.13434200 | -4.57068700 |
| H | 4.82536500 | 1.78491500 | -3.73175400 |
| O | 3.97223700 | 0.88643400 | 0.80276700 |
| C | 4.13869000 | -3.08074500 | 1.27873600 |
| C | 3.36127100 | -2.90315500 | 2.64260200 |
| O | 3.25130400 | -2.43685800 | 0.32151600 |
| O | 2.51424900 | -1.75111300 | 2.38119000 |
| C | 5.47111300 | -2.32143300 | 1.24124500 |
| H | 6.19700900 | -2.75403300 | 1.93705200 |
| H | 5.88991900 | -2.38067700 | 0.23149200 |
| H | 5.32219400 | -1.26647600 | 1.48615000 |
| C | 4.34408300 | -4.52627700 | 0.83296200 |

| | | | |
|---|---|---|---|
| H | 4.86001800 | -4.53944100 | -0.13275500 |
| H | 4.95833100 | -5.07430300 | 1.55608700 |
| H | 3.39159300 | -5.04719300 | 0.71345500 |
| C | 2.43170700 | -4.07871200 | 2.96806100 |
| H | 2.99448700 | -5.00073400 | 3.14440900 |
| H | 1.86283700 | -3.84951400 | 3.87372300 |
| H | 1.71869000 | -4.23965600 | 2.15612500 |
| C | 4.24018700 | -2.58078000 | 3.84901300 |
| H | 3.60761200 | -2.44187100 | 4.73175500 |
| H | 4.93780800 | -3.40067900 | 4.05371800 |
| H | 4.81027100 | -1.66215600 | 3.69524100 |
| B | 2.41500400 | -1.59795100 | 1.02276200 |

## TS VII-XII

E = -5397.210579

$G_{Corr}$= 1.005622

| | | | |
|---|---|---|---|
| Co | 0.57853100 | -0.00677500 | 0.16425600 |
| H | -1.04765100 | 0.50703100 | 0.29403600 |
| P | 1.12354600 | 1.46209900 | 1.68494400 |
| O | 2.36958300 | 1.09150800 | 2.73262500 |
| O | -0.06530300 | 1.86511600 | 2.76707300 |
| C | 1.75608400 | 3.10657200 | 1.16485100 |
| C | 2.14716400 | -0.03548700 | 3.59360400 |
| C | 0.19411600 | 2.84971000 | 3.78441500 |
| C | 0.84233300 | 4.00037200 | 0.58568400 |
| C | 3.11176300 | 3.45655600 | 1.22111800 |
| H | 1.81433300 | -0.90855700 | 3.02314400 |
| H | 3.09969800 | -0.25955400 | 4.07524100 |
| H | 1.39652500 | 0.20670800 | 4.35393400 |
| H | 0.01138500 | 3.85539800 | 3.39235400 |
| H | -0.49653700 | 2.64416500 | 4.60464800 |
| H | 1.22424300 | 2.78177200 | 4.14618300 |
| C | 1.27590000 | 5.22246500 | 0.06976900 |
| H | -0.20769700 | 3.73613500 | 0.52286600 |
| C | 3.54668000 | 4.67403500 | 0.69368400 |
| H | 3.82299300 | 2.77296000 | 1.66948200 |
| C | 2.63217200 | 5.55834400 | 0.11437500 |
| H | 0.55741100 | 5.90599500 | -0.37434800 |
| H | 4.60139900 | 4.93366100 | 0.73675500 |
| H | 2.97344000 | 6.50451500 | -0.29684400 |
| P | -0.08805800 | -2.02018700 | 0.75883100 |
| O | -1.60775100 | -2.32299800 | 0.21548500 |
| O | -0.17918500 | -2.41328600 | 2.37667900 |
| C | 0.78669000 | -3.55515500 | 0.24499200 |
| C | -2.30912200 | -3.54881800 | 0.47454000 |
| C | -0.91374300 | -1.54161300 | 3.24459900 |
| C | 1.11243700 | -3.67588100 | -1.11505300 |
| C | 1.10823900 | -4.60081100 | 1.12220000 |
| H | -3.27627100 | -3.41835300 | -0.00961500 |
| H | -1.77069300 | -4.40340900 | 0.04914600 |
| H | -2.43294400 | -3.70404300 | 1.55271500 |
| H | -0.81699900 | -0.49507700 | 2.93991800 |
| H | -1.97411600 | -1.81633400 | 3.24485800 |
| H | -0.50648800 | -1.67130000 | 4.25090500 |
| C | 1.76704300 | -4.81487300 | -1.58657900 |
| H | 0.89200100 | -2.86062100 | -1.79350400 |
| C | 1.77081500 | -5.73641000 | 0.65057000 |
| H | 0.85051700 | -4.51583400 | 2.17176900 |
| C | 2.10519300 | -5.84433400 | -0.70210700 |
| H | 2.01690200 | -4.89651300 | -2.64111300 |
| H | 2.02658100 | -6.53685100 | 1.33981600 |
| H | 2.62389000 | -6.72702500 | -1.06648400 |
| P | 0.45171800 | 0.65825600 | -1.97131600 |
| O | -0.58644200 | 1.92944900 | -2.10379200 |
| O | 0.00904200 | -0.39173000 | -3.15940000 |
| C | 1.87636800 | 1.37252900 | -2.90859200 |
| C | -1.22900400 | 2.23285400 | -3.34863100 |
| C | -1.03543700 | -1.36438200 | -2.96649900 |
| C | 2.30218100 | 2.66322800 | -2.56166600 |
| C | 2.53790300 | 0.69022700 | -3.93708400 |
| H | -1.99843100 | 1.48585700 | -3.56201600 |
| H | -1.69883200 | 3.21090600 | -3.22020000 |
| H | -0.50612000 | 2.27994100 | -4.17137400 |
| H | -0.71216000 | -2.28290800 | -3.46630200 |
| H | -1.22318400 | -1.54953500 | -1.90924300 |
| H | -1.95884800 | -0.99698700 | -3.41728500 |
| C | 3.36830000 | 3.26178200 | -3.23281900 |
| H | 1.80447600 | 3.19950700 | -1.76158500 |
| C | 3.60633000 | 1.29032900 | -4.61122500 |
| H | 2.20642100 | -0.30415800 | -4.21443700 |
| C | 4.02390700 | 2.57723000 | -4.26081400 |
| H | 3.68664500 | 4.26127500 | -2.94810100 |
| H | 4.10600500 | 0.75478000 | -5.41471000 |
| H | 4.85431900 | 3.04299000 | -4.78516000 |
| P | 2.71603700 | -0.50121300 | -0.16900800 |
| O | 3.51621600 | 0.91638900 | -0.32275400 |
| O | 2.87315300 | -1.32436700 | -1.57414000 |
| C | 3.86048900 | -1.42323900 | 0.94412100 |
| C | 4.81443500 | 1.08046500 | -0.90732000 |
| C | 4.07635400 | -1.83611900 | -2.16722200 |
| C | 3.95581100 | -2.82226800 | 0.88577300 |
| C | 4.66282100 | -0.72582800 | 1.86205200 |
| H | 5.11976000 | 2.10255300 | -0.67557000 |
| H | 5.53922600 | 0.37559100 | -0.48535100 |
| H | 4.75199800 | 0.96317600 | -1.99156700 |
| H | 4.31087800 | -1.22900400 | -3.04519200 |
| H | 4.91934000 | -1.82714200 | -1.47180800 |
| H | 3.87230700 | -2.86751900 | -2.46687400 |
| C | 4.81094800 | -3.51049500 | 1.74746900 |
| H | 3.38144700 | -3.37799900 | 0.15708300 |
| C | 5.52378700 | -1.41525000 | 2.71630400 |
| H | 4.59580000 | 0.35411600 | 1.91829700 |
| C | 5.59387400 | -2.81018500 | 2.66748300 |
| H | 4.86415100 | -4.59405400 | 1.69117900 |
| H | 6.13864700 | -0.86186400 | 3.42098400 |
| H | 6.26062200 | -3.34648200 | 3.33719800 |
| C | -3.71105000 | 2.83022300 | 3.75452800 |
| H | -4.49471100 | 2.40404700 | 4.39333300 |
| H | -2.74022000 | 2.55977900 | 4.18390700 |
| H | -3.80621000 | 3.92248600 | 3.78744300 |
| C | -3.83783000 | 2.32094700 | 2.30717600 |
| C | -2.74608400 | 2.96422400 | 1.42789300 |
| C | -3.67284800 | 0.81904400 | 2.27385600 |
| C | -2.61785900 | 2.30693500 | 0.05416000 |
| H | -1.79552500 | 2.88010300 | 1.95351700 |
| H | -2.95986700 | 4.03425200 | 1.29985300 |
| C | -3.08526200 | 0.12925500 | 1.28454600 |
| H | -4.15155400 | 0.26332800 | 3.08213200 |
| C | -2.53238700 | 0.78140600 | 0.07815000 |
| H | -1.78390400 | 2.70487800 | -0.51982500 |
| H | -3.52107300 | 2.48619800 | -0.54232000 |
| H | -3.08304000 | -0.94971800 | 1.26472100 |
| C | -5.25043700 | 2.65413300 | 1.76980600 |
| H | -6.02327300 | 2.19425900 | 2.39816400 |
| H | -5.41226500 | 3.74001200 | 1.77722600 |
| H | -5.38152900 | 2.27622000 | 0.75241000 |
| O | -2.76087600 | 0.22308500 | -1.09148900 |
| C | -6.36467800 | -0.29019700 | -1.14603100 |
| C | -5.81565900 | -1.69494200 | -0.64979700 |
| O | -5.19663300 | 0.52374800 | -1.16027700 |
| O | -4.43952900 | -1.67198200 | -1.02478300 |
| C | -6.94424400 | -0.34379000 | -2.57570900 |
| H | -7.88237000 | -0.90857000 | -2.62115900 |
| H | -7.14133600 | 0.68174300 | -2.90704000 |
| H | -6.23339400 | -0.78952200 | -3.27616700 |
| C | -7.41209100 | 0.34092700 | -0.22435200 |
| H | -7.71214200 | 1.31637900 | -0.62418200 |
| H | -8.30697100 | -0.28966600 | -0.15771800 |
| H | -7.02162200 | 0.49890400 | 0.78243200 |
| C | -5.90983500 | -1.86643800 | 0.87628300 |
| H | -6.94533800 | -2.00481400 | 1.20979200 |

| | | | |
|---|---|---|---|
| H | -5.33576100 | -2.75262800 | 1.17004400 |
| H | -5.48806100 | -1.00384300 | 1.39236300 |
| C | -6.48853900 | -2.90436800 | -1.31483400 |
| H | -6.05632300 | -3.82951700 | -0.91499400 |
| H | -7.56788700 | -2.92327900 | -1.11844100 |
| H | -6.32958300 | -2.90247400 | -2.39591200 |
| B | -4.09931400 | -0.35625500 | -1.57850600 |
| H | -3.91725500 | -0.39349000 | -2.79539000 |

# XII[3]

E = -5397.255153

$G_{Corr}$= 0.999779

| | | | |
|---|---|---|---|
| Co | 0.78179300 | 0.16675000 | -0.07435100 |
| H | -1.70964500 | -0.60900300 | -0.65886100 |
| P | 1.15635200 | -1.24372900 | -1.86456400 |
| O | 2.47158100 | -0.87252000 | -2.80539300 |
| O | -0.07561800 | -1.34720300 | -2.95461000 |
| C | 1.55604900 | -2.98964300 | -1.51946500 |
| C | 2.46768200 | 0.43889300 | -3.39729900 |
| C | 0.04216600 | -2.15449700 | -4.14382400 |
| C | 0.48862700 | -3.86034500 | -1.24662900 |
| C | 2.87449600 | -3.45552100 | -1.42820400 |
| H | 2.20334000 | 1.20350300 | -2.65903500 |
| H | 3.47778300 | 0.62697500 | -3.76156100 |
| H | 1.75811300 | 0.47981500 | -4.23098900 |
| H | 0.04095400 | -3.21815400 | -3.88866100 |
| H | -0.82925500 | -1.92306200 | -4.75753400 |
| H | 0.95997000 | -1.90981600 | -4.68712200 |
| C | 0.74063100 | -5.18992400 | -0.90609600 |
| H | -0.53389100 | -3.49752300 | -1.29210100 |
| C | 3.12163700 | -4.78395000 | -1.07829500 |
| H | 3.69822300 | -2.77974500 | -1.62714500 |
| C | 2.05733600 | -5.65267900 | -0.81986300 |
| H | -0.08922500 | -5.86033300 | -0.70140000 |
| H | 4.14530600 | -5.14192100 | -1.00880600 |
| H | 2.25297900 | -6.68639500 | -0.54822500 |
| P | 0.07346000 | 2.28901600 | -0.75603500 |
| O | -1.35748800 | 2.42316500 | -0.00066500 |
| O | -0.12529200 | 2.81874700 | -2.29774100 |
| C | 1.05666900 | 3.68905800 | -0.11619300 |
| C | -2.14432200 | 3.62388100 | 0.14031200 |
| C | -0.88646500 | 1.99346000 | -3.20736500 |
| C | 1.28639300 | 3.70390100 | 1.26972500 |
| C | 1.61370500 | 4.68966700 | -0.92231500 |
| H | -3.02146700 | 3.30736500 | 0.70761200 |
| H | -1.57624100 | 4.39034500 | 0.67845100 |
| H | -2.43612400 | 4.00376200 | -0.84437900 |
| H | -0.56726600 | 0.94859800 | -3.15729800 |
| H | -1.95181300 | 2.04366700 | -2.96888300 |
| H | -0.70483600 | 2.39259300 | -4.20733300 |
| C | 2.05427800 | 4.71698100 | 1.84344300 |
| H | 0.87544600 | 2.91630800 | 1.89489800 |
| C | 2.38963100 | 5.69817600 | -0.34559100 |
| H | 1.43760300 | 4.67790300 | -1.99270300 |
| C | 2.60911500 | 5.71482200 | 1.03439400 |
| H | 2.22385900 | 4.72545300 | 2.91650100 |
| H | 2.82139900 | 6.47196000 | -0.97435500 |
| H | 3.21252300 | 6.50127800 | 1.47925000 |
| P | 0.29095500 | -1.26067400 | 1.76870300 |
| O | -0.74243400 | -2.45191600 | 1.35877800 |
| O | -0.28553300 | -0.47230600 | 3.07413100 |
| C | 1.61508500 | -2.18608100 | 2.63842100 |
| C | -1.62119100 | -3.02883100 | 2.34664700 |
| C | -0.94196500 | 0.81119000 | 2.95972800 |
| C | 1.99561000 | -3.45036500 | 2.16928100 |
| C | 2.31377200 | -1.60975000 | 3.70918100 |
| H | -2.39993300 | -2.30345400 | 2.59078900 |
| H | -2.06344500 | -3.91043400 | 1.87864700 |

| | | | |
|---|---|---|---|
| H | -1.06313900 | -3.32843700 | 3.24134400 |
| H | -0.32436300 | 1.54236100 | 3.49310900 |
| H | -1.08595700 | 1.09851100 | 1.91830100 |
| H | -1.92850500 | 0.72526100 | 3.41425300 |
| C | 3.05306800 | -4.13506100 | 2.76997700 |
| H | 1.46436500 | -3.89962400 | 1.33816900 |
| C | 3.36686900 | -2.29960900 | 4.31427100 |
| H | 2.02201300 | -0.63111700 | 4.07478800 |
| C | 3.73867100 | -3.56399400 | 3.84539700 |
| H | 3.33710200 | -5.11550400 | 2.39710900 |
| H | 3.89102300 | -1.85465200 | 5.15637300 |
| H | 4.55874400 | -4.09966800 | 4.31635300 |
| P | 3.04501000 | 0.41806500 | 0.46088400 |
| O | 3.68146400 | -1.09034300 | 0.47357300 |
| O | 3.12029900 | 1.05406600 | 1.97317500 |
| C | 4.32401500 | 1.36526400 | -0.45371700 |
| C | 4.95819900 | -1.47061200 | 1.00983800 |
| C | 4.31403500 | 1.25987500 | 2.74733100 |
| C | 4.46229000 | 2.74669700 | -0.24881600 |
| C | 5.07869600 | 0.74172700 | -1.46074100 |
| H | 5.22270900 | -2.41743900 | 0.53404200 |
| H | 5.72924900 | -0.72426100 | 0.79125000 |
| H | 4.86797000 | -1.62395300 | 2.08716700 |
| H | 4.47610200 | 0.39199500 | 3.39308600 |
| H | 5.18938300 | 1.42256200 | 2.11107300 |
| H | 4.14636400 | 2.14882900 | 3.36004300 |
| C | 5.34650500 | 3.48936600 | -1.03222000 |
| H | 3.88138900 | 3.24290500 | 0.52060500 |
| C | 5.96623700 | 1.48610500 | -2.23872800 |
| H | 4.95548400 | -0.32082500 | -1.64519700 |
| C | 6.10091600 | 2.86141400 | -2.02656300 |
| H | 5.43978900 | 4.55863100 | -0.86506400 |
| H | 6.54999300 | 0.99362300 | -3.01171700 |
| H | 6.78874500 | 3.44085300 | -2.63621100 |
| C | -3.96500500 | -1.20523200 | -4.48817400 |
| H | -4.64195600 | -0.53112700 | -5.02877200 |
| H | -2.93924100 | -0.85177800 | -4.65269700 |
| H | -4.05856800 | -2.20443700 | -4.93367700 |
| C | -4.29666400 | -1.24165000 | -2.98308800 |
| C | -3.35679800 | -2.24448400 | -2.27127400 |
| C | -4.11099600 | 0.14231200 | -2.39150700 |
| C | -3.30710600 | -2.04428900 | -0.75250300 |
| H | -2.34358300 | -2.11221200 | -2.66899100 |
| H | -3.66501300 | -3.26991400 | -2.52069100 |
| C | -3.47059400 | 0.40309500 | -1.24700100 |
| H | -4.58932000 | 0.95861400 | -2.93828000 |
| C | -2.78833800 | -0.64138900 | -0.39515100 |
| H | -2.65473400 | -2.79100300 | -0.28756600 |
| H | -4.29900800 | -2.14414700 | -0.30344700 |
| H | -3.41603800 | 1.41162500 | -0.85960000 |
| C | -5.77442300 | -1.65899500 | -2.80456800 |
| H | -6.44565300 | -0.92498000 | -3.26850300 |
| H | -5.96468500 | -2.63362500 | -3.27390500 |
| H | -6.03656700 | -1.72404300 | -1.74443400 |
| O | -2.84176500 | -0.38881200 | 0.98722900 |
| C | -6.36478300 | 0.26568900 | 1.53388500 |
| C | -5.73639700 | 1.72813300 | 1.42997200 |
| O | -5.28736400 | -0.58455300 | 1.19442900 |
| O | -4.33946500 | 1.51757300 | 1.56253300 |
| C | -6.84310300 | -0.08415600 | 2.96065900 |
| H | -7.71363300 | 0.50712200 | 3.26875100 |
| H | -7.12146900 | -1.14411900 | 2.98067800 |
| H | -6.04217100 | 0.06246900 | 3.68956800 |
| C | -7.52125400 | 0.00211100 | 0.56095300 |
| H | -7.87504500 | -1.02796600 | 0.68815800 |
| H | -8.36653900 | 0.67672500 | 0.74574700 |
| H | -7.20090100 | 0.11724500 | -0.47717300 |
| C | -6.00797700 | 2.39891500 | 0.06942100 |
| H | -7.06333600 | 2.67230900 | -0.05395600 |
| H | -5.41108900 | 3.31673500 | 0.00235700 |
| H | -5.71586600 | 1.74623100 | -0.75405900 |
| C | -6.20597700 | 2.69053000 | 2.53465300 |

| | | | |
|---|---|---|---|
| H | -5.72542300 | 3.66688700 | 2.39511300 |
| H | -7.29275600 | 2.84324900 | 2.50990000 |
| H | -5.92814500 | 2.32284400 | 3.52574100 |
| B | -4.04965400 | 0.04776600 | 1.69510800 |
| H | -3.82007600 | -0.23208100 | 2.89089800 |

# XIII$^3$

E = -4985.141958
$G_{Corr}$= 0.814076

| | | | |
|---|---|---|---|
| Co | -0.09186400 | -0.23221700 | -0.29148800 |
| H | -2.42402000 | -0.47490900 | -0.81258300 |
| P | -0.61171200 | 0.52390200 | 1.82807500 |
| O | 0.47611900 | 0.15803600 | 3.03122500 |
| O | -2.04125900 | -0.00776200 | 2.44920400 |
| C | -0.69752100 | 2.32868700 | 2.09910500 |
| C | 0.75107200 | -1.24020800 | 3.21778000 |
| C | -2.49763600 | 0.35525400 | 3.76373900 |
| C | -1.86405600 | 2.99317300 | 1.68877000 |
| C | 0.37392000 | 3.06696200 | 2.61906000 |
| H | 0.99281700 | -1.72500400 | 2.26614700 |
| H | 1.61283100 | -1.30896200 | 3.88248900 |
| H | -0.10975000 | -1.74510800 | 3.67036200 |
| H | -3.43580400 | -0.18159100 | 3.91617500 |
| H | -1.76720900 | 0.05743400 | 4.52276200 |
| H | -2.67460200 | 1.43297200 | 3.83101900 |
| C | -1.96117300 | 4.37989000 | 1.81355900 |
| H | -2.68953700 | 2.42845500 | 1.26654100 |
| C | 0.27702000 | 4.45467200 | 2.73249600 |
| H | 1.27649400 | 2.55519400 | 2.93213100 |
| C | -0.88970200 | 5.11282000 | 2.33248900 |
| H | -2.86857600 | 4.88685900 | 1.49772800 |
| H | 1.11112700 | 5.02250300 | 3.13618500 |
| H | -0.96287600 | 6.19326100 | 2.42232700 |
| P | -0.14130700 | -2.57308100 | -0.39971800 |
| O | -1.00789500 | -3.01909700 | -1.71710700 |
| O | -0.67192800 | -3.52198900 | 0.83404200 |
| C | 1.45111400 | -3.44404400 | -0.67556700 |
| C | -1.20964000 | -4.40276200 | -2.05445000 |
| C | -1.94635200 | -3.22060400 | 1.44535600 |
| C | 2.10026900 | -3.23105300 | -1.90340600 |
| C | 2.04012200 | -4.28783000 | 0.27523500 |
| H | -1.91496000 | -4.41362300 | -2.88687900 |
| H | -0.26652000 | -4.86971800 | -2.35857100 |
| H | -1.63211700 | -4.95016600 | -1.20623600 |
| H | -1.96190300 | -2.20191800 | 1.83983800 |
| H | -2.75724900 | -3.32988100 | 0.72080100 |
| H | -2.06737000 | -3.93929700 | 2.25900500 |
| C | 3.31065000 | -3.86685400 | -2.18087600 |
| H | 1.65442700 | -2.57702900 | -2.64756100 |
| C | 3.25464000 | -4.91944300 | -0.00305300 |
| H | 1.54118600 | -4.45929400 | 1.22310400 |
| C | 3.88965100 | -4.71405000 | -1.22984200 |
| H | 3.79987600 | -3.70385100 | -3.13724600 |
| H | 3.70365600 | -5.57332500 | 0.73948100 |
| H | 4.83244400 | -5.20953400 | -1.44510500 |
| P | -0.41763500 | 1.64798700 | -1.72821700 |
| O | -1.84712800 | 2.37607300 | -1.45122200 |
| O | -0.30554500 | 1.18885200 | -3.29286500 |
| C | 0.70462800 | 3.09826200 | -1.85247900 |
| C | -2.64391100 | 2.91072200 | -2.53213700 |
| C | -0.50906700 | -0.21772100 | -3.60233100 |
| C | 0.51381300 | 4.19134800 | -0.99624600 |
| C | 1.78986300 | 3.10296900 | -2.74034400 |
| H | -3.35906100 | 3.59494400 | -2.06855900 |
| H | -2.02214700 | 3.46207600 | -3.24703400 |
| H | -3.15263000 | 2.05820100 | -2.99373300 |
| H | -0.38539900 | -0.30120900 | -4.68555500 |
| H | 0.26973000 | -0.81337000 | -3.10941200 |
| H | -1.52734500 | -0.50607800 | -3.29079500 |
| C | 1.38806300 | 5.27834600 | -1.03497100 |
| H | -0.31944200 | 4.19280600 | -0.30290400 |
| C | 2.65947300 | 4.19606800 | -2.78462500 |
| H | 1.93947800 | 2.25919600 | -3.40529300 |
| C | 2.46038500 | 5.28625100 | -1.93161000 |
| H | 1.22796200 | 6.11946200 | -0.36544300 |
| H | 3.48709100 | 4.19955900 | -3.48967900 |
| H | 3.13779000 | 6.13557100 | -1.96560500 |
| P | 2.22155400 | 0.24312000 | -0.04450600 |
| O | 2.24215200 | 1.78908200 | 0.49827400 |
| O | 2.91691700 | 0.18030900 | -1.53423800 |
| C | 3.49765000 | -0.53009100 | 1.03129000 |
| C | 3.37689200 | 2.66402600 | 0.57479500 |
| C | 4.24357700 | 0.62727400 | -1.86274700 |
| C | 4.23646200 | -1.63374800 | 0.58157200 |
| C | 3.66255500 | -0.09061900 | 2.35563500 |
| H | 3.13552500 | 3.41583100 | 1.32941700 |
| H | 4.28526900 | 2.12852200 | 0.87011500 |
| H | 3.51970800 | 3.15735800 | -0.38854300 |
| H | 4.20719400 | 1.67918600 | -2.15981600 |
| H | 4.93585900 | 0.49892800 | -1.02493400 |
| H | 4.58367800 | 0.02014500 | -2.70501800 |
| C | 5.13317200 | -2.27837700 | 1.43481500 |
| H | 4.10855400 | -1.99370200 | -0.43327600 |
| C | 4.56017300 | -0.73569600 | 3.20666400 |
| H | 3.07444800 | 0.74443600 | 2.72180800 |
| C | 5.29869200 | -1.83060400 | 2.74735500 |
| H | 5.69413900 | -3.13479600 | 1.07209300 |
| H | 4.68277000 | -0.38440100 | 4.22771300 |
| H | 5.99607900 | -2.33391800 | 3.41146600 |
| C | -5.66490200 | -2.22825300 | 1.88321900 |
| H | -6.21974100 | -3.17182200 | 1.79709500 |
| H | -4.65136100 | -2.46304100 | 2.23114700 |
| H | -6.15401600 | -1.62059300 | 2.65661800 |
| C | -5.62642900 | -1.47237000 | 0.53865300 |
| C | -4.83664500 | -0.15498900 | 0.71803500 |
| C | -4.94213000 | -2.33413100 | -0.50760200 |
| C | -4.37559900 | 0.45719200 | -0.60780200 |
| H | -3.94877200 | -0.36457300 | 1.32222400 |
| H | -5.44731800 | 0.55785100 | 1.29376400 |
| C | -3.96105600 | -1.91015500 | -1.31539100 |
| H | -5.31652900 | -3.35870000 | -0.59024500 |
| C | -3.43351200 | -0.47792400 | -1.41674100 |
| H | -3.86776900 | 1.41248600 | -0.44287200 |
| H | -5.23422200 | 0.66003400 | -1.26236200 |
| H | -3.52334000 | -2.59030100 | -2.04337900 |
| C | -7.07950300 | -1.19062900 | 0.09355600 |
| H | -7.62479300 | -2.12687500 | -0.08230500 |
| H | -7.62134800 | -0.62470700 | 0.86349100 |
| H | -7.10193700 | -0.61301200 | -0.83626600 |
| O | -3.27315600 | -0.10832600 | -2.69555200 |

# XIII$^3_{Sep}$

E = -800.2350086
$G_{Corr}$= 0.342194

| | | | |
|---|---|---|---|
| H | 1.18798400 | 0.53726500 | -1.86087000 |
| C | 5.15021200 | -1.09612800 | -0.43049200 |
| H | 5.37733100 | -2.00139000 | 0.14784800 |
| H | 4.90905000 | -1.40273100 | -1.45530600 |
| H | 6.05957300 | -0.48088700 | -0.46340300 |
| C | 3.98100300 | -0.31191600 | 0.19962900 |
| C | 3.71878500 | 0.97286500 | -0.62531100 |
| C | 2.73460300 | -1.18063800 | 0.20525100 |
| C | 2.33573600 | 1.57654900 | -0.35852700 |
| H | 3.78898800 | 0.72190600 | -1.69410700 |
| H | 4.51489600 | 1.70398100 | -0.42455900 |
| C | 1.52985000 | -0.78808500 | -0.22269900 |
| H | 2.86806000 | -2.18936200 | 0.60427800 |

| | | | |
|---|---|---|---|
| C | 1.21786100 | 0.60182700 | -0.75255000 |
| H | 2.20708400 | 2.52028200 | -0.90288800 |
| H | 2.21094100 | 1.80600100 | 0.70774700 |
| H | 0.67258300 | -1.45612200 | -0.19384200 |
| C | 4.35933800 | 0.04439300 | 1.65582200 |
| H | 4.49078500 | -0.86405300 | 2.25747700 |
| H | 5.30055800 | 0.61006500 | 1.68882500 |
| H | 3.57904100 | 0.64712900 | 2.13177900 |
| O | -0.00573100 | 1.07776400 | -0.26758000 |
| C | -3.03991500 | 0.53351500 | 0.47864900 |
| C | -2.82952800 | -0.87745400 | -0.18610700 |
| O | -2.39434000 | 1.40120700 | -0.43714900 |
| O | -1.54872500 | -0.76869300 | -0.77411000 |
| C | -4.50536800 | 0.96054400 | 0.62190400 |
| H | -5.07052000 | 0.24886300 | 1.23794700 |
| H | -4.56018300 | 1.94449300 | 1.10374200 |
| H | -4.99090300 | 1.04044000 | -0.35462200 |
| C | -2.34784700 | 0.63757300 | 1.85486600 |
| H | -2.35913700 | 1.68770500 | 2.16952800 |
| H | -2.85131100 | 0.03963900 | 2.62583700 |
| H | -1.30390400 | 0.32686000 | 1.77160300 |
| C | -2.83851800 | -2.05364100 | 0.79836500 |
| H | -3.79085800 | -2.11784400 | 1.34151200 |
| H | -2.69409200 | -2.99495300 | 0.25351700 |
| H | -2.02666800 | -1.96038300 | 1.52474800 |
| C | -3.85921200 | -1.14431100 | -1.30429700 |
| H | -3.53276500 | -2.02024400 | -1.87676500 |
| H | -4.86856700 | -1.33853500 | -0.91839000 |
| H | -3.89992300 | -0.28855100 | -1.98508300 |
| B | -1.23647900 | 0.68288600 | -1.00034300 |
| H | -1.08729500 | 0.93319800 | -2.21113800 |

# XIV

E = -5008.574472

$G_{Corr}$= 0.810602

| | | | |
|---|---|---|---|
| Co | -0.29144500 | -0.19017500 | 0.05774500 |
| P | -0.94879800 | 1.16206000 | 1.60203800 |
| O | -0.22394000 | 1.08935300 | 3.10713000 |
| O | -2.56110800 | 1.17042500 | 2.02153200 |
| C | -0.70501300 | 2.97505600 | 1.36220900 |
| C | -0.14939000 | -0.20410800 | 3.71599500 |
| C | -3.05127000 | 2.01090700 | 3.07130500 |
| C | -1.56544200 | 3.62905200 | 0.46754900 |
| C | 0.30397200 | 3.71100100 | 1.99637100 |
| H | 0.26257900 | -0.93452200 | 3.01427200 |
| H | 0.51383300 | -0.11250000 | 4.57839500 |
| H | -1.14213600 | -0.53411800 | 4.04601800 |
| H | -4.08768800 | 1.71080900 | 3.24722800 |
| H | -2.46935400 | 1.87444100 | 3.98867600 |
| H | -3.02178900 | 3.06732300 | 2.78141900 |
| C | -1.40427100 | 4.98920700 | 0.19579800 |
| H | -2.34422400 | 3.06621400 | -0.03480100 |
| C | 0.47043600 | 5.06921000 | 1.71897200 |
| H | 0.95763300 | 3.21874500 | 2.70643600 |
| C | -0.37905500 | 5.71119600 | 0.81329800 |
| H | -2.07623400 | 5.48304800 | -0.50162400 |
| H | 1.26098000 | 5.62795000 | 2.21409400 |
| H | -0.24792800 | 6.76827000 | 0.59661700 |
| P | -0.00586400 | -2.31255700 | 0.05210700 |
| O | -1.21745900 | -3.20544600 | -0.68400400 |
| O | 0.20655000 | -3.18672600 | 1.46336400 |
| C | 1.44759500 | -3.05211400 | -0.82820100 |
| C | -1.10195200 | -4.62192900 | -0.84122200 |
| C | -0.88149300 | -3.27888100 | 2.39072800 |
| C | 1.73542700 | -2.55969400 | -2.10934300 |
| C | 2.21427500 | -4.11057000 | -0.32020900 |
| H | -2.10981400 | -5.00026700 | -1.03111600 |
| H | -0.45110200 | -4.86870500 | -1.68775600 |
| H | -0.70220900 | -5.09418900 | 0.06218400 |
| H | -1.48734800 | -2.37137000 | 2.39267100 |
| H | -1.52818600 | -4.12550400 | 2.12743300 |
| H | -0.44612500 | -3.44961800 | 3.37950500 |
| C | 2.77330300 | -3.10598700 | -2.86411800 |
| H | 1.16149600 | -1.72791700 | -2.50100600 |
| C | 3.26533700 | -4.64813900 | -1.06917200 |
| H | 1.98935400 | -4.50577200 | 0.66468000 |
| C | 3.54658000 | -4.14852800 | -2.34288600 |
| H | 2.98603100 | -2.71092000 | -3.85440800 |
| H | 3.85891200 | -5.46149000 | -0.65929000 |
| H | 4.36177700 | -4.56866800 | -2.92641500 |
| P | -0.36367000 | 0.79470800 | -1.88287500 |
| O | -1.60373000 | 1.88727400 | -2.04326300 |
| O | -0.46163400 | -0.07830400 | -3.30670500 |
| C | 1.03612600 | 1.84411400 | -2.49116500 |
| C | -1.96574500 | 2.42421400 | -3.31603200 |
| C | -1.37027900 | -1.17788700 | -3.43999200 |
| C | 1.07180700 | 3.20180300 | -2.14111500 |
| C | 2.09950800 | 1.30712400 | -3.22919200 |
| H | -2.66199200 | 3.24498900 | -3.12145800 |
| H | -1.09654700 | 2.81199300 | -3.86005700 |
| H | -2.46298200 | 1.66530900 | -3.93027000 |
| H | -1.04266800 | -1.74334300 | -4.31775100 |
| H | -1.35722700 | -1.81965300 | -2.55718000 |
| H | -2.39197400 | -0.81654500 | -3.58992000 |
| C | 2.13851400 | 4.01041500 | -2.53641100 |
| H | 0.26246400 | 3.62548500 | -1.55606200 |
| C | 3.17209000 | 2.11332800 | -3.61851800 |
| H | 2.08949400 | 0.25737700 | -3.49973500 |
| C | 3.19297000 | 3.46898800 | -3.27697800 |
| H | 2.14668200 | 5.06203200 | -2.26061700 |
| H | 3.98719800 | 1.68429800 | -4.19637300 |
| H | 4.02556400 | 4.09695100 | -3.58402300 |
| P | 1.83249700 | 0.28495700 | 0.42281700 |
| O | 2.08461600 | 1.90617100 | 0.50016200 |
| O | 2.80184600 | -0.29314900 | -0.76570600 |
| C | 2.73028100 | -0.26765500 | 1.93866400 |
| C | 3.36121000 | 2.54332100 | 0.41362200 |
| C | 4.22885700 | -0.31528100 | -0.83744400 |
| C | 3.18583400 | -1.59066800 | 2.04134600 |
| C | 2.88567600 | 0.59009700 | 3.03718700 |
| H | 3.21487400 | 3.56769900 | 0.76552600 |
| H | 4.10979300 | 2.04755700 | 1.04238900 |
| H | 3.69633300 | 2.56668000 | -0.62623800 |
| H | 4.56495000 | 0.49823700 | -1.48801500 |
| H | 4.69656200 | -0.22233600 | 0.14760200 |
| H | 4.50597600 | -1.27643200 | -1.27963900 |
| C | 3.77526200 | -2.04858100 | 3.22015800 |
| H | 3.08525700 | -2.26308400 | 1.19736100 |
| C | 3.47996500 | 0.13402000 | 4.21509500 |
| H | 2.52411800 | 1.60982000 | 2.97478100 |
| C | 3.92171800 | -1.18798600 | 4.31167500 |
| H | 4.12242500 | -3.07652800 | 3.28419900 |
| H | 3.59357800 | 0.81036900 | 5.05830100 |
| H | 4.37953600 | -1.54460300 | 5.23051000 |
| B | -2.23222000 | -0.67903000 | -0.06148300 |
| O | -2.92705500 | -1.37984300 | 0.91973800 |
| O | -3.09379700 | -0.35596700 | -1.10214500 |
| C | -4.28315800 | -1.62675800 | 0.48588500 |
| C | -4.45944000 | -0.60085000 | -0.69688800 |
| C | -5.21108800 | -1.39789800 | 1.67835500 |
| C | -4.35809000 | -3.09044800 | 0.03445400 |
| C | -5.23129800 | -1.12996300 | -1.90509900 |
| C | -5.04009000 | 0.75156800 | -0.26048400 |
| H | -6.26278300 | -1.49296000 | 1.38417500 |
| H | -5.00475800 | -2.14669700 | 2.45104200 |
| H | -5.05197400 | -0.40976500 | 2.11493800 |
| H | -4.04058200 | -3.73255800 | 0.86264800 |
| H | -5.37646500 | -3.37250800 | -0.25445900 |
| H | -3.68083300 | -3.26663500 | -0.80242300 |
| H | -6.26095100 | -1.38667300 | -1.63060300 |

| | | | |
|---|---|---|---|
| H | -5.26827500 | -0.35702100 | -2.68033900 |
| H | -4.75266600 | -2.01366800 | -2.33325900 |
| H | -4.97348400 | 1.44899000 | -1.10235900 |
| H | -6.09105200 | 0.66619500 | 0.03687100 |
| H | -4.46072700 | 1.16364600 | 0.56820600 |

# XV

E = -798.9498486
$G_{Corr}$= 0.320432

| | | | |
|---|---|---|---|
| C | 5.46665600 | 0.11381300 | -0.51961700 |
| H | 6.16327300 | 0.04691300 | 0.32531300 |
| H | 5.58879800 | -0.78854700 | -1.13024800 |
| H | 5.75772100 | 0.97954900 | -1.12754500 |
| C | 4.01508400 | 0.25514500 | -0.02401000 |
| C | 3.06508300 | 0.33969500 | -1.24529600 |
| C | 3.63254000 | -0.94571500 | 0.81374400 |
| C | 1.57371600 | 0.26763600 | -0.87038300 |
| H | 3.29519000 | -0.49815500 | -1.91632700 |
| H | 3.26269800 | 1.26303600 | -1.80410200 |
| C | 2.33704900 | -1.44321400 | 0.82729000 |
| H | 4.38933800 | -1.38553800 | 1.45877600 |
| C | 1.32340300 | -0.87904000 | 0.06465600 |
| H | 0.96218900 | 0.14441500 | -1.77392000 |
| H | 1.23660900 | 1.20872400 | -0.41392600 |
| H | 2.08048700 | -2.28947200 | 1.46090100 |
| C | 3.90182300 | 1.53774500 | 0.83698800 |
| H | 4.57643100 | 1.48531300 | 1.69956500 |
| H | 4.17069400 | 2.42483500 | 0.24796800 |
| H | 2.88465300 | 1.67313200 | 1.21909400 |
| O | 0.07117100 | -1.44818300 | 0.13116800 |
| C | -2.63673200 | 0.90669500 | 0.25455300 |
| C | -3.32549600 | -0.42834700 | -0.22159700 |
| O | -1.22330500 | 0.61349600 | 0.04588500 |
| O | -2.30313600 | -1.42500600 | 0.06857900 |
| C | -2.99994800 | 2.14617400 | -0.55609300 |
| H | -4.07554400 | 2.34431400 | -0.49278900 |
| H | -2.46837100 | 3.01624800 | -0.15760300 |
| H | -2.72512000 | 2.03280000 | -1.60703300 |
| C | -2.81313800 | 1.17438700 | 1.75341100 |
| H | -2.14216700 | 1.98639300 | 2.04967800 |
| H | -3.84063600 | 1.46803200 | 1.99004900 |
| H | -2.55797200 | 0.28847100 | 2.34359600 |
| C | -4.59772600 | -0.80008500 | 0.53273200 |
| H | -5.36016900 | -0.02376500 | 0.40517200 |
| H | -4.99793300 | -1.73996200 | 0.13945200 |
| H | -4.40679800 | -0.93313100 | 1.59980500 |
| C | -3.56720400 | -0.47171400 | -1.73506000 |
| H | -3.84721500 | -1.49032000 | -2.01990700 |
| H | -4.37247000 | 0.20722300 | -2.03276400 |
| H | -2.66048500 | -0.20102400 | -2.28575600 |
| B | -1.10434000 | -0.75619400 | 0.07791800 |

# XVI

E = -4204.979099
$G_{Corr}$= 0.635653

| | | | |
|---|---|---|---|
| Co | 0.37972100 | -0.08108600 | -0.28760600 |
| P | 0.54587200 | 1.04188700 | 1.47371100 |
| O | 0.40360700 | 2.70182500 | 1.50879900 |
| O | 1.89694800 | 0.80175500 | 2.41524100 |
| C | -0.79610600 | 0.63911100 | 2.65625700 |
| C | 1.12382200 | 3.40796300 | 0.48753700 |
| C | 2.02523700 | 1.44244700 | 3.69065700 |
| C | -0.82371000 | -0.66436200 | 3.17821600 |
| C | -1.82766800 | 1.53069200 | 2.97697100 |
| H | 1.07252800 | 2.87111600 | -0.46584500 |
| H | 0.65267200 | 4.38702300 | 0.37996100 |
| H | 2.17620800 | 3.52582000 | 0.77129500 |
| H | 3.03515600 | 1.22553500 | 4.04738400 |
| H | 1.89097700 | 2.52578700 | 3.60151300 |
| H | 1.29312000 | 1.04571400 | 4.40361500 |
| C | -1.87071400 | -1.06727800 | 4.00833400 |
| H | -0.03882700 | -1.36697100 | 2.90906000 |
| C | -2.87872000 | 1.12233300 | 3.80143200 |
| H | -1.80067600 | 2.54153500 | 2.58424700 |
| C | -2.90400000 | -0.17652800 | 4.31633000 |
| H | -1.88462900 | -2.07788800 | 4.40837000 |
| H | -3.67641600 | 1.81982500 | 4.04460400 |
| H | -3.72328000 | -0.49313300 | 4.95646800 |
| P | 0.08117400 | -2.12858400 | -0.55752400 |
| O | 0.65109700 | -2.95102400 | 0.76714200 |
| O | 0.62235300 | -3.05343300 | -1.83460900 |
| C | -1.66717700 | -2.71204300 | -0.60711100 |
| C | 0.54786100 | -4.37869700 | 0.84417700 |
| C | 1.77044600 | -2.60110400 | -2.55623900 |
| C | -2.42408000 | -2.66867700 | 0.57268800 |
| C | -2.30599400 | -3.02965400 | -1.81357200 |
| H | 1.05426400 | -4.67799600 | 1.76492100 |
| H | -0.49979100 | -4.70036000 | 0.88316100 |
| H | 1.03676800 | -4.85174800 | -0.01389800 |
| H | 1.80948900 | -3.17523100 | -3.48590800 |
| H | 1.68721700 | -1.53173600 | -2.78765100 |
| H | 2.68505500 | -2.76681600 | -1.97772200 |
| C | -3.79211200 | -2.94202400 | 0.55009600 |
| H | -1.94479800 | -2.40206000 | 1.50850000 |
| C | -3.67585400 | -3.30036100 | -1.83834200 |
| H | -1.72862500 | -3.05716300 | -2.73138100 |
| C | -4.42318200 | -3.25501000 | -0.65707800 |
| H | -4.36469800 | -2.90153700 | 1.47326200 |
| H | -4.15992300 | -3.54951200 | -2.77950200 |
| H | -5.48990600 | -3.46347500 | -0.67810900 |
| P | -1.52636300 | 0.65364800 | -1.04874000 |
| O | -2.79715900 | 0.44401400 | -0.03203600 |
| O | -1.89591500 | -0.02515300 | -2.51098000 |
| C | -1.63496300 | 2.44458400 | -1.45605600 |
| C | -4.17471500 | 0.71511600 | -0.31278500 |
| C | -3.04825700 | 0.26745400 | -3.31051800 |
| C | -1.06239200 | 2.92204700 | -2.64561200 |
| C | -2.12675900 | 3.36316100 | -0.51735100 |
| H | -4.65583200 | 0.91103100 | 0.64899500 |
| H | -4.29872300 | 1.59030200 | -0.96006100 |
| H | -4.63515100 | -0.16394800 | -0.77357700 |
| H | -3.86913700 | -0.40015600 | -3.03019200 |
| H | -3.36353400 | 1.31191100 | -3.21120000 |
| H | -2.77214100 | 0.07866400 | -4.35131500 |
| C | -0.98960200 | 4.29354400 | -2.89413300 |
| H | -0.67690700 | 2.21722000 | -3.37780400 |
| C | -2.06039700 | 4.73424900 | -0.76947700 |
| H | -2.55323400 | 3.00180500 | 0.41264700 |
| C | -1.48907200 | 5.20212700 | -1.95627000 |
| H | -0.54598900 | 4.65304200 | -3.81874300 |
| H | -2.44916600 | 5.43735800 | -0.03766800 |
| H | -1.43197300 | 6.26994900 | -2.14914200 |
| B | 2.29758400 | -0.32524100 | -0.19156600 |
| O | 2.94089300 | 0.76253500 | -0.79568100 |
| O | 3.22024500 | -1.28601500 | 0.18866300 |
| C | 4.34779800 | 0.44621200 | -0.96286700 |
| C | 4.55399400 | -0.72285500 | 0.07303000 |
| C | 5.15408000 | 1.71146500 | -0.67348500 |
| C | 4.55379100 | 0.02176000 | -2.42173800 |
| C | 5.50603900 | -1.82565200 | -0.38467500 |
| C | 4.95166500 | -0.23073600 | 1.47067800 |
| H | 6.23040800 | 1.50576200 | -0.70142300 |
| H | 4.93207100 | 2.46981800 | -1.43194200 |
| H | 4.90156500 | 2.12745900 | 0.30444800 |
| H | 4.20814600 | 0.82814500 | -3.07683300 |
| H | 5.60860100 | -0.17525300 | -2.64114900 |
| H | 3.97608700 | -0.87496300 | -2.65723000 |
| H | 6.51423500 | -1.42686600 | -0.54575100 |

| | | | |
|---|---|---|---|
| H | 5.56491500 | -2.60160600 | 0.38561400 |
| H | 5.16514600 | -2.29529500 | -1.31053400 |
| H | 4.90493700 | -1.07532600 | 2.16583000 |
| H | 5.96958000 | 0.17362700 | 1.48293700 |
| H | 4.25147400 | 0.53012200 | 1.82235400 |

## $CoP_4{}^{+}{}_{T}$

E = -4596.968604
$G_{Corr}$= 0.629992

| | | | |
|---|---|---|---|
| Co | 0.13542200 | -0.67986700 | -0.65087300 |
| P | -0.98284100 | -1.98299600 | 0.90927000 |
| O | -0.42669600 | -1.93181600 | 2.46278900 |
| O | -0.98672400 | -3.58187700 | 0.51039200 |
| C | -2.73372500 | -1.61092700 | 1.26464100 |
| C | 0.96972900 | -2.19885100 | 2.68226900 |
| C | -1.57020500 | -4.57021900 | 1.38910800 |
| C | -3.71574500 | -2.11874700 | 0.39904600 |
| C | -3.10729200 | -0.75273600 | 2.30890900 |
| H | 1.59142300 | -1.69705100 | 1.93426300 |
| H | 1.21625200 | -1.81131000 | 3.67080400 |
| H | 1.15749000 | -3.27713700 | 2.65132200 |
| H | -1.32179600 | -5.54326000 | 0.96293300 |
| H | -1.14959300 | -4.48546600 | 2.39557300 |
| H | -2.65649000 | -4.45163800 | 1.43683800 |
| C | -5.05738600 | -1.78164100 | 0.58537300 |
| H | -3.43069100 | -2.76761500 | -0.42308800 |
| C | -4.44906400 | -0.41259500 | 2.48727500 |
| H | -2.35059400 | -0.35250700 | 2.97312200 |
| C | -5.42524100 | -0.92557500 | 1.62793300 |
| H | -5.81285000 | -2.18329000 | -0.08380800 |
| H | -4.73348400 | 0.25084400 | 3.29927600 |
| H | -6.46884900 | -0.65922200 | 1.76959900 |
| P | 2.31139700 | -1.45350300 | -1.03027800 |
| O | 2.37044800 | -2.09556000 | -2.54420600 |
| O | 3.10051800 | -2.53543400 | -0.08044600 |
| C | 3.54388700 | -0.11445600 | -1.04034100 |
| C | 3.63580100 | -2.43949300 | -3.15253000 |
| C | 2.52942200 | -3.85486400 | 0.04925500 |
| C | 3.16436800 | 1.06555000 | -1.70128400 |
| C | 4.79732700 | -0.19456600 | -0.41865500 |
| H | 3.39872700 | -2.93919300 | -4.09256400 |
| H | 4.22317700 | -1.53641800 | -3.34637600 |
| H | 4.20483200 | -3.11490200 | -2.50632100 |
| H | 1.44116500 | -3.81083200 | 0.16339200 |
| H | 2.77866100 | -4.45770600 | -0.82976300 |
| H | 2.97802500 | -4.30043000 | 0.93840500 |
| C | 4.03522400 | 2.15401900 | -1.74690200 |
| H | 2.17783200 | 1.14397600 | -2.15149600 |
| C | 5.66080700 | 0.90195600 | -0.45572700 |
| H | 5.08635100 | -1.10442700 | 0.09638200 |
| C | 5.28298500 | 2.07306900 | -1.11967400 |
| H | 3.73763400 | 3.06474600 | -2.25841400 |
| H | 6.62917800 | 0.84238000 | 0.03261300 |
| H | 5.95820400 | 2.92357700 | -1.14492900 |
| P | -1.59219900 | 0.21970900 | -1.98364000 |
| O | -2.72794700 | -0.90156800 | -2.35140300 |
| O | -1.04371000 | 0.80620500 | -3.42079900 |
| C | -2.52585500 | 1.68620600 | -1.44331700 |
| C | -3.83948900 | -0.59245200 | -3.22296700 |
| C | -0.12817500 | -0.00421500 | -4.18291100 |
| C | -3.60711700 | 1.50955500 | -0.56706400 |
| C | -2.11207800 | 2.98165100 | -1.78528900 |
| H | -4.30311900 | -1.54697000 | -3.47576300 |
| H | -4.56105200 | 0.04748900 | -2.70768100 |
| H | -3.48994900 | -0.09627400 | -4.13316900 |
| H | 0.39635900 | 0.66842900 | -4.86394400 |
| H | 0.59780200 | -0.51010900 | -3.53506900 |
| H | -0.67842500 | -0.75515300 | -4.75931700 |
| C | -4.28541200 | 2.61769100 | -0.06075700 |
| H | -3.92019900 | 0.51213800 | -0.27983000 |
| C | -2.79262400 | 4.08838700 | -1.27264400 |
| H | -1.26901500 | 3.12213100 | -2.45267300 |
| C | -3.88124700 | 3.90838400 | -0.41363400 |
| H | -5.12573900 | 2.46957700 | 0.61137000 |
| H | -2.47786800 | 5.09045700 | -1.55080100 |
| H | -4.40972000 | 4.77149500 | -0.01855600 |
| P | 0.38357400 | 1.19387300 | 0.72382800 |
| O | -1.06165600 | 1.43399600 | 1.45091300 |
| O | 0.71689300 | 2.46599400 | -0.25393400 |
| C | 1.56640500 | 1.38417600 | 2.10806700 |
| C | -1.45453500 | 2.62918600 | 2.14842200 |
| C | 0.89228200 | 3.83523600 | 0.15531200 |
| C | 2.89397500 | 1.76636800 | 1.85737400 |
| C | 1.19114800 | 1.01068200 | 3.40884300 |
| H | -2.31421700 | 2.35650000 | 2.76354300 |
| H | -0.65027300 | 3.00526400 | 2.78897000 |
| H | -1.75729100 | 3.39071600 | 1.42628300 |
| H | -0.03866500 | 4.37907500 | -0.02713700 |
| H | 1.17359700 | 3.91310400 | 1.20935000 |
| H | 1.69232200 | 4.25277000 | -0.46061900 |
| C | 3.82765200 | 1.78104500 | 2.89397100 |
| H | 3.19888000 | 2.05551500 | 0.85763300 |
| C | 2.12662100 | 1.03208100 | 4.44397000 |
| H | 0.17199500 | 0.69212100 | 3.60607500 |
| C | 3.44634500 | 1.41527800 | 4.18771900 |
| H | 4.85201600 | 2.07774100 | 2.68779900 |
| H | 1.82621100 | 0.74882800 | 5.44887100 |
| H | 4.17464300 | 1.42759600 | 4.99358600 |

## XVII

E = -411.3780814
$G_{Corr}$= 0.148196

| | | | |
|---|---|---|---|
| C | 0.00000000 | 0.78759600 | -0.15405800 |
| C | 0.00000000 | -0.78759600 | -0.15405800 |
| O | 0.32897600 | 1.10229400 | 1.25287800 |
| O | -0.32897600 | -1.10229400 | 1.25287800 |
| C | 1.05439600 | 1.43429300 | -1.04441700 |
| H | 0.88793000 | 1.16245100 | -2.09285900 |
| H | 0.98934900 | 2.52351000 | -0.95977800 |
| H | 2.06410400 | 1.13115900 | -0.75991600 |
| C | -1.37811800 | 1.39972500 | -0.41950900 |
| H | -1.33783000 | 2.47198800 | -0.20616800 |
| H | -1.67889400 | 1.26392800 | -1.46338900 |
| H | -2.14017800 | 0.95265800 | 0.22602800 |
| C | -1.05439600 | -1.43429300 | -1.04441700 |
| H | -0.88793000 | -1.16245100 | -2.09285900 |
| H | -0.98934900 | -2.52351000 | -0.95977800 |
| H | -2.06410400 | -1.13115900 | -0.75991600 |
| C | 1.37811800 | -1.39972500 | -0.41950900 |
| H | 1.33783000 | -2.47198800 | -0.20616800 |
| H | 1.67889400 | -1.26392800 | -1.46338900 |
| H | 2.14017800 | -0.95265800 | 0.22602800 |
| B | 0.00000000 | 0.00000000 | 1.97638400 |

## XVIII

E = -4597.536952
$G_{Corr}$= 0.640895

| | | | |
|---|---|---|---|
| Co | 0.21532300 | 0.10948000 | -0.55174000 |
| H | 0.68290000 | 0.46905900 | -1.93701700 |
| P | 0.80577100 | 2.26194700 | -0.48382300 |
| O | 0.36369400 | 3.32200400 | 0.69495800 |
| O | 0.23248300 | 3.07682900 | -1.76853800 |
| C | 2.62417800 | 2.44369100 | -0.56409000 |
| C | 0.70949500 | 3.13852300 | 2.08151300 |

| | | | |
|---|---|---|---|
| C | 0.52173900 | 4.47752200 | -1.96199500 |
| C | 3.36928600 | 1.49474300 | -1.28474400 |
| C | 3.28643700 | 3.52408300 | 0.04119300 |
| H | -0.21187600 | 3.17841400 | 2.66465300 |
| H | 1.20924900 | 2.18320700 | 2.26297200 |
| H | 1.37515500 | 3.95165600 | 2.38369700 |
| H | 0.08107900 | 4.74145100 | -2.92398300 |
| H | 0.07199000 | 5.07460200 | -1.16477300 |
| H | 1.60266300 | 4.64966400 | -1.99212300 |
| C | 4.75636800 | 1.61455600 | -1.37854000 |
| H | 2.86498400 | 0.66691800 | -1.77487400 |
| C | 4.67407600 | 3.63958300 | -0.05495100 |
| H | 2.71971000 | 4.27899600 | 0.57526700 |
| C | 5.41041900 | 2.68304200 | -0.75917600 |
| H | 5.32390600 | 0.87412500 | -1.93494000 |
| H | 5.17913700 | 4.47793100 | 0.41605200 |
| H | 6.49050700 | 2.77398200 | -0.83003800 |
| P | -1.82258900 | 0.14079700 | -1.70247800 |
| O | -1.53132000 | -0.73909000 | -3.04940300 |
| O | -2.30172800 | 1.55154000 | -2.41234600 |
| C | -3.44009400 | -0.48084400 | -1.07755100 |
| C | -2.49234500 | -0.88632900 | -4.11398900 |
| C | -2.78194600 | 2.65081700 | -1.62655700 |
| C | -3.47040700 | -1.33792600 | 0.03157600 |
| C | -4.65246500 | -0.11119700 | -1.68634500 |
| H | -1.98882300 | -1.45736800 | -4.89519900 |
| H | -3.37444500 | -1.43400000 | -3.76638200 |
| H | -2.78862700 | 0.09225500 | -4.50022300 |
| H | -2.05646200 | 2.95085500 | -0.86633100 |
| H | -2.92638300 | 3.48237800 | -2.31833000 |
| H | -3.73878800 | 2.40276900 | -1.15210700 |
| C | -4.68300400 | -1.82517000 | 0.52090400 |
| H | -2.54377000 | -1.62773200 | 0.51063700 |
| C | -5.86517200 | -0.59665700 | -1.19611500 |
| H | -4.64399100 | 0.56114400 | -2.53871000 |
| C | -5.88215000 | -1.45423300 | -0.09209700 |
| H | -4.69189200 | -2.48911500 | 1.38083400 |
| H | -6.79622800 | -0.30497200 | -1.67363400 |
| H | -6.82753700 | -1.82853400 | 0.29017600 |
| P | 1.10866200 | -1.89240600 | -1.18594800 |
| O | 2.02198700 | -1.65265700 | -2.51929700 |
| O | 0.29512100 | -3.25427000 | -1.57927300 |
| C | 2.31570500 | -2.54823300 | 0.02769600 |
| C | 2.82979500 | -2.71855400 | -3.06613900 |
| C | -1.07039600 | -3.50789400 | -1.21025800 |
| C | 3.38185700 | -1.72658700 | 0.42713200 |
| C | 2.19289800 | -3.83412500 | 0.57171100 |
| H | 3.34425600 | -2.29260900 | -3.92834100 |
| H | 3.56244400 | -3.06625800 | -2.33048500 |
| H | 2.19609800 | -3.55099800 | -3.38309300 |
| H | -1.73630300 | -2.78379700 | -1.68018100 |
| H | -1.29916100 | -4.50843000 | -1.58204800 |
| H | -1.19014000 | -3.48151200 | -0.12450300 |
| C | 4.32685500 | -2.19673300 | 1.33867000 |
| H | 3.47993900 | -0.72254100 | 0.02705100 |
| C | 3.13215900 | -4.29592800 | 1.49725000 |
| H | 1.37483600 | -4.47473600 | 0.25965800 |
| C | 4.20262400 | -3.48158100 | 1.87602800 |
| H | 5.15614800 | -1.55924300 | 1.63222400 |
| H | 3.03384900 | -5.29488700 | 1.91274400 |
| H | 4.93664600 | -3.84591800 | 2.58914400 |
| P | -0.18686200 | -0.41097300 | 1.58842800 |
| O | 1.21170400 | -0.27157200 | 2.43974300 |
| O | -0.65423800 | -1.97753300 | 1.77373900 |
| C | -1.35680300 | 0.58482900 | 2.57069400 |
| C | 1.72777700 | -1.25287200 | 3.35679200 |
| C | -1.35317200 | -2.51879200 | 2.91385600 |
| C | -2.29741100 | 1.36150600 | 1.88205300 |
| C | -1.29219400 | 0.66535900 | 3.97186000 |
| H | 2.77741100 | -1.00377800 | 3.50873800 |
| H | 1.20073400 | -1.20888900 | 4.31614200 |
| H | 1.65774400 | -2.25616200 | 2.93139100 |
| H | -1.63255200 | -3.53622600 | 2.63576900 |
| H | -0.70857100 | -2.54686700 | 3.79642300 |
| H | -2.25086800 | -1.93350300 | 3.13216800 |
| C | -3.17319900 | 2.19649200 | 2.57532900 |
| H | -2.32693800 | 1.32595700 | 0.80133500 |
| C | -2.16630900 | 1.50082400 | 4.66561400 |
| H | -0.55152700 | 0.09547100 | 4.52235700 |
| C | -3.10791700 | 2.26635200 | 3.96869200 |
| H | -3.89453100 | 2.79650200 | 2.02853600 |
| H | -2.10939300 | 1.56179300 | 5.74841100 |
| H | -3.78274600 | 2.92094500 | 4.51245800 |

# XIX

E = -387.5145578
$G_{Corr}$= 0.146287

| | | | |
|---|---|---|---|
| C | -2.51843100 | 0.22669400 | -0.79679800 |
| H | -3.11489200 | 1.05141200 | -0.38217400 |
| H | -2.31055600 | 0.46256100 | -1.84790800 |
| H | -3.13857900 | -0.68144000 | -0.76456500 |
| C | -1.20695500 | 0.05029400 | -0.00609700 |
| C | -0.38013800 | -1.09439600 | -0.64573600 |
| C | -0.38325800 | 1.31800700 | -0.05155600 |
| C | 1.03333400 | -1.22742900 | -0.05511600 |
| H | -0.28781200 | -0.87923900 | -1.72031000 |
| H | -0.93077500 | -2.04543000 | -0.56023200 |
| C | 1.02027100 | 1.27540400 | -0.01779200 |
| H | -0.91280600 | 2.26744600 | 0.03615600 |
| C | 1.80963600 | 0.10040200 | -0.01794100 |
| H | 1.62174600 | -1.94896600 | -0.64049900 |
| H | 0.97692600 | -1.65217000 | 0.96231200 |
| H | 1.57576100 | 2.21633300 | 0.00972800 |
| C | -1.57224700 | -0.32125100 | 1.45550300 |
| H | -2.18069100 | 0.47144700 | 1.91079200 |
| H | -2.14357400 | -1.26251200 | 1.51155000 |
| H | -0.66846900 | -0.43202200 | 2.06310200 |
| O | 3.08755600 | 0.05827800 | 0.02940600 |

# XX

E = -5008.438823
$G_{Corr}$= 0.806435

| | | | |
|---|---|---|---|
| Co | -0.37620000 | -0.13423100 | 0.17487500 |
| P | -1.12419700 | 1.79475400 | 1.12989200 |
| O | -0.95711700 | 1.77184400 | 2.76675400 |
| O | -2.71641600 | 2.10123900 | 0.88095600 |
| C | -0.37382400 | 3.43107100 | 0.82161400 |
| C | -1.39752000 | 0.59060800 | 3.46842400 |
| C | -3.41210800 | 3.10087400 | 1.65480900 |
| C | -0.85983200 | 4.20516000 | -0.24428900 |
| C | 0.68642400 | 3.91566600 | 1.59990200 |
| H | -0.69754800 | -0.22864700 | 3.29301700 |
| H | -1.39801600 | 0.84547800 | 4.52942700 |
| H | -2.39971000 | 0.29365200 | 3.14793000 |
| H | -4.43645500 | 3.11422300 | 1.27944900 |
| H | -3.40892300 | 2.83240100 | 2.71477500 |
| H | -2.95868600 | 4.08756800 | 1.52356300 |
| C | -0.26680400 | 5.43123300 | -0.54842700 |
| H | -1.69081000 | 3.84162000 | -0.83662500 |
| C | 1.27687500 | 5.14166300 | 1.29233100 |
| H | 1.04799900 | 3.33607100 | 2.43987900 |
| C | 0.81038400 | 5.89532400 | 0.21127500 |
| H | -0.64655900 | 6.02237800 | -1.37671300 |
| H | 2.09775100 | 5.51168400 | 1.90033300 |
| H | 1.27595500 | 6.84683200 | -0.02938000 |
| P | -0.24548200 | -2.38499500 | -0.11220400 |
| O | -1.48844200 | -2.98580700 | -1.00447900 |
| O | -0.24328000 | -3.25898400 | 1.26934400 |
| C | 1.20975100 | -3.18315200 | -0.89134100 |

| | | | |
|---|---|---|---|
| C | -1.57849600 | -4.39441900 | -1.30315400 |
| C | -0.97336700 | -2.86170100 | 2.44707900 |
| C | 1.54676600 | -2.82357600 | -2.20409200 |
| C | 1.98515200 | -4.14027900 | -0.22089300 |
| H | -2.55812200 | -4.54449900 | -1.75983700 |
| H | -0.79168600 | -4.68824100 | -2.00391500 |
| H | -1.50151300 | -4.98866900 | -0.38787000 |
| H | -1.68626300 | -2.06677500 | 2.22579500 |
| H | -1.51227100 | -3.74239700 | 2.80546500 |
| H | -0.25158800 | -2.54093200 | 3.20315900 |
| C | 2.65613600 | -3.39404900 | -2.82769300 |
| H | 0.95727700 | -2.08895500 | -2.73895800 |
| C | 3.09847500 | -4.70626400 | -0.84695700 |
| H | 1.71347000 | -4.44760600 | 0.78237800 |
| C | 3.43987700 | -4.33104000 | -2.14842700 |
| H | 2.90928900 | -3.10514300 | -3.84409100 |
| H | 3.69364500 | -5.44558700 | -0.31826500 |
| H | 4.30663500 | -4.77135200 | -2.63316200 |
| P | 0.03764100 | 0.70204900 | -1.99426200 |
| O | -0.96066200 | 1.97616200 | -2.27002000 |
| O | -0.09361400 | -0.21310500 | -3.36412200 |
| C | 1.70768600 | 1.36649000 | -2.35307400 |
| C | -0.86997500 | 2.75564800 | -3.47960900 |
| C | -1.33099700 | -0.88690900 | -3.68070000 |
| C | 2.06143900 | 2.61413600 | -1.81758700 |
| C | 2.67399600 | 0.61330300 | -3.03517000 |
| H | -1.82317700 | 3.27765600 | -3.58223600 |
| H | -0.05513100 | 3.48135900 | -3.40268600 |
| H | -0.70703000 | 2.11053900 | -4.34768700 |
| H | -1.14261200 | -1.42851300 | -4.61008300 |
| H | -1.61846400 | -1.57495600 | -2.88563500 |
| H | -2.13307600 | -0.15924300 | -3.82780200 |
| C | 3.35651000 | 3.10823700 | -1.97719300 |
| H | 1.33167300 | 3.19962400 | -1.27134600 |
| C | 3.97168500 | 1.10532900 | -3.18499900 |
| H | 2.41558100 | -0.35449200 | -3.44799200 |
| C | 4.31604100 | 2.35355200 | -2.65741000 |
| H | 3.61258900 | 4.08097700 | -1.56644300 |
| H | 4.71240800 | 0.51600700 | -3.71865000 |
| H | 5.32573500 | 2.73626100 | -2.77856300 |
| P | 1.78885100 | 0.04466000 | 0.89193400 |
| O | 2.29437800 | 1.58788000 | 1.03260800 |
| O | 2.77472600 | -0.75546200 | -0.11880400 |
| C | 2.16819800 | -0.65582200 | 2.53963300 |
| C | 3.62670300 | 1.99716200 | 1.38479600 |
| C | 4.19808800 | -0.93637900 | -0.00557000 |
| C | 2.53469500 | -2.00316500 | 2.67467000 |
| C | 1.94545000 | 0.11845500 | 3.68984500 |
| H | 3.54752700 | 3.03313500 | 1.72025400 |
| H | 4.03665600 | 1.38031600 | 2.19089300 |
| H | 4.26875200 | 1.95226100 | 0.50180400 |
| H | 4.69504200 | -0.19282700 | -0.63360100 |
| H | 4.53967300 | -0.85063400 | 1.03024600 |
| H | 4.41057200 | -1.94014700 | -0.37889700 |
| C | 2.67678100 | -2.56803800 | 3.94288100 |
| H | 2.70858700 | -2.60674000 | 1.79115200 |
| C | 2.08920000 | -0.44888500 | 4.95626800 |
| H | 1.64698600 | 1.15815100 | 3.59278500 |
| C | 2.45171800 | -1.79313500 | 5.08429600 |
| H | 2.96408500 | -3.61115900 | 4.03958200 |
| H | 1.91762900 | 0.15636000 | 5.84178900 |
| H | 2.56020400 | -2.23462300 | 6.07074700 |
| B | -2.33129000 | -0.45042400 | -0.07536500 |
| O | -3.10748500 | -0.86804500 | 0.98630000 |
| O | -3.04417000 | -0.32150200 | -1.23273700 |
| C | -4.45170000 | -1.17234400 | 0.50571700 |
| C | -4.45363800 | -0.57608700 | -0.96792100 |
| C | -5.44133500 | -0.51358200 | 1.46481600 |
| C | -4.59255200 | -2.69634200 | 0.56870600 |
| C | -4.95244200 | -1.54035800 | -2.04402200 |
| C | -5.16852400 | 0.77161300 | -1.10249600 |
| H | -6.47374500 | -0.67728300 | 1.13828500 |
| H | -5.32646900 | -0.95202000 | 2.46154000 |
| H | -5.26306000 | 0.56066000 | 1.54477100 |
| H | -4.46544200 | -3.02155900 | 1.60643800 |
| H | -5.57732900 | -3.02642000 | 0.22384600 |
| H | -3.82066600 | -3.17963100 | -0.03277700 |
| H | -6.00449500 | -1.79895200 | -1.88374200 |
| H | -4.86475600 | -1.06265500 | -3.02505500 |
| H | -4.36136500 | -2.45849700 | -2.06223100 |
| H | -5.02562300 | 1.14629400 | -2.12108300 |
| H | -6.24328400 | 0.67961100 | -0.91715800 |
| H | -4.74303400 | 1.50360200 | -0.41464100 |

## VII$_{OEt}$

E = -4911.79625

$G_{Corr}$= 0.847746

| | | | |
|---|---|---|---|
| Co | 0.06726800 | -0.55453400 | -0.29585000 |
| P | -1.07860600 | -1.47851700 | 1.31163900 |
| O | -0.78850300 | -1.19424500 | 2.93365900 |
| O | -0.99925800 | -3.14066700 | 1.21169700 |
| C | -2.89366500 | -1.20654100 | 1.44014300 |
| C | 0.54016400 | -1.40928700 | 3.43291800 |
| C | -1.67808500 | -3.99356400 | 2.15767900 |
| C | -3.74207200 | -1.89184000 | 0.55589900 |
| C | -3.43725200 | -0.23267400 | 2.28929800 |
| H | 1.25317500 | -0.79893000 | 2.86629000 |
| H | 0.81136300 | -2.46318200 | 3.29859400 |
| H | -1.79006800 | -3.46972600 | 3.11343700 |
| H | -2.68048600 | -4.21941100 | 1.77524100 |
| C | -5.10722000 | -1.60551100 | 0.51914200 |
| H | -3.32450400 | -2.62826700 | -0.12188600 |
| C | -4.80274600 | 0.05793000 | 2.24772200 |
| H | -2.78743900 | 0.30179300 | 2.97162500 |
| C | -5.64078200 | -0.62373000 | 1.36040100 |
| H | -5.75289100 | -2.14167800 | -0.17186400 |
| H | -5.21248100 | 0.81811900 | 2.90838800 |
| H | -6.70221300 | -0.39279700 | 1.32498500 |
| P | 2.09831700 | -1.20312700 | -0.74565000 |
| O | 2.09431700 | -2.32885100 | -1.98284200 |
| O | 3.11951100 | -1.93026900 | 0.36559700 |
| C | 3.32434600 | 0.05595300 | -1.30569900 |
| C | 3.25145700 | -2.71663700 | -2.74702600 |
| C | 2.62184300 | -3.07302100 | 1.07647100 |
| C | 2.84464900 | 1.10803200 | -2.10092500 |
| C | 4.67670700 | 0.04877300 | -0.92665500 |
| H | 2.85154300 | -3.07741100 | -3.70078800 |
| H | 3.87140900 | -1.83615100 | -2.95563000 |
| H | 1.54739800 | -2.95817500 | 1.25733800 |
| H | 2.75692600 | -3.96777000 | 0.45558300 |
| C | 3.69602300 | 2.13920200 | -2.50178100 |
| H | 1.79107600 | 1.14612900 | -2.35253000 |
| C | 5.52527100 | 1.08478700 | -1.32120200 |
| H | 5.05050500 | -0.75529600 | -0.30261200 |
| C | 5.03664900 | 2.13324200 | -2.10740600 |
| H | 3.30739400 | 2.95515100 | -3.10552200 |
| H | 6.56732000 | 1.07691400 | -1.01181400 |
| H | 5.69715600 | 2.94296800 | -2.40660100 |
| P | -1.26494800 | -0.03690300 | -1.95450400 |
| O | -2.41093800 | -1.23013700 | -2.14041200 |
| O | -0.78438200 | 0.23364800 | -3.53637600 |
| C | -2.29214900 | 1.48608900 | -1.83706700 |
| C | -3.48557900 | -1.11832900 | -3.09249500 |
| C | 0.24430800 | -0.58848600 | -4.11444700 |
| C | -3.36678900 | 1.49968900 | -0.93538500 |
| C | -1.95946000 | 2.66512200 | -2.51726400 |
| H | -4.26330400 | -0.46728900 | -2.67520200 |
| H | -3.11462100 | -0.66076100 | -4.01636600 |
| H | 0.61071700 | -0.02412400 | -4.97881000 |
| H | 1.06416700 | -0.70947100 | -3.40337600 |
| C | -4.10085000 | 2.66662400 | -0.72460800 |

| | | | |
|---|---|---|---|
| H | -3.62596900 | 0.59983800 | -0.39134400 |
| C | -2.68975800 | 3.83647600 | -2.30064700 |
| H | -1.12619800 | 2.66394900 | -3.21149300 |
| C | -3.76175000 | 3.84110200 | -1.40358500 |
| H | -4.93256500 | 2.65599300 | -0.02486100 |
| H | -2.42137700 | 4.74544500 | -2.83347300 |
| H | -4.32884100 | 4.75324900 | -1.23554400 |
| P | 0.27252600 | 1.51791000 | 0.52179600 |
| O | -1.12424700 | 2.02945700 | 1.23995100 |
| O | 0.64155300 | 2.58224800 | -0.68707400 |
| C | 1.52047900 | 1.91701700 | 1.82540200 |
| C | -1.43338700 | 3.35366300 | 1.71065500 |
| C | 0.61060200 | 4.01872300 | -0.77336300 |
| C | 2.87691700 | 2.04033200 | 1.48118100 |
| C | 1.16536100 | 1.93163200 | 3.18197100 |
| H | -0.52400200 | 3.86432600 | 2.05091900 |
| H | -1.86666600 | 3.91619700 | 0.87769000 |
| H | -0.38133500 | 4.38198300 | -0.49479400 |
| H | 0.73117900 | 4.21854200 | -1.84327100 |
| C | 3.84983200 | 2.18410200 | 2.46973300 |
| H | 3.17278500 | 2.03167400 | 0.43919300 |
| C | 2.13838200 | 2.08592400 | 4.17238800 |
| H | 0.12493100 | 1.80545000 | 3.46262700 |
| C | 3.48385000 | 2.20985600 | 3.81934600 |
| H | 4.89421600 | 2.27773300 | 2.18358600 |
| H | 1.84497700 | 2.10068100 | 5.21876500 |
| H | 4.24229300 | 2.32311800 | 4.58938600 |
| C | -2.44918500 | 3.23865400 | 2.83429300 |
| H | -3.34156700 | 2.71718500 | 2.47617100 |
| H | -2.03972500 | 2.67989300 | 3.68258000 |
| H | -2.74027100 | 4.23624700 | 3.18277700 |
| C | 1.70427900 | 4.73338900 | 0.00887800 |
| H | 2.69112300 | 4.36188400 | -0.28262100 |
| H | 1.65603700 | 5.80680300 | -0.21149800 |
| H | 1.59483800 | 4.59859800 | 1.08786900 |
| C | 3.40184400 | -3.20121500 | 2.37365500 |
| H | 3.05127600 | -4.06857900 | 2.94527200 |
| H | 4.47036300 | -3.33044400 | 2.16922400 |
| H | 3.27812900 | -2.30343200 | 2.98811000 |
| C | 0.54724000 | -1.05793100 | 4.90974400 |
| H | -0.14361800 | -1.70463400 | 5.46126000 |
| H | 1.55404700 | -1.18694900 | 5.32264000 |
| H | 0.24406000 | -0.01838100 | 5.06156200 |
| C | -0.85245300 | -5.25859000 | 2.32236200 |
| H | 0.14001900 | -5.01956500 | 2.72020900 |
| H | -1.34748200 | -5.95135700 | 3.01269600 |
| H | -0.72328900 | -5.76114000 | 1.35785000 |
| C | 4.07608200 | -3.80307100 | -2.06840800 |
| H | 4.52248900 | -3.42901700 | -1.14346000 |
| H | 3.44732800 | -4.66762500 | -1.82956400 |
| H | 4.87887000 | -4.13504100 | -2.73790900 |
| C | -4.02520100 | -2.51444400 | -3.35144900 |
| H | -4.37805100 | -2.96772300 | -2.41881000 |
| H | -4.86364900 | -2.47473000 | -4.05641700 |
| H | -3.24378700 | -3.15602800 | -3.77178800 |
| C | -0.26793100 | -1.95931800 | -4.53920700 |
| H | -1.12368800 | -1.86453800 | -5.21682900 |
| H | 0.52770500 | -2.50828100 | -5.05714600 |
| H | -0.57654600 | -2.54221600 | -3.66689000 |

## TS IX-X$^{BS}_{OEt}$

E = -5711.328231

$G_{Corr}$= 1.206303

| | | | |
|---|---|---|---|
| Co | -0.78057800 | -0.06953000 | -0.09690300 |
| H | 1.72712900 | -0.82747300 | 0.62800600 |
| P | -0.01358100 | 1.23663600 | -1.71414500 |
| O | -0.90706900 | 1.31873300 | -3.11752900 |
| O | 1.52936900 | 0.94806200 | -2.25229200 |
| C | 0.08678900 | 3.04422900 | -1.39766300 |
| C | -1.66237700 | 0.16785700 | -3.54831700 |
| C | 2.16802200 | 1.74671300 | -3.27661400 |
| C | 1.23647400 | 3.54897000 | -0.76762000 |
| C | -0.98151000 | 3.90944500 | -1.67069500 |
| H | -1.98716300 | -0.40259900 | -2.67177200 |
| H | -2.55288600 | 0.56712200 | -4.04045300 |
| H | 1.57950800 | 2.64598200 | -3.48304100 |
| H | 3.12855300 | 2.06335400 | -2.85800800 |
| C | 1.32216900 | 4.90110900 | -0.43397400 |
| H | 2.05869200 | 2.88063000 | -0.53448200 |
| C | -0.89664100 | 5.26082500 | -1.32808700 |
| H | -1.87633600 | 3.51898600 | -2.14061900 |
| C | 0.25449800 | 5.76091300 | -0.71227500 |
| H | 2.21895200 | 5.28105800 | 0.04850600 |
| H | -1.72936100 | 5.92512300 | -1.54503300 |
| H | 0.31836300 | 6.81310600 | -0.44767100 |
| P | -0.58653100 | -2.30553500 | -0.53432800 |
| O | 0.04778200 | -3.13762000 | 0.74569700 |
| O | 0.32936500 | -2.82642900 | -1.81822700 |
| C | -2.04652500 | -3.34966600 | -0.93789400 |
| C | 0.57852000 | -4.47430700 | 0.60285400 |
| C | 1.69820500 | -2.35711300 | -1.91513800 |
| C | -2.80475500 | -3.87926600 | 0.11838500 |
| C | -2.43691000 | -3.61286500 | -2.25824700 |
| H | 1.66939900 | -4.37868700 | 0.61631900 |
| H | 0.28230900 | -4.90161800 | -0.36099800 |
| H | 1.70414000 | -1.26556000 | -1.94235300 |
| H | 2.25477600 | -2.67737400 | -1.03024500 |
| C | -3.91837500 | -4.67966100 | -0.14086300 |
| H | -2.51154300 | -3.67368300 | 1.14386200 |
| C | -3.55786200 | -4.40449600 | -2.51660300 |
| H | -1.84679200 | -3.22044900 | -3.07978300 |
| C | -4.29582400 | -4.94485900 | -1.46063100 |
| H | -4.48924300 | -5.09660700 | 0.68482200 |
| H | -3.85140400 | -4.60519200 | -3.54358800 |
| H | -5.16287700 | -5.56750400 | -1.66464100 |
| P | -0.47401700 | 1.04493500 | 1.90679400 |
| O | 1.03825500 | 1.66901000 | 1.97121500 |
| O | -0.64606400 | 0.12674800 | 3.26827300 |
| C | -1.47049800 | 2.47319300 | 2.51421400 |
| C | 1.63965800 | 2.07732300 | 3.21977100 |
| C | -0.61603000 | -1.31049700 | 3.11526600 |
| C | -1.20790800 | 3.75156200 | 2.00276200 |
| C | -2.50392200 | 2.31167600 | 3.44878800 |
| H | 0.90977800 | 2.64134600 | 3.81509700 |
| H | 1.93021100 | 1.17267900 | 3.75802000 |
| H | -1.34206100 | -1.59486900 | 2.34639100 |
| H | 0.37341600 | -1.61122600 | 2.77350700 |
| C | -1.96358700 | 4.84949600 | 2.41729200 |
| H | -0.40955000 | 3.89065700 | 1.28434600 |
| C | -3.25979100 | 3.41059300 | 3.86399400 |
| H | -2.70424000 | 1.32601700 | 3.85289600 |
| C | -2.99282500 | 4.68258500 | 3.34724300 |
| H | -1.74608300 | 5.83270800 | 2.00823400 |
| H | -4.05396200 | 3.27460200 | 4.59404100 |
| H | -3.58253100 | 5.53689200 | 3.66964000 |
| P | -3.02250400 | 0.46744800 | -0.12403900 |
| O | -3.14699800 | 2.08610200 | -0.42929700 |
| O | -3.59336300 | 0.18264200 | 1.39899600 |
| C | -4.39366200 | -0.18412900 | -1.17719100 |
| C | -4.01309600 | 3.05594500 | 0.19222900 |
| C | -4.93211000 | 0.31812300 | 1.91750800 |
| C | -5.00181300 | -1.41128500 | -0.87734800 |
| C | -4.77327800 | 0.49090400 | -2.34796800 |
| H | -3.50255500 | 4.00910800 | 0.03396800 |
| H | -4.03144800 | 2.88047100 | 1.26930700 |
| H | -5.00849600 | 1.29628200 | 2.40421000 |
| H | -5.66582100 | 0.27132900 | 1.10664400 |
| C | -5.98189200 | -1.94241900 | -1.71679200 |
| H | -4.70737100 | -1.95840500 | 0.01100400 |
| C | -5.74690000 | -0.04307300 | -3.19257400 |

| | | | |
|---|---|---|---|
| H | -4.30799900 | 1.44058800 | -2.58903100 |
| C | -6.35643500 | -1.26080600 | -2.87683400 |
| H | -6.44128900 | -2.89401200 | -1.46761500 |
| H | -6.03292600 | 0.49324000 | -4.09367600 |
| H | -7.11614000 | -1.67689600 | -3.53313000 |
| C | 7.98106300 | 1.34748100 | -2.26343800 |
| H | 8.25749000 | 2.32811200 | -2.66990400 |
| H | 8.34042800 | 1.29346300 | -1.22951400 |
| H | 8.50410800 | 0.58016700 | -2.84788700 |
| C | 6.45350900 | 1.12769900 | -2.33303100 |
| C | 6.11386600 | -0.28673200 | -1.80223600 |
| C | 5.77442800 | 2.17626500 | -1.48108000 |
| C | 4.64899900 | -0.44798200 | -1.35849900 |
| H | 6.75449800 | -0.50118800 | -0.93700200 |
| H | 6.35702400 | -1.03440200 | -2.56744300 |
| C | 4.83691600 | 1.90674200 | -0.52574300 |
| H | 6.10468300 | 3.20412000 | -1.62604300 |
| C | 4.30816500 | 0.59168900 | -0.32278200 |
| H | 4.46304400 | -1.44233800 | -0.95558100 |
| H | 3.96949500 | -0.30440500 | -2.20995200 |
| H | 4.46787000 | 2.69474600 | 0.12603400 |
| C | 6.00506100 | 1.27997300 | -3.80507600 |
| H | 6.15942700 | 2.30604400 | -4.15936000 |
| H | 6.58326400 | 0.60663700 | -4.45052000 |
| H | 4.94744200 | 1.03847300 | -3.92628400 |
| O | 3.59569300 | 0.37159500 | 0.72036000 |
| C | 4.36157300 | -2.46656400 | 1.99505500 |
| C | 3.52582600 | -1.91370600 | 3.21299300 |
| O | 3.51880900 | -2.15472600 | 0.87957300 |
| O | 2.77053900 | -0.84185300 | 2.64279200 |
| C | 5.71714800 | -1.75200600 | 1.83033900 |
| H | 6.41987100 | -2.03250200 | 2.62279100 |
| H | 6.15513600 | -2.04007800 | 0.86855000 |
| H | 5.59936700 | -0.66634900 | 1.83938900 |
| C | 4.60149300 | -3.97789400 | 2.00580300 |
| H | 5.17276200 | -4.26431700 | 1.11540600 |
| H | 5.17439800 | -4.28177400 | 2.88983500 |
| H | 3.66003300 | -4.53194700 | 1.99289500 |
| C | 2.53802800 | -2.95542000 | 3.75929600 |
| H | 3.04634800 | -3.79157800 | 4.25274100 |
| H | 1.88173400 | -2.47374900 | 4.49022700 |
| H | 1.91667600 | -3.34325100 | 2.94926300 |
| C | 4.36848600 | -1.36226100 | 4.36711700 |
| H | 3.70656300 | -1.00342400 | 5.16340000 |
| H | 5.02145300 | -2.13539000 | 4.79029300 |
| H | 4.98501900 | -0.52097800 | 4.04117600 |
| B | 2.79352100 | -0.94004700 | 1.20161700 |
| C | -5.15270600 | -0.80186100 | 2.92020000 |
| H | -6.13861800 | -0.70645600 | 3.38909000 |
| H | -5.09114600 | -1.77843600 | 2.42843000 |
| H | -4.38747800 | -0.76410700 | 3.70299200 |
| C | -5.41979800 | 3.10176300 | -0.38866200 |
| H | -5.98875300 | 3.89313300 | 0.11425100 |
| H | -5.39673100 | 3.32294600 | -1.46033200 |
| H | -5.95161800 | 2.15647200 | -0.25013200 |
| C | -0.88331700 | -0.73292400 | -4.49186900 |
| H | -1.52727500 | -1.55780500 | -4.82019100 |
| H | -0.55286000 | -0.18565100 | -5.38021500 |
| H | -0.01284800 | -1.15948100 | -3.98786000 |
| C | 2.35491900 | 0.92832900 | -4.54381100 |
| H | 2.86098300 | -0.01883400 | -4.32803700 |
| H | 1.39065800 | 0.70256400 | -5.00131500 |
| H | 2.96059900 | 1.48781800 | -5.26627300 |
| C | 2.85236800 | 2.92576100 | 2.88495500 |
| H | 3.55164200 | 2.33309100 | 2.29052600 |
| H | 2.55729500 | 3.81051600 | 2.30916700 |
| H | 3.35070000 | 3.25560400 | 3.80422800 |
| C | -0.96902100 | -1.92499100 | 4.45516100 |
| H | -0.93891600 | -3.01835800 | 4.38194800 |
| H | -0.25892500 | -1.61132700 | 5.22802200 |
| H | -1.97551200 | -1.62434400 | 4.76914500 |
| C | 0.08335700 | -5.33721000 | 1.75238600 |
| H | 0.31436100 | -4.87042700 | 2.71504600 |
| H | -0.99919400 | -5.48368300 | 1.68928400 |
| H | 0.56930200 | -6.31960200 | 1.72067100 |
| C | 2.29906900 | -2.94704900 | -3.17830000 |
| H | 3.34566900 | -2.63469400 | -3.27015400 |
| H | 2.26701500 | -4.04179000 | -3.15025200 |
| H | 1.75987400 | -2.60992700 | -4.07019900 |

## $\mathbf{II_{OEt}}$

E = -4417.627179

$G_{Corr}$= 0.80798

| | | | |
|---|---|---|---|
| Co | -0.26057000 | 0.33158500 | -0.08203600 |
| H | -1.36573000 | 0.84353200 | 0.79428200 |
| P | 1.30153500 | -0.36596500 | -1.44908200 |
| O | 1.85349500 | 0.73895400 | -2.55310200 |
| O | 0.81150600 | -1.64502900 | -2.37347800 |
| C | 2.95441300 | -0.92095600 | -0.87617800 |
| C | 0.96338800 | 1.76364700 | -3.04707000 |
| C | 1.58507200 | -2.10002500 | -3.50268700 |
| C | 3.12593300 | -2.24511700 | -0.44510500 |
| C | 4.03056900 | -0.02672900 | -0.78817900 |
| H | 1.60332700 | 2.62383700 | -3.26506000 |
| H | 0.26277900 | 2.04664500 | -2.25675200 |
| H | 1.42664800 | -1.40967900 | -4.33853300 |
| H | 2.65324300 | -2.09284800 | -3.25433500 |
| C | 4.35207800 | -2.66488300 | 0.07281000 |
| H | 2.30609900 | -2.94924700 | -0.52275900 |
| C | 5.25838600 | -0.45028300 | -0.27611100 |
| H | 3.90647700 | 0.99362400 | -1.13186800 |
| C | 5.42078100 | -1.76877100 | 0.15962600 |
| H | 4.46757000 | -3.69280900 | 0.40574500 |
| H | 6.08833500 | 0.24927700 | -0.21736800 |
| H | 6.37570700 | -2.09561800 | 0.56289900 |
| P | -0.95534800 | -1.40317300 | 0.88783800 |
| O | -2.49802200 | -1.86647600 | 0.42566100 |
| O | -1.09942000 | -1.37456900 | 2.54699400 |
| C | -0.08435800 | -3.02899400 | 0.85592200 |
| C | -3.21845200 | -2.87538300 | 1.16081700 |
| C | -1.65882800 | -0.23681900 | 3.22328800 |
| C | -0.23021900 | -3.84964500 | -0.27361900 |
| C | 0.80189400 | -3.41922900 | 1.87108200 |
| H | -3.76856800 | -2.38849500 | 1.97608300 |
| H | -2.51978400 | -3.59099600 | 1.60875700 |
| H | -2.72079800 | -0.14144900 | 2.95622200 |
| H | -1.14882400 | 0.67588900 | 2.90495700 |
| C | 0.48880400 | -5.04097700 | -0.38046000 |
| H | -0.89900100 | -3.54630000 | -1.07283500 |
| C | 1.53221000 | -4.60460500 | 1.75682500 |
| H | 0.91075000 | -2.79669200 | 2.75278400 |
| C | 1.37803700 | -5.41833600 | 0.63164200 |
| H | 0.35872400 | -5.67399300 | -1.25430000 |
| H | 2.21936000 | -4.89348900 | 2.54796700 |
| H | 1.94471000 | -6.34143800 | 0.54313000 |
| P | 0.84540300 | 1.82669000 | 0.90410300 |
| O | 2.21292000 | 1.35185600 | 1.73295800 |
| O | -0.01975400 | 2.61118900 | 2.09529000 |
| C | 1.63461300 | 3.26503200 | 0.05874000 |
| C | 2.16128200 | 0.09836300 | 2.43865900 |
| C | 0.61514200 | 3.56966500 | 2.96262400 |
| C | 0.77927000 | 4.18830000 | -0.56424600 |
| C | 3.02018300 | 3.42862200 | -0.07525500 |
| H | 1.94576900 | -0.70669100 | 1.73017800 |
| H | 1.34684300 | 0.12227600 | 3.17419000 |
| H | 1.63642900 | 3.24495600 | 3.19039800 |
| H | 0.67219200 | 4.53661600 | 2.44646700 |
| C | 1.29472100 | 5.25020300 | -1.30643400 |
| H | -0.29841400 | 4.06683000 | -0.47322300 |
| C | 3.53811900 | 4.48422300 | -0.83124000 |
| H | 3.68771600 | 2.73136800 | 0.41871100 |

| | | | |
|---|---|---|---|
| C | 2.67892400 | 5.39560100 | -1.44942100 |
| H | 0.62019800 | 5.95978500 | -1.77880900 |
| H | 4.61469800 | 4.59709700 | -0.93236400 |
| H | 3.08331500 | 6.21529900 | -2.03736500 |
| C | -6.91312100 | 1.92982600 | -0.70542400 |
| H | -7.13766300 | 2.39227800 | 0.26339300 |
| H | -6.88322800 | 2.72310100 | -1.46173800 |
| H | -7.73871400 | 1.25320900 | -0.95573400 |
| C | -5.58215800 | 1.15830000 | -0.65369500 |
| C | -5.29658200 | 0.52377700 | -2.03567400 |
| C | -4.44775200 | 2.09981400 | -0.30853400 |
| C | -3.89844900 | -0.09750700 | -2.13237100 |
| H | -5.39456900 | 1.30878800 | -2.79720600 |
| H | -6.05991100 | -0.23228400 | -2.25803600 |
| C | -3.17800600 | 1.90881800 | -0.71529900 |
| H | -4.67918800 | 2.93578000 | 0.35146100 |
| C | -2.80449000 | 0.76889400 | -1.54825000 |
| H | -3.63350200 | -0.34467300 | -3.16687100 |
| H | -3.83680600 | -1.03661700 | -1.56700500 |
| H | -2.35976800 | 2.54403900 | -0.38938200 |
| C | -5.65739900 | 0.07515800 | 0.45208600 |
| H | -5.87086200 | 0.53131900 | 1.42598200 |
| H | -6.45980400 | -0.63990300 | 0.22996600 |
| H | -4.71252400 | -0.46941100 | 0.53729700 |
| O | -1.61735500 | 0.45401000 | -1.73489200 |
| C | -0.21829800 | 3.66928800 | 4.22947700 |
| H | -0.25481100 | 2.70044600 | 4.73949400 |
| H | 0.21281800 | 4.40880600 | 4.91408500 |
| H | -1.24424400 | 3.97146300 | 3.99264600 |
| C | 3.50298200 | -0.11152900 | 3.11575500 |
| H | 3.50268800 | -1.06386200 | 3.65879600 |
| H | 4.30134500 | -0.13989100 | 2.36890700 |
| H | 3.71189000 | 0.69485800 | 3.82753500 |
| C | -1.49267000 | -0.46609300 | 4.71607600 |
| H | -1.91652600 | 0.37542100 | 5.27615600 |
| H | -1.99728400 | -1.38570900 | 5.03164500 |
| H | -0.43040300 | -0.55191500 | 4.97199200 |
| C | -4.16638300 | -3.58011000 | 0.20470800 |
| H | -4.86561700 | -2.86780100 | -0.24586100 |
| H | -3.60255700 | -4.06593800 | -0.59876200 |
| H | -4.74666800 | -4.34374700 | 0.73571800 |
| C | 0.21389500 | 1.29307000 | -4.28423200 |
| H | -0.40408500 | 2.10847800 | -4.67986500 |
| H | 0.91226500 | 0.97772100 | -5.06736700 |
| H | -0.44247600 | 0.45594100 | -4.02952400 |
| C | 1.12067800 | -3.50381600 | -3.84799500 |
| H | 1.65558700 | -3.87483500 | -4.72971000 |
| H | 1.30745100 | -4.18266200 | -3.01060500 |
| H | 0.04671400 | -3.50999800 | -4.06390200 |

# TS III-IV$_{OEt}$

E = -4417.620013

$G_{Corr}$= 0.817469

| | | | |
|---|---|---|---|
| Co | 0.11620800 | 0.53776200 | -0.27186100 |
| H | 0.52096300 | 1.91838500 | 0.20903500 |
| P | -0.22022800 | -1.44461500 | -1.09934600 |
| O | 1.19387500 | -2.16647600 | -1.54810100 |
| O | -1.04674800 | -1.68555900 | -2.50650600 |
| C | -0.99511500 | -2.72294800 | -0.01818500 |
| C | 1.27151100 | -3.47501800 | -2.15052600 |
| C | -2.39630400 | -1.16749800 | -2.60437700 |
| C | -0.81076300 | -2.64047800 | 1.36876500 |
| C | -1.75388700 | -3.78076300 | -0.54309600 |
| H | 0.81066300 | -4.21371200 | -1.48265100 |
| H | 0.71387200 | -3.46936700 | -3.09270500 |
| H | -2.40499200 | -0.13753800 | -2.25702600 |
| H | -3.05687500 | -1.76276700 | -1.96206300 |
| C | -1.36909600 | -3.59687500 | 2.21758300 |
| H | -0.24865400 | -1.81314700 | 1.78138800 |
| C | -2.32336800 | -4.73135300 | 0.30632000 |
| H | -1.90159400 | -3.85279300 | -1.61622300 |
| C | -2.13122500 | -4.64178800 | 1.68818800 |
| H | -1.22249000 | -3.51398300 | 3.29136800 |
| H | -2.91728600 | -5.54097800 | -0.10946900 |
| H | -2.57701000 | -5.38109700 | 2.34833700 |
| P | -1.62023900 | 0.85739400 | 0.97580600 |
| O | -2.01874900 | 2.45615600 | 1.22904100 |
| O | -1.57016000 | 0.29829600 | 2.54926600 |
| C | -3.27102900 | 0.15182700 | 0.56464700 |
| C | -3.12552700 | 2.84377500 | 2.06784600 |
| C | -0.54096700 | 0.83905700 | 3.40143500 |
| C | -4.00813400 | 0.77225000 | -0.45784400 |
| C | -3.77213900 | -1.01875400 | 1.15002200 |
| H | -4.05050100 | 2.79197100 | 1.48156300 |
| H | -3.21623400 | 2.14329400 | 2.90543800 |
| H | 0.27969600 | 1.23821000 | 2.79686800 |
| H | -0.97900600 | 1.66882900 | 3.97232800 |
| C | -5.21612300 | 0.22765600 | -0.89235900 |
| H | -3.62605200 | 1.66913200 | -0.93675500 |
| C | -4.97646000 | -1.57027700 | 0.70569500 |
| H | -3.21965700 | -1.50003900 | 1.94741300 |
| C | -5.69967600 | -0.95164900 | -0.31672600 |
| H | -5.77355700 | 0.71771900 | -1.68616000 |
| H | -5.34621300 | -2.48603000 | 1.15980300 |
| H | -6.63491600 | -1.38368400 | -0.66302500 |
| P | 1.97983200 | 0.02023100 | 0.70694200 |
| O | 2.14303300 | -1.24225100 | 1.78567500 |
| O | 2.37549200 | 1.24433100 | 1.74434000 |
| C | 3.51213300 | -0.19921300 | -0.31967200 |
| C | 2.49540200 | -2.59422200 | 1.43615700 |
| C | 3.37257500 | 1.21358800 | 2.78391900 |
| C | 3.38790700 | -0.11132500 | -1.71265400 |
| C | 4.78307200 | -0.45064500 | 0.22606800 |
| H | 1.77226900 | -2.99577700 | 0.72283500 |
| H | 3.48064400 | -2.60582500 | 0.95693800 |
| H | 2.84870300 | 1.47613900 | 3.71045400 |
| H | 3.76535100 | 0.20152000 | 2.91205300 |
| C | 4.50400100 | -0.23924700 | -2.54249600 |
| H | 2.40209400 | 0.02380300 | -2.14261300 |
| C | 5.89982500 | -0.58437600 | -0.59983400 |
| H | 4.90610400 | -0.56157600 | 1.29832000 |
| C | 5.76347300 | -0.47200500 | -1.98710200 |
| H | 4.38636700 | -0.16655800 | -3.62039400 |
| H | 6.87489200 | -0.78108700 | -0.16202500 |
| H | 6.63374300 | -0.57513000 | -2.62963200 |
| C | 2.19104500 | 4.47674300 | -1.60669600 |
| H | 3.06167000 | 4.10504200 | -1.05221200 |
| H | 2.32536500 | 4.22161600 | -2.66489000 |
| H | 2.17569200 | 5.57013800 | -1.52141600 |
| C | 0.88193400 | 3.87681300 | -1.04729100 |
| C | -0.31594000 | 4.51664100 | -1.78989900 |
| C | 0.94006500 | 2.35436600 | -1.35559000 |
| C | -1.57308900 | 3.63852500 | -1.75059000 |
| H | -0.03949600 | 4.68276300 | -2.84051400 |
| H | -0.52336500 | 5.50508200 | -1.36087600 |
| C | -0.00792200 | 1.69481400 | -2.17870300 |
| H | 1.96528100 | 2.01008100 | -1.44349200 |
| C | -1.30263600 | 2.31801900 | -2.45899000 |
| H | -2.41863400 | 4.12046000 | -2.25197000 |
| H | -1.85736500 | 3.43955400 | -0.71287100 |
| H | 0.30156100 | 0.90947500 | -2.86350000 |
| C | 0.81116100 | 4.19227600 | 0.45927300 |
| H | 1.61829900 | 3.68503300 | 0.99872400 |
| H | 0.91046300 | 5.27366400 | 0.61842300 |
| H | -0.13711700 | 3.86053700 | 0.88903600 |
| O | -2.11760500 | 1.85293800 | -3.26384500 |
| C | -0.03620000 | -0.25265700 | 4.33027900 |
| H | 0.51339100 | -1.01135900 | 3.76669000 |
| H | 0.64295000 | 0.17556100 | 5.07738400 |
| H | -0.87172400 | -0.72866800 | 4.85540400 |

| | | | |
|---|---|---|---|
| C | -2.86022200 | 4.25739600 | 2.55653000 |
| H | -2.74729800 | 4.94125200 | 1.70846700 |
| H | -3.69142100 | 4.60843900 | 3.17859600 |
| H | -1.94002900 | 4.29161000 | 3.14958200 |
| C | 4.46750200 | 2.22400900 | 2.47897400 |
| H | 5.19392500 | 2.25694300 | 3.29968100 |
| H | 4.99338400 | 1.96132700 | 1.55626900 |
| H | 4.03617000 | 3.22323500 | 2.35717600 |
| C | 2.49957700 | -3.40594400 | 2.71865000 |
| H | 2.79691400 | -4.43914200 | 2.50674500 |
| H | 3.20267400 | -2.98067100 | 3.44270700 |
| H | 1.50114300 | -3.41870700 | 3.16692000 |
| C | 2.74315200 | -3.78014700 | -2.37111200 |
| H | 2.85942300 | -4.76351900 | -2.84025700 |
| H | 3.19994900 | -3.02339300 | -3.01617300 |
| H | 3.28157200 | -3.78038600 | -1.41767300 |
| C | -2.81897000 | -1.23524100 | -4.05652100 |
| H | -3.85579500 | -0.89403700 | -4.15127800 |
| H | -2.18596000 | -0.57673200 | -4.65745400 |
| H | -2.75036800 | -2.25932200 | -4.44092700 |

# 8. NMR Spectra

## 1,3-diphenylpropan-1-one (2a)

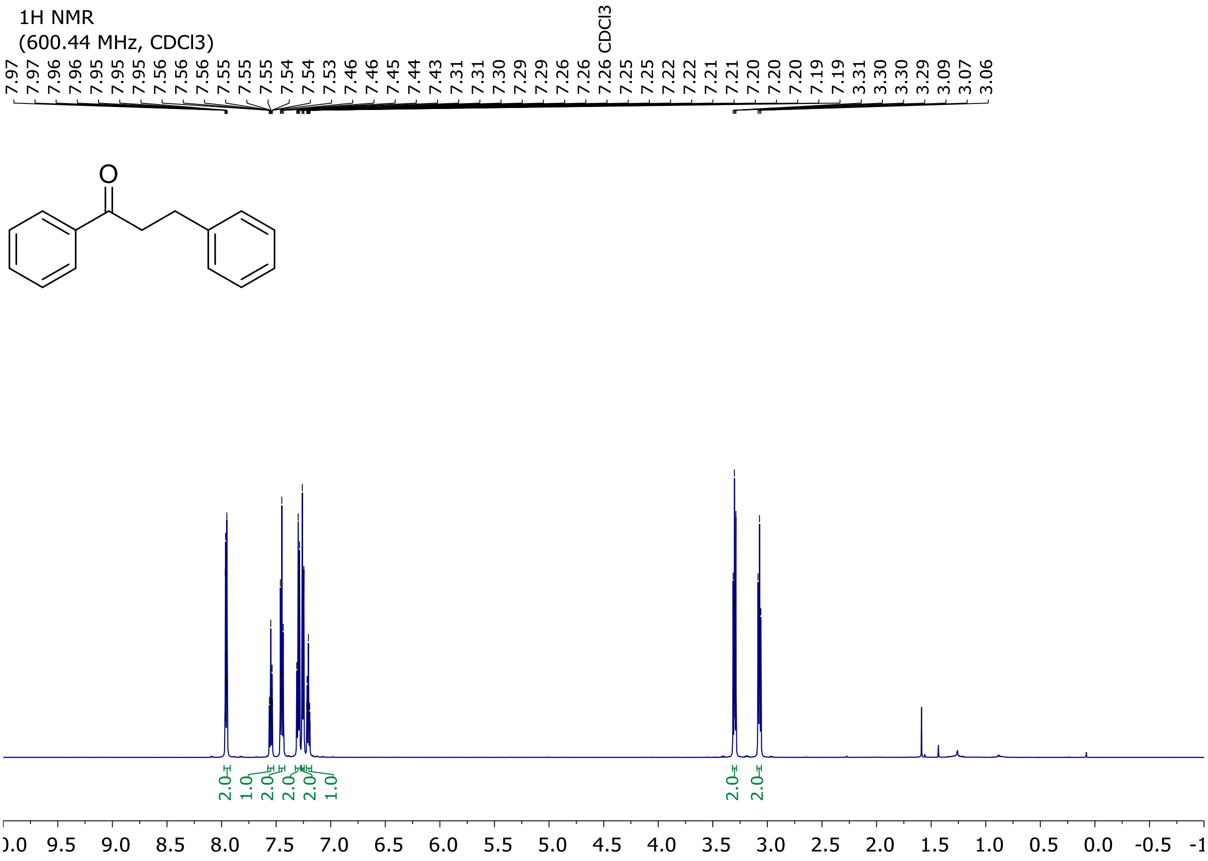


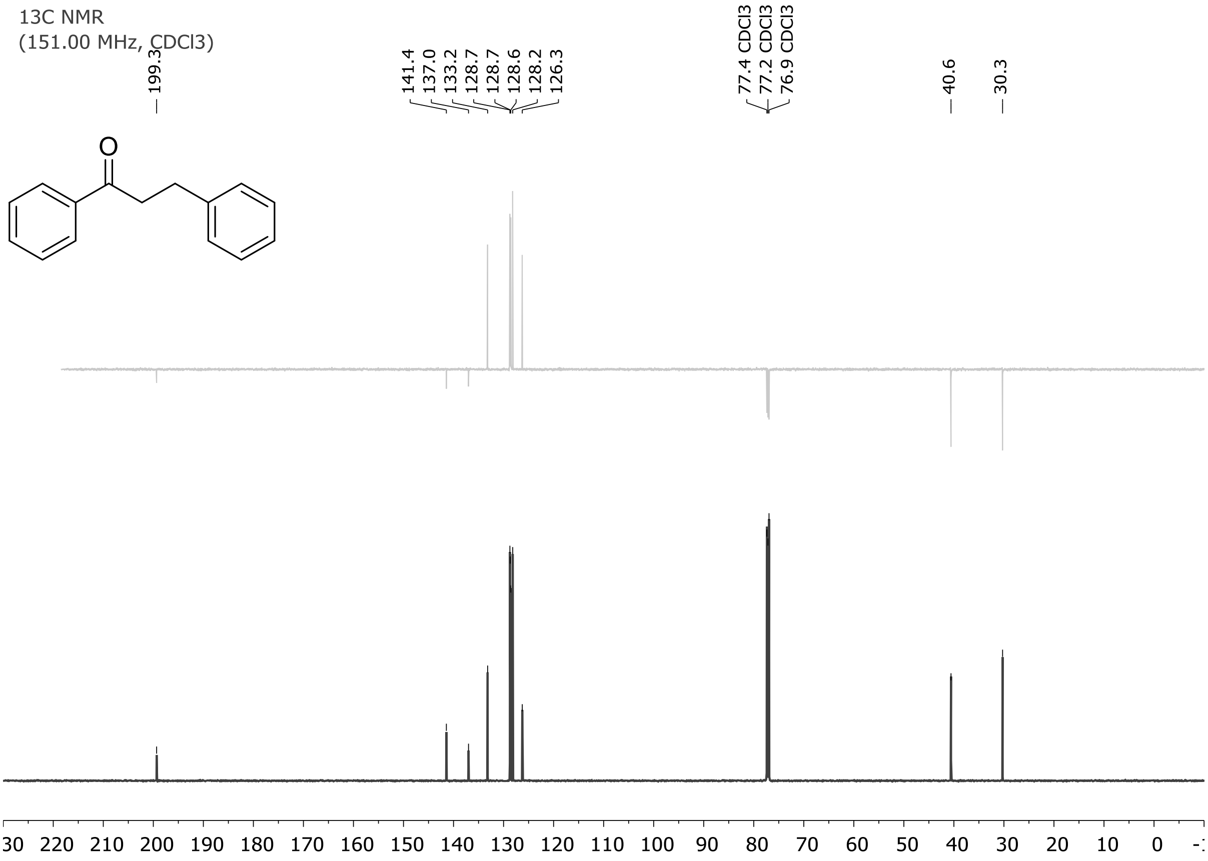

# 3-(4-methoxyphenyl)-1-phenylpropan-1-one (2b)

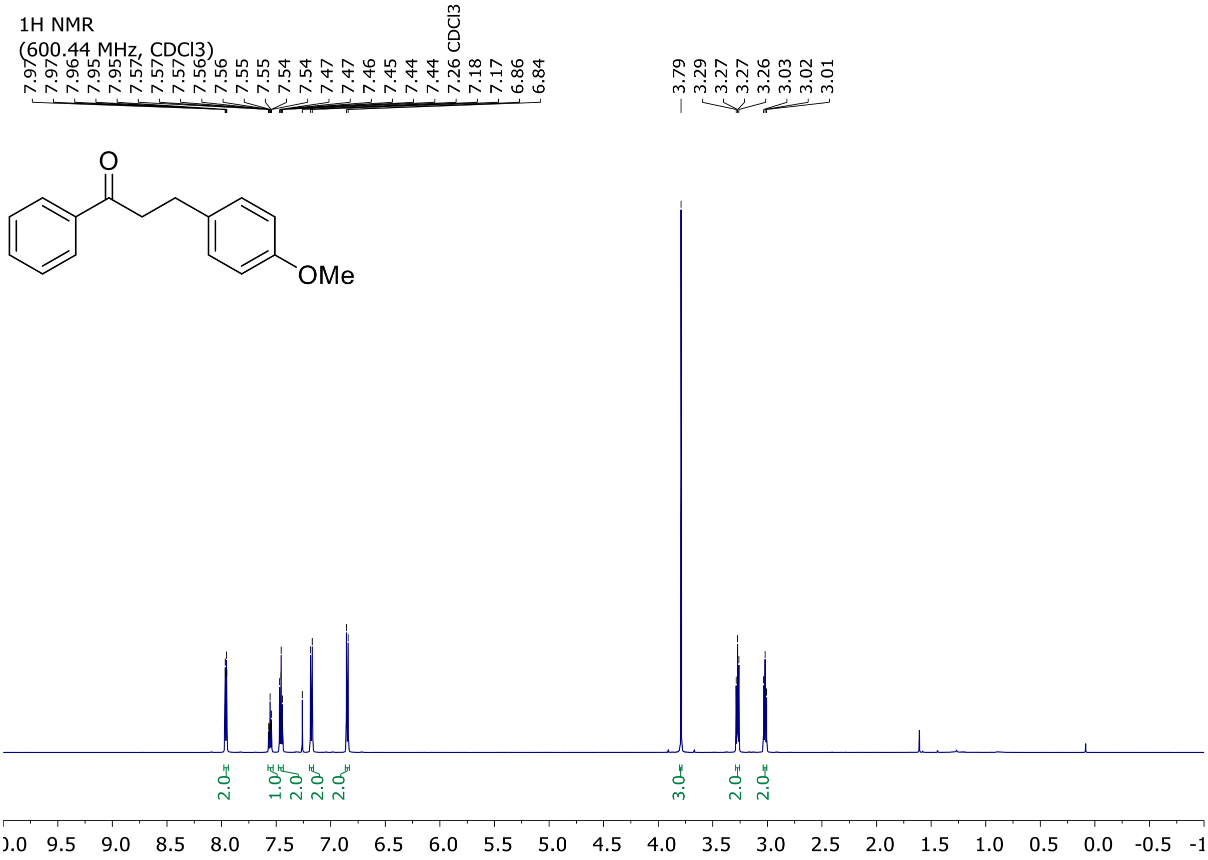


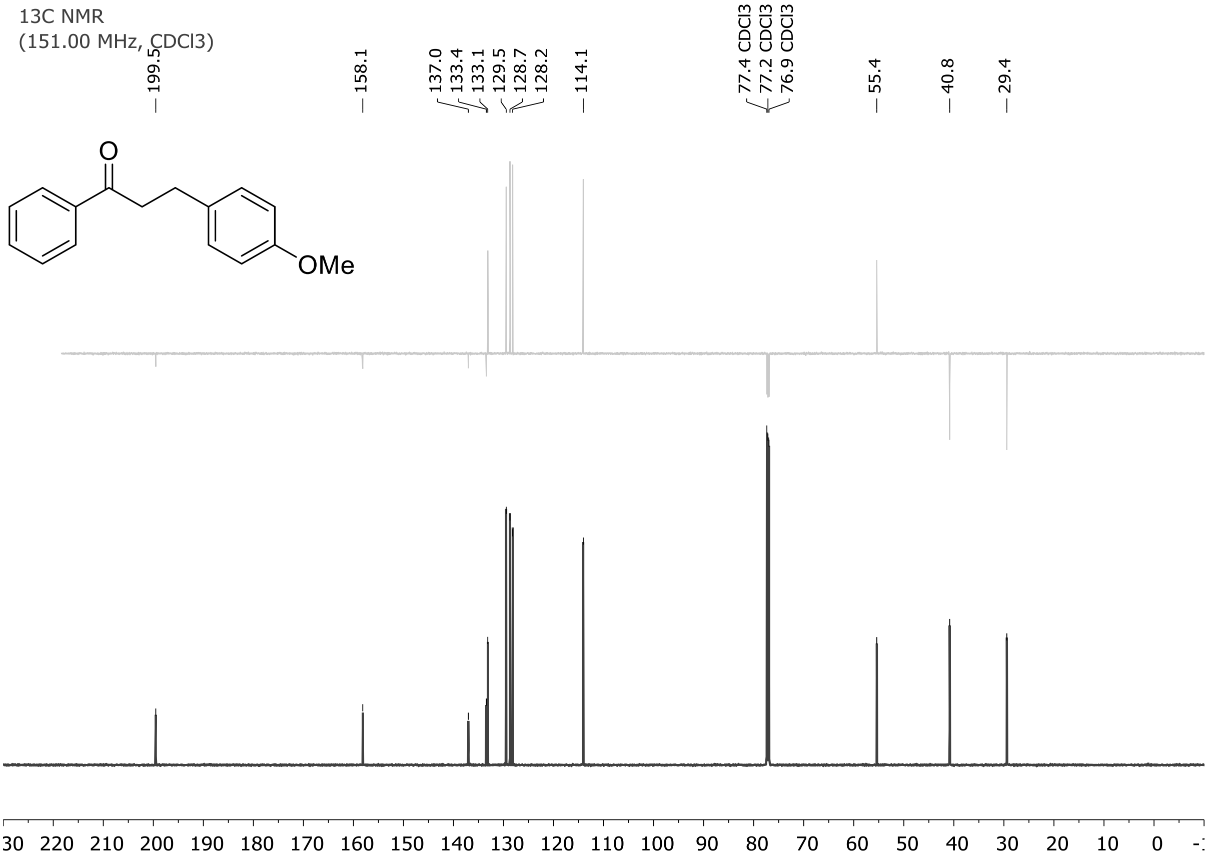

## methyl 4-(3-oxo-3-phenylpropyl)benzoate (2c)

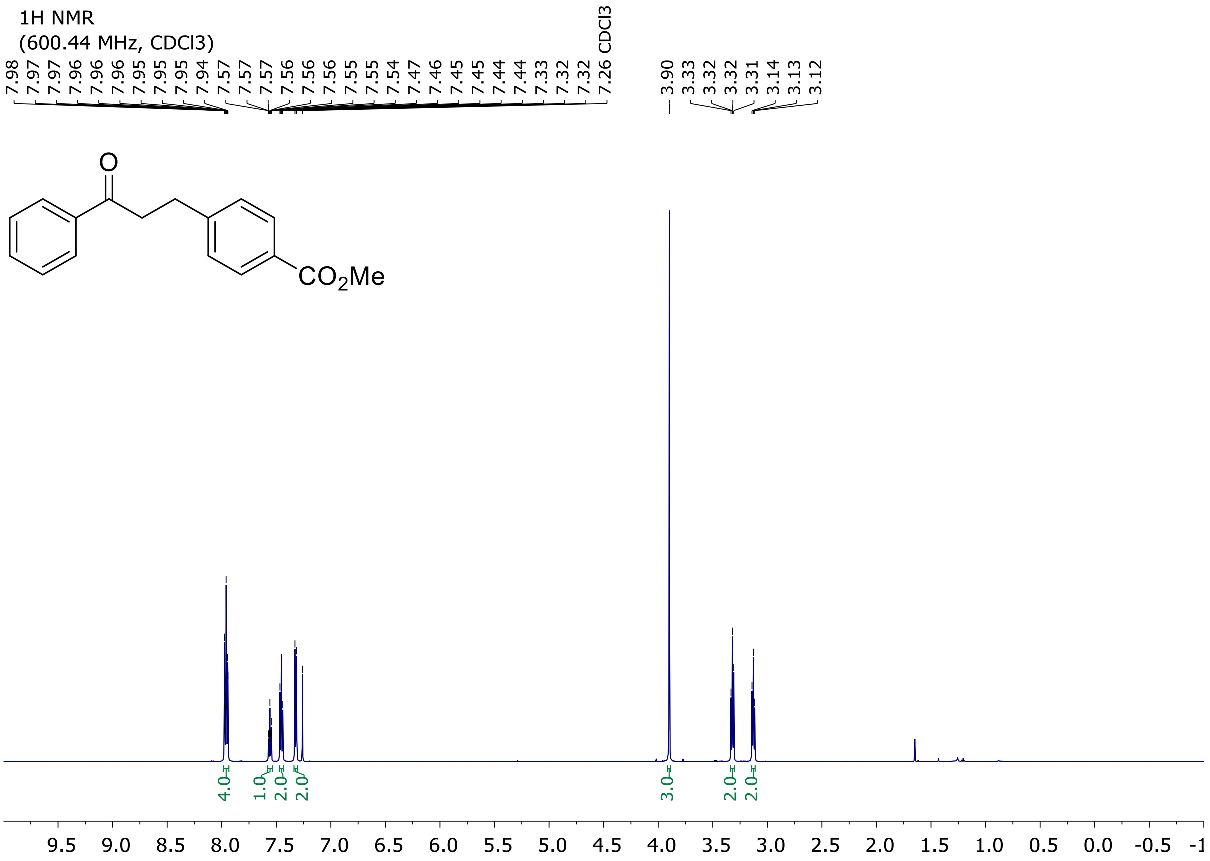


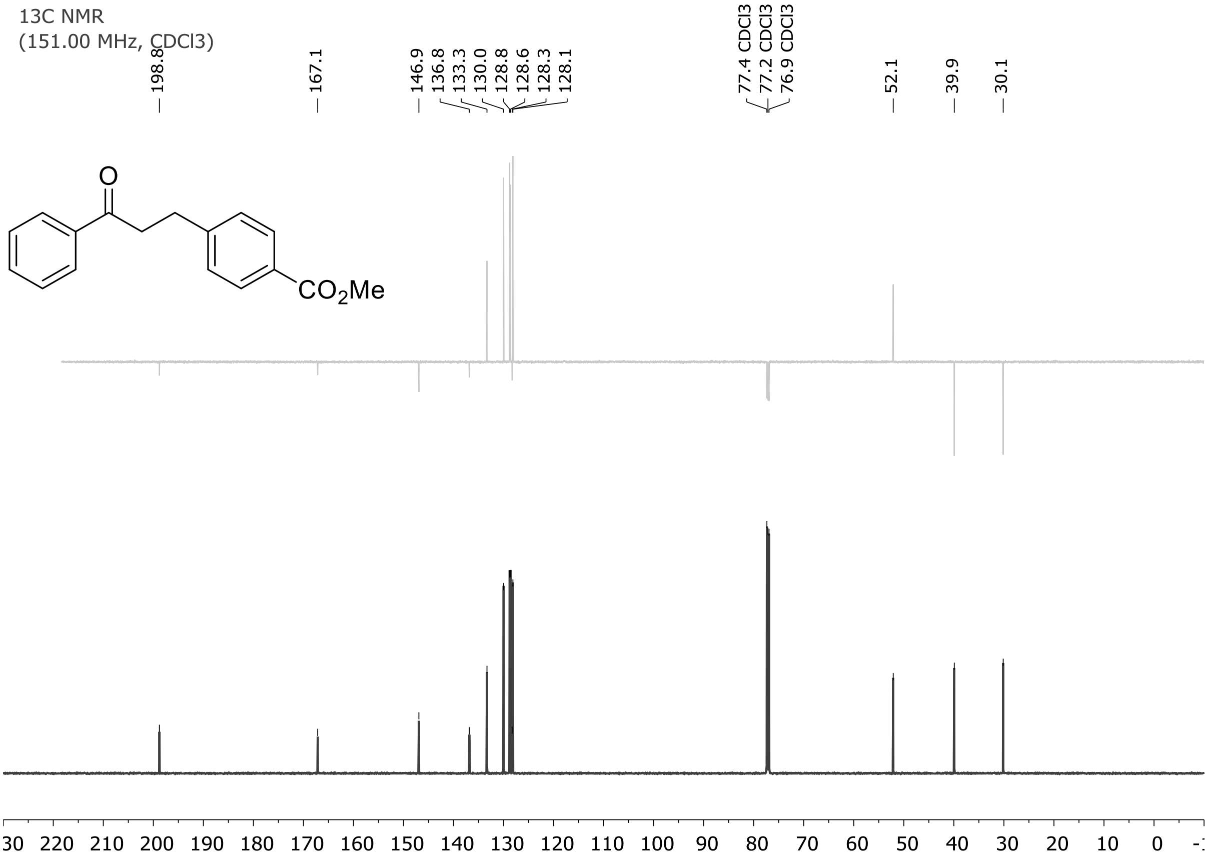

## 3-(4-chlorophenyl)-1-phenylpropan-1-one (2d)

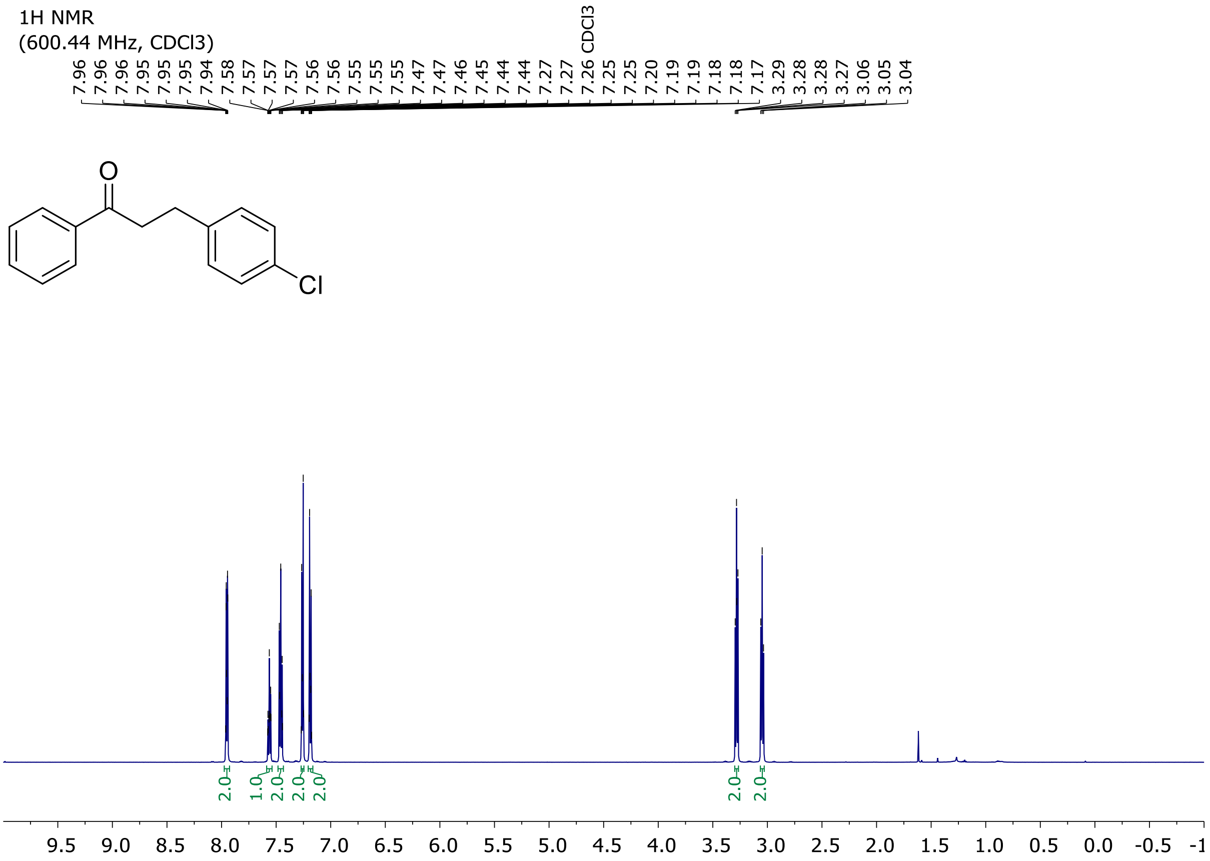


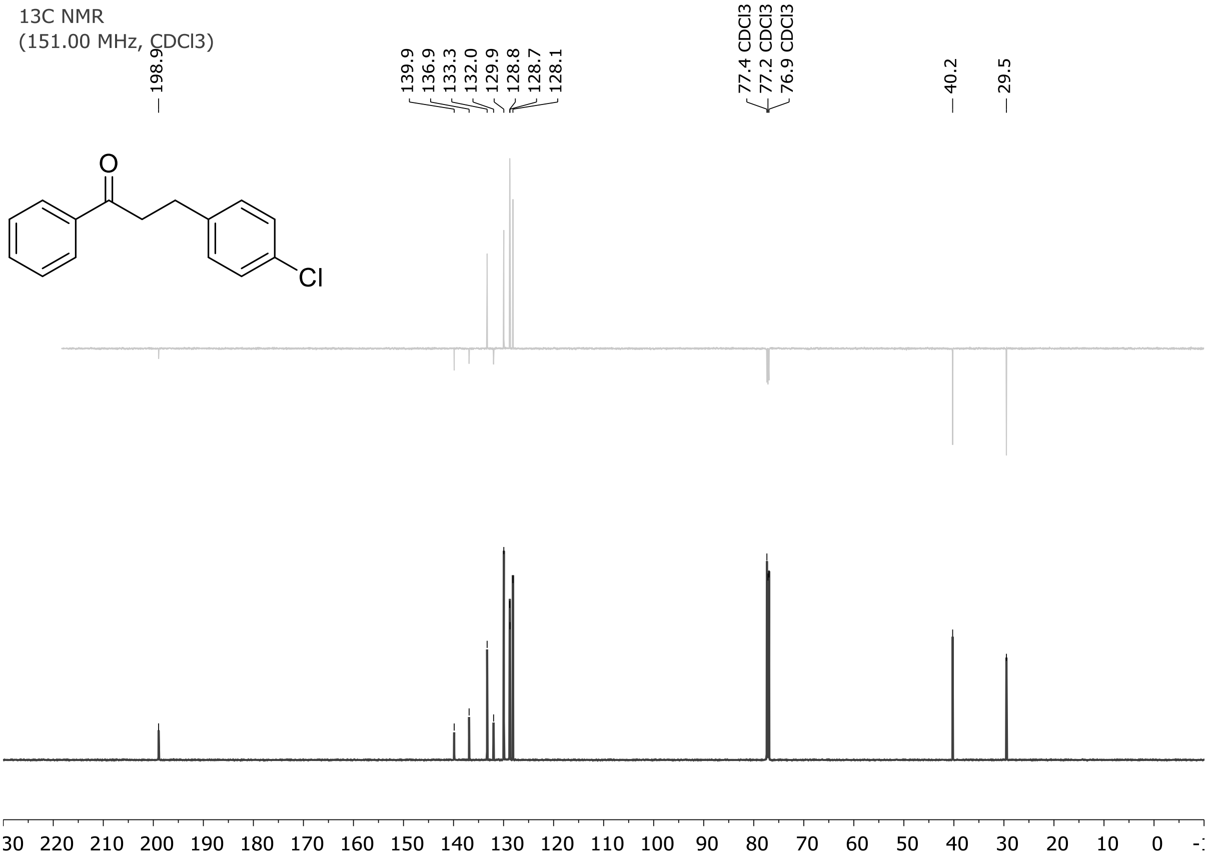

# 1-(4-fluorophenyl)-3-phenylpropan-1-one (2e)

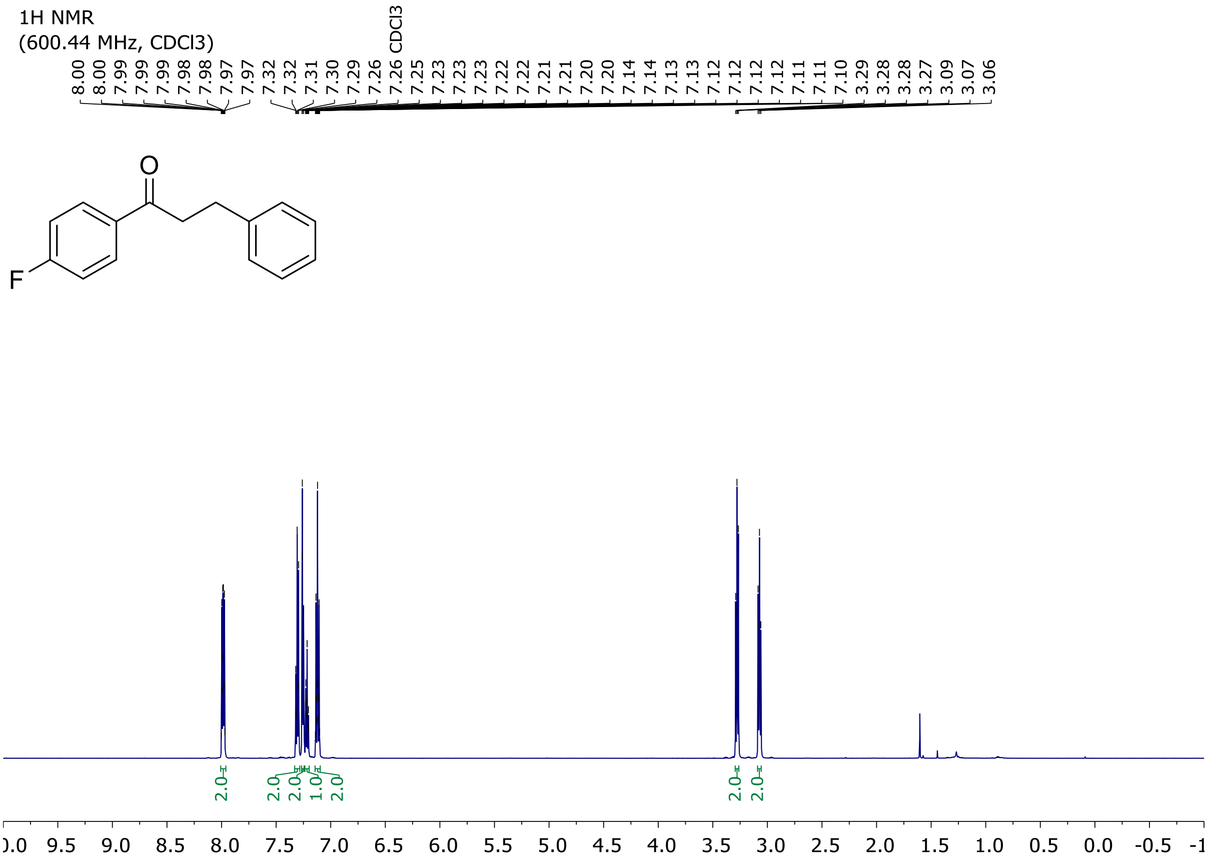


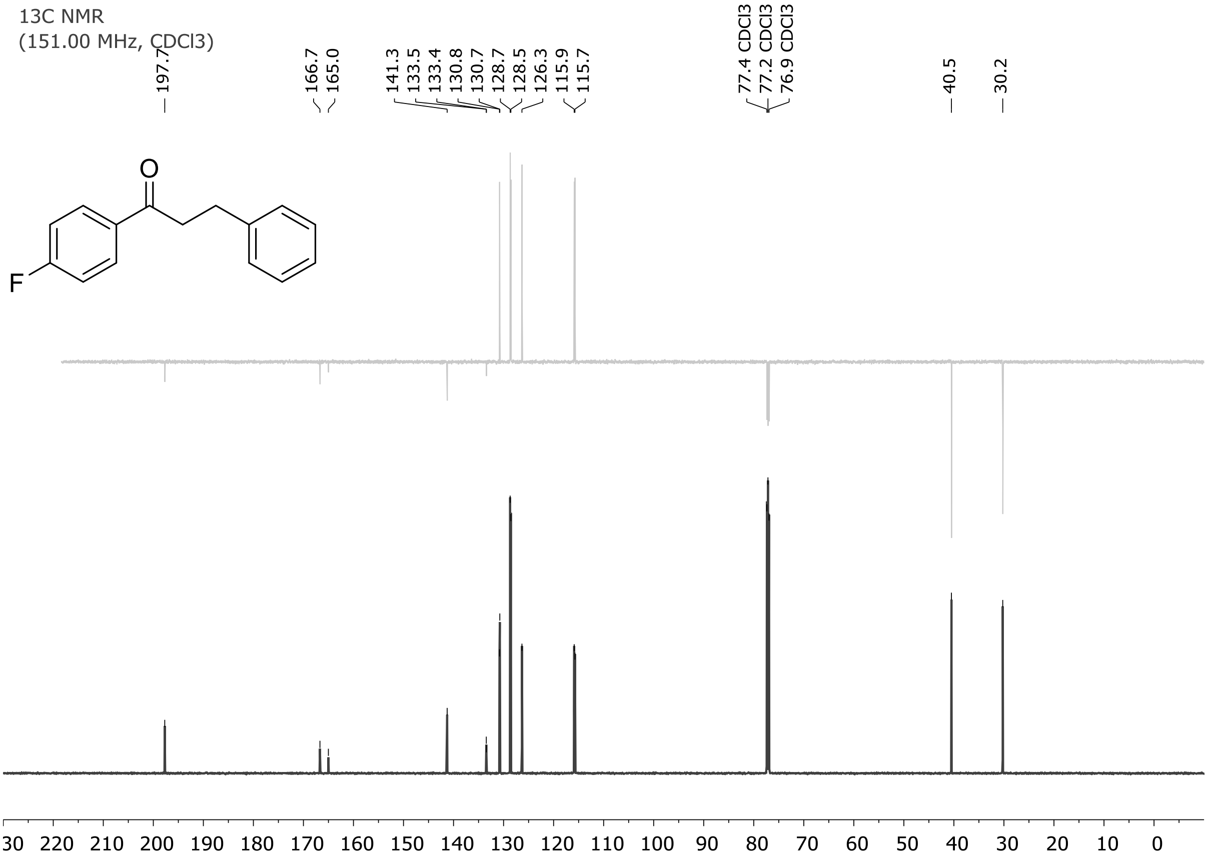

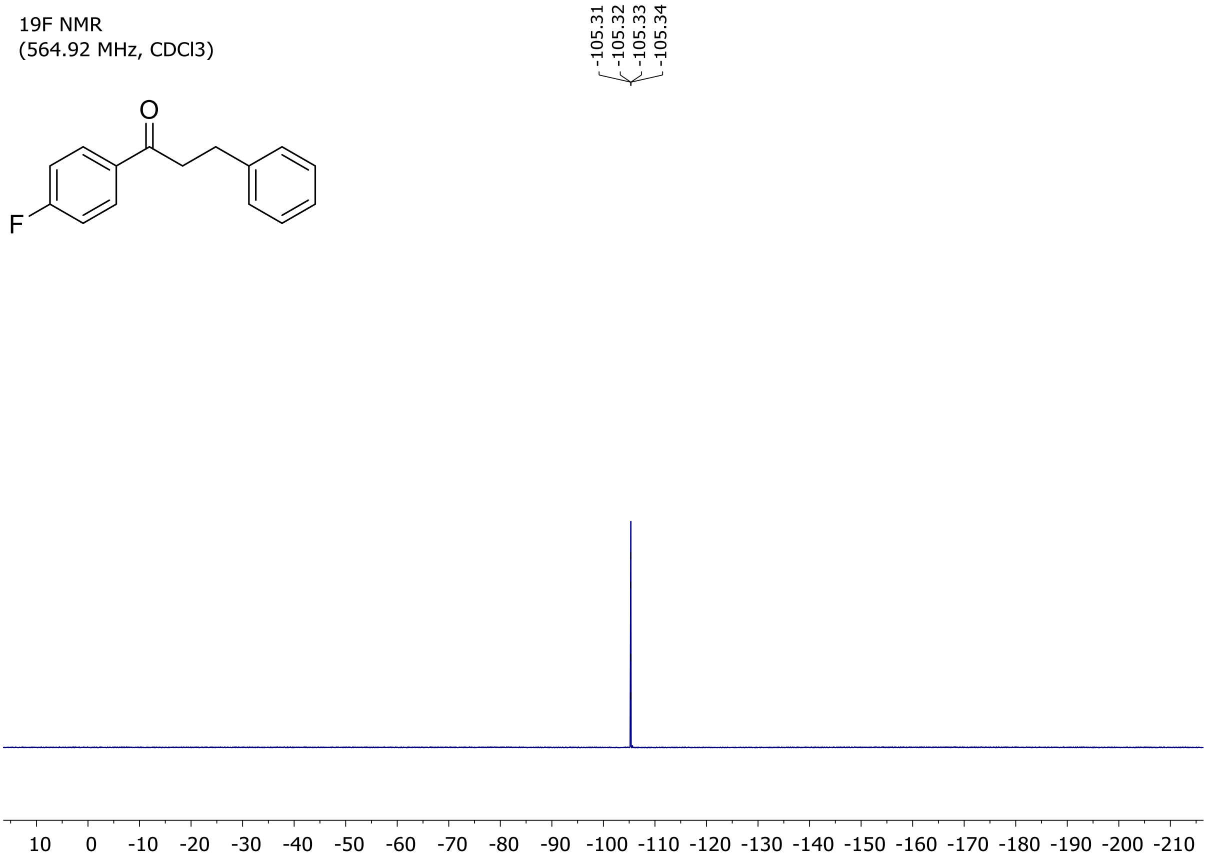


## 3-(4-chlorophenyl)-1-(4-fluorophenyl)propan-1-one (2f)

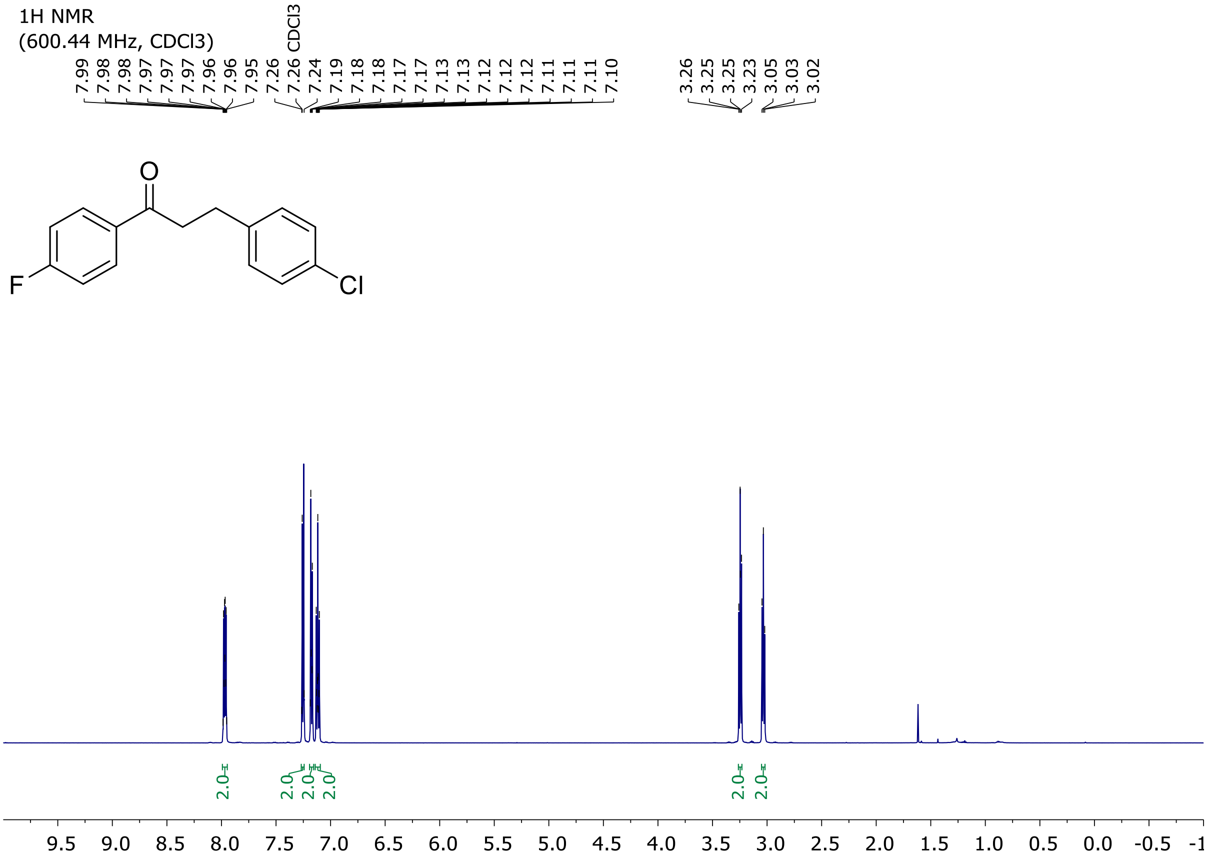

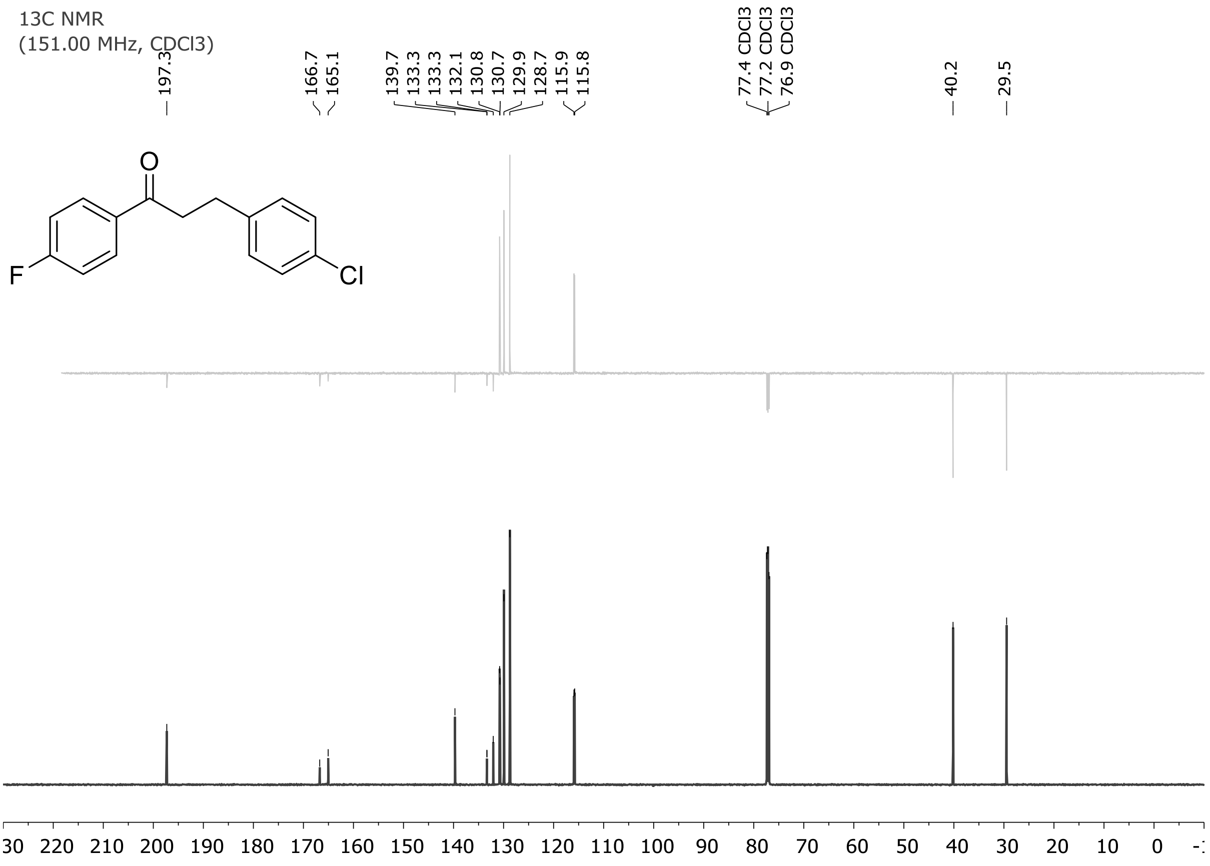
13C NMR
(151.00 MHz, CDCl3)
197.3
166.7
165.1
139.7
133.3
133.3
132.1
130.8
130.7
129.9
128.7
115.9
115.8
77.4 CDCl3
77.2 CDCl3
76.9 CDCl3
40.2
29.5
O
F
Cl
30 220 210 200 190 180 170 160 150 140 130 120 110 100 90 80 70 60 50 40 30 20 10 0

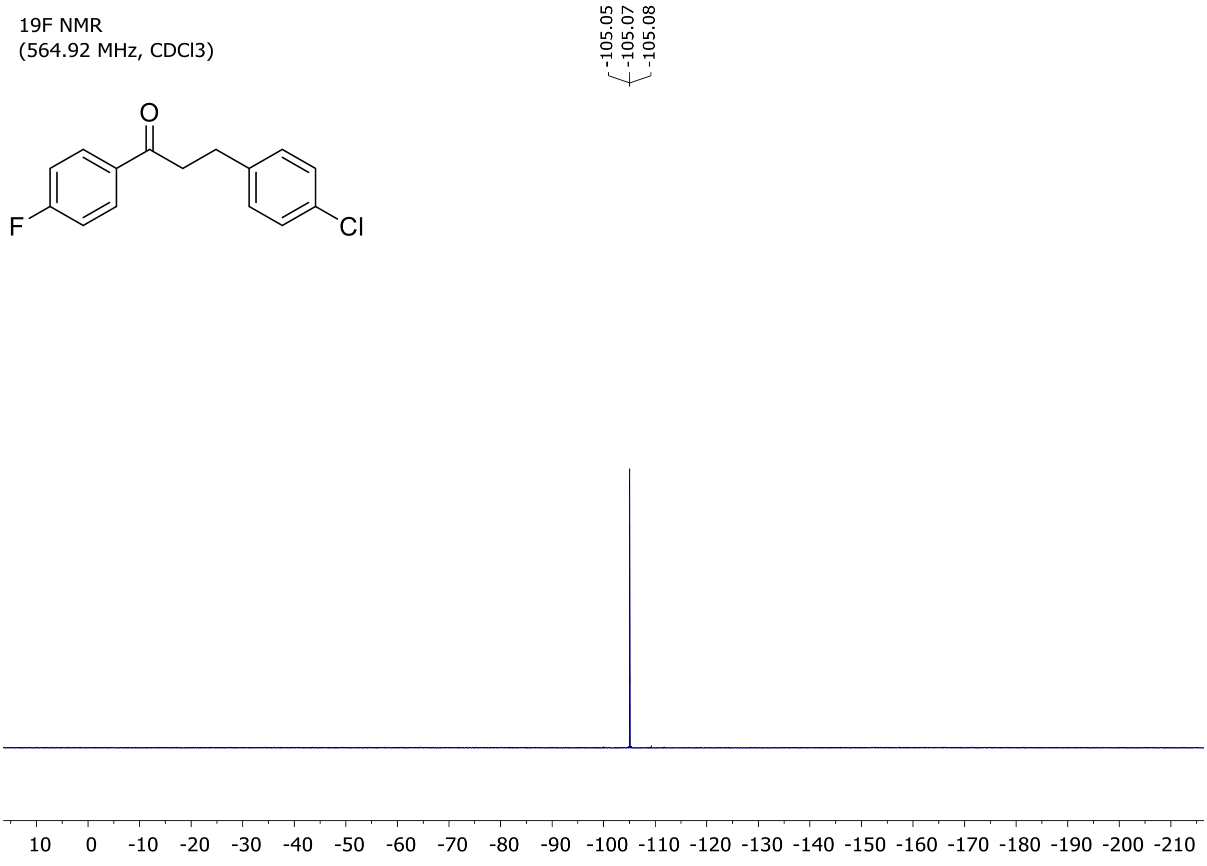
19F NMR
(564.92 MHz, CDCl3)
-105.05
-105.07
-105.08
O
F
Cl
10 0 -10 -20 -30 -40 -50 -60 -70 -80 -90 -100 -110 -120 -130 -140 -150 -160 -170 -180 -190 -200 -210

**4-phenylbutan-2-one (2g)**

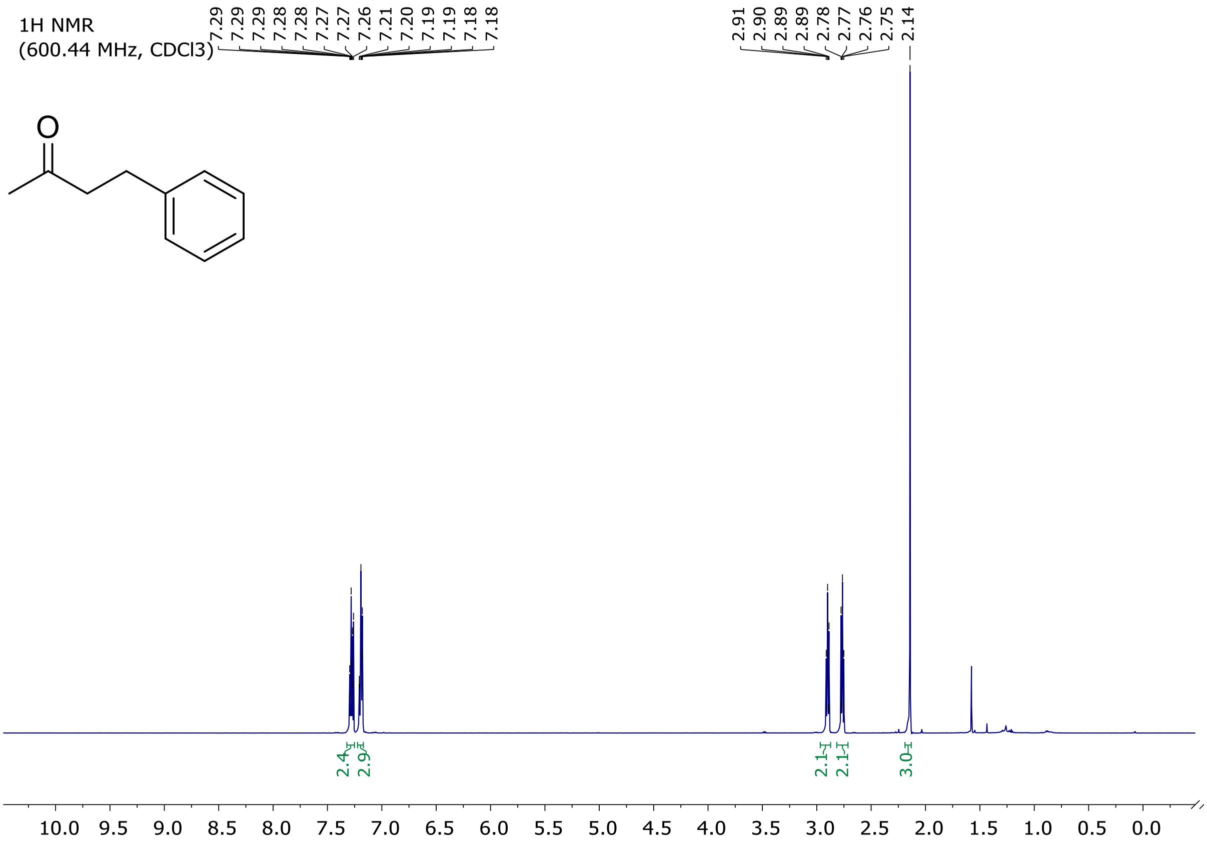


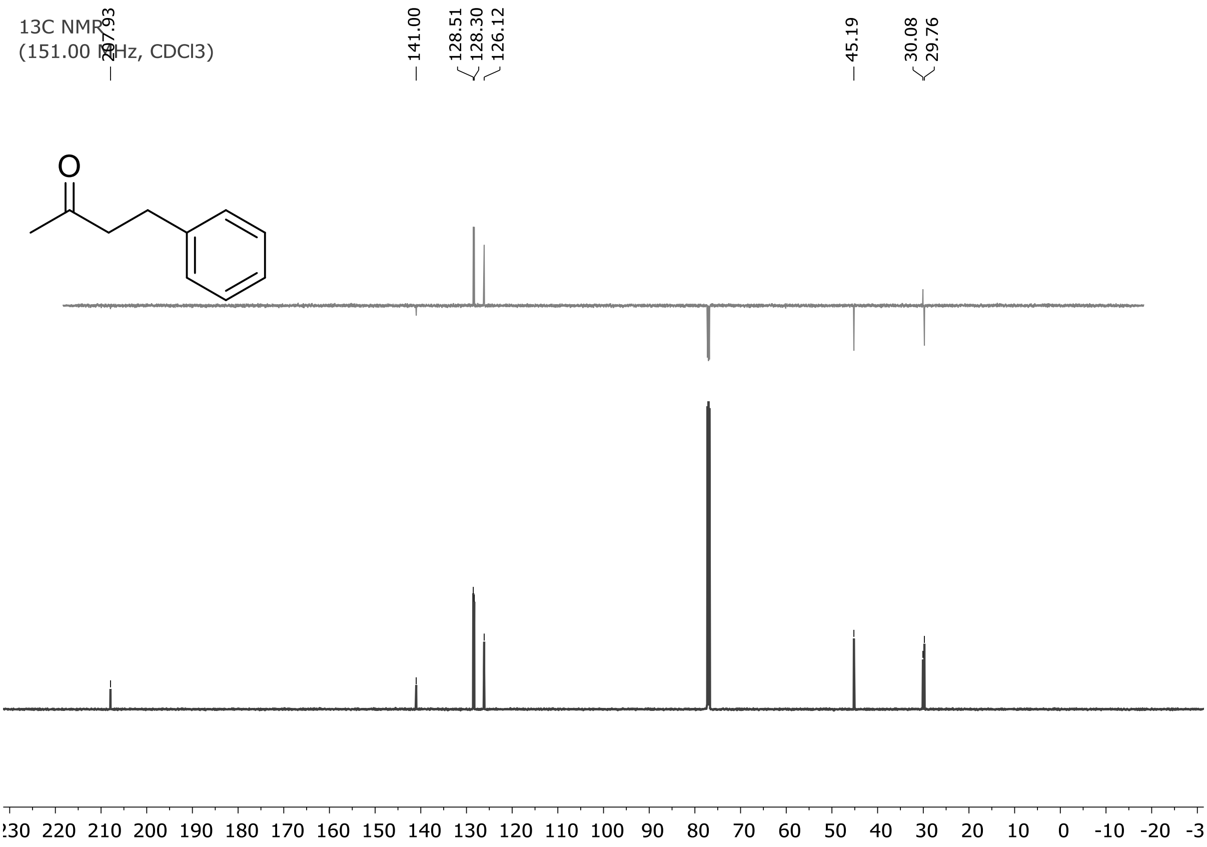

## Nonan-2-one (2h)

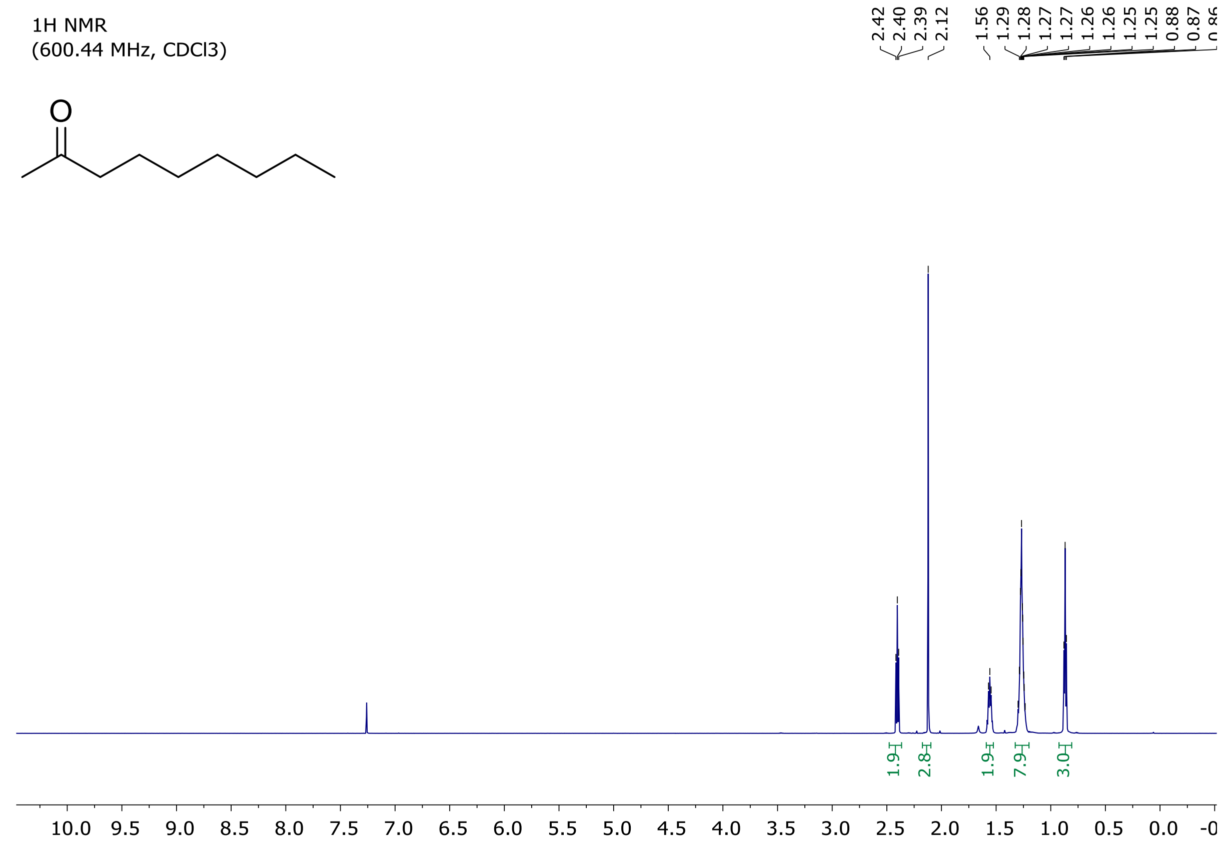


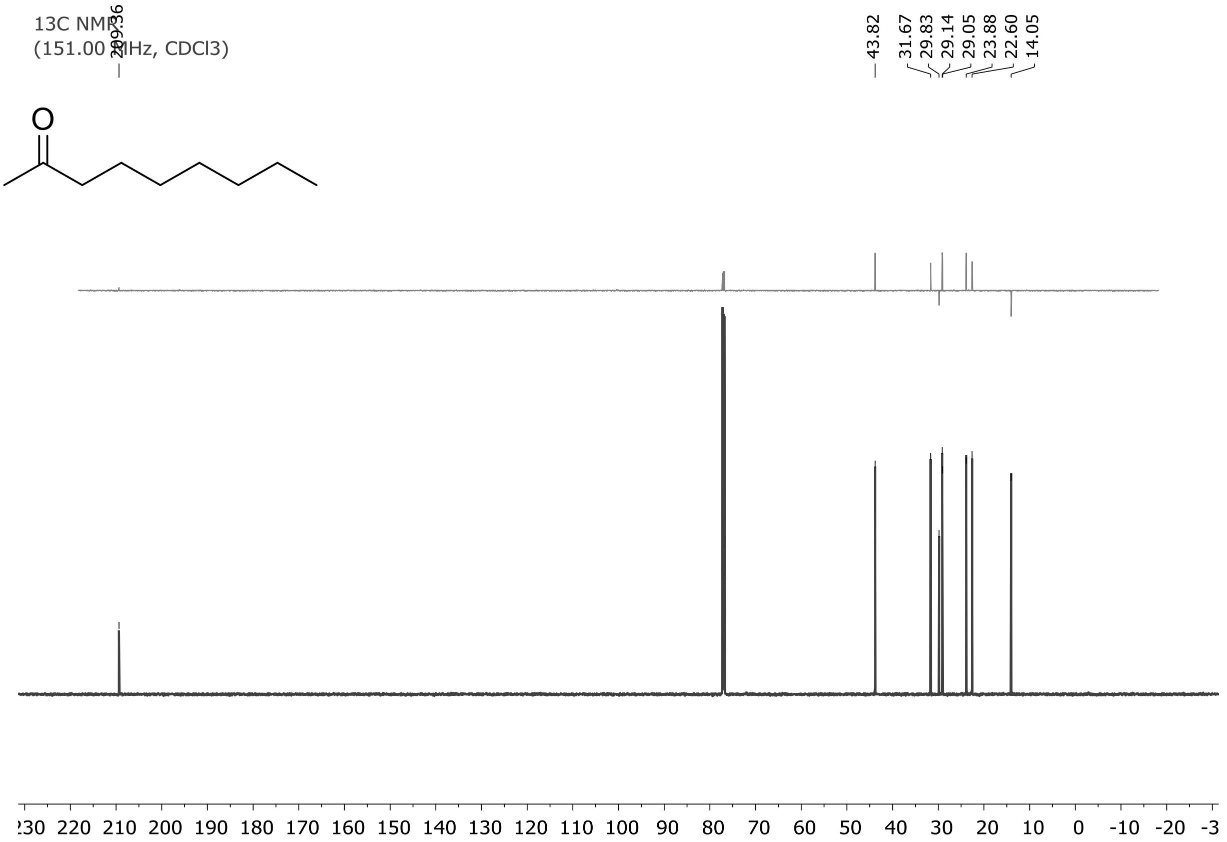

## 4-(2,6,6-trimethylcyclohex-1-en-1-yl)butan-2-one (2i)

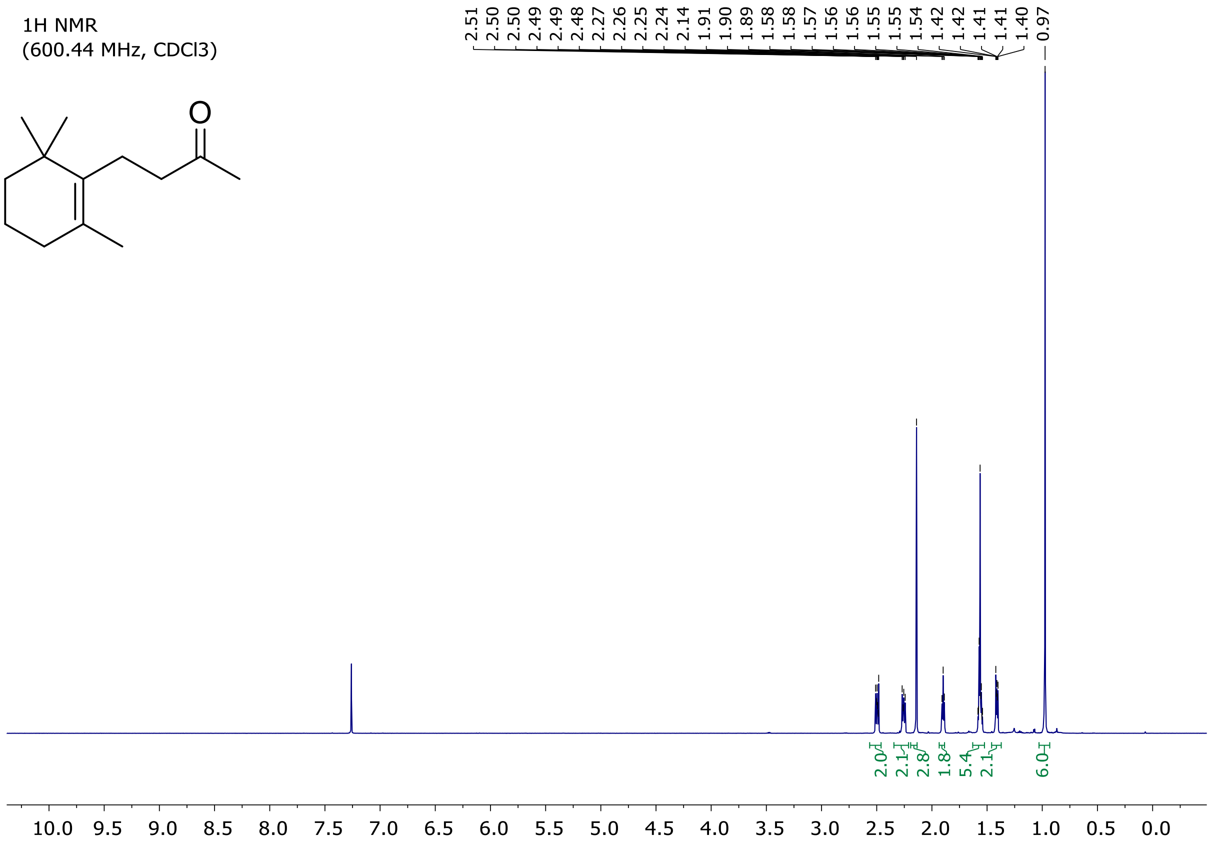


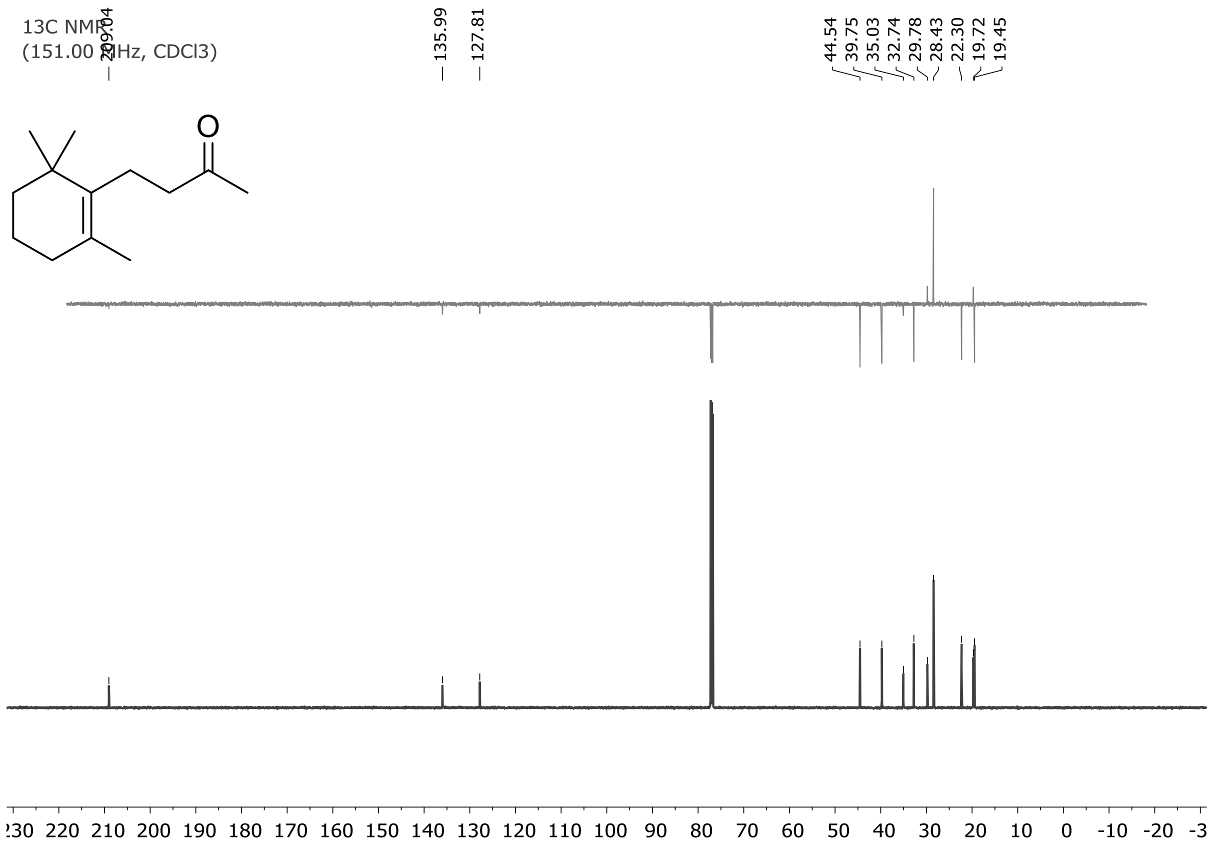

## 1-phenylbutan-1-one (2j)

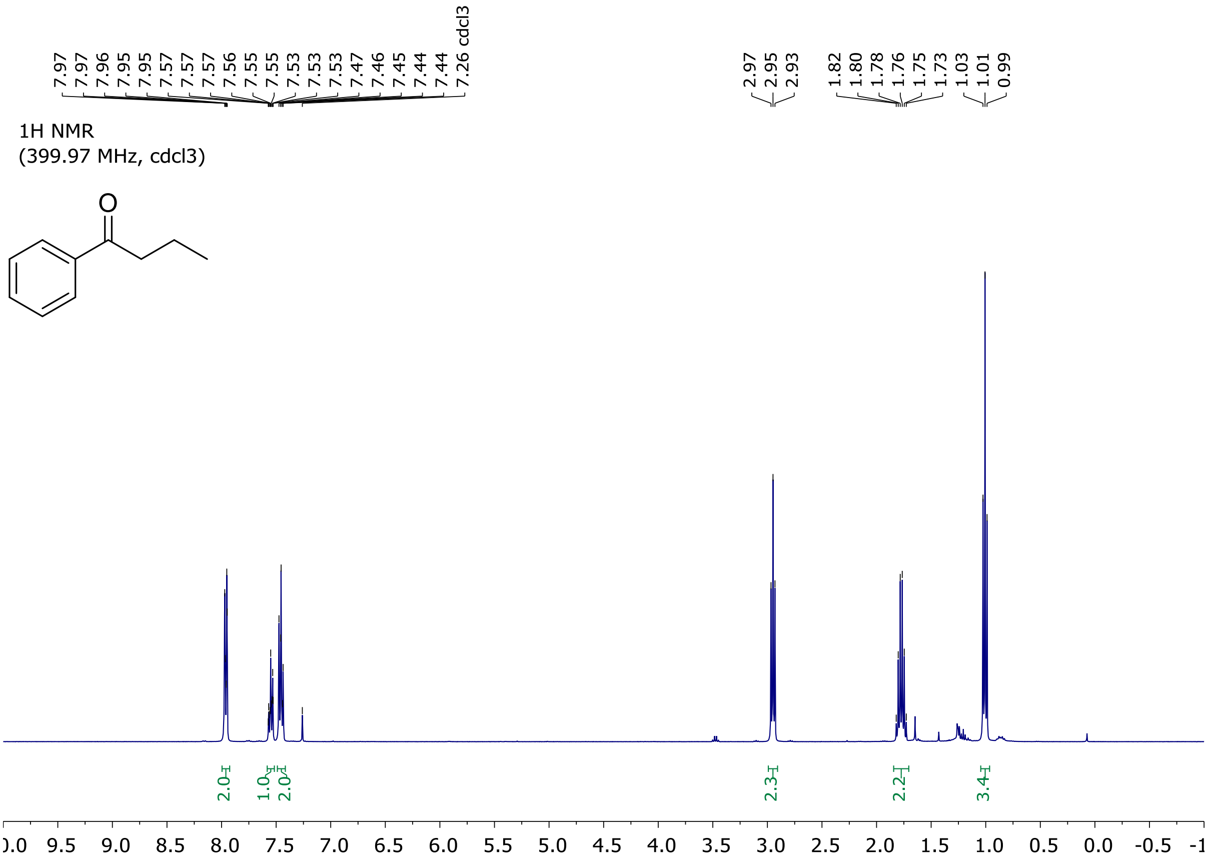


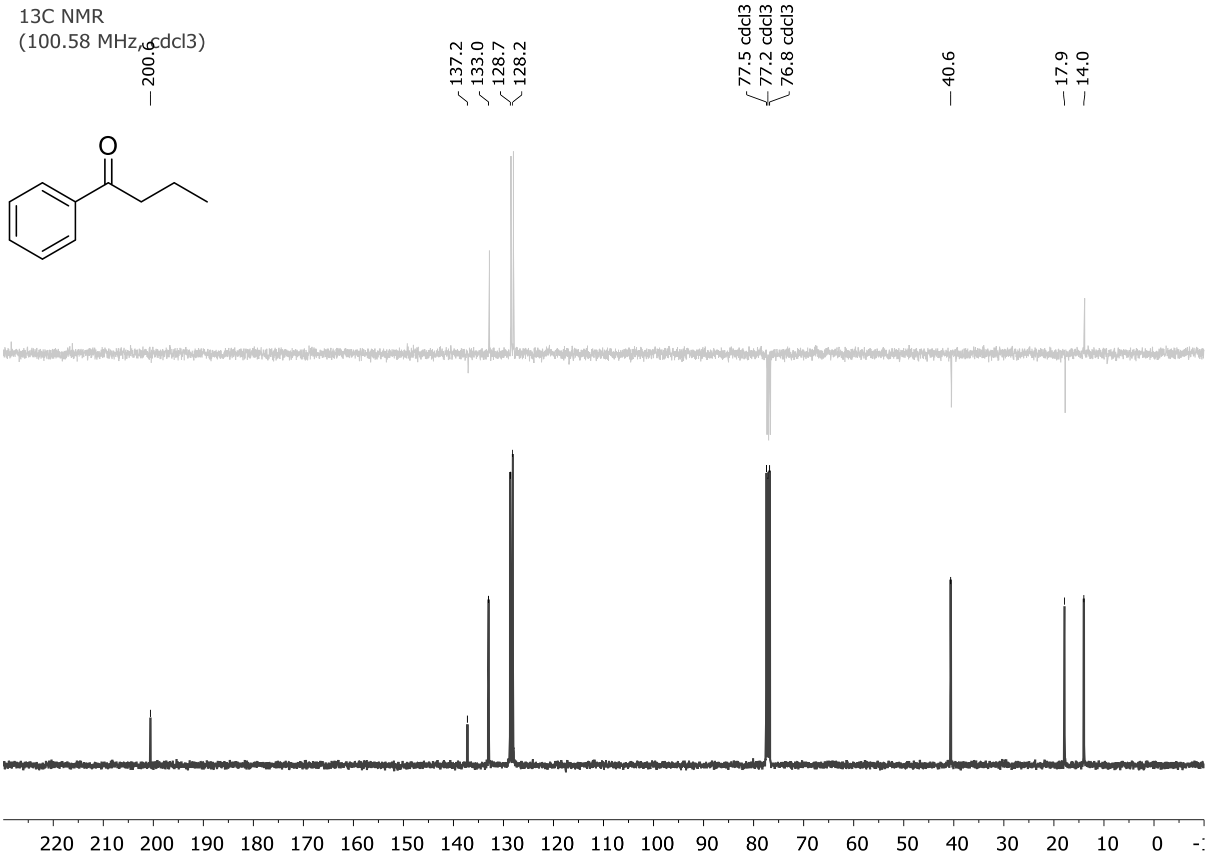

# 4,4-dimethylcyclohexan-1-one (2k)

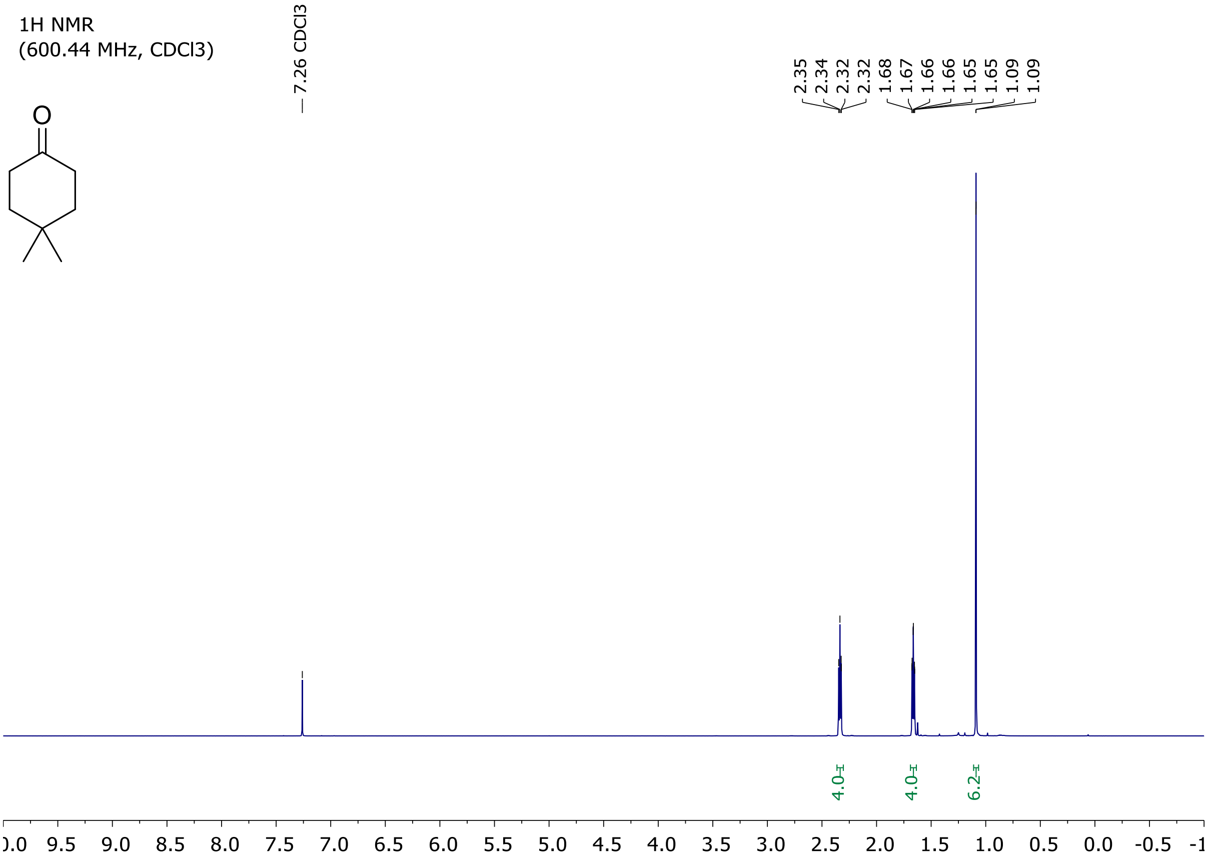


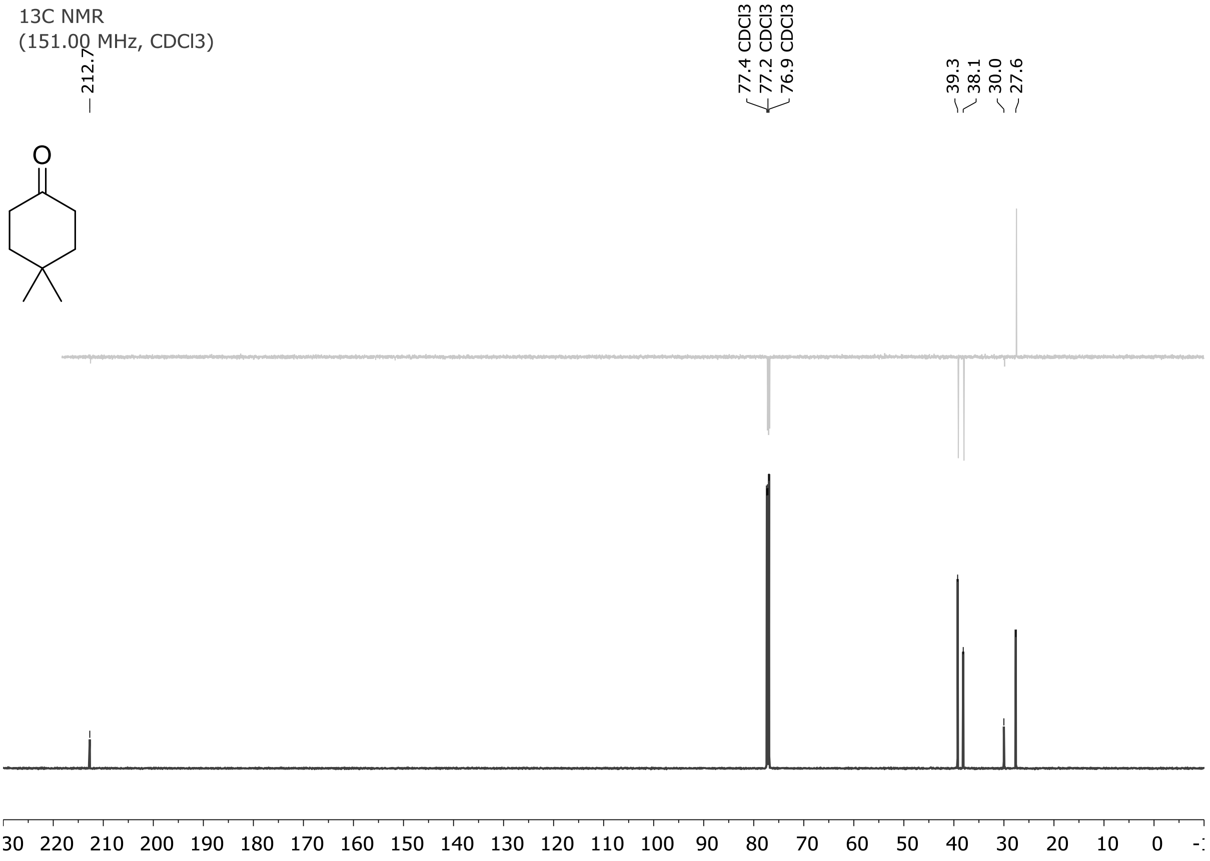

## ethyl 2-(2-oxocyclohexyl)acetate (2l)

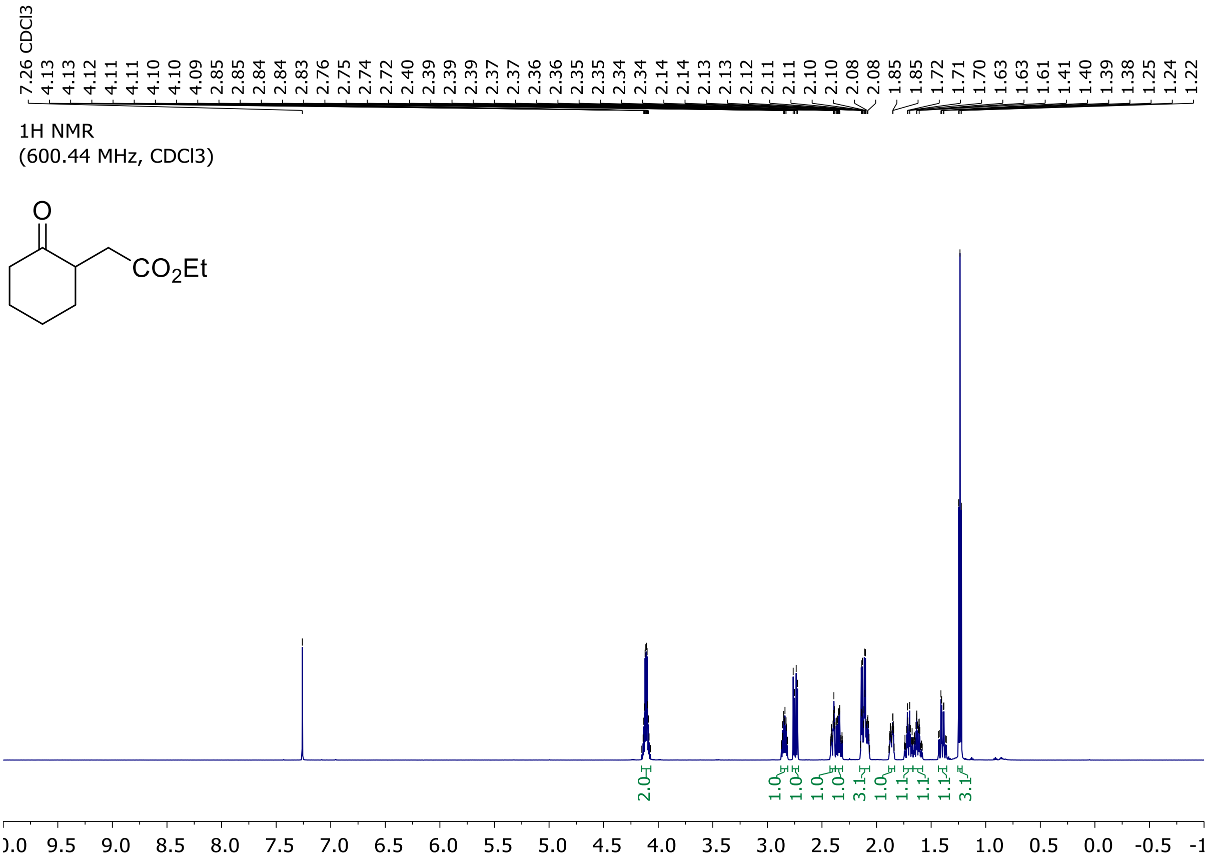


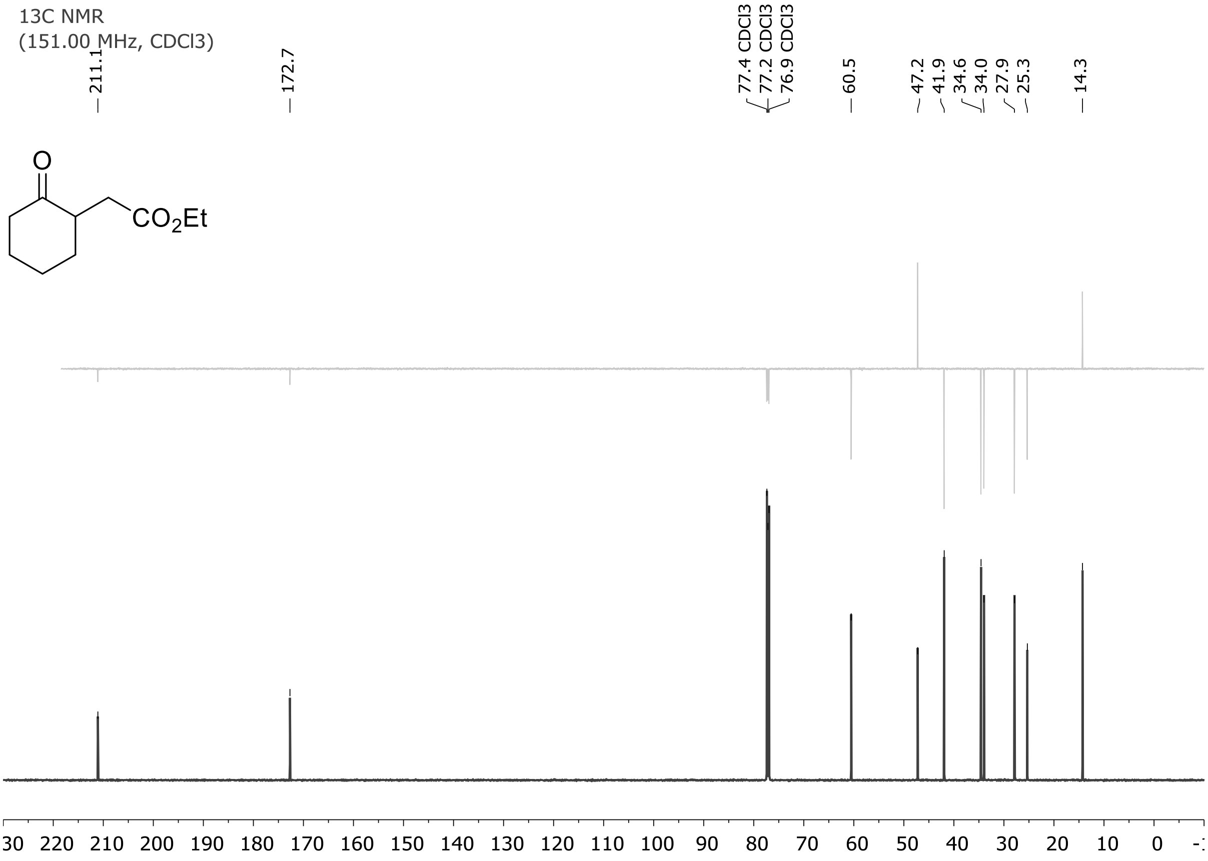

## 4-phenylcyclohexan-1-one (2m)

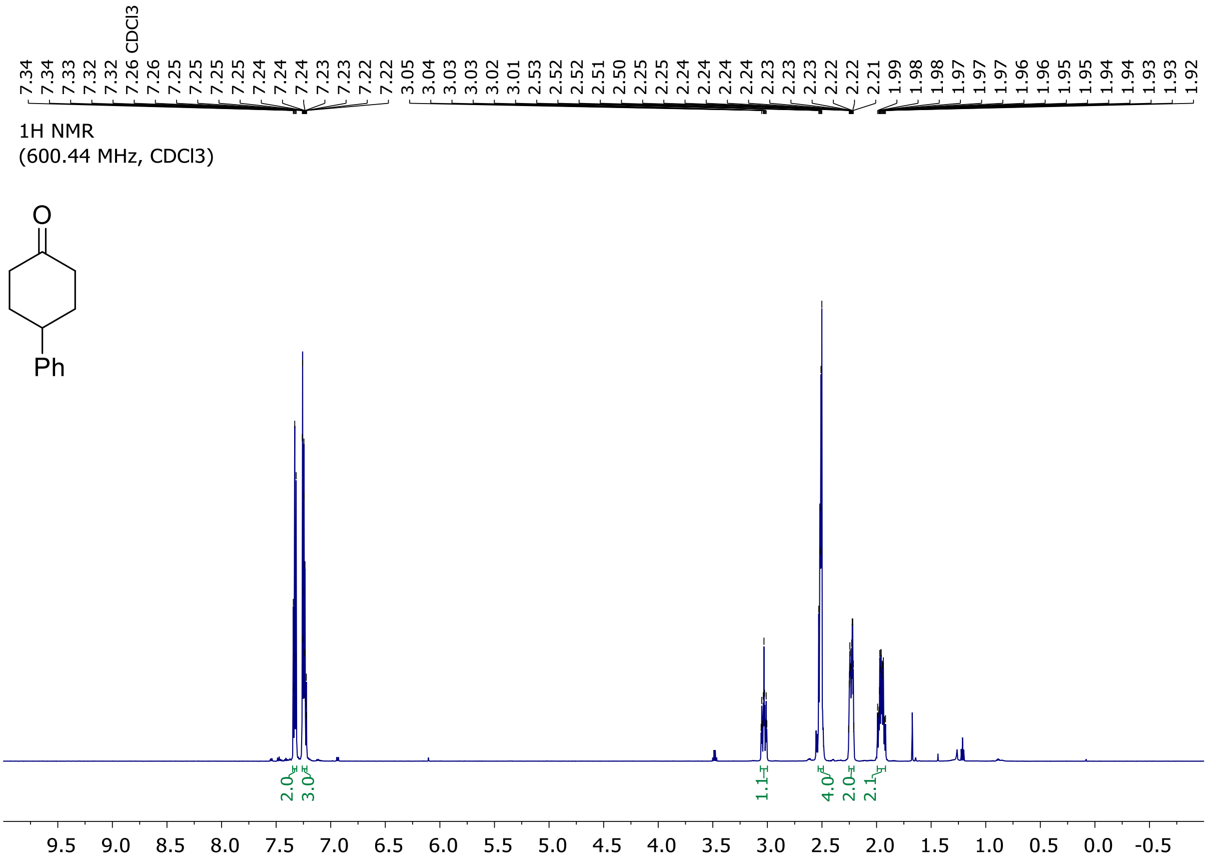


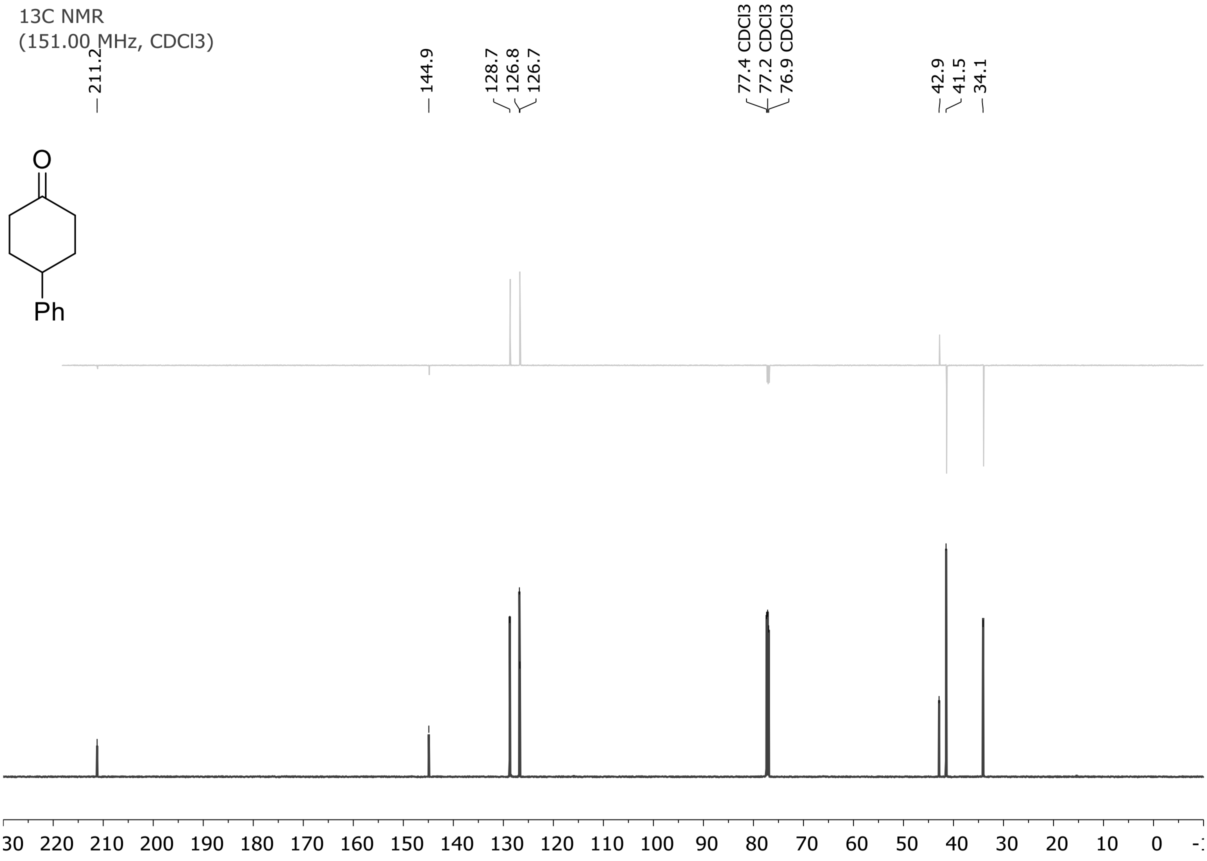

# 4-(tert-butyl)cyclohexan-1-one (2n)

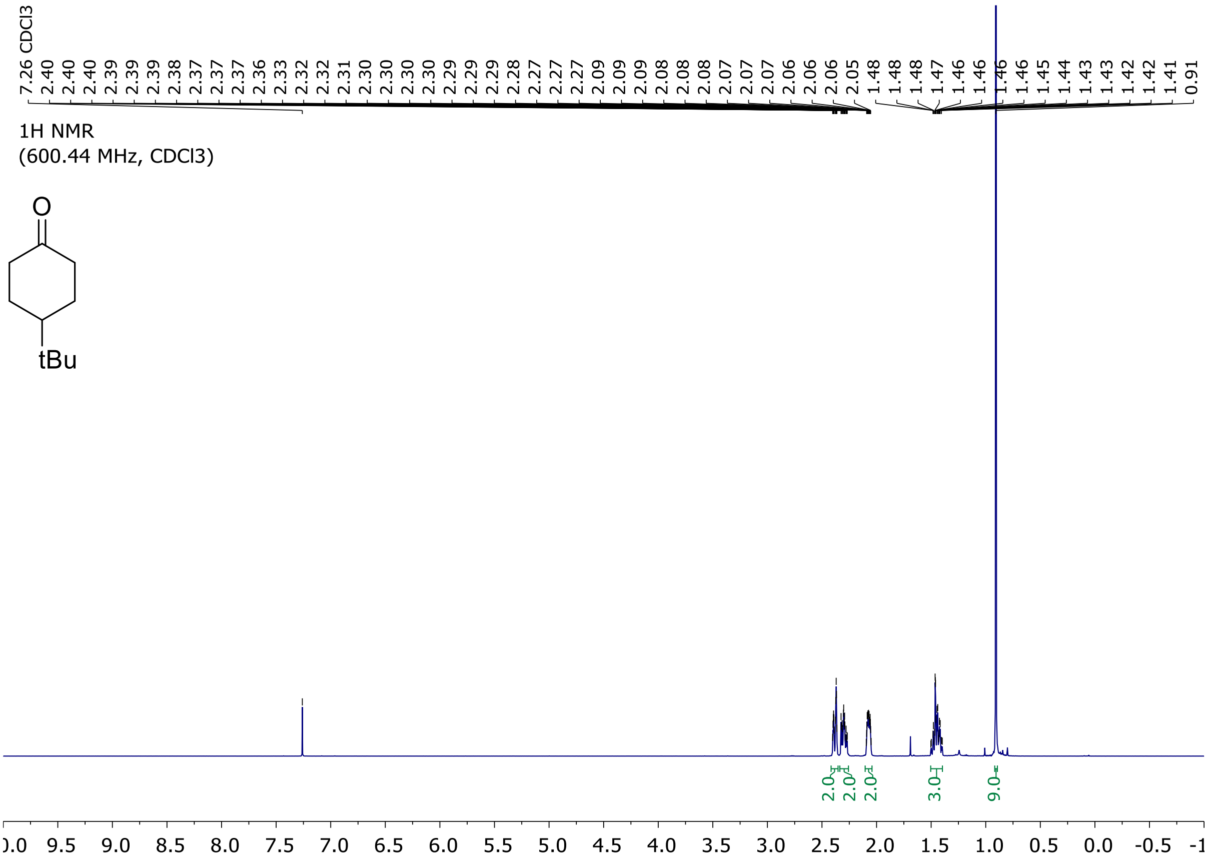


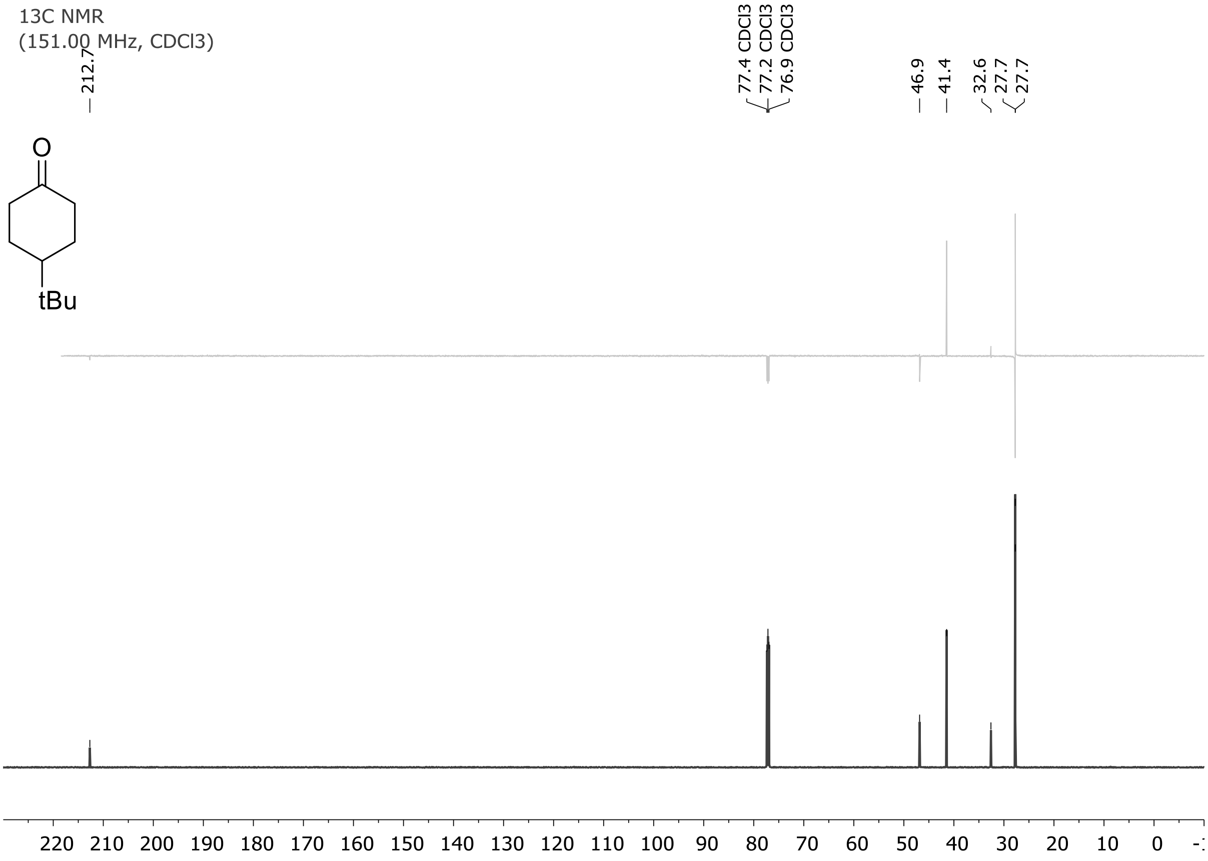

## (2R,5R)-2-methyl-5-(prop-1-en-2-yl)cyclohexan-1-one (2o)

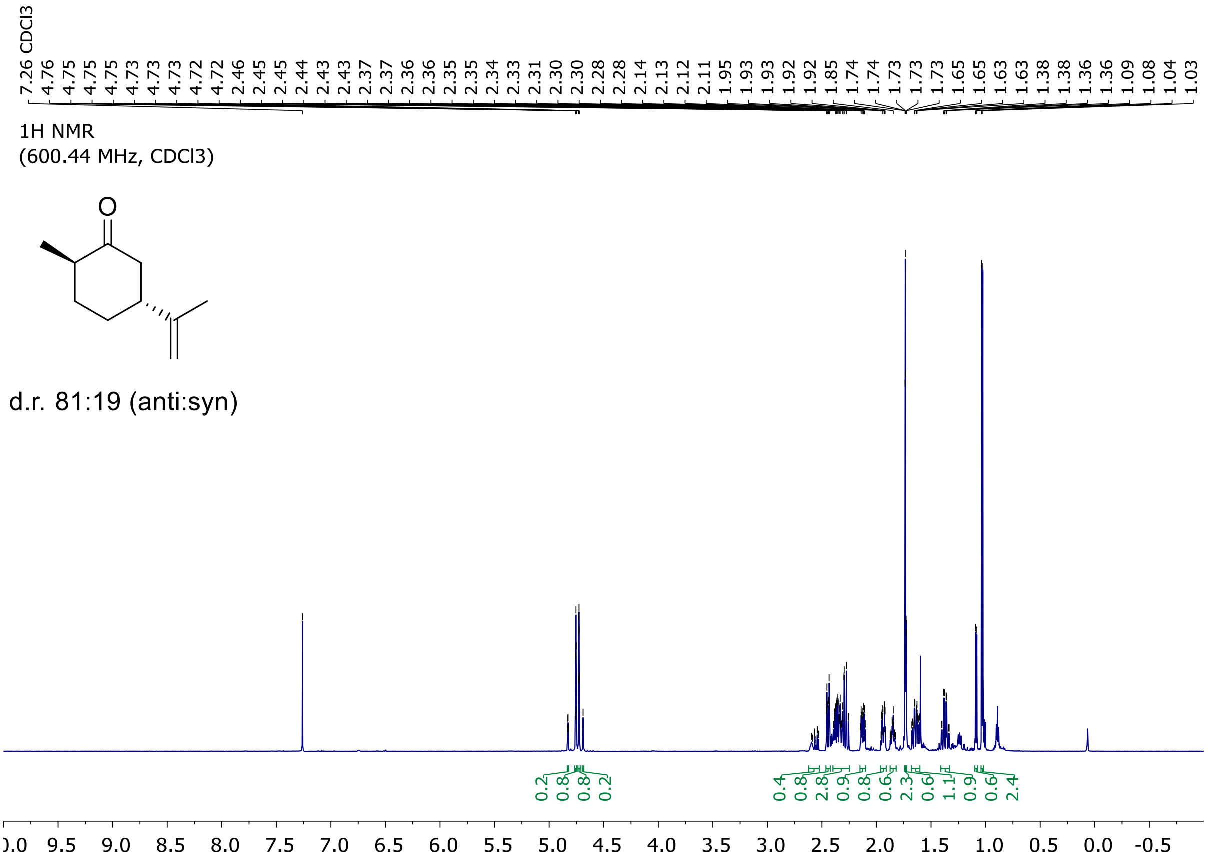


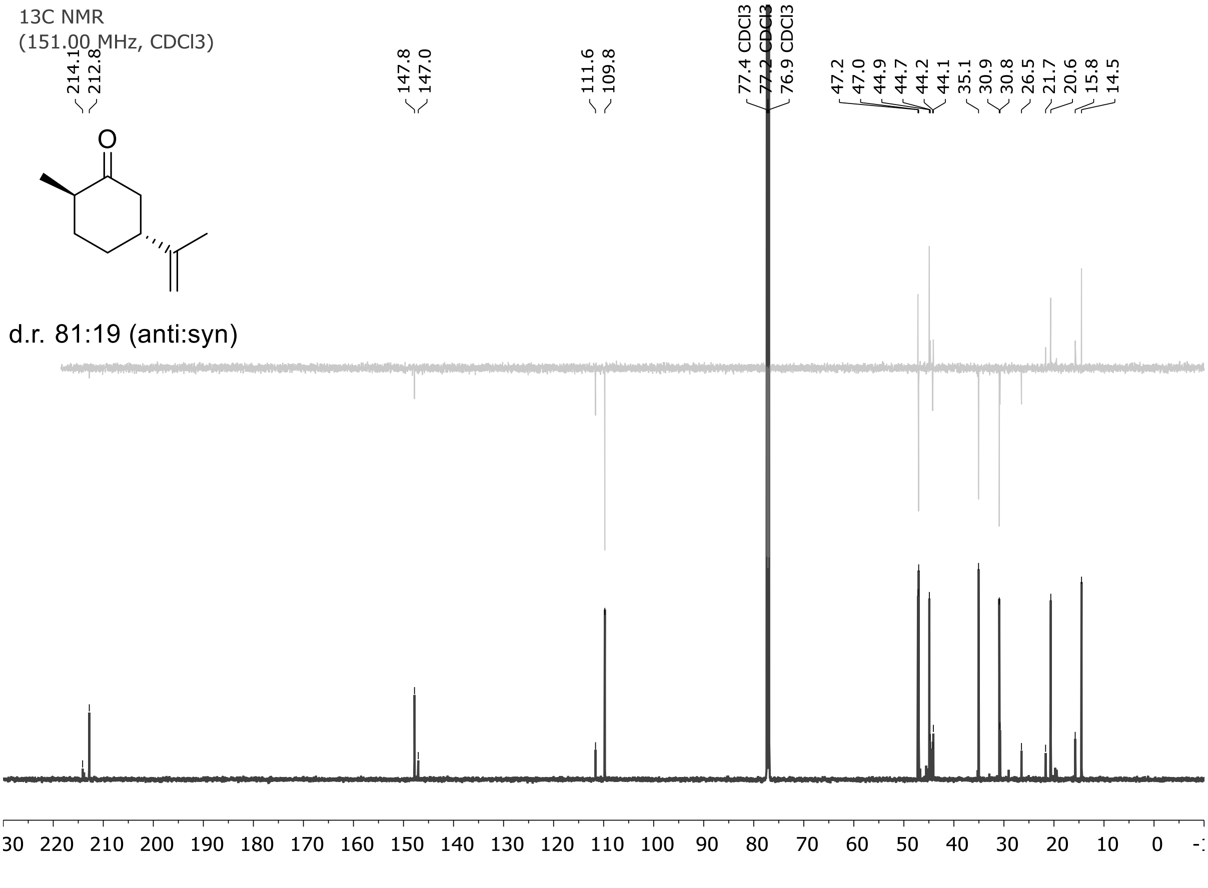

## (E)-1,3-diphenylprop-2-en-1-ol (3a)

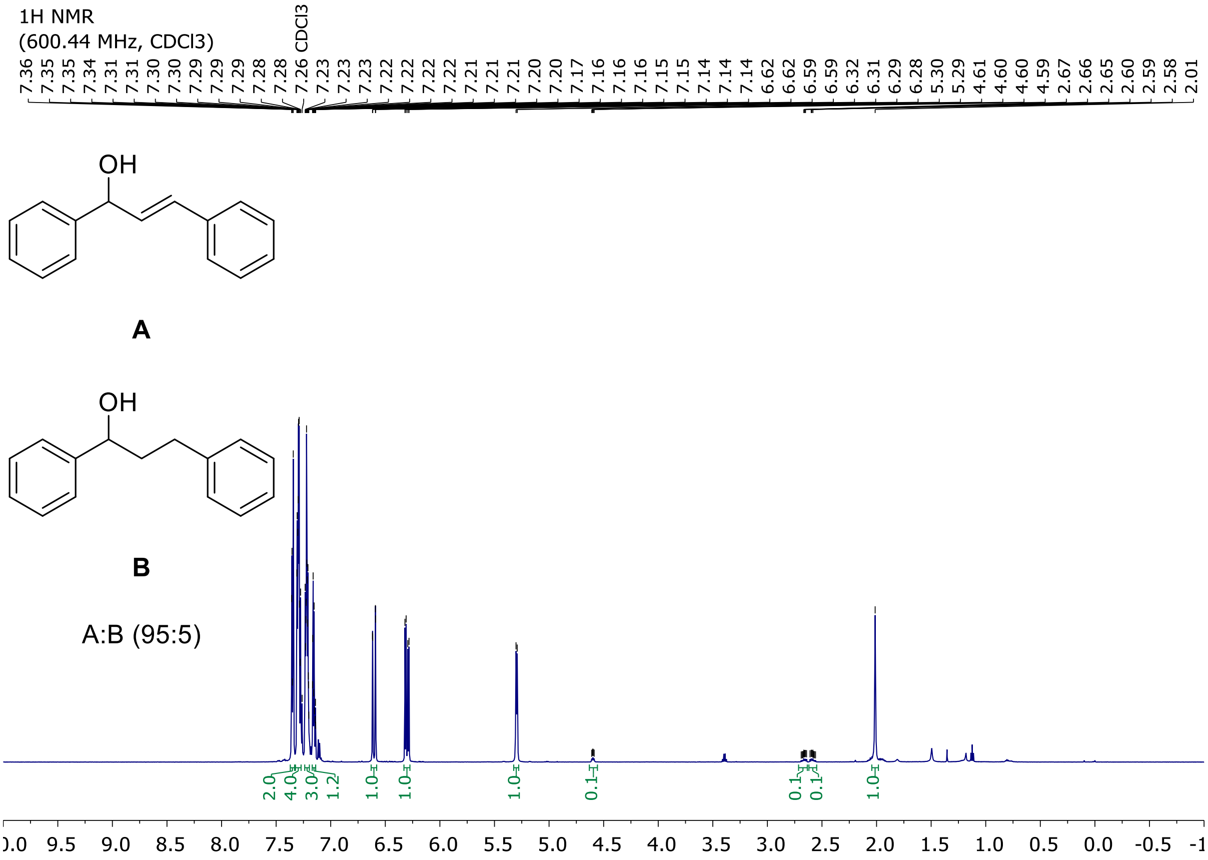


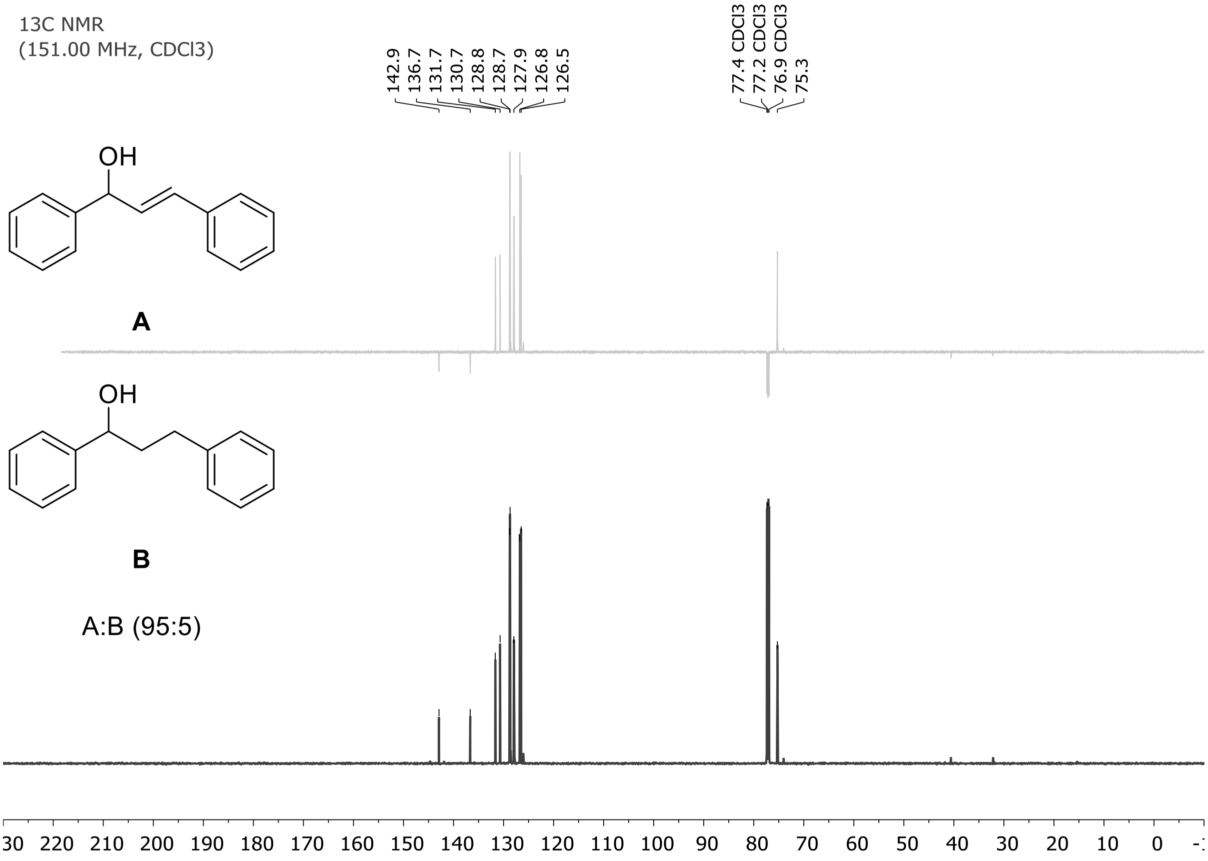

## (E)-3-(4-methoxyphenyl)-1-phenylprop-2-en-1-ol (3b)

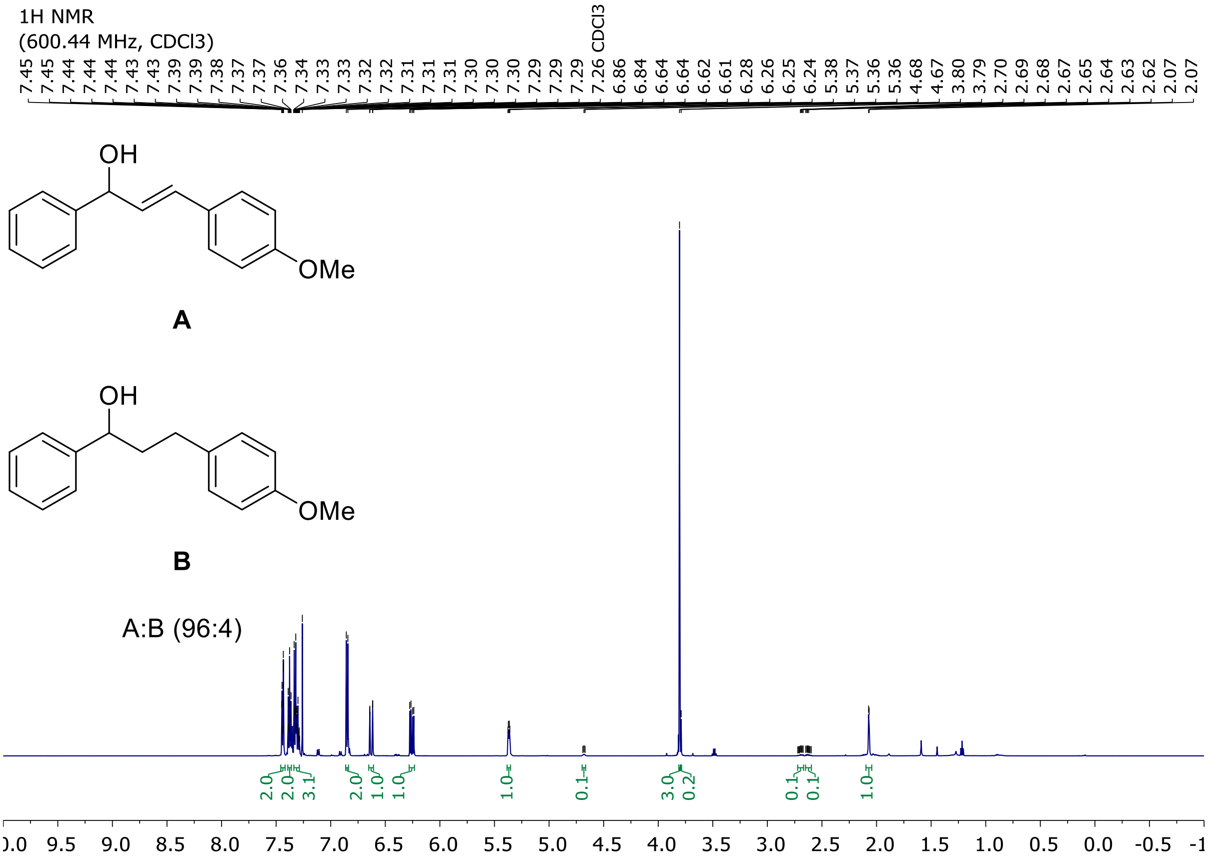


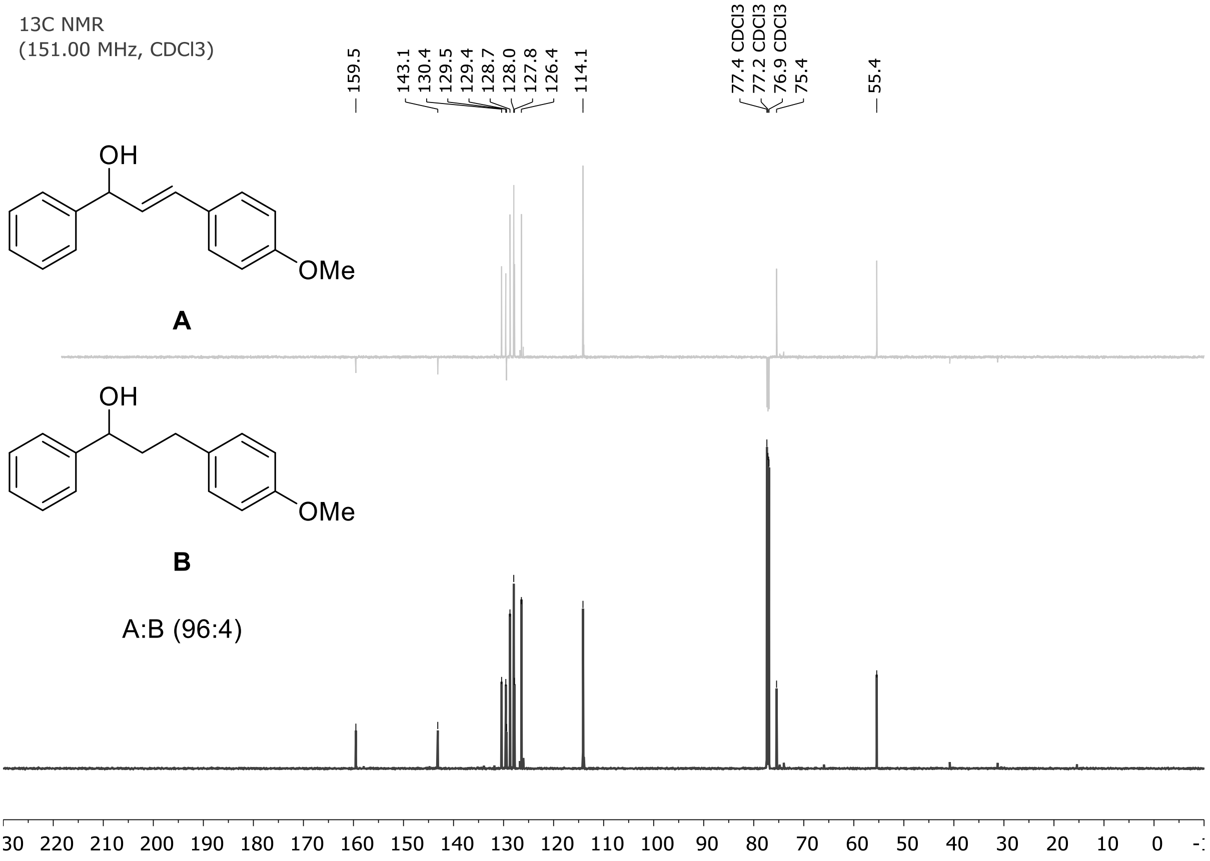

**methyl (E)-4-(3-hydroxy-3-phenylprop-1-en-1-yl)benzoate (3c)**

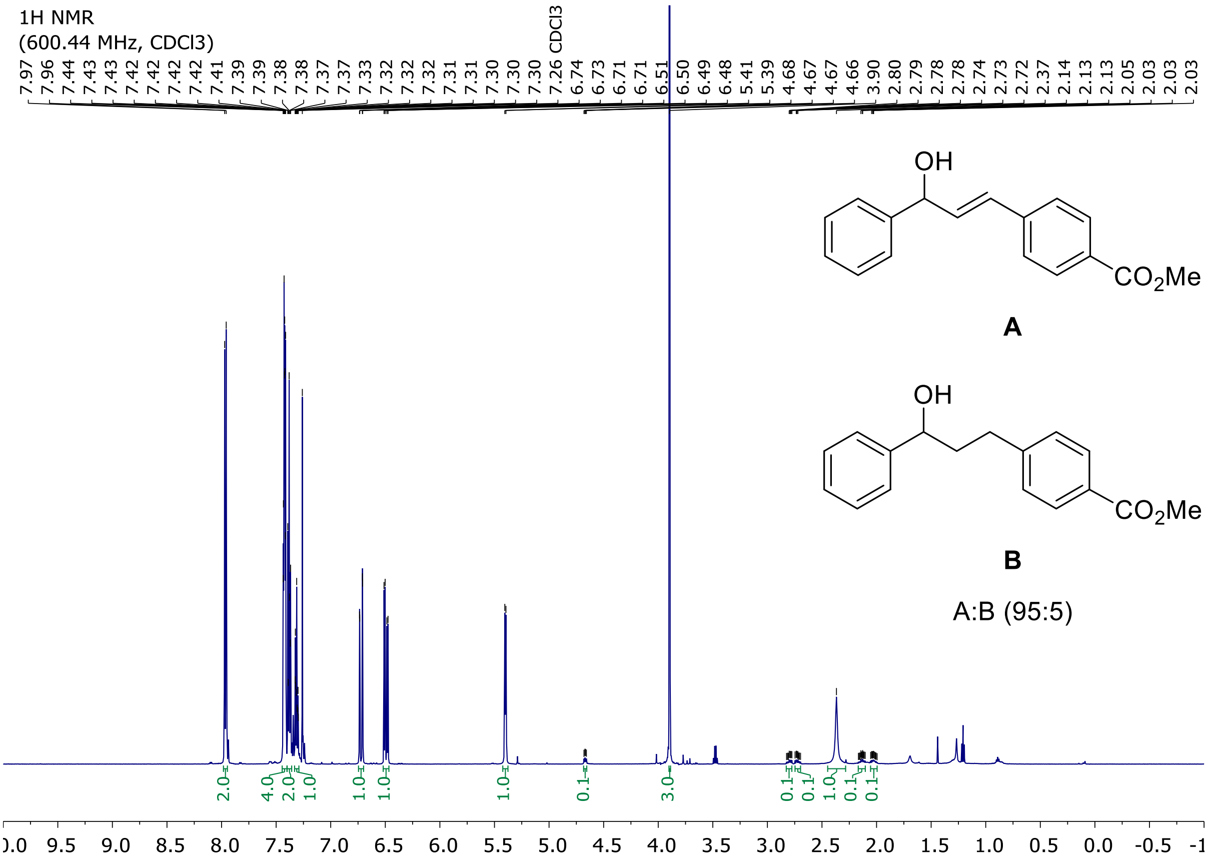


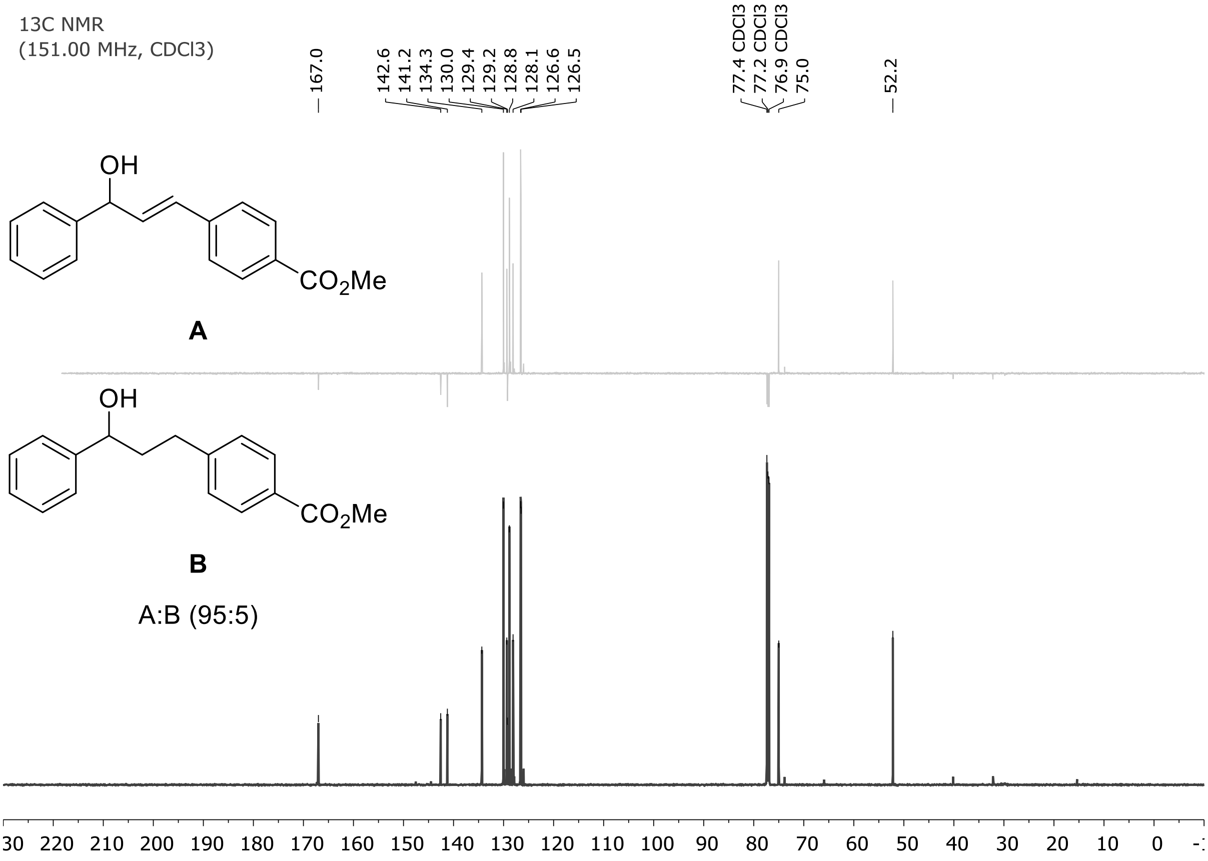

**(E)-3-(4-chlorophenyl)-1-phenylprop-2-en-1-ol (3d)**

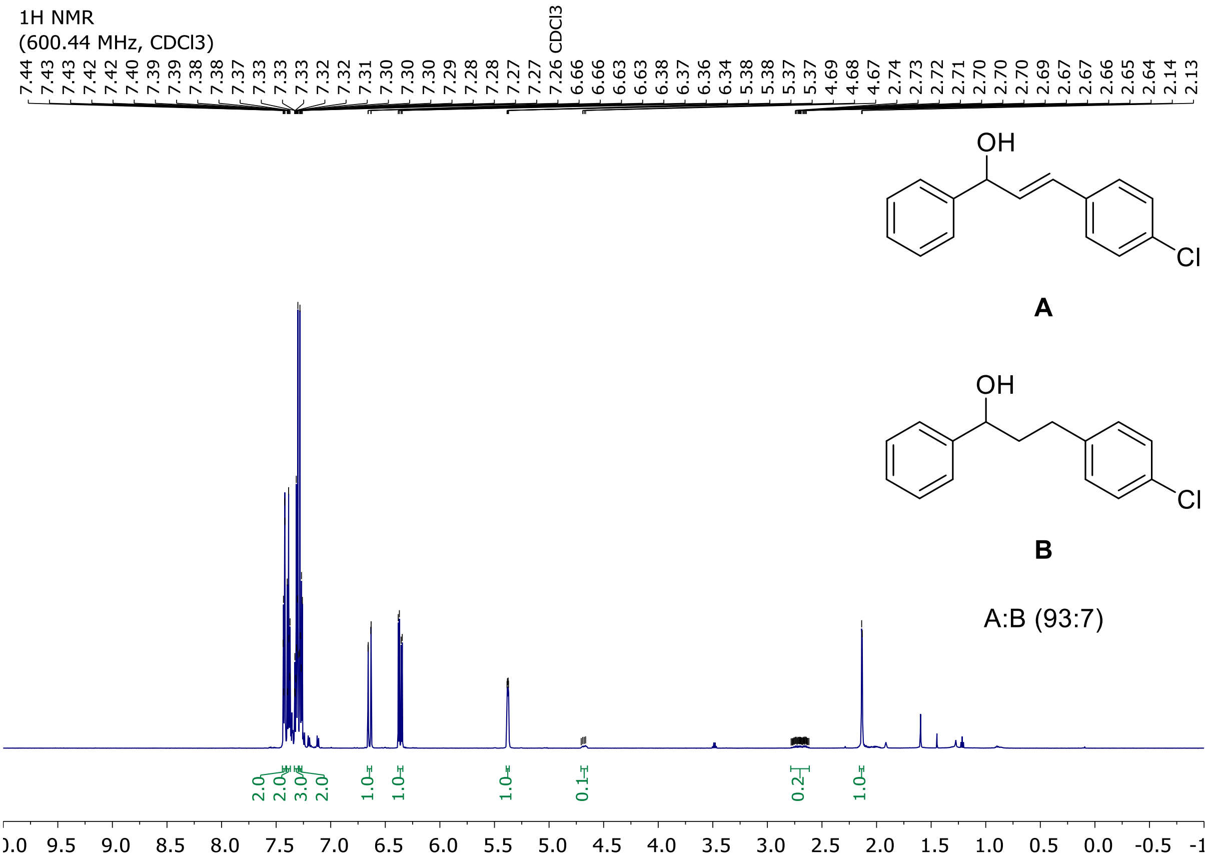


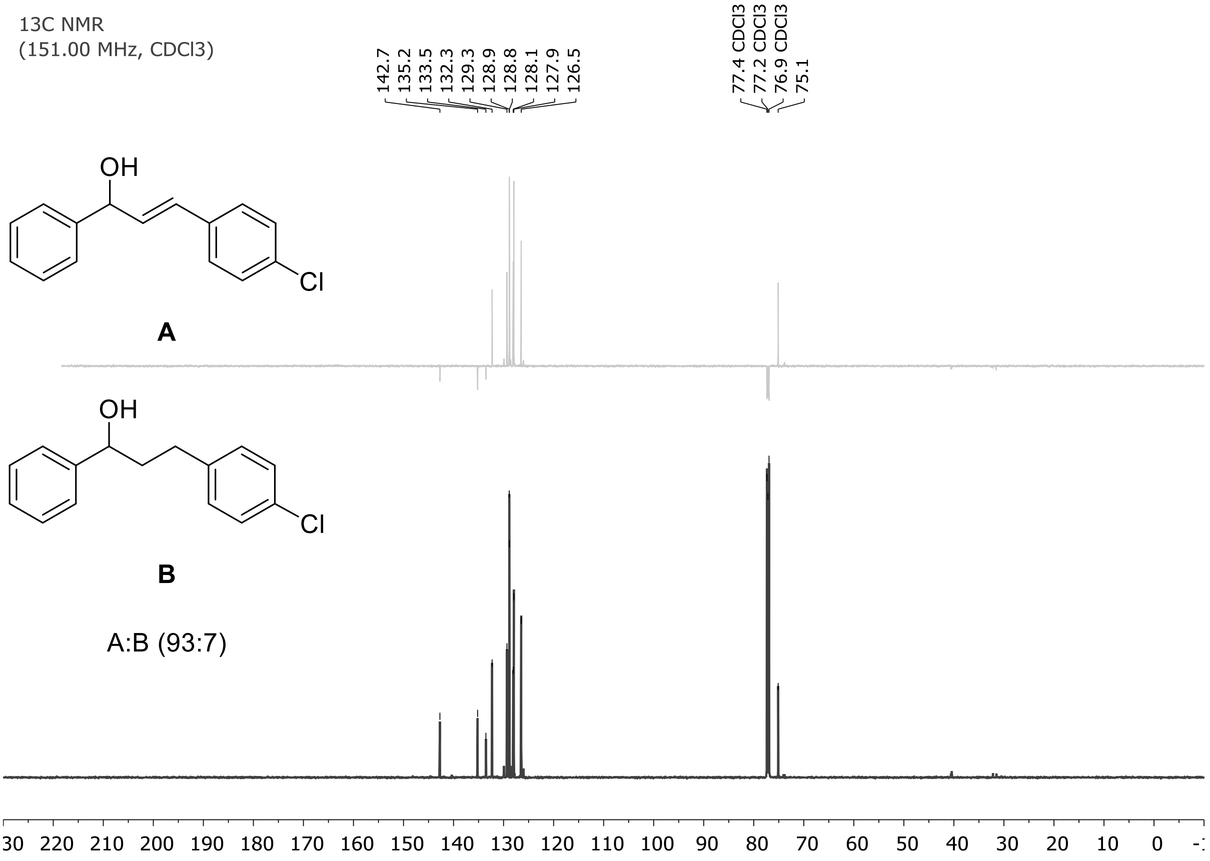

## (E)-1-(4-fluorophenyl)-3-phenylprop-2-en-1-ol (3e)

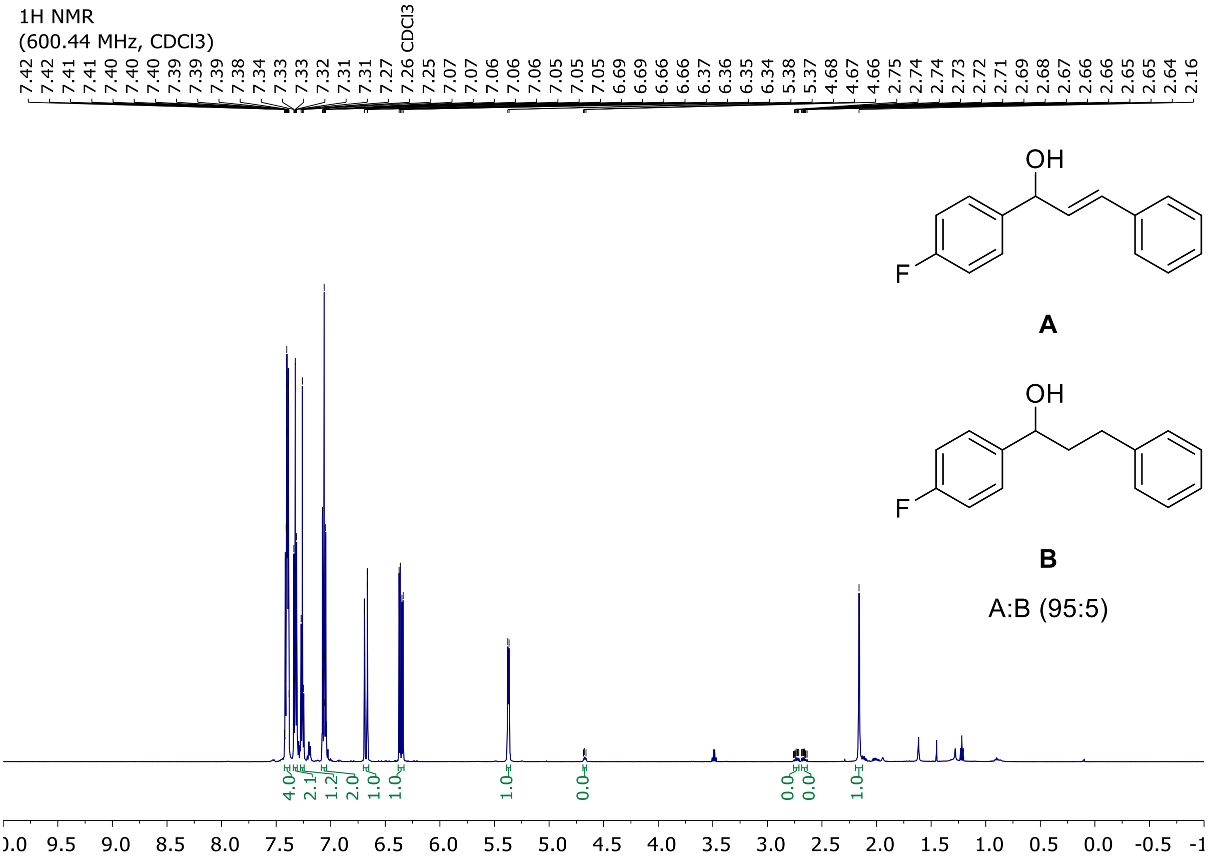


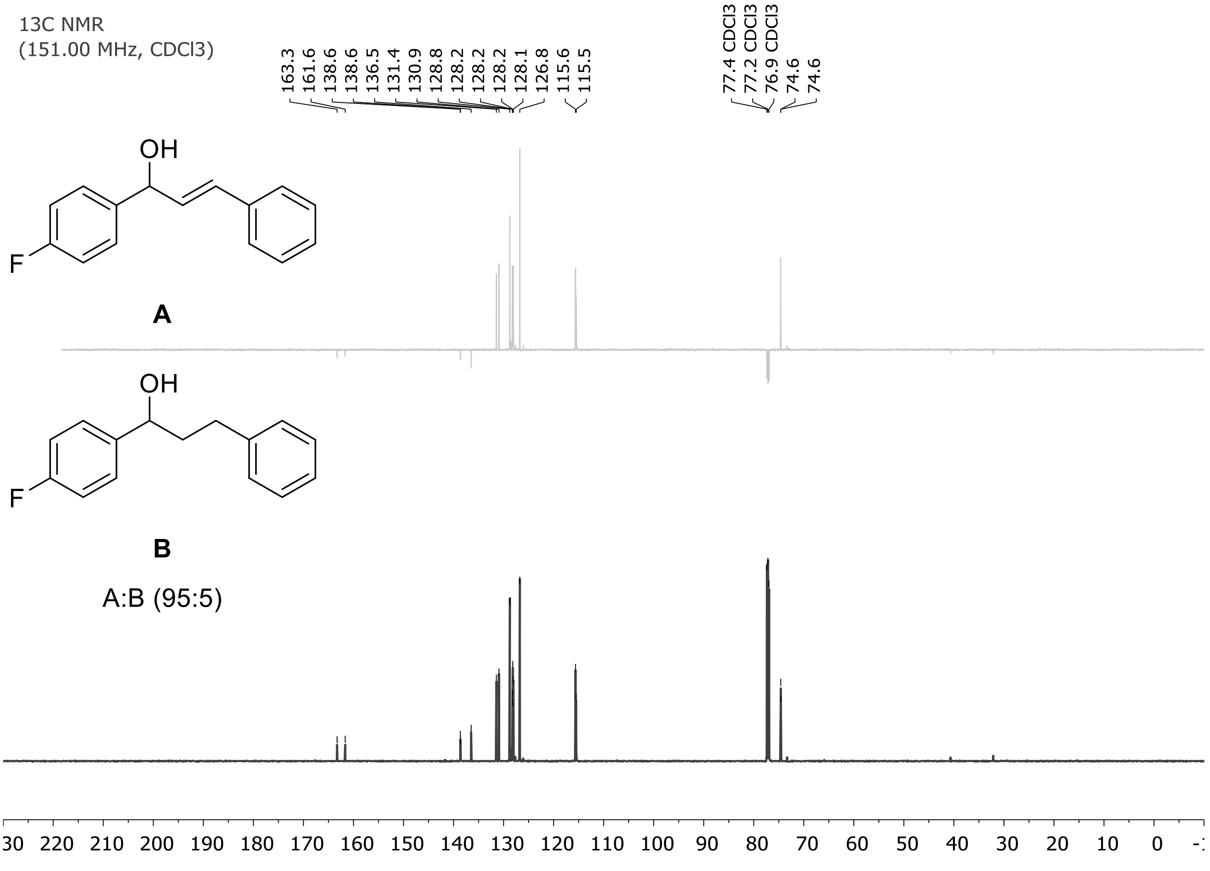

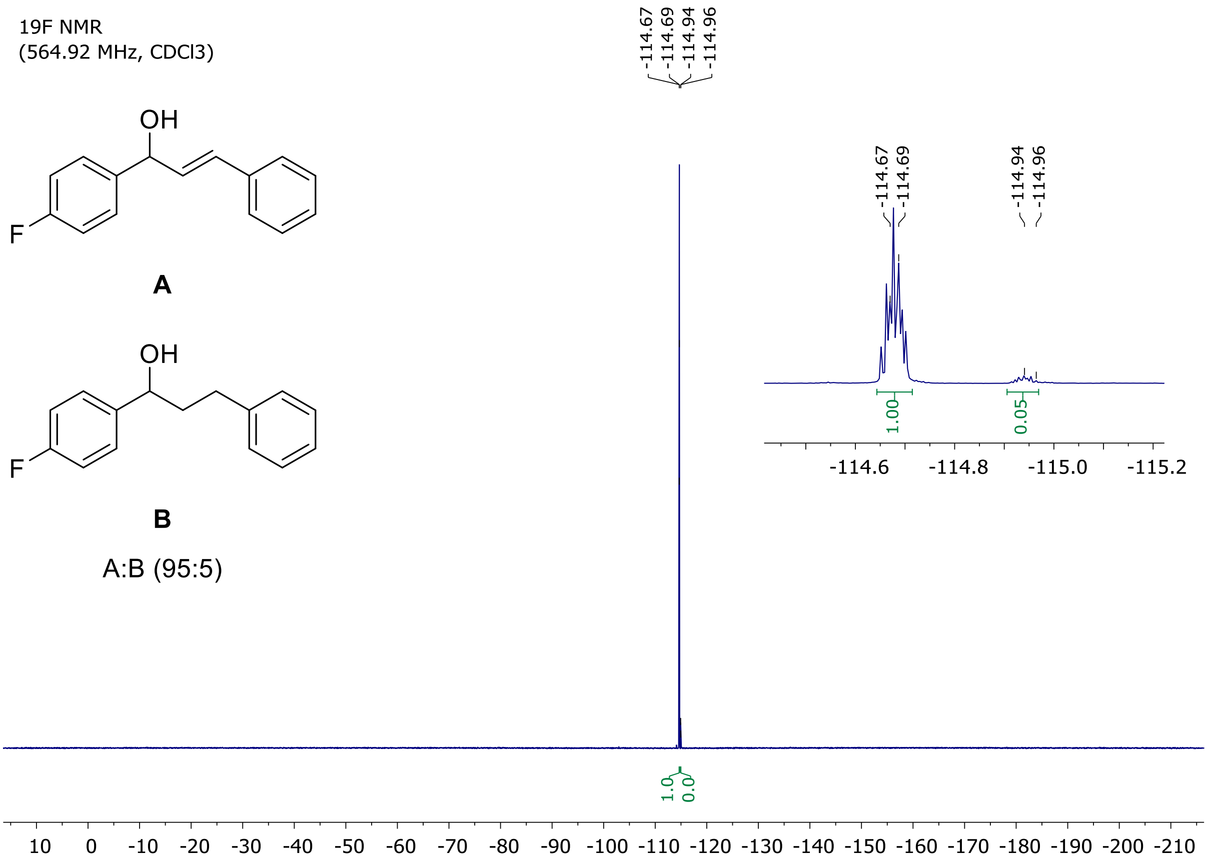


## (E)-3-(4-chlorophenyl)-1-(4-fluorophenyl)prop-2-en-1-ol (3f)

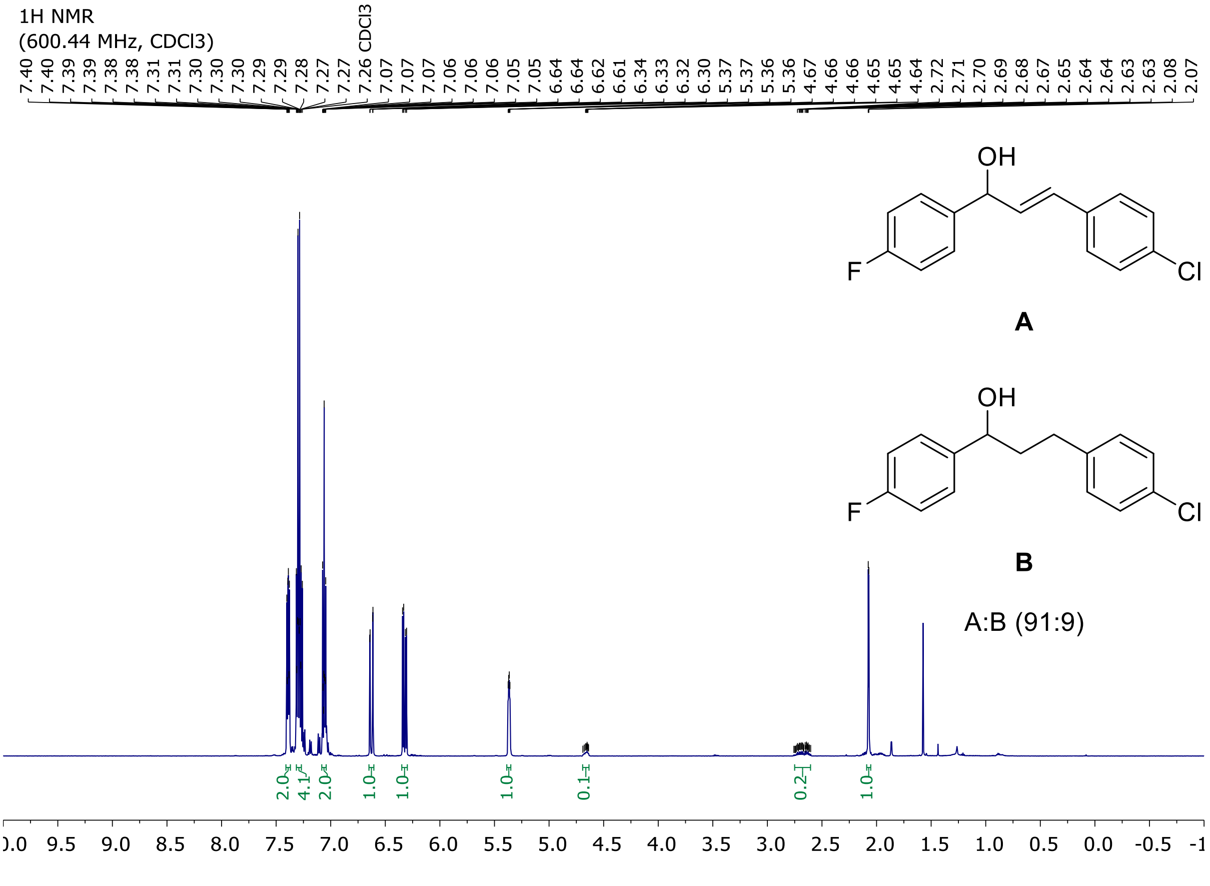

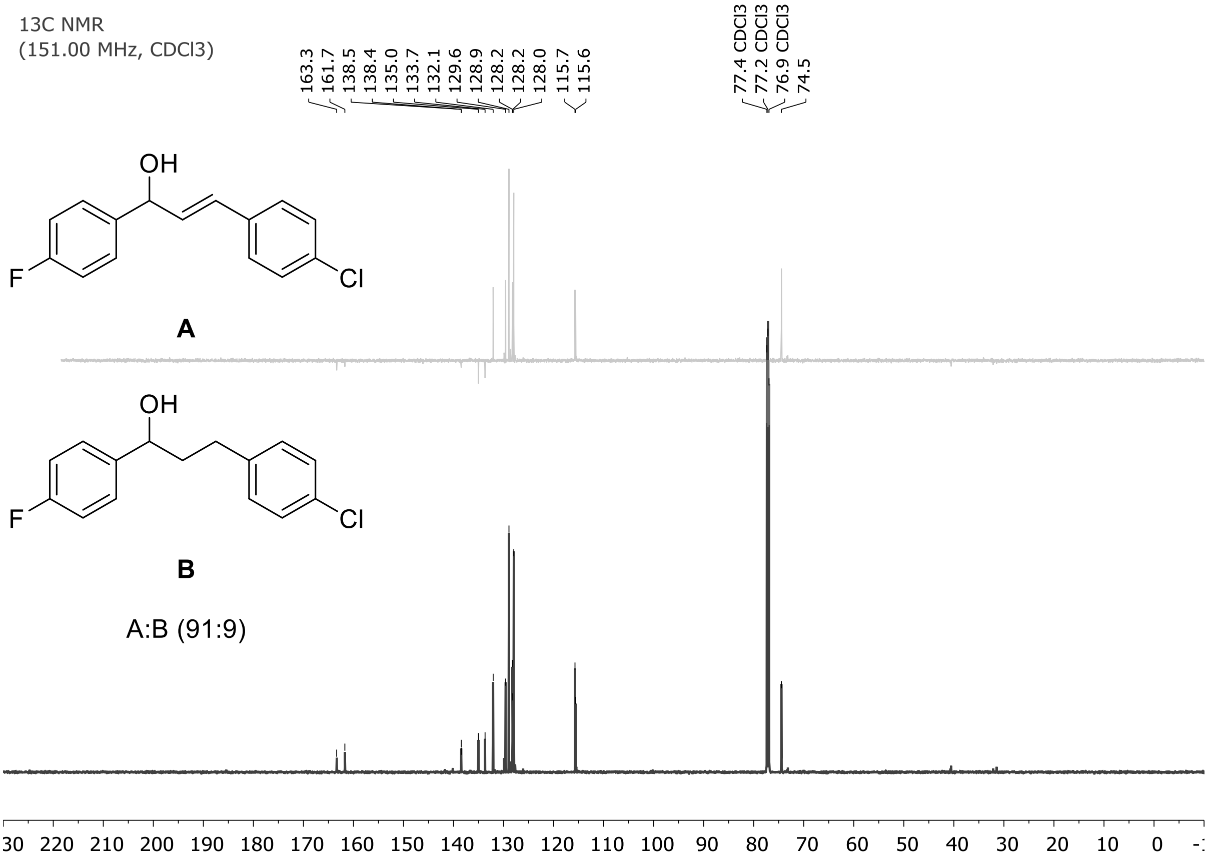
13C NMR
(151.00 MHz, CDCl3)
163.3
161.7
138.5
138.4
135.0
133.7
132.1
129.6
128.9
128.2
128.2
128.0
115.7
115.6
77.4 CDCl3
77.2 CDCl3
76.9 CDCl3
74.5
OH
F
Cl
A
OH
F
Cl
B
A:B (91:9)
30 220 210 200 190 180 170 160 150 140 130 120 110 100 90 80 70 60 50 40 30 20 10 0

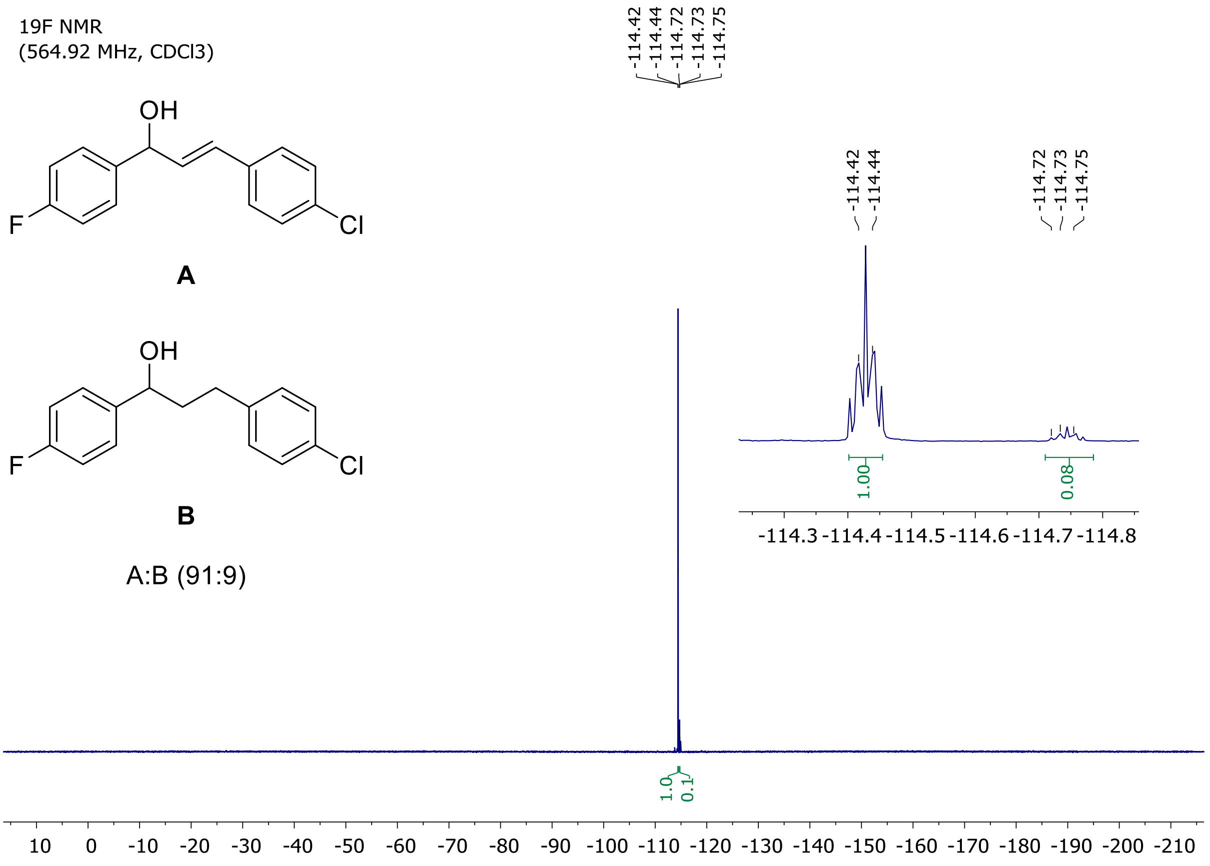
19F NMR
(564.92 MHz, CDCl3)
-114.42
-114.44
-114.72
-114.73
-114.75
OH
F
Cl
A
OH
F
Cl
B
A:B (91:9)
1.00
0.08
-114.3 -114.4 -114.5 -114.6 -114.7 -114.8
1.0
0.1
10 0 -10 -20 -30 -40 -50 -60 -70 -80 -90 -100 -110 -120 -130 -140 -150 -160 -170 -180 -190 -200 -210

## (E)-4-phenylbut-3-en-2-ol (3g)

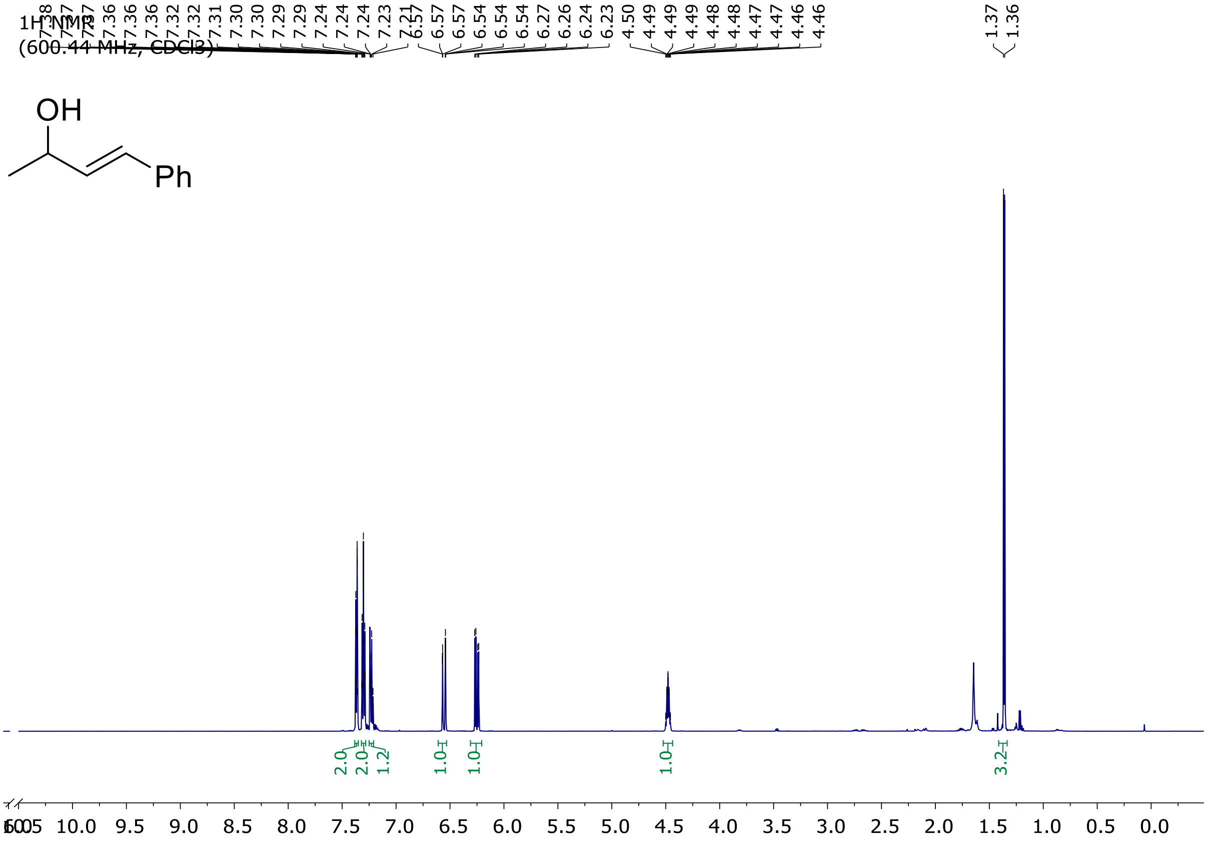


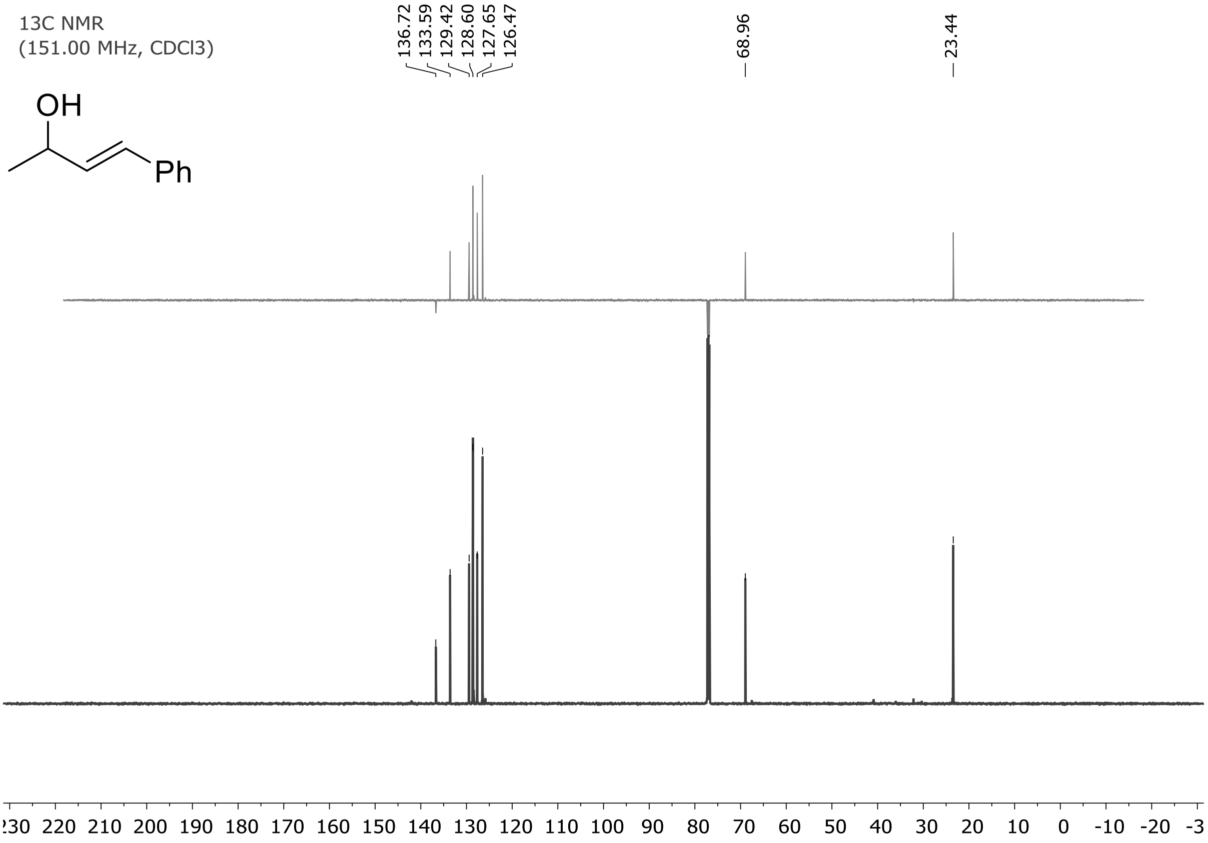

## (E)-non-3-en-2-ol (3h)

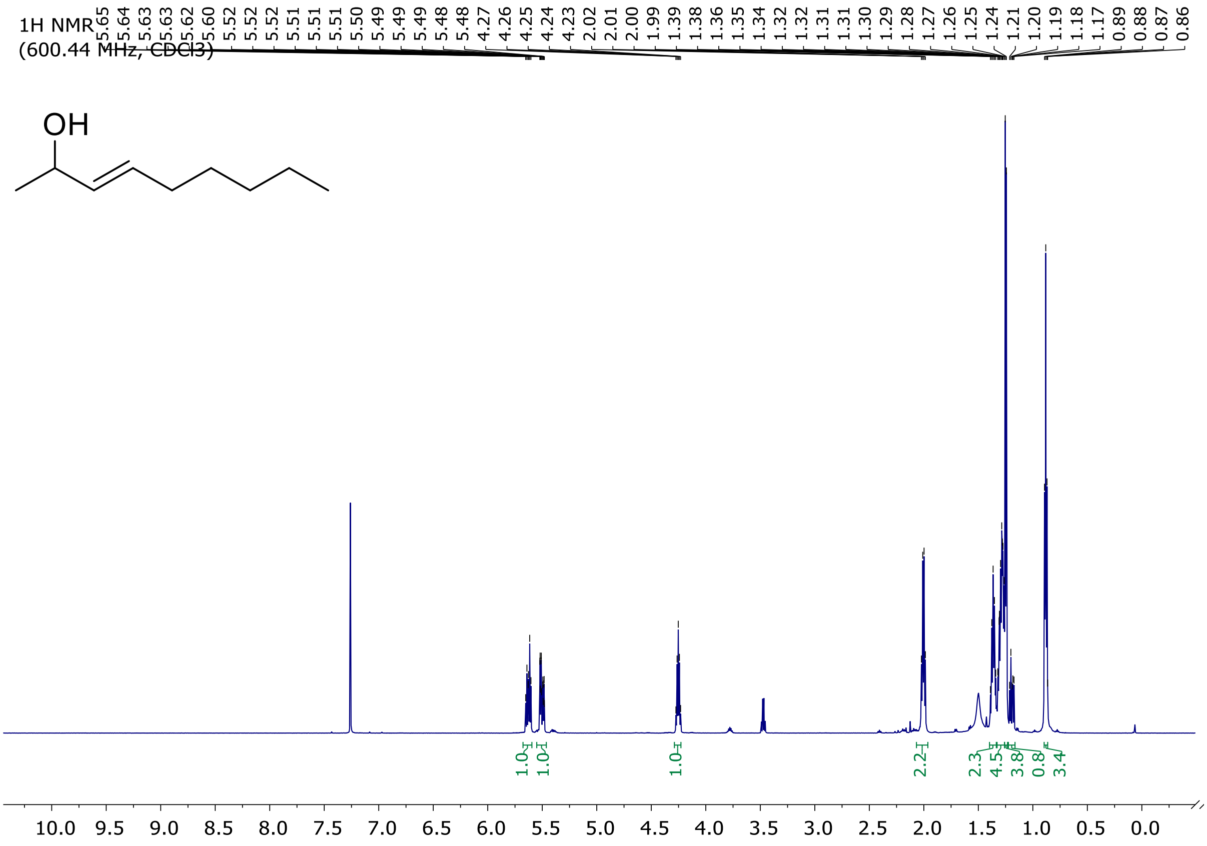


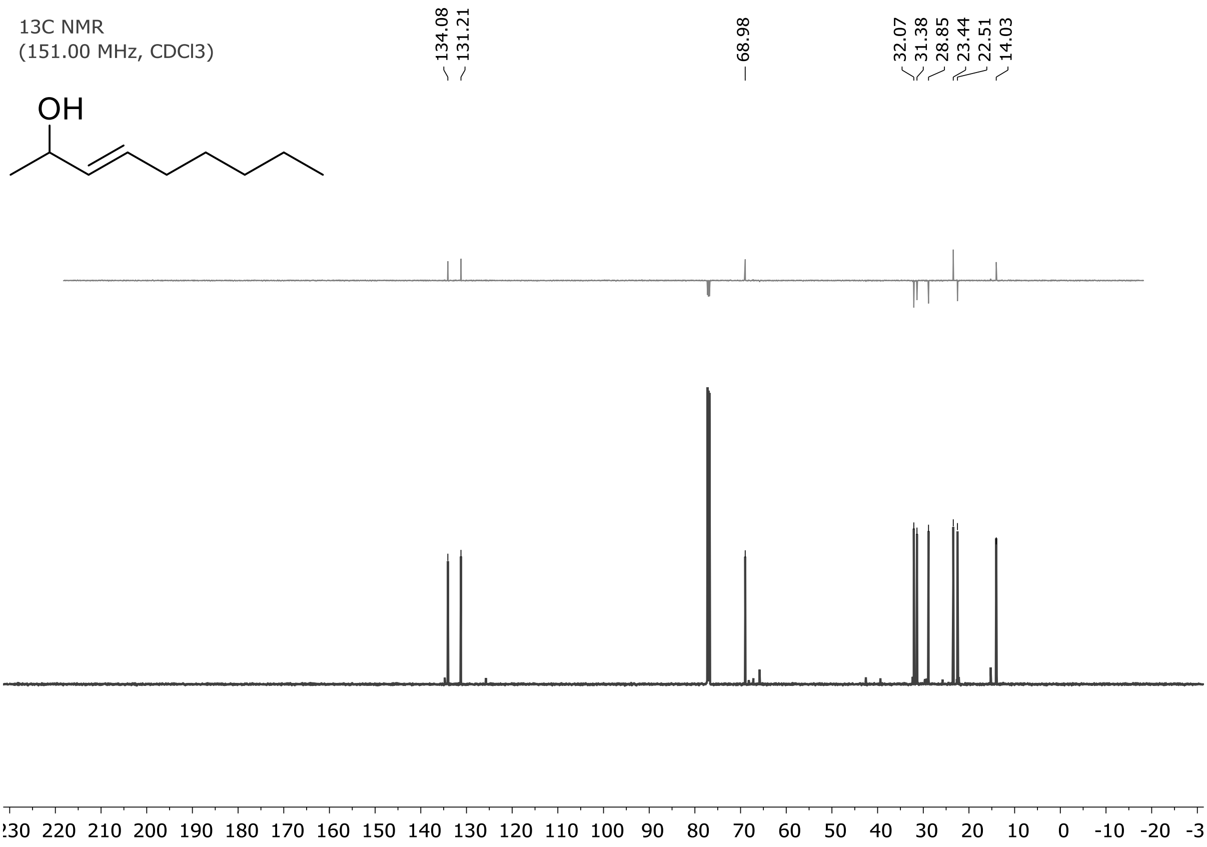

## (E)-4-(2,6,6-trimethylcyclohex-1-en-1-yl)but-3-en-2-ol (3i)

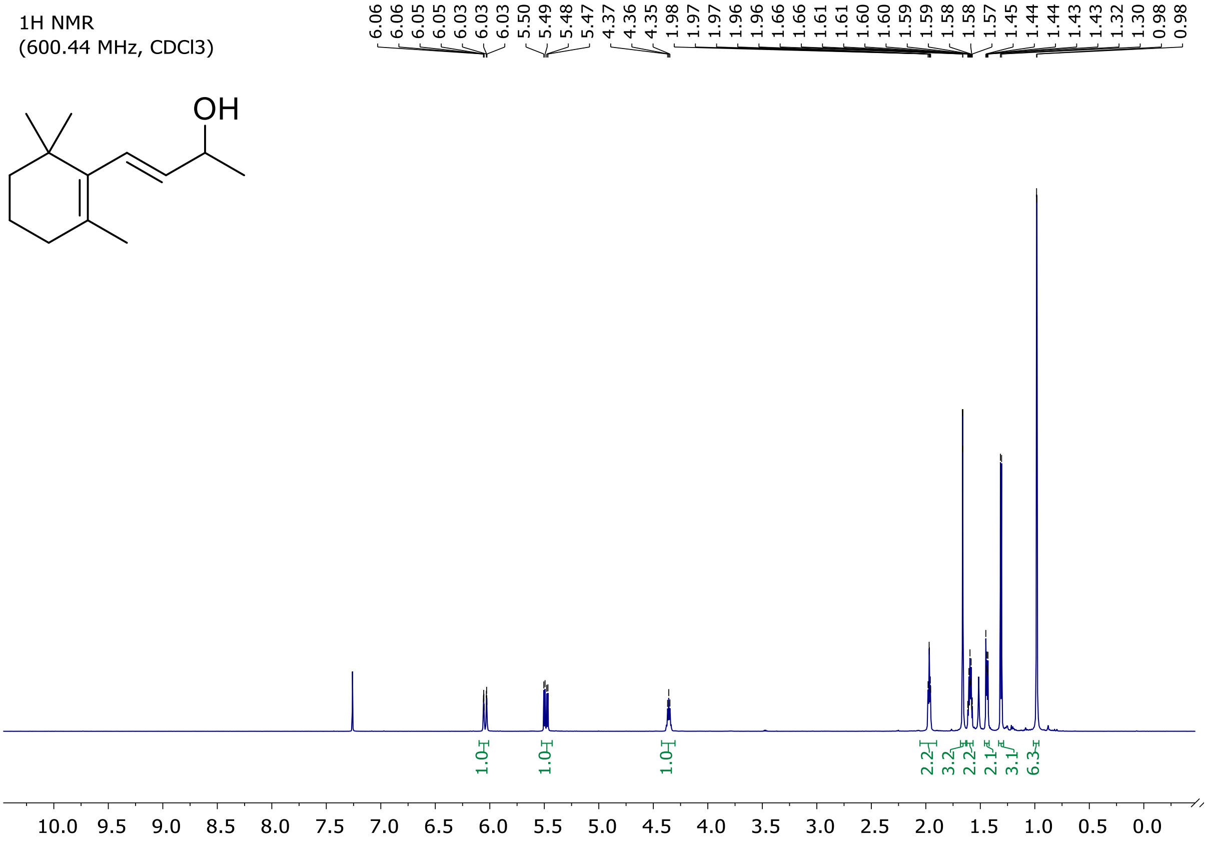


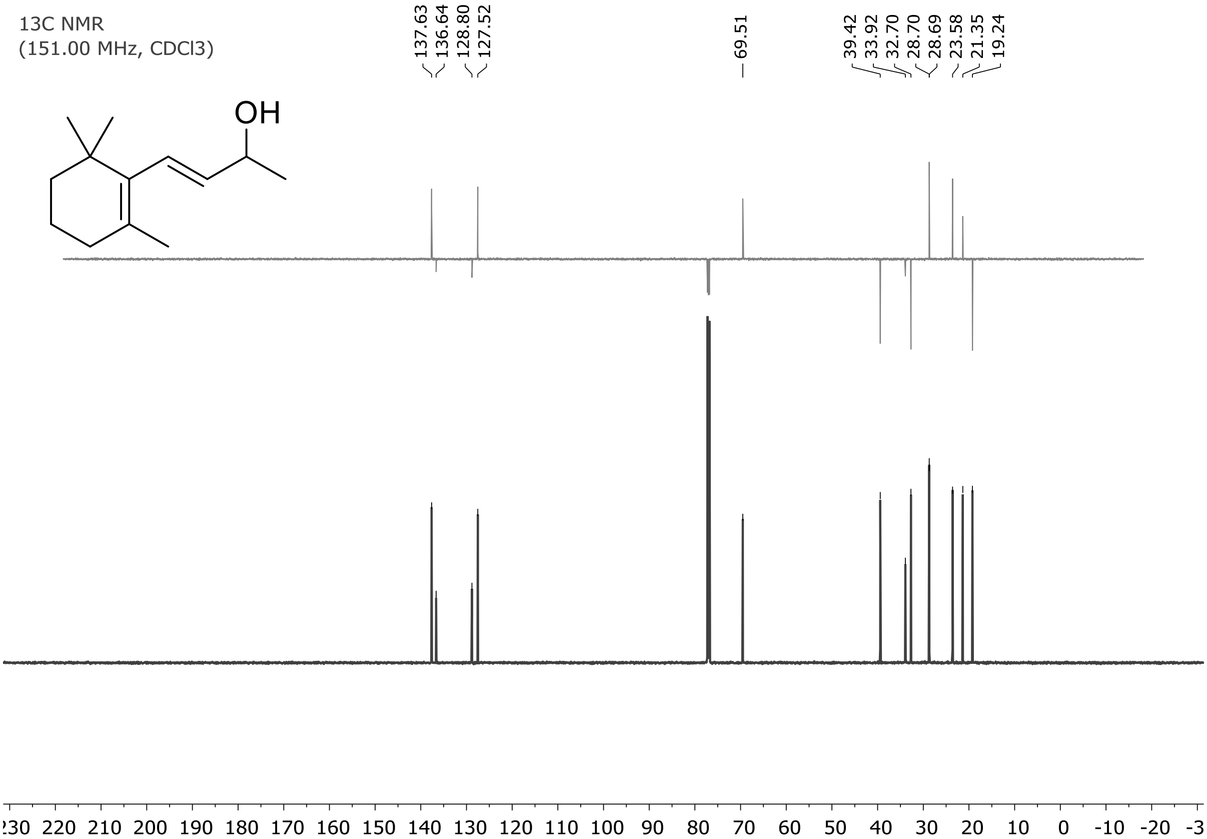

## 1-phenylbut-3-en-1-ol (3j)

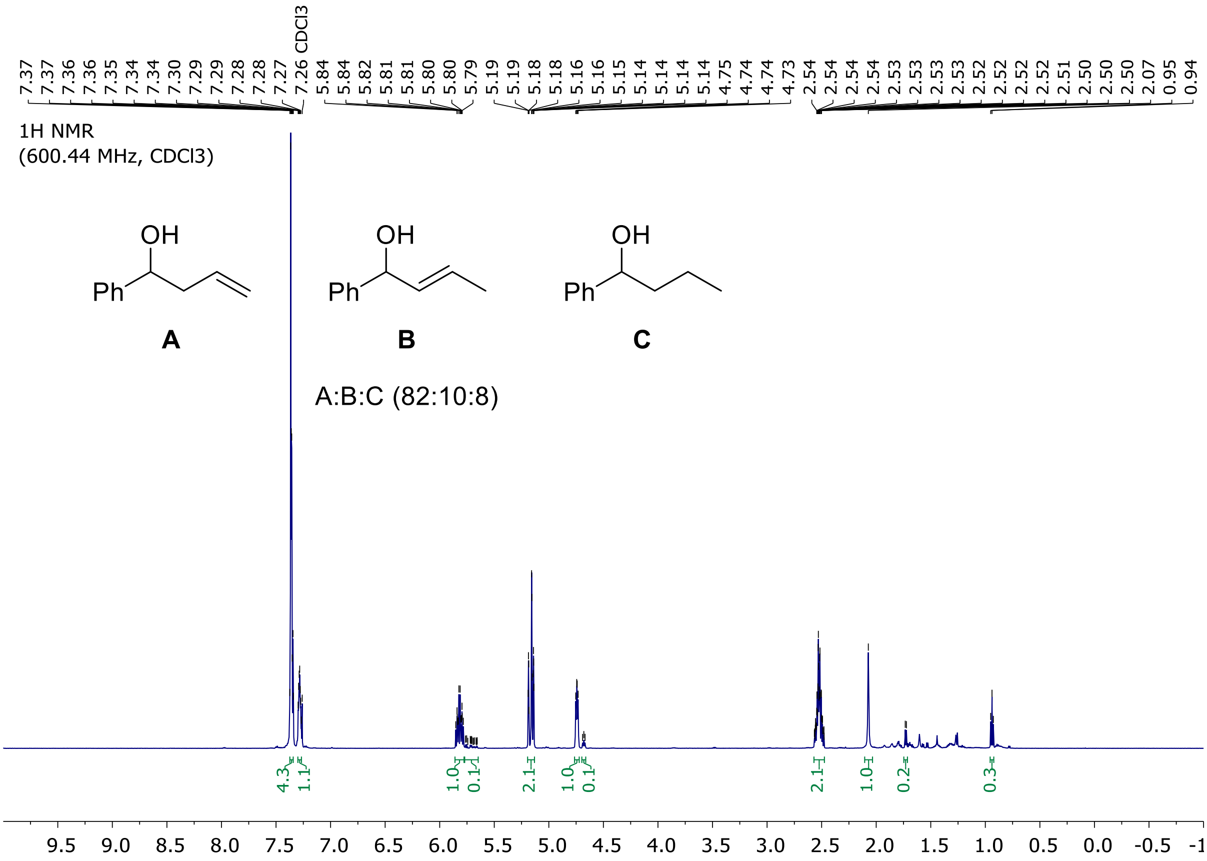


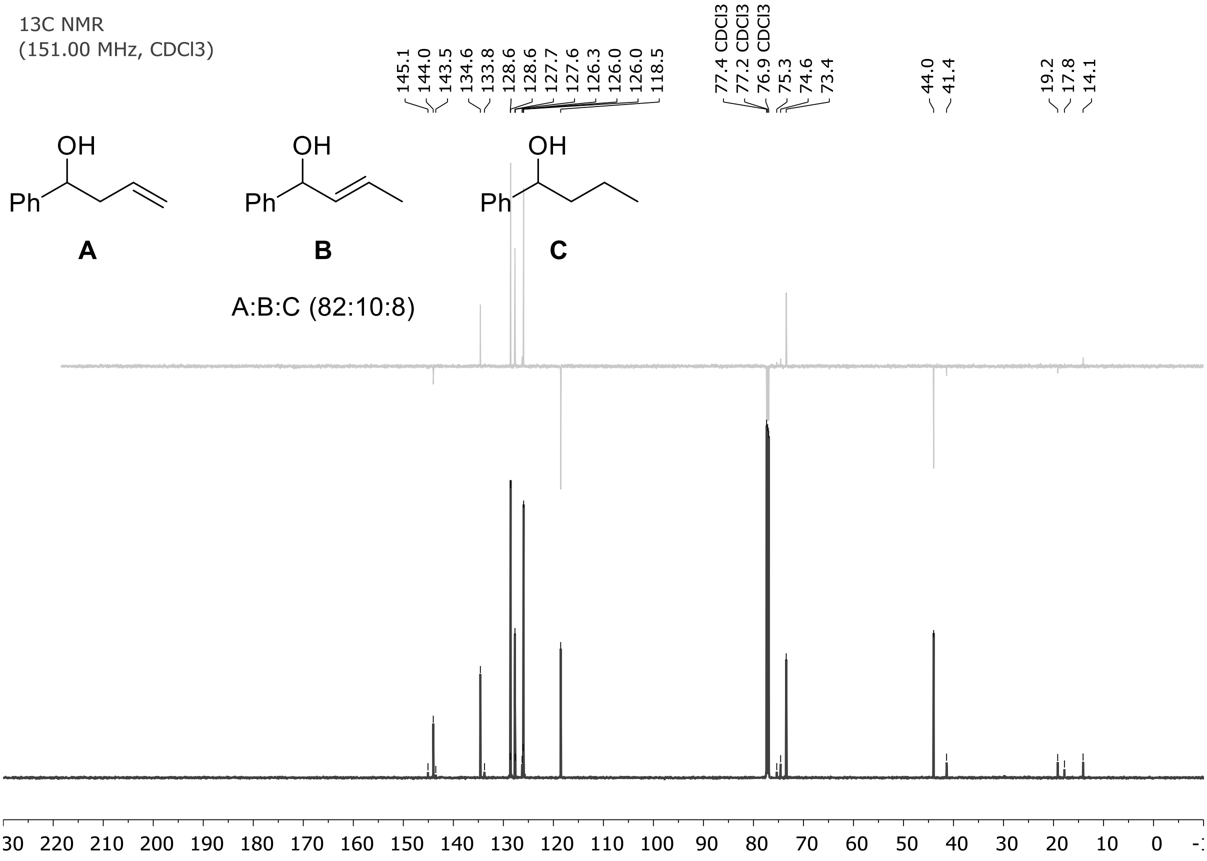

## 4,4-dimethylcyclohex-2-en-1-ol (3k)

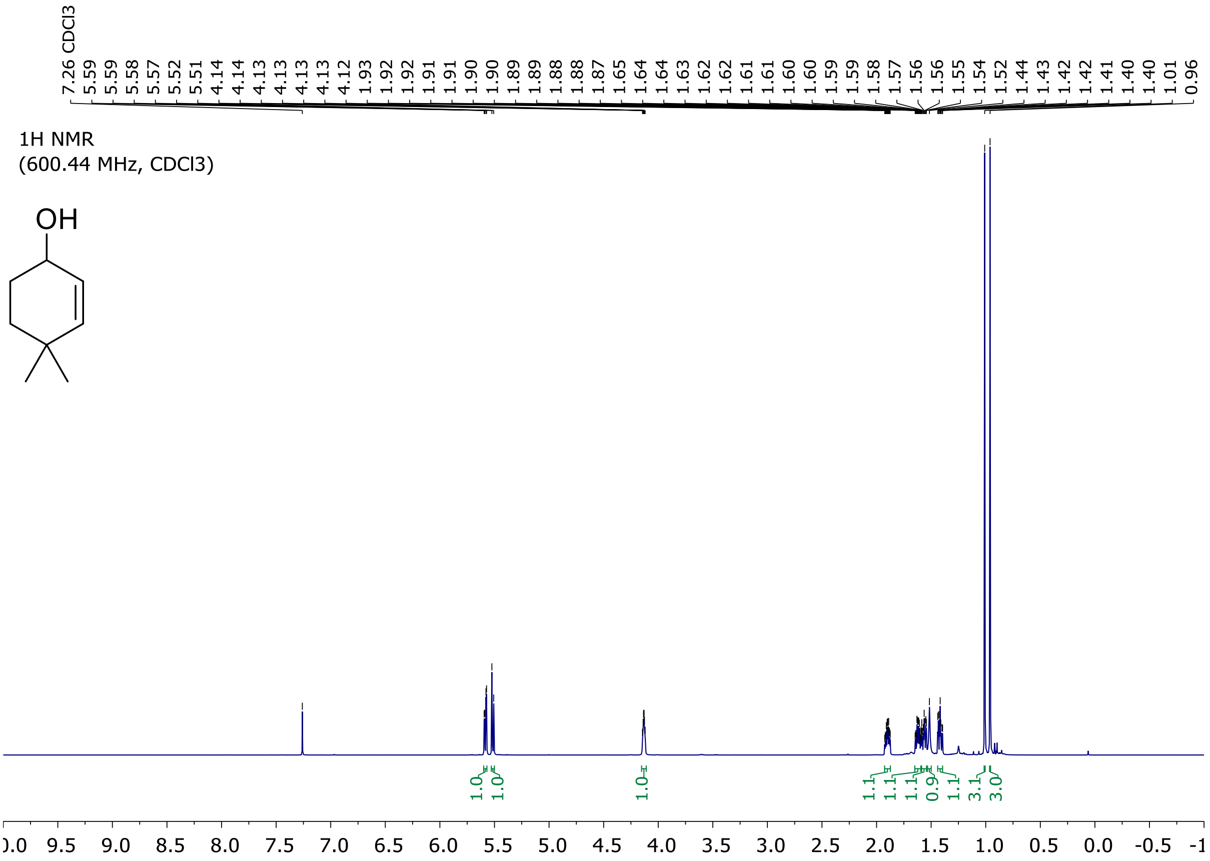


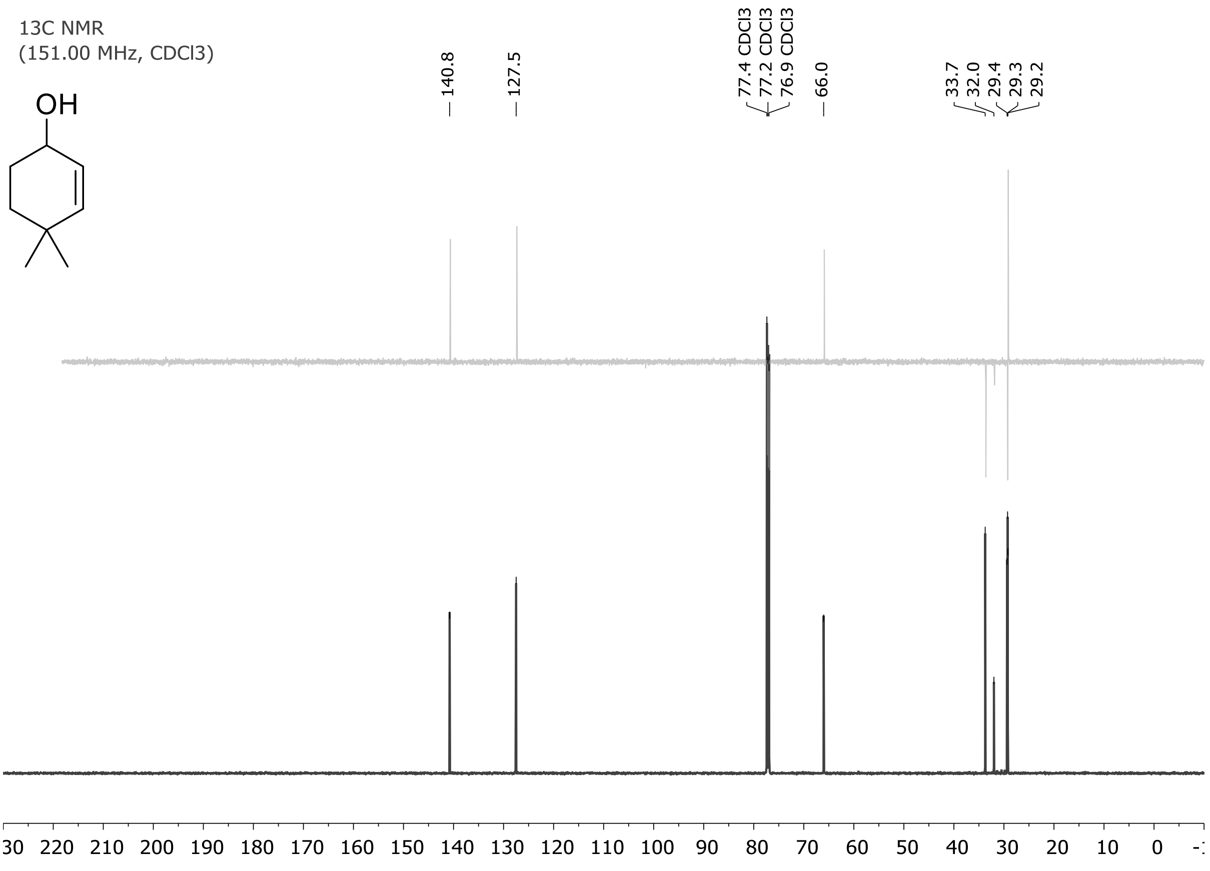


## ethyl 2-((1R,2R)-2-hydroxycyclohex-3-en-1-yl)acetate (3l)

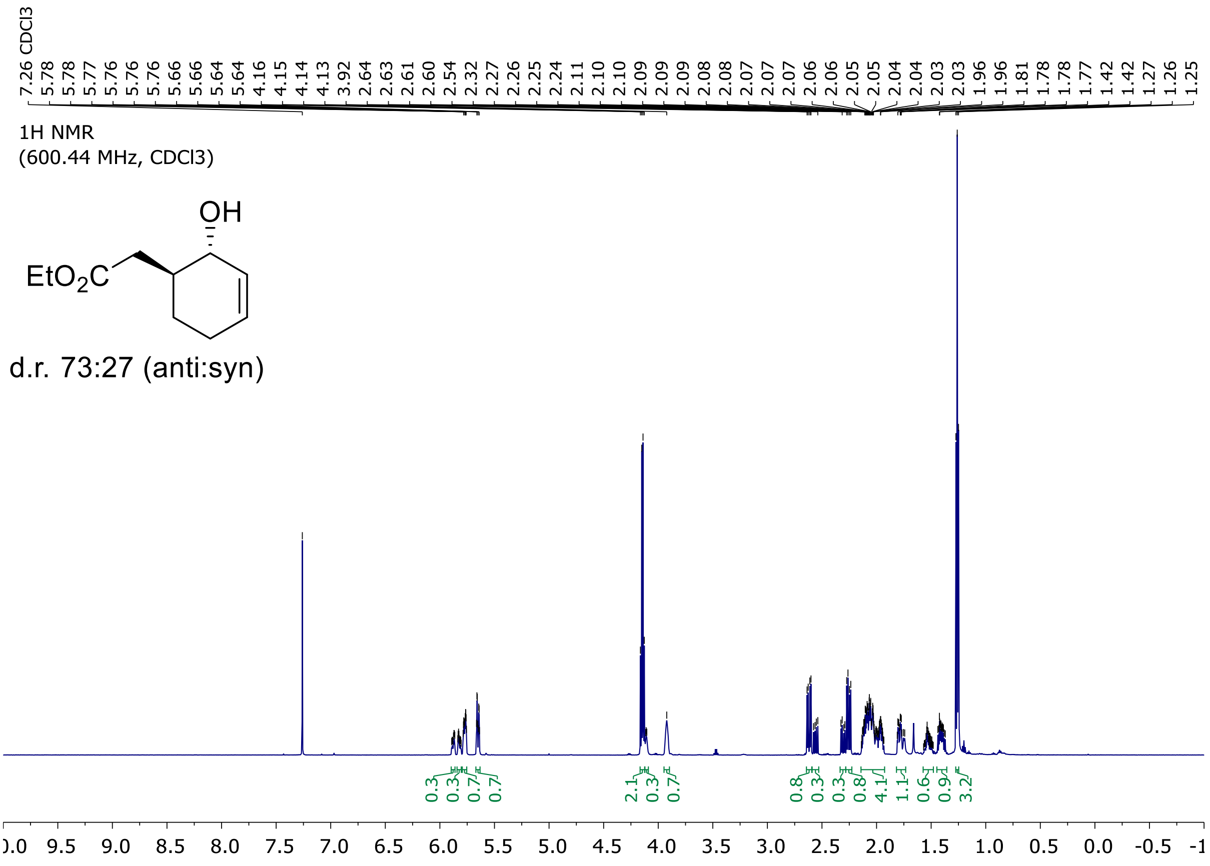


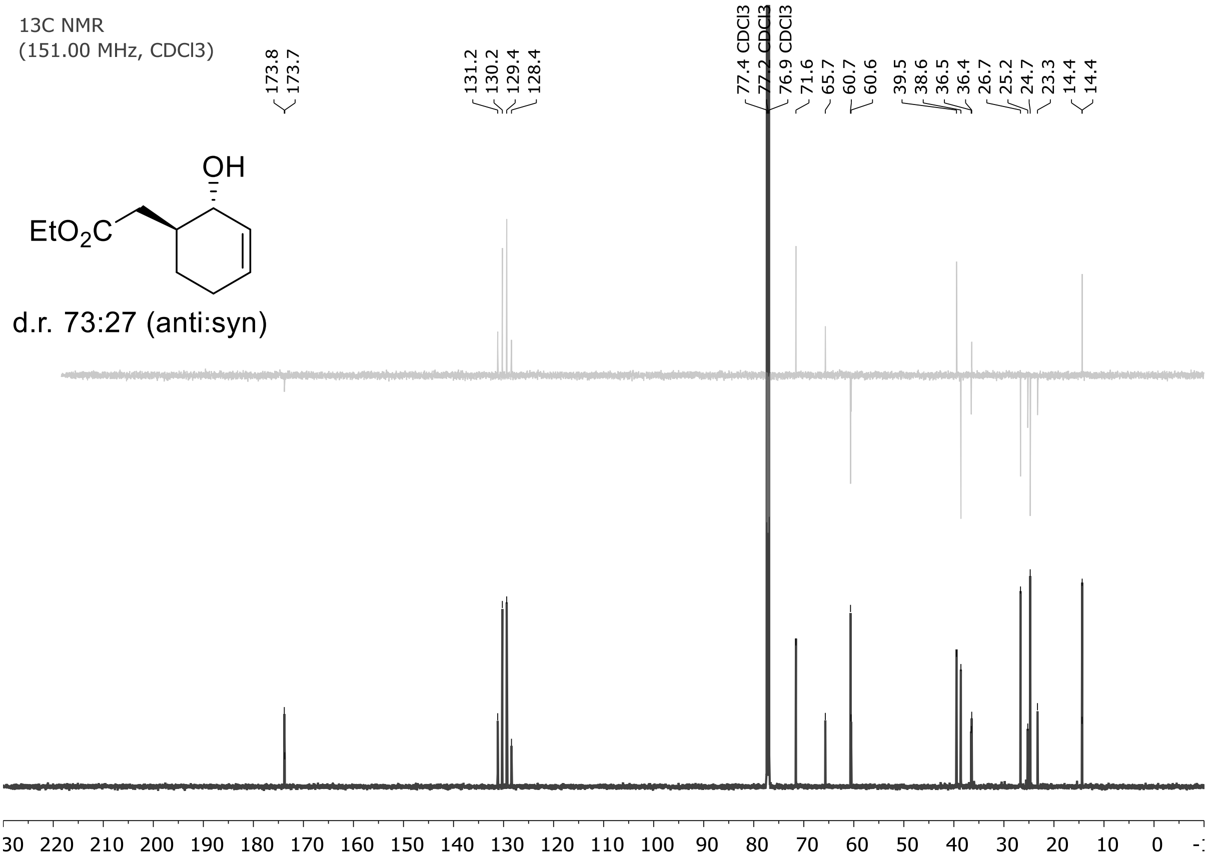


**(1S,4S)-1,2,3,4-tetrahydro-[1,1'-biphenyl]-4-ol (3m)**

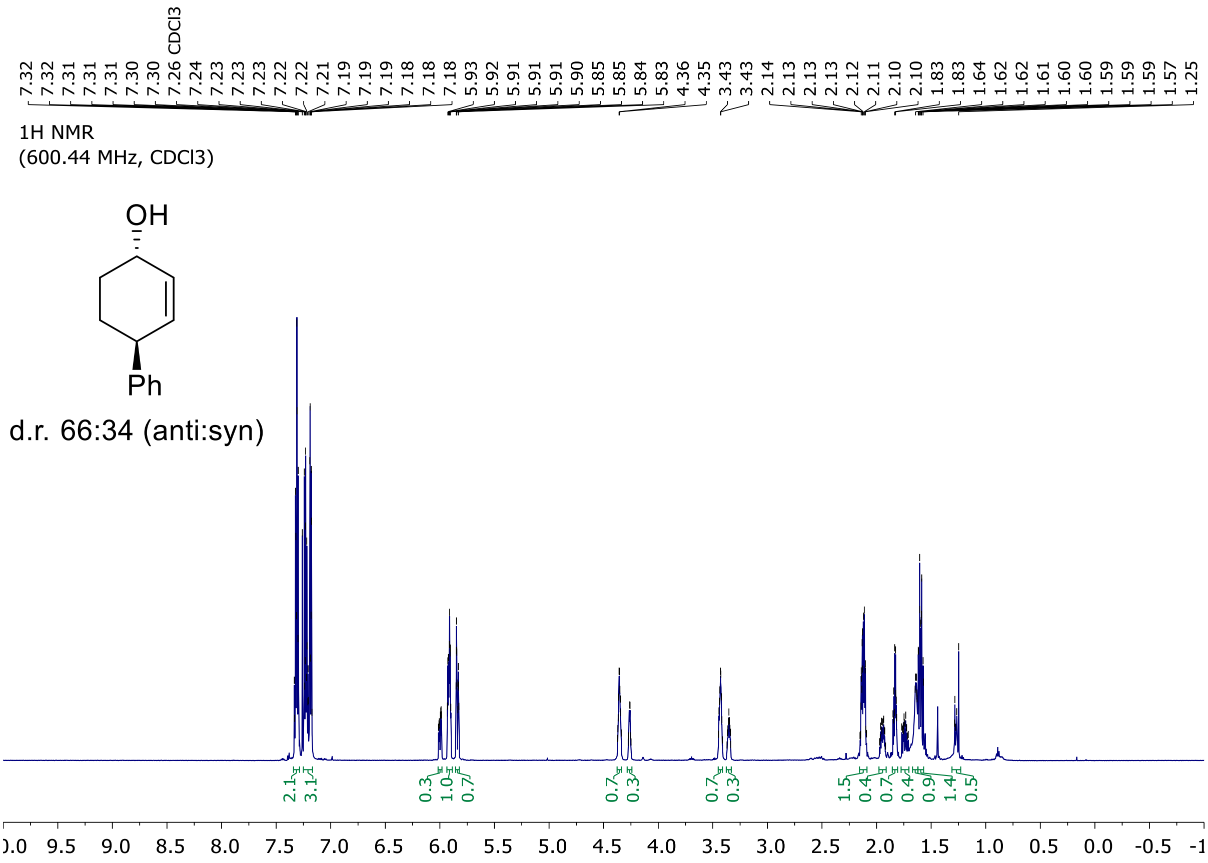


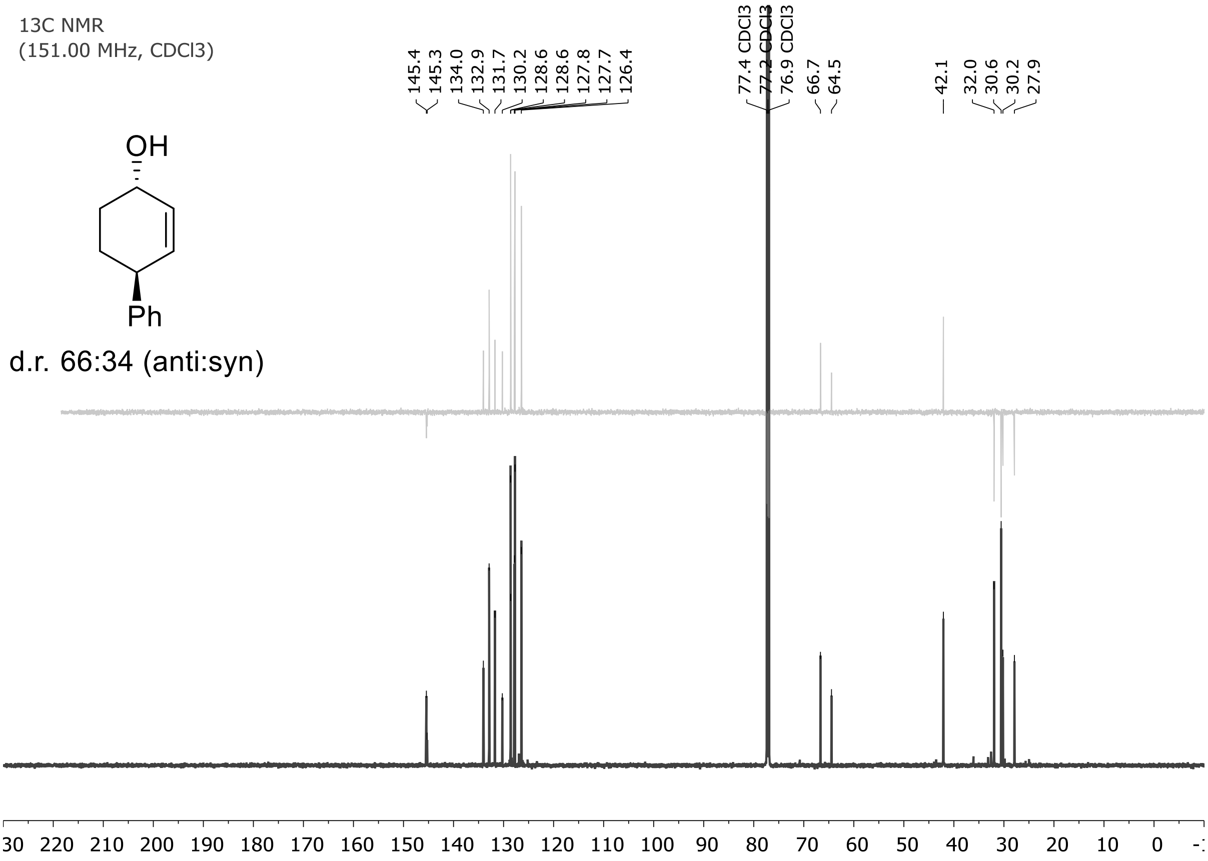


**(1R,4R)-4-(tert-butyl)cyclohex-2-en-1-ol (3n)**

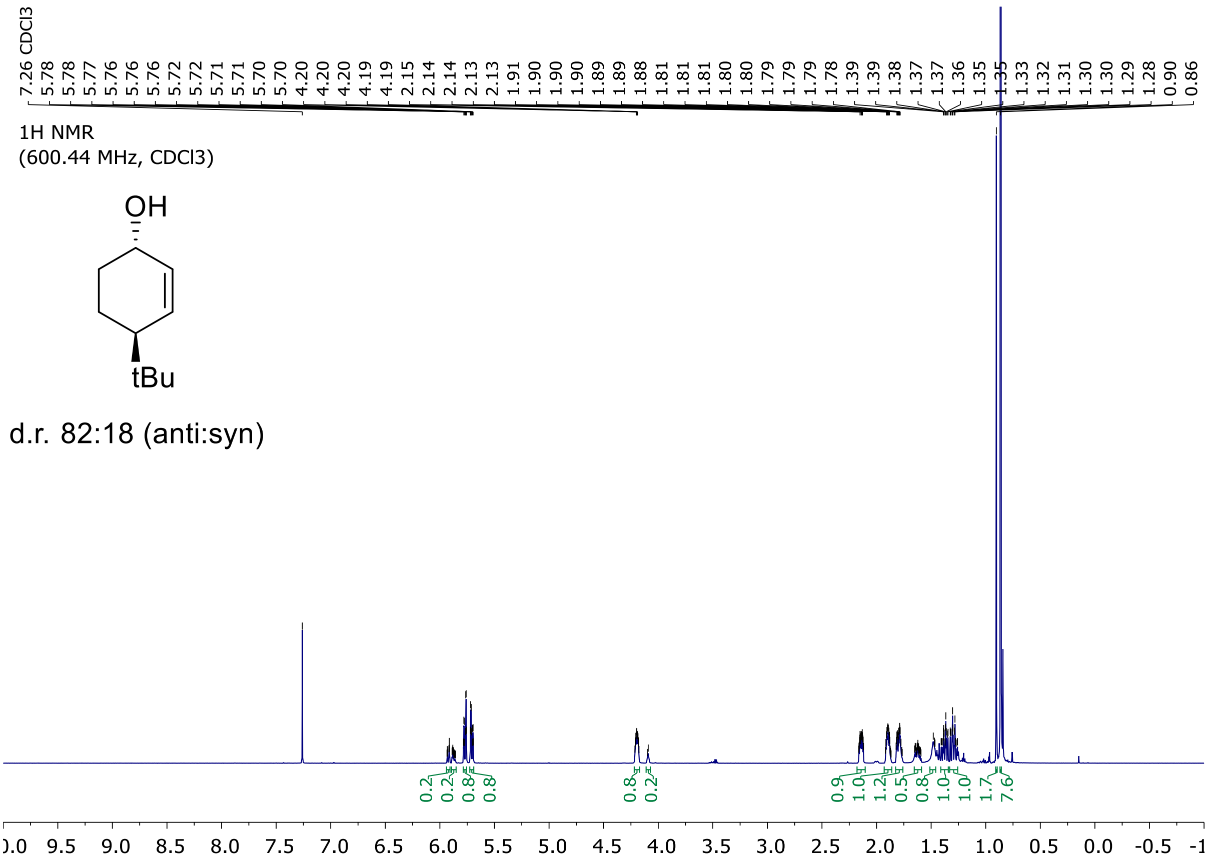
7.26 CDCl3
5.78 5.78 5.77 5.76 5.76 5.72 5.72 5.71 5.71 5.70 5.70 4.20 4.20 4.20 4.19 4.19 2.15 2.14 2.14 2.13 2.13 1.91 1.90 1.90 1.90 1.89 1.89 1.88 1.81 1.81 1.81 1.80 1.80 1.79 1.79 1.79 1.78 1.39 1.39 1.38 1.37 1.37 1.36 1.35 1.35 1.33 1.32 1.31 1.30 1.30 1.29 1.28 0.90 0.86
1H NMR
(600.44 MHz, CDCl3)
OH
tBu
d.r. 82:18 (anti:syn)
0.2 0.2 0.8 0.8 0.8 0.2 0.9 1.0 1.2 0.5 0.8 1.0 1.0 1.7 7.6

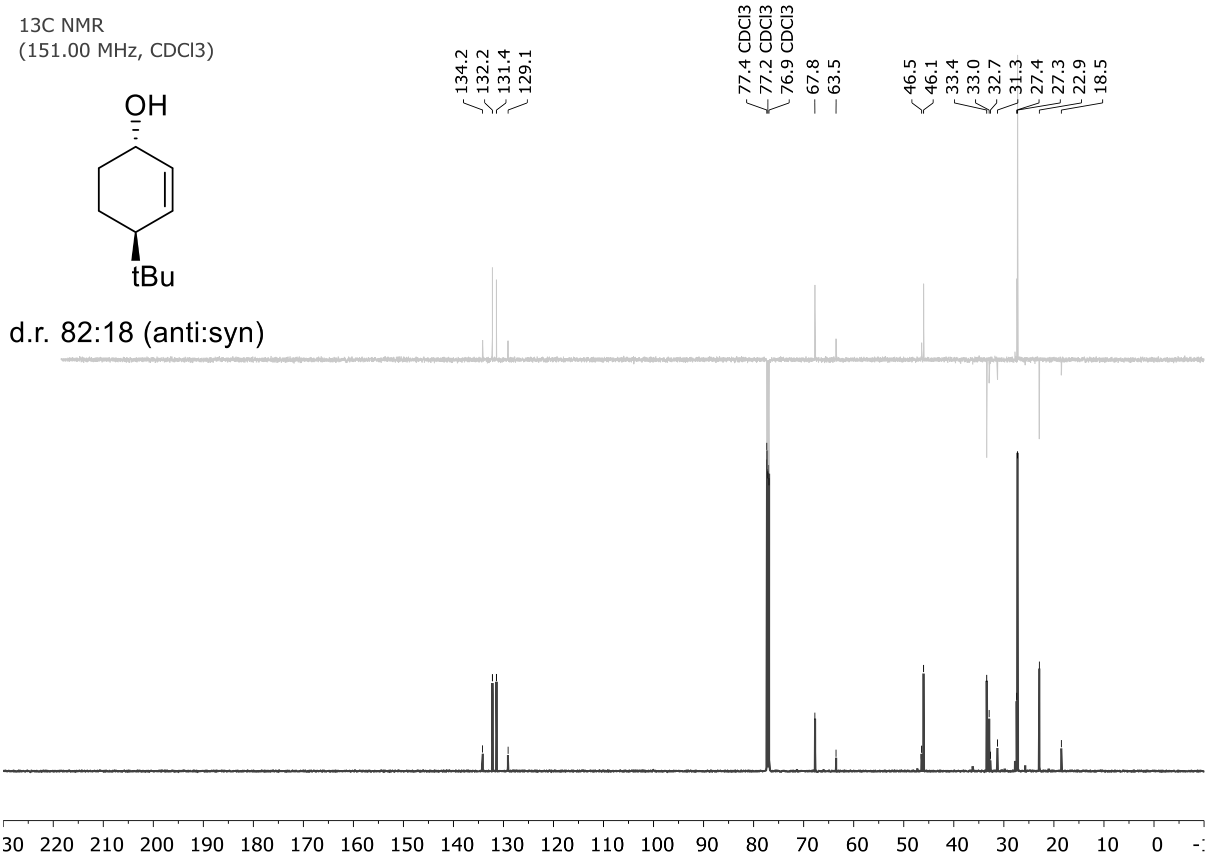
13C NMR
(151.00 MHz, CDCl3)
134.2 132.2 131.4 129.1
77.4 CDCl3
77.2 CDCl3
76.9 CDCl3
67.8 63.5
46.5 46.1 33.4 33.0 32.7 31.3 27.4 27.3 22.9 18.5
OH
tBu
d.r. 82:18 (anti:syn)

**(1R,5R)-2-methyl-5-(prop-1-en-2-yl)cyclohex-2-en-1-ol (3o)**

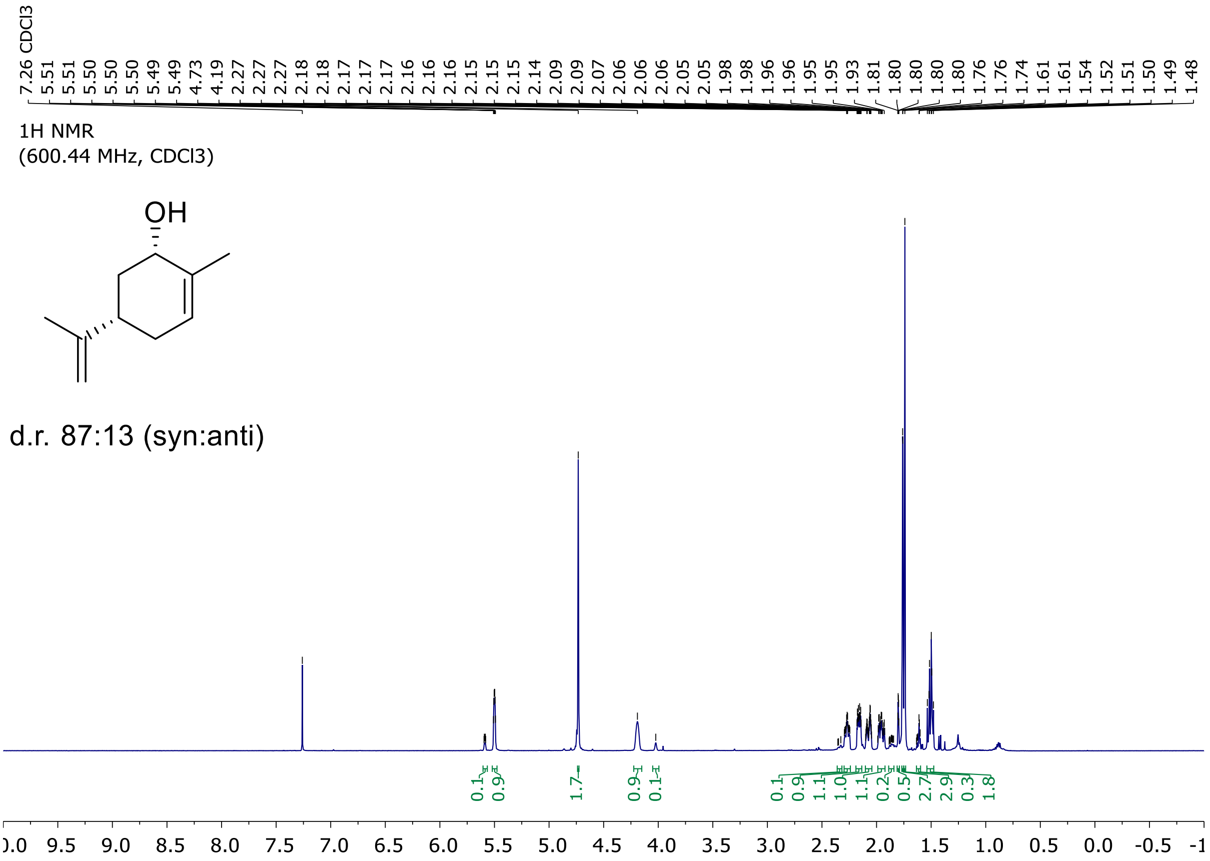


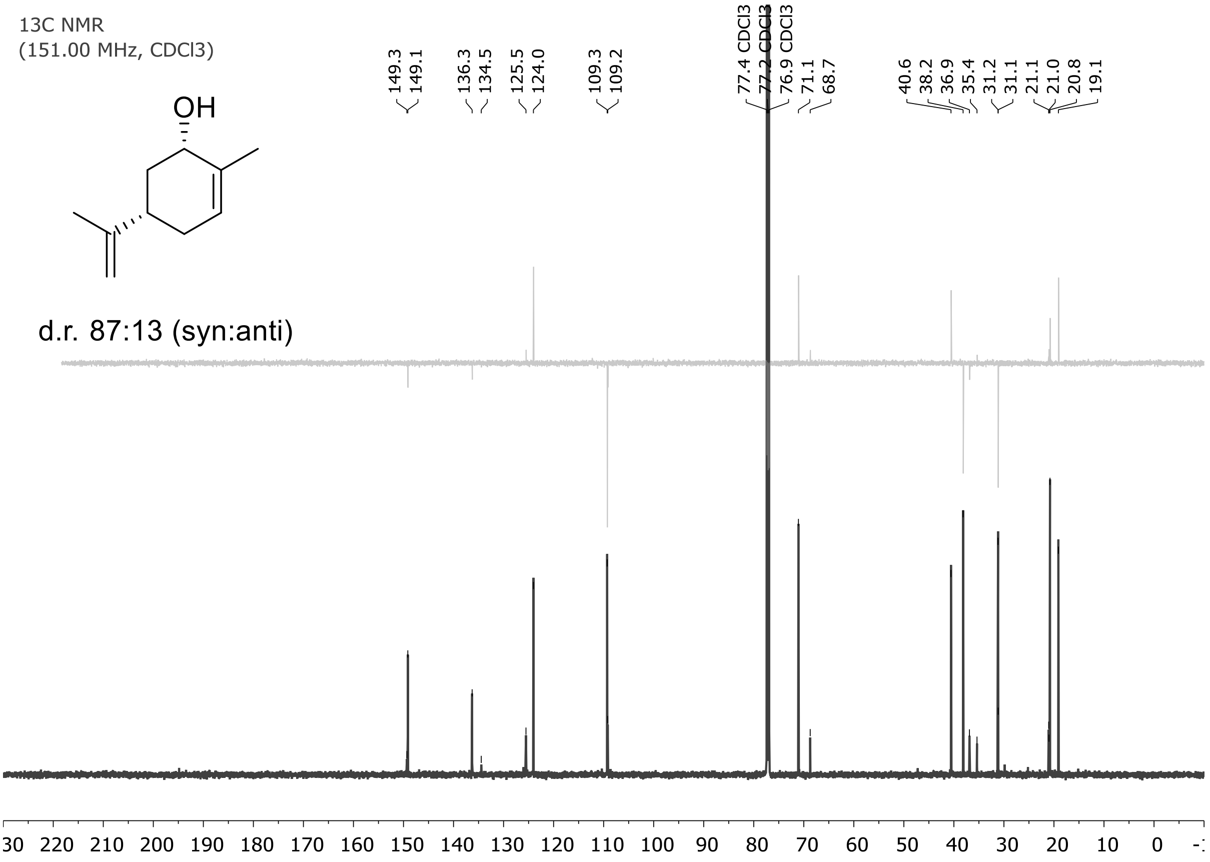

## (E)-3-phenylprop-2-en-1-ol (3p)

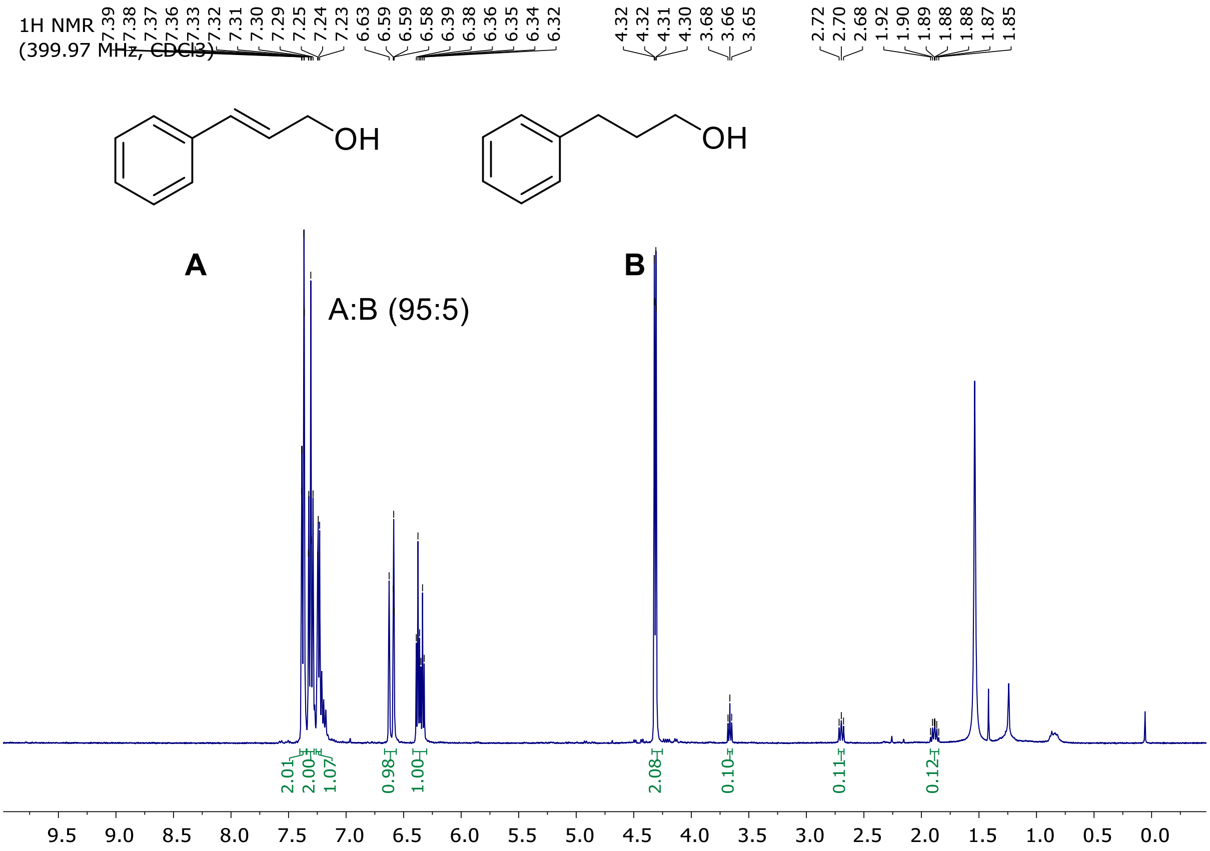


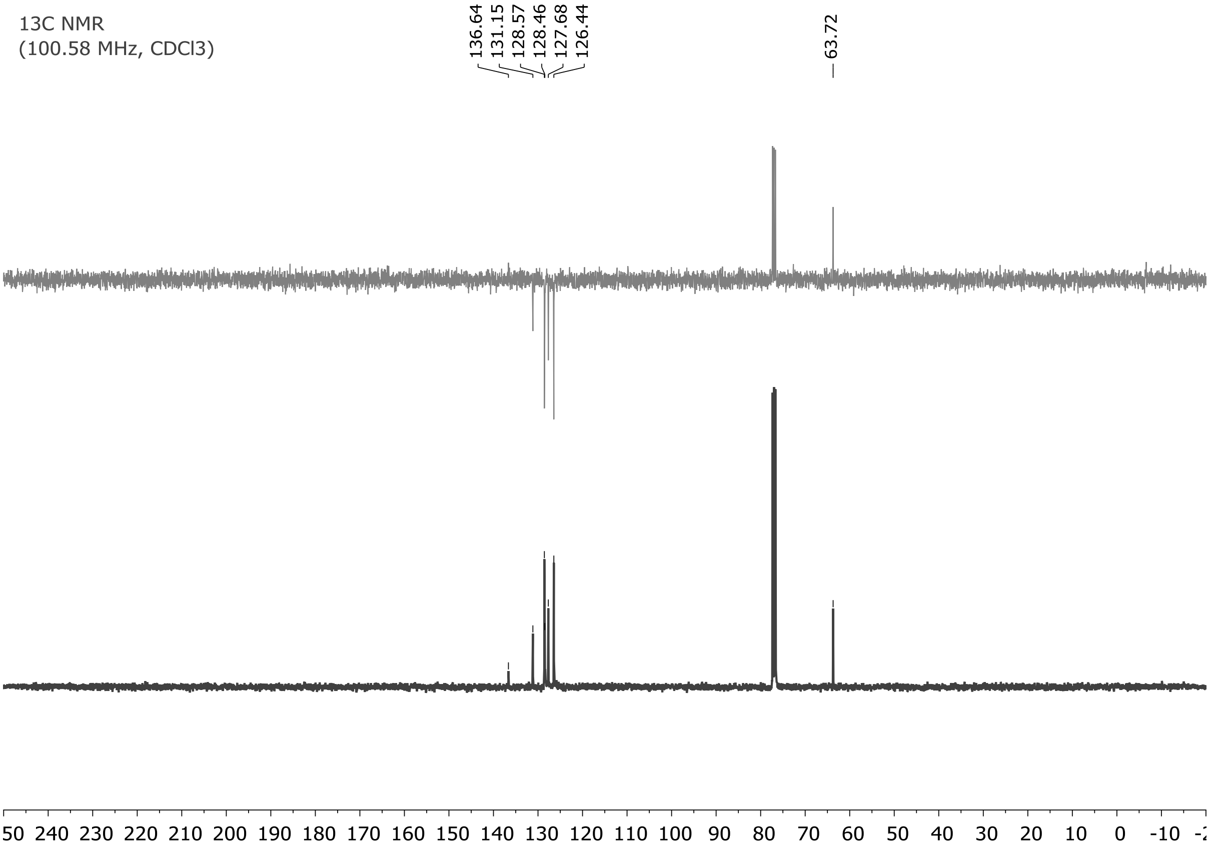

**2-(hydroxy(phenyl)methyl)-4,4-dimethylcyclohexan-1-one (4a)**

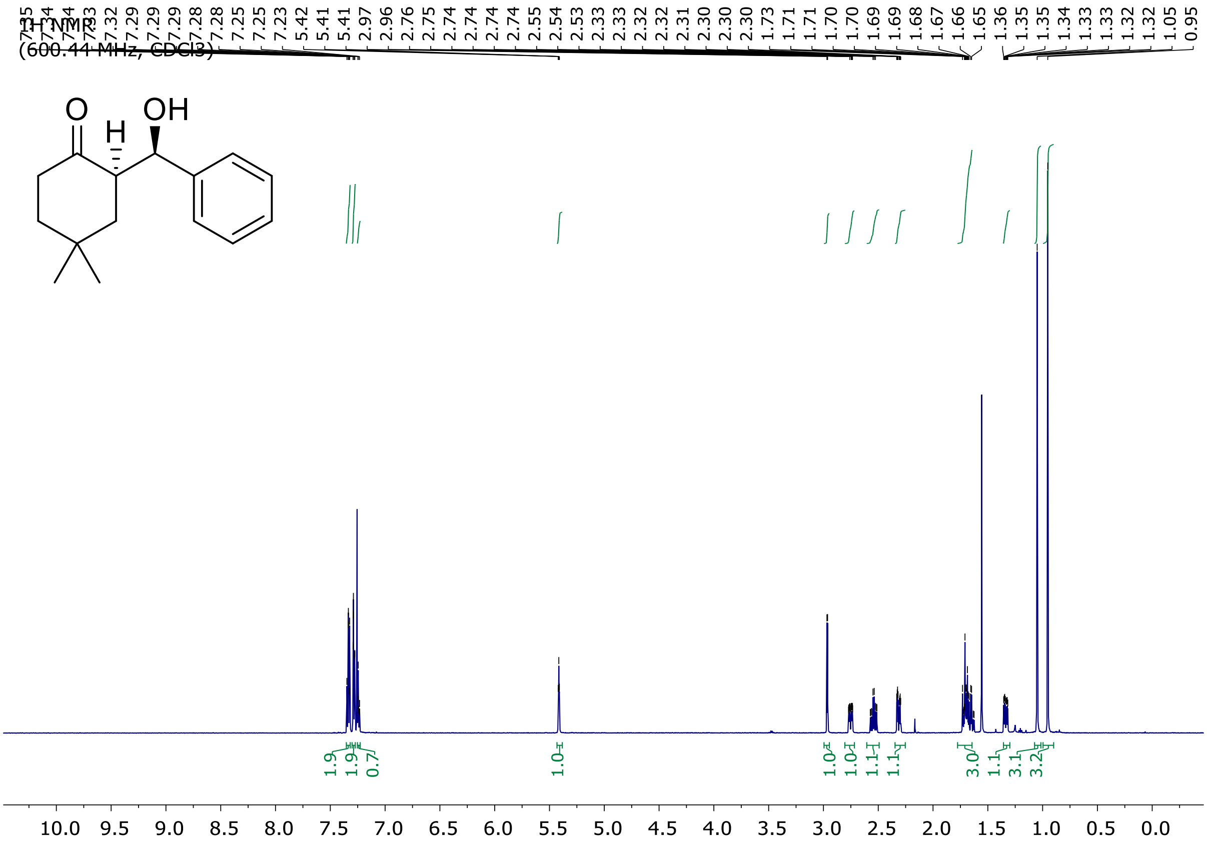


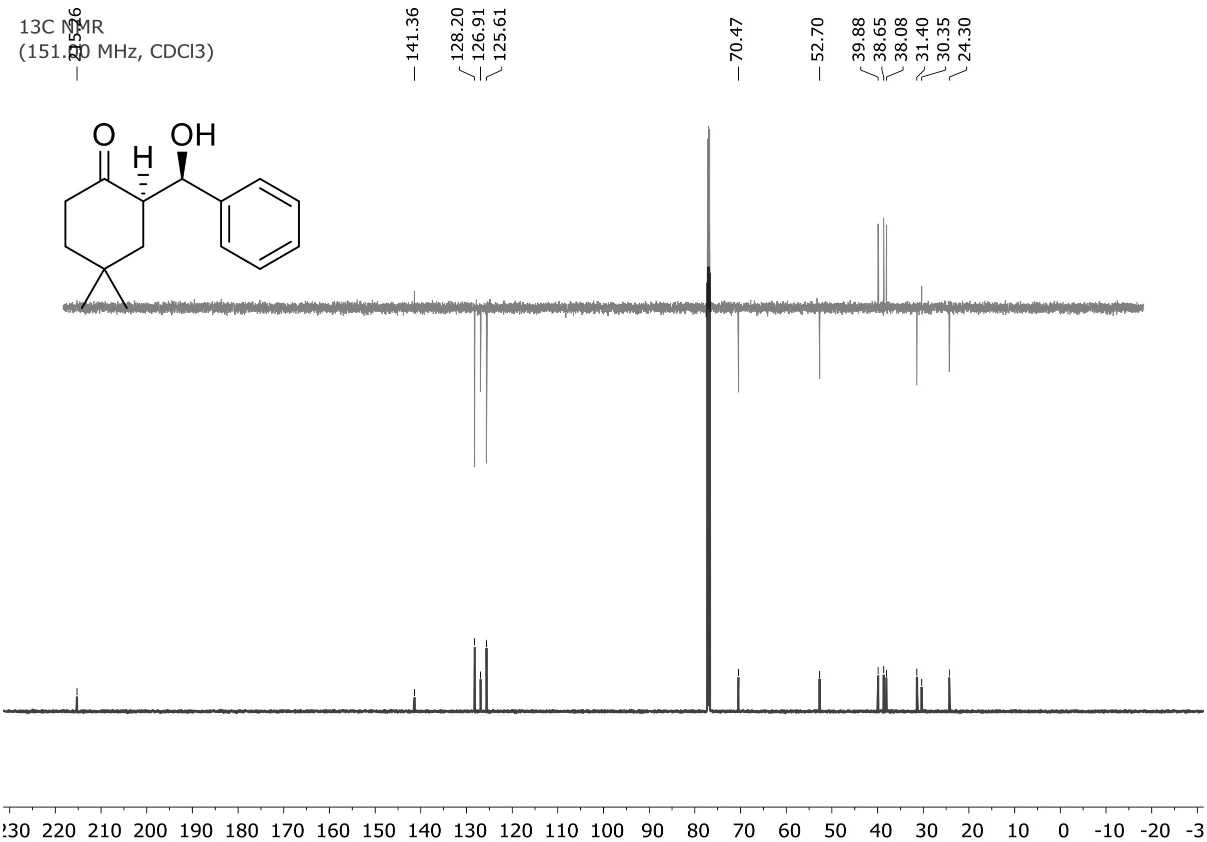

## 2-(hydroxy(phenyl)methyl)cyclohexan-1-one (4b)

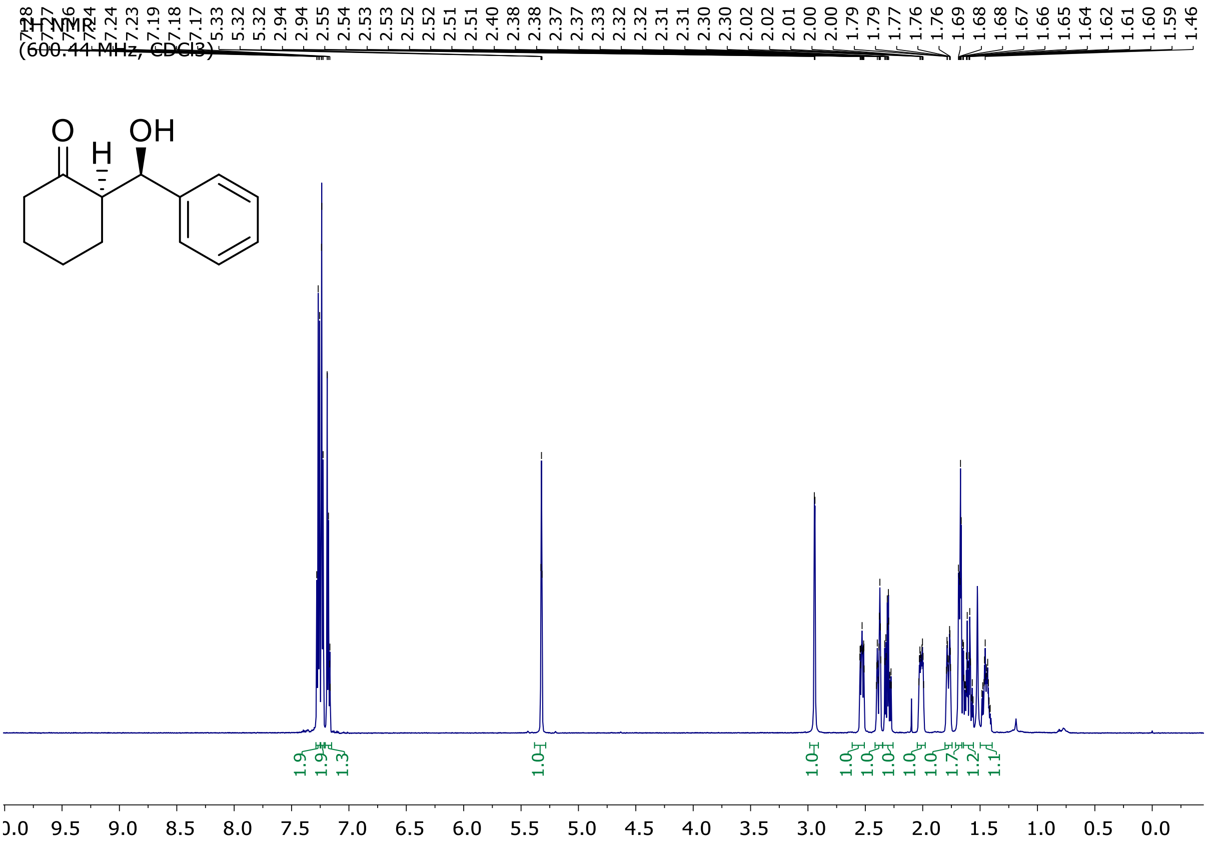


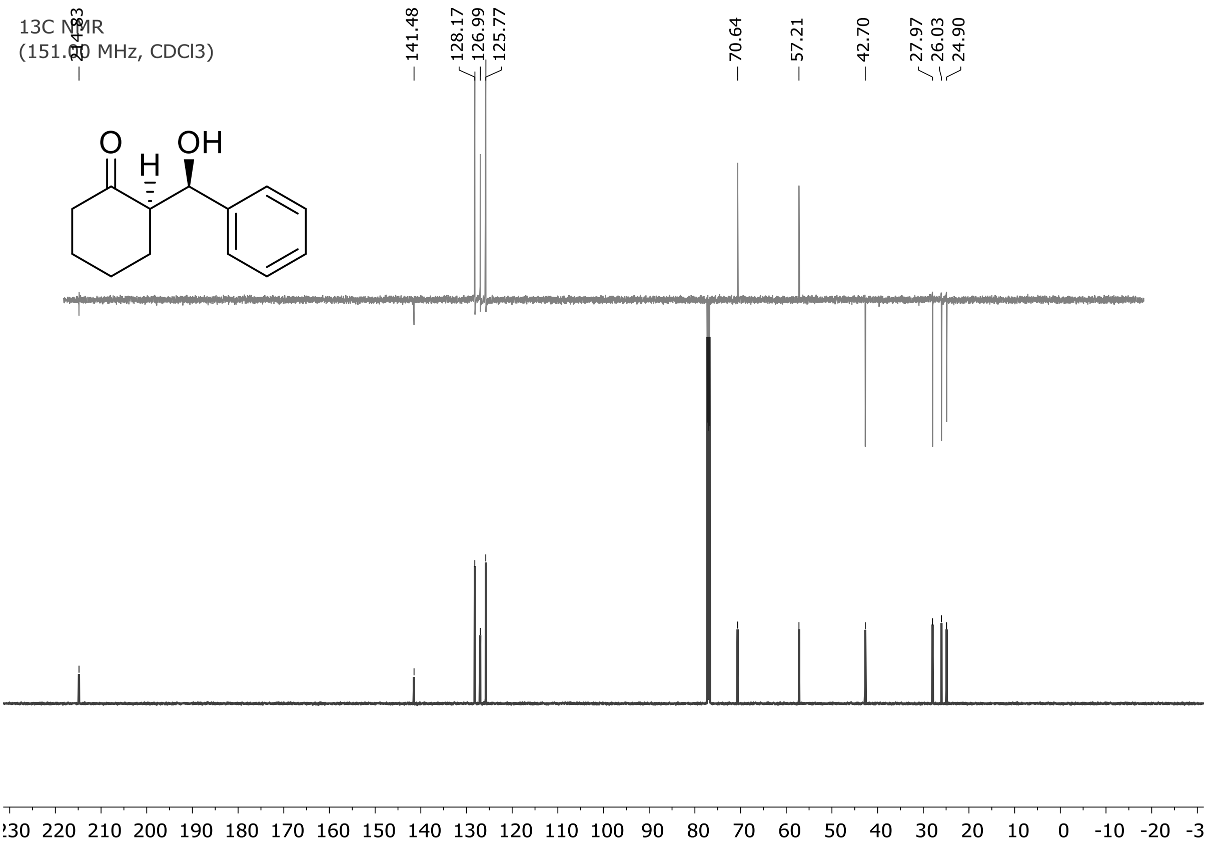

**2-(hydroxy(phenyl)methyl)cyclopentan-1-one (4c)**

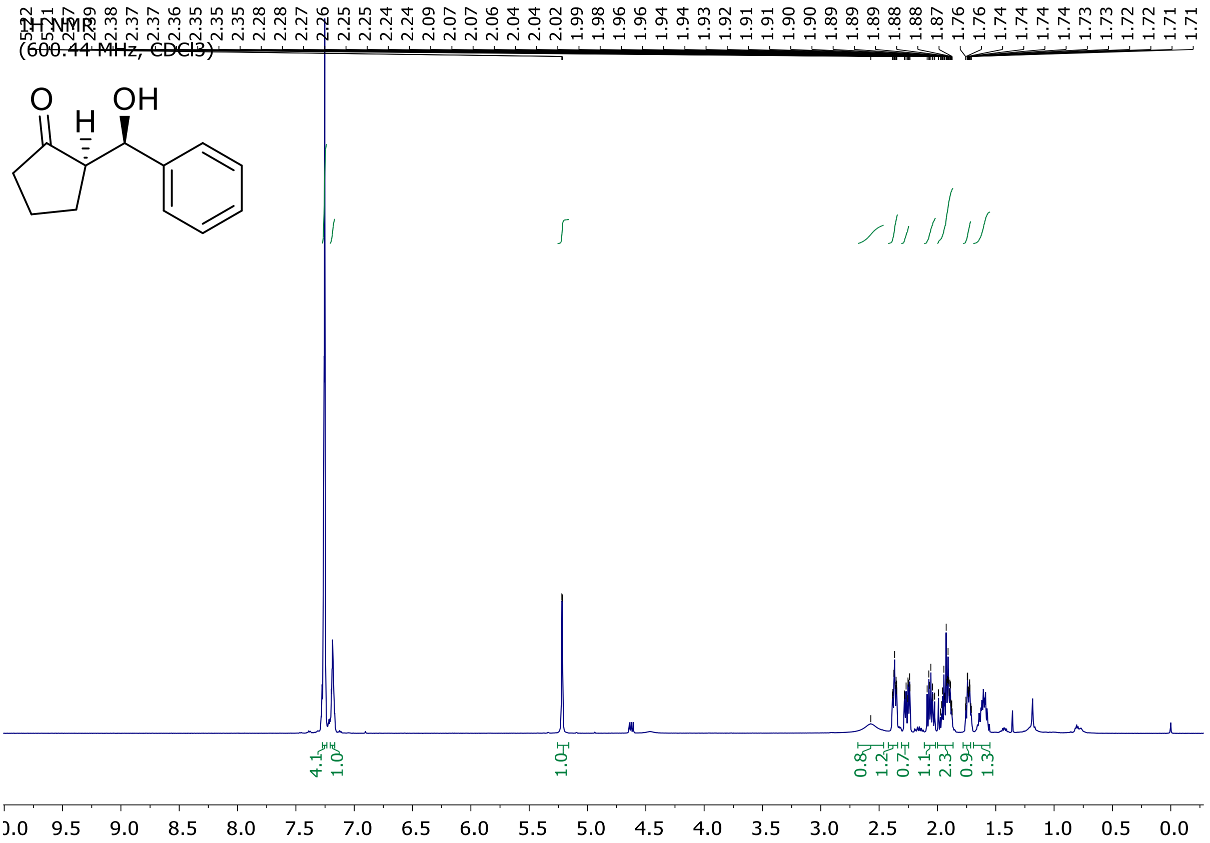


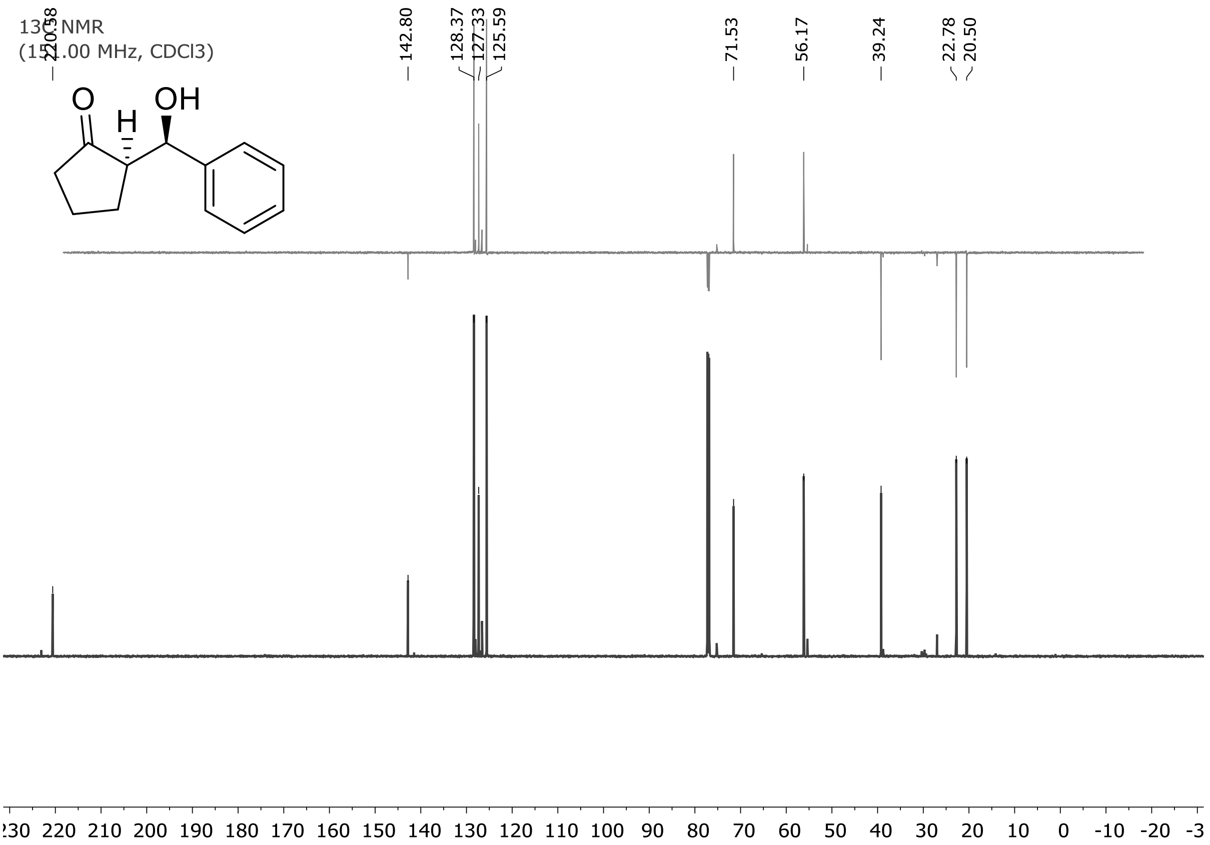

## 2-(hydroxy(phenyl)methyl)cycloheptan-1-one (4d)

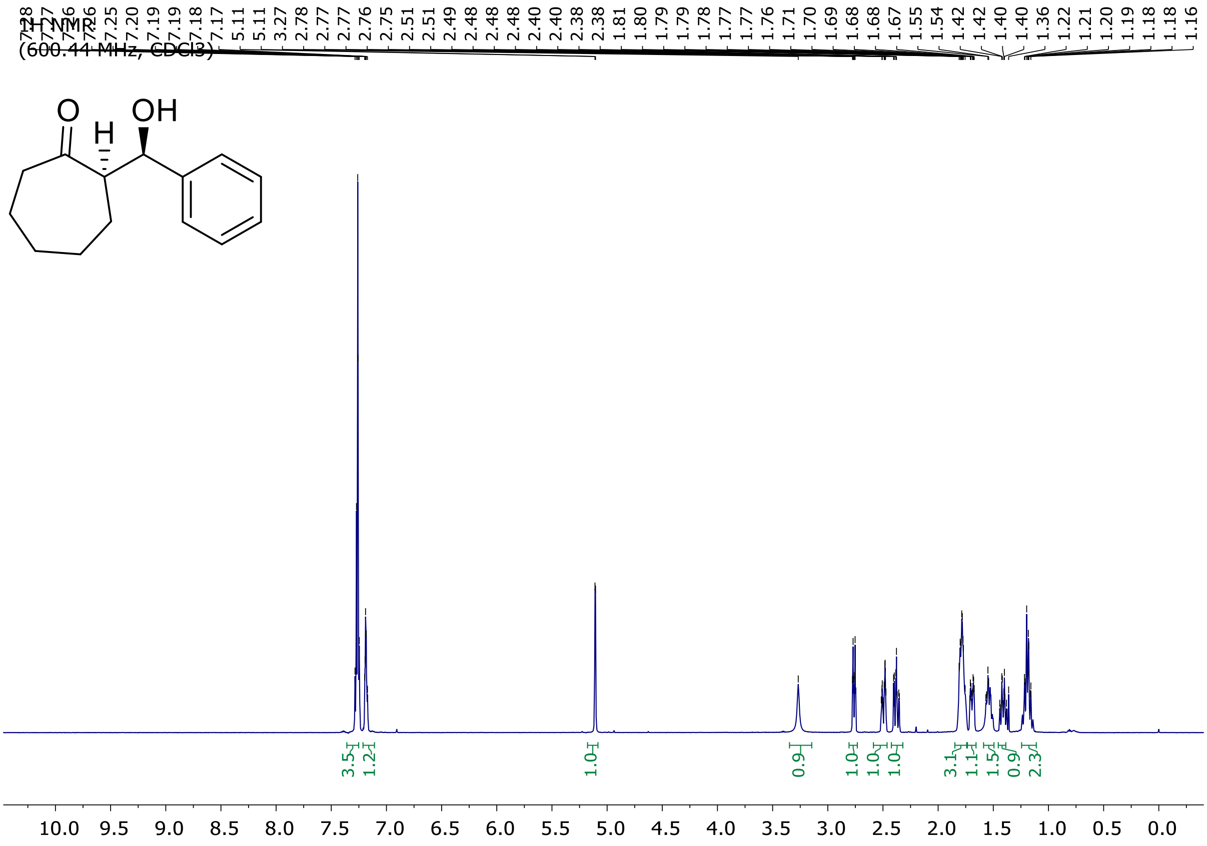


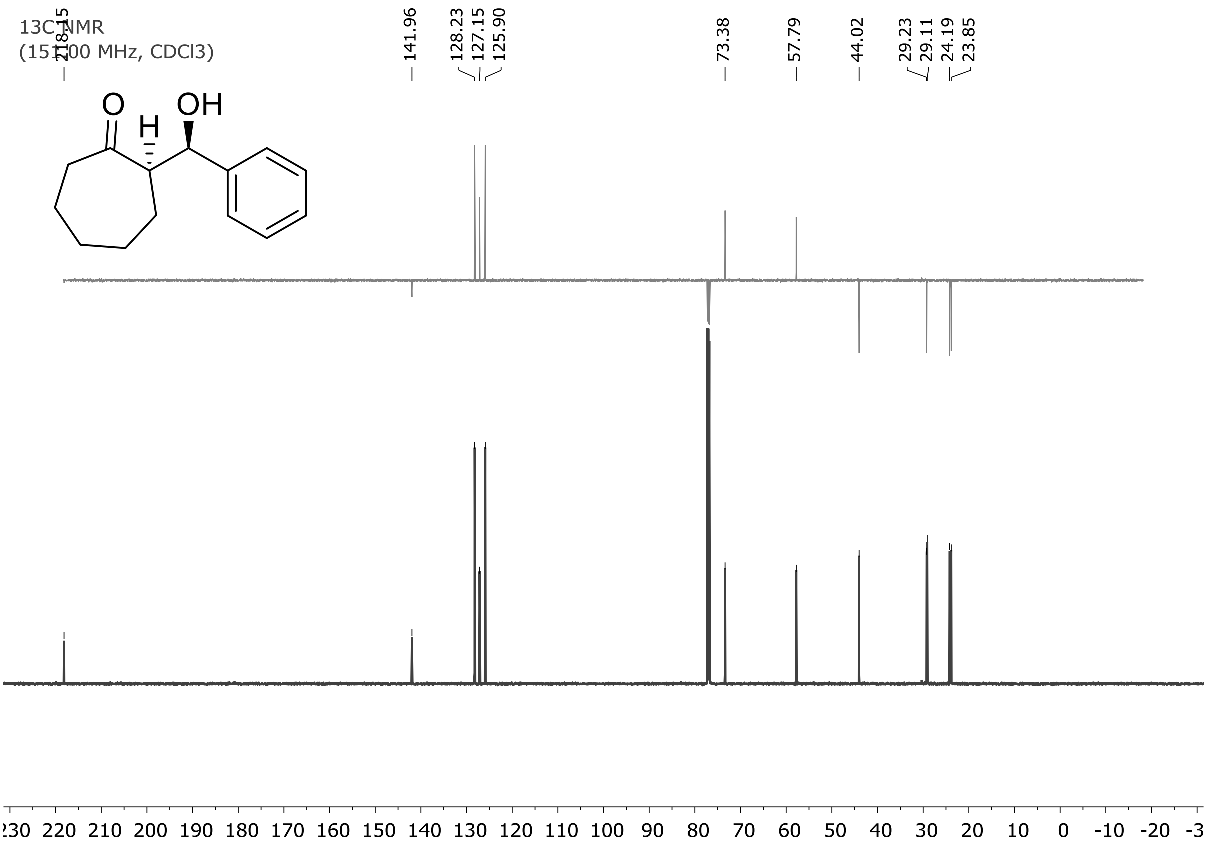

## 6-(hydroxy(phenyl)methyl)-6,7,8,9-tetrahydro-5H-benzo[7]annulen-5-one (4e)

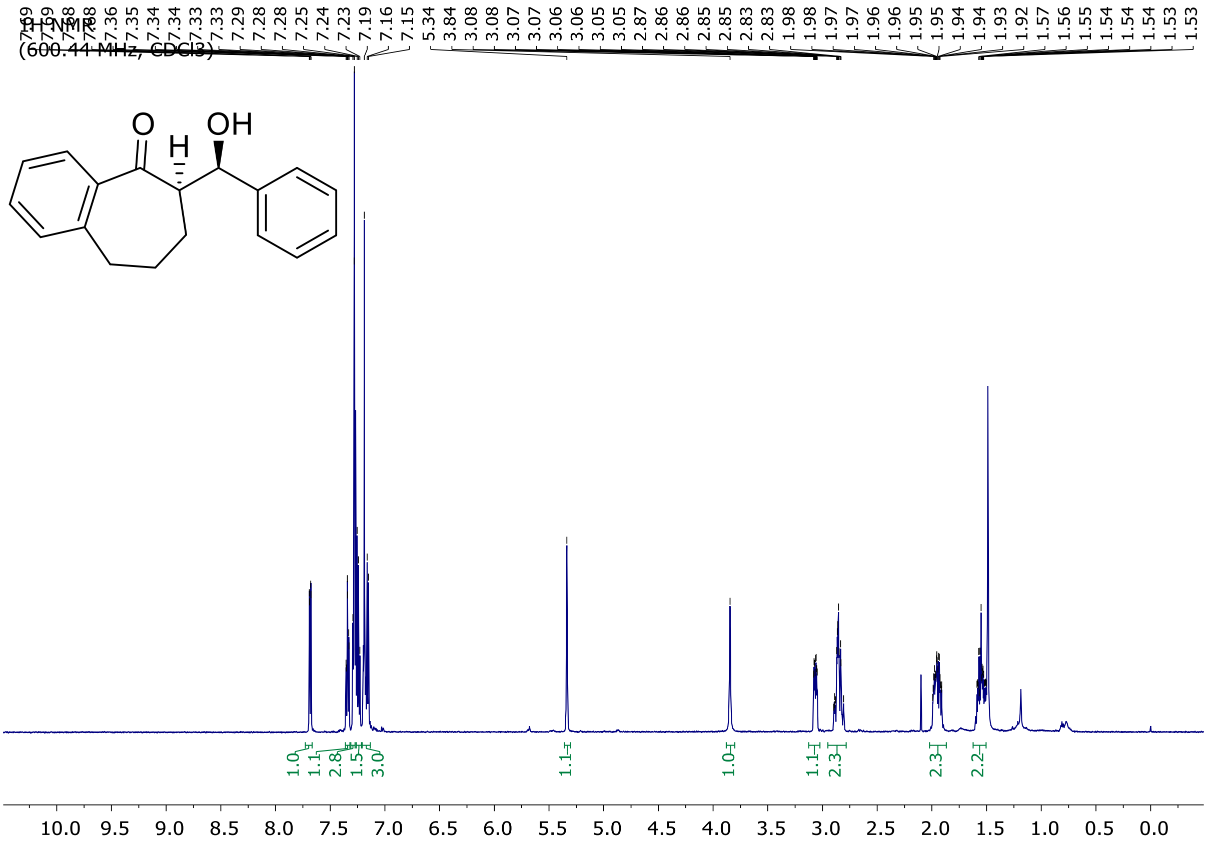


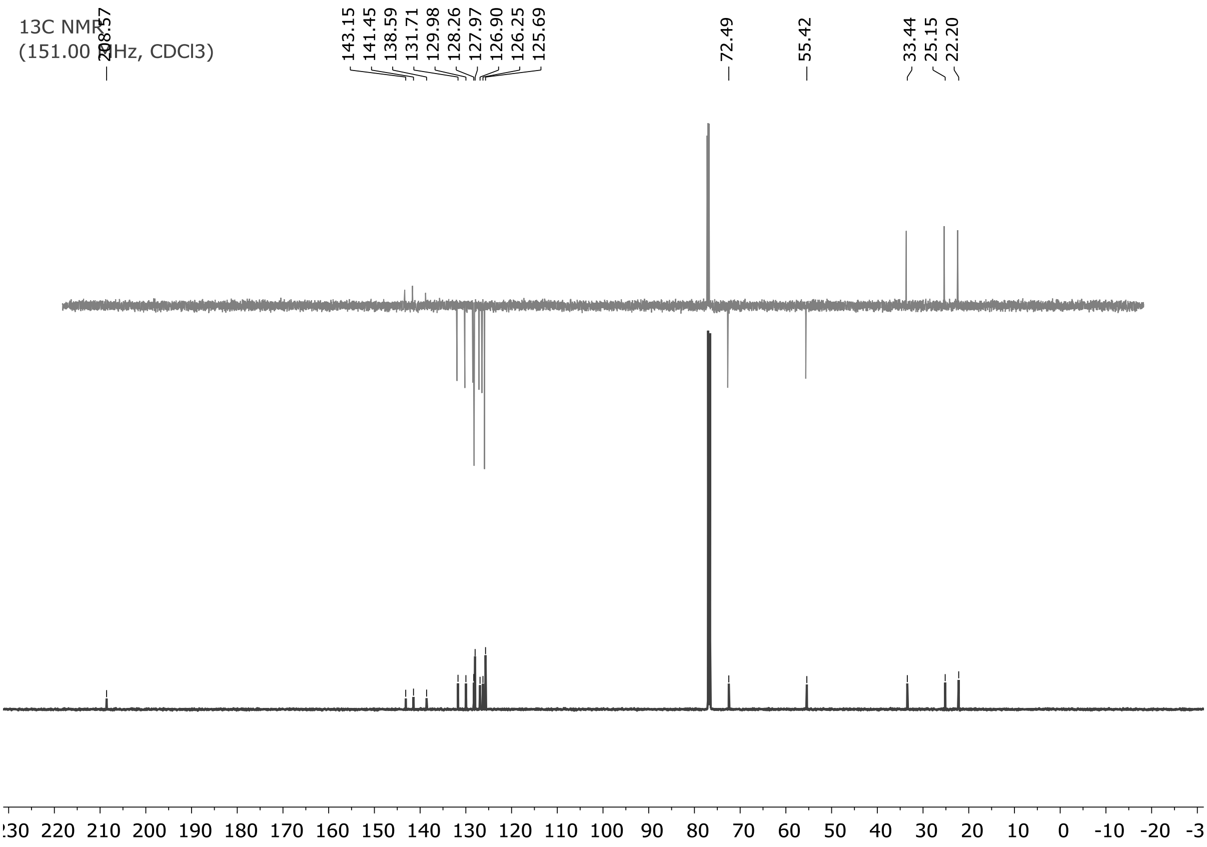

# 2-(hydroxy(phenyl)methyl)-2-methylcyclopentan-1-one (4f)

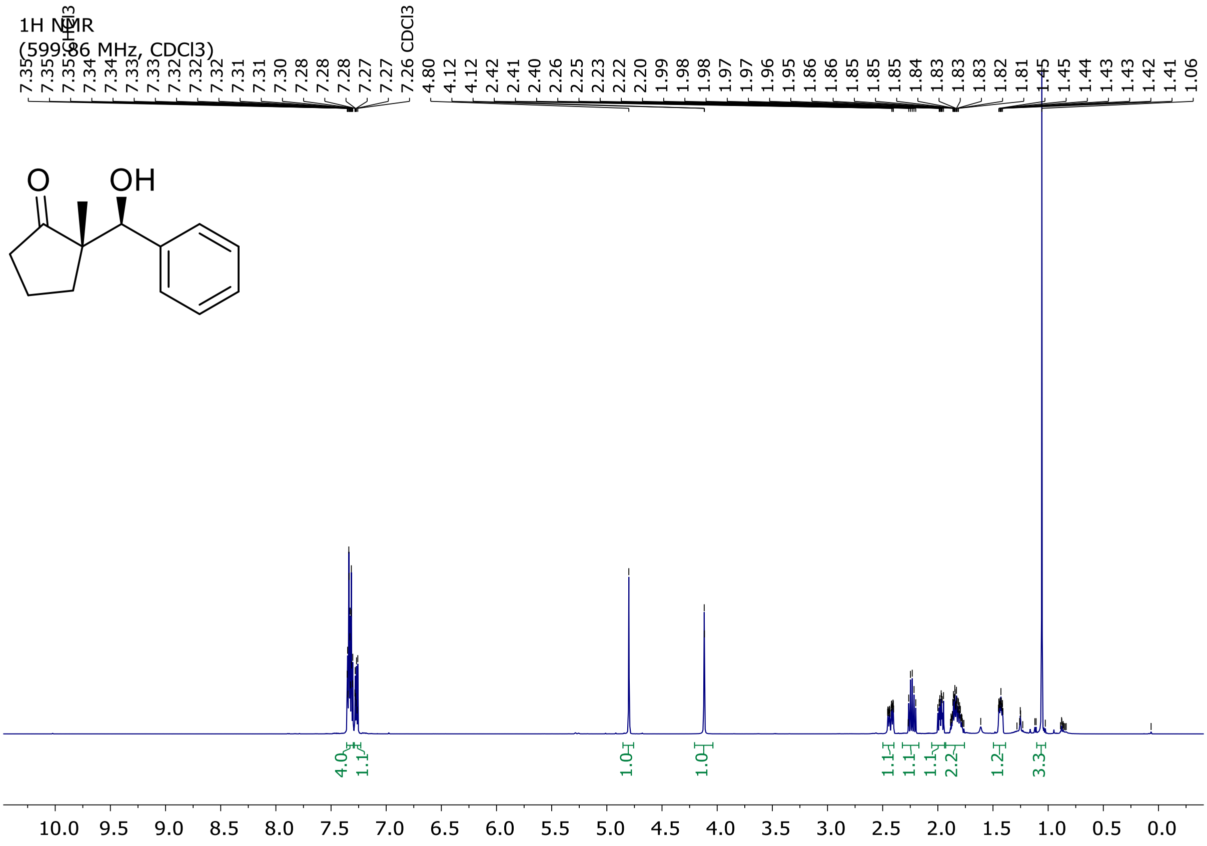


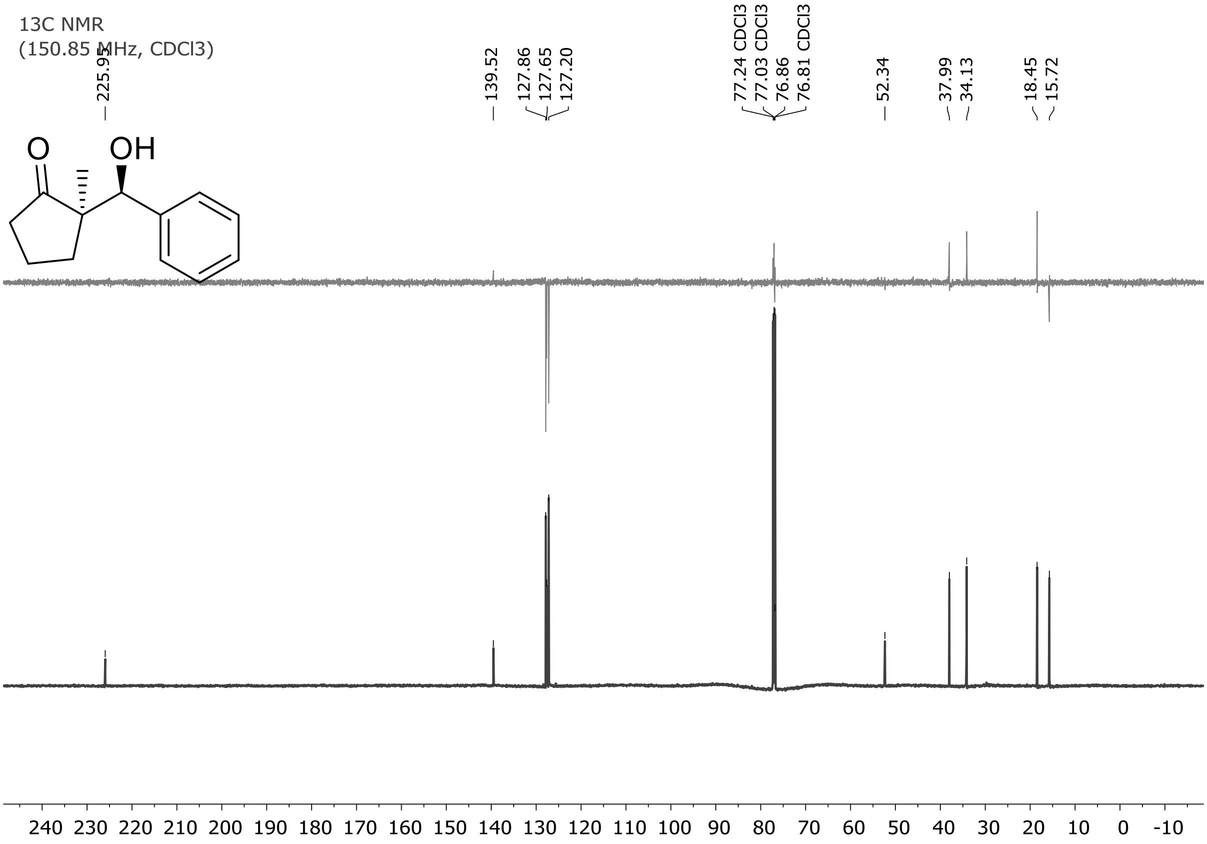

## 3-(hydroxy(phenyl)methyl)nonan-2-one (4g)

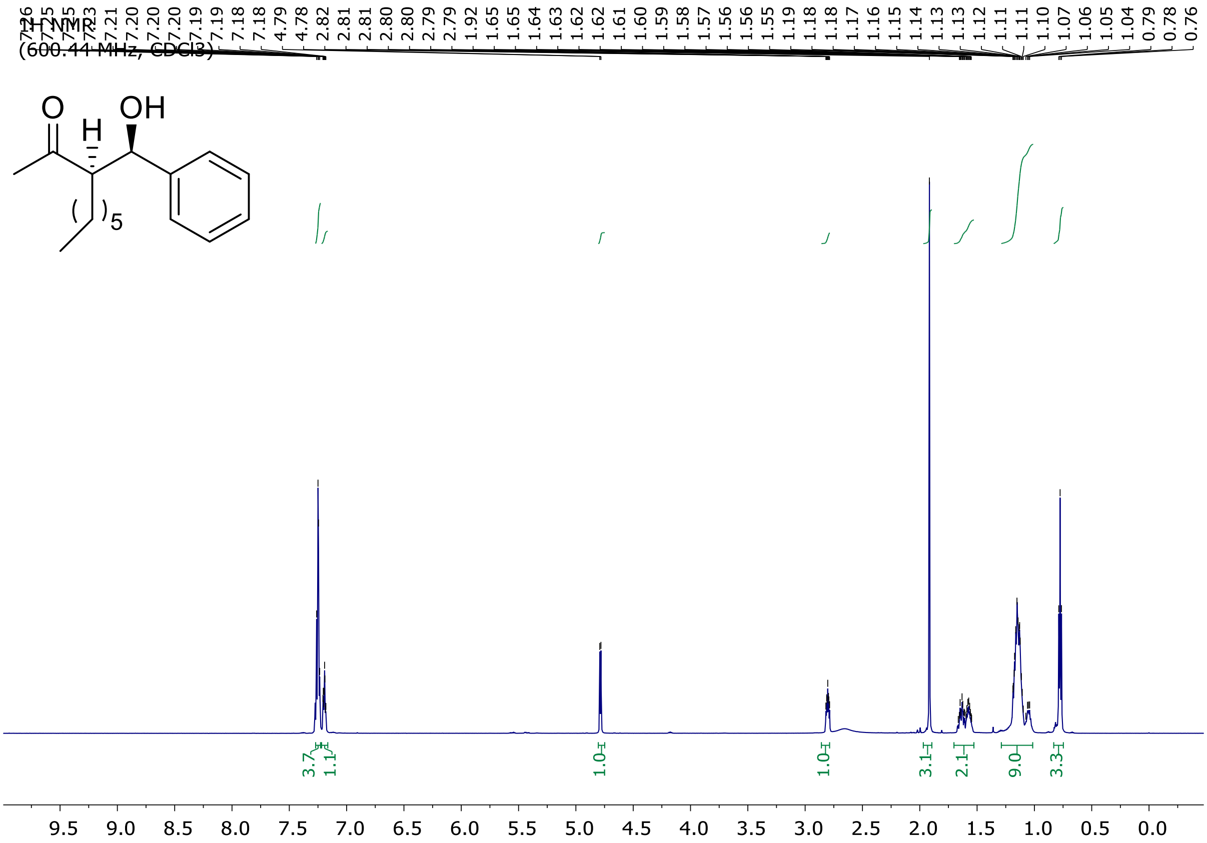


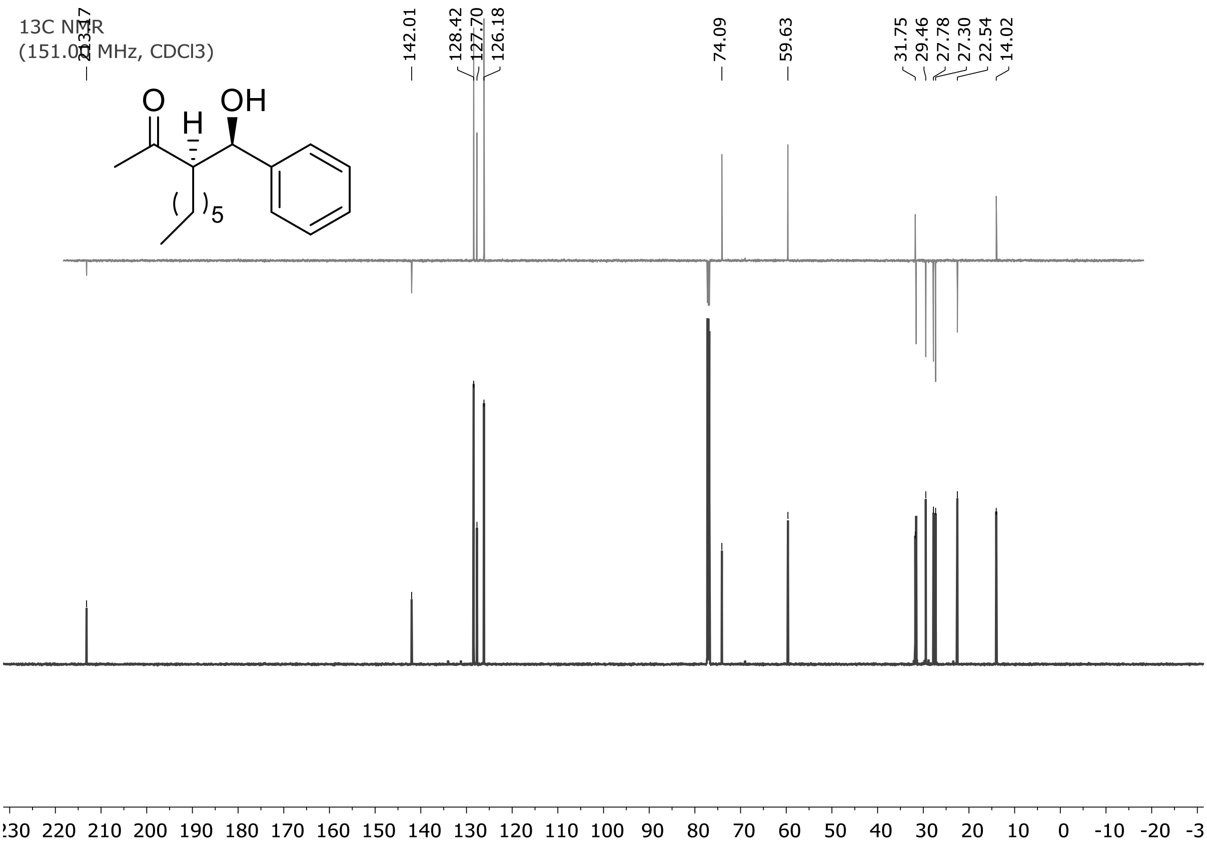

**(2R,8R,9S,10R,13S,14S,17S)-2-(hydroxy(phenyl)methyl)-10,13,17-trimethyl-17-((trimethylsilyl)oxy)-1,2,6,7,8,9,10,11,12,13,14,15,16,17-tetradecahydro-3H-cyclopenta[a]phenanthren-3-one (4h)**

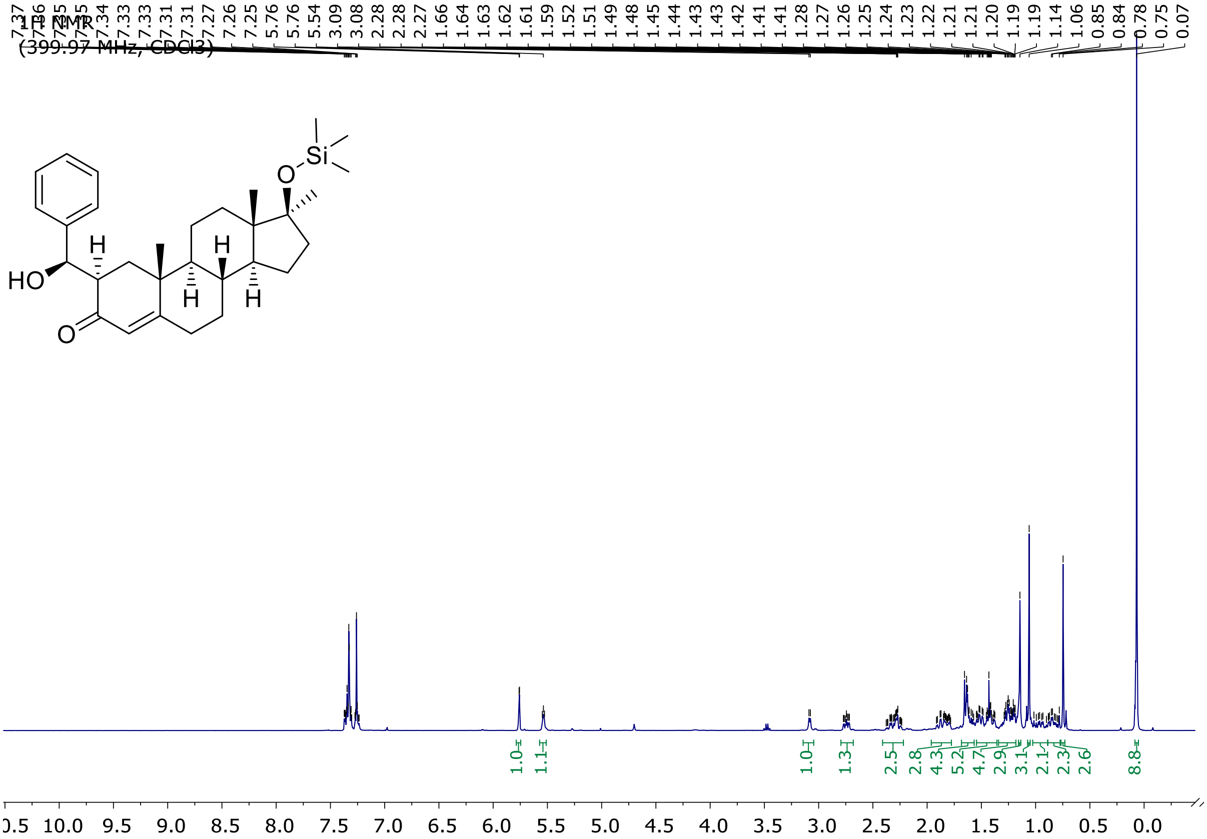


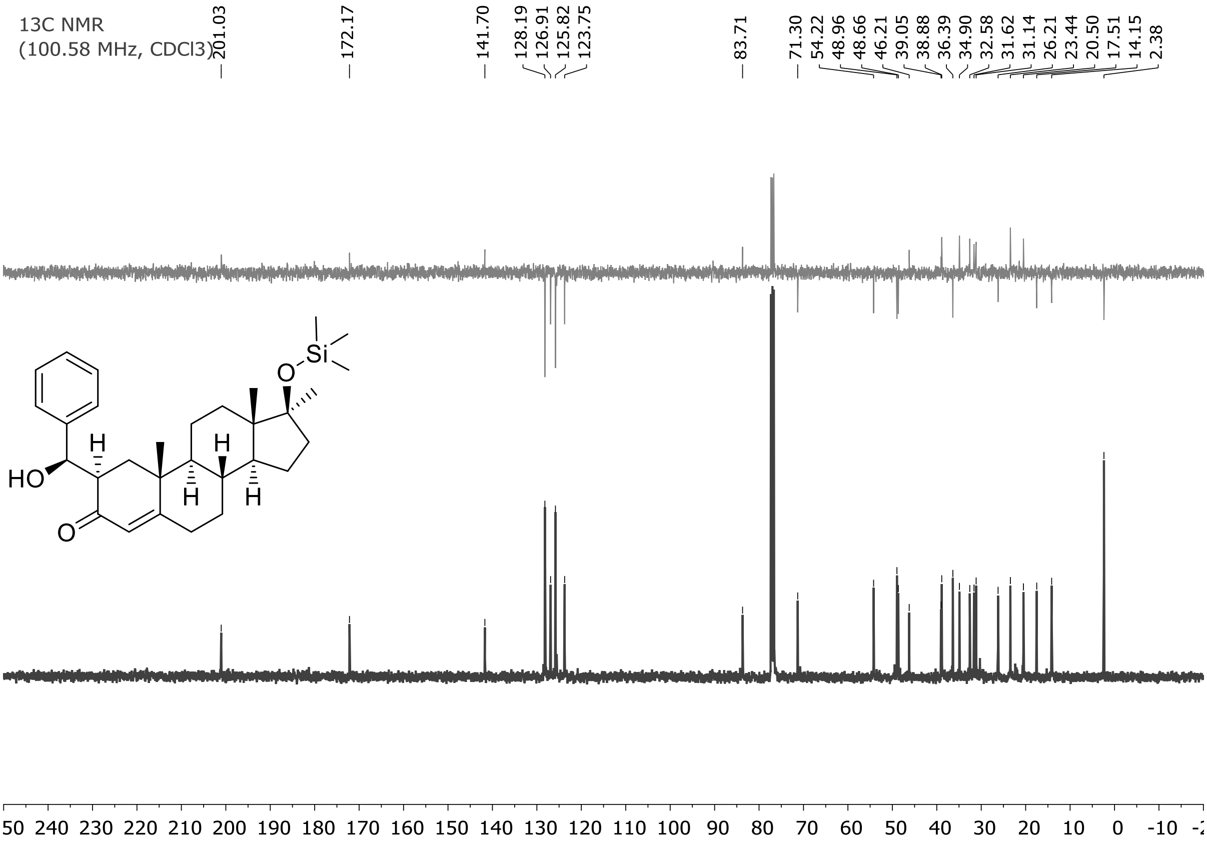

**2-((4-ethoxyphenyl)(hydroxy)methyl)-4,4-dimethylcyclohexan-1-one (4i)**

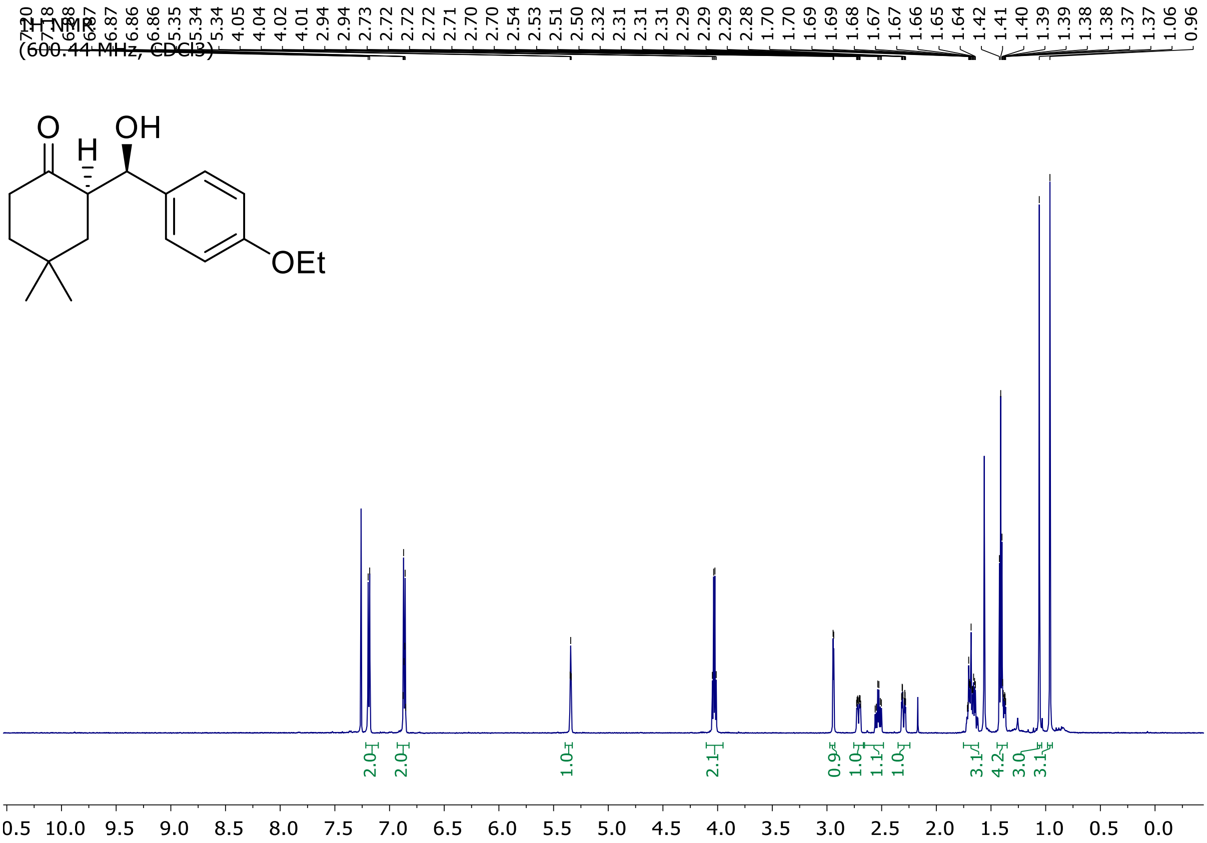


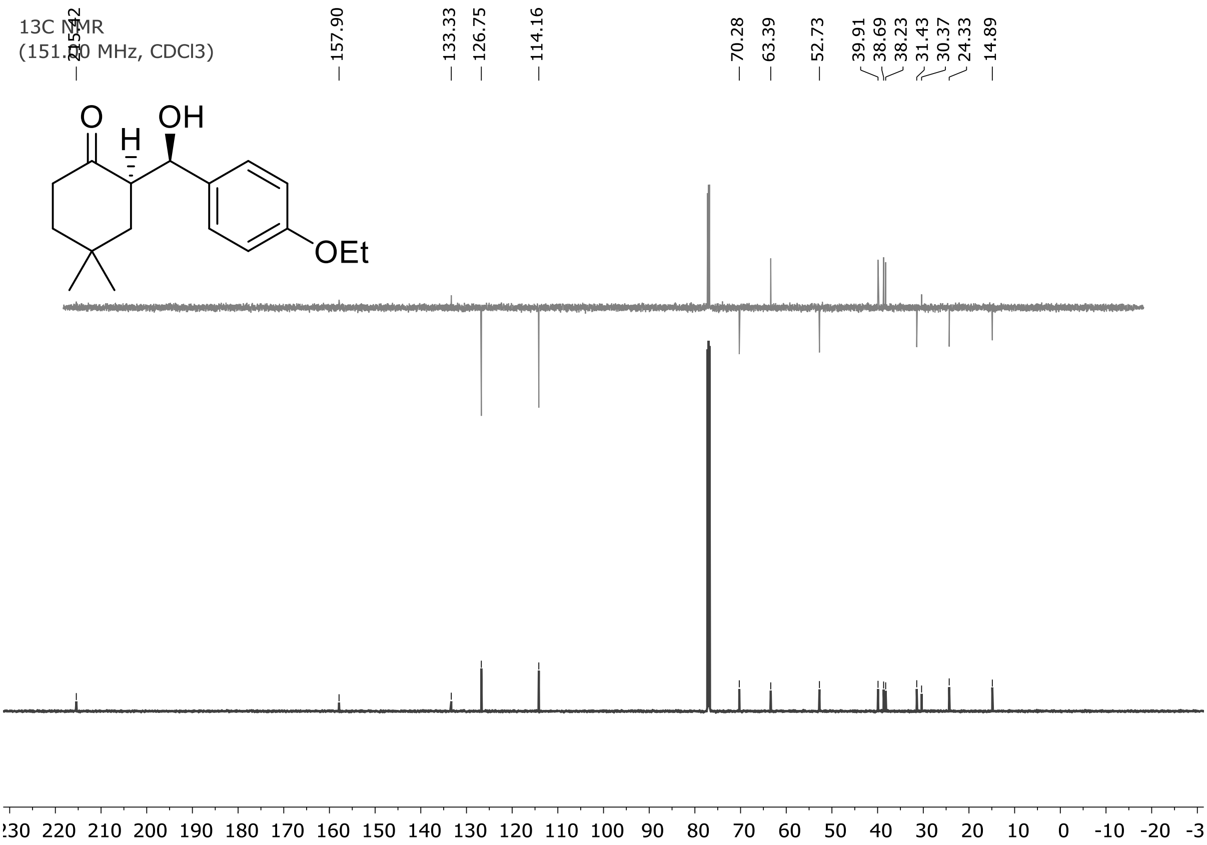

## 2-(hydroxy(p-tolyl)methyl)-4,4-dimethylcyclohexan-1-one (4j)

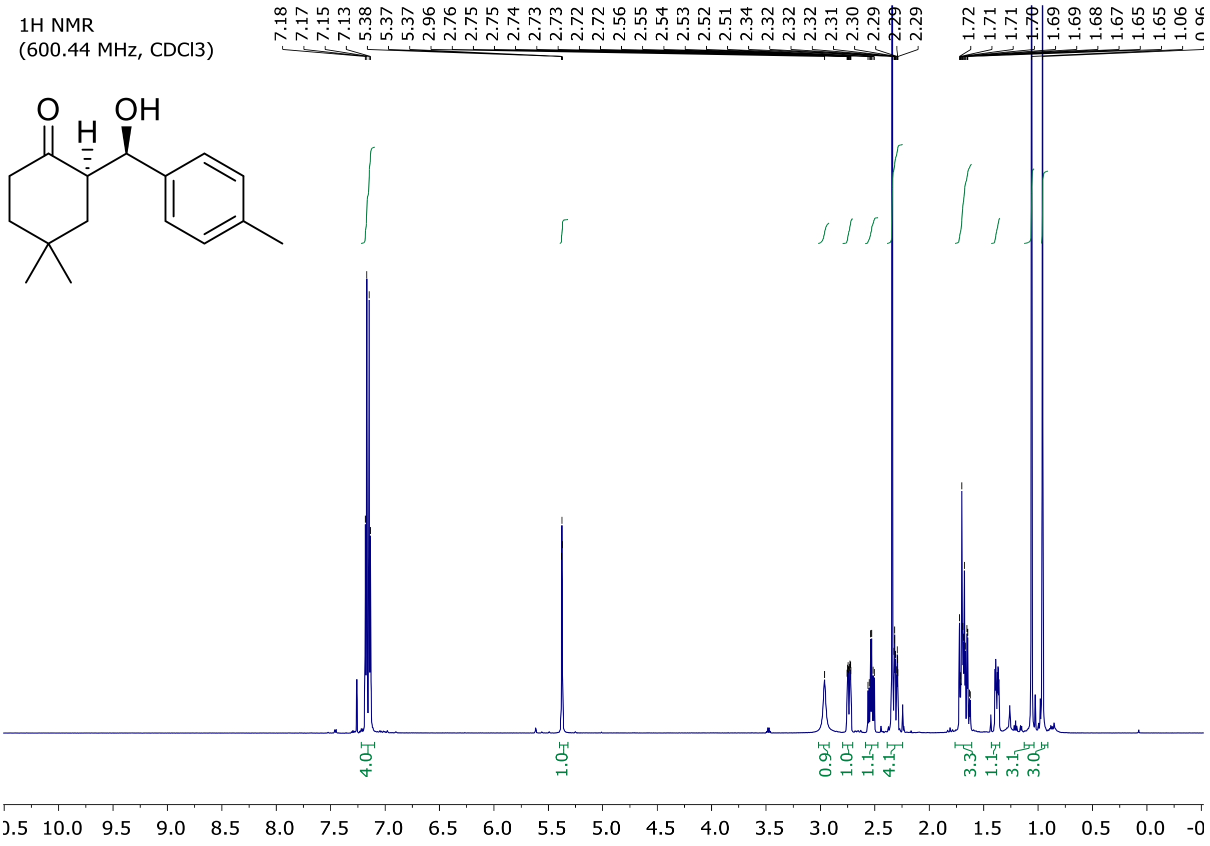


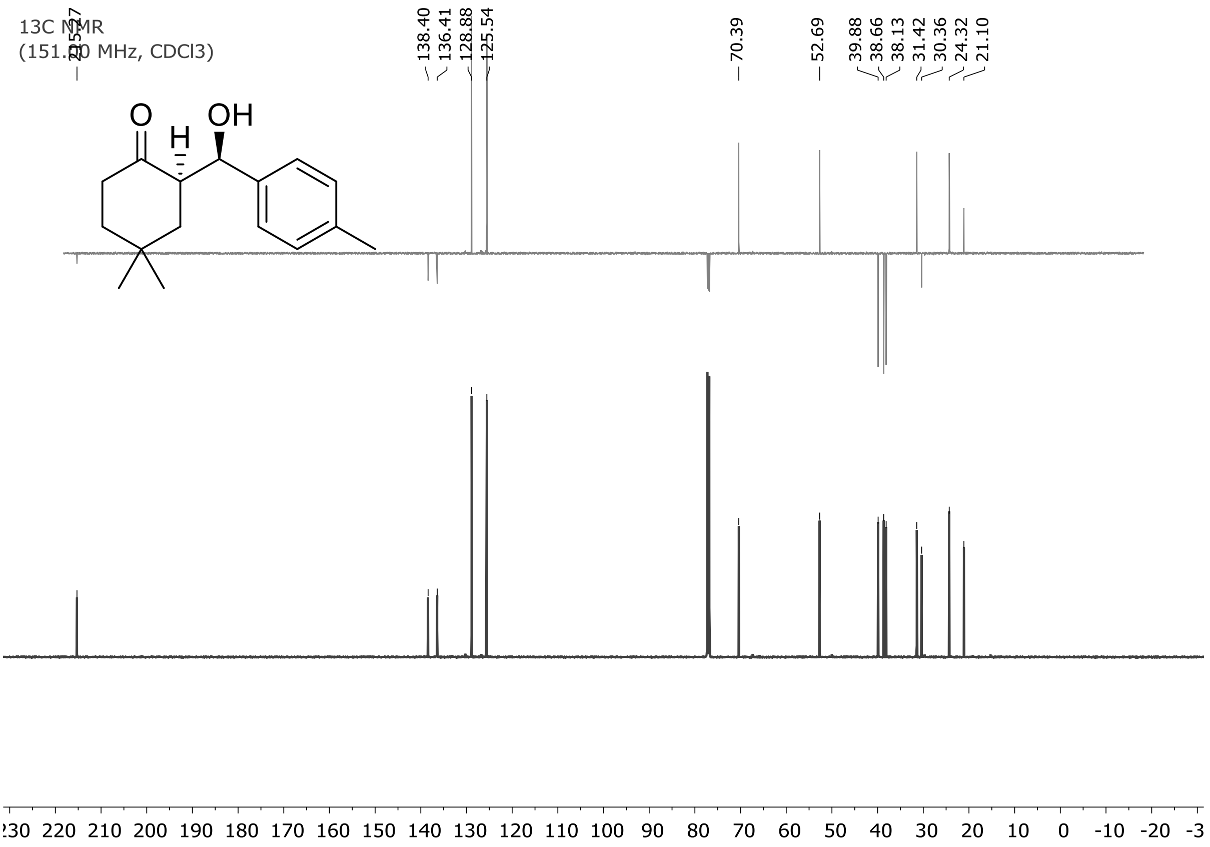

**2-((4-chlorophenyl)(hydroxy)methyl)-4,4-dimethylcyclohexan-1-one (4k)**

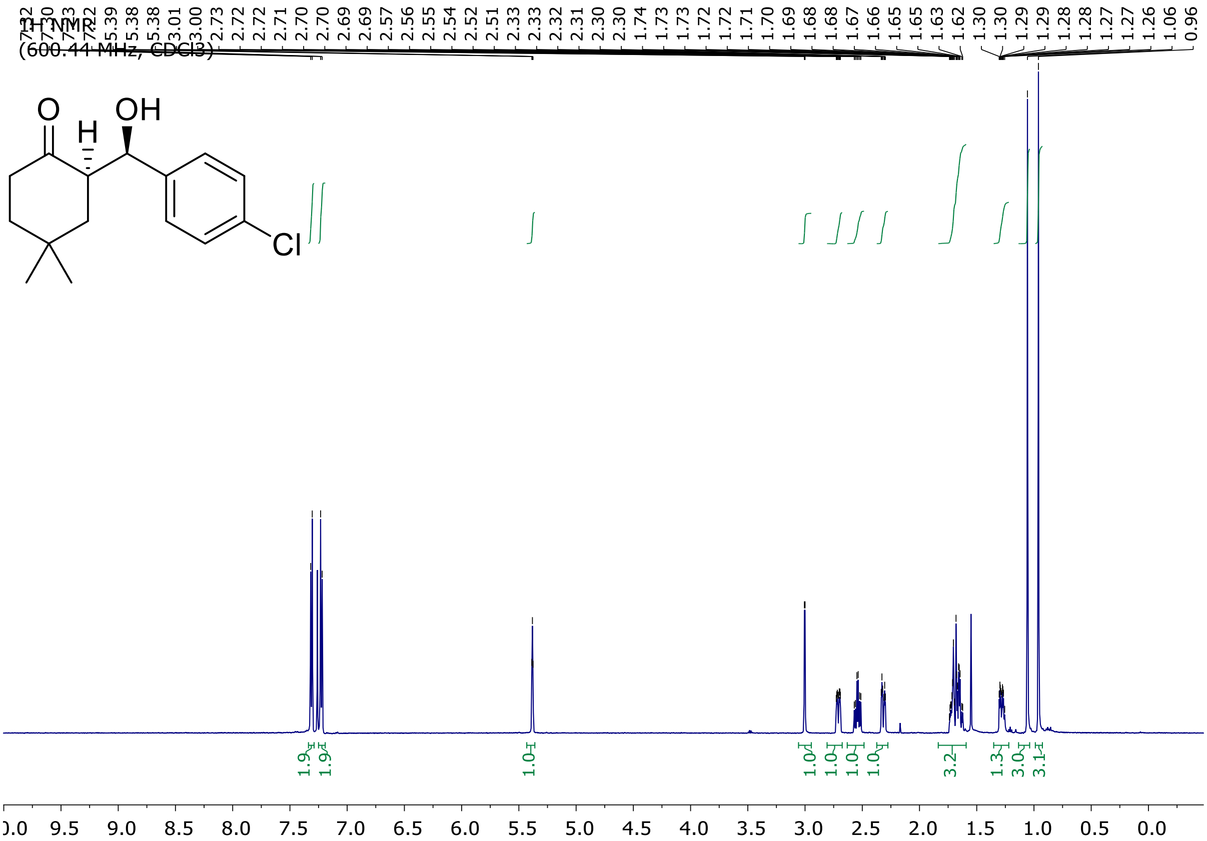


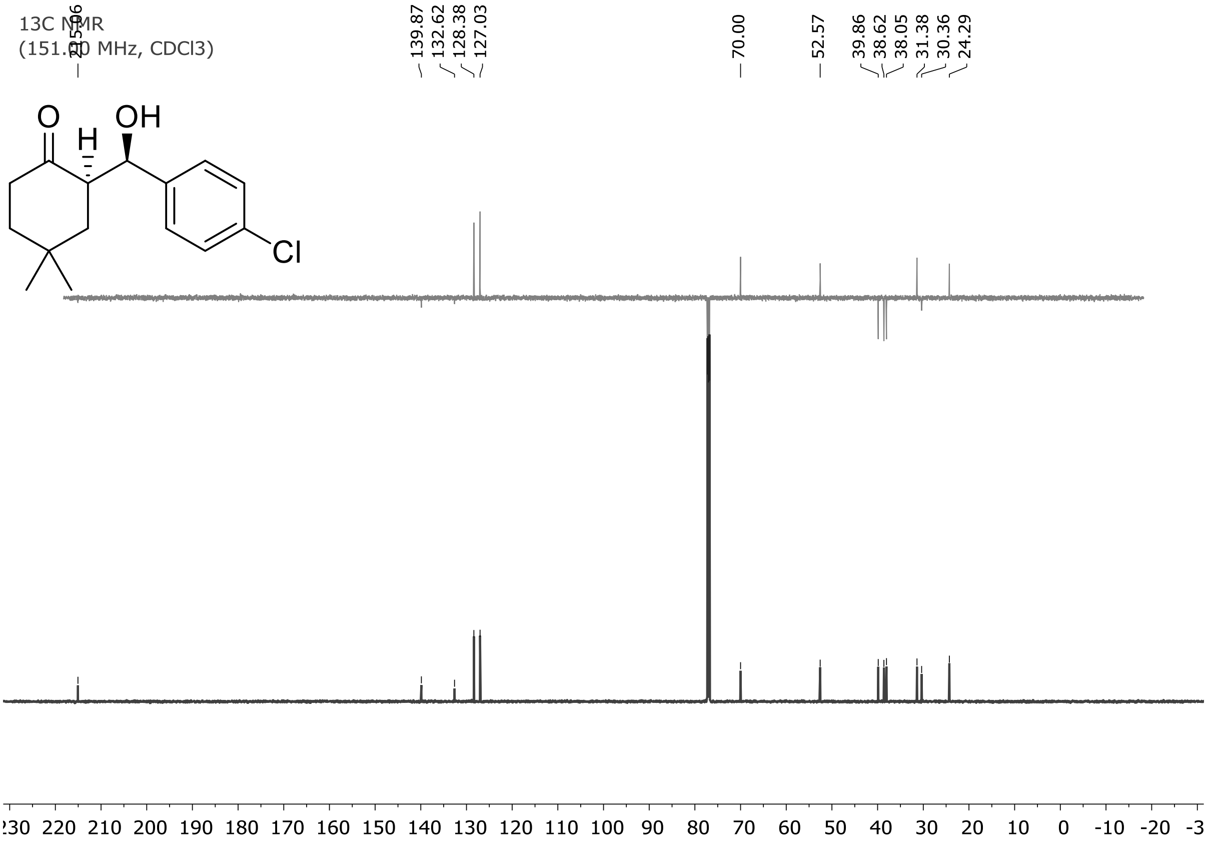

## 2-(-hydroxy(4-(trifluoromethyl)phenyl)methyl)-4,4-dimethylcyclohexan-1-one (4l)

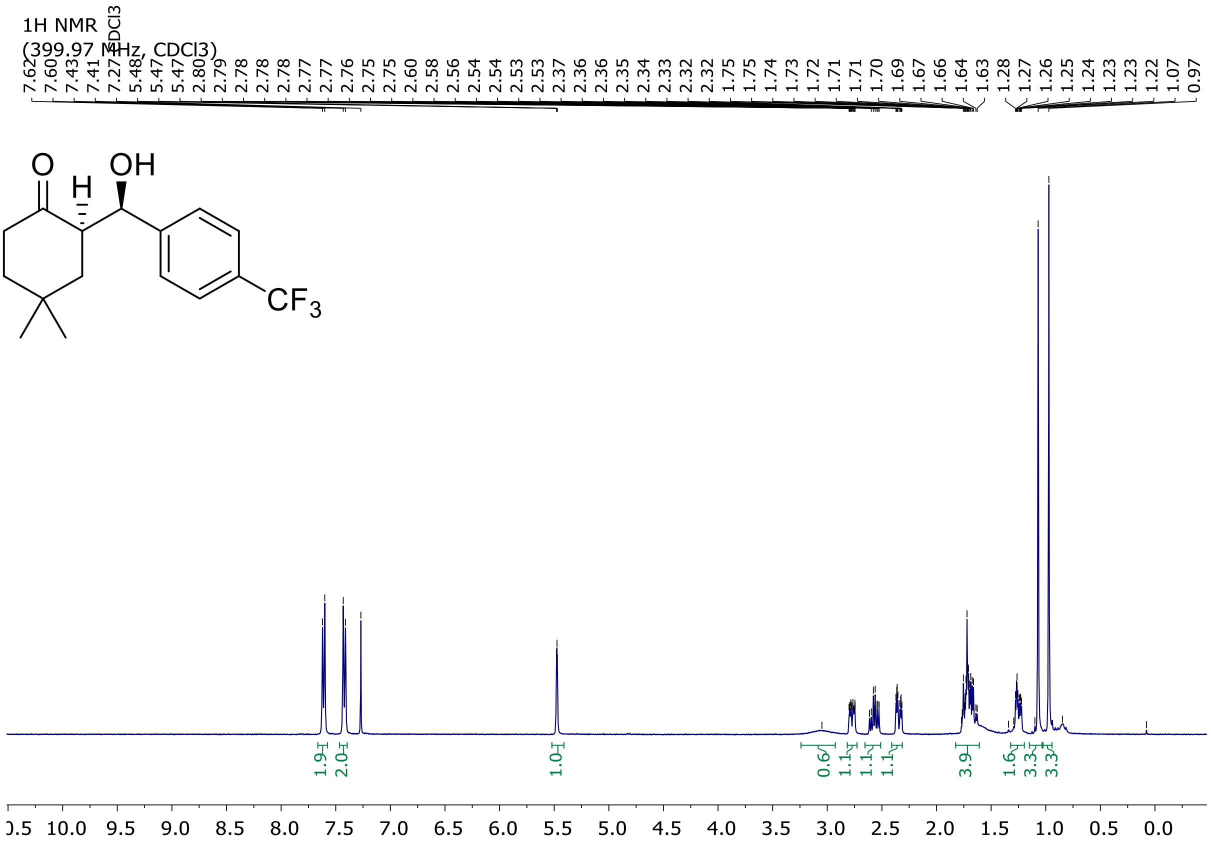


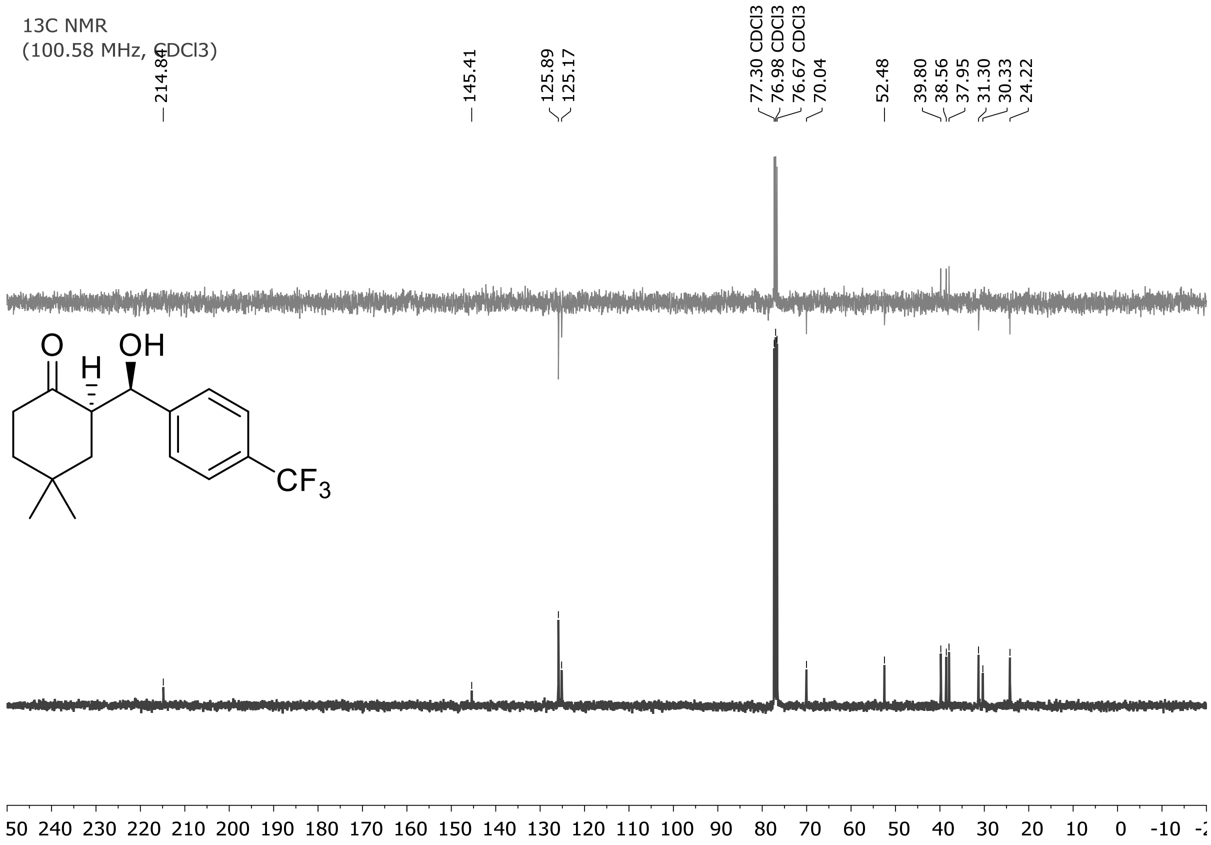

## 2-(furan-2-yl(hydroxy)methyl)-4,4-dimethylcyclohexan-1-one (4m)

1H NMR
(600.44 MHz, CDCl3)

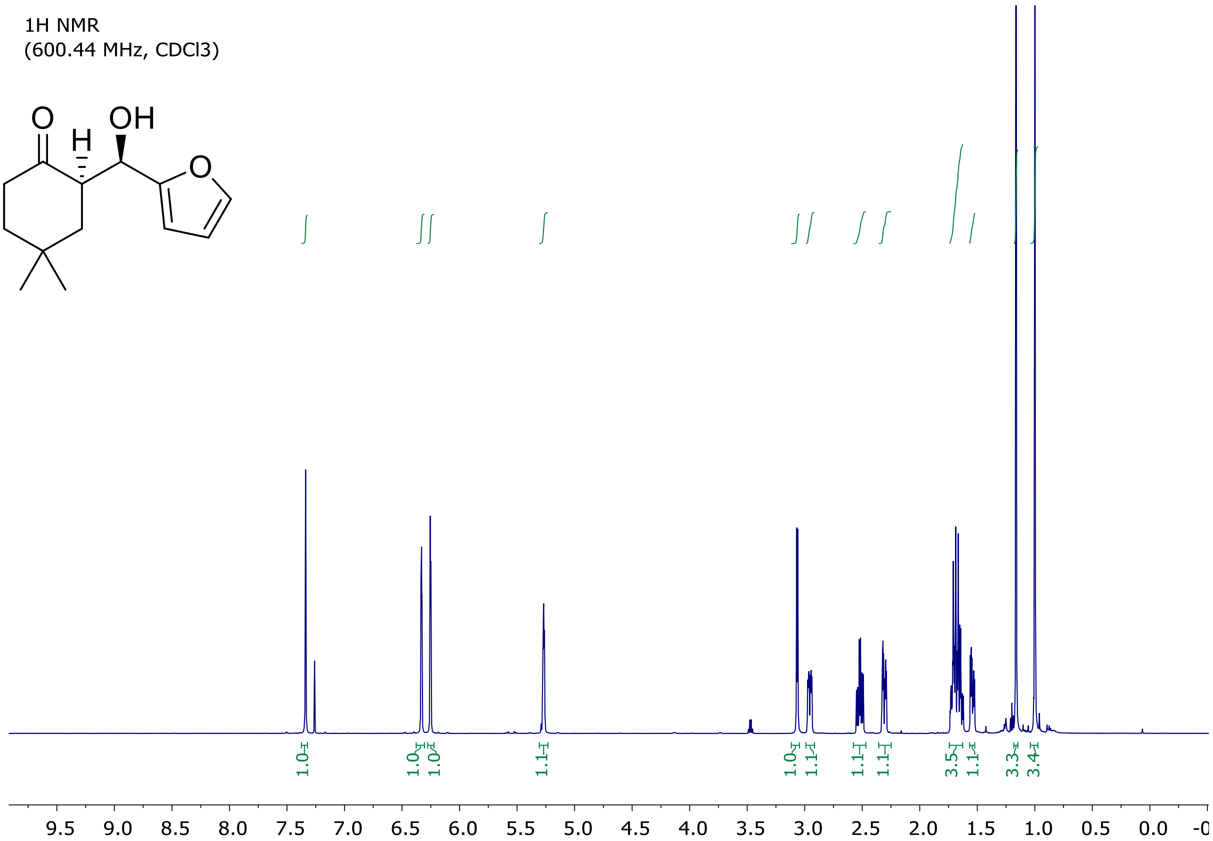


13C NMR
(151.00 MHz, CDCl3)

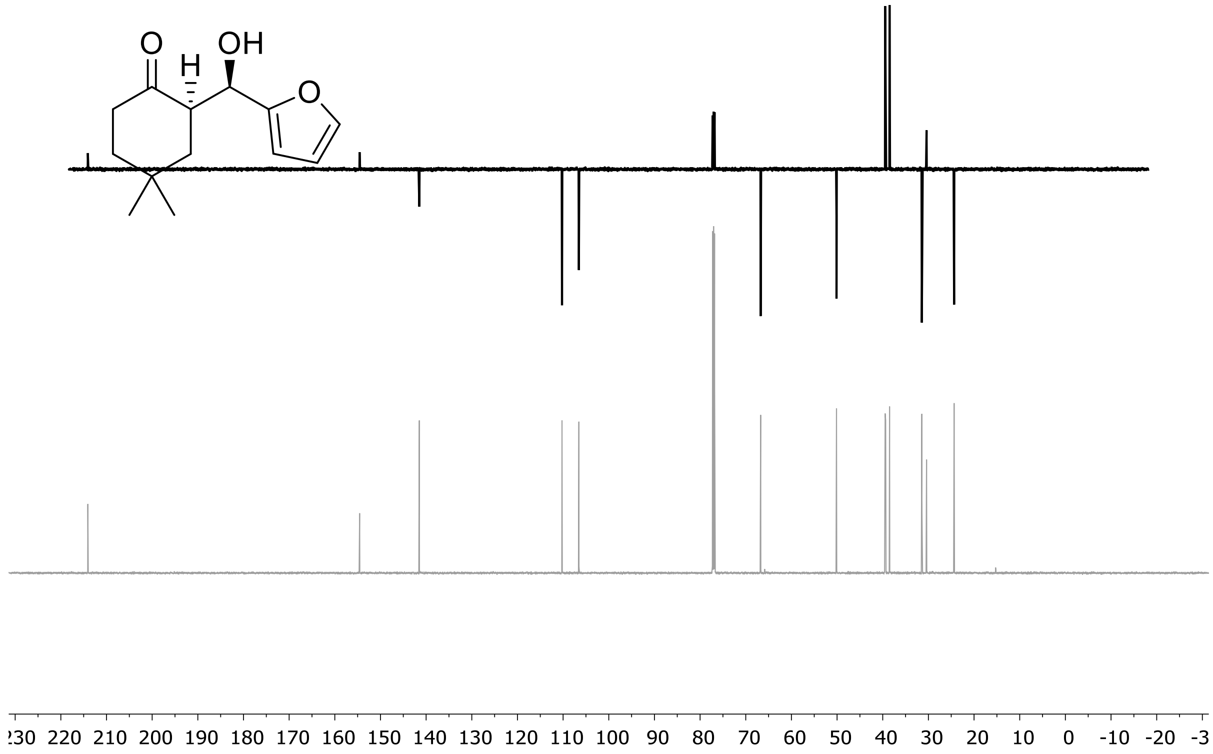

## 2-(hydroxy(thiophen-2-yl)methyl)-4,4-dimethylcyclohexan-1-one (4n)

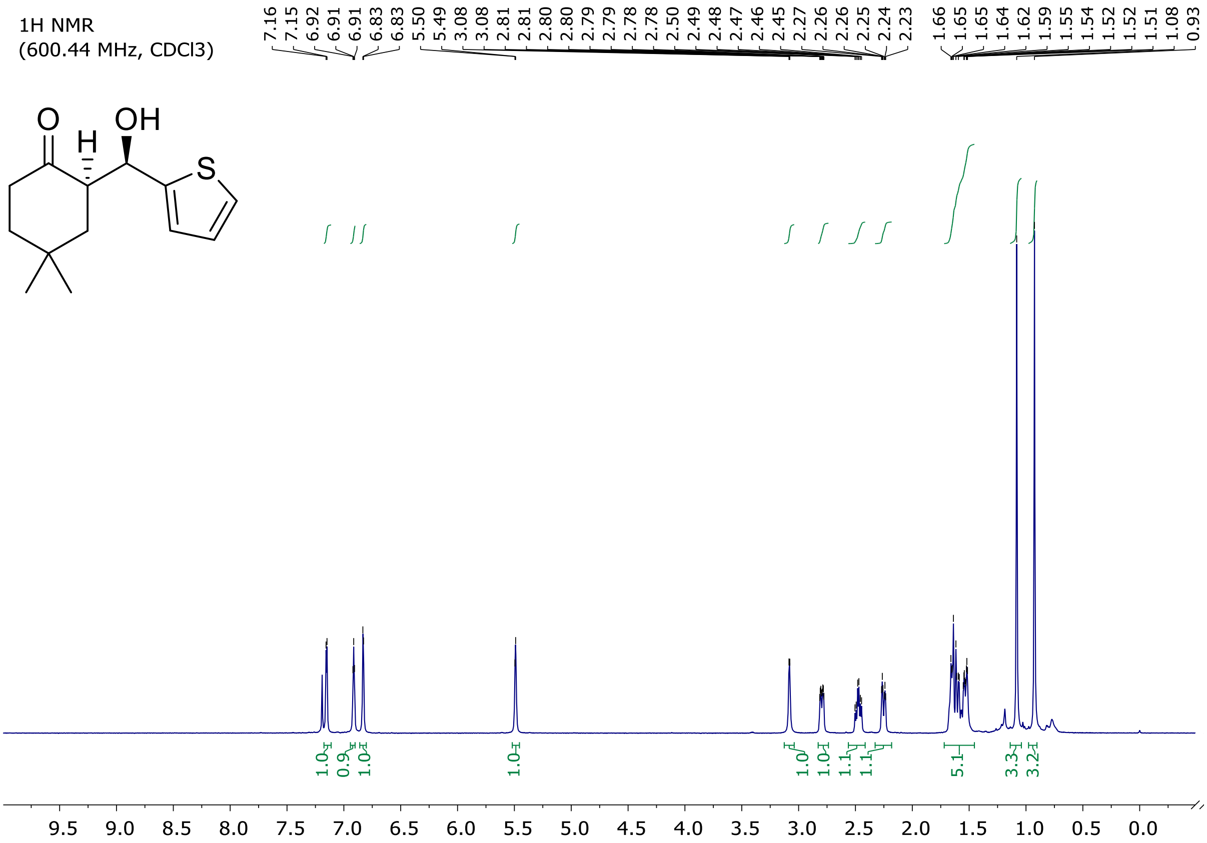


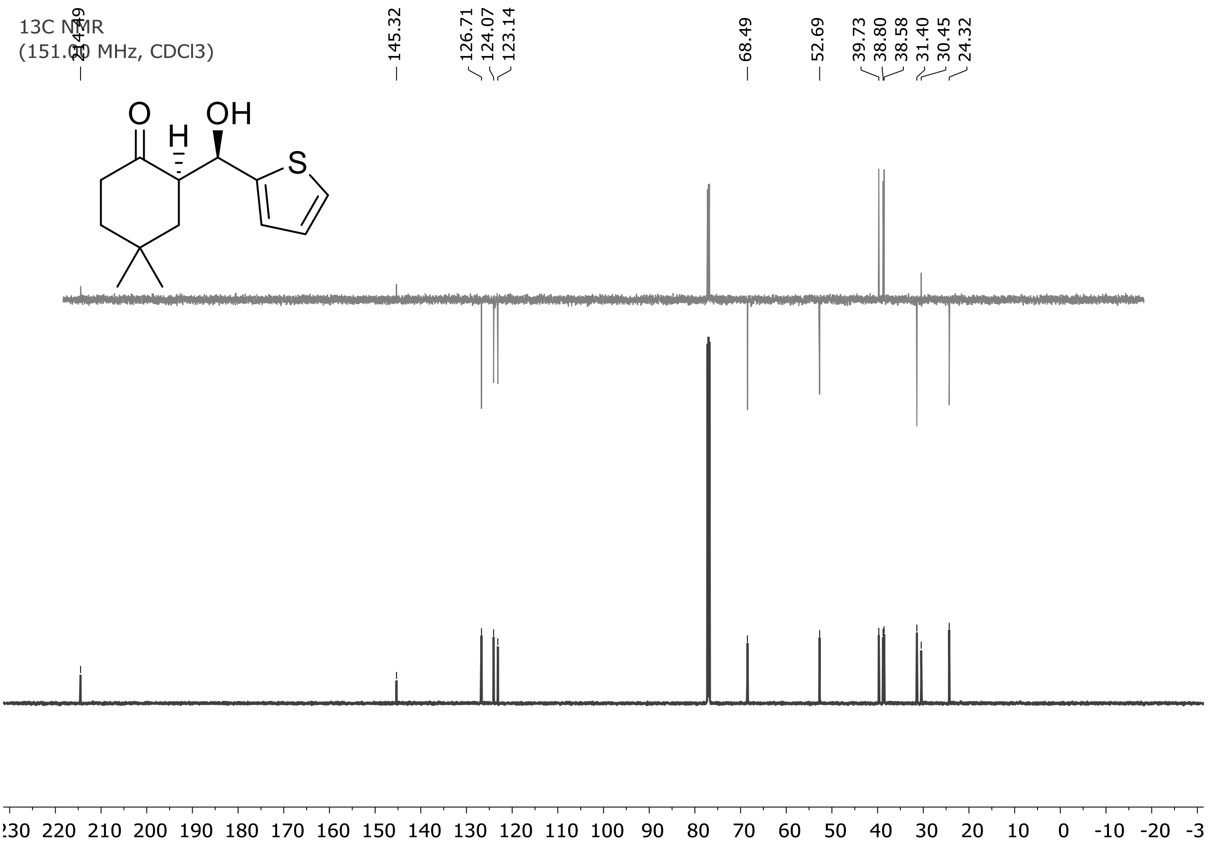

## 2-(E-1-hydroxy-3-phenylallyl)-4,4-dimethylcyclohexan-1-one (4o)

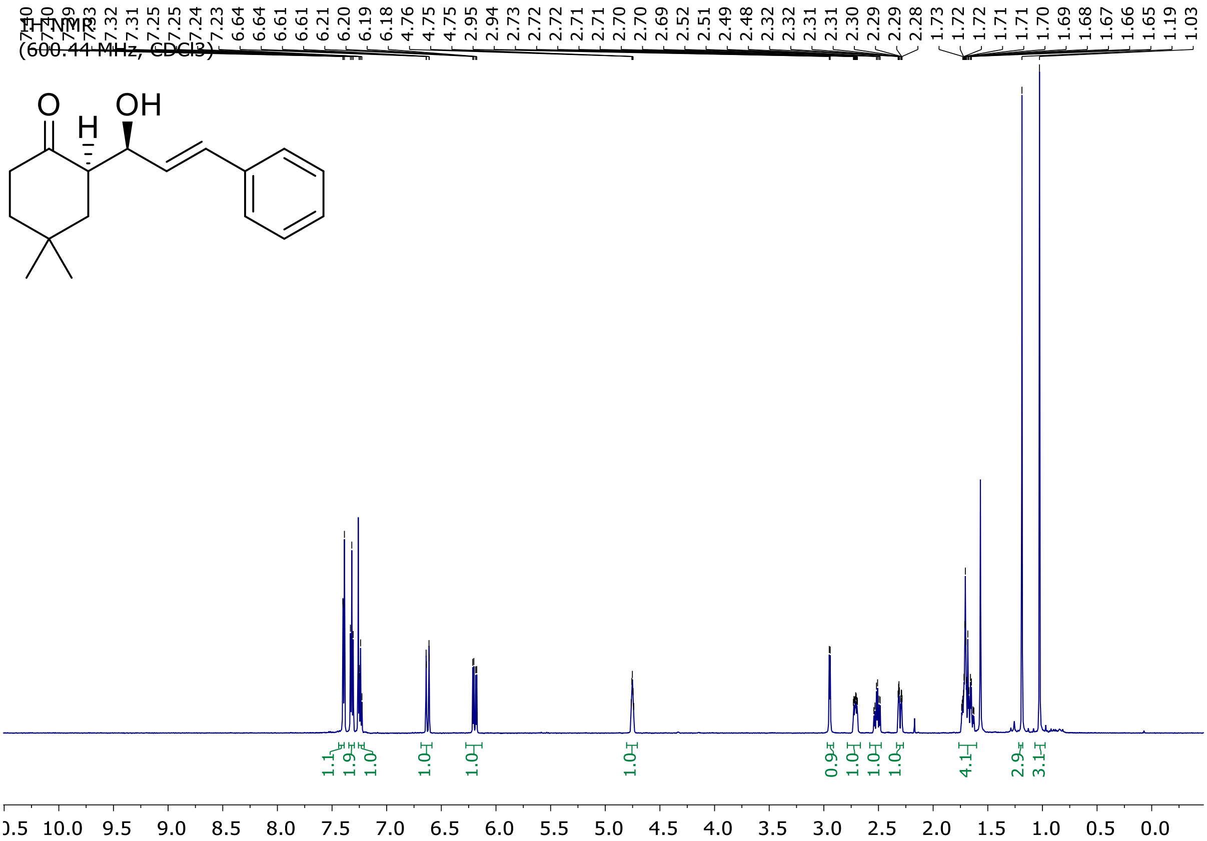


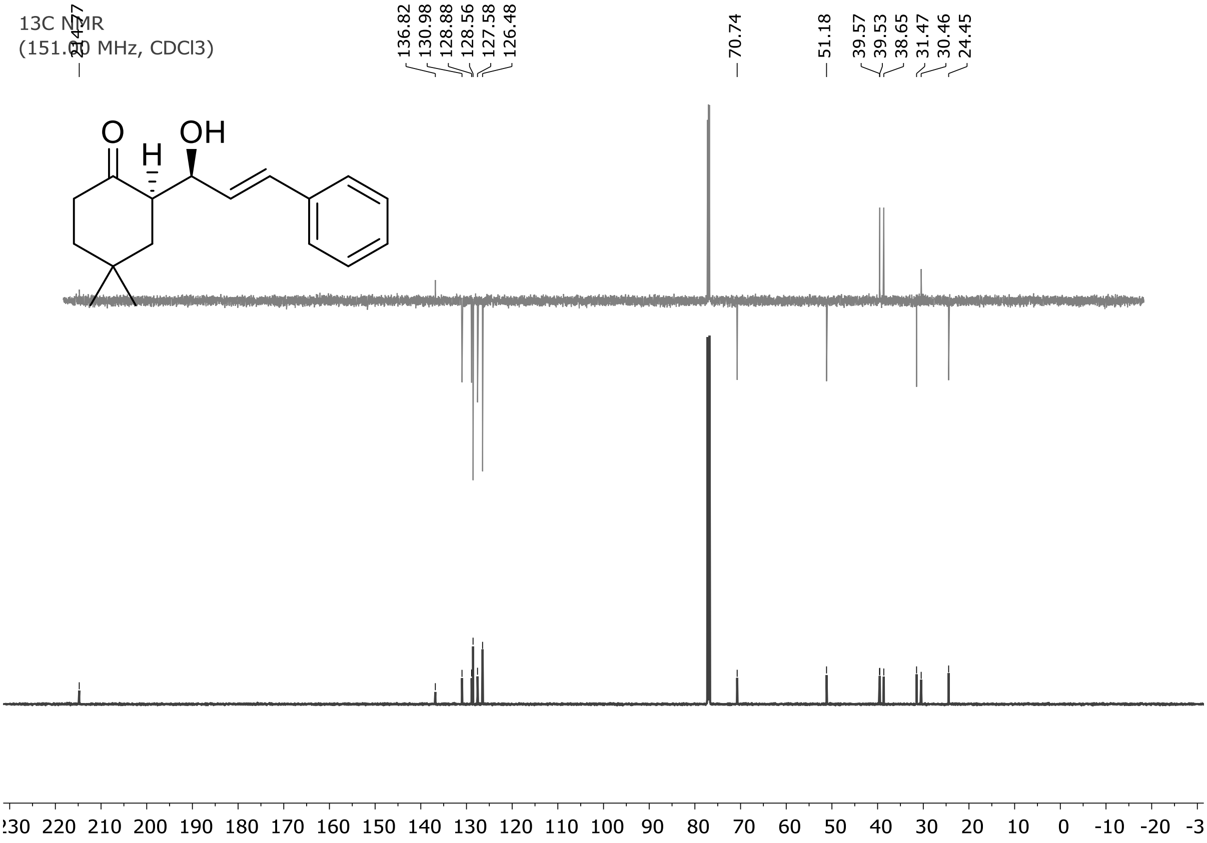

## 2-(1-hydroxy-2-methylpropyl)-4,4-dimethylcyclohexan-1-one (4p)

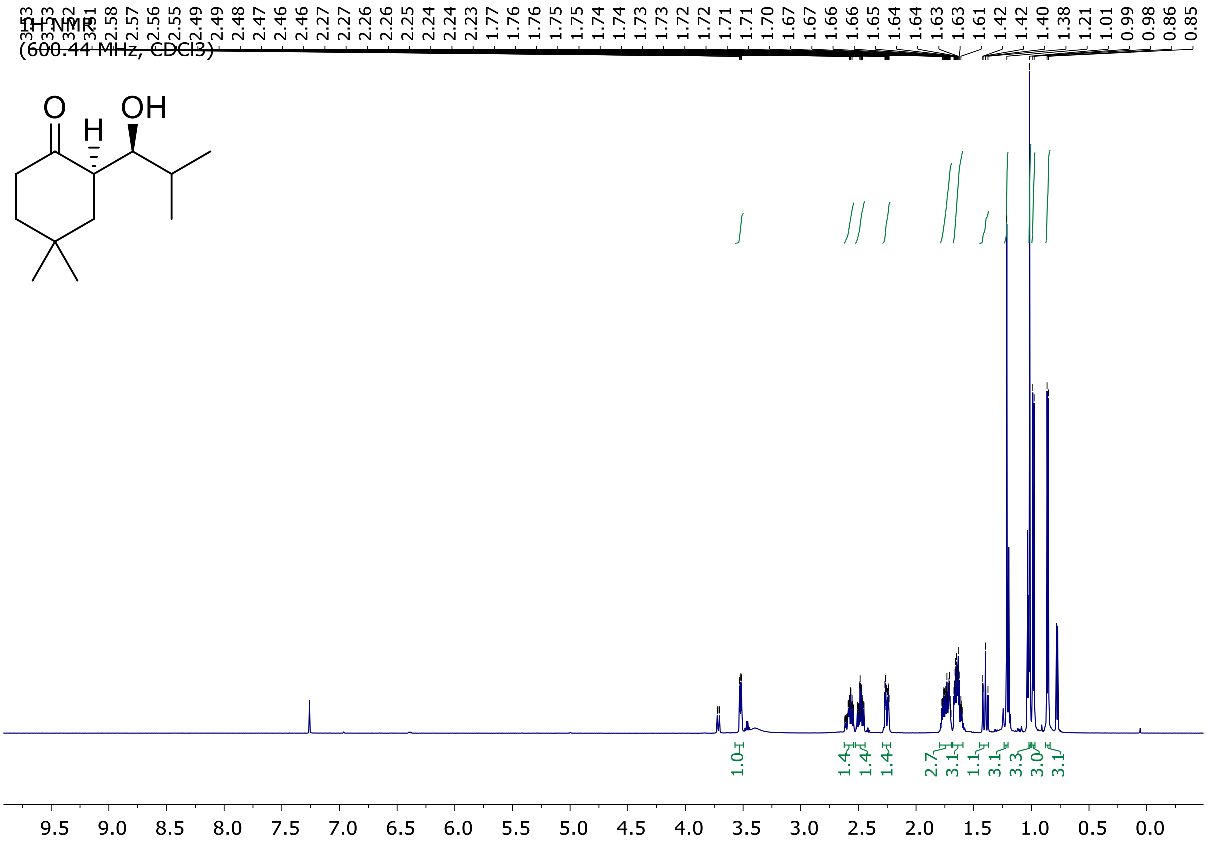


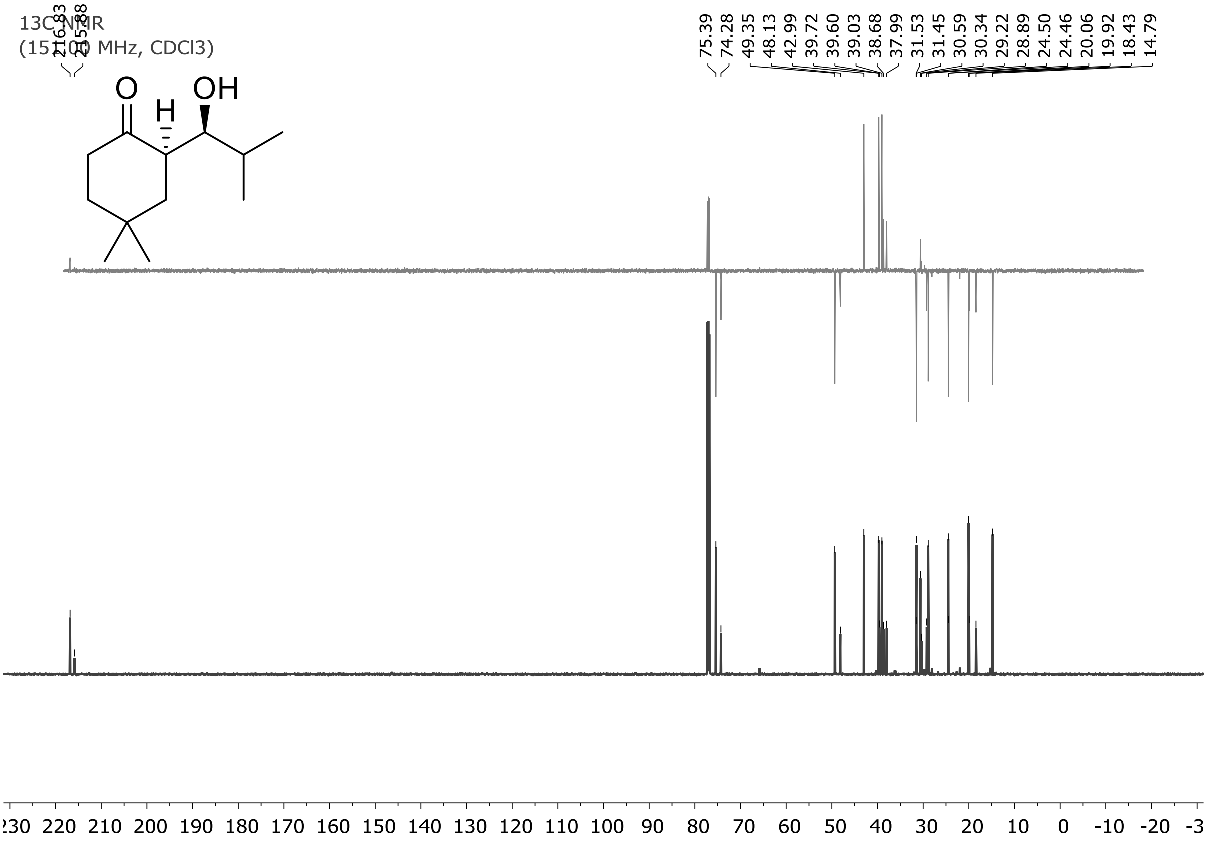

## 8. References


(1) Liang, S.; Zeng, C.-C.; Tian, H.-Y.; Sun, B.-G.; Luo, X.-G.; Ren, F. Electrochemically Oxidative α-C–H Functionalization of Ketones: A Cascade Synthesis of α-Amino Ketones Mediated by $NH_4$ I. *J. Org. Chem.* **2016**, *81* (23), 11565–11573. https://doi.org/10.1021/acs.joc.6b01595.

(2) Podraza, K. F.; Bassfield, R. L. Regiospecific Alkylation of 3-Substituted-2-Cyclohexen-1-Ones. Synthesis and Conformational Analysis of 6-(Carbethoxymethyl)-3-Substituted-2-Cyclohexen-1-Ones. *J. Org. Chem.* **1989**, *54* (25), 5919–5922. https://doi.org/10.1021/jo00286a023.

(3) Yuan, Q.; Prater, M. B.; Sigman, M. S. Enantioselective Synthesis of Γ-Functionalized Cyclopentenones and Δ-Functionalized Cycloheptenones Utilizing a Redox-Relay Heck Strategy. *Adv. Synth. Catal.* **2020**, *362* (2), 326–330. https://doi.org/10.1002/adsc.201901239.

(5) Diao, T.; Stahl, S.; Synthesis of Cyclic Enones via Direct Palladium-Catalyzed Aerobic Dehydrogenation of Ketones. *J. Am. Chem. Soc.* **2011**, 133, 37, 14566–14569. https://doi.org/10.1021/ja206575j

(4) Yusubov, M. S.; Soldatova, N. S.; Postnikov, P. S.; Valiev, R. R.; Yoshimura, A.; Wirth, T.; Nemykin, V. N.; Zhdankin, V. V. 2-Iodoxybenzoic Acid Ditriflate: The Most Powerful Hypervalent Iodine( v ) Oxidant. *Chem. Commun.* **2019**, *55* (54), 7760–7763. https://doi.org/10.1039/C9CC04203B.

(6) Bai, X.-F.; Xu, L.-W.; Zheng, L.-S.; Jiang, J.-X.; Lai, G.-Q.; Shang, J.-Y. Aromatic-Amide-Derived Olefins as a Springboard: Isomerization-Initiated Palladium-Catalyzed Hydrogenation of Olefins and Reductive Decarbonylation of Acyl Chlorides with Hydrosilane. *Chem. - Eur. J.* **2012**, *18* (26), 8174–8179. https://doi.org/10.1002/chem.201200039.

(7) Jiang, Q.; Guo, T.; Wang, Q.; Wu, P.; Yu, Z. Rhodium(I)-Catalyzed Arylation of β-Chloro Ketones and Related Derivatives through Domino Dehydrochlorination/ Conjugate Addition. *Adv. Synth. Catal.* **2013**, *355* (9), 1874–1880. https://doi.org/10.1002/adsc.201200821.

(8) Colbon, P.; Ruan, J.; Purdie, M.; Mulholland, K.; Xiao, J. Double Arylation of Allyl Alcohol via a One-Pot Heck Arylation–Isomerization–Acylation Cascade. *Org. Lett.* **2011**, *13* (20), 5456–5459. https://doi.org/10.1021/ol202144z.

(9) Shang, J.-Y.; Li, F.; Bai, X.-F.; Jiang, J.-X.; Yang, K.-F.; Lai, G.-Q.; Xu, L.-W. Malononitrile-Assisted Highly Chemoselective Bismuth Triflate Catalyzed Conjugate Reduction of α,β-Unsaturated Ketones. *Eur. J. Org. Chem.* **2012**, *2012* (14), 02809–02815. https://doi.org/10.1002/ejoc.201200020.

(10) Lei, C.; Zhu, D.; Tangcueco, V. I. T.; Zhou, J. S. Arylation of Aldehydes To Directly Form Ketones via Tandem Nickel Catalysis. *Org. Lett.* **2019**, *21* (15), 5817–5822. https://doi.org/10.1021/acs.orglett.9b01782.

(11) Wang, Q.-D.; Yang, J.-M.; Fang, D.; Ren, J.; Dong, B.; Zhou, B.; Zeng, B.-B. Chemoselective Cu(I) Catalyzed Bis(Pinacolato)Diboron Conjugate Addition and Reduction onto α,β-Unsaturated Carbonyl Compounds. *Tetrahedron Lett.* **2016**, *57* (24), 2587–2590. https://doi.org/10.1016/j.tetlet.2016.04.094.

(12) Hayes, J. F.; Shipman, M.; Twin, H. Multicomponent Reactions Involving 2-Methyleneaziridines: Rapid Synthesis of 1,3-Disubstituted Propanones *J. Org. Chem*. **2002**, 67, 935-942. https://doi.org/10.1021/jo016164v

(13) Boqiang D., Zhenfeng Z., Yangang L., Masashi S., Tsuneo I., Wanbin Z. Chemoselective Transfer Hydrogenation of α,β-Unsaturated Ketones Catalyzed by Pincer-Pd Complexes Using Alcohol as a Hydrogen Source. *Organic Letters* **2013** *15* (14), 3690-3693 https://doi.org/10.1021/ol401560q

(14) T. Laube; J. Schroder, R. Stehle, K. Seifert. Total synthesis of yahazunol, zonarone and isozonarone. *Tetrahedron* 58, 4299 (**2002**) https://doi.org/10.1016/S0040-4020(02)00346-0

(15) Billings, S. B.; Woerpel, K. A. Nucleophilic Substitution Reactions of Sulfur-Substituted Cyclohexanone Acetals: An Analysis of the Factors Controlling Stereoselectivity. *J. Org. Chem.* **2006**, *71* (14), 5171–5178. https://doi.org/10.1021/jo060077r.

(16) Nodes, W. J.; Nutt, D. R.; Chippindale, A. M.; Cobb, A. J. A. Enantioselective Intramolecular Michael Addition of Nitronates onto Conjugated Esters: Access to Cyclic γ-Amino Acids with up to Three Stereocenters. *J. Am. Chem. Soc.* **2009**, *131* (44), 16016–16017. https://doi.org/10.1021/ja9070915.

(17) Lu, Z.; Yang, Y.-Q. Catalyst-Free Photodriven Reduction of α-Haloketones with Hantzsch Ester. *Synthesis* **2019**, *51* (02), 508–515. https://doi.org/10.1055/s-0037-1610629.

(18) Porcheddu, A.; Delogu, F.; De Luca, L.; Fattuoni, C.; Colacino, E. Metal-Free Mechanochemical Oxidations in Ertalyte ® Jars. *Beilstein J. Org. Chem.* **2019**, *15*, 1786–1794. https://doi.org/10.3762/bjoc.15.172.

(19) Lator, A.; Gaillard, S.; Poater, A.; Renaud, J.-L. Iron-Catalyzed Chemoselective Reduction of α,β-Unsaturated Ketones. *Chem. - Eur. J.* **2018**, *24* (22), 5770–5774. https://doi.org/10.1002/chem.201800995.

(20) Milker, S.; Fink, M. J.; Oberleitner, N.; Ressmann, A. K.; Bornscheuer, U. T.; Mihovilovic, M. D.; Rudroff, F. Kinetic Modeling of an Enzymatic Redox Cascade In Vivo Reveals Bottlenecks Caused by Cofactors. *ChemCatChem* **2017**, *9* (17), 3420–3427. https://doi.org/10.1002/cctc.201700573.

(21) Jang, Y. K.; Magre, M.; Rueping, M. Chemoselective Luche-Type Reduction of α,β-Unsaturated Ketones by Magnesium Catalysis. *Org. Lett.* **2019**, *21* (20), 8349–8352. https://doi.org/10.1021/acs.orglett.9b03131.

(22) Kose, O.; Saito, S. Cross-Coupling Reaction of Alcohols for Carbon–Carbon Bond Formation Using Pincer-Type NHC/Palladium Catalysts. *Org Biomol Chem* **2010**, *8* (4), 896–900. https://doi.org/10.1039/B914618K.

(23) Roy, S. R.; Sau, S. C.; Mandal, S. K. Chemoselective Reduction of the Carbonyl Functionality through Hydrosilylation: Integrating Click Catalysis with Hydrosilylation in One Pot. *J. Org. Chem.* **2014**, *79* (19), 9150–9160. https://doi.org/10.1021/jo501505j.

(24) Hari Babu, M.; Ranjith Kumar, G.; Kant, R.; Sridhar Reddy, M. Ni-Catalyzed Regio- and Stereoselective Addition of Arylboronic Acids to Terminal Alkynes with a Directing Group Tether. *Chem. Commun.* **2017**, *53* (27), 3894–3897. https://doi.org/10.1039/C6CC10256E.

(25) Genç, S.; Arslan, B.; Gülcemal, S.; Günnaz, S.; Çetinkaya, B.; Gülcemal, D. Iridium(I)-Catalyzed C–C and C–N Bond Formation Reactions via the Borrowing Hydrogen Strategy. *J. Org. Chem.* **2019**, *84* (10), 6286–6297. https://doi.org/10.1021/acs.joc.9b00632.

(26) Johnston, A. J. S.; McLaughlin, M. G.; Reid, J. P.; Cook, M. J. NaH Mediated Isomerisation–Allylation Reaction of 1,3-Substituted Propenols. *Org. Biomol. Chem.* **2013**, *11* (44), 7662. https://doi.org/10.1039/c3ob41857j.

(27) Zhang, M.-J.; Li, H.-X.; Young, D. J.; Li, H.-Y.; Lang, J.-P. Reaction Condition Controlled Nickel( II )-Catalyzed C–C Cross-Coupling of Alcohols. *Org. Biomol. Chem.* **2019**, *17* (14), 3567–3574. https://doi.org/10.1039/C9OB00418A.

(28) Zhang G., Cheng J., Davis K., Bonifacio M. G., Zajaczkowski C., Practical and selective hydroboration of aldehydes and ketones in air catalysed by an iron(II) coordination polymer. *Green Chem*., **2019**, 21, 1114–1121 https://doi.org/10.1039/C9GC00078J

(29) *Zhan L., Shengming M.;* Studies on the Cu(I)-Catalyzed Regioselective *anti*-Carbometallation of Secondary Terminal Propargylic Alcohols. *J. Org. Chem.* **2006**, 71, 7, 2655–2660 https://doi.org/10.1021/jo0524021

(30) Bruneau-Voisine A.; Wang D.; Dorcet V.; Roisnel T.; Darcel C.; Sortaisa J.; *Org. Lett.* 2017, 19, 13, 3656–3659

(31) Wang, Y.; Kang, Q. Palladium-Catalyzed Allylic Esterification via C–C Bond Cleavage of a Secondary Homoallyl Alcohol. *Org. Lett.* **2014**, *16* (16), 4190–4193. https://doi.org/10.1021/ol501887a.

(32) Akai, S.; Hanada, R.; Fujiwara, N.; Kita, Y.; Egi, M. One-Pot Synthesis of Optically Active Allyl Esters via Lipase–Vanadium Combo Catalysis. *Org. Lett.* **2010**, *12* (21), 4900–4903. https://doi.org/10.1021/ol102053a.

(33) Jakhar, V. K.; Barman, M. Kr.; Nembenna, S. Aluminum Monohydride Catalyzed Selective Hydroboration of Carbonyl Compounds. *Org. Lett.* **2016**, *18* (18), 4710–4713. https://doi.org/10.1021/acs.orglett.6b02310.

(34) Rideau, E.; You, H.; Sidera, M.; Claridge, T. D. W.; Fletcher, S. P. Mechanistic Studies on a Cu-Catalyzed Asymmetric Allylic Alkylation with Cyclic Racemic Starting Materials. *J. Am. Chem. Soc.* **2017**, *139* (15), 5614–5624. https://doi.org/10.1021/jacs.7b02440.

(35) Tueting, D. R.; Echavarren, A. M.; Stille, J. K. Palladium Catalyzed Coupling of Organostannanes with Vinyl Epoxides. *Tetrahedron* **1989**, *45* (4), 979–992. https://doi.org/10.1016/0040-4020(89)80010-9.

(36) Xue, S.; Li, Y.; Han, K.; Yin, W.; Wang, M.; Guo, Q. Addition of Organozinc Species to Cyclic 1,3-Diene Monoepoxide. *Org. Lett.* **2002**, *4* (6), 905–907. https://doi.org/10.1021/ol017279o.

(37) Graton, J.; Wang, Z.; Brossard, A.-M.; Gonçalves Monteiro, D.; Le Questel, J.-Y.; Linclau, B. An Unexpected and Significantly Lower Hydrogen-Bond-Donating Capacity of Fluorohydrins Compared to Nonfluorinated Alcohols. *Angew. Chem. Int. Ed.* **2012**, *51* (25), 6176–6180. https://doi.org/10.1002/anie.201202059.

(38) Xu, Z.-B.; Qu, J. Hot Water-Promoted S $_N$ 1 Solvolysis Reactions of Allylic and Benzylic Alcohols. *Chem. - Eur. J.* **2013**, *19* (1), 314–323. https://doi.org/10.1002/chem.201202886.

(39) Tomita, R.; Mantani, K.; Hamasaki, A.; Ishida, T.; Tokunaga, M. Homogeneous Pd-Catalyzed Transformation of Terminal Alkenes into Primary Allylic Alcohols and Derivatives. *Chem. Eur. J.* **2014**, *20* (32), 9914–9917. https://doi.org/10.1002/chem.201403373.

(40) Kotani S.; Haschimoto, S.; Nakajima, M.; Chiral phosphine oxide BINAPO as a Lewis base catalyst for asymmetric allylation and aldol reaction of trichlorosilyl compounds *Tetrahedron*, 2007, 63 3122–3132 https://doi.org/10.1016/j.tet.2007.02.014

(41) Sai, M.; Yamamoto, H.; Chiral Brønsted Acid as a True Catalyst: Asymmetric Mukaiyama Aldol and Hosomi–Sakurai Allylation Reactions*J. Am. Chem. Soc.* 2015, 137, 22, 7091–7094 https://doi.org/10.1021/jacs.5b04168

(42) Denmark, S.; Stavenger, R.; Wong, K.; Asymmetric aldol additions catalyzed by chiral phosphoramides: Electronic effects of the aldehyde component *Tetrahedron* 1998, 54, 10389-10402 https://doi.org/10.1016/S0040-4020(98)00493-1

(43) Schetter, B; Ziemer, B.; Schnakenburg, G.; Mahrwald, R.; Tetranuclear BINOL–Titanium Complex in Selective Direct Aldol Additions. *J. Org. Chem.* **2008**, 73, 3, 813–819. https://doi.org/10.1021/jo7014054

(44) Ghatak, T.; Makarov, K.; Fridman, N.; Eisen, M. S. Catalytic Regeneration of a Th–H Bond from a Th–O Bond through a Mild and Chemoselective Carbonyl Hydroboration. *Chem. Commun.* **2018**, *54* (78), 11001–11004. https://doi.org/10.1039/C8CC05030A.

(45) Gaussian 16, Revision A.03, Frisch, M. J.; Trucks, G. W.; Schlegel, H. B.; Scuseria, G. E.; Robb, M. A.; Cheeseman, J. R.; Scalmani, G.; Barone, V.; Petersson, G. A.; Nakatsuji, H.; Li, X.; Caricato, M.; Marenich, A. V.; Bloino, J.; Janesko, B. G.; Gomperts, R.; Mennucci, B.; Hratchian, H. P.; Ortiz, J. V.; Izmaylov, A. F.; Sonnenberg, J. L.; Williams-Young, D.; Ding, F.; Lipparini, F.; Egidi, F.; Goings, J.; Peng, B.; Petrone, A.; Henderson, T.; Ranasinghe, D.; Zakrzewski, V. G.; Gao, J.; Rega, N.; Zheng, G.; Liang, W.; Hada, M.; Ehara, M.; Toyota, K.; Fukuda, R.; Hasegawa, J.; Ishida, M.; Nakajima, T.; Honda, Y.; Kitao, O.; Nakai, H.; Vreven, T.; Throssell, K.; Montgomery, J. A., Jr.; Peralta, J. E.; Ogliaro, F.; Bearpark, M. J.; Heyd, J. J.; Brothers, E. N.; Kudin, K. N.; Staroverov, V. N.; Keith, T. A.; Kobayashi, R.; Normand, J.; Raghavachari, K.; Rendell, A. P.; Burant, J. C.; Iyengar, S. S.; Tomasi, J.; Cossi, M.; Millam, J. M.; Klene, M.; Adamo, C.; Cammi, R.; Ochterski, J. W.; Martin, R. L.; Morokuma, K.; Farkas, O.; Foresman, J. B.; Fox, D. J. Gaussian, Inc., Wallingford CT, 2016.

(46) a) Becke, A. D. *J. Chem. Phys.* **1993**, *98*, 5648. b) Lee, C.; Yang, W.; Parr, R. G. *Phys. Rev. B. Condens. Matter*. **1988**, *37*, 785. c) Stephens, P. J.; Devlin, F. J.; Chabalowski, C. F.; Frisch, M. J. *J. Phys. Chem.* **1994**, *98*, 11623. d) Grimme, S.; Antony, J.; Ehrlich, S.; Krieg, H. *J. Chem. Phys.* **2010**, *132*, 154104.

(47) Fallon, B. J.; Derat, E.; Amatore, M.; Aubert, C.; Chemla, F.; Ferreira, F.; Perez-Luna, A.; Petit, M. *J. Am. Chem. Soc.* **2015**, *137*, 2448-2451.

(48) - Program available at https://github.com/jaimergp/easymecp

(49) 2- Jeremy N. Harvey, Massimiliano Aschi, Helmut Schwarz & Wolfram Koch, Theoretical Chemistry Accounts volume 99, pages 95–99(1998)

(50) CYLview, 1.0b; Legault, C. Y., Université de Sherbrooke, **2009** (http://www.cylview.org).

(51) Bergamaschi, E.; Beltran, F.; Teskey, C. J. Visible-Light Controlled Divergent Catalysis Using a Bench-Stable Cobalt(I) Hydride Complex. *Chem. Eur. J.* **2020**, *26* (23), 5180–5184. https://doi.org/10.1002/chem.202000410.
